# Continuous Flow Preparation of Benzylic Sodium Organometallics

**DOI:** 10.1002/anie.202203807

**Published:** 2022-05-11

**Authors:** Johannes H. Harenberg, Rajasekar Reddy Annapureddy, Konstantin Karaghiosoff, Paul Knochel

**Affiliations:** ^1^ Department Chemie Ludwig-Maximilians-Universität München Butenandtstraße 5–13, Haus F 81377 München Germany

**Keywords:** Cross-Coupling, Flow Chemistry, Metalation, Ring Openings, Sodium

## Abstract

We report a lateral sodiation of alkyl(hetero)arenes using on‐demand generated hexane‐soluble (2‐ethylhexyl)sodium (**1**) in the presence of TMEDA. (2‐Ethylhexyl)sodium (**1**) is prepared via a sodium packed‐bed reactor and used for metalations at ambient temperature in batch as well as in continuous flow. The resulting benzylic sodium species are subsequently trapped with various electrophiles including carbonyl compounds, epoxides, oxetane, allyl/benzyl chlorides, alkyl halides and alkyl tosylates. Wurtz‐type couplings with secondary alkyl halides and tosylates proceed under complete inversion of stereochemistry. Furthermore, the utility of this lateral sodiation is demonstrated in the synthesis of pharmaceutical relevant compounds. Thus, fingolimod is prepared from *p*‐xylene applying the lateral sodiation twice. In addition, 7‐fold isotopically labeled salmeterol‐*d*_7_ and fenpiprane as well as precursors to super linear alkylbenzene (SLAB) surfactants are prepared.

## Graphical abstract

A lateral sodiation of alkyl(hetero)arenes using on‐demand generated (2‐ethylhexyl)sodium in the presence of TMEDA is reported. Various electrophiles were used for trapping of the benzyl sodium species generated in continuous flow as well as in batch. The continuous flow metalation was scaled‐up using an in‐line quench. The method was used in the synthesis of several industrial relevant compounds.

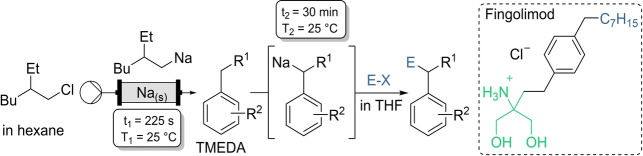

## Introduction

**Scheme 1:**
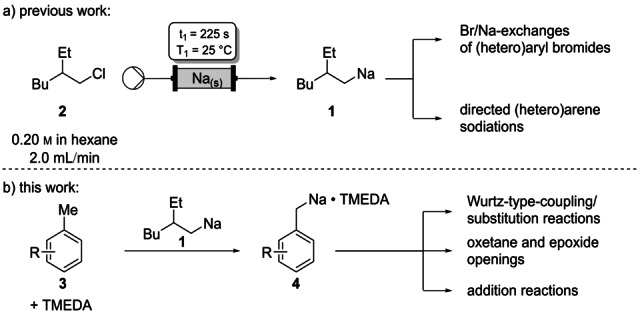
a) On‐demand continuous flow generation of (2‐ethylhexyl)sodium (**1**) and subsequent in‐line Br/Na‐exchange and directed metalation. b) Lateral metalations of methylarenes using in continuous flow prepared (2‐ethylhexyl)sodium (**1**) and subsequent use of the benzylic sodiums for additive free Wurtz‐type‐couplings, oxetane, epoxide openings and addition to carbonyl derivatives.

Among all alkali metal organometallics, lithium compounds have found most applications in organic chemistry.^[1]^ However, the increased use of lithium in battery technologies leads to unpredictable prices^[2]^ and drives the search for using alternative alkali metals for applications in fine organic synthesis.^[3]^ Although sodium is 1200 times more abundant in the earth's crust^[4]^ and more environmentally friendly,^[5]^ it is underexploited in organic chemistry.^[6]^ This is a result of the high reactivity of sodium organometallics as well as the poor solubility of most organosodiums.^[7]^ Recently, we have reported that the reactivity of organosodiums can be well tuned by continuous flow techniques.^[8]^ Also, the findings that (2‐ethylhexyl)sodium (**1**) is easily prepared from 3‐(chloromethyl)heptane (**2**) using a sodium packed‐bed reactor^[9]^ and uniquely soluble in *n*‐hexane,^[10]^ make this new reagent ideal for preparing various organosodiums either by a Br/Na‐exchange or more attractively by directed sodiation of arenes and heteroarenes (Scheme 1a).^[11]^ Since the lateral metalation of various methyl‐ and alkyl‐substituted (hetero)arenes has proven to be an excellent method for preparing benzylic alkali metal reagents,^[12]^ we have envisioned to use the new base **1** for the lateral metalation of various substituted arenes of type **3**. Herein, we report the successful preparation of various benzylic sodium species of type **4** as well as their reactivity in nucleophilic substitutions including Wurtz‐type couplings,^[13]^ epoxide^[14]^ and oxetane openings^[15]^ and additions to carbonyl electrophiles (Scheme 1b).

**Scheme 2:**
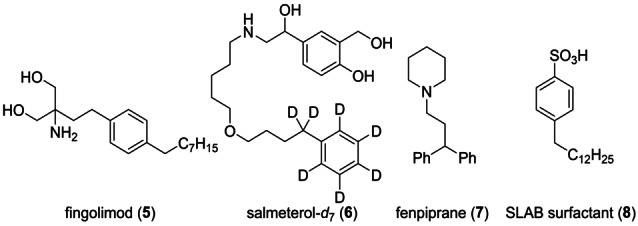
Pharmaceuticals and super linear alkylbenzene surfactant (SLAB) potentially prepared using benzylic organometallics.

Such benzylic organometallics are potentially important reagents for the synthesis of drug targets such as the immunomodulator fingolimod (**5**), which is approved for the treatment of multiple sclerosis,^[16]^ the *β*_2_ adrenoreceptor agonist salmeterol (**6**) used for the therapy of the asthma disease^[17]^ or other pharmaceuticals such as fenpiprane (**7**). Furthermore, the procedure might enable a straightforward and scalable synthesis of super linear alkylbenzene (SLAB) surfactants like **8**.^[18]^ Herein, we demonstrate that benzylic sodium intermediates prepared by this continuous flow method are well suited for the preparation of **5** to **8** (Scheme 2).

## Results and Discussion

**Scheme 3:**
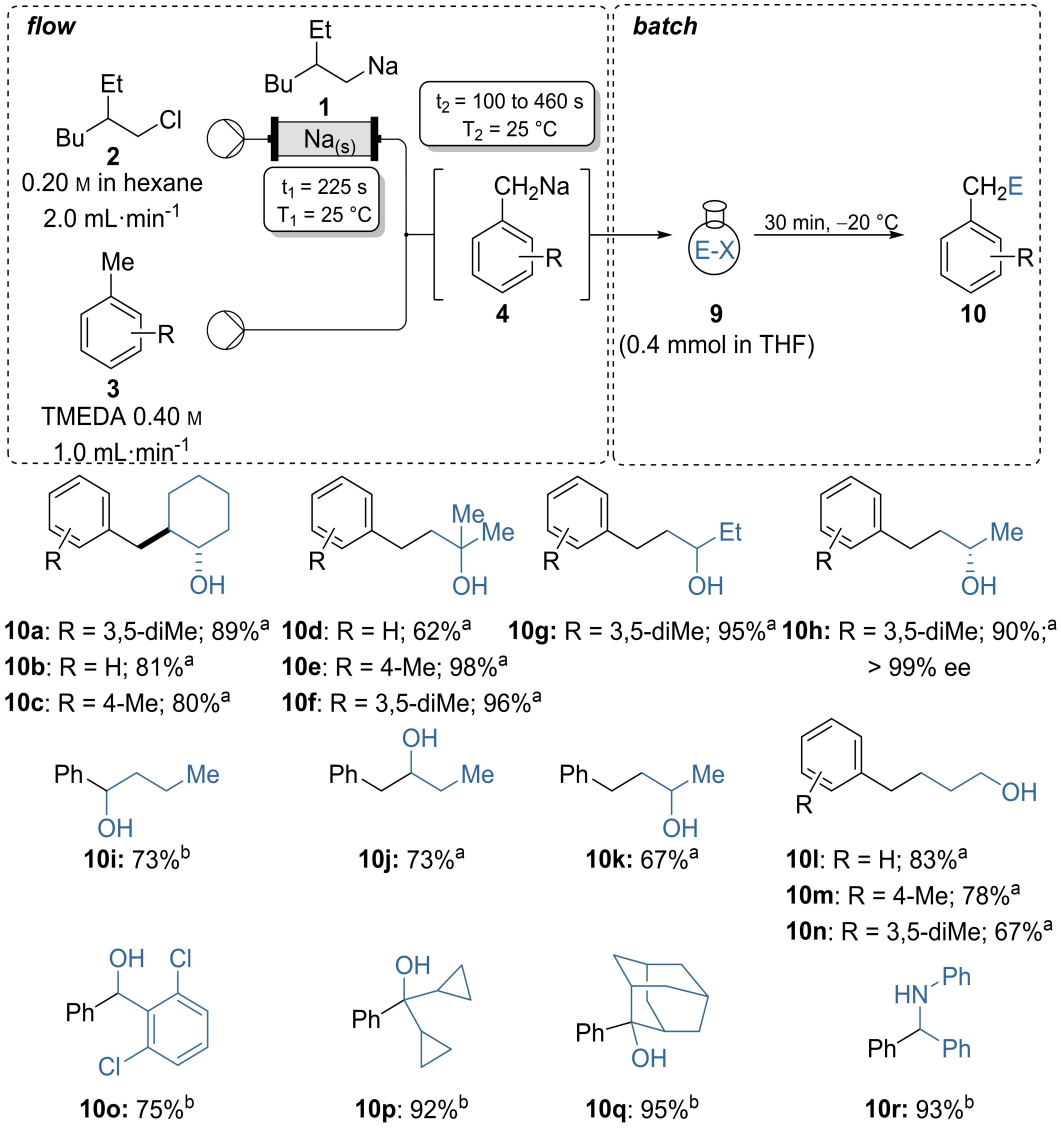
On‐demand preparation of alkyl sodium reagent **1** from alkyl chloride **2** followed by inline sodiations on methyl aryls of type **3** or benzene **3 d** leading to benzylic sodiums of type **4** and subsequent batch quench with electrophiles of type **9** leading to products of type **10**. Isolated yields of analytically pure products. [a] *t*_2_=460 s; [b] *t*_2_=100 s.

Thus, in preliminary experiments, we have generated (2‐ethylhexyl)sodium (**1**) as a 0.15 m solution in *n*‐hexane by pumping alkyl chloride **2** (0.20 m in hexane, 2.0 mL min^−1^) through the previously developed sodium packed‐bed reactor.^[11]^ Mixing the stream of **1** (2.0 mL min^−1^) with a 0.4 m solution of *N,N,N*′*,N*′*‐*tetramethylethylenediamine (TMEDA) in neat mesitylene (**3 a**) (1.0 mL min^−1^) in a T‐shaped mixer led to an optimum metalation within 460 s at 25 °C producing 3,5‐dimethylbenzylsodium (**4 a**). Even though the metalation proceeded without TMEDA, its presence accelerated the sodiation and increased dramatically the solubility of the resulting benzylic intermediates.[^19^, ^20^] A subsequent batch reaction with cyclohexene oxide (**9 a**) produced the ring opening alcohol product **10 a** in 89 % isolated yield (Scheme 3).^[21]^

These optimized conditions were extended to continuous flow sodiations of other commonly used methyl substituted arenes, such as toluene (**3 b**) and *p*‐xylene (**3 c**). Batch quench of the resulting benzylic sodium species (**4 b**, **4 c**) with substituted epoxides led to the secondary and tertiary alcohols **10 b**–**10 g** in 62–98 % isolated yield with a nucleophilic attack on the sterically less hindered epoxide carbon. Reaction of **4 a** with the enantiopure (*S*)‐2‐methyloxirane (**9 d**) gave the alcohol **10 h** (90 % yield,>99 % ee) with complete retention of stereochemistry.

In addition to the lateral metalation of methyl substituted arenes, it was possible to sodiate neat benzene (**3 d**) in the presence of TMEDA (0.4 m) using the same flow rates as for the lateral metalation, but a shorter coil reactor resulting in a residence time of 100 s at 25 °C. Thus, quenching of phenylsodium (**4 d**) with butyraldehyde (**9 e**) led to alcohol **10 i** in 73 % yield. In fact, our method allowed the introduction of an alcohol function in α, β, γ, and δ‐ position to the aromatic ring. Thus, the 1,2‐addition of benzylsodium (**4 b**) to propionaldehyde (**9 f**) and the Lewis acid free ring openings of epoxide **9 g** and unsubstituted oxetane (**9 h**) proceeded smoothly and led to the phenylbutanols **10 i**–**10 l** (67–83 % yield). The oxetane opening products of mesitylene and *p*‐xylene **10 m**, **10 n** were obtained in 67–78 % yield. Batch quench of phenylsodium (**4 d**) with aldehyde, ketone and imine electrophiles led to the formation of the desired products **10 o**–**10 r** in 75–95 % yield.

**Scheme 4:**
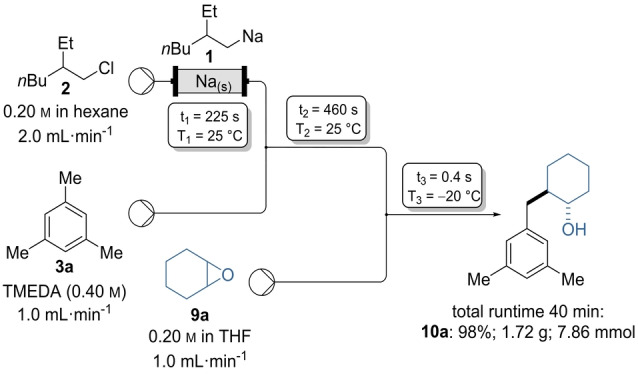
20‐Fold scale‐up of the lateral metalation of mesitylene (**3 a**), using (2‐ethylhexyl)sodium (**1**) as base and cyclohexene oxide (**9 a**) as electrophile, applying in‐line quenching conditions.

Scalability of this synthesis was shown by transferring the reaction sequence into a continuous flow set‐up using a subsequent in‐line quench. Thus, after formation of the solution **4 a** in a coiled tube reactor (*t*_2_=460s, *T*_2_=25 °C) the solution was mixed in a second T‐shaped mixer with a solution of **9 a** in THF (0.20 m, 1.0 mL min^−1^). Increasing the runtime to 40 min led to a 20‐fold scale up (8.0 mmol) and the desired product **10 a** in 98 % yield (1.72 g, 7.86 mmol, Scheme 4).

**Scheme 5:**
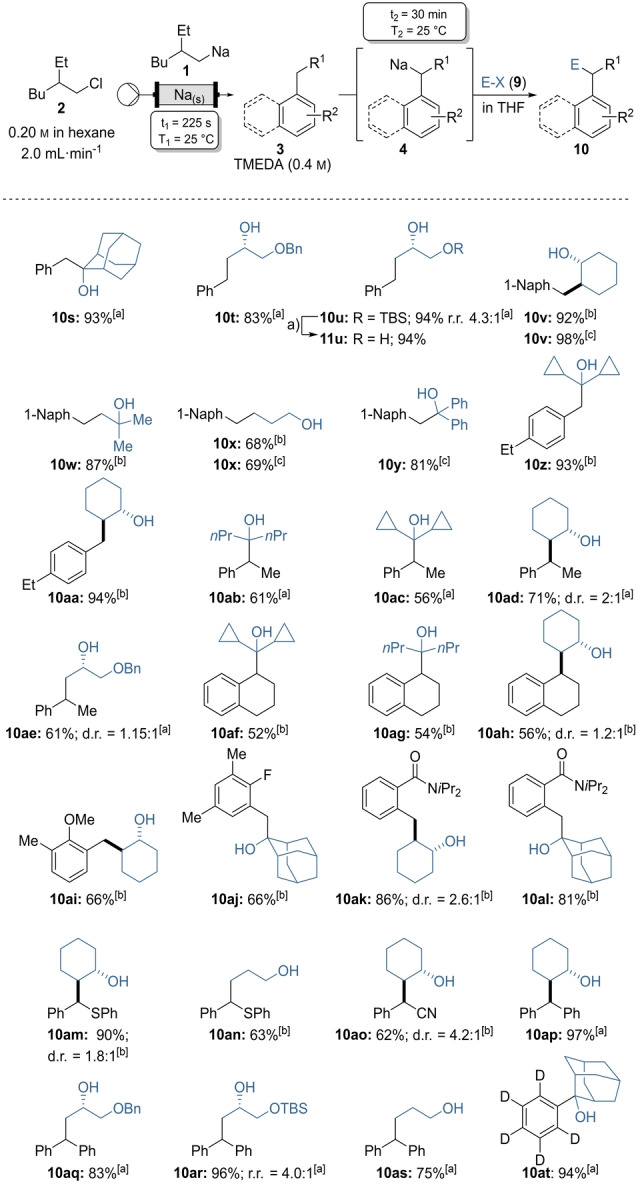
On‐demand preparation of alkylsodium reagent **1** from alkyl chloride **2** followed by batch sodiations on substituted aryls of type **3** or benzene‐*d*_6_ (**3 n**) leading to benzylic sodiums of type **4** and subsequent quench with electrophiles of type **9** leading to products of type **10**. Yield of analytically pure products. [a] Arene **3** was used neat with TMEDA (2.0 equiv) and electrophile **9** (1.0 equiv in THF). [b] Arene **3** in hexane (1.0 equiv) with TMEDA (2.0 equiv) and electrophile **9** (2.0 equiv. in THF). [c] Arene **3** in hexane (2.5 equiv) with TMEDA (2.0 equiv) and electrophile **9** (1.0 equiv in THF) a) Deprotection was achieved with TBAF in THF 94 % yield.

The moderate solubility of more complex benzylic sodium derivatives in hexane led us to develop an alternative procedure, in which the sodiation step took place in batch. In preliminary experiments, addition of on‐demand generated solution of **1** for 2 min to a mixture of TMEDA (0.8 mmol) in toluene (**3 b**) in batch gave benzylsodium (**4 b**). After quenching **4 b** with adamantanone (**9 k**) or the protected epoxy‐propanols **9 m** and **9 n** the desired alcohol products **10 s**–**10 u** were obtained in 83–94 % yield. In case of **10 u**, a silyl migration was observed, however a deprotection with TBAF in THF gave the corresponding 1,2‐diol **11 u** in 94 % yield (Scheme 5).

In all above described experiments the methyl arenes of type **3** were used in large excess (solvent!). For more expensive or solid substituted arenes, we designed another more general procedure. Thus, the solution of **1** prepared in continuous flow was added to a mixture of 1‐methylnaphthalene (**3 e**, 1.0 equiv) and TMEDA (2.0 equiv) in hexane. Stirring for 30 min at 25 °C resulted in benzylsodium **4 e**. Batch quench with epoxides, oxetane or ketones gave the alcohols **10 v** ^[22]^–**10 x** (68–92 % yield). Similar results were obtained using **3 e** in excess (2.5 equiv) and the electrophiles (1.0 equiv) as limiting reagent, leading to **10 v** (98 % yield), **10 x** (69 % yield), and **10 y** (81 % yield). The procedure was used for the metalation of 1‐ethyl‐4‐methylbenzene (**3 f**) which was selectively sodiated at the methyl group. After addition of electrophiles the expected products **10 z** and **10 aa** were obtained in 93–94 % yield. Substituted alkylarenes such as ethylbenzene (**3 g**) and tetralin (**3 h**) were similarly sodiated and reaction of the secondary benzylsodium species **4 g** and **4 h** with ketones and epoxides resulted in alcohols **10 ab**–**10 ah** (52–71 % yield).

Lateral metalation of methylarenes substituted by halide atoms or methoxy‐ or amide‐functionalities on the aromatic ring (**3 i**–**3 k**) and quenching with electrophiles led to the functionalized alcohols **10 ai**–**10 al** (66–86 % yield).^[23]^ This sodiation procedure was extended to the metalation of benzylic positions adjacent to functional groups. Thus, benzyl(phenyl)sulfane (**3 l**) and phenylacetonitrile (**3 m**) were converted into sulfides and nitriles **10 am**–**10 ao** in 62–90 % yield.

Diphenylmethane **3 n** was sodiated in a hexane solution or neat.^[24]^ The resulting benzhydrylsodium **4 n** was an excellent nucleophile for the Lewis acid free ring opening of epoxides and oxetane (**9 h**) and gave the desired alcohols **10 ap**–**10 as** in 75–97 % yield. The sodiation of benzene (**3 d**) in continuous flow gave phenylsodium (**4 d**) in high yields, we have extended this metalation to benzene‐*d*_6_ (**3 o**).^[25]^ Indeed, the sodiation of neat **3 o** in the presence of TMEDA proceeded smoothly and quenching with adamantanone (**9 k**) gave the desired penta‐deuterated tertiary alcohol **10 at** in 94 % yield.

**Scheme 6:**
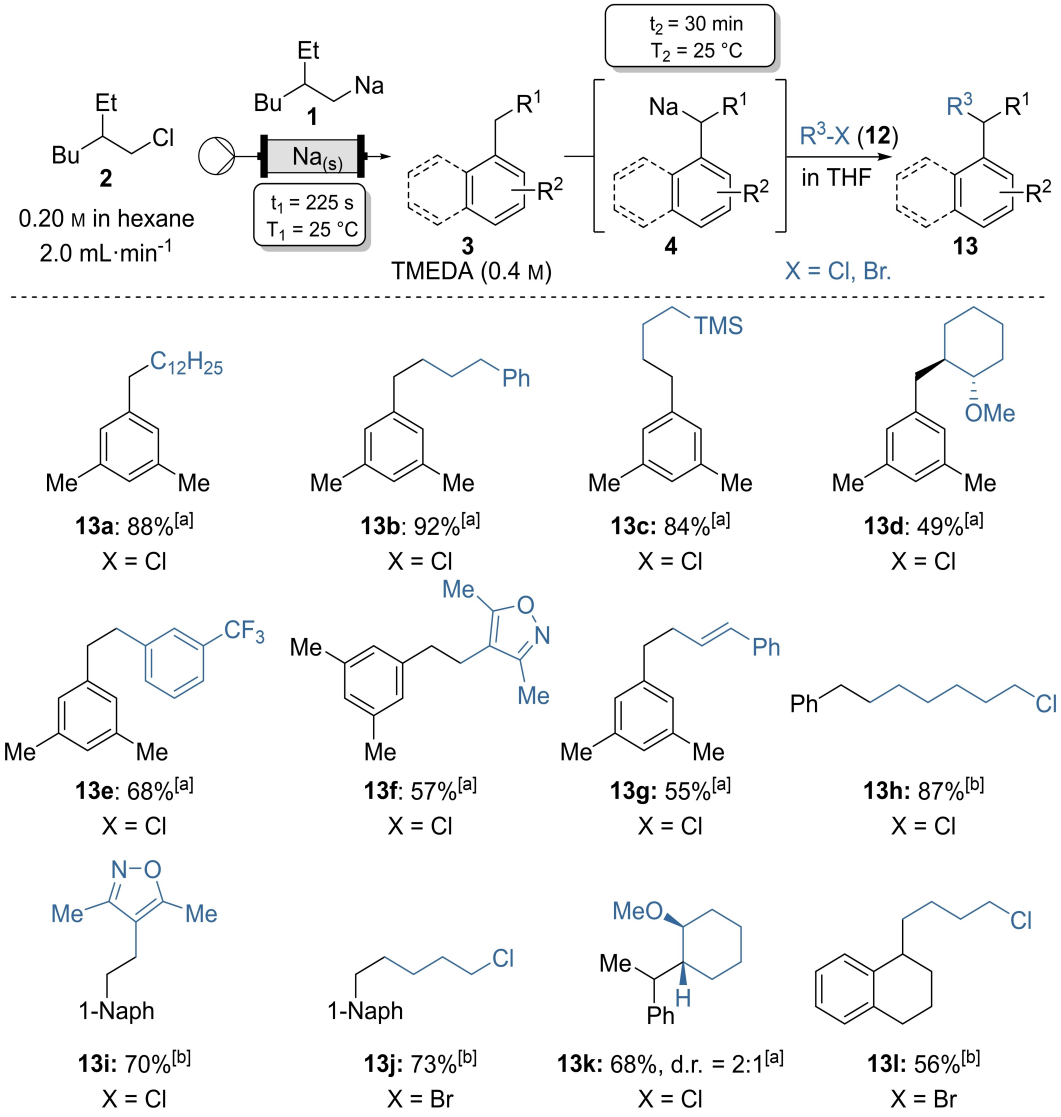
On‐demand preparation of alkylsodium reagent **1** from alkyl chloride **2** followed by batch sodiations on substituted arenes of type **3** leading to benzylic sodiums of type **4** and subsequent quench with electrophiles of type **12** leading to products of type **13**. Yield of analytically pure products. [a] Arene **3** was used neat with TMEDA (0.8 mmol) and alkyl halides of type **12** (0.4 mmol in THF). [b] **3** in hexane (0.4 mmol) with TMEDA (0.8 mmol) and **12** (0.8 mmol in THF).

Carbon–carbon bond formation is a major goal of synthetic organometallic chemistry. Most cross‐couplings performed required expensive transition metal catalysts and ligands.^[26]^ On another hand, the Wurtz‐type‐coupling between an alkali metal organyl and an alkyl halide proceeded without transition metal catalysts and is therefore an attractive alternative to conventional cross‐couplings. Nevertheless, Wurtz‐type couplings are scarce. This is most likely due to their low selectivity and the fact that most reactions of this type are limited to alkyl iodides.[^13a^, ^27^] We have found that benzylsodiums of type **4** are excellent nucleophiles for such cross‐couplings and are capable of forming Csp^3^−Csp^3^ bonds with readily accessible electrophiles such as alkyl‐, allyl‐, and benzyl chlorides. Thus, metalation in continuous flow as well as batch sodiation of neat mesitylene (**3 a**) in the presence of TMEDA with on‐demand generated **1**, led to **4 a**. Subsequent addition of primary alkyl chlorides **12 a**–**12 c** in THF at −20 °C and stirring of the mixture at 25 °C overnight gave the corresponding alkylbenzenes **13 a**–**13 c** in 84–92 % yield (Scheme 6). Quenching with the secondary *cis*‐1‐chloro‐2‐methoxycyclohexane (**12 d**) gave the expected substitution on the alkyl chloride with inversion of stereochemistry, indicating a S_N_2‐type reaction mechanism, and led to the *trans*‐product **13 d** in 49 % yield. Reaction of **4 a** with the (hetero)benzyl chlorides **12 e** and **12 f** led to the 1,2‐diarylethanes **13 e** and **13 f** (57–68 %). Allylation with *E*‐cinnamyl chloride (**12 g**) gave the S_N_2‐type product and maintained *E*‐configuration in the product (**13 g**; 55 %). Interestingly, allyl bromides gave significantly lower yields and led to a mixture of S_N_2‐ and S_N_2′‐type products. Benzylsodium (**4 b**) and the sodiated intermediate of 1‐methylnaphthalene (**3 e**) underwent Wurtz‐type couplings with benzyl chloride **12 e** and alkyl dihalides **12 h** and **12 i**. Thus, the monosubstituted products (**13 h**–**13 j**; 73–87 %) were obtained. In case of the 1‐bromo‐3‐chlorobutane (**12 i**) the bromide was selectively substituted over the chloride. Similarly substituted products (**13 k**, **13 l**; 56–68 %) were obtained from secondary benzylsodium organometallics **4 g** and **4 h**. Stereo‐inversion was observed for the reaction between **4 g** and **12 d**, leading to the formation of a C−C‐bond between two secondary carbons (**13 k**).

**Scheme 7:**
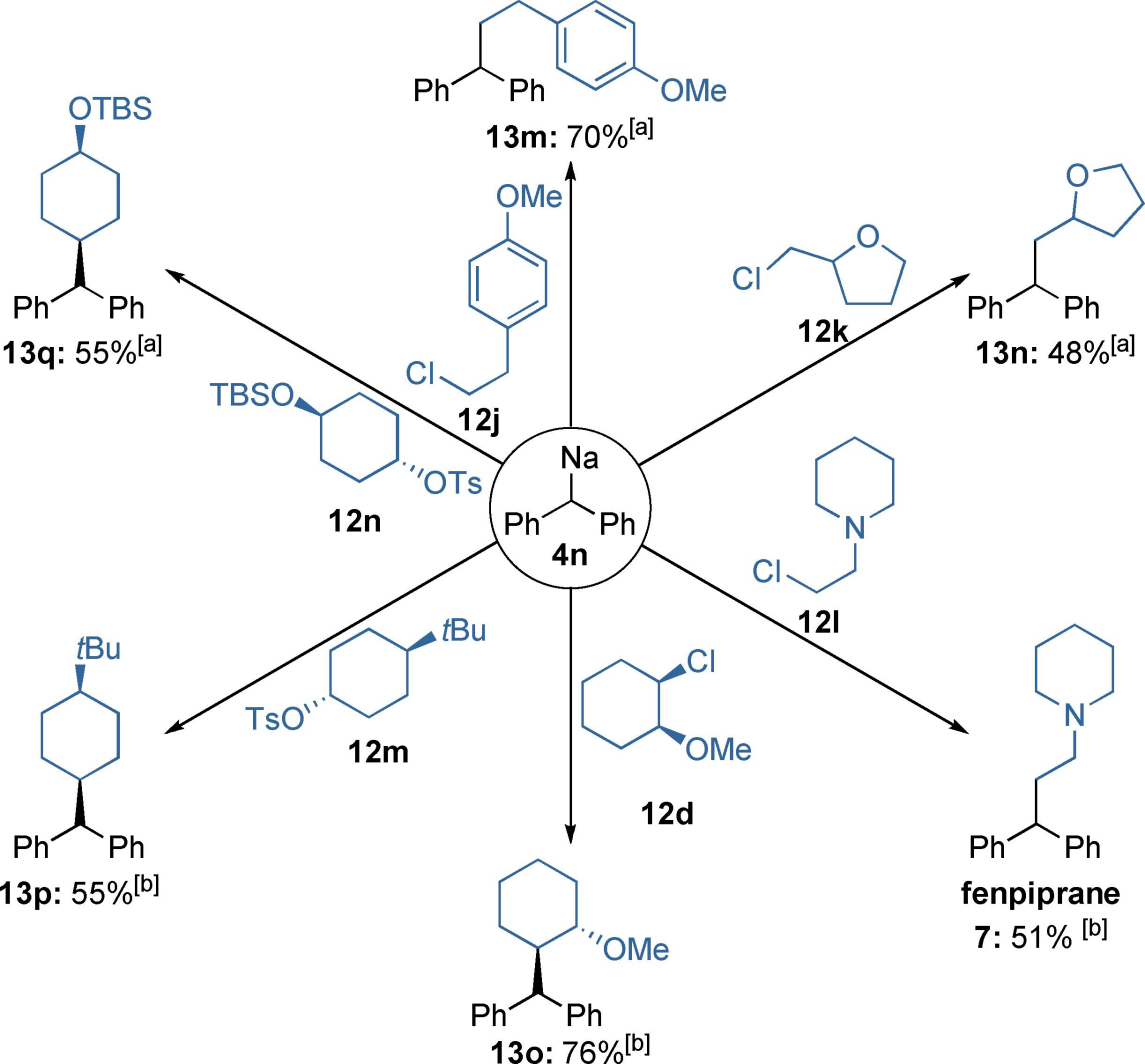
Wurtz‐type reactions of benzhydrylsodium (**4 n**) with electrophiles of type **12**. Isolated yields of analytically pure products. [a] Diphenylmethane (**3 n**) was used neat with TMEDA (0.8 mmol) and electrophiles **12** as limiting reagent (0.4 mmol in THF). [b] Diphenylmethane (**3 n**) was used as limiting reagent in hexane (0.4 mmol) with TMEDA (0.8 mmol) and electrophiles **12** (0.8 mmol in THF).

Benzhydrylsodium (**4 n**) was quenched with ether bearing primary alkyl chlorides **12 j** and **12 k** to give the functionalized Wurtz‐type products **13 m** and **13 n** in 48–70 % isolated yield (Scheme 7). Substitution reaction with chloroethylpiperidine **12 l** led to the spasmolytic and antiallergic drug fenpiprane **7** (51 % yield).^[28]^ Reaction of **4 n** with the enantiopure alkyl chloride **12 d** resulted in the *trans*‐cyclohexane **13 o** (76 %) with full inversion of stereochemistry.^[29]^

Secondary tosylates are also good substrates. We obtained the 1,4‐disubstituted cyclohexanes **13 p** and **13 q** in 55 % yield, solely as the *cis* isomers,^[30]^ from the reaction of the *trans*‐1,4‐disubstituted cyclohexane tosylates **12 m** and **12 n** with **4 n**.^[31]^

**Scheme 8:**
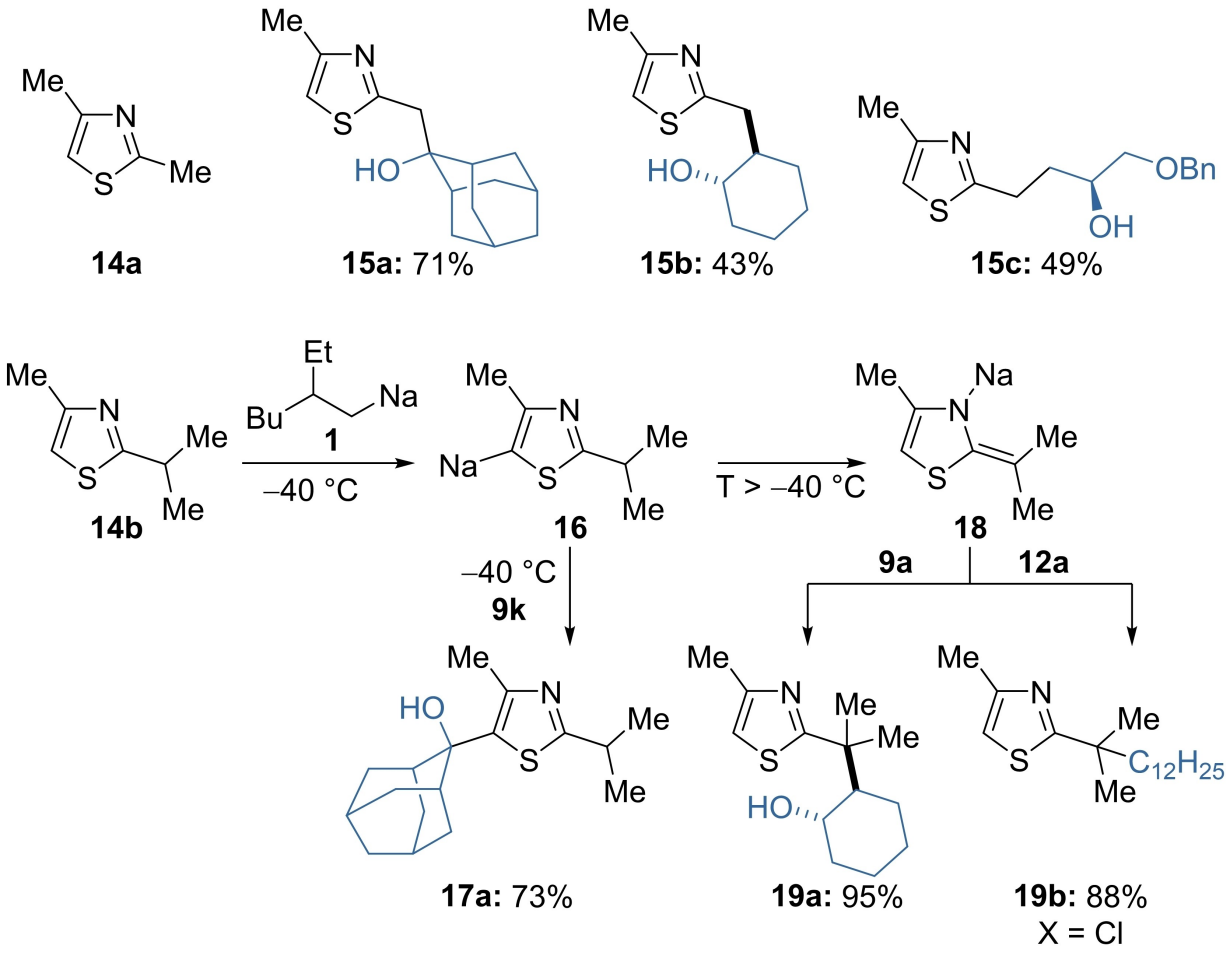
On‐demand preparation of alkyl sodium reagent **1** from alkyl chloride **2** followed by batch sodiations on substituted thiazoles of type **14**, leading to sodium species of type **16** and upon migration to **18**. A subsequent quench with electrophiles of type **9** and **12** led to products of type **15**, **17** and **19**. Yields of analytically pure products.

The treatment of 2,4‐dimethylthiazole (**14 a**) with (2‐ethylhexyl)sodium (**1**) at −40 °C for 30 min enabled a selective lateral sodiation at the methyl group in position 2. Quenching with ketone **9 k** and epoxides **9 a** and **9 m** provided the expected thiazoles **15 a**–**c** in 43–71 % yield (Scheme 8).^[32]^ Interestingly, in the case of the more sterically hindered 2‐isopropyl‐4‐methylthiazole **14 b**, we observed a kinetically controlled ring sodiation at position 5 at −40 °C affording the sodiated thiazole **16**. Quenching with adamantanone (**9 k**) provided the alcohol **17 a** in 73 % yield. The sodiated thiazole **16** isomerized at higher temperatures to the more stable lateral metalation derivative **18** with extensive decomposition in the absence of an electrophile.^[33]^ However, in the presence of cyclohexene oxide (**9 a**) or dodecyl chloride (**12 a**), the expected products **19 a** and **19 b** were obtained in 88–95 % yield.

**Scheme 9:**
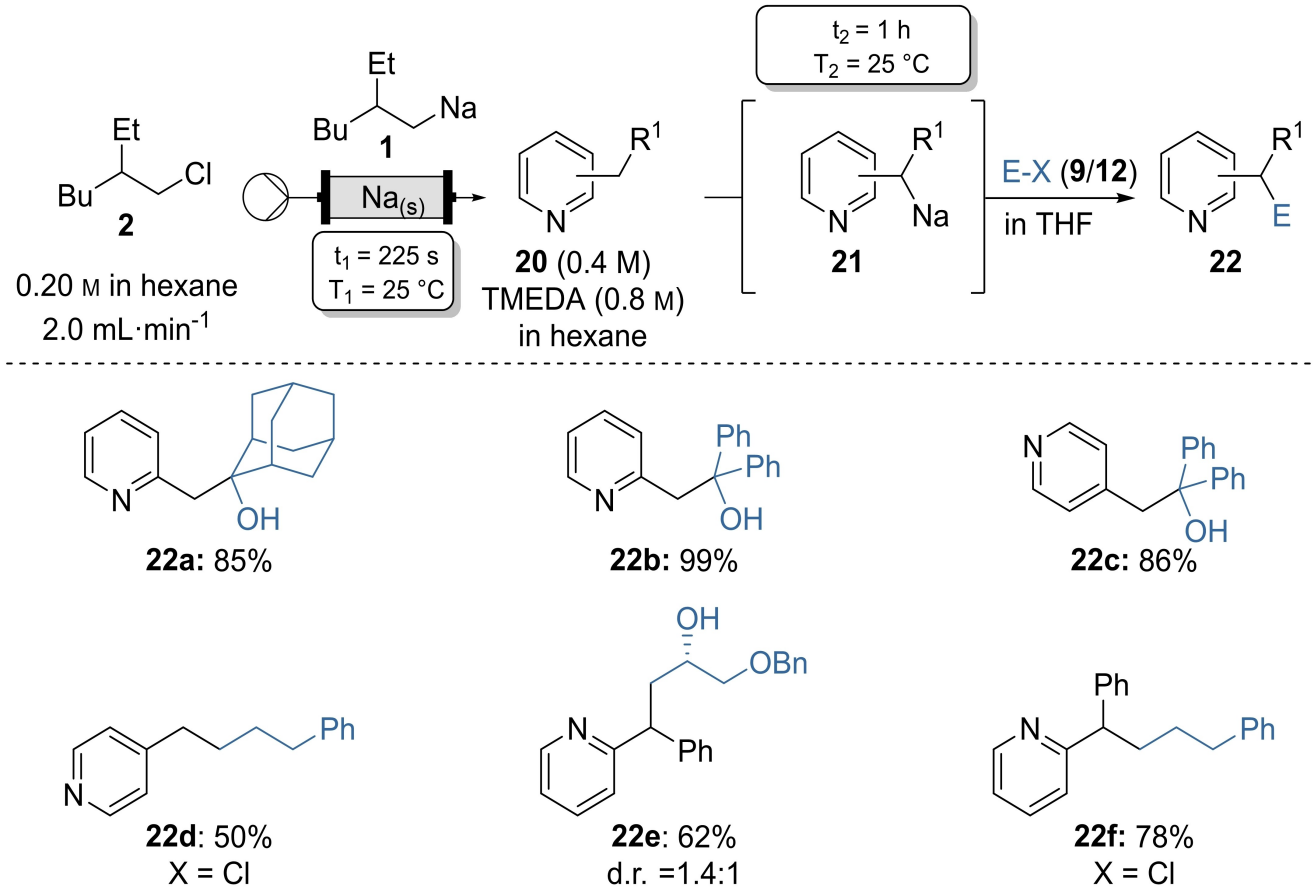
On‐demand preparation of alkylsodium reagent **1** from alkyl chloride **2** followed by batch sodiations on substituted arenes of type **20** leading to heterobenzylic sodiums of type **21** and subsequent quench with electrophiles of type **9** or **12** leading to products of type **22**. Yields of analytically pure products.

Similarly, six‐membered N‐heterocycles were sodiated at the benzylic position at 25 °C within 1 h. Thus, 2‐ and 4‐picoline (**20 a**–**20 b**) gave the corresponding metalated species **21 a**–**21 b** which upon quench with ketones **9 k** and **9 o** and alkyl chloride **12 b** gave the desired heterocyclic products **22 a**–**22 d** in 50–99 % yield (Scheme 9). 2‐Benzylpyridine (**20 c**) underwent after metalation with (2‐ethylhexyl)sodium (**1**) a ring opening reaction with the chiral epoxide **9 m** to give protected glycol **22 e** in 62 % yield (d.r.=1.4 : 1). Wurtz‐type coupling of the same benzylic organosodium with (3‐chloropropyl)benzene (**12 b**) gave the substituted pyridine **22 f** in 78 % yield.

**Scheme 10:**
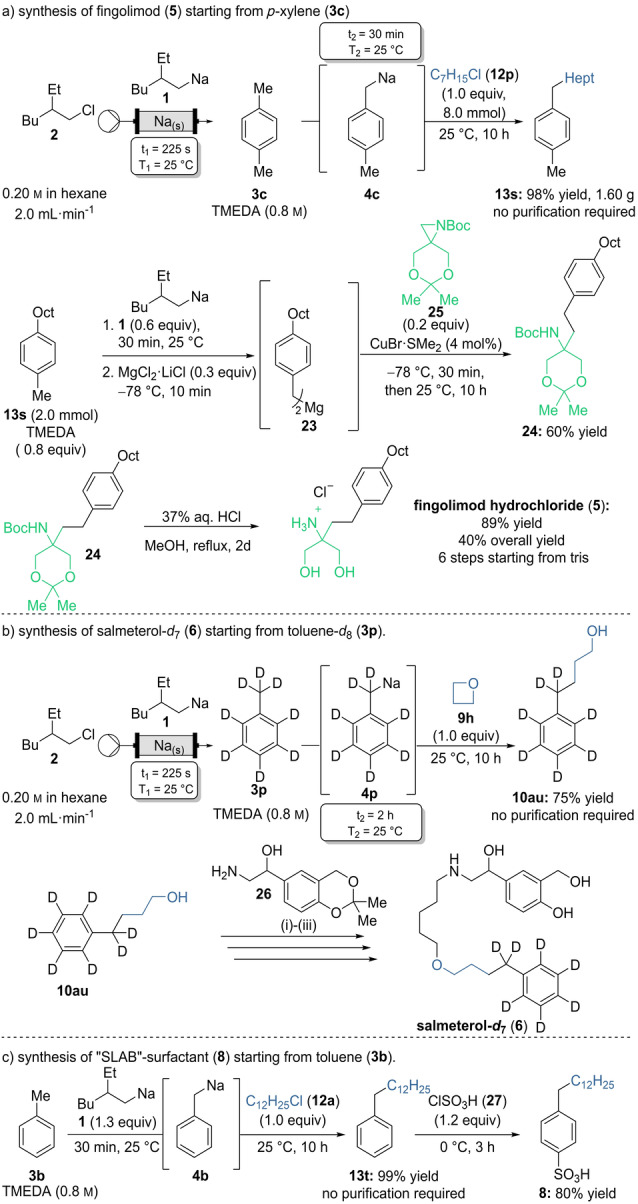
Applications of the lateral sodiation procedure: a) Synthesis of fingolimod hydrochloride (**5**). b) Synthesis of salmeterol‐*d*_7_ (**6**); i) NaH, 1,6‐dibromohexane, NBu_4_Br, THF, 25 °C, 64 %; ii) **26**, KI, DMF, 40 °C, 50 %; iii) HCl, THF, 25 °C, 6 h, 77 %. c) Synthesis of the SLAB‐surfactant **8**.

The utility of the lateral sodiation procedure was demonstrated by the straightforward synthesis of industrial relevant compounds (Scheme 10). Fingolimod (**5**) and salmeterol (**6**) are among the 100 best selling drugs, both exceeding retail sales of 2 billion USD in 2020.^[34]^ The multiple scleroses therapeutics fingolimod (**5**) has previously been synthesized by various approaches.[^16b^, ^16c^, ^16d^] Most of them require transition metal catalyzed cross‐coupling reaction to introduce the octyl group. We envisioned a synthesis starting from inexpensive *p*‐xylene (**3 c**). The described Wurtz‐type coupling reaction allowed to install the octyl chain, and a selective sodiation of the obtained 4‐octyltoluene (**13 s**), was enabling an aziridine opening to give the protected fingolimod precursor **24**.

On‐demand generated (2‐ethylhexyl)sodium (**1**) was used for the lateral sodiation of **3 c** in the presence of TMEDA. Trapping with 1‐chloroheptane (**12 p**, 8.0 mmol) resulted in 4‐octyltoluene (**13 s**) in 98 % yield on a gram scale and did not require any purification. Benzylic sodiation of **13 s** followed by a transmetalation to magnesium allowed a CuBr SMe_2_‐catalyzed aziridine opening of **25** to give the protected tertiary amine **24** in 60 % yield. Fingolimod hydrochloride (**5**) was obtained by deprotection of **24** using an aq. HCl solution (89 % yield). Azridine **25** is conveniently prepared from the readily available tris(hydroxymethyl)aminomethane known as tris, commonly used as gel electrophoresis buffer.^[16c]^ Fingolimod (**5**) was obtained in an overall yield of 40 % within 6 steps starting from tris.

7‐Fold isotopically labeled salmeterol‐*d*_7_ was easily prepared via sodiation of toluene‐*d*_8_ with the alkylsodium reagent **1**. The resulting benzylic metal species **4 p** gave upon addition of oxetane (**9 h**) the primary alcohol **10 au** in 75 % yield without the need of a chromatographical purification. Alkylation of **10 au** with 1,6‐dibromohexane and amination of the resulting product with **26** gave the acetal protected salmeterol precursor. Cleavage of the acetal group gave salmeterol‐*d*_7_ (**6**).^[35]^ The synthesis showed the applicability of the dedeuteration by facilitating the introduction of multiple times isotopically labeled groups.

SLAB surfactants are valuable detergents. Compared to their branched counterparts they are easily biodegradable and therefore more environmentally friendly. However, the installation of the linear alkyl chain can be challenging as cationic methods often lead to branched structures.[^18^, ^36^] As we showed earlier, the introduction of linear alkyl chain using our Wurtz‐type procedure is uncomplicated and high yielding. The SLAB‐sulfonic acid **8** was readily obtained from toluene by Wurtz‐type coupling of sodium species **4 b** with 1‐cholododecane (**12 a**) followed by sulfonation with chlorosulfonic acid (**23**). Again no purification was required for the synthesis of **13 t** obtained in quantitative yield.

## Conclusion

In summary, we reported a lateral sodiation of alkyl (hetero)arenes using on‐demand generated hexane soluble (2‐ethylhexyl)sodium (**1**) in the presence of TMEDA. (2‐Ethylhexyl)sodium (**1**) was prepared via a sodium packed‐bed reactor and used for metalations at ambient temperature in batch as well as in continuous flow. The resulting benzylic sodium species of type (**4**) were subsequently trapped with various electrophiles including carbonyl compounds, epoxides, oxetane, allyl/benzyl chlorides, alkyl halides and alkyl tosylates. The Wurtz‐type reactions with secondary alkyl halides and tosylates proceeded under complete inversion of stereochemistry. A 20‐fold reaction scale‐up using an in‐line quenching procedure with cyclohexene oxide was reported. Furthermore, the utility of the lateral sodiation was shown in the synthesis of pharmaceutical relevant compounds. Thus, fingolimod (**5**) was prepared from *p‐*xylene (**3 c**) applying the lateral sodiation twice. In addition, multiple times isotopically labeled salmeterol‐*d*_7_ (**6**) and fenpiprane (**7**) as well as precursors to super linear alkylbenzene surfactants such as **8** were easily accessible and did not require purification steps. Further investigations on (2‐ethylhexyl)sodium and its applications are currently under way in our laboratories.

## Conflict of interest

The authors declare no conflict of interest.

1

## Supporting information

As a service to our authors and readers, this journal provides supporting information supplied by the authors. Such materials are peer reviewed and may be re‐organized for online delivery, but are not copy‐edited or typeset. Technical support issues arising from supporting information (other than missing files) should be addressed to the authors.

Supporting Information

## Data Availability

The data that support the findings of this study are available in the Supporting Information of this article.
