# Supplementary material for: Continuous Flow Preparation of Benzylic Sodium Organometallics
Source: Angew Chem Int Ed Engl. 2022 May 11;61(30):e202203807. doi: 10.1002/anie.202203807 (PMC9400861; doi:10.1002/anie.202203807)

Angewandte Chemie
Eine Zeitschrift der Gesellschaft Deutscher Chemiker

Supporting Information

Continuous Flow Preparation of Benzylic Sodium Organometallics

*J. H. Harenberg, R. Reddy Annapureddy, K. Karaghiosoff, P. Knochel**

Supporting Information

General information 2
Analytical data 2
Typical procedures and screenings 3
Preparation of starting materials 19
Preparation of products 25
Synthesis of fingolimod (**5**) 112
Synthesis of salmeterol-d_7 (**6**) 119
Synthesis of the SLAB 4-tridecylbenzenesulfonic acid (**8**) 130
Stereo- amd chemoselectivity studies on the Wurtz-type coupling of benzhydrylsodium (**4n**) 132
Temperature influence on the metalation site of 2-isopropyl-4-methylthiazole (**17a**) 137
Single crystal X-ray diffraction studies 139
Chiral HPLC Analysis 156
NMR-Spectra 158

General information

Caution: Sodium metal requires careful handling, since it is easily flammable. As stated in **TP1** we suggest handling the sodium-packed-bed reactor in an *i*-PrOH cooling bath at 25 °C.

Hexane was continuously refluxed and freshly distilled from sodium benzophenone ketyl under nitrogen and stored over molecular sieves. Solvents for column chromatography were distilled prior to use.

Reagents

All reagents were obtained from commercial sources and used without further purification unless otherwise stated.

Chromatography

Flash column chromatography was performed using SiO_2 60 (0.040-0.063 mm, 230-400 mesh ASTM) from Merck. Thin layer chromatography (TLC) was performed using aluminum plates covered with SiO_2 (Merck 60, F-254). Spots were visualized under UV light.

Analytical data

Yields refer to isolated yields of compounds estimated to be >95% pure as determined by ^{1}H-NMR (25 °C) and capillary GC. NMR spectra were recorded on Bruker ARX 200, AC 300, WH 400 or AMX 600 instruments. Chemical shifts are reported as δ-values in ppm relative to the deuterated solvent peak: $CDCl_3$ (δH: 7.26; δC: 77.16). For the observation of the observed signal multiplicities, the following abbreviations were used: s (singlet), d (doublet), dd (doublet of doublets), t (triplet), q (quartet), quint (quintet), sext (sextet), sept (septet) and m (multiplet). Melting points are uncorrected and were measured on a Büchi B.540 apparatus. Infrared spectra were recorded from 4000-400 cm^{-1} on a Nicolet 510 FT-IR or a Perkin-Elmer 281 IR spectrometer. Absorption bands are reported in wavenumbers (cm^{-1}). Gas chromatography (GC) was performed with instruments of the type Hewlett-Packard 6890 or 5890 Series II, using a column of the type HP 5 (Hewlett-Packard, 5% phenylmethylpolysiloxane; length: 10 m, diameter: 0.25 mm, film thickness: 0.25 µm). The detection was accomplished using a flame ionization detector. Mass spectra (MS) and high resolution mass spectra (HRMS) were recorded on a Finnigan MAT95Q or Finnigan MAT90 instrument for electron impact ionization (EI) and electrospray ionization (ESI). For the combination of gas chromatography with mass spectroscopic detection, a GC-MS of the type Hewlett-Packard 6890 / MSD 5793 networking was used (column: HP 5-MS, Hewlett-Packard; 5% phenylmethylpolysiloxane; length: 15 m, diameter 0.25 mm; film thickness: 0.25 µm).

Typical procedures and screenings

General remarks on flow and subsequent batch quenching reactions

Tetradecane (n-$C_{14}H_{30}$), tridecane (n-$C_{13}H_{28}$), dodecane (n-$C_{12}H_{26}$) or undecane (n-$C_{11}H_{24}$) were used as internal standards. All flasks were heat gun dried (650 °C) under vacuum and backfilled with argon after cooling. Syringes, which were used to transfer reagents and solvents, were purged with argon three times prior to use. Batch quenching reactions were carried out with magnetic stirring. Flow reactions were performed on commercially available flow systems. A Vapourtec E-series Integrated Flow Chemistry System with 3rd Pump Kit, Organometallic Kit, Collection Valve Kit and Cryogenic Reaction Kit was used. Hexane solutions of the 3-(chloromethyl)heptane and THF solutions of the remaining reactants were kept in flasks with rubber septa under an argon atmosphere during the reactions. All reactions were performed in coiled tube reactors. Coiled reactors were made from PFA or PTFE Teflon (I.D. = 0.8 mm or 0.25 mm, O.D. = 1.6 mm) tubing and T-pieces (I.D. = 0.5 mm) were used as mixers. Prior to performing reactions, the system was dried by flushing with dry THF (flow rate: 1.00 mL/min; run-time: 10 to 30 min) or by first flushing six times with MeOH followed by dry *n*-hexane (flow rate: 1.00 mL/min; run-time: 10 to 30 min).

Typical procedure 1 (TP1): Preparation and activation of the sodium-packed-bed reactor

A 50 mL round bottom flask was charged with an oval shaped stirring bar (length: 2.5 cm; width: 1.2 cm) and sodium dispersion (30 wt% in toluene, particle size <0.1 mm, 10 mL). The sodium dispersion was stirred for 4 h at 300 to 400 rpm (Figure SI 1). An ovendried Omnifit® Labware glass column (length: 25 cm; inner diameter: 6.6 mm, figure SI 2) was closed at one side with a nonadjustable PTFE endpiece. The column was charged with the previously stirred sodium dispersion (particle size ca. 1 mm) using a 10 mL syringe without a cannula until the sodium metal reached a height of 10 cm (≙ 3.4 mL of sodium, Figure SI 3). The adjustable PTFE endpiece was used to close the column and was adjusted to give the maximum height of 22 cm (≙ V_{R1} = 7.5 mL). The packed-bed reactor was then installed in the flow setup, attaching the ETFE nut of the inlet to the tubing (V_{pre1} = 0.60 mL) connected to the pump and the nut of the outlet to the precooling loop (V_{pre2} = 0.35 mL) connected to the T-shaped mixer. The packed-bed reactor was placed upright (the inlet of the reactor facing downwards, Figure SI 4) in an *i*-PrOH bath to maintain a temperature of 25 °C. After washing with *n*-hexane (runtime: 10 min; flow rate: 2.0 mL/min), the sodium was activated by pumping a solution of *i*-PrOH (0.1 M in *n*-hexane; runtime: 2 min; flow rate: 5.0 mL/min) through the packed-bed reactor. A solution of 3-(chloromethyl)heptane (**2**, 0.2 M in *n*-hexane; flow rate: 2.0 mL/min) was pumped through the column. After 15 min, an aliquot was taken and analyzed by GC to monitor full conversion of the 3-(chloromethyl)heptane (**2**) to the corresponding organosodium derivative and steady state.

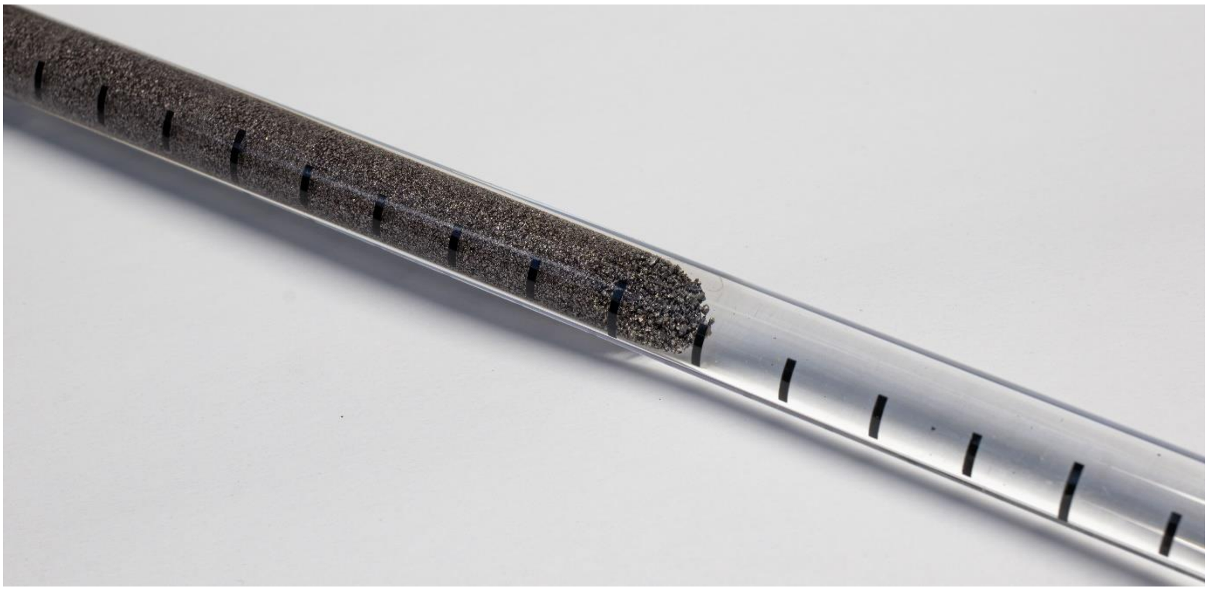

Figure SI 1: Packed-bed reactor filled with sodium particles of appropriate size.

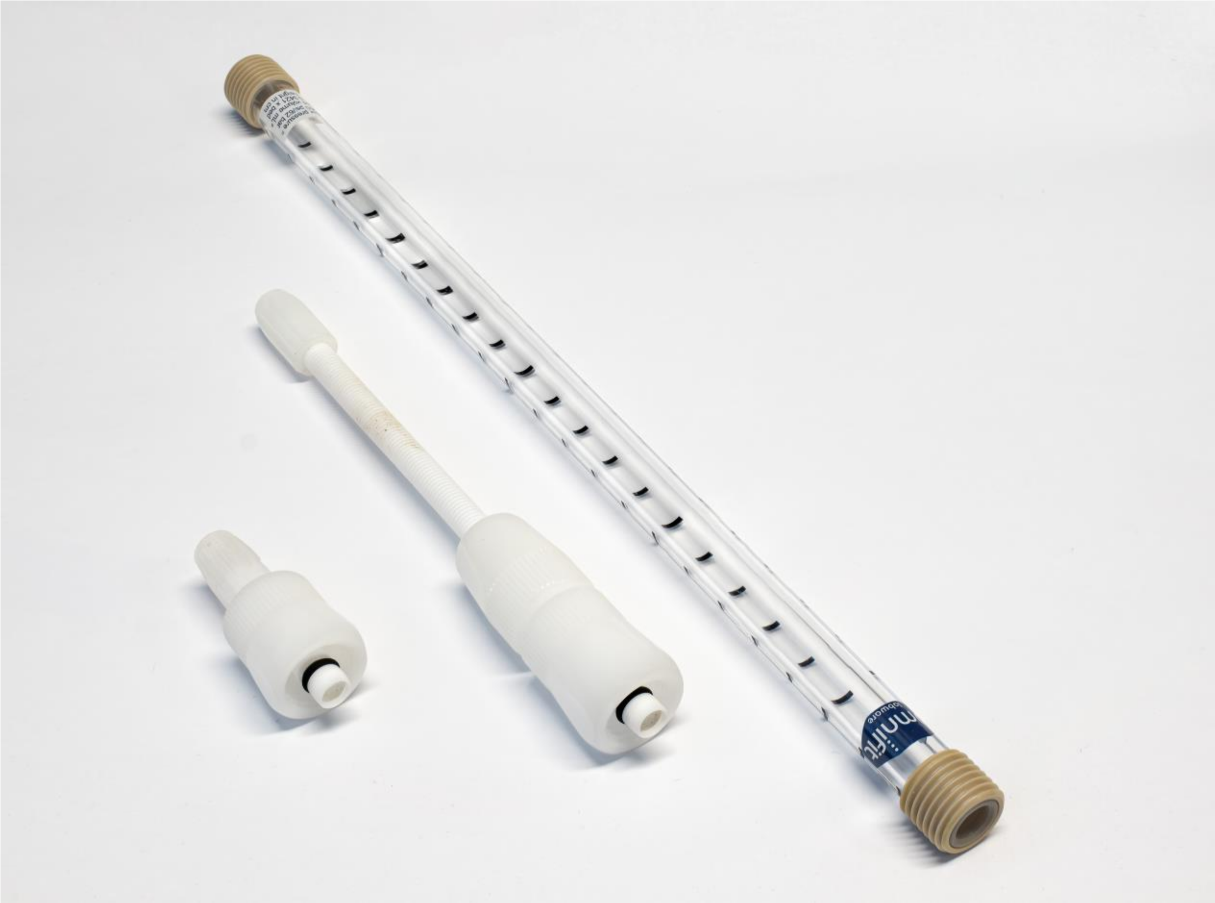

Figure SI 2: From left to right: nonadjustable PTFE endpiece with ETFE nut; adjustable PTFE endpiece with ETFE nut; Omnifit® Labware glass column.

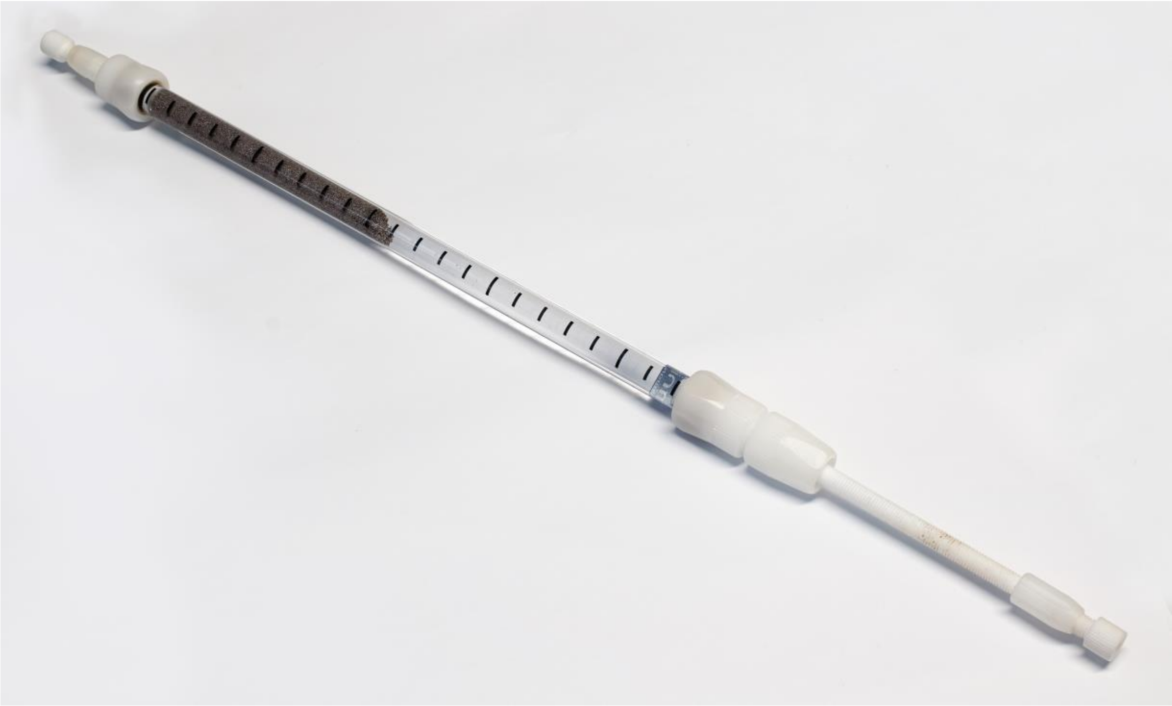

Figure SI 3: Closed packed-bed reactor filled with sodium particles. Adjustable endpiece set to give the maximum volume of V_{R1} = 7.5 mL.

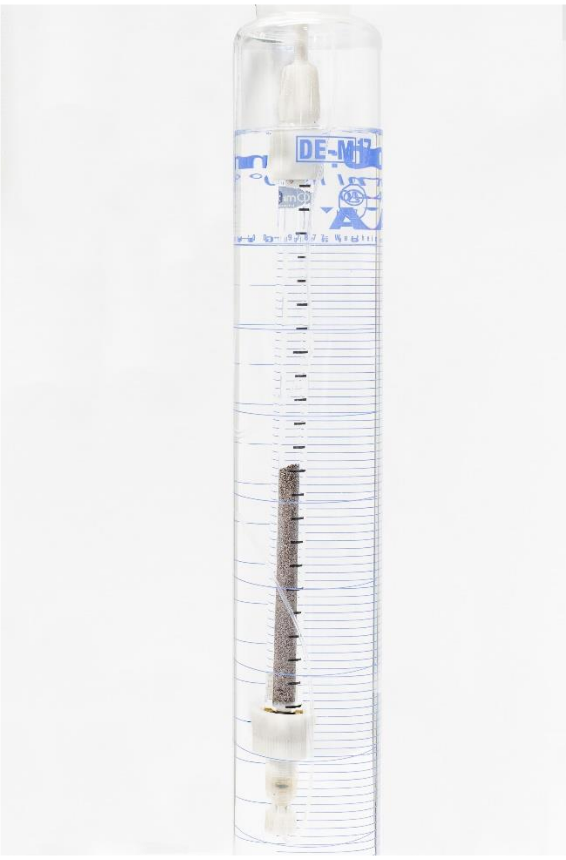

Figure SI 4: Packed-bed sodium reactor in an *i*-PrOH bath connected *via* a nonadjustable endpiece (bottom) to the tubing (V_{pre1}) attached to the pump. Adjustable endpiece (top) connected to the tubing (V_{pre2}) attached to the T-shaped mixer.

Screenings

Ketones were used as electrophiles in most metalation screenings, since sodiation of alkylsubstituted arenes proceeded in the presence of epoxides. Therefore, monitoring of the metalation time is complicated using epoxide electrophiles. Screenings at lower temperatures in continuous flow were not feasible due to significantly increased precipitation and therefore clogging.

Time screening for the sodiation of methyl substituted arenes in continuous flow

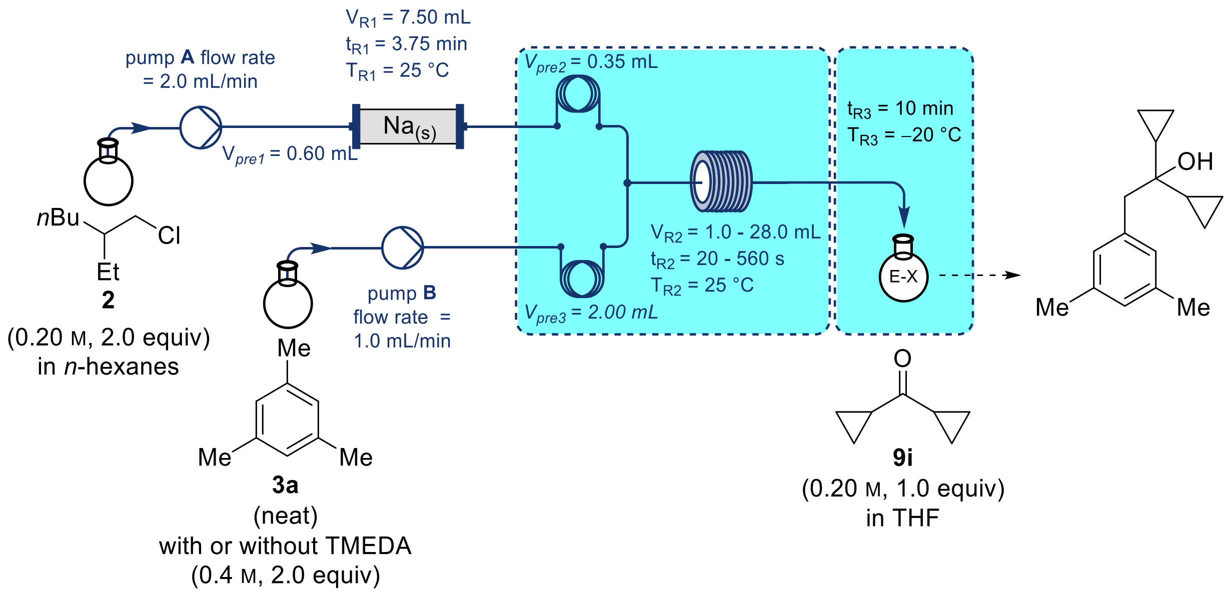

Scheme SI 1: Set-up for the on demand generation of (2-ethylhexyl)sodium (**1**), in-line benzylic sodiation of mesitylene (**3a**) and subsequent batch quench with dicyclopropylketone (**9i**) as electrophile.

Solutions of mesitylene (**3a**) with and without TMEDA (0.4 M, 2.0 equiv) and 3-(chloromethyl)heptane (**2**, 0.2 M, 2.0 equiv) in *n*-hexane were prepared. The solution of **2** was pumped through the activated sodium packed-bed reactor (see **TP1**) by pump A (flow rate: 2.0 mL/min) into the precooling loop (V_{pre2} = 0.35 mL) at 25 °C. The solution of **3a** was pumped by pump B (flow rate: 1.0 mL/min) through a precooling loop (V_{pre3} = 2.00 mL), at 25 °C. The solutions were mixed with an overall flow rate of 3.0 mL/min in a T-shaped mixer. The combined stream passed through a tube reactor (V_{R2} = 1.0 – 28.0 mL, t_{R2} = 20 s – 560 s) and were, subsequently upon reaching the steady state, injected for 1 min into a flask charged with dicyclopropylketone (**9i**, 0.20 mmol, 1.0 equiv). Formation of the product was monitored by GC.

Table SI 1: Time screening for the sodiation of mesitylene (3a) in continuous flow

Entry	Reactorsize [mL]	t [s]	Additive	Normalized GC-Yield [%][a]
1	1	20	TMEDA	86
2	5	100	TMEDA	92
3	13	260	TMEDA	98
4	23	460	TMEDA	100
5	28	560	TMEDA	95
6	5	100	-	12
7	13	260	-	11
8	23	460	-	9
9	28	560	-	5

[a]The largest integrated area under the curve corresponding to dicyclopropylketone was normalized to 100% GC-yield the other integrals were adjusted accordingly.

Using TMEDA as additive a plateau value is reached at a reaction time between 100 and 260 s (entry 2 and 3). The low GC-yields obtained without additive (entry 6 - 9) are explained by high the degree of precipitation in the tube reactors. TMEDA is crucial to solubilize the benzyl sodium species (**4a**). Even though a plateau level was reached earlier, we used the conditions resulting in the absolute highest product formation (entry 4)

Time screening for the sodiation of methyl substituted arenes in continuous flow followed by inline quench

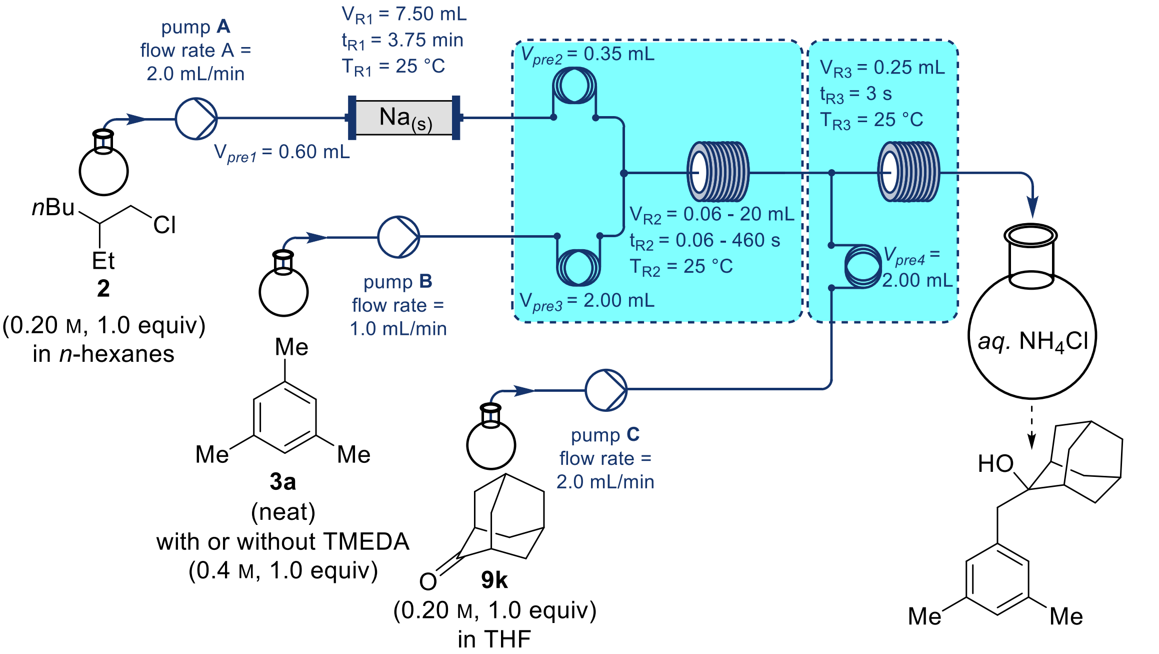

Scheme SI 2: Set-up for the on demand generation of (2-ethylhexyl)sodium (**1**), in-line benzylic sodiation of mesitylene (**3a**) and subsequent in-line quench with adamantanone (**9m**) as electrophile.

Solutions of mesitylene (**3a**) with and without TMEDA (0.4 M, 1.0 equiv), 3-(chloromethyl)heptane (**2**, 0.2 M, 1.0 equiv) in *n*-hexane and adamantone (**9k**, 0.2 M, 1.0 equiv) in THF were prepared. The solution of **2** was pumped through the activated sodium packed-bed reactor (see **TP1**) by pump A (flow rate: 2.0 mL/min) into the precooling loop (V_{pre2} = 0.35 mL) at 25 °C. The solution of **3a** was pumped by pump B (flow rate: 1.0 mL/min) through a precooling loop (V_{pre3} = 2.00 mL), at 25 °C. The solutions were mixed with an overall flow rate of 3.0 mL/min in a T-shaped mixer. The combined stream passed through a tube reactor (V_{R2} = 0.06 – 20.00 mL, t_{R2} = 0.6 s – 400 s). Pump C (flow rate: 2.0 mL/min) pumped the solution of adamantone **9k**, through a precooling loop (V_{pre4} = 2.00 mL), which at 25 °C. The two streams were mixed in another T-shaped mixer, the combined reaction mixture passed through a tube reactor (V_{R3} = 0.25 mL) and was, subsequently upon reaching the steady state, injected into a flask charged with sat. *aq.* NH_4Cl at 25 °C. Formation of the product was monitored by GC.

Table SI 2: Time screening for the sodiation of mesitylene (3a) in continuous flow followed by an in-line quench

Entry	Reactorsize [mL]	t [s]	Additive	Normalized GC-Yield [%][a]
1	0.06	1.2	TMEDA	26
2	0.25	5	TMEDA	70
3	0.5	10	TMEDA	82
4	1	20	TMEDA	90
5	2	40	TMEDA	88
6	5	100	TMEDA	96
7	10	200	TMEDA	95
8	20	400	TMEDA	100
9	0.06	1.2	-	16
10	0.25	5	-	16
11	0.5	10	-	16
12	1	20	-	21
13	2	40	-	18
14	5	100	-	17
15	10	200	-	12
16	20	400	-	7

[a]The largest integrated area under the curve corresponding to product was normalized to 100% GC-yield the other integrals were adjusted accordingly.

Using TMEDA as additive a plateau value is reached at a reaction time of around 100 s (entry 6). The low GC-yields obtained without additive (entry 9 – 16) are explained by high the degree of precipitation in the tube reactors. TMEDA is crucial to solubilize the benzyl sodium species (**4a**).

Temperature screening for the sodiation of alkylsubstituted arenes in batch

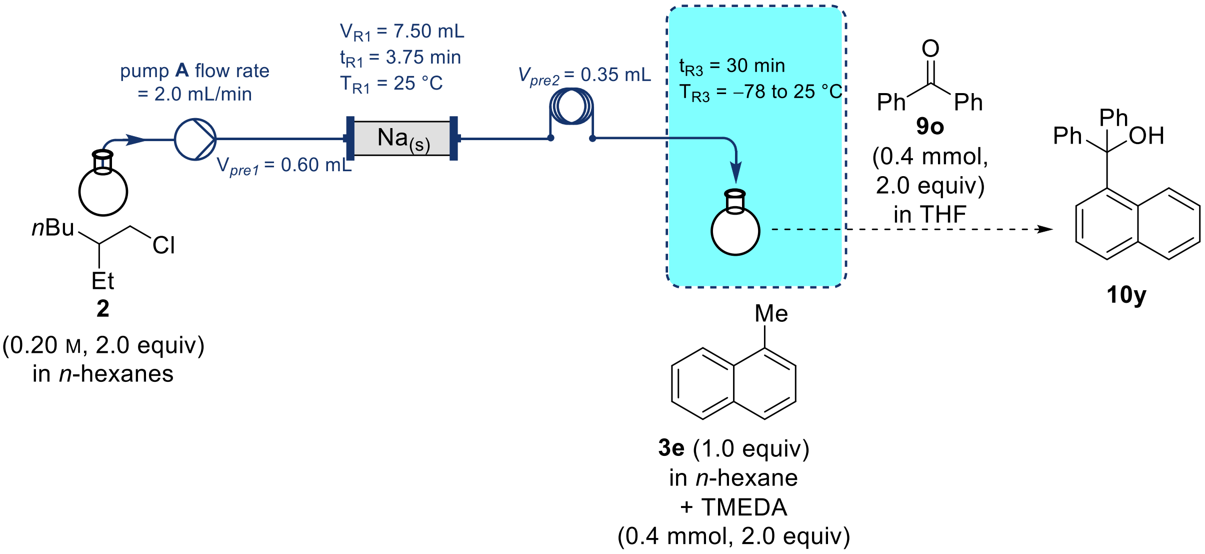

Scheme SI 3: Set-up for the on demand generation of (2-ethylhexyl)sodium (**1**), benzylic sodiation of 1-methylnaphtalene (**3e**) and subsequent batch quench with benzophenone (**9o**) as electrophile.

A solution of 3-(chloromethyl)heptane (**2**, 0.2 M, 2.0 equiv) in *n*-hexane was prepared. The solution of **2** was pumped through the activated sodium packed-bed reactor (see **TP1**) by pump A (flow rate: 2.0 mL/min) into the precooling loop (V_{pre2} = 0.35 mL) at 25 °C. It was injected for 1 min into a flask charged with 1-methylnaphtalene (**3e**, 28 mg, 0.20 mmol, 1.0 equiv) and TMEDA (0.40 mmol, 2.0 equiv) in *n*-hexane (1.0 mL) at the corresponding temperature (–78 to 25 °C). The mixture was stirred at the same temperature for 30 min before benzophenone (**9o**, 73 mg, 0.40 mmol, 2.0 equiv) was added. Conversion of 1-methylnaphtalene (**3e**) was monitored by GC.

Table SI 3: Temperature screening for the sodiation of 1-methylnaphtalene (3e) in batch

Entry	T [°C]	Conversion (**3e**) [%]
1	-78	13
2	-40	50
3	0	85
4	25	100[a]

[a] Product was isolated in 81% yield in case of entry 4.

Comparison between the metalation with *n*BuLi and (2-Ethylhexyl)sodium (1)

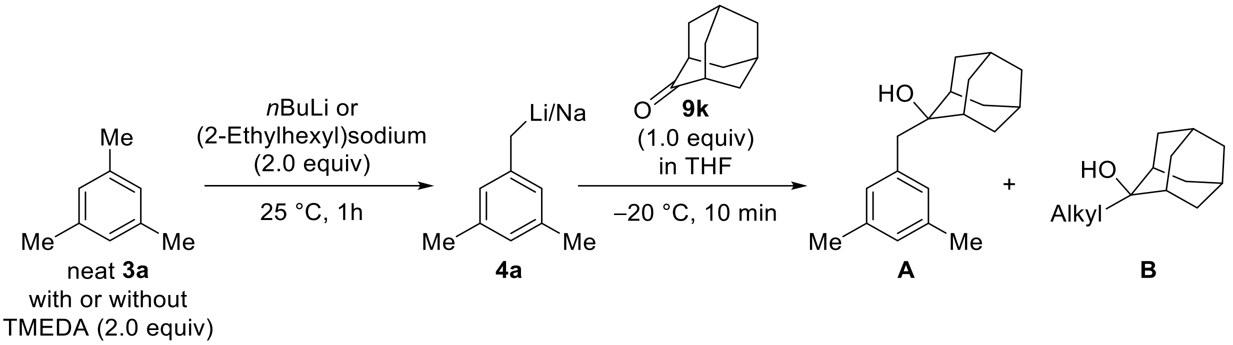

Scheme SI 4: Benzylic metalation of mesitylene (**3a**) using either (2-ethylhexyl)sodium (**1**) or *n*BuLi with or without the presence of TMEDA, and subsequent quench with adamantanone (**9m**) as electrophile.

*n*BuLi (0.80 mmol, 2.0 equiv) or (2-Ethylhexyl)sodium (0.80 mmol, 2.0 equiv) in *n*-hexane was added to a mixture of either neat mesitylene (**3a**, 1.0 mL) or a solution of TMEDA (0.80 mmol, 2.0 equiv) in mesitylene (**3a**, 1.0 mL) at 25 °C. The mixture was stirred at the same temperature for 1 h. The mixture was cooled to –20 °C and adamantone (**9k**, 120 mg, 0.40 mmol, 1.0 equiv) in THF (1.0 mL) was added and stirred at –20 °C for 10 min before sat. *aq.* NH_4Cl was added. Formation of the products **A** and **B** were monitored by GC and GCMS.

Table SI 4: Comparison between the metalation with *n*BuLi and (2-Ethylhexyl)sodium (1).

Entry	Base	Additive	Ratio **A**/**B**
1	*n*BuLi	-	**A** was not detected
2	*n*BuLi	TMEDA	>95/5 (75%)[a]
3	(2-Ethylhexyl)sodium	-	89/11
4	(2-Ethylhexyl)sodium	TMEDA	>95/5

[a]Isolated yield of product **A**.

Time screening for the continous flow preparation of *trans*-2-(3,5-dimethylbenzyl)cyclohexan-1-ol (10a) under Barbiertype conditions

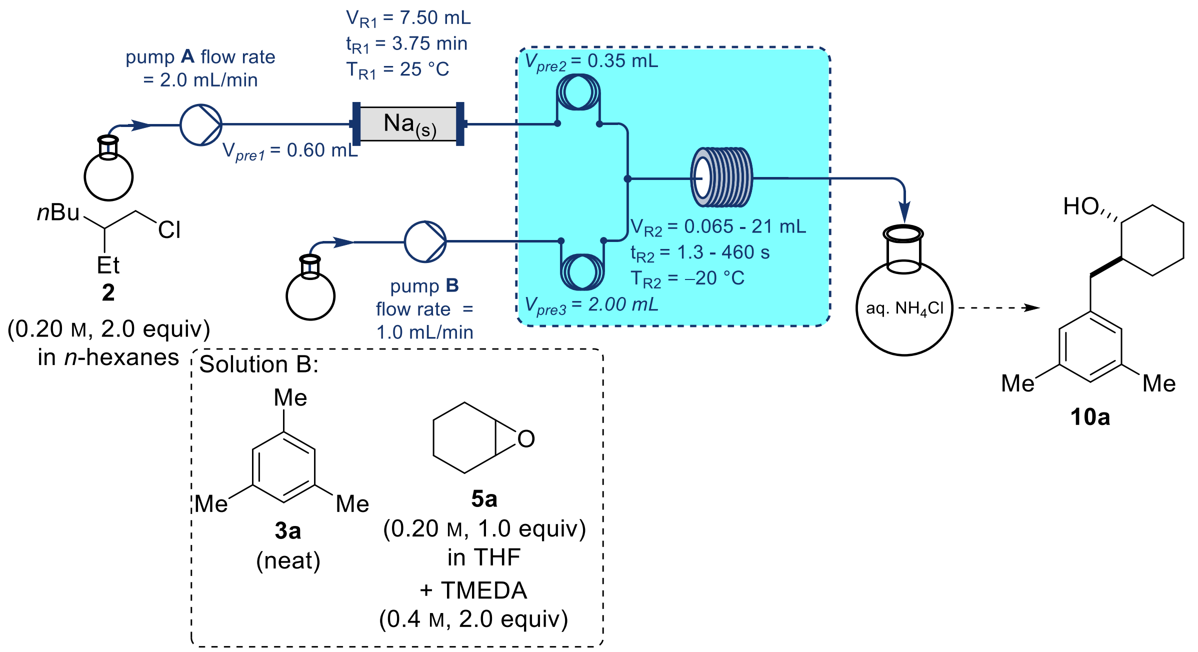

Scheme SI 5: Set-up for the on demand generation of (2-ethylhexyl)sodium (**1**), followed by an in-line benzylic sodiation of mesitylene (**3a**) in the presence of cyclohexeneoxide (**9a**) as electrophile.

A Solution **A** of 3-(chloromethyl)heptane (**2**, 0.2 M) in *n*-hexane and a solution **B** cyclohexeneoxide (**9a** 0.2 M) and TMEDA (0.4 M) in a 1:1 mixture of mesitylene (**3a**) and THF were prepared. The solution of **2** was pumped through the activated sodium packed-bed reactor (see **TP1**) by pump A (flow rate: 2.0 mL/min) into the precooling loop (V_{pre2} = 0.35 mL), which was cooled to –20 °C. Solution **B** was pumped by pump B (flow rate: 1.0 mL/min) through a precooling loop (V_{pre3} = 2.00 mL), which was cooled to –20 °C. The precooled solutions were mixed with an overall flow rate of 3.0 mL/min in a T-shaped mixer. The combined stream passed through a tube reactor and a metal needle (V_{R2} = 0.065 mL to 21 mL, t_{R2} = 1.3 s to 420 s), subsequently upon reaching the steady state, it was injected into a vial charged with a sat. *aq.* NH_4Cl-solution. Formation of product **10a** was monitored by GC.

Table SI 5: Time screening for the continous flow preparation of 2-(3,5-dimethylbenzylcyclohexan-1-ol (10a) under Barbiertype conditions

Entry	Reactorsize [mL]	t [s]	Normalized GC-Yield [%][a]
1	21	420	98
2	19	380	97
3	17	340	107
4	15	300	100
5	10	200	99

6	5	100	98
7	2	40	90
8	1	20	71
9	0.5	10	40
10	0.25	5	22
11	0.065	1.3	13

[a]The largest integrated area under the curve corresponding to the product **10a** was normalized to 100% GC-yield the other integrals were adjusted accordingly.

No significant increase in yield was observable by prolonging the reaction time longer than 100 s (entry 6). The high yield in entry 3 might be considered as an outlier.

Typical procedure 2 (TP2): On-demand synthesis of (2-ethylhexyl)sodium and its use in-line benzylic sodiations.

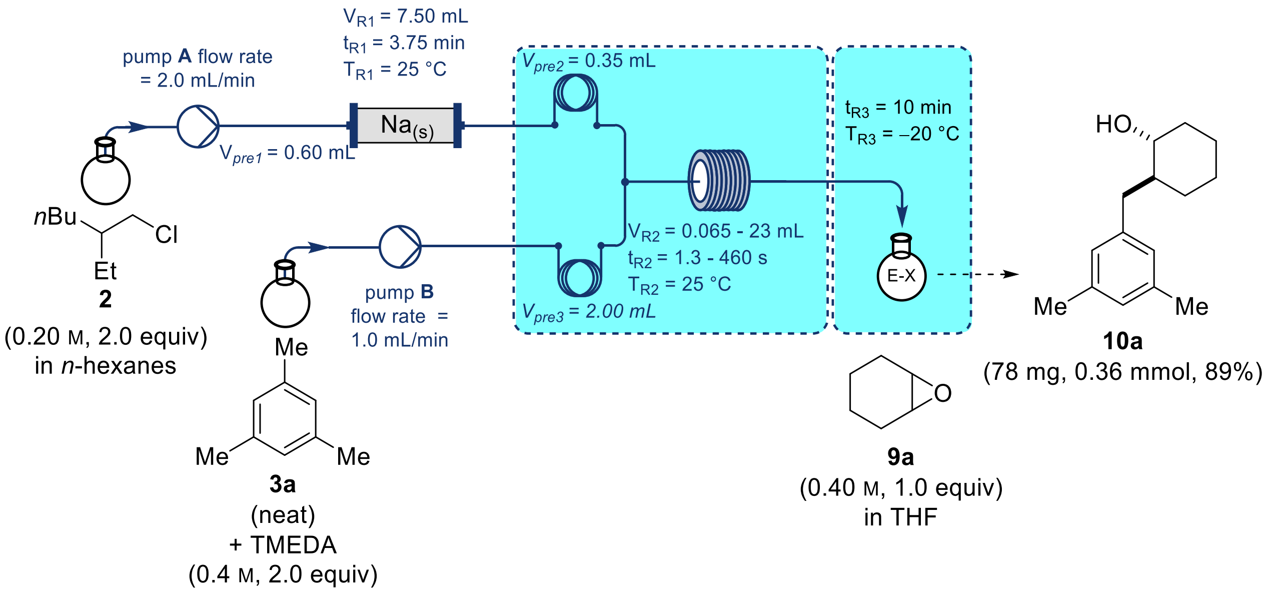

Scheme SI 6: Set-up for the on demand generation of (2-ethylhexyl)sodium (**1**), in-line benzylic sodiations and subsequent batch quench with electrophiles.

Solutions of TMEDA (0.4 M, 2.0 equiv) in mesitylene (**3a**) and 3-(chloromethyl)heptane (**2**, 0.2 M, 2.0 equiv) in *n*-hexane were prepared. The solution of **2** was pumped through the activated sodium packed-bed reactor (see **TP1**) by pump A (flow rate: 2.0 mL/min) into the precooling loop (V_{pre2} = 0.35 mL) at 25 °C. The solution of **3a** was pumped by pump B (flow rate: 1.0 mL/min) through a precooling loop (V_{pre3} = 2.00 mL), at 25 °C. The solutions were mixed with an overall flow rate of 3.0 mL/min in a T-shaped mixer. The combined stream passed through a tube reactor connected to a metal needle (V_{R2} = 0.065 mL to 23.0 mL, t_{R2} = 1.3 s to 460 s). Subsequently upon reaching the steady state, it was injected into a flask charged with cyclohexene oxide (**9a**, 55 mg, 0.40 mmol, 1.0 equiv) in THF (1.0 mL) at –20 °C for 2 min. The reaction mixture was stirred at –20 °C for 10 min followed by another 30 min at 25 °C, before sat. *aq.* NH_4Cl solution was added for quenching the reaction mixture. The aqueous layer was extracted three times with EtOAc (3×30 mL) and the combined organic layers were dried over anhydrous $MgSO_4$ and filtrated. After removal of the solvent, flash column chromatographical purification (silica gel, pentane:EtOAc = 99:1 → 98:2) afforded the title compound **10a** as a white solid (78 mg, 0.36 mmol, 89% yield).

Typical procedure 3 (TP3): On-demand synthesis of (2-ethylhexyl)sodium and its use in batch sodiations.

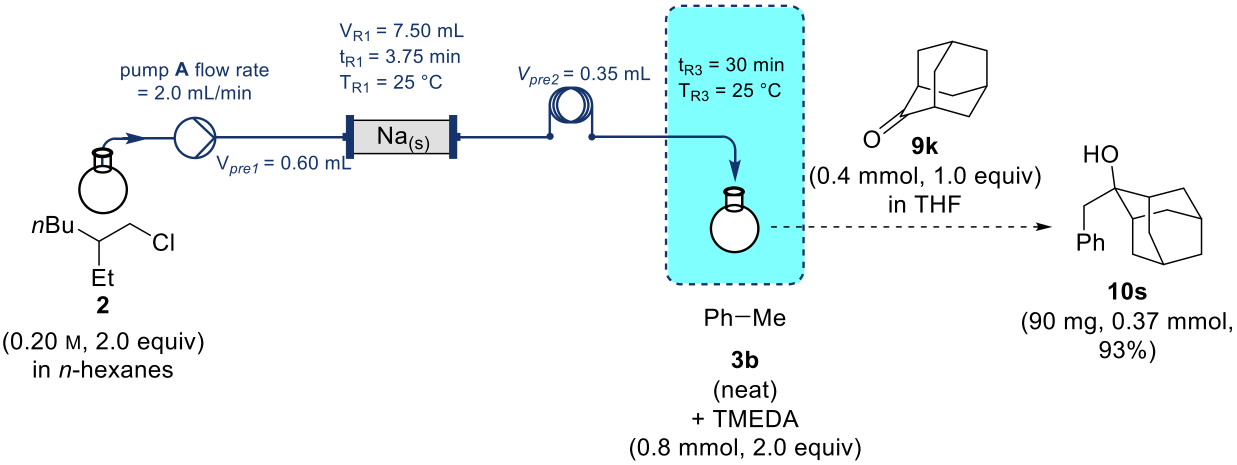

Scheme SI 7: Set-up for the on demand generation of (2-ethylhexyl)sodium (**1**), followed by benzylic batch sodiation of neat **3b** and subsequent electrophile quench with **9k**.

A solution of 3-(chloromethyl)heptane (**2**, 0.2 M, 2.0 equiv) in *n*-hexane was prepared. The solution of **2** was pumped through the activated sodium packed-bed reactor (see **TP1**) by pump A (flow rate: 2.0 mL/min) into the precooling loop (V_{pre2} = 0.35 mL) at 25 °C. Subsequently upon reaching the steady state, it was injected (2 min) into a flask charged with TMEDA (0.80 mmol, 2.0 equiv) in toluene (**3b**, 1.0 mL) and the resulting mixture was stirred at 25 °C for 30 min. The reaction flask was cooled to –20 °C and a solution of adamantanone (**9k**, 60 mg, 0.40 mmol, 1.0 equiv) in THF (1.0 mL) was added The reaction mixture was stirred at –20 °C for 10 min followed by another 30 min at 25 °C, before sat. *aq.* NH_4Cl solution was added for quenching the reaction mixture. The aqueous layer was extracted three times with EtOAc (3×30 mL) and the combined organic layers were dried over anhydrous $MgSO_4$ and filtrated. After removal of the solvent, flash column chromatographical purification (silica gel, pentane:EtOAc = 95:5 → 80:20) afforded the title compound **10s** as a white solid (90 mg, 0.37 mmol, 93% yield).

Typical procedure 4 (TP4): On-demand synthesis of (2-ethylhexyl)sodium and its use in batch sodiations.

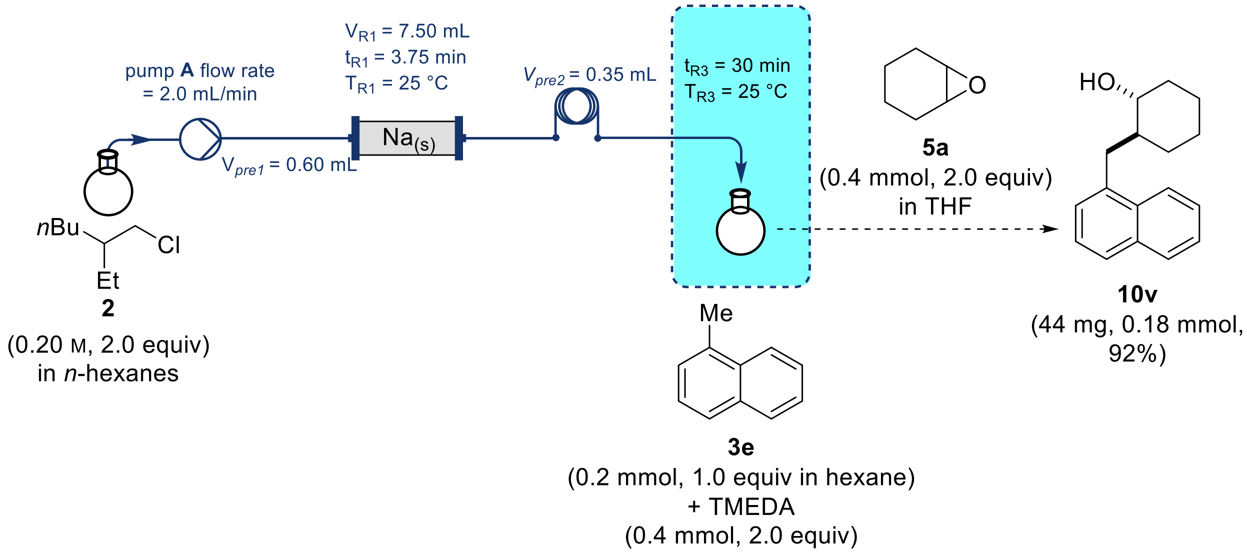

Scheme SI 8: Set-up for the on demand generation of (2-ethylhexyl)sodium (**1**), followed by benzylic batch sodiation of **3e** and subsequent electrophile quench with **5a**.

A solution of 3-(chloromethyl)heptane (**2**, 0.2 M, 2.0 equiv) in *n*-hexane was prepared. The solution of **2** was pumped through the activated sodium packed-bed reactor (see **TP1**) by pump A (flow rate: 2.0 mL/min) into the precooling loop (V_{pre2} = 0.35 mL) at 25 °C. Subsequently upon reaching the steady state, it was injected for 1 min into a flask charged with a solution of TMEDA (0.40 mmol, 2.0 equiv) and 1–methylnaphtalene (**3e**, 28 mg, 0.20 mmol, 1.0 equiv) in hexane (1.0 ml) the mixture was stirred for 30 min at 25 °C. Before it was cooled to –20 °C and a solution of cyclohexene oxide (**9a**, 39 mg, 0.40 mmol, 2.0 equiv) in THF (1.0 mL) was added. The reaction mixture was stirred at –20 °C for 10 min followed by another 30 min at 25 °C, before sat. *aq.* NH_4Cl solution was added for quenching the reaction mixture. The aqueous layer was extracted three times with EtOAc (3×30 mL) and the combined organic layers were dried over anhydrous $MgSO_4$ and filtrated. After removal of the solvent, flash column chromatographical purification (silica gel, pentane:EtOAc = 85:15) afforded the title compound **10v** as a colorless solid (44 mg, 0.18 mmol, 92% yield).

Typical procedure 5 (TP5): On-demand synthesis of (2-ethylhexyl)sodium and its use in batch sodiations using excess of alkyl substituted arenes.

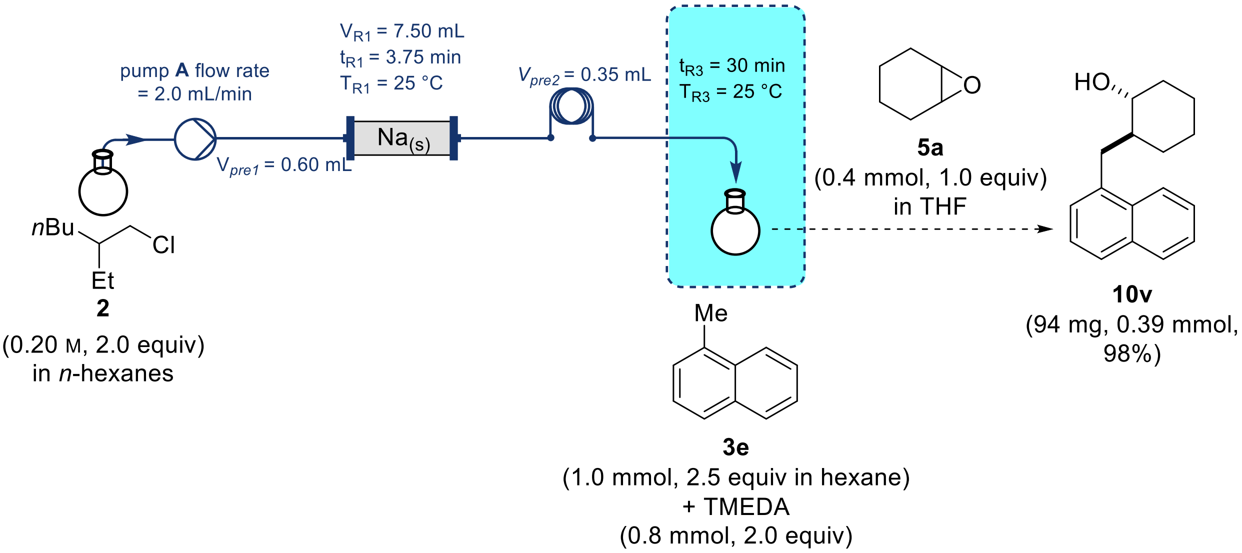

Scheme SI 9: Set-up for the on demand generation of (2-ethylhexyl)sodium (**1**), followed by benzylic batch sodiation of **3e** and subsequent electrophile quench with **5a**.

A solution of 3-(chloromethyl)heptane (**2**, 0.2 M, 2.0 equiv) in *n*-hexane was prepared. The solution of **2** was pumped through the activated sodium packed-bed reactor (see **TP1**) by pump A (flow rate: 2.0 mL/min) into the precooling loop (V_{pre2} = 0.35 mL) at 25 °C. Subsequently upon reaching the steady state, it was injected for 2 min into a flask charged with a solution of TMEDA (0.80 mmol, 2.0 equiv) and 1–methylnaphtalene (**3e**, 142 mg, 1.00 mmol, 2.5 equiv) in hexane (1.0 ml) the mixture was stirred for 30 min at 25 °C. Before it was cooled to –20 °C and a solution of cyclohexene oxide (**9a**, 39 mg, 0.40 mmol, 2.0 equiv) in THF (1.0 mL) was added. The reaction mixture was stirred at –20 °C for 10 min followed by another 30 min at 25 °C, before sat. *aq.* NH_4Cl solution was added for quenching the reaction mixture. The aqueous layer was extracted three times with EtOAc (3×30 mL) and the combined organic layers were dried over anhydrous $MgSO_4$ and filtrated. After removal of the solvent, flash column chromatographical purification (silica gel, pentane:EtOAc = 85:15) afforded the title compound **10v** as a colorless solid (94 mg, 0.39 mmol, 98% yield).

On-demand synthesis of (2-ethylhexyl)sodium and its use in a lateral metalation reaction followed by an in-line quench.

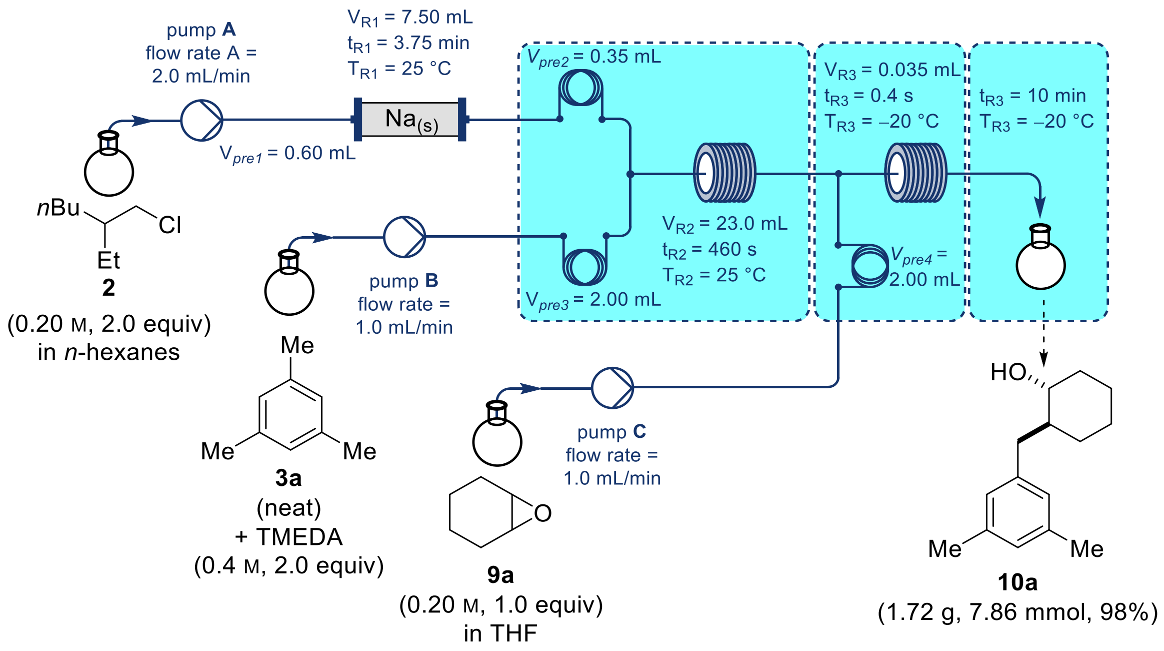

Scheme SI 10: Set-up for the on demand generation of (2-ethylhexyl)sodium (**1**), in-line benzylic sodiation of mesitylene (**3a**) and subsequent in-line quench cyclohexene oxide (**5a**) as electrophile.

Solutions of TMEDA (0.4 M, 2.0 equiv) in mesitylene (**3a**) and 3-(chloromethyl)heptane (**2**, 0.2 M, 2.0 equiv) in *n*-hexane and cyclohexene oxide (**9a**, 0.2 M, 1.0 equiv) in THF were prepared. The solution of **2** was pumped through the activated sodium packed-bed reactor (see **TP1**) by pump A (flow rate: 2.0 mL/min) into the precooling loop (V_{pre2} = 0.35 mL) at 25 °C. The solution of **3a** was pumped by pump B (flow rate: 1.0 mL/min) through a precooling loop (V_{pre3} = 2.00 mL), at 25 °C. The solutions were mixed with an overall flow rate of 3.0 mL/min in a T-shaped mixer. The combined stream passed through a tube reactor (V_{R2} = 0.030 mL, t_{R2} = 0.6 s). Pump C (flow rate: 1.0 mL/min) pumped the solution of cyclohexene oxide **9a**, through a precooling loop (V_{pre4} = 2.00 mL), which was cooled to –20 °C. The two streams were mixed in another T-shaped mixer, the combined reaction mixture passed a metal needle (V_{R3} = 0.035 mL) and was, subsequently upon reaching the steady state, injected into an argon filled flask cooled to –20 °C for 40 min. The reaction mixture was stirred at –20 °C for 10 min and allowed to warm to 25 °C and stirred at this temperature for 30 min before sat. *aq.* NH_4Cl solution was added for quenching the reaction mixture. The aqueous layer was extracted three times with EtOAc (3×70 mL) and the combined organic layers were dried over anhydrous $MgSO_4$ and filtrated. After removal of the solvent, flash column chromatographical purification (silica gel, pentane:EtOAc = 95:5 → 90:10) afforded the title compound **10a** as colorless crystals (1.72 g, 7.86 mmol, 98% yield).

Preparation of starting materials

3-(chloromethyl)heptane (2)

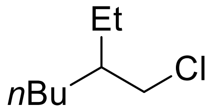

Thionyl chloride (61.7 mL, 0.85 mol, 1.7 equiv) was added over 1 h to a solution of 2-ethylhexan-1-ol (78.5 mL, 0.50 mol, 1.0 equiv) and pyridine (23.0 mL) at 0 °C. The reaction mixture was stirred over night at 55 °C. Afterwards the mixture was quenched with H_2O (100 mL) at 0 °C. The aqueous layer was extracted three times with EtOAc (3×100 mL) and the combined organic layers were dried over anhydrous $MgSO_4$ and filtrated. After removal of the solvent, flash column chromatographical purification (silica gel, isohexane:EtOAc = 99.5:0.5) afforded the title compound **2** as colorless oil (66 g, 0.45 mol, 89% yield).

^{1}H-NMR (400 MHz, $CDCl_3$): δ / ppm = 3.58 – 3.49 (m, 2H), 1.59 (m, 1H), 1.51 – 1.12 (m, 8H), 0.90 (m, 6H)

^{13}C-NMR (100 MHz, $CDCl_3$): δ / ppm = 48.4, 41.6, 31.0, 29.0, 24.3, 23.0, 14.1, 10.9.

IR (Diamond-ATR, neat): $\tilde{\nu}$ / cm^{-1} = 2959, 2928, 2873, 2859, 1458, 1446, 1380, 1294, 782, 768, 724, 683.

MS (EI, 70 eV): *m/z* (%) = 83 (41), 70 (15), 57 (100), 55 (29), 41 (71).

HRMS (EI-orbitrap): *m/z:* [M – C_2H_5] calc. for [$C_6H_{12}Cl$]: 119.0628; found 119.0622.

***trans*-4-(tert-butyl)cyclohexyl 4-methylbenzenesulfonate (12n)**

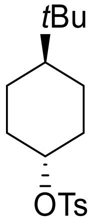

According to the literature[1] 4-toluenesulfonylchloride (11.44 g, 60 mmol, 2.0 equiv) was added portionwise to a solution of *trans*-4-(tert-butyl)cyclohexan-1-ol (4.69 g, 30 mmol, 1.0 equiv), triethylamine (9.2 mL, 60 mmol, 2 equiv), DMAP (0.73 g, 6 mmol, 0.20 mmol) and DCM (300 mL) at 0 °C. The reaction mixture was stirred overnight at 25 °C. Afterwards the mixture was quenched with H_2O (200 mL). The aqueous layer was extracted with DCM (3×150 mL) and the combined organic layers were dried over anhydrous $MgSO_4$ and filtrated. After removal of the solvent, flash column chromatographical purification (silica gel, *n*-pentane:EtOAc = 98:2) afforded the title compound **12n** as colorless crystals (2.93 g, 9.43 mmol, 31% yield).

^{1}H-NMR (400 MHz, $CDCl_3$): δ / ppm = 7.82 – 7.76 (m, 2H), 7.36 – 7.29 (m, 2H), 4.38 – 4.30 (m, 1H), 2.44 (s, 3H), 2.01 – 1.92 (m, 2H), 1.80 – 1.72 (m, 2H), 1.49 – 1.35 (m, 2H), 1.08 – 0.87 (m, 3H), 0.80 (s, 9H).

^{13}C-NMR (100 MHz, $CDCl_3$): δ / ppm = 144.5, 134.9, 129.9 (2C), 127.7 (2C), 82.8, 46.7, 33.0 (2C), 32.3, 27.6 (3C), 25.7 (2C), 21.8.

IR (Diamond-ATR, neat): $\tilde{\nu}$ / cm^{-1} = 2948, 2865, 1381, 1366, 1361, 1345, 1327, 1306, 1186, 1179, 1170, 1097, 1043, 1004, 948, 930, 923, 901, 878, 858, 844, 819, 805, 800, 757, 666.

m.p. (°C): 89 – 90.

[1] Y. Wang, X. Hu, C. A. Morales-Rivera, G.-X. Li, X. Huang, G. He, P. Liu, G. Chen, *J. Am. Chem. Soc.* **2018**, *140*, 9678-9684.

(1*R*,2*S*)-1-chloro-2-methoxycyclohexane (12d)

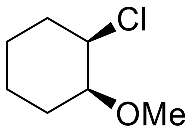

According to the literature[2] *trans*-2-methoxycyclohexan-1-ol (6.50 g, 50 mmol, 1.0 equiv) was added dropwise to a solution of NCS (13,4 g, 100 mmol, 2.0 equiv), PPh_3 (19.7 g, 75 mmol, 1.5 equiv and THF (150 mL) at 0 °C. The reaction mixture was stirred over night at 25 °C. Afterwards the mixture was quenched with H_2O (250 mL). The aqueous layer was extracted three times with EtOAc (3×150 mL) and the combined organic layers were dried over anhydrous $MgSO_4$ and filtrated. After removal of the solvent, pentane was added to precipitate triphenylphosphine oxide the precipitate was removed by filtration. Flash column chromatographical purification (silica gel, *n*-pentane:EtOAc = 98:2) afforded the title compound **12d** as a yellow oil (1.50 g, 10.1 mmol, 20% yield).

^{1}H-NMR (400 MHz, $CDCl_3$): δ / ppm = 4.40 – 4.32 (m, 1H), 3.40 (s, 3H), 3.35 (dt, *J* = 9.0, 3.2 Hz, 1H), 2.12 – 2.01 (m, 1H), 1.88 – 1.58 (m, 5H), 1.46 – 1.35 (m, 1H), 1.35 – 1.21 (m, 1H).

^{13}C-NMR (100 MHz, $CDCl_3$): δ / ppm = 79.8, 61.3, 56.3, 32.3, 26.8, 22.6, 21.4.

Spectral data is in accordance with the literature.[3]

---

[2] E. A. Jaseer, A. B. Naidu, S. S. Kumar, R, K. Rao, K. G. Thakur, G. Sekar *Chem. Commun.* **2007**, 867 – 869.

[3] C. N. Barry, S. J. Baumrucker, R. C. Andrews, S. A. Evans Jr. *J. org. Chem.* **1982**, *47*, 3890 – 3983.

trans-4-((tert-butyldimethylsilyl)oxy)cyclohexan-1-ol

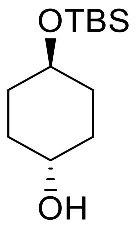

According to the literature,[4] TBSCl (3.17 g, 21 mmol, 1.05 equiv) was dissolved in DMF (10 mL) and the resulting solution was added dropwise to a mixture of *trans*-cyclohexane-1,4-diol (2.32 g, 20 mmol, 1.0 equiv) and imidazole (3.40 g, 50 mmol, 2.5 equiv) in DMF (7.5 mL) and THF (10 mL) at 0 °C. After stirring for 1 h at the same temperature brine (100 mL) was added and the aqueous layer was extracted with EtOAc (3x100 mL) the organic layer was dried over $MgSO_4$. Flash column chromatographical purification (silica gel, *n*-pentane:EtOAc = 4:1) afforded the title compound as a colorless oil (2.50 g, 11 mmol, 54% yield).

^{1}H-NMR (400 MHz, $CDCl_3$): δ / ppm = 3.79 – 3.51 (m, 2H), 1.98 – 1.90 (m, 2H), 1.89 – 1.80 (m, 2H), 1.44 – 1.22 (m, 5H), 0.88 (s, 9H), 0.05 (s, 6H).

^{13}C-NMR (100 MHz, $CDCl_3$): δ / ppm = 70.2, 69.7, 32.9 (2C), 32.8 (2C), 26.0 (3C), 18.3, -4.6. (2C).

MS (EI, 70 eV): *m/z* (%) = 173 (79), 171 (16), 98 (13), 97 (100), 97 (100), 97 (17) 97 (17), 81 (47), 75 (47), 75 (47), 75 (34), 75 (34), 73 (25).

HRMS (EI-orbitrap): *m/z:* [M] calc. for [$C_{12}H_{26}O_2Si$]: 230.1702; found 230.1698.

[4] Z. L. Song, B. M. Wang, Y. Q. Tu, C. A. Fan, S. Y. Zhang, *Org. Lett*. **2003**, *5*, 2319 – 2321.

trans-4-((tert-butyldimethylsilyl)oxy)cyclohexyl 4-methylbenzenesulfonate (12m)

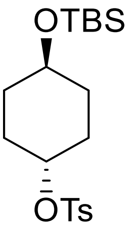

TsCl (3.81 g, 20 mmol, 2.0 equiv) was added portionwise to a mixture of *trans*-4-((tert-butyldimethylsilyl)oxy)cyclohexan-1-ol (2.30 g, 10 mmol, 1.0 equiv), Et_3N (5.6 mL, 40 mmol, 4.0 equiv) in DCM (30 mL) at 0 °C the mixture was allowed to slowly warm to 25 °C and stirred over night at the same temperature. Brine (100 mL) was added and the aqueous layer was extracted with EtOAc (3x250 mL) the organic layer was dried over $MgSO_4$. Flash column chromatographical purification (silica gel, *n*-pentane:EtOAc = 99:1) afforded the title compound **12m** as a colorless solid (3.08 g, 8.0 mmol, 80% yield).

^{1}H-NMR (400 MHz, $CDCl_3$): δ / ppm = 7.83 – 7.74 (m, 2H), 7.36 – 7.29 (m, 2H), 4.62 – 4.49 (m, 1H), 3.83 – 3.66 (m, 1H), 2.44 (s, 3H), 1.96 – 1.84 (m, 2H), 1.83 – 1.72 (m, 2H), 1.59 – 1.47 (m, 2H), 1.41 – 1.30 (m, 2H), 0.84 (s, 9H), 0.01 (s, 6H).

^{13}C-NMR (100 MHz, $CDCl_3$): δ / ppm = 144.6, 134.7, 129.9 (2C), 127.8 (2C), 80.7, 67.7, 30.8 (2C), 28.0 (2C), 25.9 (3C), 21.8, 18.2, -4.7 (2C).

3-chloropropyl 4-methylbenzenesulfonate (12o)

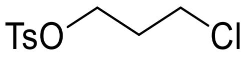

Et_3N (11.2 mL, 80 mmol, 4.0 equiv) was added dropwise to a mixture of 3-chloropropan-1-ol (1.89 g, 20 mmol, 1.0 equiv) and TsCl (7.63 g, 40 mmol, 2.0 equiv) in DCM (60 mL) at 0 °C. The mixture was allowed to warm to 25 °C and stirred over night at the same temperature. Brine (100 mL) was added and the aqueous layer was extracted with EtOAc (3x250 mL) the organic layer was dried over $MgSO_4$. Flash column chromatographical purification (silica gel, *n*-pentane:EtOAc = 99:1) afforded the title compound **12o** as a colorless oil (4.13 g, 16.6 mmol, 83% yield).

^{1}H-NMR (400 MHz, $CDCl_3$): δ / ppm = 7.82 – 7.73 (m, 2H), 7.39 – 7.30 (m, 2H), 4.17 (t, *J* = 5.9 Hz, 2H), 3.55 (t, *J* = 6.2 Hz, 2H), 2.44 (s, 3H), 2.13 – 2.04 (m, 2H).

^{13}C-NMR (100 MHz, $CDCl_3$): δ / ppm = 145.1, 132.7, 130.0 (2C), 127.9 (2C), 66.9, 40.4, 31.7, 21.7.

MS (EI, 70 eV): *m/z* (%) = 173 (51), 172 (100), 155 (60), 119 (24), 108 (26), 107 (12), 91 (86), 65 (10).

HRMS (EI-orbitrap): *m/z:* [M] calc. for [$C_{10}H_{13}ClO_3S$]: 248.0274; found 248.0269.

Preparation of products

***trans*-2-(3,5-dimethylbenzyl)cyclohexan-1-ol (10a)**

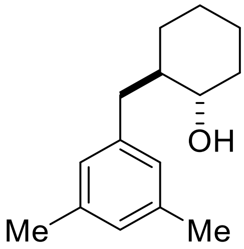

According to **TP2**, solutions of TMEDA (0.4 M, 2.0 equiv) in mesitylene (**3a**) and 3-(chloromethyl)heptane (**2**, 0.2 M, 2.0 equiv) in *n*-hexane were prepared. The solution of **2** was pumped through the activated sodium packed-bed reactor (see **TP1**) by pump A (flow rate: 2.0 mL/min) into the precooling loop (V_{pre2} = 0.35 mL) at 25 °C. The solution of **3a** was pumped by pump B (flow rate: 1.0 mL/min) through a precooling loop (V_{pre3} = 2.00 mL), at 25 °C. The solutions were mixed with an overall flow rate of 3.0 mL/min in a T-shaped mixer. The combined stream passed through a tube reactor connected to a metal needle (V_{R2} = 23.0 mL, t_{R2} = 460 s). Subsequently upon reaching the steady state, it was injected into a flask charged with cyclohexene oxide (**9a**, 39 mg, 0.40 mmol, 1.0 equiv) in THF (1.0 mL) at –20 °C for 2 min. The reaction mixture was stirred at –20 °C for 10 min followed by another 30 min at 25 °C, before sat. *aq.* NH_4Cl solution was added to quench the reaction mixture. The aqueous layer was extracted three times with EtOAc (3×30 mL) and the combined organic layers were dried over anhydrous $MgSO_4$ and filtrated. After removal of the solvent, flash column chromatographical purification (silica gel, pentane:EtOAc = 95:5 → 90:10) afforded the title compound **10a** as a colorless solid (78 mg, 0.36 mmol, 89% yield).

In-line scale-up reaction

Solutions of TMEDA (0.40 M, 2.0 equiv) in mesitylene (**3a**) and 3-(chloromethyl)heptane (**2**, 0.20 M, 2.0 equiv) in *n*-hexane and cyclohexene oxide (**9a**, 0.07 M, 1.0 equiv) in THF were prepared. The solution of **2** was pumped through the activated sodium packed-bed reactor (see **TP1**) by pump A (flow rate: 2.0 mL/min) into the precooling loop (V_{pre2} = 0.35 mL) at 25 °C. The solution of **3a** was pumped by pump B (flow rate: 1.0 mL/min) through a precooling loop (V_{pre3} = 2.00 mL), at 25 °C. The solutions were mixed with an overall flow rate of 3.0 mL/min in a T-shaped mixer. The combined stream passed through a tube reactor (V_{R2} = 0.030 mL, t_{R2} = 0.6 s). Pump C (flow rate: 3.0 mL/min) pumped the solution of **9a**, through a precooling loop (V_{pre4} = 2.00 mL), which was cooled to –20 °C. The two streams were mixed in another T-shaped mixer, the combined reaction mixture passed a metal needle (V_{R3} = 0.035 mL) and was, subsequently upon reaching the steady state, injected into an argon filled flask cooled to –20 °C for 40 min. The reaction mixture was stirred at –20 °C for 10 min and allowed to warm to 25 °C and stirred at this temperature for 30 min before sat. *aq.* NH_4Cl solution

was added for quenching the reaction mixture. The aqueous layer was extracted three times with EtOAc (3×70 mL) and the combined organic layers were dried over anhydrous $MgSO_4$ and filtrated. After removal of the solvent, flash column chromatographical purification (silica gel, isohexane:EtOAc = 95:5 → 90:10) afforded the title compound **10a** as colorless crystals (1.72 g, 7.86 mmol, 98% yield).

Barbier-type reaction

Solutions of TMEDA (0.4 M, 2.0 equiv) and cyclohexene oxide (**9a**, 39 mg, 0.40 mmol, 1.0 equiv) in a mesitylene (**3a,** 1.0 mL) THF (1.0 mL) mixture and a second solution of 3-(chloromethyl)heptane (**2**, 0.2 M, 2.0 equiv) in *n*-hexane were prepared. The solution of **2** was pumped through the activated sodium packed-bed reactor (see **TP1**) by pump A (flow rate: 2.0 mL/min) into the precooling loop (V_{pre2} = 0.35 mL) at 25 °C. Subsequently upon reaching the steady state, it was injected for 2 min into a flask charged with the first solution of cyclohexene oxide (**9a**) and mesitylene (**3a**) at –20 °C. The reaction mixture was stirred at –20 °C for 10 min followed by another 30 min at 25 °C, before sat. *aq.* NH_4Cl solution was added to quench the reaction mixture. The aqueous layer was extracted three times with EtOAc (3×30 mL) and the combined organic layers were dried over anhydrous $MgSO_4$ and filtrated. After removal of the solvent, flash column chromatographical purification (silica gel, pentane:EtOAc = 95:5 → 90:10) afforded the title compound **10a** as a colorless solid (72 mg, 0.33 mmol, 82% yield).

^{1}H-NMR (400 MHz, $CDCl_3$): δ / ppm = 6.83 (s, 1H), 6.81 (s, 2H), 3.37 – 3.24 (m, 1H), 3.07 (dd, *J* = 13.2, 4.2 Hz, 1H), 2.29 (s, 7H), 2.02 – 1.91 (m, 1H), 1.79 – 1.62 (m, 2H), 1.62 – 1.54 (m, 1H), 1.54 – 1.40 (m, 2H), 1.33 – 1.19 (m, 2H), 1.16 – 1.02 (m, 1H), 0.91 (qd, *J* = 12.8, 3.5 Hz, 1H).

^{13}C-NMR (100 MHz, $CDCl_3$): δ / ppm = 140.8, 137.7 (2C), 127.5, 127.3 (2C), 74.8, 47.2, 39.1, 35.9, 30.3, 25.6, 25.0, 21.4 (2C).

IR (Diamond-ATR, neat): $\tilde{\nu}$ / cm^{-1} = 3338, 2920, 2854, 1605, 1461, 1447, 1070, 1058, 1034, 845, 706.

MS (EI, 70 eV): *m/z* (%) = 131 (13), 129 (10), 128 (11), 121 (10), 120 (100), 119 (42), 117 (19), 115 (24), 105 (76), 91 (42), 79 (18), 77 (14).

HRMS (EI-orbitrap): *m/z:* [M] calc. for [$C_{15}H_{22}O$]: 218.1671; found 218.1663.

m.p. (°C): 88.3 – 90.4.

trans-2-benzylcyclohexan-1-ol (10b)

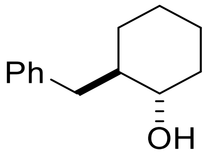

According to **TP2,** solutions of TMEDA (0.4 M, 2.0 equiv) in toluene (**3b**) and 3-(chloromethyl)heptane (**2**, 0.2 M, 2.0 equiv) in *n*-hexane were prepared. The solution of **2** was pumped through the activated sodium packed-bed reactor (see **TP1**) by pump A (flow rate: 2.0 mL/min) into the precooling loop (V_{pre2} = 0.35 mL) at 25 °C. The solution of **3b** was pumped by pump B (flow rate: 1.0 mL/min) through a precooling loop (V_{pre3} = 2.00 mL), at 25 °C. The solutions were mixed with an overall flow rate of 3.0 mL/min in a T-shaped mixer. The combined stream passed through a tube reactor connected to a metal needle (V_{R2} = 23.0 mL, t_{R2} = 460 s). Subsequently upon reaching the steady state, it was injected into a flask charged with cyclohexene oxide (**9a**, 39 mg, 0.40 mmol, 1.0 equiv) in THF (1.0 mL) at –20 °C for 2 min. The reaction mixture was stirred at –20 °C for 10 min followed by another 30 min at 25 °C, before sat. *aq.* NH_4Cl solution was added to quench the reaction mixture. The aqueous layer was extracted three times with EtOAc (3×30 mL) and the combined organic layers were dried over anhydrous $MgSO_4$ and filtrated. After removal of the solvent, flash column chromatographical purification (silica gel, pentane:EtOAc = 95:5 → 80:20) afforded the title compound **10b** as a colorless solid (62 mg, 0.33 mmol, 81% yield).

^{1}H-NMR (400 MHz, $CDCl_3$): δ / ppm = 7.33 – 7.26 (m, 2H), 7.22 – 7.18 (m, 3H), 3.35 – 3.27 (m, 1H), 3.19 (dd, *J* = 13.3, 3.9 Hz, 1H), 2.36 (dd, *J* = 13.3, 9.2 Hz, 1H), 2.02 – 1.97 (m, 1H), 1.80 – 1.46 (m, 5H), 1.36 – 1.18 (m, 2H), 1.17 – 1.01 (m, 1H), 0.97 0.87 (m, 1H).

^{13}C-NMR (100 MHz, $CDCl_3$): δ / ppm = 140.9, 129.5 (2C), 128.3 (2C), 125.8, 74.6, 47.1, 39.1, 35.9, 30.1, 25.5, 25.0.

IR (Diamond-ATR, neat): $\tilde{\nu}$ / cm^{-1} = 3338, 3025, 2922, 2853, 1603, 1494, 1447, 1077, 1061, 1032, 1027, 853, 743, 699.

MS (EI, 70 eV): *m/z* (%) = 172 (60), 143 (10), 130 (14), 129 (36), 128 (19), 117 (23), 115 (33), 104 (56), 92 (55), 91 (100), 81 (37), 80 (18), 79 (26), 77 (11), 65 (11).

HRMS (EI-orbitrap): *m/z:* [M – H_2O] calc. for [$C_{13}H_{16}$]: 172.1252; found 172.1246.

m.p. (°C): 78.0 – 80.2.

***trans*-2-(4-methylbenzyl)cyclohexan-1-ol (10c)**

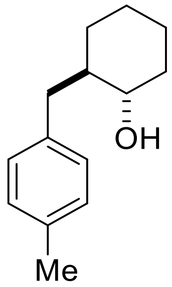

According to **TP2,** solutions of TMEDA (0.4 M, 2.0 equiv) in *p*-xylene (**3c**) and 3-(chloromethyl)heptane (**2**, 0.2 M, 2.0 equiv) in *n*-hexane were prepared. The solution of **2** was pumped through the activated sodium packed-bed reactor (see **TP1**) by pump A (flow rate: 2.0 mL/min) into the precooling loop (V_{pre2} = 0.35 mL) at 25 °C. The solution of **3c** was pumped by pump B (flow rate: 1.0 mL/min) through a precooling loop (V_{pre3} = 2.00 mL), at 25 °C. The solutions were mixed with an overall flow rate of 3.0 mL/min in a T-shaped mixer. The combined stream passed through a tube reactor connected to a metal needle (V_{R2} = 23.0 mL, t_{R2} = 460 s). Subsequently upon reaching the steady state, it was injected into a flask charged with cyclohexene oxide (**9a**, 39 mg, 0.40 mmol, 1.0 equiv) in THF (1.0 mL) at –20 °C for 2 min. The reaction mixture was stirred at –20 °C for 10 min followed by another 30 min at 25 °C, before sat. *aq.* NH_4Cl solution was added to quench the reaction mixture. The aqueous layer was extracted three times with EtOAc (3×30 mL) and the combined organic layers were dried over anhydrous $MgSO_4$ and filtrated. After removal of the solvent, flash column chromatographical purification (silica gel, pentane:EtOAc = 90:10 → 80:20) afforded the title compound **10c** as a white solid (65 mg, 0.32 mmol, 80% yield).

^{1}H-NMR (400 MHz, $CDCl_3$): δ / ppm = 7.10 (s, 4H), 3.36 – 3.25 (m, 1H), 3.14 (dd, *J* = 13.4, 4.0 Hz, 1H), 2.34 (s, 4H), 2.06 – 1.92 (m, 1H), 1.82 – 1.55 (m, 4H), 1.55 – 1.44 (m, 1H), 1.37 – 1.18 (m, 2H), 1.18 – 1.04 (m, 1H), 0.98 – 0.82 (m, 1H).

^{13}C-NMR (100 MHz, $CDCl_3$): δ / ppm = 137.7, 135.3, 129.4 (2C), 129.0 (2C), 74.6, 47.2, 38.6, 35.9, 30.1, 25.5, 25.0, 21.1.

IR (Diamond-ATR, neat): $\tilde{\nu}$ / cm^{-1} = 3342, 2921, 2854, 1514, 1447, 1067, 1058, 1029, 1021, 807, 778.

MS (EI, 70 eV): *m/z* (%) = 187 (14), 186 (100), 171 (34), 157 (13), 144 (18), 143 (34), 157 (13), 144 (18), 143 (34), 131 (12), 129 (34), 128 (14), 118 (69), 117 (30), 115 (20), 106 (41), 105 (86), 103 (12), 91 (66), 81 (12), 80 (10), 79 (30), 77 (13).

HRMS (EI-orbitrap): *m/z:* [M] calc. for [$C_{14}H_{20}O$]: 204.1514; found 204.1509.

m.p. (°C): 99.3 – 101.2.

2-methyl-4-phenylbutan-2-ol (10d)

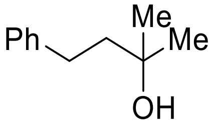

According to **TP2,** solutions of TMEDA (0.4 M, 2.0 equiv) in toluene (**3b**) and 3-(chloromethyl)heptane (**2**, 0.2 M, 2.0 equiv) in *n*-hexane were prepared. The solution of **2** was pumped through the activated sodium packed-bed reactor (see **TP1**) by pump A (flow rate: 2.0 mL/min) into the precooling loop (V_{pre2} = 0.35 mL) at 25 °C. The solution of **3b** was pumped by pump B (flow rate: 1.0 mL/min) through a precooling loop (V_{pre3} = 2.00 mL), at 25 °C. The solutions were mixed with an overall flow rate of 3.0 mL/min in a T-shaped mixer. The combined stream passed through a tube reactor connected to a metal needle (V_{R2} = 23.0 mL, t_{R2} = 460 s). Subsequently upon reaching the steady state, it was injected into a flask charged with 2,2-dimethyloxirane (**9b**, 29 mg, 0.40 mmol, 1.0 equiv) in THF (1.0 mL) at –20 °C for 2 min. The reaction mixture was stirred at –20 °C for 10 min followed by another 30 min at 25 °C, before sat. *aq.* NH_4Cl solution was added to quench the reaction mixture. The aqueous layer was extracted three times with EtOAc (3×30 mL) and the combined organic layers were dried over anhydrous $MgSO_4$ and filtrated. After removal of the solvent, flash column chromatographical purification (silica gel, isohexane:EtOAc = 95:5 → 80:20) afforded the title compound **10d** as a colorless oil (41 mg, 0.25 mmol, 62% yield).

^{1}H-NMR (400 MHz, $CDCl_3$): δ / ppm = 7.34 – 7.27 (m, 2H), 7.25 – 7.16 (m, 3H), 2.78 – 2.66 (m, 2H), 1.86 – 1.74 (m, 2H), 1.46 (s, 1H), 1.31 (s, 6H).

^{13}C-NMR (100 MHz, $CDCl_3$): δ / ppm = 142.6, 128.5 (2C), 128.4 (2C), 125.8, 71.0, 45.9, 30.9, 29.4 (2C).

IR (Diamond-ATR, neat): $\tilde{\nu}$ / cm^{-1} = 3366, 2968, 2967, 2931, 1494, 1454, 1376, 1365, 1210, 1150, 1124, 926, 913, 738, 696.

MS (EI, 70 eV): *m/z* (%) = 146 (37), 132 (10), 131 (100), 129 (10), 91 (78), 59 (10).

HRMS (EI-orbitrap): *m/z:* [M – H_2O] calc. for [$C_{11}H_{14}$]: 146.1096; found 146.1090.

2-methyl-4-(p-tolyl)butan-2-ol (10e)

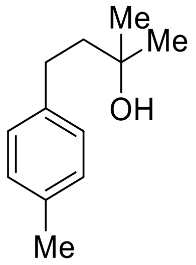

According to **TP2,** solutions of TMEDA (0.4 M, 2.0 equiv) in *p*-xylene (**3c**) and 3-(chloromethyl)heptane (**2**, 0.2 M, 2.0 equiv) in *n*-hexane were prepared. The solution of **2** was pumped through the activated sodium packed-bed reactor (see **TP1**) by pump A (flow rate: 2.0 mL/min) into the precooling loop (V_{pre2} = 0.35 mL) at 25 °C. The solution of **3c** was pumped by pump B (flow rate: 1.0 mL/min) through a precooling loop (V_{pre3} = 2.00 mL), at 25 °C. The solutions were mixed with an overall flow rate of 3.0 mL/min in a T-shaped mixer. The combined stream passed through a tube reactor connected to a metal needle (V_{R2} = 23.0 mL, t_{R2} = 460 s). Subsequently upon reaching the steady state, it was injected into a flask charged with 2,2-dimethyloxirane (**9b**, 29 mg, 0.40 mmol, 1.0 equiv) in THF (1.0 mL) at –20 °C for 2 min. The reaction mixture was stirred at –20 °C for 10 min followed by another 30 min at 25 °C, before sat. *aq.* NH_4Cl solution was added to quench the reaction mixture. The aqueous layer was extracted three times with EtOAc (3×30 mL) and the combined organic layers were dried over anhydrous $MgSO_4$ and filtrated. After removal of the solvent, flash column chromatographical purification (silica gel, isohexane:EtOAc = 95:5 → 80:20) afforded the title compound **10e** as a colorless oil (70 mg, 0.39 mmol, 98% yield).

^{1}H-NMR (400 MHz, $CDCl_3$): δ / ppm = 7.12 (s, 4H), 2.73 – 2.59 (m, 2H), 2.34 (s, 3H), 1.84 – 1.72 (m, 2H), 1.46 (s, 1H), 1.30 (s, 6H).

^{13}C-NMR (100 MHz, $CDCl_3$): δ / ppm = 139.5, 135.3, 129.2 (2C), 128.3 (2C), 71.0, 46.0, 30.4, 29.4 (2C), 21.1.

IR (Diamond-ATR, neat): $\tilde{\nu}$ / cm^{-1} = 3364, 2968, 2926, 1515, 1468, 1455, 1377, 1364, 1210, 1150, 1128, 1100, 925, 909, 808.

MS (EI, 70 eV): *m/z* (%) = 160 (32), 146 (12), 145 (100), 117 (16), 115 (11), 105 (57), 91 (11).

HRMS (EI-orbitrap): *m/z:* [M – H_2O] calc. for [$C_{12}H_{16}$]: 160.1252; found 160.1245.

m.p. (°C): 50.4 – 52.3.

4-(3,5-dimethylphenyl)-2-methylbutan-2-ol (10f)

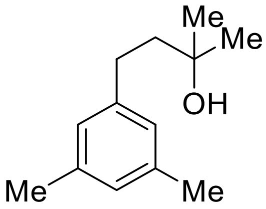

According to **TP2,** solutions of TMEDA (0.4 M, 2.0 equiv) in mesitylene (**3a**) and 3-(chloromethyl)heptane (**2**, 0.2 M, 2.0 equiv) in *n*-hexane were prepared. The solution of **2** was pumped through the activated sodium packed-bed reactor (see **TP1**) by pump A (flow rate: 2.0 mL/min) into the precooling loop (V_{pre2} = 0.35 mL) at 25 °C. The solution of **3a** was pumped by pump B (flow rate: 1.0 mL/min) through a precooling loop (V_{pre3} = 2.00 mL), at 25 °C. The solutions were mixed with an overall flow rate of 3.0 mL/min in a T-shaped mixer. The combined stream passed through a tube reactor connected to a metal needle (V_{R2} = 23.0 mL, t_{R2} = 460 s). Subsequently upon reaching the steady state, it was injected into a flask charged 2,2-dimethyloxirane (**9b**, 29 mg, 0.40 mmol, 1.0 equiv) in THF (1.0 mL) at –20 °C for 2 min. The reaction mixture was stirred at –20 °C for 10 min followed by another 30 min at 25 °C, before sat. *aq.* NH_4Cl solution was added to quench the reaction mixture. The aqueous layer was extracted three times with EtOAc (3×30 mL) and the combined organic layers were dried over anhydrous $MgSO_4$ and filtrated. After removal of the solvent, flash column chromatographical purification (silica gel, isohexane:EtOAc = 95:5 → 85:15) afforded the title compound **10f** as a colorless oil (74 mg, 0.38 mmol, 96% yield).

^{1}H-NMR (400 MHz, $CDCl_3$): δ / ppm = 6.87 (s, 3H), 2.77 – 2.52 (m, 2H), 2.33 (s, 6H), 1.92 – 1.71 (m, 2H), 1.54 (s, 1H), 1.32 (s, 6H).

^{13}C-NMR (100 MHz, $CDCl_3$): δ / ppm = 142.6, 138.0 (2C), 127.5, 126.3 (2C), 71.0, 46.0, 30.7, 29.4 (2C), 21.4 (2C).

IR (Diamond-ATR, neat): $\tilde{\nu}$ / cm^{-1} = 3374, 3014, 2969, 2942, 2920, 2864, 1606, 1468, 1376, 1364, 1210, 1149, 1120, 922, 910, 844, 699.

MS (EI, 70 eV): *m/z* (%) = 174 (40), 160 (13), 159 (100), 144 (10), 131 (13), 119 (69), 117 (14), 115 (10), 91 (18),

HRMS (EI-orbitrap): *m/z:* [M – H_2O] calc. for [$C_{13}H_{18}$]: 174.1409; found 174.1402.

1-(3,5-dimethylphenyl)pentan-3-ol (10g)

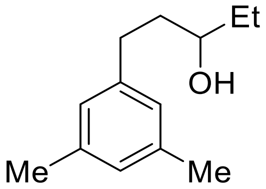

According to **TP2,** solutions of TMEDA (0.4 M, 2.0 equiv) in mesitylene (**3a**) and 3-(chloromethyl)heptane (**2**, 0.2 M, 2.0 equiv) in *n*-hexane were prepared. The solution of **2** was pumped through the activated sodium packed-bed reactor (see **TP1**) by pump A (flow rate: 2.0 mL/min) into the precooling loop (V_{pre2} = 0.35 mL) at 25 °C. The solution of **3a** was pumped by pump B (flow rate: 1.0 mL/min) through a precooling loop (V_{pre3} = 2.00 mL), at 25 °C. The solutions were mixed with an overall flow rate of 3.0 mL/min in a T-shaped mixer. The combined stream passed through a tube reactor connected to a metal needle (V_{R2} = 23.0 mL, t_{R2} = 460 s). Subsequently upon reaching the steady state, it was injected into a flask charged with 2-ethyloxirane (**9c**, 29 mg, 0.40 mmol, 1.0 equiv) in THF (1.0 mL) at –20 °C for 2 min. The reaction mixture was stirred at –20 °C for 10 min followed by another 30 min at 25 °C, before sat. *aq.* NH_4Cl solution was added to quench the reaction mixture. The aqueous layer was extracted three times with EtOAc (3×30 mL) and the combined organic layers were dried over anhydrous $MgSO_4$ and filtrated. After removal of the solvent, flash column chromatographical purification (silica gel, isohexane:EtOAc = 95:5 → 85:15) afforded the title compound **10g** as a colorless oil (73 mg, 0.38 mmol, 95% yield).

^{1}H-NMR (400 MHz, CDCl$_3$): δ / ppm = 6.86 (s, 3H), 3.65 – 3.51 (m, 1H), 2.81 – 2.69 (m, 1H), 2.69 – 2.57 (m, 1H), 2.32 (s, 6H), 1.87 – 1.67 (m, 2H), 1.65 – 1.42 (m, 3H), 0.98 (t, *J* = 7.5 Hz, 3H).

^{13}C-NMR (100 MHz, CDCl$_3$): δ / ppm = 142.3, 138.0 (2C), 127.6, 126.4 (2C), 72.9, 38.8, 32.1, 30.4, 21.4 (2C), 10.0.

IR (Diamond-ATR, neat): $\tilde{\nu}$ / cm^{-1} = 3343, 3012, 2961, 2959, 2933, 2929, 2918, 2874, 2859, 1606, 1456, 1119, 1035, 948, 843, 702.

MS (EI, 70 eV): *m/z* (%) = 145 (32), 133 (12), 131 (15), 120 (78), 120 (10), 119 (100), 117 (58), 115 (56), 115 (56), 105 (84), 103 (19), 91 (93), 79 (15), 78 (14), 77 (27).

HRMS (EI-orbitrap): *m/z:* [M] calc. for [$C_{13}H_{20}O$]: 192.1514; found 192.1507.

(*S*)-4-(3,5-dimethylphenyl)butan-2-ol (10h)

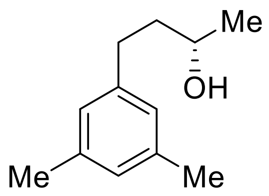

According to **TP2,** solutions of TMEDA (0.4 M, 2.0 equiv) in mesitylene (**3a**) and 3-(chloromethyl)heptane (**2**, 0.2 M, 2.0 equiv) in *n*-hexane were prepared. The solution of **2** was pumped through the activated sodium packed-bed reactor (see **TP1**) by pump A (flow rate: 2.0 mL/min) into the precooling loop (V_{pre2} = 0.35 mL) at 25 °C. The solution of **3a** was pumped by pump B (flow rate: 1.0 mL/min) through a precooling loop (V_{pre3} = 2.00 mL), at 25 °C. The solutions were mixed with an overall flow rate of 3.0 mL/min in a T-shaped mixer. The combined stream passed through a tube reactor connected to a metal needle (V_{R2} = 23.0 mL, t_{R2} = 460 s). Subsequently upon reaching the steady state, it was injected into a flask charged with (*S*)-2-methyloxirane (**9d**, 23 mg, 0.40 mmol, 1.0 equiv) in THF (1.0 mL) at –20 °C for 2 min. The reaction mixture was stirred at –20 °C for 10 min followed by another 30 min at 25 °C, before sat. *aq.* NH_4Cl solution was added to quench the reaction mixture. The aqueous layer was extracted three times with EtOAc (3×30 mL) and the combined organic layers were dried over anhydrous $MgSO_4$ and filtrated. After removal of the solvent, flash column chromatographical purification (silica gel, isohexane:EtOAc = 95:5 → 85:15) afforded the title compound **10h** as a colorless oil (64 mg, 0.36 mmol, 90% yield, ee > 99:1).

^{1}H-NMR (400 MHz, $CDCl_3$): δ / ppm = 6.85 (s, 3H), 3.91 – 3.77 (m, 1H), 2.75 – 2.56 (m, 2H), 2.32 (s, 6H), 1.86 – 1.70 (m, 2H), 1.58 (s, 1H), 1.25 (d, *J* = 6.2 Hz, 3H).

^{13}C-NMR (100 MHz, $CDCl_3$): δ / ppm = 142.1, 138.0 (2C), 127.6, 126.3 (2C), 67.7, 41.0, 32.1, 23.7, 21.4 (2C).

IR (Diamond-ATR, neat): $\tilde{\nu}$ / cm^{-1} = 3340, 3014, 2965, 2919, 2861, 1606, 1460, 1374, 1128, 1072, 840, 702.

MS (EI, 70 eV): *m/z* (%) = 145 (59), 120 (100), 119 (37), 117 (26), 115 (18), 105 (91), 91 (27).

HRMS (EI-orbitrap): *m/z:* [M] calc. for [$C_{12}H_{18}O$]: 178.1358; found 178.1351.

Chiral HPLC: >99% *ee*, OD-H column, heptane:*i*-PrOH = 99.3:0.7, 1.0 mL/min, 30 °C.

1-phenylbutan-1-ol (10i)

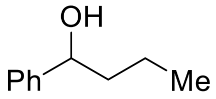

According to **TP2,** solutions of TMEDA (0.4 M, 2.0 equiv) in benzene (**3d**) and 3-(chloromethyl)heptane (**2**, 0.2 M, 2.0 equiv) in *n*-hexane were prepared. The solution of **2** was pumped through the activated sodium packed-bed reactor (see **TP1**) by pump A (flow rate: 2.0 mL/min) into the precooling loop (V_{pre2} = 0.35 mL) at 25 °C. The solution of **3d** was pumped by pump B (flow rate: 1.0 mL/min) through a precooling loop (V_{pre3} = 2.00 mL), at 25 °C. The solutions were mixed with an overall flow rate of 3.0 mL/min in a T-shaped mixer. The combined stream passed through a tube reactor connected to a metal needle (V_{R2} = 5.0 mL, t_{R2} = 100 s). Subsequently upon reaching the steady state, it was injected into a flask charged with butyraldehyde (**9e**, 29 mg, 0.40 mmol, 1.0 equiv) in THF (1.0 mL) at –20 °C for 2 min. The reaction mixture was stirred at –20 °C for 10 min followed by another 30 min at 25 °C, before sat. *aq.* NH_4Cl solution was added to quench the reaction mixture. The aqueous layer was extracted three times with EtOAc (3×30 mL) and the combined organic layers were dried over anhydrous $MgSO_4$ and filtrated. After removal of the solvent, flash column chromatographical purification (silica gel, pentane:EtOAc = 99:1 → 95:5) afforded the title compound **10i** as a colorless oil (44 mg, 0.29 mmol, 73% yield).

^{1}H-NMR (400 MHz, $CDCl_3$): δ / ppm = 7.35 (d, *J* = 4.4 Hz, 4H), 7.31 – 7.24 (m, 1H), 4.68 (dd, *J* = 7.6, 5.8 Hz, 1H), 1.86 – 1.74 (m, 2H), 1.74 – 1.62 (m, 1H), 1.51 – 1.39 (m, 1H), 1.37 – 1.22 (m, 1H), 0.93 (t, *J* = 7.4 Hz, 3H).

^{13}C-NMR (100 MHz, $CDCl_3$): δ / ppm = 145.1, 128.6 (2C), 127.6, 126.0 (2C), 74.6, 41.4, 19.2, 14.1.

IR (Diamond-ATR, neat): $\tilde{\nu}$ / cm^{-1} = 3360, 2958, 2932, 2872, 1454, 1028, 762, 700.

MS (EI, 70 eV): *m/z* (%) = 107 (91), 79 (100), 77 (25).

HRMS (EI-orbitrap): *m/z:* [M] calc. for [$C_{10}H_{14}O$]: 150.1045; found 150.1038.

1-phenylbutan-2-ol (10j)

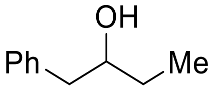

According to **TP2,** solutions of TMEDA (0.4 M, 2.0 equiv) in toluene (**3b**) and 3-(chloromethyl)heptane (**2**, 0.2 M, 2.0 equiv) in *n*-hexane were prepared. The solution of **2** was pumped through the activated sodium packed-bed reactor (see **TP1**) by pump A (flow rate: 2.0 mL/min) into the precooling loop (V_{pre2} = 0.35 mL) at 25 °C. The solution of **3b** was pumped by pump B (flow rate: 1.0 mL/min) through a precooling loop (V_{pre3} = 2.00 mL), at 25 °C. The solutions were mixed with an overall flow rate of 3.0 mL/min in a T-shaped mixer. The combined stream passed through a tube reactor connected to a metal needle (V_{R2} = 23.0 mL, t_{R2} = 460 s). Subsequently upon reaching the steady state, it was injected into a flask charged with propionaldehyde (**9f**, 23 mg, 0.40 mmol, 1.0 equiv) in THF (1.0 mL) at –20 °C for 2 min. The reaction mixture was stirred at –20 °C for 10 min followed by another 30 min at 25 °C, before sat. *aq.* NH_4Cl solution was added to quench the reaction mixture. The aqueous layer was extracted three times with EtOAc (3×30 mL) and the combined organic layers were dried over anhydrous $MgSO_4$ and filtrated. After removal of the solvent, flash column chromatographical purification (silica gel, isohexane:EtOAc = 90:10 → 80:20) afforded the title compound **10j** as a colorless oil (44 mg, 0.29 mmol, 73% yield).

^{1}H-NMR (400 MHz, $CDCl_3$): δ / ppm = 7.36 – 7.29 (m, 2H), 7.26 – 7.20 (m, 3H), 3.83 – 3.69 (m, 1H), 2.84 (dd, *J* = 13.5, 4.3 Hz, 1H), 2.65 (dd, *J* = 13.6, 8.4 Hz, 1H), 1.68 – 1.41 (m, 3H), 1.00 (t, *J* = 7.4 Hz, 3H).

^{13}C-NMR (100 MHz, $CDCl_3$): δ / ppm = 138.8, 129.6 (2C), 128.7 (2C), 126.6, 74.2, 43.7, 29.7, 10.2.

IR (Diamond-ATR, neat): $\tilde{\nu}$ / cm^{-1} = 3378, 2956, 2932, 2871, 1495, 1454, 1123, 1080, 1026, 1015, 745, 738, 699.

MS (EI, 70 eV): *m/z* (%) = 103 (13), 92 (95), 91 (100).

HRMS (EI-orbitrap): *m/z:* [M – H] calc. for [$C_{10}H_{13}O$]: 149.0966; found 149.0961.

4-phenylbutan-2-ol (10k)

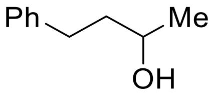

According to **TP2,** solutions of TMEDA (0.4 M, 2.0 equiv) in toluene (**3b**) and 3-(chloromethyl)heptane (**2**, 0.2 M, 2.0 equiv) in *n*-hexane were prepared. The solution of **2** was pumped through the activated sodium packed-bed reactor (see **TP1**) by pump A (flow rate: 2.0 mL/min) into the precooling loop (V_{pre2} = 0.35 mL) at 25 °C. The solution of **3b** was pumped by pump B (flow rate: 1.0 mL/min) through a precooling loop (V_{pre3} = 2.00 mL), at 25 °C. The solutions were mixed with an overall flow rate of 3.0 mL/min in a T-shaped mixer. The combined stream passed through a tube reactor connected to a metal needle (V_{R2} = 23.0 mL, t_{R2} = 460 s). Subsequently upon reaching the steady state, it was injected into a flask charged with 2-methyloxirane (**9g**, 23 mg, 0.40 mmol, 1.0 equiv) in THF (1.0 mL) at –20 °C for 2 min. The reaction mixture was stirred at –20 °C for 10 min followed by another 30 min at 25 °C, before sat. *aq.* NH_4Cl solution was added to quench the reaction mixture. The aqueous layer was extracted three times with EtOAc (3×30 mL) and the combined organic layers were dried over anhydrous $MgSO_4$ and filtrated. After removal of the solvent, flash column chromatographical purification (silica gel, isohexane:EtOAc = 90:10 → 80:20) afforded the title compound **10k** as a colorless oil (40 mg, 0.27 mmol, 67% yield).

^{1}H-NMR (400 MHz, $CDCl_3$): δ / ppm = 7.33 – 7.26 (m, 2H), 7.24 – 7.16 (m, 3H), 3.84 (h, *J* = 6.2 Hz, 1H), 2.82 – 2.61 (m, 2H), 1.82 – 1.74 (m, 2H), 1.48 (s, 1H), 1.24 (d, *J* = 6.2 Hz, 3H).

^{13}C-NMR (100 MHz, $CDCl_3$): δ / ppm = 142.2, 128.5 (4C), 125.9, 67.6, 41.0, 32.3, 23.8.

IR (Diamond-ATR, neat): $\tilde{\nu}$ / cm^{-1} = 3350, 3027, 2966, 2928, 2860, 1496, 1454, 1374, 1128, 1082, 1055, 954, 746, 698.

MS (EI, 70 eV): *m/z* (%) = 132 (26), 117 (100), 115 (18), 92 (17), 91 (55).

HRMS (EI-orbitrap): *m/z:* [M] calc. for [$C_{10}H_{14}O$]: 150.1045; found 150.1038.

4-phenylbutan-1-ol (10l)

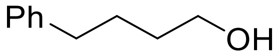

According to **TP2,** solutions of TMEDA (0.4 M, 2.0 equiv) in toluene (**3b**) and 3-(chloromethyl)heptane (**2**, 0.2 M, 2.0 equiv) in *n*-hexane were prepared. The solution of **2** was pumped through the activated sodium packed-bed reactor (see **TP1**) by pump A (flow rate: 2.0 mL/min) into the precooling loop (V_{pre2} = 0.35 mL) at 25 °C. The solution of **3b** was pumped by pump B (flow rate: 1.0 mL/min) through a precooling loop (V_{pre3} = 2.00 mL), at 25 °C. The solutions were mixed with an overall flow rate of 3.0 mL/min in a T-shaped mixer. The combined stream passed through a tube reactor connected to a metal needle (V_{R2} = 23.0 mL, t_{R2} = 460 s). Subsequently upon reaching the steady state, it was injected into a flask charged with oxetane (**9h**, 23 mg, 0.40 mmol, 1.0 equiv) in THF (1.0 mL) at –20 °C for 2 min. The reaction mixture was stirred at –20 °C for 10 min followed by another 30 min at 25 °C, before sat. *aq.* NH_4Cl solution was added to quench the reaction mixture. The aqueous layer was extracted three times with EtOAc (3×30 mL) and the combined organic layers were dried over anhydrous $MgSO_4$ and filtrated. After removal of the solvent, flash column chromatographical purification (silica gel, isohexane:EtOAc = 95:5 → 80:20) afforded the title compound **10l** as a colorless oil (50 mg, 0.33 mmol, 83% yield).

^{1}H-NMR (400 MHz, $CDCl_3$): δ / ppm = 7.34 – 7.27 (m, 2H), 7.25 – 7.16 (m, 3H), 3.65 (t, *J* = 6.5 Hz, 2H), 2.66 (t, *J* = 7.5 Hz, 2H), 1.88 (s, 1H), 1.78 – 1.66 (m, 2H), 1.66 – 1.54 (m, 2H).

^{13}C-NMR (100 MHz, $CDCl_3$): δ / ppm = 142.4, 128.5 (2C), 128.4 (2C), 125.8, 62.7, 35.7, 32.3, 27.6.

IR (Diamond-ATR, neat): $\tilde{\nu}$ / cm^{-1} = 3324, 3025, 2934, 2858, 1603, 1495, 1452, 1058, 1029, 982, 936, 745, 696.

MS (EI, 70 eV): *m/z* (%) = 132 (23), 117 (30), 115 (15), 104 (100), 91 (75), 78 (12).

HRMS (EI-orbitrap): *m/z:* [M] calc. for [$C_{10}H_{14}O$]: 150.1045; found 150.1039.

4-(p-tolyl)butan-1-ol (10m)

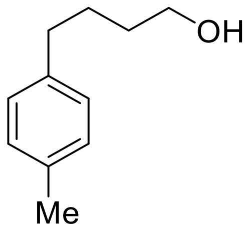

According to **TP2,** solutions of TMEDA (0.4 M, 2.0 equiv) in *p*-xylene (**3c**) and 3-(chloromethyl)heptane (**2**, 0.2 M, 2.0 equiv) in *n*-hexane were prepared. The solution of **2** was pumped through the activated sodium packed-bed reactor (see **TP1**) by pump A (flow rate: 2.0 mL/min) into the precooling loop (V_{pre2} = 0.35 mL) at 25 °C. The solution of **3c** was pumped by pump B (flow rate: 1.0 mL/min) through a precooling loop (V_{pre3} = 2.00 mL), at 25 °C. The solutions were mixed with an overall flow rate of 3.0 mL/min in a T-shaped mixer. The combined stream passed through a tube reactor connected to a metal needle (V_{R2} = 23.0 mL, t_{R2} = 460 s). Subsequently upon reaching the steady state, it was injected into a flask charged with oxetane (**9h**, 23 mg, 0.40 mmol, 1.0 equiv) in THF (1.0 mL) at –20 °C for 2 min. The reaction mixture was stirred at –20 °C for 10 min followed by another 30 min at 25 °C, before sat. *aq.* NH_4Cl solution was added to quench the reaction mixture. The aqueous layer was extracted three times with EtOAc (3×30 mL) and the combined organic layers were dried over anhydrous $MgSO_4$ and filtrated. After removal of the solvent, flash column chromatographical purification (silica gel, isohexane:EtOAc = 95:5 → 80:20) afforded the title compound **10m** as a colorless oil (51 mg, 0.31 mmol, 78% yield).

^{1}H-NMR (400 MHz, $CDCl_3$): δ / ppm = 7.10 (s, 4H), 3.66 (t, *J* = 6.4 Hz, 2H), 2.62 (t, *J* = 7.4 Hz, 2H), 2.33 (s, 3H), 1.76 – 1.49 (m, 5H).

^{13}C-NMR (100 MHz, $CDCl_3$): δ / ppm = 139.3, 135.3, 129.1 (2C), 128.4 (2C), 62.9, 35.3, 32.4, 27.8, 21.1.

IR (Diamond-ATR, neat): $\tilde{\nu}$ / cm^{-1} = 3327, 2933, 2931, 2928, 2858, 1515, 1453, 1058, 1032, 1021, 805.

MS (EI, 70 eV): *m/z* (%) = 164 (20), 146 (19), 131 (43), 129 (14), 128 (11), 119 (10), 118 (100), 117 (47), 115 (22), 105 (83), 103 (16), 91 (26), 79 (15), 77 (10), 45 (11), 44 (91, 43 (15), 42 (24).

HRMS (EI-orbitrap): *m/z:* [M] calc. for [$C_{11}H_{16}O$]: 164.1201; found 164.1194.

4-(3,5-dimethylphenyl)butan-1-ol (10n)

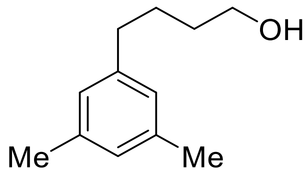

According to **TP2,** solutions of TMEDA (0.4 M, 2.0 equiv) in mesitylene (**3a**) and 3-(chloromethyl)heptane (**2**, 0.2 M, 2.0 equiv) in *n*-hexane were prepared. The solution of **2** was pumped through the activated sodium packed-bed reactor (see **TP1**) by pump A (flow rate: 2.0 mL/min) into the precooling loop (V_{pre2} = 0.35 mL) at 25 °C. The solution of **3a** was pumped by pump B (flow rate: 1.0 mL/min) through a precooling loop (V_{pre3} = 2.00 mL), at 25 °C. The solutions were mixed with an overall flow rate of 3.0 mL/min in a T-shaped mixer. The combined stream passed through a tube reactor connected to a metal needle (V_{R2} = 23.0 mL, t_{R2} = 460 s). Subsequently upon reaching the steady state, it was injected into a flask charged with oxetane (**9h**, 23 mg, 0.40 mmol, 1.0 equiv) in THF (1.0 mL) at –20 °C for 2 min. The reaction mixture was stirred at –20 °C for 10 min followed by another 30 min at 25 °C, before sat. *aq.* NH_4Cl solution was added to quench the reaction mixture. The aqueous layer was extracted three times with EtOAc (3×30 mL) and the combined organic layers were dried over anhydrous $MgSO_4$ and filtrated. After removal of the solvent, flash column chromatographical purification (silica gel, isohexane:EtOAc = 95:5 → 80:20) afforded the title compound **10n** as a colorless oil (48 mg, 0.27 mmol, 67% yield).

^{1}H-NMR (400 MHz, $CDCl_3$): δ / ppm = 6.84 (s, 1H), 6.82 (s, 2H), 3.67 (t, *J* = 6.3 Hz, 2H), 2.58 (t, *J* = 7.4 Hz, 2H), 2.30 (s, 6H), 1.74 – 1.55 (m, 4H), 1.43 (s, 1H).

^{13}C-NMR (100 MHz, $CDCl_3$): δ / ppm = 142.4, 137.9 (2C), 127.5, 126.4 (2C), 63.0, 35.6, 32.6, 27.7, 21.4 (2C).

IR (Diamond-ATR, neat): $\tilde{\nu}$ / cm^{-1} = 3326, 3014, 2934, 2920, 2859, 1606, 1460, 1376, 1062, 1037, 985, 843, 701.

MS (EI, 70 eV): *m/z* (%) = 178 (10), 160 (43), 145 (52), 132 (45), 120 (11), 119 (100), 117 (37), 115 (16), 107 (13), 105 (27), 91 (33).

HRMS (EI-orbitrap): *m/z:* [M] calc. for [$C_{12}H_{18}O$]: 178.1358; found 178.1351.

(2,6-dichlorophenyl)(phenyl)methanol (10o)

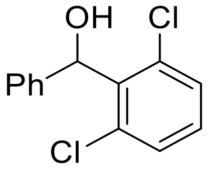

According to **TP2,** solutions of TMEDA (0.4 M, 2.0 equiv) in benzene (**3d**) and 3-(chloromethyl)heptane (**2**, 0.2 M, 2.0 equiv) in *n*-hexane were prepared. The solution of **2** was pumped through the activated sodium packed-bed reactor (see **TP1**) by pump A (flow rate: 2.0 mL/min) into the precooling loop (V_{pre2} = 0.35 mL) at 25 °C. The solution of **3d** was pumped by pump B (flow rate: 1.0 mL/min) through a precooling loop (V_{pre3} = 2.00 mL), at 25 °C. The solutions were mixed with an overall flow rate of 3.0 mL/min in a T-shaped mixer. The combined stream passed through a tube reactor connected to a metal needle (V_{R2} = 5.0 mL, t_{R2} = 100 s). Subsequently upon reaching the steady state, it was injected into a flask charged with 2,6-dichlorobenzaldehyde (**9i**, 70 mg, 0.40 mmol, 1.0 equiv) in THF (1.0 mL) at –20 °C for 2 min. The reaction mixture was stirred at –20 °C for 10 min followed by another 30 min at 25 °C, before sat. *aq.* NH_4Cl solution was added to quench the reaction mixture. The aqueous layer was extracted three times with EtOAc (3×30 mL) and the combined organic layers were dried over anhydrous $MgSO_4$ and filtrated. After removal of the solvent, flash column chromatographical purification (silica gel, pentane:EtOAc = 99:1 → 95:5) afforded the title compound **10o** as a colorless oil (76 mg, 0.30 mmol, 75% yield).

^{1}H-NMR (400 MHz, $CDCl_3$): δ / ppm = 7.38 – 7.27 (m, 7H), 7.22 (dd, *J* = 8.6, 7.5 Hz, 1H), 6.66 (d, *J* = 10.9 Hz, 1H), 3.43 (d, *J* = 10.9 Hz, 1H).

^{13}C-NMR (100 MHz, $CDCl_3$): δ / ppm = 141.6, 137.9, 135.3, 129.6 (2C), 129.5 (2C), 128.4 (2C), 127.4, 125.5 (2C), 72.5.

IR (Diamond-ATR, neat): $\tilde{\nu}$ / cm^{-1} = 3566, 3428, 1579, 1562, 1494, 1449, 1436, 1178, 1089, 1037, 1023, 829, 827, 779, 767, 737, 697.

MS (EI, 70 eV): *m/z* (%) = 254 (19), 252 (30), 251 (10), 199 (27), 176 (10), 175 (12), 175 (63), 173 (100), 165 (10), 163 (10), 152 (15), 146 (11), 79 (14), 78 (22), 77 (10).

HRMS (EI-orbitrap): *m/z:* [M] calc. for [$C_{13}H_{10}Cl_2O$]: 252.0109; found 252.0101.

dicyclopropyl(phenyl)methanol (10p)

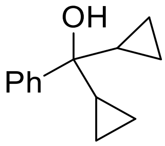

According to **TP2,** solutions of TMEDA (0.4 M, 2.0 equiv) in benzene (**3d**) and 3-(chloromethyl)heptane (**2**, 0.2 M, 2.0 equiv) in *n*-hexane were prepared. The solution of **2** was pumped through the activated sodium packed-bed reactor (see **TP1**) by pump A (flow rate: 2.0 mL/min) into the precooling loop (V_{pre2} = 0.35 mL) at 25 °C. The solution of **3d** was pumped by pump B (flow rate: 1.0 mL/min) through a precooling loop (V_{pre3} = 2.00 mL), at 25 °C. The solutions were mixed with an overall flow rate of 3.0 mL/min in a T-shaped mixer. The combined stream passed through a tube reactor connected to a metal needle (V_{R2} = 5.0 mL, t_{R2} = 100 s). Subsequently upon reaching the steady state, it was injected into a flask charged with dicyclopropylmethanone (**9j**, 44 mg, 0.40 mmol, 1.0 equiv) in THF (1.0 mL) at –20 °C for 2 min. The reaction mixture was stirred at –20 °C for 10 min followed by another 30 min at 25 °C, before sat. *aq.* NH_4Cl solution was added to quench the reaction mixture. The aqueous layer was extracted three times with EtOAc (3×30 mL) and the combined organic layers were dried over anhydrous $MgSO_4$ and filtrated. After removal of the solvent, flash column chromatographical purification (silica gel, isohexane:EtOAc = 99:1 → 95:5) afforded the title compound **10p** as a colorless oil (69 mg, 0.37 mmol, 92% yield).

^{1}H-NMR (400 MHz, CDCl$_3$): δ / ppm = 7.66 – 7.54 (m, 2H), 7.34 (t, *J* = 7.5 Hz, 2H), 7.30 – 7.21 (m, 1H), 1.47 (s, 1H), 1.25 – 1.15 (m, 2H), 0.69 – 0.47 (m, 4H), 0.46 – 0.26 (m, 4H).

^{13}C-NMR (100 MHz, CDCl$_3$): δ / ppm = 147.3, 127.9 (2C), 126.8, 125.9 (2C), 73.9, 20.8 (2C), 2.1 (2C), 0.3 (2C).

IR (Diamond-ATR, neat): $\tilde{\nu}$ / cm^{-1} = 3583, 3471, 3086, 3008, 1492, 1446, 1317, 1154, 1025, 995, 970, 916, 870, 851, 823, 780, 753, 699, 680.

MS (EI, 70 eV): *m/z* (%) = 160 (53), 159 (39), 155 (14), 147 (53), 146 (10), 145 (100), 142 (13), 141 (23), 131 (17), 129 (19), 128 (24), 127 (10), 118 (31), 117 (33), 115 (31), 105 (99), 91 (22), 90 (12), 77 (26).

HRMS (EI-orbitrap): *m/z:* [M – H] calc. for [$C_{13}H_{15}O$]: 187.1123; found 187.1115.

2-phenyladamantan-2-ol (10q)

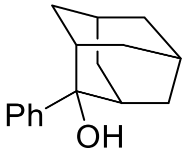

According to **TP2,** solutions of TMEDA (0.4 M, 2.0 equiv) in benzene (**3d**) and 3-(chloromethyl)heptane (**2**, 0.2 M, 2.0 equiv) in *n*-hexane were prepared. The solution of **2** was pumped through the activated sodium packed-bed reactor (see **TP1**) by pump A (flow rate: 2.0 mL/min) into the precooling loop (V_{pre2} = 0.35 mL) at 25 °C. The solution of **3d** was pumped by pump B (flow rate: 1.0 mL/min) through a precooling loop (V_{pre3} = 2.00 mL), at 25 °C. The solutions were mixed with an overall flow rate of 3.0 mL/min in a T-shaped mixer. The combined stream passed through a tube reactor connected to a metal needle (V_{R2} = 5.0 mL, t_{R2} = 100 s). Subsequently upon reaching the steady state, it was injected into a flask charged with adamantanone (**9k**, 60 mg, 0.40 mmol, 1.0 equiv) in THF (1.0 mL) at –20 °C for 2 min. The reaction mixture was stirred at –20 °C for 10 min followed by another 30 min at 25 °C, before sat. *aq.* NH_4Cl solution was added to quench the reaction mixture. The aqueous layer was extracted three times with EtOAc (3×30 mL) and the combined organic layers were dried over anhydrous $MgSO_4$ and filtrated. After removal of the solvent, flash column chromatographical purification (silica gel, isohexane:EtOAc = 99:1 → 95:5) afforded the title compound **10q** as a colorless solid (87 mg, 0.38 mmol, 95% yield).

^{1}H-NMR (400 MHz, CDCl$_3$): δ / ppm = 7.60 – 7.48 (m, 2H), 7.43 – 7.33 (m, 2H), 7.32 – 7.24 (m, 1H), 2.58 (s, 2H), 2.47 – 2.35 (m, 2H), 1.91 (p, *J* = 3.1 Hz, 1H), 1.79 – 1.65 (m, 9H), 1.52 (s, 1H).

^{13}C-NMR (100 MHz, CDCl$_3$): δ / ppm = 145.5, 128.9 (2C), 127.5, 125.6 (2C), 75.8, 37.8, 35.8 (2C), 35.0 (2C), 33.1 (2C), 27.6, 27.1.

IR (Diamond-ATR, neat): $\tilde{\nu}$ / cm^{-1} = 3354, 2909, 2854, 1449, 1102, 1043, 1005, 998, 969, 937, 914, 767, 697.

MS (EI, 70 eV): *m/z* (%) = 228 (24), 211 (16), 210 (100), 185 (22), 168 (11) 167 (15), 155 (10), 151 (14), 150 (19), 129 (10), 128 (10), 117 (10), 115 (14), 105 (79), 92 (11), 91 (21), 80 (11), 79 (28), 77 (26).

HRMS (EI-orbitrap): *m/z:* [M] calc. for [$C_{16}H_{20}O$]: 228.1514; found 228.1504.

m.p. (°C): 79.7 – 81.6.

N-benzhydrylaniline (10r)

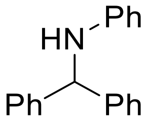

According to **TP2,** solutions of TMEDA (0.4 M, 2.0 equiv) in benzene (**3d**) and 3-(chloromethyl)heptane (**2**, 0.2 M, 2.0 equiv) in *n*-hexane were prepared. The solution of **2** was pumped through the activated sodium packed-bed reactor (see **TP1**) by pump A (flow rate: 2.0 mL/min) into the precooling loop (V_{pre2} = 0.35 mL) at 25 °C. The solution of **3d** was pumped by pump B (flow rate: 1.0 mL/min) through a precooling loop (V_{pre3} = 2.00 mL), at 25 °C. The solutions were mixed with an overall flow rate of 3.0 mL/min in a T-shaped mixer. The combined stream passed through a tube reactor connected to a metal needle (V_{R2} = 5.0 mL, t_{R2} = 100 s). Subsequently upon reaching the steady state, it was injected into a flask charged with *N*,1-diphenylmethanimine (**9l**, 72 mg, 0.40 mmol, 1.0 equiv) in THF (1.0 mL) at –20 °C for 2 min. The reaction mixture was stirred at –20 °C for 10 min followed by another 30 min at 25 °C, before sat. *aq.* NH_4Cl solution was added to quench the reaction mixture. The aqueous layer was extracted three times with EtOAc (3×30 mL) and the combined organic layers were dried over anhydrous $MgSO_4$ and filtrated. After removal of the solvent, flash column chromatographical purification (silica gel, isohexane:EtOAc = 99:1 → 95:5) afforded the title compound **10r** as a yellow resin (96 mg, 0.37 mmol, 93% yield).

^{1}H-NMR (400 MHz, $CDCl_3$): δ / ppm = 7.45 – 7.34 (m, 8H), 7.34 – 7.28 (m, 2H), 7.20 – 7.14 (m, 2H), 6.75 (t, *J* = 7.3 Hz, 1H), 6.63 – 6.57 (m, 2H), 5.56 (s, 1H), 4.28 (s, 1H).

^{13}C-NMR (100 MHz, $CDCl_3$): δ / ppm = 147.5, 143.0 (2C), 129.2 (2C), 128.9 (4C), 127.6 (4C), 127.5 (2C), 117.8, 113.6 (2C), 63.1.

IR (Diamond-ATR, neat): $\tilde{\nu}$ / cm^{-1} = 3410, 3058, 3053, 3026, 1600, 1586, 1501, 1451, 1426, 1314, 1267, 1028, 747, 699.

MS (EI, 70 eV): *m/z* (%) = 168 (13), 167 (100), 165 (39), 152 (23).

HRMS (EI-orbitrap): *m/z:* [M] calc. for [$C_{19}H_{17}N$]: 259.1361; found 259.1356.

2-benzyladamantan-2-ol (10s)

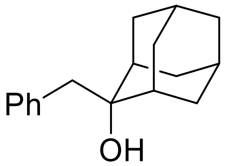

According to **TP3**, a solution of 3-(chloromethyl)heptane (**2**, 0.2 M, 2.0 equiv) in *n*-hexane was prepared. The solution of **2** was pumped through the activated sodium packed-bed reactor (see **TP1**) by pump A (flow rate: 2.0 mL/min) into the precooling loop (V_{pre2} = 0.35 mL) at 25 °C. Subsequently upon reaching the steady state, it was injected (2 min) into a flask charged with TMEDA (0.80 mmol, 2.0 equiv) in toluene (**3b**, 1.0 mL) and the resulting mixture was stirred at 25 °C for 30 min. The reaction flask was cooled to –20 °C and a solution of adamantanone (**9k**, 60 mg, 0.40 mmol, 1.0 equiv) in THF (1.0 mL) was added The reaction mixture was stirred at –20 °C for 10 min followed by another 30 min at 25 °C, before sat. *aq.* NH_4Cl solution was added for quenching the reaction mixture. The aqueous layer was extracted three times with EtOAc (3×30 mL) and the combined organic layers were dried over anhydrous $MgSO_4$ and filtrated. After removal of the solvent, flash column chromatographical purification (silica gel, pentane:EtOAc = 95:5 → 80:20) afforded the title compound **10s** as a colorless solid (90 mg, 0.37 mmol, 93% yield).

^{1}H-NMR (400 MHz, $CDCl_3$): δ / ppm = 7.37 – 7.29 (m, 2H), 7.29 – 7.22 (m, 3H), 3.02 (s, 2H), 2.25 – 2.05 (m, 4H), 1.99 – 1.90 (m, 1H), 1.87 – 1.76 (m, 3H), 1.76 – 1.65 (m, 4H), 1.59 – 1.49 (m, 2H), 1.41 (s, 1H).
^{13}C-NMR (100 MHz, $CDCl_3$): δ / ppm = 137.4, 130.8 (2C), 128.4 (2C), 126.6, 74.8, 44.0, 38.6, 37.0 (2C), 34.8 (2C), 33.1 (2C), 27.7, 27.5.
IR (Diamond-ATR, neat): $\tilde{\nu}$ / cm^{-1} = 3544, 3499, 3027, 2898, 2852, 1602, 1493, 1451, 1352, 1282, 1196, 1184, 1157, 1149, 1114, 1100, 1082, 1022, 1006, 987, 962, 930, 891, 879, 868, 778, 760, 700, 667.
MS (EI, 70 eV): *m/z* (%) = 225 (18), 224 (100), 223 (24), 181 (18), 167 (42), 166 (12), 165 (26), 155 (10), 153 (17), 152 (10), 151 (98), 150 (15), 142 (14), 141 (27), 133 (14), 129 (35), 128 (28), 117 (11), 115 (23), 105 (15), 91 (85), 79 (24), 77 (12).
HRMS (EI-orbitrap): *m/z:* [M – H_2O] calc. for [$C_{17}H_{20}$]: 224.1565; found 224.1562.
m.p. (°C): 61.7 – 63.4.

(*S*)-1-(benzyloxy)-4-phenylbutan-2-ol (10t)

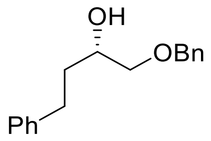

According to **TP3**, a solution of 3-(chloromethyl)heptane (**2**, 0.2 M, 2.0 equiv) in *n*-hexane was prepared. The solution of **2** was pumped through the activated sodium packed-bed reactor (see **TP1**) by pump A (flow rate: 2.0 mL/min) into the precooling loop (V_{pre2} = 0.35 mL) at 25 °C. Subsequently upon reaching the steady state, it was injected (2 min) into a flask charged with TMEDA (0.80 mmol, 2.0 equiv) in toluene (**3b**, 1.0 mL) and the resulting mixture was stirred at 25 °C for 30 min. The reaction flask was cooled to –20 °C and a solution of (*S*)-2-((benzyloxy)methyl)oxirane (**9m**, 66 mg, 0.40 mmol, 1.0 equiv) in THF (1.0 mL) was added The reaction mixture was stirred at –20 °C for 10 min followed by another 30 min at 25 °C, before sat. *aq.* NH_4Cl solution was added for quenching the reaction mixture. The aqueous layer was extracted three times with EtOAc (3×30 mL) and the combined organic layers were dried over anhydrous $MgSO_4$ and filtrated. After removal of the solvent, flash column chromatographical purification (silica gel, pentane:EtOAc = 90:10 → 85:15) afforded the title compound **10t** as a colorless oil (85 mg, 0.33 mmol, 83% yield).

^{1}H-NMR (400 MHz, $CDCl_3$): δ / ppm = 7.41 – 7.26 (m, 7H), 7.21 (d, *J* = 7.4 Hz, 3H), 4.56 (s, 2H), 3.85 (t, *J* = 7.6 Hz, 1H), 3.52 (dd, *J* = 9.4, 3.1 Hz, 1H), 3.37 (dd, *J* = 9.4, 7.7 Hz, 1H), 2.89 – 2.77 (m, 1H), 2.75 – 2.63 (m, 1H), 2.45 (s, 1H), 1.88 – 1.64 (m, 2H).

^{13}C-NMR (100 MHz, $CDCl_3$): δ / ppm = 142.0, 138.0, 128.6 (4C), 128.5 (2C), 127.9, 127.9 (2C), 126.0, 74.7, 73.5, 69.8, 34.9, 31.9.

IR (Diamond-ATR, neat): $\tilde{\nu}$ / cm^{-1} = 3442, 3062, 3027, 2922, 2859, 1603, 1495, 1453, 1365, 1310, 1258, 1207, 1155, 1118, 1088, 1075, 1028, 1002, 942, 908, 737, 696.

MS (EI, 70 eV): *m/z* (%) = 105 (39), 92 (23), 91 (100).

HRMS (EI-orbitrap): *m/z:* [M] calc. for [$C_{16}H_{20}O$]: 256.1463; found 256.1473.

(*S*)-1-((tert-butyldimethylsilyl)oxy)-4-phenylbutan-2-ol and (*S*)-2-((tert-butyldimethylsilyl)oxy)-4-phenylbutan-1-ol (10u)

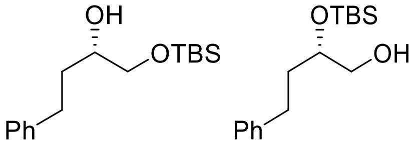

According to **TP3**, a solution of 3-(chloromethyl)heptane (**2**, 0.2 M, 2.0 equiv) in *n*-hexane was prepared. The solution of **2** was pumped through the activated sodium packed-bed reactor (see **TP1**) by pump A (flow rate: 2.0 mL/min) into the precooling loop (V_{pre2} = 0.35 mL) at 25 °C. Subsequently upon reaching the steady state, it was injected (2 min) into a flask charged with TMEDA (0.80 mmol, 2.0 equiv) in toluene (**3b**, 1.0 mL) and the resulting mixture was stirred at 25 °C for 30 min. The reaction flask was cooled to –20 °C and a solution of (*S*)-tert-butyldimethyl(oxiran-2-ylmethoxy)silane (**9n**, 75 mg, 0.40 mmol, 1.0 equiv) in THF (1.0 mL) was added The reaction mixture was stirred at –20 °C for 10 min followed by another 30 min at 25 °C, before sat. *aq.* NH_4Cl solution was added for quenching the reaction mixture. The aqueous layer was extracted three times with EtOAc (3×30 mL) and the combined organic layers were dried over anhydrous $MgSO_4$ and filtrated. After removal of the solvent, flash column chromatographical purification (silica gel, pentane:EtOAc = 97:3 → 90:10) afforded the title compounds as colorless oils (*S*)-1-((tert-butyldimethylsilyl)oxy)-4-phenylbutan-2-ol (86 mg, 0.31 mmol, 76% yield) and (*S*)-2-((tert-butyldimethylsilyl)oxy)-4-phenylbutan-1-ol (20 mg, 0.07 mmol, 18%). Combined yield: 106 mg, 0.38 mmol, 94%.

(*S*)-1-((tert-butyldimethylsilyl)oxy)-4-phenylbutan-2-ol

^{1}H-NMR (400 MHz, $CDCl_3$): δ / ppm = 7.32 – 7.27 (m, 2H), 7.20 (dd, *J* = 14.3, 7.1 Hz, 3H), 3.72 – 3.58 (m, 2H), 3.43 (dd, *J* = 9.7, 7.2 Hz, 1H), 2.84 (ddd, *J* = 15.2, 9.6, 5.6 Hz, 1H), 2.70 (ddd, *J* = 13.8, 9.5, 7.1 Hz, 1H), 2.48 (s, 1H), 1.83 – 1.64 (m, 2H), 0.91 (s, 9H), 0.08 (s, 6H).
^{13}C-NMR (100 MHz, $CDCl_3$): δ / ppm = 142.2, 128.6 (2C), 128.5 (2C), 126.0, 71.2, 67.3, 34.6, 32.0, 26.0, 18.4 (3C), -5.2, -5.3.
IR (Diamond-ATR, neat): $\tilde{\nu}$ / cm^{-1} = 2953, 2929, 2884, 2857, 1255, 1113, 1044, 835, 776, 698.
MS (EI, 70 eV): *m/z* (%) = 131 (100), 91 (24), 75 (40), 73 (19).
HRMS (EI-orbitrap): *m/z:* [M – CH_3 and – H_2O] calc. for [$C_{15}H_{23}OSi$]: 247.1518; found 247.1474.

(*S*)-2-((tert-butyldimethylsilyl)oxy)-4-phenylbutan-1-ol

^{1}H-NMR (400 MHz, $CDCl_3$): δ / ppm = 7.32 – 7.26 (m, 2H), 7.22 – 7.15 (m, 3H), 3.85 – 3.73 (m, 1H), 3.62 (dt, *J* = 11.2, 4.3 Hz, 1H), 3.53 (dt, *J* = 10.8, 5.2 Hz, 1H), 2.72 – 2.56 (m, 2H), 1.95 – 1.78 (m, 3H), 0.93 (s, 9H), 0.10 (s, 3H), 0.09 (s, 3H).

^{13}C-NMR (100 MHz, $CDCl_3$): δ / ppm = 142.2, 128.5 (2C), 128.4 (2C), 126.0, 72.5, 66.3, 35.9, 31.8, 26.0, 18.2 (3C), -4.3, -4.4.

IR (Diamond-ATR, neat): $\tilde{\nu}$ / cm^{-1} = 3399, 3028, 2953, 2929, 2884, 2857, 2359, 2343, 2332, 1496, 1472, 1462, 1456, 1388, 1374, 1361, 1255, 1113, 1044, 1004, 986, 979, 835, 811, 776, 748, 698, 668.

MS (EI, 70 eV): *m/z* (%) = 131 (100), 91 (24), 75 (40), 73 (19).

HRMS (EI-orbitrap): *m/z:* [M – CH_2OH] calc. for [$C_{15}H_{25}OSi$]: 249.1675; found 249.1705.

(S)-4-phenylbutane-1,2-diol (11u)

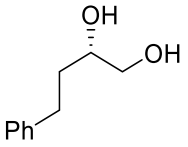

According to the literature[5] TBAF (1.0 M in THF, 2.0 mL) was added to a regioisomeric mixture of **10u** and **10u´** (90 mg, 0.32 mmol, 1.0 equiv), the mixture was stirred for 2.5 h at 25 °C. EtOAc (20 mL) was added to the mixture and the organic layer was washed with Brine (2x20 mL). The organic layer was dried over $MgSO_4$. The crude mixture was purified by flash column chramotography (silica gel, pentane:EtOAc = 88:12 → 85:15) to afforded the title compound **11u** as a colorless oil (50 mg, 0.30 mmol, 94%).

^{1}H-NMR (400 MHz, $CDCl_3$): δ / ppm = 7.32 – 7.23 (m, 2H), 7.20 (d, *J* = 7.3 Hz, 3H), 3.78 – 3.67 (m, 1H), 3.63 (dd, *J* = 11.2, 2.9 Hz, 1H), 3.45 (dd, *J* = 11.1, 7.6 Hz, 1H), 3.01 – 2.62 (m, 4H), 1.84 – 1.64 (m, 2H).
^{13}C-NMR (100 MHz, $CDCl_3$): δ / ppm = 141.8, 128.6 (2C), 128.5 (2C), 126.1, 71.7, 66.9, 34.7, 31.9.
IR (Diamond-ATR, neat): $\tilde{\nu}$ / cm^{-1} = 3334, 2927, 2862, 1496, 1454, 1098, 1067, 1038, 1032, 748, 698.
MS (EI, 70 eV): *m/z* (%) = 130 (15), 117 (51), 115 (13), 104 (16), 92 (11), 91 (100).
HRMS (EI-orbitrap): *m/z:* [M – H_2O] calc. for [$C_{10}H_{12}O$]: 148.0888; found 148.0882.

[5] M. Akehi, M. Kawamoto, T. Mandai, *Tetrahedron*. **2015**, *71*, 6488 6498.

***trans*-2-(naphthalen-1-ylmethyl)cyclohexan-1-ol (10v)**

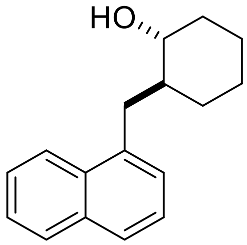

According to **TP4**, a solution of 3-(chloromethyl)heptane (**2**, 0.2 M, 2.0 equiv) in *n*-hexane was prepared. The solution of **2** was pumped through the activated sodium packed-bed reactor (see **TP1**) by pump A (flow rate: 2.0 mL/min) into the precooling loop (V_{pre2} = 0.35 mL) at 25 °C. Subsequently upon reaching the steady state, it was injected for 1 min into a flask charged with a solution of TMEDA (0.40 mmol, 2.0 equiv) and 1–methylnaphtalene (**3e**, 28 mg, 0.20 mmol, 1.0 equiv) in hexane (1.0 mL) the mixture was stirred for 30 min at 25 °C. Before it was cooled to –20 °C and a solution of cyclohexene oxide (**9a**, 39 mg, 0.40 mmol, 2.0 equiv) in THF (1.0 mL) was added. The reaction mixture was stirred at –20 °C for 10 min followed by another 30 min at 25 °C, before sat. *aq.* NH_4Cl solution was added for quenching the reaction mixture. The aqueous layer was extracted three times with EtOAc (3×30 mL) and the combined organic layers were dried over anhydrous $MgSO_4$ and filtrated. After removal of the solvent, flash column chromatographical purification (silica gel, pentane:EtOAc = 95:5 → 80:20) afforded the title compound **10v** as a colorless solid (44 mg, 0.18 mmol, 92% yield).

According to **TP5**, a solution of 3-(chloromethyl)heptane (**2**, 0.2 M, 2.0 equiv) in *n*-hexane was prepared. The solution of **2** was pumped through the activated sodium packed-bed reactor (see **TP1**) by pump A (flow rate: 2.0 mL/min) into the precooling loop (V_{pre2} = 0.35 mL) at 25 °C. Subsequently upon reaching the steady state, it was injected for 2 min into a flask charged with a solution of TMEDA (0.80 mmol, 2.0 equiv) and 1–methylnaphtalene (**3e**, 142 mg, 1.00 mmol, 2.5 equiv) in hexane (1.0 mL) the mixture was stirred for 30 min at 25 °C. Before it was cooled to –20 °C and a solution of cyclohexene oxide (**9a**, 39 mg, 0.40 mmol, 1.0 equiv) in THF (1.0 mL) was added. The reaction mixture was stirred at –20 °C for 10 min followed by another 30 min at 25 °C, before sat. *aq.* NH_4Cl solution was added for quenching the reaction mixture. The aqueous layer was extracted three times with EtOAc (3×30 mL) and the combined organic layers were dried over anhydrous $MgSO_4$ and filtrated. After removal of the solvent, flash column chromatographical purification (silica gel, pentane:EtOAc = 85:15) afforded the title compound **10v** as a colorless solid (94 mg, 0.39 mmol, 98% yield).

^{1}H-NMR (400 MHz, $CDCl_3$): δ / ppm = 8.20 (d, *J* = 8.4 Hz, 1H), 7.86 (dd, *J* = 7.9, 1.6 Hz, 1H), 7.73 (d, *J* = 8.2 Hz, 1H), 7.57 – 7.44 (m, 2H), 7.43 – 7.36 (m, 1H), 7.31 (d, *J* = 6.6 Hz, 1H), 3.91 (dd, *J* = 13.5, 3.4 Hz, 1H), 3.52 – 3.38 (m, 1H), 2.62 – 2.51 (m, 1H), 2.11 – 1.96 (m, 1H), 1.80 – 1.59 (m, 4H), 1.59 – 1.49 (m, 1H), 1.38 – 1.22 (m, 2H), 1.06 – 0.92 (m, 2H).

^{13}C-NMR (100 MHz, $CDCl_3$): δ / ppm = ^{13}C NMR (101 MHz, $CDCl_3$) δ 137.3, 134.1, 132.3, 128.8, 127.6, 126.8, 125.8, 125.5, 125.4, 124.6, 75.5, 46.4, 36.6, 36.1, 30.7, 25.5, 25.1.

IR (Diamond-ATR, neat): $\tilde{\nu}$ / cm^{-1} = 3375, 3306, 2935, 2918, 2894, 2882, 2854, 1509, 1449, 1442, 1393, 1360, 1126, 1064, 1047, 1026, 1014, 930, 875, 798, 776, 766.

MS (EI, 70 eV): *m/z* (%) = 240 (13), 179 (10), 165 (36), 153 (16), 152 (10), 143 (12), 142 (100), 141 (76), 115 (25).

HRMS (EI-orbitrap): *m/z:* [M] calc. for [$C_{17}H_{20}O$]: 240.1514; found 240.1508.

m.p. (°C): 83.8 – 85.6.

2-methyl-4-(naphthalen-1-yl)butan-2-ol (10w)

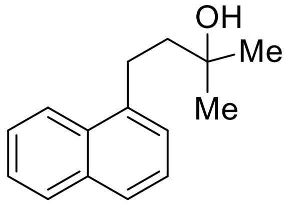

According to **TP4**, A solution of 3-(chloromethyl)heptane (**2**, 0.2 M, 2.0 equiv) in *n*-hexane was prepared. The solution of **2** was pumped through the activated sodium packed-bed reactor (see **TP1**) by pump A (flow rate: 2.0 mL/min) into the precooling loop (V_{pre2} = 0.35 mL) at 25 °C. Subsequently upon reaching the steady state, it was injected for 2 min into a flask charged with a solution of TMEDA (0.80 mmol, 2.0 equiv) and 1–methylnaphtalene (**3e**, 56 mg, 0.40 mmol, 1.0 equiv) in hexane (2.0 mL) the mixture was stirred for 30 min at 25 °C. Before it was cooled to –20 °C and a solution of 2,2-dicyclopropylmethanone (**9j**, 88 mg, 0.80 mmol, 2.0 equiv) in THF (2.0 mL) was added. The reaction mixture was stirred at –20 °C for 10 min followed by another 30 min at 25 °C, before sat. *aq.* NH_4Cl solution was added for quenching the reaction mixture. The aqueous layer was extracted three times with EtOAc (3×30 mL) and the combined organic layers were dried over anhydrous $MgSO_4$ and filtrated. After removal of the solvent, flash column chromatographical purification (silica gel, pentane:EtOAc = 95:5 → 80:20) afforded the title compound **10w** as a colorless oil (75 mg, 0.35 mmol, 87% yield).

^{1}H-NMR (400 MHz, $CDCl_3$): δ / ppm = 8.09 (d, *J* = 8.3 Hz, 1H), 7.88 (d, *J* = 9.0 Hz, 1H), 7.74 (d, *J* = 8.0 Hz, 1H), 7.60 – 7.46 (m, 2H), 7.45 – 7.39 (m, 1H), 7.37 (d, *J* = 6.3 Hz, 1H), 3.26 – 3.11 (m, 2H), 1.98 – 1.85 (m, 2H), 1.52 (s, 1H), 1.39 (s, 6H).

^{13}C-NMR (100 MHz, $CDCl_3$): δ / ppm = 138.8, 134.0, 131.9, 128.9, 126.7, 125.9, 125.9, 125.8, 125.6, 123.8, 71.2, 45.0, 29.4 (2C), 27.9.

IR (Diamond-ATR, neat): $\tilde{\nu}$ / cm^{-1} = 3385, 2968, 1597, 1510, 1467, 1396, 1376, 1364, 1279, 1217, 1165, 1150, 1121, 927, 911, 801, 781.

MS (EI, 70 eV): *m/z* (%) = 196 (29), 182 (15), 181 (100), 166 (23), 165 (17), 153 (36), 152 (11), 141 (68), 128 (10), 115 (23).

HRMS (EI-orbitrap): *m/z:* [M] calc. for [$C_{15}H_{18}O$]: 214.1358; found 214.1351.

4-(naphthalen-1-yl)butan-1-ol (10x)

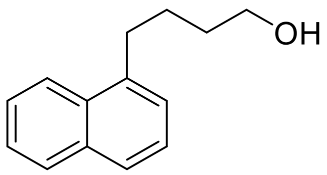

According to **TP4**, a solution of 3-(chloromethyl)heptane (**2**, 0.2 M, 2.0 equiv) in *n*-hexane was prepared. The solution of **2** was pumped through the activated sodium packed-bed reactor (see **TP1**) by pump A (flow rate: 2.0 mL/min) into the precooling loop (V_{pre2} = 0.35 mL) at 25 °C. Subsequently upon reaching the steady state, it was injected for 1 min into a flask charged with a solution of TMEDA (0.40 mmol, 2.0 equiv) and 1–methylnaphtalene (**3e**, 28 mg, 0.20 mmol, 1.0 equiv) in hexane (1.0 mL) the mixture was stirred for 30 min at 25 °C. Before it was cooled to –20 °C and a solution of oxetane (**9h**, 23 mg, 0.40 mmol, 2.0 equiv) in THF (2.0 mL) was added. The reaction mixture was stirred at –20 °C for 10 min followed by another 30 min at 25 °C, before sat. *aq.* NH_4Cl solution was added for quenching the reaction mixture. The aqueous layer was extracted three times with EtOAc (3×30 mL) and the combined organic layers were dried over anhydrous $MgSO_4$ and filtrated. After removal of the solvent, flash column chromatographical purification (silica gel, pentane:EtOAc = 95:5 → 75:25) afforded the title compound **10x** as a colorless oil (27 mg, 0.14 mmol, 68% yield).

According to **TP5**, a solution of 3-(chloromethyl)heptane (**2**, 0.2 M, 2.0 equiv) in *n*-hexane was prepared. The solution of **2** was pumped through the activated sodium packed-bed reactor (see **TP1**) by pump A (flow rate: 2.0 mL/min) into the precooling loop (V_{pre2} = 0.35 mL) at 25 °C. Subsequently upon reaching the steady state, it was injected for 2 min into a flask charged with a solution of TMEDA (0.80 mmol, 2.0 equiv) and 1–methylnaphtalene (**3e**, 142 mg, 1.00 mmol, 2.5 equiv) in hexane (2.0 mL) the mixture was stirred for 30 min at 25 °C. Before it was cooled to –20 °C and a solution of oxetane (**9h**, 23 mg, 0.40 mmol, 2.0 equiv) in THF (1.0 mL) was added. The reaction mixture was stirred at –20 °C for 10 min followed by another 30 min at 25 °C, before sat. *aq.* NH_4Cl solution was added for quenching the reaction mixture. The aqueous layer was extracted three times with EtOAc (3×30 mL) and the combined organic layers were dried over anhydrous $MgSO_4$ and filtrated. After removal of the solvent, flash column chromatographical purification (silica gel, pentane:EtOAc = 95:5 → 75:25) afforded the title compound **10v** as a colorless solid (55 mg, 0.27 mmol, 69% yield).

^{1}H-NMR (400 MHz, $CDCl_3$): δ / ppm = 8.06 (d, *J* = 8.4, 1.4 Hz, 1H), 7.93 – 7.82 (m, 1H), 7.73 (d, *J* = 8.1 Hz, 1H), 7.57 – 7.45 (m, 2H), 7.45 – 7.37 (m, 1H), 7.34 (d, *J* = 6.8 Hz, 1H), 3.68 (t, *J* = 6.5 Hz, 2H), 3.17 – 3.05 (m, 2H), 1.90 – 1.79 (m, 2H), 1.75 – 1.65 (m, 2H), 1.48 (s, 1H).

^{13}C-NMR (100 MHz, $CDCl_3$): δ / ppm = 138.5, 134.0, 131.9, 128.9, 126.7, 126.1, 125.8, 125.6, 125.5, 123.9, 62.9, 32.9, 32.8, 27.0.

IR (Diamond-ATR, neat): $\tilde{\nu}$ / cm^{-1} = 3327, 3045, 2934, 2863, 1596, 1510, 1462, 1395, 1354, 1261, 1166, 1056, 1029, 1011, 978, 796, 788, 774, 732.

MS (EI, 70 eV): *m/z* (%) = 200 (25), 167 (11), 154 (30), 153 (18), 142 (12), 141 (100), 115 (32).

HRMS (EI-orbitrap): *m/z:* [M] calc. for [$C_{14}H_{16}O$]: 200.1201; found 200.1195.

2-(naphthalen-1-yl)-1,1-diphenylethan-1-ol (10y)

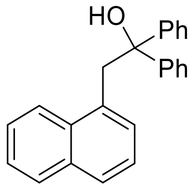

According to **TP5**, a solution of 3-(chloromethyl)heptane (**2**, 0.2 M, 2.0 equiv) in *n*-hexane was prepared. The solution of **2** was pumped through the activated sodium packed-bed reactor (see **TP1**) by pump A (flow rate: 2.0 mL/min) into the precooling loop (V_{pre2} = 0.35 mL) at 25 °C. Subsequently upon reaching the steady state, it was injected for 2 min into a flask charged with a solution of TMEDA (0.80 mmol, 2.0 equiv) and 1–methylnaphtalene (**3e**, 142 mg, 1.00 mmol, 2.5 equiv) in hexane (1.0 mL) the mixture was stirred for 30 min at 25 °C. Before it was cooled to –20 °C and a solution of benzophenone (**9o**, 73 mg, 0.40 mmol, 2.0 equiv) in THF (1.0 mL) was added. The reaction mixture was stirred at –20 °C for 10 min followed by another 30 min at 25 °C, before sat. *aq.* NH_4Cl solution was added for quenching the reaction mixture. The aqueous layer was extracted three times with EtOAc (3×30 mL) and the combined organic layers were dried over anhydrous $MgSO_4$ and filtrated. After removal of the solvent, flash column chromatographical purification (silica gel, pentane:EtOAc = 99:1 → 95:5) afforded the title compound **10y** as a colorless oil (105 mg, 0.32 mmol, 81% yield).

^{1}H-NMR (400 MHz, $CDCl_3$): δ / ppm = 8.08 (dd, *J* = 8.6, 1.5 Hz, 1H), 7.93 – 7.83 (m, 1H), 7.78 (d, *J* = 8.2 Hz, 1H), 7.54 – 7.43 (m, 6H), 7.40 – 7.34 (m, 4H), 7.34 – 7.27 (m, 3H), 7.01 – 6.89 (m, 1H), 4.22 (s, 2H), 2.43 (s, 1H).

^{13}C-NMR (100 MHz, $CDCl_3$): δ / ppm = 146.9 (2C), 133.9, 133.7, 132.0, 129.4, 128.7, 128.2 (4C), 127.6, 127.1 (2C), 126.4, 126.0 (4C), 125.5, 125.1, 124.6, 78.9, 43.7.

IR (Diamond-ATR, neat): $\tilde{\nu}$ / cm^{-1} = 3560, 3056, 1597, 1510, 1493, 1447, 1397, 1341, 1265, 1165, 1057, 1032, 1020, 1002, 944, 799, 781, 772, 756, 736, 698, 662.

MS (EI, 70 eV): *m/z* (%) = 184 (15), 183 (62), 183 (100), 183 (42), 142 (64), 141 (18), 115 (10), 105 (88), 77, (35).

HRMS (EI-orbitrap): *m/z:* [M] calc. for [$C_{24}H_{20}O$]: 324.1514; found 324.1506.

1,1-dicyclopropyl-2-(4-ethylphenyl)ethan-1-ol (10z)

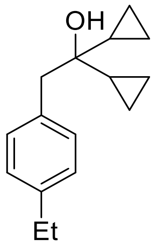

According to **TP4**, a solution of 3-(chloromethyl)heptane (**2**, 0.2 M, 2.0 equiv) in *n*-hexane was prepared. The solution of **2** was pumped through the activated sodium packed-bed reactor (see **TP1**) by pump A (flow rate: 2.0 mL/min) into the precooling loop (V_{pre2} = 0.35 mL) at 25 °C. Subsequently upon reaching the steady state, it was injected for 1 min into a flask charged with a solution of TMEDA (0.40 mmol, 2.0 equiv) and 4-ethyltoluene (**3f**, 24 mg, 0.20 mmol, 1.0 equiv) in hexane (1.0 mL) the mixture was stirred for 30 min at 25 °C. Before it was cooled to –20 °C and a solution of 2,2-dicyclopropylmethanone (**9j**, 44 mg, 0.40 mmol, 2.0 equiv) in THF (2.0 mL) was added. The reaction mixture was stirred at –20 °C for 10 min followed by another 30 min at 25 °C, before sat. *aq.* NH_4Cl solution was added for quenching the reaction mixture. The aqueous layer was extracted three times with EtOAc (3×30 mL) and the combined organic layers were dried over anhydrous $MgSO_4$ and filtrated. After removal of the solvent, flash column chromatographical purification (silica gel, pentane:EtOAc = 98:2 → 95:5) afforded the title compound **10z** as a colorless oil (43 mg, 0.19 mmol, 93% yield).

^{1}H-NMR (400 MHz, $CDCl_3$): δ / ppm = 7.22 (d, *J* = 8.0 Hz, 2H), 7.12 (d, *J* = 8.0 Hz, 2H), 2.85 (s, 2H), 2.64 (q, *J* = 7.6 Hz, 2H), 1.24 (t, *J* = 7.6 Hz, 3H), 0.92 (s, 1H), 0.84 – 0.71 (m, 2H), 0.45 – 0.31 (m, 6H), 0.31 – 0.19 (m, 2H).

^{13}C-NMR (100 MHz, $CDCl_3$): δ / ppm = 142.3, 134.8, 130.9 (2C), 127.5 (2C), 71.1, 48.3, 28.6, 18.5 (2C), 15.8, 1.2 (2C), -0.3 (2C).

IR (Diamond-ATR, neat): $\tilde{\nu}$ / cm^{-1} = 3588, 3484, 3086, 3049, 3008, 2962, 2925, 2871, 2855, 2360, 2342, 1514, 1463, 1458, 1437, 1420, 1376, 1320, 1247, 1181, 1122, 1045, 1022, 996, 929, 914, 821.

MS (EI, 70 eV): *m/z* (%) = 212 (16). 197 (20), 184 (12), 183 (82), 171 (51), 169 (36), 168 (19), 167 (23), 165 (22), 156 (12), 155 (92), 154 (23), 153 (51), 152 (25), 143 (33), 142 (20), 132 (12), 131 (100), 129 (31), 128 (48), 119 (13), 117 (20), 115 (38), 91 (45), 78 (11).

HRMS (EI-orbitrap): *m/z:* [M – H_2O] calc. for [$C_{16}H_{20}$]: 212.1565; found 212.1559.

***trans*-2-(4-ethylbenzyl)cyclohexan-1-ol (10aa)**

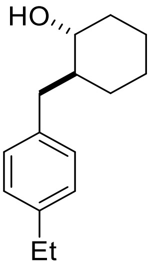

According to **TP4**, a solution of 3-(chloromethyl)heptane (**2**, 0.2 M, 2.0 equiv) in *n*-hexane was prepared. The solution of **2** was pumped through the activated sodium packed-bed reactor (see **TP1**) by pump A (flow rate: 2.0 mL/min) into the precooling loop (V_{pre2} = 0.35 mL) at 25 °C. Subsequently upon reaching the steady state, it was injected for 1 min into a flask charged with a solution of TMEDA (0.40 mmol, 2.0 equiv) and 4-ethyltoluene (**3f**, 24 mg, 0.20 mmol, 1.0 equiv) in hexane (1.0 mL) the mixture was stirred for 30 min at 25 °C. Before it was cooled to –20 °C and a solution of 2,2-cyclohexene oxide (**9a**, 39 mg, 0.40 mmol, 2.0 equiv) in THF (2.0 mL) was added. The reaction mixture was stirred at –20 °C for 10 min followed by another 30 min at 25 °C, before sat. *aq.* NH_4Cl solution was added for quenching the reaction mixture. The aqueous layer was extracted three times with EtOAc (3×30 mL) and the combined organic layers were dried over anhydrous $MgSO_4$ and filtrated. After removal of the solvent, flash column chromatographical purification (silica gel, pentane:EtOAc = 90:10 → 80:20) afforded the title compound **10aa** as colorless solid (41 mg, 0.19 mmol, 94% yield).

^{1}H-NMR (400 MHz, $CDCl_3$): δ / ppm = 7.11 (s, 4H), 3.36 – 3.23 (m, 1H), 3.12 (dd, *J* = 13.3, 4.1 Hz, 1H), 2.62 (q, *J* = 7.6 Hz, 2H), 2.34 (dd, *J* = 13.4, 9.1 Hz, 1H), 2.02 – 1.93 (m, 1H), 1.76 – 1.43 (m, 5H), 1.33 – 1.18 (m, 5H), 1.09 (qt, *J* = 12.6, 3.5 Hz, 1H), 0.98 – 0.84 (m, 1H).

^{13}C-NMR (100 MHz, $CDCl_3$): δ / ppm = 141.8, 138.0, 129.5 (2C), 127.8 (2C), 74.8, 47.2, 38.8, 35.9, 30.2, 28.6, 25.6, 25.0, 15.8.

IR (Diamond-ATR, neat): $\tilde{\nu}$ / cm^{-1} = 3350, 2963, 2926, 2855, 2360, 2342, 1740, 1735, 1456, 1448, 1031.

MS (EI, 70 eV): *m/z* (%) = 201 (11), 200 (71), 172 (14), 171 (100), 143 (20), 132 (34), 129 (54), 128 (12), 120 (27), 119 (85), 117 (58), 115 (22), 105 (61), 91 (67), 71 (11), 79 (18).

HRMS (EI-orbitrap): *m/z:* [M] calc. for [$C_{15}H_{22}O$]: 218.1671; found 218.1664.

m.p. (°C): 63.6 – 65.5.

4-(1-phenylethyl)heptan-4-ol (10ab)

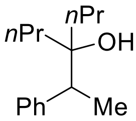

According to **TP3**, a solution of 3-(chloromethyl)heptane (**2**, 0.2 M, 2.0 equiv) in *n*-hexane was prepared. The solution of **2** was pumped through the activated sodium packed-bed reactor (see **TP1**) by pump A (flow rate: 2.0 mL/min) into the precooling loop (V_{pre2} = 0.35 mL) at 25 °C. Subsequently upon reaching the steady state, it was injected (2 min) into a flask charged with TMEDA (0.80 mmol, 2.0 equiv) in ethylbenzene (**3g**, 1.0 mL) and the resulting mixture was stirred at 25 °C for 30 min. The reaction flask was cooled to –20 °C and a solution of heptan-4-one (**9p**, 46 mg, 0.40 mmol, 1.0 equiv) in THF (1.0 mL) was added The reaction mixture was stirred at –20 °C for 10 min followed by another 30 min at 25 °C, before sat. *aq.* NH_4Cl solution was added for quenching the reaction mixture. The aqueous layer was extracted three times with EtOAc (3×30 mL) and the combined organic layers were dried over anhydrous $MgSO_4$ and filtrated. After removal of the solvent, flash column chromatographical purification (silica gel, pentane:EtOAc = 98:2 → 95:5) afforded the title compound **10ab** as a colorless oil (54 mg, 0.25 mmol, 61% yield).

^{1}H-NMR (400 MHz, $CDCl_3$): δ / ppm = 7.37 – 7.30 (m, 2H), 7.30 – 7.21 (m, 3H), 2.88 (q, *J* = 7.2 Hz, 1H), 1.61 – 1.47 (m, 2H), 1.39 – 1.26 (m, 8H), 1.25 – 1.15 (m, 1H), 1.10 (s, 1H), 0.96 (t, *J* = 7.2 Hz, 3H), 0.85 (t, *J* = 7.0 Hz, 3H).

^{13}C-NMR (100 MHz, $CDCl_3$): δ / ppm = 143.8, 129.3 (2C), 128.2 (2C), 126.5, 75.8, 46.3, 39.9, 38.1, 17.1, 16.8, 15.5, 14.9, 14.7.

IR (Diamond-ATR, neat): $\tilde{\nu}$ / cm^{-1} = 3478, 3027, 2957, 2934, 2873, 1494, 1453, 1378, 1343, 1287, 1132, 1097, 1008, 997, 981, 952, 908, 846, 775, 723, 702.

MS (EI, 70 eV): *m/z* (%) = 177 (30), 117 (14), 115 (100), 106 (18), 105 (23), 97 (15), 91 (46), 79 (11), 77 (11), 73 (16), 55 (24).

HRMS (EI-orbitrap): *m/z:* [M – H_2O] calc. for [$C_{15}H_{22}$]: 202.1722; found 202.1715.

1,1-dicyclopropyl-2-phenylpropan-1-ol (10ac)

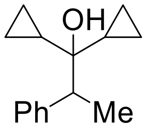

According to **TP3**, a solution of 3-(chloromethyl)heptane (**2**, 0.2 M, 2.0 equiv) in *n*-hexane was prepared. The solution of **2** was pumped through the activated sodium packed-bed reactor (see **TP1**) by pump A (flow rate: 2.0 mL/min) into the precooling loop (V_{pre2} = 0.35 mL) at 25 °C. Subsequently upon reaching the steady state, it was injected (2 min) into a flask charged with TMEDA (0.80 mmol, 2.0 equiv) in ethylbenzene (**3g**, 1.0 mL) and the resulting mixture was stirred at 25 °C for 30 min. The reaction flask was cooled to –20 °C and a solution of dicyclopropylmethanone (**9j**, 44 mg, 0.40 mmol, 1.0 equiv) in THF (1.0 mL) was added The reaction mixture was stirred at –20 °C for 10 min followed by another 30 min at 25 °C, before sat. *aq.* NH_4Cl solution was added for quenching the reaction mixture. The aqueous layer was extracted three times with EtOAc (3×30 mL) and the combined organic layers were dried over anhydrous $MgSO_4$ and filtrated. After removal of the solvent, flash column chromatographical purification (silica gel, pentane:EtOAc = 98:2) afforded the title compound **10ac** as a colorless oil (48 mg, 0.22 mmol, 55% yield).

^{1}H-NMR (400 MHz, $CDCl_3$): δ / ppm = 7.38 – 7.32 (m, 2H), 7.31 – 7.26 (m, 2H), 7.25 – 7.19 (m, 1H), 3.01 (q, *J* = 7.3 Hz, 1H), 1.47 (d, *J* = 7.3 Hz, 3H), 0.95 – 0.86 (m, 1H), 0.85 (s, 1H), 0.73 – 0.64 (m, 1H), 0.41 – 0.16 (m, 8H).

^{13}C-NMR (100 MHz, $CDCl_3$): δ / ppm = 143.5, 129.6 (2C), 127.8 (2C), 126.4, 72.3, 51.2, 17.2, 16.4, 15.9, 1.7, 1.1, -0.4, -0.4.

IR (Diamond-ATR, neat): $\tilde{\nu}$ / cm^{-1} = 3585, 3085, 3025, 3007, 2936, 1494, 1452, 1373, 1288, 1181, 1021, 990, 969, 928, 913, 764, 702.

MS (EI, 70 eV): *m/z* (%) = 183 (12), 155 (17), 153 (11), 142 (10), 141 (29), 129 (19), 128 (20), 115 (18), 111 (100), 105 (35), 103 (14), 91 (27), 79 (20), 77 (21), 69 (98).

HRMS (EI-orbitrap): *m/z:* [M – H_2O] calc. for [$C_{15}H_{18}$]: 198.1409; found 198.1402.

trans-(1-phenylethyl)cyclohexan-1-ol (10ad) mixture of diastereoisomeres

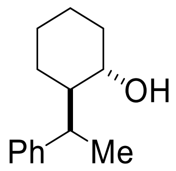

According to **TP3**, a solution of 3-(chloromethyl)heptane (**2**, 0.2 M, 2.0 equiv) in *n*-hexane was prepared. The solution of **2** was pumped through the activated sodium packed-bed reactor (see **TP1**) by pump A (flow rate: 2.0 mL/min) into the precooling loop (V_{pre2} = 0.35 mL) at 25 °C. Subsequently upon reaching the steady state, it was injected (2 min) into a flask charged with TMEDA (0.80 mmol, 2.0 equiv) in ethylbenzene (**3g**, 1.0 mL) and the resulting mixture was stirred at 25 °C for 30 min. The reaction flask was cooled to –20 °C and a solution of cyclohexene oxide (**9a**, 39 mg, 0.40 mmol, 1.0 equiv) in THF (1.0 mL) was added The reaction mixture was stirred at –20 °C for 10 min followed by another 30 min at 25 °C, before sat. *aq.* NH_4Cl solution was added for quenching the reaction mixture. The aqueous layer was extracted three times with EtOAc (3×30 mL) and the combined organic layers were dried over anhydrous $MgSO_4$ and filtrated. After removal of the solvent, flash column chromatographical purification (silica gel, pentane:EtOAc = 90:10 → 80:20) afforded the title compound **10ad** as a colorless oil (58 mg, 0.28 mmol, 71% yield, d.r. = 2:1).

Major:

^{1}H-NMR (400 MHz, $CDCl_3$): δ / ppm = 7.30 – 7.20 (m, 4H), 7.19 – 7.14 (m, 1H), 3.48 – 3.41 (m, 1H), 3.12 – 3.04 (m, 1H), 1.91 – 1.84 (m, 1H), 1.83 – 1.76 (m, 1H), 1.63 – 1.36 (m, 4H) 1.31 (d, *J* = 7.4 Hz, 3H), 1.27 – 1.19 (m, 1H), 1.15 – 0.97 (m, 2H), 0.74 – 0.63 (m, 1H)

^{13}C-NMR (100 MHz, $CDCl_3$): δ / ppm = 143.7, 128.9 (2C), 127.8 (2C), 126.0, 71.6, 49.3, 37.8, 36.3, 25.7, 25.0, 24.7, 18.5.

Minor:

^{1}H-NMR (400 MHz, $CDCl_3$): δ / ppm = 7.30 – 7.20 (m, 4H), 7.19 – 7.14 (m, 1H), 3.48 – 3.41 (m, 1H), 3.35 – 3.28 (m, 1H), 2.00 – 1.93 (m, 1H), 1.70 – 1.65 (m, 1H), 1.63 – 1.36 (m, 4H), 1.27 – 1.19 (m, 5H), 1.15 – 0.97 (m, 2H),

^{13}C-NMR (100 MHz, $CDCl_3$): δ / ppm = 146.5, 128.2 (2C), 128.0 (2C), 125.9, 72.3, 51.5, 38.3, 35.5, 25.5, 25.0, 24.6, 13.8.

Mixture:

IR (Diamond-ATR, neat): $\tilde{\nu}$ / cm^{-1} = 3343, 3028, 2962, 2929, 2856, 1494, 1451, 1378, 1053, 1024, 975, 843, 769, 748, 702.

MS (EI, 70 eV): *m/z* (%) = 186 (54), 171 (28), 157 (14), 144 (22), 143 (26), 129 (33), 128 (13), 118 (29), 117 (26), 115 (25), 106 (49), 105 (100), 104 (10), 103 (19), 91 (96), 81 (26), 79 (39), 78 (11), 77 (22).

HRMS (EI-orbitrap): *m/z:* [M – H_2O] calc. for [$C_{14}H_{18}$]: 186.1409; found 186.1401.

(2*S*)-1-(benzyloxy)-4-phenylpentan-2-ol (10ae) mixture of diastereoisomeres

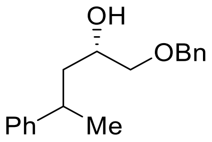

According to **TP3**, a solution of 3-(chloromethyl)heptane (**2**, 0.2 M, 2.0 equiv) in *n*-hexane was prepared. The solution of **2** was pumped through the activated sodium packed-bed reactor (see **TP1**) by pump A (flow rate: 2.0 mL/min) into the precooling loop (V_{pre2} = 0.35 mL) at 25 °C. Subsequently upon reaching the steady state, it was injected (2 min) into a flask charged with TMEDA (0.80 mmol, 2.0 equiv) in ethylbenzene (**3g**, 1.0 mL) and the resulting mixture was stirred at 25 °C for 30 min. The reaction flask was cooled to –20 °C and a solution of (S)-2-((benzyloxy)methyl)oxirane (**9m**, 66 mg, 0.40 mmol, 1.0 equiv) in THF (1.0 mL) was added The reaction mixture was stirred at –20 °C for 10 min followed by another 30 min at 25 °C, before sat. *aq.* NH_4Cl solution was added for quenching the reaction mixture. The aqueous layer was extracted three times with EtOAc (3×30 mL) and the combined organic layers were dried over anhydrous $MgSO_4$ and filtrated. After removal of the solvent, flash column chromatographical purification (silica gel, pentane:EtOAc = 90:10 → 80:20) afforded the title compound **10ae** as a colorless oil (66 mg, 0.24 mmol, 61% yield d.r. = 1.15:1).

Major:

^{1}H-NMR (400 MHz, CDCl$_3$): δ / ppm = 7.40 – 7.28 (m, 7H), 7.25 – 7.18 (m, 3H), 4.49 (s, 2H) 3.60 – 3.54 (m, 1H), 3.40 – 3.31 (m, 1H), 3.30 – 3.24 (m, 1H), 3.12 – 3.00 (m, 1H), 2.41 – 2.27 (m, 1H), 1.89 – 1.56 (m, 2H), 1. 30 (d, *J* = 7.0 Hz, 3H)

Minor:

^{1}H-NMR (400 MHz, CDCl$_3$): δ / ppm = 7.40 – 7.28 (m, 7H), 7.25 – 7.18 (m, 3H), 4.55 (s, 2H), 3.90 – 3.80 (m, 1H), 3.52 (dd, *J* = 9.5, 3.0 Hz, 1H), 3.40 – 3.31 (m, 1H), 2.99 – 2.88 (m, 1H), 2.41 – 2.27 (m, 1H), 1.89 – 1.56 (m, 2H), 1.29 (d, *J* = 6.9 Hz, 3H).

Mixture:

^{13}C-NMR (100 MHz, CDCl$_3$): δ / ppm = 147.5, 146.6, 138.0, 138.0, 128.6 (2C), 128.6 (2C), 128.5 (2C), 128.5 (2C), 127.9, 127.9 (2C), 127.9, 127.8 (2C), 127.3 (2C), 127.0 (2C), 126.2, 126.2, 75.1, 74.6, 73.4, 73.4, 68.5, 68.5, 41.6, 41.6, 36.2, 36.0, 23.3, 21.8.

IR (Diamond-ATR, neat): $\tilde{\nu}$ / cm^{-1} = 3441, 3028, 2927, 2866, 1494, 1453, 1365, 1308, 1205, 1087, 1076, 1028, 1012, 952, 907, 850, 763, 737, 698.

MS (EI, 70 eV): *m/z* (%) = 119 (70), 106 (20), 105 (53), 105 (53), 105 (85), 105 (92), 105 (49), 105 (49), 92 (18), 91 (100), 79 (12), 77 (12).

HRMS (EI-orbitrap): *m/z:* [M] calc. for [$C_{18}H_{22}O_2$]: 270.1620; found 270.1614.

dicyclopropyl(1,2,3,4-tetrahydronaphthalen-1-yl)methanol (10af)

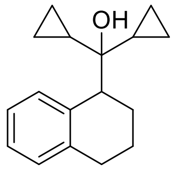

According to **TP4**, a solution of 3-(chloromethyl)heptane (**2**, 0.2 M, 2.0 equiv) in *n*-hexane was prepared. The solution of **2** was pumped through the activated sodium packed-bed reactor (see **TP1**) by pump A (flow rate: 2.0 mL/min) into the precooling loop (V_{pre2} = 0.35 mL) at 25 °C. Subsequently upon reaching the steady state, it was injected for 2 min into a flask charged with a solution of TMEDA (0.80 mmol, 2.0 equiv) and tetralin (**3h**, 53 mg, 0.40 mmol, 1.0 equiv) in hexane (2.0 mL) the mixture was stirred for 30 min at 25 °C. Before it was cooled to –20 °C and a solution of dicyclopropylmethanone (**9j**, 88 mg, 0.80 mmol, 2.0 equiv) in THF (2.0 mL) was added. The reaction mixture was stirred at –20 °C for 10 min followed by another 30 min at 25 °C, before sat. *aq.* NH_4Cl solution was added for quenching the reaction mixture. The aqueous layer was extracted three times with EtOAc (3×30 mL) and the combined organic layers were dried over anhydrous $MgSO_4$ and filtrated. After removal of the solvent, flash column chromatographical purification (silica gel, pentane:EtOAc = 98:2) afforded the title compound **10af** as a colorless oil (50 mg, 0.21 mmol, 52% yield).

^{1}H-NMR (400 MHz, $CDCl_3$): δ / ppm = 7.61 – 7.50 (m, 1H), 7.12 (dd, *J* = 9.3, 7.0 Hz, 3H), 3.16 (dd, *J* = 7.5, 5.7 Hz, 1H), 2.83 – 2.65 (m, 2H), 2.16 – 1.98 (m, 3H), 1.62 – 1.49 (m, 1H), 1.02 (s, 1H), 0.88 (tt, *J* = 8.3, 5.6 Hz, 1H), 0.75 (tt, *J* = 8.5, 5.6 Hz, 1H), 0.60 – 0.50 (m, 1H), 0.50 – 0.33 (m, 4H), 0.32 – 0.19 (m, 2H), 0.17 – 0.05 (m, 1H).

^{13}C-NMR (100 MHz, $CDCl_3$): δ / ppm = 140.4, 137.4, 131.6, 128.7, 125.8, 125.0, 74.2, 48.6, 30.5, 25.2, 22.1, 17.4, 16.2, 3.3, 1.8, -0.2, -1.3.

IR (Diamond-ATR, neat): $\tilde{\nu}$ / cm^{-1} = 3083, 3009, 2931, 2891, 2862, 1489, 1450, 1436, 1427, 1377, 1308, 1286, 1263, 1208, 1178, 1160, 1122, 1080, 1020, 986, 939, 926, 913, 890, 831, 762, 745.

MS (EI, 70 eV): *m/z* (%) = 131 (24), 129 (20), 128 (17), 115 (21), 111 (100), 91 (25), 69 (76).

HRMS (EI-orbitrap): *m/z:* [M] calc. for [$C_{17}H_{22}O$]: 242.1671; found 242.1664.

4-(1,2,3,4-tetrahydronaphthalen-1-yl)heptan-4-ol (10ag)

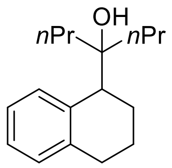

According to **TP4**, a solution of 3-(chloromethyl)heptane (**2**, 0.2 M, 2.0 equiv) in *n*-hexane was prepared. The solution of **2** was pumped through the activated sodium packed-bed reactor (see **TP1**) by pump A (flow rate: 2.0 mL/min) into the precooling loop (V_{pre2} = 0.35 mL) at 25 °C. Subsequently upon reaching the steady state, it was injected for 2 min into a flask charged with a solution of TMEDA (0.80 mmol, 2.0 equiv) and tetralin (**3h**, 53 mg, 0.40 mmol, 1.0 equiv) in hexane (2.0 mL) the mixture was stirred for 30 min at 25 °C. Before it was cooled to –20 °C and a solution of heptan-4-one (**9p**, 92 mg, 0.80 mmol, 2.0 equiv) in THF (2.0 mL) was added. The reaction mixture was stirred at –20 °C for 10 min followed by another 30 min at 25 °C, before sat. *aq.* NH_4Cl solution was added for quenching the reaction mixture. The aqueous layer was extracted three times with EtOAc (3×30 mL) and the combined organic layers were dried over anhydrous $MgSO_4$ and filtrated. After removal of the solvent, flash column chromatographical purification (silica gel, pentane:EtOAc = 100:0 → 98:2) afforded the title compound **10ag** as a colorless oil (53 mg, 0.22 mmol, 54% yield).

^{1}H-NMR (400 MHz, $CDCl_3$): δ / ppm = 7.34 – 7.27 (m, 1H), 7.18 – 7.07 (m, 3H), 3.05 – 2.96 (m, 1H), 2.83 – 2.66 (m, 2H), 2.21 – 2.07 (m, 1H), 1.93 – 1.76 (m, 2H), 1.61 – 1.25 (m, 9H), 1.10 (s, 1H), 0.95 (t, *J* = 7.0 Hz, 3H), 0.90 (t, *J* = 7.0 Hz, 3H).

^{13}C-NMR (100 MHz, $CDCl_3$): δ / ppm = 140.5, 137.0, 131.2, 129.2, 126.1, 125.0, 77.5, 44.4, 40.3, 37.8, 29.7, 25.0, 21.2, 16.9, 16.8, 14.9, 14.8.

IR (Diamond-ATR, neat): $\tilde{\nu}$ / cm^{-1} = 2956, 2931, 2871, 1490, 1455, 1378, 1294, 1152, 1136, 994, 976, 740.

MS (EI, 70 eV): *m/z* (%) = 199 (12), 185 (18), 157 (38), 141 (14), 132 (33), 131 (43), 130 (19), 130 (11), 129 (100), 128 (53), 117 (12), 115 (18), 115 (31), 104 (36), 91 (30), 55 (13).

HRMS (EI-orbitrap): *m/z:* [M – H_2O] calc. for [$C_{17}H_{24}$]: 228.1878; found 228.1871.

trans-2-(1,2,3,4-tetrahydronaphthalen-1-yl)cyclohexan-1-ol (10ah) mixture of diastereoisomers

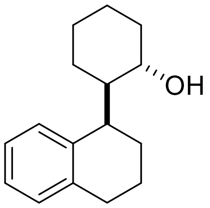

According to **TP4**, a solution of 3-(chloromethyl)heptane (**2**, 0.2 M, 2.0 equiv) in *n*-hexane was prepared. The solution of **2** was pumped through the activated sodium packed-bed reactor (see **TP1**) by pump A (flow rate: 2.0 mL/min) into the precooling loop (V_{pre2} = 0.35 mL) at 25 °C. Subsequently upon reaching the steady state, it was injected for 2 min into a flask charged with a solution of TMEDA (0.80 mmol, 2.0 equiv) and tetralin (**3h**, 53 mg, 0.40 mmol, 1.0 equiv) in hexane (2.0 mL) the mixture was stirred for 30 min at 25 °C. Before it was cooled to –20 °C and a solution of cyclohexene oxide (**9a**, 79 mg, 0.80 mmol, 2.0 equiv) in THF (2.0 mL) was added. The reaction mixture was stirred at –20 °C for 10 min followed by another 30 min at 25 °C, before sat. *aq.* NH_4Cl solution was added for quenching the reaction mixture. The aqueous layer was extracted three times with EtOAc (3×30 mL) and the combined organic layers were dried over anhydrous $MgSO_4$ and filtrated. After removal of the solvent, flash column chromatographical purification (silica gel, pentane:EtOAc = 95:5 → 85:15) afforded the title compound **10ah** as a colorless oil (52 mg, 0.33 mmol, 56% yield, d.r. = 1.2:1).

Major:

^{1}H-NMR (400 MHz, $CDCl_3$): δ / ppm = 7.31 (d, *J* = 7.6 Hz, 1H), 7.17 – 7.04 (m, 3H), 3.72 – 3.56 (m, 1H), 3.13 – 3.03 (m, 1H), 2.86 – 2.64 (m, 2H), 2.03 – 1.84 (m, 4H), 1.84 – 1.74 (m, 1H), 1.73 – 1.57 (m, 4H), 1.29 – 1.18 (m, 4H), 1.16 – 1.04 (m, 1H).

^{13}C-NMR (100 MHz, $CDCl_3$): δ / ppm = 139.6, 138.2, 129.5, 128.9, 125.8, 125.7, 73.1, 51.0, 39.8, 36.3, 30.4, 30.0, 26.5, 26.3, 24.9, 22.2.

IR (Diamond-ATR, neat): $\tilde{\nu}$ / cm^{-1} = 3371, 2926, 2856, 1491, 1449, 1052, 1037, 1013, 767, 740.

MS (EI, 70 eV): *m/z* (%) = 212 (22), 132 (18), 131 (100), 130 (33), 129 (22), 128 (14), 91 (16), 57 (13).

HRMS (EI-orbitrap): *m/z:* [M] calc. for [$C_{16}H_{22}O$]: 230.1671; found 230.1674.

Minor:

^{1}H-NMR (400 MHz, $CDCl_3$): δ / ppm = 7.26 (d, *J* = 7.7 Hz, 1H), 7.17 – 7.03 (m, 3H), 3.61 (td, *J* = 10.1, 4.2 Hz, 1H), 3.47 – 3.38 (m, 1H), 2.70 (dd, *J* = 7.7, 4.5 Hz, 2H), 2.11 – 2.03 (m, 1H), 2.01 – 1.89 (m, 4H), 1.73 – 1.57 (m, 4H), 1.29 – 1.18 (m, 4H), 1.07 – 0.93 (m, 1H).

^{13}C-NMR (100 MHz, $CDCl_3$): δ / ppm = 139.6, 139.0, 129.1, 127.2, 125.9, 125.1, 71.6, 50.1, 36.7, 36.7, 30.6, 26.1, 25.4, 25.1, 23.7, 22.6.

IR (Diamond-ATR, neat): $\tilde{\nu}$ / cm^{-1} = 3355, 2928, 2856, 1492, 1450, 1062, 1034, 762, 739.

MS (EI, 70 eV): *m/z* (%) = 213 (18), 212 (91), 207 (23), 184 (20), 183 (11), 170 (10), 169 (19), 165 (10), 155 (12), 145 (10), 144 (49), 143 (16), 142 (15), 141 (25), 132 (51), 130 (100), 129 (74), 128 (74), 128 (57), 127 (14), 117 (17), 116 (27), 115 (34), 103 (21), 97 (15), 91 (63), 83 (14), 77 (14), 71 (17), 67 (11), 57 (30), 55 (21), 44 (67), 43 (23), 43 (43), 41 (32).

HRMS (EI-orbitrap): *m/z:* [M] calc. for [$C_{16}H_{22}O$]: 230.1671; found 230.1669.

trans-2-(2-methoxy-3-methylbenzyl)cyclohexan-1-ol (10ai)

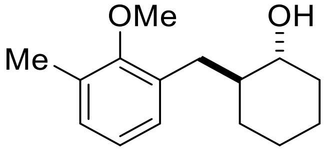

According to **TP4**, a solution of 3-(chloromethyl)heptane (**2**, 0.2 M, 2.0 equiv) in *n*-hexane was prepared. The solution of **2** was pumped through the activated sodium packed-bed reactor (see **TP1**) by pump A (flow rate: 2.0 mL/min) into the precooling loop (V_{pre2} = 0.35 mL) at 25 °C. Subsequently upon reaching the steady state, it was injected for 2 min into a flask charged with a solution of TMEDA (0.80 mmol, 2.0 equiv) and 2-methoxy-1,3-dimethylbenzene (**3i**, 54 mg, 0.40 mmol, 1.0 equiv) in hexane (2.0 mL) the mixture was stirred for 30 min at 25 °C. Before it was cooled to –20 °C and a solution of cyclohexene oxide (**9a**, 79 mg, 0.80 mmol, 2.0 equiv) in THF (2.0 mL) was added. The reaction mixture was stirred at –20 °C for 10 min followed by another 30 min at 25 °C, before sat. *aq.* NH_4Cl solution was added for quenching the reaction mixture. The aqueous layer was extracted three times with EtOAc (3×30 mL) and the combined organic layers were dried over anhydrous $MgSO_4$ and filtrated. After removal of the solvent, flash column chromatographical purification (silica gel, pentane:EtOAc = 95:5 → 80:20) afforded the title compound **10ai** as a colorless oil (62 mg, 0.26 mmol, 66% yield).

^{1}H-NMR (400 MHz, $CDCl_3$): δ / ppm = 7.05 (d, *J* = 6.9 Hz, 1H), 7.01 – 6.92 (m, 2H), 3.75 (s, 3H), 3.18 (s, 1H), 3.13 – 3.03 (m, 1H), 2.86 – 2.72 (m, 2H), 2.31 (s, 3H), 1.95 (d, *J* = 12.7 Hz, 1H), 1.73 – 1.62 (m, 2H), 1.62 – 1.48 (m, 2H), 1.34 – 1.19 (m, 1H), 1.18 – 0.94 (m, 3H).

^{13}C-NMR (100 MHz, $CDCl_3$): δ / ppm = 156.9, 132.6, 130.9, 129.6, 129.5, 124.0, 73.4, 60.6, 46.7, 35.0, 32.7, 30.5, 25.8, 25.1, 16.4.

IR (Diamond-ATR, neat): $\tilde{\nu}$ / cm^{-1} = 3414, 2925, 2855, 1469, 1448, 1422, 1259, 1213, 1167, 1086, 1068, 1059, 1031, 1013, 810, 767.

MS (EI, 70 eV): *m/z* (%) = 216 (20), 201 (13), 185 (10), 137 (10), 136 (100), 133 (15), 122 (14), 121 (79), 115 (10), 105 (88), 103 (10), 91 (25), 79 (18).

HRMS (EI-orbitrap): *m/z:* [M] calc. for [$C_{15}H_{22}O_2$]: 234.1620; found: 234.1612.

2-(2-fluoro-3,5-dimethylbenzyl)adamantan-2-o (10aj)

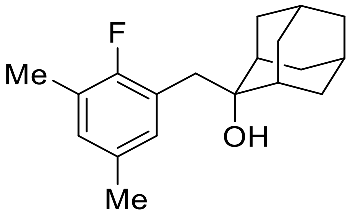

According to **TP4**, a solution of 3-(chloromethyl)heptane (**2**, 0.2 M, 2.0 equiv) in *n*-hexane was prepared. The solution of **2** was pumped through the activated sodium packed-bed reactor (see **TP1**) by pump A (flow rate: 2.0 mL/min) into the precooling loop (V_{pre2} = 0.35 mL) at 25 °C. Subsequently upon reaching the steady state, it was injected for 2 min into a flask charged with a solution of TMEDA (0.80 mmol, 2.0 equiv) and 2-fluoro-1,3,5-trimethylbenzene (**3j**, 55 mg, 0.40 mmol, 1.0 equiv) in hexane (2.0 mL) the mixture was stirred for 30 min at –40 °C. Before a solution of adamantanone (**9k**, 120 mg, 0.80 mmol, 2.0 equiv) in THF (2.0 mL) was added. The reaction mixture was stirred at –40 °C for 10 min followed by another 30 min at 25 °C, before sat. *aq.* NH_4Cl solution was added for quenching the reaction mixture. The aqueous layer was extracted three times with EtOAc (3×30 mL) and the combined organic layers were dried over anhydrous $MgSO_4$ and filtrated. After removal of the solvent, flash column chromatographical purification (silica gel, pentane:EtOAc = 100:0 → 95:5) afforded the title compound **10aj** as a colorless solid (76 mg, 0.26 mmol, 66% yield).

^{1}H-NMR (400 MHz, $CDCl_3$): δ / ppm = 6.91 – 6.81 (m, 2H), 3.02 – 2.94 (m, 2H), 2.25 (s, 3H), 2.24 – 2.15 (m, 5H), 2.16 – 2.07 (m, 2H), 1.95 – 1.85 (m, 1H), 1.84 – 1.74 (m, 3H), 1.74 – 1.65 (m, 4H), 1.56 – 1.47 (m, 3H).

^{13}C-NMR (100 MHz, $CDCl_3$): δ / ppm = 158.65 (d, *J* = 240.8 Hz), 132.61 (d, *J* = 4.1 Hz), 130.86 (d, *J* = 4.7 Hz), 130.52 (d, *J* = 4.8 Hz), 124.46 (d, *J* = 19.2 Hz), 123.55 (d, *J* = 17.2 Hz), 75.1, 38.5, 37.5 (2C), 37.5, 34.7, 34.7, 33.2 (2C), 27.6, 27.5, 20.7, 14.9 (d, *J* = 4.3 Hz).

IR (Diamond-ATR, neat): $\tilde{\nu}$ / cm^{-1} = 2903, 2857, 1484, 1474, 1456, 1442, 1215, 1202, 1141, 1124, 1100, 1062, 1043, 1022, 1008, 993, 928, 859, 756.

MS (EI, 70 eV): *m/z* (%) = 270 (31), 175 (17), 152 (10), 151 (98), 138 (100), 137 (41), 133 (16), 123 (33), 115 (15), 105 (14), 91 (49), 79 (18).

HRMS (EI-orbitrap): *m/z:* [M – H] calc. for [$C_{19}H_{24}FO$]: 287.1811; found 287.1803.

m.p. (°C): 66.3 – 68.7.

***trans*-2-((2-hydroxycyclohexyl)methyl)-N,N-diisopropylbenzamide (10ak) mixture of diastereoisomers**

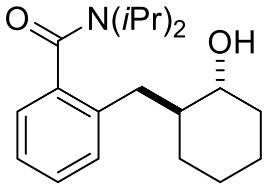

According to **TP4**, a solution of 3-(chloromethyl)heptane (**2**, 0.2 M, 2.0 equiv) in *n*-hexane was prepared. The solution of **2** was pumped through the activated sodium packed-bed reactor (see **TP1**) by pump A (flow rate: 2.0 mL/min) into the precooling loop (V_{pre2} = 0.35 mL) at 25 °C. Subsequently upon reaching the steady state, it was injected for 2 min into a flask charged with a solution of TMEDA (0.80 mmol, 2.0 equiv) and *N,N*-diisopropyl-2-methylbenzamide (**3k**, 88 mg, 0.40 mmol, 1.0 equiv) in hexane (2.0 mL) the mixture was stirred for 30 min at –40 °C. Before a solution of cyclohexene oxide (**9a**, 79 mg, 0.80 mmol, 2.0 equiv) in THF (2.0 mL) was added. The reaction mixture was stirred at –40 °C for 10 min followed by another 30 min at 25 °C, before sat. *aq.* NH_4Cl solution was added for quenching the reaction mixture. The aqueous layer was extracted three times with EtOAc (3×30 mL) and the combined organic layers were dried over anhydrous $MgSO_4$ and filtrated. After removal of the solvent, flash column chromatographical purification (silica gel, pentane:EtOAc = 95:5 → 80:20) afforded the title compound **10ak** as a colorless solid (109 mg, 0.34 mmol, 86% yield, d.r. 2.6:1).

Major:

^{1}H-NMR (400 MHz, $CDCl_3$): δ / ppm = 7.36 – 7.33 (m, 2H), 7.26 – 7.20 (m, 1H), 7.14 – 7.08 (m, 1H), 4.38 (d, *J* = 5.6 Hz, 1H), 3.82 (hept, *J* = 6.7 Hz, 1H), 3.67 – 3.53 (m, 1H), 3.41 – 3.27 (m, 1H), 2.86 – 2.77 (m, 1H), 2.47 (dd, *J* = 13.6, 4.6 Hz, 1H), 2.07 – 1.86 (m, 2H), 1.84 – 1.70 (m, 3H), 1.68 (d, *J* = 6.8 Hz, 3H), 1.64 (d, *J* = 6.8 Hz, 3H), 1.38 – 1.23 (m, 3H), 1.19 (t, *J* = 6.6 Hz, 6H), 1.14 (d, *J* = 6.7 Hz, 1H).

^{13}C-NMR (100 MHz, $CDCl_3$): δ / ppm = 172.3, 139.0, 136.5, 130.4, 128.6, 125.7, 124.7, 75.9, 51.2, 49.0, 46.1, 38.8, 35.4, 33.1, 26.0, 25.3, 21.1, 20.7, 20.5 (2C).

Minor:

^{1}H-NMR (400 MHz, $CDCl_3$): δ / ppm = 7.39 – 7.33 (m, 2H), 7.31 – 7.27 (m, 1H), 7.16 (dt, *J* = 7.4, 1.2 Hz, 1H), 3.73 (d, *J* = 4.9 Hz, 1H), 3.67 – 3.53 (m, 1H), 3.25 – 3.15 (m, 1H), 3.00 (dd, *J* = 13.7, 3.8 Hz, 1H), 2.76 (dd, *J* = 13.7, 6.9 Hz, 1H), 2.07 – 1.86 (m, 2H), 1.84 – 1.70 (m, 3H), 1.68 (d, *J* = 6.8 Hz, 3H), 1.64 (d, *J* = 6.8 Hz, 3H), 1.38 – 1.23 (m, 3H), 1.19 (t, *J* = 6.6 Hz, 6H), 1.14 (d, *J* = 6.7 Hz, 1H).

^{13}C-NMR (100 MHz, $CDCl_3$): δ / ppm = 172.0, 138.7, 136.0, 131.2, 127.9, 126.1, 125.0, 72.0, 51.1, 47.1, 46.1, 35.5, 34.8, 30.7, 26.1, 25.2, 20.9, 20.7, 20.5 (2C).

Mixture:

IR (Diamond-ATR, neat): $\tilde{\nu}$ / cm^{-1} = 3400, 2968, 2927, 2855, 1611, 1597, 1492, 1446, 1438, 1370, 1338, 1212, 1203, 1186, 1161, 1136, 1123, 1101, 1082, 1067, 1031, 920, 771, 745, 732.

MS (EI, 70 eV): *m/z* (%) = 298 (16), 288 (17), 274 (24), 218 (20), 217 (16), 204 (100), 199 (36), 198 (27), 189 (20), 181 (78), 179 (22), 176 (25), 171 (39), 170 (23), 169 (23), 166 (26), 165 (18), 162 (18), 157 (28), 145 (39), 143 (20), 141 (30),135 (24), 131 (46), 129 (76), 128 (26), 119 (82), 117 (40), 116 (23), 115 (47), 103 (18), 91 (52), 86 (74), 79 (15), 77 (16).

HRMS (EI-orbitrap): *m/z:* [M – H] calc. for [$C_{20}H_{30}NO_2$]: 316.2277; found 316.2271.

m.p. (°C): 121.7 – 125.0.

2-((2-hydroxyadamantan-2-yl)methyl)-N,N-diisopropylbenzamide (10al)

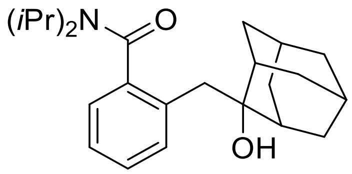

According to **TP4**, a solution of 3-(chloromethyl)heptane (**2**, 0.2 M, 2.0 equiv) in *n*-hexane was prepared. The solution of **2** was pumped through the activated sodium packed-bed reactor (see **TP1**) by pump A (flow rate: 2.0 mL/min) into the precooling loop (V_{pre2} = 0.35 mL) at 25 °C. Subsequently upon reaching the steady state, it was injected for 2 min into a flask charged with a solution of TMEDA (0.80 mmol, 2.0 equiv) *N,N*-diisopropyl-2-methylbenzamide (**3k**, 88 mg, 0.40 mmol, 1.0 equiv) in hexane (2.0 mL) the mixture was stirred for 30 min at –40 °C. Before a solution of adamantanone (**9k**, 120 mg, 0.80 mmol, 2.0 equiv) in THF (2.0 mL) was added. The reaction mixture was stirred at –40 °C for 10 min followed by another 30 min at 25 °C, before sat. *aq.* NH_4Cl solution was added for quenching the reaction mixture. The aqueous layer was extracted three times with EtOAc (3×30 mL) and the combined organic layers were dried over anhydrous $MgSO_4$ and filtrated. After removal of the solvent, flash column chromatographical purification (silica gel, pentane:EtOAc = 95:5 → 80:20) afforded the title compound **10al** as a colorless solid (120 mg, 0.32 mmol, 81% yield).

^{1}H-NMR (400 MHz, $CDCl_3$): δ / ppm = 7.32 – 7.26 (m, 1H), 7.23 – 7.14 (m, 2H), 7.11 (dd, *J* = 7.6, 1.5 Hz, 1H), 4.79 (s, 1H), 3.70 (hept, *J* = 6.7 Hz, 1H), 3.58 – 3.43 (m, 2H), 2.46 (d, *J* = 9.1 Hz, 1H), 2.35 (d, *J* = 13.8 Hz, 1H), 2.20 (d, *J* = 12.0 Hz, 2H), 1.96 – 1.85 (m, 3H), 1.85 – 1.68 (m, 5H), 1.62 – 1.52 (m, 7H), 1.51 – 1.42 (m, 2H), 1.13 (d, *J* = 6.7 Hz, 3H), 1.06 (d, *J* = 6.7 Hz, 3H).

^{13}C-NMR (100 MHz, $CDCl_3$): δ / ppm = 172.6, 138.1, 136.0, 130.9, 128.4, 126.0, 124.9, 73.4, 51.2, 46.2, 42.1, 40.7, 38.7, 35.0, 34.6, 34.3, 33.8, 32.7, 27.8, 27.6, 21.3, 20.7, 20.5, 20.5.

IR (Diamond-ATR, neat): $\tilde{\nu}$ / cm^{-1} = 3325, 2964, 2904, 2855, 1721, 1610, 1596, 1492, 1472, 1453, 1441, 1379, 1371, 1344, 1322, 1307, 1213, 1203, 1161, 1128, 1100, 1057, 1040, 1031, 997, 776, 761, 735, 701.

MS (EI, 70 eV): *m/z* (%) = 350 (10), 308 (23), 252 (18), 251 (100), 233 (14), 223 (29), 195 (14), 181 (19), 167 (17), 165 (22), 157 (15), 152 (10), 141 (31), 129 (15), 128 (13), 115 (14), 91 (16), 86 (17).

HRMS (EI-orbitrap): *m/z:* [M – H_2O] calc. for [$C_{24}H_{35}NO$]: 351.2562; found 351.2556.

m.p. (°C): 138.5 – 143.5.

***trans*-2-(phenyl(phenylthio)methyl)cyclohexan-1-ol (10am) mixture of diastereoisomeres**

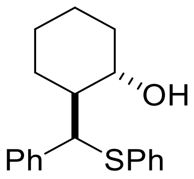

According to **TP4**, a solution of 3-(chloromethyl)heptane (**2**, 0.2 M, 2.0 equiv) in *n*-hexane was prepared. The solution of **2** was pumped through the activated sodium packed-bed reactor (see **TP1**) by pump A (flow rate: 2.0 mL/min) into the precooling loop (V_{pre2} = 0.35 mL) at 25 °C. Subsequently upon reaching the steady state, it was injected for 2 min into a flask charged with a solution of TMEDA (0.80 mmol, 2.0 equiv) and benzyl(phenyl)sulfane (**3l**, 80 mg, 0.40 mmol, 1.0 equiv) in hexane (2.0 mL) the mixture was stirred for 30 min at 25 °C. Before it was cooled to –20 °C and a solution of cyclohexene oxide (**9a**, 79 mg, 0.80 mmol, 2.0 equiv) in THF (2.0 mL) was added. The reaction mixture was stirred at –20 °C for 10 min followed by another 30 min at 25 °C, before sat. *aq.* NH_4Cl solution was added for quenching the reaction mixture. The aqueous layer was extracted three times with EtOAc (3×30 mL) and the combined organic layers were dried over anhydrous $MgSO_4$ and filtrated. After removal of the solvent, flash column chromatographical purification (silica gel, pentane:EtOAc = 95:5 → 90:10) afforded the title compound **10am** as a colorless oil (107 mg, 0.36 mmol, 90% yield, d.r. = 1.8:1).

Major:

^{1}H-NMR (400 MHz, $CDCl_3$): δ / ppm = 7.49 – 7.43 (m, 2H), 7.38 – 7.08 (m, 8H), 4.98 (d, *J* = 4.2 Hz, 1H), 3.16 (td, *J* = 10.2, 4.4 Hz, 1H), 2.13 – 1.90 (m, 3H), 1.88 – 1.61 (m, 3H), 1.39 – 0.87 (m, 4H).

^{13}C-NMR (100 MHz, $CDCl_3$): δ / ppm = 139.2, 135.5, 130.7 (2C), 129.6 (2C), 128.8 (2C), 128.0 (2C), 127.1, 126.5, 70.0, 53.0, 48.9, 36.3, 26.1, 25.5, 24.7.

Minor:

^{1}H-NMR (400 MHz, $CDCl_3$): δ / ppm = 7.56 – 7.52 (m, 2H), 7.38 – 7.08 (m, 8H), 4.95 (d, *J* = 3.2 Hz, 1H), 3.93 – 3.85 (m, 1H), 2.13 – 1.90 (m, 3H), 1.88 – 1.61 (m, 3H), 1.39 – 0.87 (m, 4H).

^{13}C-NMR (100 MHz, $CDCl_3$): δ / ppm = 141.6, 136.6, 129.8 (2C), 128.8 (2C), 128.7 (2C), 128.3 (2C), 126.9, 126.1, 73.0, 54.4, 52.9, 32.5, 25.9, 25.7, 24.8.

Mixture:

IR (Diamond-ATR, neat): $\tilde{\nu}$ / cm^{-1} = 3372, 2929, 2856, 1583, 1494, 1480, 1449, 1439, 1088, 1064, 1040, 1025, 852, 764, 750, 736, 702, 690.

MS (EI, 70 eV): *m/z* (%) = 199 (15), 197 (11), 190 (13), 189 (100), 188 (20), 172 (13), 171 (91), 143 (17), 129 (58), 117 (17), 115 (21), 110 (14), 97 (13), 91 (71), 84 (12).

HRMS (EI-orbitrap): *m/z:* [M] calc. for [$C_{19}H_{22}OS$]: 298.1391; found 298.1384.

4-phenyl-4-(phenylthio)butan-1-ol (10an)

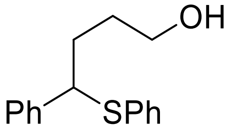

According to **TP4**, a solution of 3-(chloromethyl)heptane (**2**, 0.2 M, 2.0 equiv) in *n*-hexane was prepared. The solution of **2** was pumped through the activated sodium packed-bed reactor (see **TP1**) by pump A (flow rate: 2.0 mL/min) into the precooling loop (V_{pre2} = 0.35 mL) at 25 °C. Subsequently upon reaching the steady state, it was injected for 2 min into a flask charged with a solution of TMEDA (0.80 mmol, 2.0 equiv) and benzyl(phenyl)sulfane (**3l**, 80 mg, 0.40 mmol, 1.0 equiv) in hexane (2.0 mL) the mixture was stirred for 30 min at 25 °C. Before it was cooled to –20 °C and a solution of oxetane (**9h**, 46 mg, 0.80 mmol, 2.0 equiv) in THF (2.0 mL) was added. The reaction mixture was stirred at –20 °C for 10 min followed by another 30 min at 25 °C, before sat. *aq.* NH_4Cl solution was added for quenching the reaction mixture. The aqueous layer was extracted three times with EtOAc (3×30 mL) and the combined organic layers were dried over anhydrous $MgSO_4$ and filtrated. After removal of the solvent, flash column chromatographical purification (silica gel, pentane:EtOAc = 90:10 → 75:25) afforded the title compound **10an** as a colorless oil (65 mg, 0.25 mmol, 63% yield).

^{1}H-NMR (400 MHz, $CDCl_3$): δ / ppm = 7.33 – 7.18 (m, 10H), 4.24 – 4.12 (m, 1H), 3.62 (t, *J* = 6.5 Hz, 2H), 2.17 – 1.95 (m, 2H), 1.72 – 1.51 (m, 2H), 1.48 (s, 1H).

^{13}C-NMR (100 MHz, $CDCl_3$): δ / ppm =142.0, 134.9, 132.5 (2C), 128.8 (2C), 128.5 (2C), 127.9 (2C), 127.3, 127.2, 62.5, 53.5, 32.6, 30.8.

IR (Diamond-ATR, neat): $\tilde{\nu}$ / cm^{-1} = 3341, 3059, 3028, 2942, 2868, 1583, 1493, 1480, 1452, 1438, 1086, 1066, 1055, 1025, 923, 747, 720, 693.

MS (EI, 70 eV): *m/z* (%) = 149 (12), 132 (11), 131 (100), 129 (12), 110 (19), 91 (22).

HRMS (EI-orbitrap): *m/z:* [M] calc. for [$C_{16}H_{18}OS$]: 258.1078; found 226.1072.

***trans*-2-((2-hydroxycyclohexyl)-2-phenylacetonitrile (10ao) mixture of diastereoisomeres**

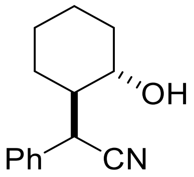

According to **TP4**, a solution of 3-(chloromethyl)heptane (**2**, 0.2 M, 2.0 equiv) in *n*-hexane was prepared. The solution of **2** was pumped through the activated sodium packed-bed reactor (see **TP1**) by pump A (flow rate: 2.0 mL/min) into the precooling loop (V_{pre2} = 0.35 mL) at 25 °C. Subsequently upon reaching the steady state, it was injected for 2 min into a flask charged with a solution of TMEDA (0.80 mmol, 2.0 equiv) 2-phenylacetonitrile (**3m**, 47 mg, 0.40 mmol, 1.0 equiv) in hexane (2.0 mL) the mixture was stirred for 30 min at 25 °C. Before it was cooled to –20 °C and a solution of cyclohexene oxide (**9a**, 79 mg, 0.80 mmol, 2.0 equiv) in THF (2.0 mL) was added. The reaction mixture was stirred at –20 °C for 10 min followed by another 30 min at 25 °C, before sat. *aq.* NH_4Cl solution was added for quenching the reaction mixture. The aqueous layer was extracted three times with EtOAc (3×30 mL) and the combined organic layers were dried over anhydrous $MgSO_4$ and filtrated. After removal of the solvent, flash column chromatographical purification (silica gel, pentane:EtOAc = 90:10 → 80:20) afforded the title compound **10ao** as a colorless oil (53 mg, 0.25 mmol, 62% yield, d.r. 4.2:1).

Major:

^{1}H-NMR (400 MHz, $CDCl_3$): δ / ppm = 7.40 – 7.29 (m, 5H), 4.72 (d, *J* = 3.4 Hz, 1H), 3.70 – 3.62 (m, 1H), 2.13 – 1.98 (m, 2H), 1.79 – 1.70 (m, 1H), 1.70 – 1.59 (m, 2H), 1.54 1.46 (m, 1H), 1.40 1.17 (m. 3H), 1.12 – 0.95 (m, 1H)

^{13}C-NMR (100 MHz, $CDCl_3$): δ / ppm = 134.7, 128.9 (2C), 127.1 (2C), 127.9, 119.3, 72.1, 50.2, 38.5, 36.1, 25.4, 25.1, 24.5.

Minor:

^{1}H-NMR (400 MHz, $CDCl_3$): δ / ppm = 7.40 – 7.29 (m, 5H), 4.59 (d, *J* = 3.7 Hz, 1H), 3.13 – 3.04 (m, 1H), 1.97 – 1.86 (m, 2H), 1.79 – 1.70 (m, 1H), 1.70 – 1.59 (m, 2H), 1.40 1.17 (m. 3H), 1.12 – 0.95 (m, 1H), 0.94 – 0.83 (m, 1H).

^{13}C-NMR (100 MHz, $CDCl_3$): δ / ppm = 132.2, 129.3 (2C), 128.6 (2C), 128.1, 121.6, 70.1, 48.7, 37.6, 36.1, 26.1, 25.2, 24.6.

Mixture:

IR (Diamond-ATR, neat): $\tilde{\nu}$ / cm^{-1} = 3426, 2931, 2858, 1494, 1452, 1354, 1344, 1067, 1045, 1031, 914, 754, 700, 678.

MS (EI, 70 eV): *m/z* (%) = 197 (18), 130 (17), 117 (100), 116 (11), 81 (26).

HRMS (EI-orbitrap): *m/z:* [M] calc. for [$C_{14}H_{17}NO$]: 215.1310; found 215.1295.

***trans*-2-benzhydrylcyclohexan-1-ol (10ap)**

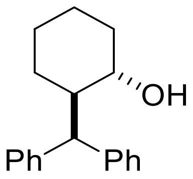

According to **TP3**, a solution of 3-(chloromethyl)heptane (**2**, 0.2 M, 2.0 equiv) in *n*-hexane was prepared. The solution of **2** was pumped through the activated sodium packed-bed reactor (see **TP1**) by pump A (flow rate: 2.0 mL/min) into the precooling loop (V_{pre2} = 0.35 mL) at 25 °C. Subsequently upon reaching the steady state, it was injected (2 min) into a flask charged with TMEDA (0.80 mmol, 2.0 equiv) in diphenylmethane (**3n**, 1.0 mL) and the resulting mixture was stirred at 25 °C for 30 min. The reaction flask was cooled to –20 °C and a solution of cyclohexene oxide (**9a**, 39 mg, 0.40 mmol, 1.0 equiv) in THF (1.0 mL) was added The reaction mixture was stirred at –20 °C for 10 min followed by another 30 min at 25 °C, before sat. *aq.* NH_4Cl solution was added for quenching the reaction mixture. The aqueous layer was extracted three times with EtOAc (3×30 mL) and the combined organic layers were dried over anhydrous $MgSO_4$ and filtrated. After removal of the solvent, flash column chromatographical purification (silica gel, pentane:EtOAc = 95:5 → 85:15) afforded the title compound **10ap** as a colorless oil (103 mg, 0.39 mmol, 97% yield).

^{1}H-NMR (400 MHz, $CDCl_3$): δ / ppm = 7.37 (d, *J* = 7.3 Hz, 2H), 7.33 – 7.26 (m, 6H), 7.24 – 7.16 (m, 2H), 4.21 (d, *J* = 8.0 Hz, 1H), 3.45 (td, *J* = 8.7, 4.0 Hz, 1H), 2.39 – 2.27 (m, 1H), 1.97 – 1.88 (m, 1H), 1.82 – 1.64 (m, 2H), 1.65 – 1.52 (m, 1H), 1.45 – 1.18 (m, 4H), 1.03 – 0.88 (m, 1H).

^{13}C-NMR (100 MHz, $CDCl_3$): δ / ppm = 144.3, 143.3, 129.2 (2C), 128.8 (2C), 128.4 (2C), 128.3 (2C), 126.5, 126.3, 72.7, 53.8, 47.5, 34.4, 27.8, 24.7, 24.0.

IR (Diamond-ATR, neat): $\tilde{\nu}$ / cm^{-1} = 3340, 3060, 3026, 2930, 2857, 1599, 1495, 1449, 1355, 1266, 1064, 1041, 1032, 1023, 1002, 970, 785, 764, 753, 745, 731, 700, 674.

MS (EI, 70 eV): *m/z* (%) = 248 (16), 206 (10), 205 (10), 192 (10), 168 (14), 167 (100), 165 (44), 152 (23).

HRMS (EI-orbitrap): *m/z:* [M – H_2O] calc. for [$C_{19}H_{20}$]: 248.1565; found 248.1559.

(*S*)-1-(benzyloxy)-4,4-diphenylbutan-2-ol (10aq)

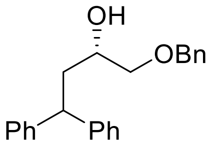

According to **TP3**, a solution of 3-(chloromethyl)heptane (**2**, 0.2 M, 2.0 equiv) in *n*-hexane was prepared. The solution of **2** was pumped through the activated sodium packed-bed reactor (see **TP1**) by pump A (flow rate: 2.0 mL/min) into the precooling loop (V_{pre2} = 0.35 mL) at 25 °C. Subsequently upon reaching the steady state, it was injected (2 min) into a flask charged with TMEDA (0.80 mmol, 2.0 equiv) in diphenylmethane (**3n**, 1.0 mL) and the resulting mixture was stirred at 25 °C for 30 min. The reaction flask was cooled to –20 °C and a solution of (*S*)-2-((benzyloxy)methyl)oxirane (**9m**, 66 mg, 0.40 mmol, 1.0 equiv) in THF (1.0 mL) was added The reaction mixture was stirred at –20 °C for 10 min followed by another 30 min at 25 °C, before sat. *aq.* NH_4Cl solution was added for quenching the reaction mixture. The aqueous layer was extracted three times with EtOAc (3×30 mL) and the combined organic layers were dried over anhydrous $MgSO_4$ and filtrated. After removal of the solvent, flash column chromatographical purification (silica gel, pentane:EtOAc = 90:10 → 85:15) afforded the title compound **10aq** as a colorless oil (110 mg, 0.33 mmol, 83% yield).

^{1}H-NMR (400 MHz, $CDCl_3$): δ / ppm = 7.35 – 7.21 (m, 13H), 7.19 – 7.12 (m, 2H), 4.48 (s, 2H), 4.25 (t, *J* = 8.0 Hz, 1H), 3.65 (t, *J* = 9.9 Hz, 1H), 3.45 (dd, *J* = 9.4, 3.1 Hz, 1H), 3.33 (dd, *J* = 9.4, 7.4 Hz, 1H), 2.43 – 2.30 (m, 1H), 2.20 – 2.08 (m, 2H).

^{13}C-NMR (100 MHz, $CDCl_3$): δ / ppm = 145.1, 144.1, 138.0, 128.6 (2C), 128.6 (4C), 128.3 (2C), 127.9, 127.9 (2C), 127.8 (2C), 126.4, 126.3, 74.7, 73.4, 68.3, 46.9, 39.0.

IR (Diamond-ATR, neat): $\tilde{\nu}$ / cm^{-1} = 3434, 3061, 3027, 2922, 2856, 1599, 1494, 1451, 1364, 1306, 1259, 1203, 1075, 1029, 1001, 945, 907, 871, 787, 749, 737, 695.

MS (EI, 70 eV): *m/z* (%) = 241 (10), 223 (15), 181 (73), 168 (13), 167 (100), 166 (11), 165 (27), 152 (12), 103 (20), 91 (38).

HRMS (EI-orbitrap): *m/z:* [M] calc. for [$C_{23}H_{24}O_2$]: 332.1766; found 332.1765.

(*S*)-1-((tert-butyldimethylsilyl)oxy)-4,4-diphenylbutan-2-ol (10ar) mixture of regioisomers

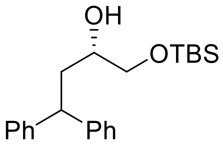

According to **TP3**, a solution of 3-(chloromethyl)heptane (**2**, 0.2 M, 2.0 equiv) in *n*-hexane was prepared. The solution of **2** was pumped through the activated sodium packed-bed reactor (see **TP1**) by pump A (flow rate: 2.0 mL/min) into the precooling loop (V_{pre2} = 0.35 mL) at 25 °C. Subsequently upon reaching the steady state, it was injected (2 min) into a flask charged with TMEDA (0.80 mmol, 2.0 equiv) in diphenylmethane (**3n**, 1.0 mL) and the resulting mixture was stirred at 25 °C for 30 min. The reaction flask was cooled to –20 °C and a solution of (S)-tert-butyldimethyl(oxiran-2-ylmethoxy)silane (**9n**, 75 mg, 0.40 mmol, 1.0 equiv) in THF (1.0 mL) was added The reaction mixture was stirred at –20 °C for 10 min followed by another 30 min at 25 °C, before sat. *aq.* NH_4Cl solution was added for quenching the reaction mixture. The aqueous layer was extracted three times with EtOAc (3×30 mL) and the combined organic layers were dried over anhydrous $MgSO_4$ and filtrated. After removal of the solvent, flash column chromatographical purification (silica gel, pentane:EtOAc = 97:3 → 90:10) afforded the title compound **10ar** as a colorless oil (137 mg, 0.38 mmol, 96% yield, r.r. = 4.0:1.0).

^{1}H-NMR (400 MHz, $CDCl_3$): δ / ppm = 7.33 – 7.26 (m, 8H), 7.24 – 7.16 (m, 2H), 4.29 (dd, *J* = 9.0, 6.9 Hz, 1H), 3.61 (dd, *J* = 9.5, 3.3 Hz, 1H), 3.58 – 3.49 (m, 1H), 3.45 (dd, *J* = 9.5, 7.0 Hz, 1H), 2.39 (s, 1H), 2.21 – 2.12 (m, 2H), 0.88 (s, 9H), 0.07 (s, 3H), 0.05 (s, 3H).

^{13}C-NMR (100 MHz, $CDCl_3$): δ / ppm = 145.3, 144.3, 128.7 (2C), 128.6 (2C), 128.3 (2C), 127.8 (2C), 126.4, 126.3, 69.6, 67.5, 47.1, 38.8, 26.0 (3C), 18.4, -5.2, -5.3.

IR (Diamond-ATR, neat): $\tilde{\nu}$ / cm^{-1} = 3419, 2953, 2928, 2885, 2856, 1494, 1472, 1462, 1451, 1253, 1109, 1074, 1041, 968, 948, 834, 811, 776, 755, 745, 699.

MS (EI, 70 eV): *m/z* (%) = 207 (56), 181 (10), 180 (12), 168 (14), 167 (100), 166 (11), 165 (30), 152 (12), 129 (83), 105 (11), 91 (64), 75 (43), 73 (15).

HRMS (EI-orbitrap): *m/z:* [M] calc. for [$C_{22}H_{32}O_2Si$]: 356.2172; found 356.2156.

(*S*)-2-((tert-butyldimethylsilyl)oxy)-4,4-diphenylbutan-1-ol (10ar´)

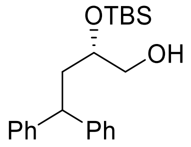

^{1}H-NMR (400 MHz, $CDCl_3$): δ / ppm = 7.31 – 7.20 (m, 8H), 7.20 – 7.14 (m, 2H), 4.05 (t, *J* = 7.9 Hz, 1H), 3.68 – 3.61 (m, 1H), 3.57 (d, *J* = 11.1 Hz, 1H), 3.53 – 3.43 (m, 1H), 2.33 (ddd, *J* = 14.2, 8.0, 6.3 Hz, 1H), 2.28 – 2.18 (m, 1H), 1.84 (dd, *J* = 7.2, 5.3 Hz, 1H), 0.91 (s, 9H), -0.02 (s, 3H), -0.04 (s, 3H).

^{13}C-NMR (100 MHz, $CDCl_3$): δ / ppm = 144.9, 144.3, 128.7 (2C), 128.7 (2C), 128.0 (2C), 127.9 (2C), 126.4 (2C), 71.2, 66.3, 47.4, 40.1, 26.0 (3C), 18.2, -4.3, -4.4.

IR (Diamond-ATR, neat): $\tilde{\nu}$ / cm^{-1} = 3411, 3062, 3027, 2953, 2928, 2885, 2856, 1600, 1494, 1472, 1462, 1451, 1388, 1361, 1253, 1109, 1074, 1041, 1004, 968, 948, 865, 834, 811, 776, 755, 745, 699, 670.

MS (EI, 70 eV): *m/z* (%) = 207 (39), 196 (10), 195 (68), 177 (27), 168 (13), 167 (100), 166 (12), 165 (30), 152 (15), 129 (40), 91 (54), 75 (69), 73 (13).

HRMS (EI-orbitrap): *m/z:* [M – C_4H_9] calc. for [$C_{18}H_{23}O_2$]: 299.1467; found 299.1460.

4,4-diphenylbutan-1-ol (10as)

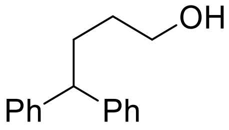

According to **TP3**, a solution of 3-(chloromethyl)heptane (**2**, 0.2 M, 2.0 equiv) in *n*-hexane was prepared. The solution of **2** was pumped through the activated sodium packed-bed reactor (see **TP1**) by pump A (flow rate: 2.0 mL/min) into the precooling loop (V_{pre2} = 0.35 mL) at 25 °C. Subsequently upon reaching the steady state, it was injected (2 min) into a flask charged with TMEDA (0.80 mmol, 2.0 equiv) in diphenylmethane (**3n**, 1.0 mL) and the resulting mixture was stirred at 25 °C for 30 min. The reaction flask was cooled to –20 °C and a solution of oxetane (**9h**, 23 mg, 0.40 mmol, 1.0 equiv) in THF (1.0 mL) was added The reaction mixture was stirred at –20 °C for 10 min followed by another 30 min at 25 °C, before sat. *aq.* NH_4Cl solution was added for quenching the reaction mixture. The aqueous layer was extracted three times with EtOAc (3×30 mL) and the combined organic layers were dried over anhydrous $MgSO_4$ and filtrated. After removal of the solvent, flash column chromatographical purification (silica gel, pentane:EtOAc = 90:10 → 80:20) afforded the title compound **10as** as a colorless oil (68 mg, 0.30 mmol, 75% yield).

^{1}H-NMR (400 MHz, $CDCl_3$): δ / ppm = 7.37 – 7.26 (m, 8H), 7.26 – 7.17 (m, 2H), 3.95 (t, *J* = 7.9 Hz, 1H), 3.66 (t, *J* = 6.5 Hz, 2H), 2.24 – 2.09 (m, 2H), 1.66 – 1.47 (m, 3H).

^{13}C-NMR (100 MHz, $CDCl_3$): δ / ppm = 145.0 (2C), 128.5 (4C), 127.9(4C), 126.3 (2C), 62.9, 51.2, 31.9, 31.3.

IR (Diamond-ATR, neat): $\tilde{\nu}$ / cm^{-1} = 3331, 3025, 2938, 2866, 1600, 1494, 1450, 1052, 1031, 761, 747, 734, 697.

MS (EI, 70 eV): *m/z* (%) = 180 (14), 168 (14), 167 (100), 165 (59), 152 (27), 130 (15).

HRMS (EI-orbitrap): *m/z:* [M] calc. for [$C_{16}H_{18}O$]: 226.1358; found 226.1353.

2-(phenyl-d_5)adamantan-2-ol (10at)

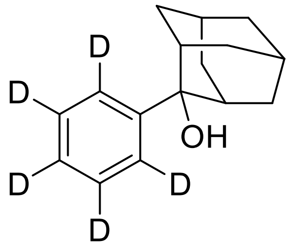

According to **TP3**, a solution of 3-(chloromethyl)heptane (**2**, 0.2 M, 2.0 equiv) in *n*-hexane was prepared. The solution of **2** was pumped through the activated sodium packed-bed reactor (see **TP1**) by pump A (flow rate: 2.0 mL/min) into the precooling loop (V_{pre2} = 0.35 mL) at 25 °C. Subsequently upon reaching the steady state, it was injected (1 min) into a flask charged with TMEDA (0.40 mmol, 2.0 equiv) in benzene-d_6 (**3n**, 0.5 mL) and the resulting mixture was stirred at 25 °C for 30 min. The reaction flask was cooled to –20 °C and a solution of adamantanone (**9k**, 30 mg, 0.20 mmol, 1.0 equiv) in THF (1.0 mL) was added The reaction mixture was stirred at –20 °C for 10 min followed by another 30 min at 25 °C, before sat. *aq.* NH_4Cl solution was added for quenching the reaction mixture. The aqueous layer was extracted three times with EtOAc (3×30 mL) and the combined organic layers were dried over anhydrous $MgSO_4$ and filtrated. After removal of the solvent, flash column chromatographical purification (silica gel, pentane:EtOAc = 95:5) afforded the title compound **10at** as a colorless solid (44 mg, 0.19 mmol, 94% yield).

^{1}H-NMR (400 MHz, $CDCl_3$): δ / ppm = 2.57 (s, 2H), 2.41 (d, *J* = 12.0 Hz, 2H), 1.91 (s, 1H), 1.79 – 1.65 (m, 9H), 1.54 (s, 1H).

^{13}C-NMR (100 MHz, $CDCl_3$): δ / ppm = 145.3, 128.3 (t, *J* = 24.3 Hz, 2C), 126.9 (t, *J* = 24.6 Hz), 125.1 (t, *J* =23.8 Hz, 2C), 75.8, 37.8, 35.7 (2C), 35.0 (2C), 33.1 (2C), 27.5, 27.0.

IR (Diamond-ATR, neat): $\tilde{\nu}$ / cm^{-1} = 2938, 2902, 2853, 2361, 1450, 1102, 1043, 1003, 997, 934, 913.

MS (EI, 70 eV): *m/z* (%) = 233 (25), 216 (17), 215 (100), 190 (22), 151 (13), 150 (17), 112 (20), 110 (88), 91 (11), 84 (18), 82 (16), 80 (11), 79 (13).

HRMS (EI-orbitrap): *m/z:* [M] calc. for [$C_{16}H_{15}D_5O$]: 233.1828; found 233.1822.

m.p. (°C): 78.5 – 80.4.

1,3-dimethyl-5-tridecylbenzene (13a)

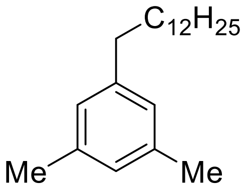

According to **TP3**, a solution of 3-(chloromethyl)heptane (**2**, 0.2 M, 2.0 equiv) in *n*-hexane was prepared. The solution of **2** was pumped through the activated sodium packed-bed reactor (see **TP1**) by pump A (flow rate: 2.0 mL/min) into the precooling loop (V_{pre2} = 0.35 mL) at 25 °C. Subsequently upon reaching the steady state, it was injected (2 min) into a flask charged with TMEDA (0.80 mmol, 2.0 equiv) in mesitylene (**3a**, 1.0 mL) and the resulting mixture was stirred at 25 °C for 30 min. The reaction flask was cooled to –20 °C and a solution of 1-chlorododecane (**12a**, 82 mg, 0.40 mmol, 1.0 equiv) in THF (1.0 mL) was added The reaction mixture was stirred at –20 °C for 10 min followed by another 30 min at 25 °C, before sat. *aq.* NH_4Cl solution was added for quenching the reaction mixture. The aqueous layer was extracted three times with EtOAc (3×30 mL) and the combined organic layers were dried over anhydrous $MgSO_4$ and filtrated. After removal of the solvent, flash column chromatographical purification (silica gel, pentane) afforded the title compound **13a** as a colorless oil (101 mg, 0.35 mmol, 88% yield).

^{1}H-NMR (400 MHz, $CDCl_3$): δ / ppm = 6.84 (s, 1H), 6.83 (s, 2H), 2.59 – 2.51 (m, 2H), 2.32 (s, 6H), 1.61 (p, *J* = 7.4 Hz, 2H), 1.31 (d, *J* = 17.4 Hz, 20H), 0.91 (t, *J* = 6.8 Hz, 3H).

^{13}C-NMR (100 MHz, $CDCl_3$): δ / ppm = 143.1, 137.8 (2C), 127.3, 126.4 (2C), 36.0, 32.1, 31.8, 29.9, 29.9 (2C), 29.8, 29.8, 29.7, 29.6, 29.5, 22.9, 21.4 (2C), 14.3.

IR (Diamond-ATR, neat): $\tilde{\nu}$ / cm^{-1} = 3015, 2955, 2922, 2853, 2361, 1607, 1466, 1377, 842, 722, 702.

MS (EI, 70 eV): *m/z* (%) = 288 (14), 133 (10), 120 (100), 119 (44), 105 (31).

HRMS (EI-orbitrap): *m/z:* [M] calc. for [$C_{21}H_{36}$]: 288.2817; found 288.2813.

1,3-dimethyl-5-(4-phenylbutyl)benzene (13b)

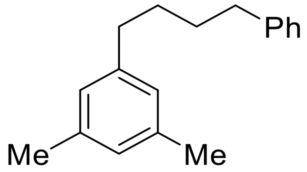

According to **TP3**, a solution of 3-(chloromethyl)heptane (**2**, 0.2 M, 2.0 equiv) in *n*-hexane was prepared. The solution of **2** was pumped through the activated sodium packed-bed reactor (see **TP1**) by pump A (flow rate: 2.0 mL/min) into the precooling loop (V_{pre2} = 0.35 mL) at 25 °C. Subsequently upon reaching the steady state, it was injected (2 min) into a flask charged with TMEDA (0.80 mmol, 2.0 equiv) in mesitylene (**3a**, 1.0 mL) and the resulting mixture was stirred at 25 °C for 30 min. The reaction flask was cooled to –20 °C and a solution of (3-chloropropyl)benzene (**12b**, 62 mg, 0.40 mmol, 1.0 equiv) in THF (1.0 mL) was added The reaction mixture was stirred at –20 °C for 10 min followed by another 30 min at 25 °C, before sat. *aq.* NH_4Cl solution was added for quenching the reaction mixture. The aqueous layer was extracted three times with EtOAc (3×30 mL) and the combined organic layers were dried over anhydrous $MgSO_4$ and filtrated. After removal of the solvent, flash column chromatographical purification (silica gel, pentane:EtOAc = 99:1 → 98:2) afforded the title compound **13b** as a colorless oil (88 mg, 0.37 mmol, 92% yield).

^{1}H-NMR (400 MHz, $CDCl_3$): δ / ppm = 7.42 – 7.34 (m, 2H), 7.31 – 7.25 (m, 3H), 6.94 6.88 (m, 3H), 2.74 (t, *J* = 7.1 Hz, 2H), 2.67 (t, *J* = 7.1 Hz, 2H), 2.39 (s, 6H), 1.83 – 1.68 (m, 4H).

^{13}C-NMR (100 MHz, $CDCl_3$): δ / ppm = 142.8, 142.6, 137.8 (2C), 128.6 (2C), 128.4 (2C), 127.4, 126.4, 125.8 (2C), 36.0, 35.8, 31.4, 31.3, 21.4 (2C).

IR (Diamond-ATR, neat): $\tilde{\nu}$ / cm^{-1} = 3024, 3017, 3015, 2933, 2930, 2923, 2916, 2855, 1606, 1496, 1462, 1460, 1453, 844, 747, 698.

MS (EI, 70 eV): *m/z* (%) = 238 (36), 195 (18), 133 (16), 120 (100), 119 (51), 117 (12), 115 (12), 105 (60), 91 (33).

HRMS (EI-orbitrap): *m/z:* [M] calc. for [$C_{18}H_{22}$]: 238.1722; found 238.1716.

(4-(3,5-dimethylphenyl)butyl)trimethylsilane (13c)

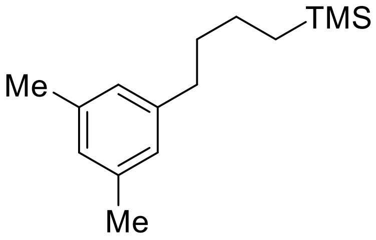

According to **TP3**, a solution of 3-(chloromethyl)heptane (**2**, 0.2 M, 2.0 equiv) in *n*-hexane was prepared. The solution of **2** was pumped through the activated sodium packed-bed reactor (see **TP1**) by pump A (flow rate: 2.0 mL/min) into the precooling loop (V_{pre2} = 0.35 mL) at 25 °C. Subsequently upon reaching the steady state, it was injected (2 min) into a flask charged with TMEDA (0.80 mmol, 2.0 equiv) in mesitylene (**3a**, 1.0 mL) and the resulting mixture was stirred at 25 °C for 30 min. The reaction flask was cooled to –20 °C and a solution of (3-chloropropyl)trimethylsilane (**12c**, 60 mg, 0.40 mmol, 1.0 equiv) in THF (1.0 mL) was added The reaction mixture was stirred at –20 °C for 10 min followed by another 30 min at 25 °C, before sat. *aq.* NH_4Cl solution was added for quenching the reaction mixture. The aqueous layer was extracted three times with EtOAc (3×30 mL) and the combined organic layers were dried over anhydrous $MgSO_4$ and filtrated. After removal of the solvent, flash column chromatographical purification (silica gel, pentane:EtOAc = 100:0 → 98:2) afforded the title compound **13c** as a colorless oil (79 mg, 0.34 mmol, 84% yield).

^{1}H-NMR (400 MHz, $CDCl_3$): δ / ppm = 6.85 (d, *J* = 5.8 Hz, 3H), 2.61 – 2.54 (m, 2H), 2.34 (s, 6H), 1.66 (dt, *J* = 15.3, 7.6 Hz, 2H), 1.45 – 1.34 (m, 2H), 0.61 – 0.54 (m, 2H), 0.03 (s, 9H).

^{13}C-NMR (100 MHz, $CDCl_3$): δ / ppm = 143.0, 137.8 (2C), 127.3, 127.1, 126.4 (2C), 35.7, 23.9, 21.4 (2C), 16.6, -1.5 (3C).

IR (Diamond-ATR, neat): $\tilde{\nu}$ / cm^{-1} = 2952, 2921, 2855, 1607, 1460, 1258, 1247, 858, 833, 757, 746, 701, 690.

MS (EI, 70 eV): *m/z* (%) = 219 (10), 217 (13), 157 (15), 145 (10), 121 (10), 120 (100), 119 (42), 117 (10), 105 (33), 91 (12), 73 (37).

HRMS (EI-orbitrap): *m/z:* [M] calc. for [$C_{15}H_{26}Si$]: 234.1804; found 234.1796.

1-(((1*R*,2*S*)-2-methoxycyclohexyl)methyl)-3,5-dimethylbenzene (13d)

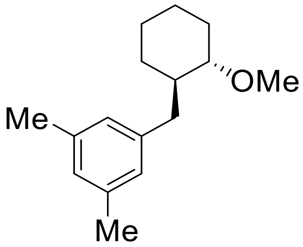

According to **TP3**, a solution of 3-(chloromethyl)heptane (**2**, 0.2 M, 2.0 equiv) in *n*-hexane was prepared. The solution of **2** was pumped through the activated sodium packed-bed reactor (see **TP1**) by pump A (flow rate: 2.0 mL/min) into the precooling loop (V_{pre2} = 0.35 mL) at 25 °C. Subsequently upon reaching the steady state, it was injected (2 min) into a flask charged with TMEDA (0.80 mmol, 2.0 equiv) in mesitylene (**3a**, 1.0 mL) and the resulting mixture was stirred at 25 °C for 30 min. The reaction flask was cooled to –20 °C and a solution of (1*R*,2*S*)-1-chloro-2-methoxycyclohexane (**12d**, 59 mg, 0.40 mmol, 1.0 equiv) in THF (1.0 mL) was added The reaction mixture was stirred at –20 °C for 10 min followed by another 30 min at 25 °C, before sat. *aq.* NH_4Cl solution was added for quenching the reaction mixture. The aqueous layer was extracted three times with EtOAc (3×30 mL) and the combined organic layers were dried over anhydrous $MgSO_4$ and filtrated. After removal of the solvent, flash column chromatographical purification (silica gel, pentane:EtOAc = 100:0 → 99:1) afforded the title compound **13d** as a colorless oil (46 mg, 0.20 mmol, 49% yield).

^{1}H-NMR (400 MHz, $CDCl_3$): δ / ppm = 6.82 (s, 1H), 6.79 (s, 2H), 3.41 (s, 3H), 3.16 – 3.08 (m, 1H), 2.86 – 2.75 (m, 1H), 2.29 (s, 6H), 2.25 – 2.11 (m, 2H), 1.80 – 1.71 (m, 1H), 1.70 – 1.63 (m, 1H), 1.60 – 1.51 (m, 2H), 1.26 – 1.02 (m, 3H), 0.99 – 0.84 (m, 1H).

^{13}C-NMR (100 MHz, $CDCl_3$): δ / ppm = 141.1, 137.5 (2C), 127.5, 127.3 (2C), 83.2, 56.2, 45.2, 38.6, 30.5, 30.2, 25.5, 24.8, 21.4 (2C).

IR (Diamond-ATR, neat): $\tilde{\nu}$ / cm^{-1} = 2923, 2856, 2818, 1606, 1461, 1448, 1373, 1194, 1181, 1111, 1098, 846, 707.

MS (EI, 70 eV): *m/z* (%) = 200 (19), 185 (24), 157 (15), 143 (20), 132 (22), 120 (100), 119 (37), 117 (17), 115 (10), 113 (13), 105 (70), 91 (16).

HRMS (EI-orbitrap): *m/z:* [M] calc. for [$C_{16}H_{24}O$]: 232.1827; found: 232.1822.

1,3-dimethyl-5-(3-(trifluoromethyl)phenethyl)benzene (13e)

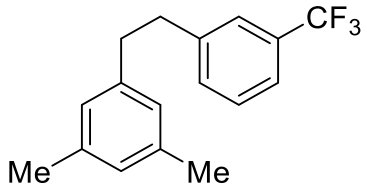

According to **TP3**, a solution of 3-(chloromethyl)heptane (**2**, 0.2 M, 2.0 equiv) in *n*-hexane was prepared. The solution of **2** was pumped through the activated sodium packed-bed reactor (see **TP1**) by pump A (flow rate: 2.0 mL/min) into the precooling loop (V_{pre2} = 0.35 mL) at 25 °C. Subsequently upon reaching the steady state, it was injected (2 min) into a flask charged with TMEDA (0.80 mmol, 2.0 equiv) in mesitylene (**3a**, 1.0 mL) and the resulting mixture was stirred at 25 °C for 30 min. The reaction flask was cooled to –20 °C and a solution of 1-(chloromethyl)-3-(trifluoromethyl)benzene (**12e**, 78 mg, 0.40 mmol, 1.0 equiv) in THF (1.0 mL) was added The reaction mixture was stirred at –20 °C for 10 min followed by another 30 min at 25 °C, before sat. *aq.* NH_4Cl solution was added for quenching the reaction mixture. The aqueous layer was extracted three times with EtOAc (3×30 mL) and the combined organic layers were dried over anhydrous $MgSO_4$ and filtrated. After removal of the solvent, flash column chromatographical purification (silica gel, pentane) afforded the title compound **13e** as a colorless oil (76 mg, 0.27 mmol, 68% yield).

^{1}H-NMR (400 MHz, $CDCl_3$): δ / ppm = 7.52 – 7.36 (m, 4H), 6.90 (s, 1H), 6.84 (s, 2H), 3.03 – 2.93 (m, 2H), 2.92 – 2.84 (m, 2H), 2.33 (s, 6H).

^{13}C-NMR (100 MHz, $CDCl_3$): δ / ppm = 142.9, 141.2, 138.1 (2C), 132.0 (q, *J* = 1.5 Hz), 130.7 (q, *J* = 31.9 Hz), 128.8, 127.9, 126.4 (2C), 125.3 (q, *J* = 3.8 Hz), 124.4 (q, *J* = 272.4 Hz), 122.9 (q, *J* = 3.8 Hz), 38.0, 37.8, 21.4 (2C).

IR (Diamond-ATR, neat): $\tilde{\nu}$ / cm^{-1} = 3016, 2921, 2863, 1607, 1450, 1347, 1327, 1198, 1162, 1122, 1094, 1075, 898, 844, 799, 701.

MS (EI, 70 eV): *m/z* (%) = 120 (10), 119 (100), 91 (14).

HRMS (EI-orbitrap): *m/z:* [M] calc. for [$C_{17}H_{17}F_3$]: 278.1282; found 278.1276.

4-(3,5-dimethylphenethyl)-3,5-dimethylisoxazole (13f)

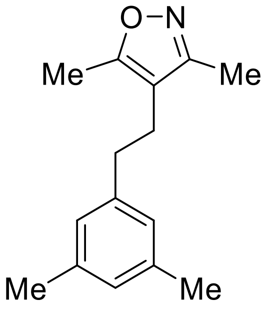

According to **TP3**, a solution of 3-(chloromethyl)heptane (**2**, 0.2 M, 2.0 equiv) in *n*-hexane was prepared. The solution of **2** was pumped through the activated sodium packed-bed reactor (see **TP1**) by pump A (flow rate: 2.0 mL/min) into the precooling loop (V_{pre2} = 0.35 mL) at 25 °C. Subsequently upon reaching the steady state, it was injected (2 min) into a flask charged with TMEDA (0.80 mmol, 2.0 equiv) in mesitylene (**3a**, 1.0 mL) and the resulting mixture was stirred at 25 °C for 30 min. The reaction flask was cooled to –20 °C and a solution of 4-(chloromethyl)-3,5-dimethylisoxazole (**12f**, 58 mg, 0.40 mmol, 1.0 equiv) in THF (1.0 mL) was added The reaction mixture was stirred at –20 °C for 10 min followed by another 30 min at 25 °C, before sat. *aq.* NH_4Cl solution was added for quenching the reaction mixture. The aqueous layer was extracted three times with EtOAc (3×30 mL) and the combined organic layers were dried over anhydrous $MgSO_4$ and filtrated. After removal of the solvent, flash column chromatographical purification (silica gel, pentane:EtOAc = 97:3 → 90:10) afforded the title compound **13f** as a colorless oil (52 mg, 0.23 mmol, 57% yield).

^{1}H-NMR (400 MHz, $CDCl_3$): δ / ppm = 6.85 (s, 1H), 6.71 (s, 2H), 2.65 (t, *J* = 7.2 Hz, 2H), 2.56 (t, *J* = 7.1 Hz, 2H), 2.28 (s, 6H), 2.12 (s, 3H), 2.09 (s, 3H).

^{13}C-NMR (100 MHz, $CDCl_3$): δ / ppm = 165.2, 159.8, 140.8, 138.1 (2C), 127.9, 126.5 (2C), 112.9, 36.1, 24.5, 21.3 (2C), 10.7, 10.2.

IR (Diamond-ATR, neat): $\tilde{\nu}$ / cm^{-1} = 3011, 2916, 2859, 1639, 1451, 1424, 1261, 1194, 890, 842, 699.

MS (EI, 70 eV): *m/z* (%) = 229 (26), 214 (44), 221 (13), 186 (39), 145 (11), 119 (100), 117 (11) 115 (10), 110 (14), 91 (24), 68 (23).

HRMS (EI-orbitrap): *m/z:* [M] calc. for [$C_{15}H_{19}NO$]: 229.1467; found 229.1461.

(*E*)-1,3-dimethyl-5-(4-phenylbut-3-en-1-yl)benzene (13g)

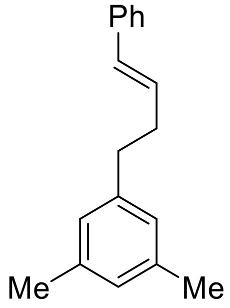

According to **TP3**, a solution of 3-(chloromethyl)heptane (**2**, 0.2 M, 2.0 equiv) in *n*-hexane was prepared. The solution of **2** was pumped through the activated sodium packed-bed reactor (see **TP1**) by pump A (flow rate: 2.0 mL/min) into the precooling loop (V_{pre2} = 0.35 mL) at 25 °C. Subsequently upon reaching the steady state, it was injected (2 min) into a flask charged with TMEDA (0.80 mmol, 2.0 equiv) in mesitylene (**3a**, 1.0 mL) and the resulting mixture was stirred at 25 °C for 30 min. The reaction flask was cooled to –20 °C and a solution of (*E*)-(3-chloroprop-1-en-1-yl)benzene (**12g**, 61 mg, 0.40 mmol, 1.0 equiv) in THF (1.0 mL) was added The reaction mixture was stirred at –20 °C for 10 min followed by another 30 min at 25 °C, before sat. *aq.* NH_4Cl solution was added for quenching the reaction mixture. The aqueous layer was extracted three times with EtOAc (3×30 mL) and the combined organic layers were dried over anhydrous $MgSO_4$ and filtrated. After removal of the solvent, flash column chromatographical purification (silica gel, pentane) afforded the title compound **13g** as a colorless oil (52 mg, 0.22 mmol, 55% yield).

^{1}H-NMR (400 MHz, $CDCl_3$): δ / ppm = 7.40 – 7.29 (m, 4H), 7.25 – 7.19 (m, 1H), 6.88 (s, 3H), 6.46 (dt, *J* = 15.7, 1.4 Hz, 1H), 6.30 (dt, *J* = 15.8, 6.7 Hz, 1H), 2.74 (dd, *J* = 9.4, 6.5 Hz, 2H), 2.61 – 2.51 (m, 2H), 2.34 (s, 6H).

^{13}C-NMR (100 MHz, $CDCl_3$): δ / ppm = 141.9, 138.0 (2C), 137.9, 130.4, 130.3, 128.6 (2C), 127.7, 127.0, 126.4 (2C), 126.1 (2C), 35.9, 35.2, 21.4 (2C).

IR (Diamond-ATR, neat): $\tilde{\nu}$ / cm^{-1} = 3431, 3299, 3028, 2923, 2874, 2855, 1718, 1700, 1684, 1603, 1493, 1451, 1267, 1179, 1076, 1065, 1030, 755, 700.

MS (EI, 70 eV): *m/z* (%) = 145 (18), 119 (79), 118 (10), 117 (100), 115 (56), 91 (27).

HRMS (EI-orbitrap): *m/z:* [M] calc. for [$C_{18}H_{20}$]: 236.1565; found 236.1558.

(7-chloroheptyl)benzene (13h)

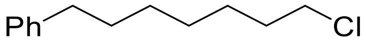

According to **TP4**, a solution of 3-(chloromethyl)heptane (**2**, 0.2 M, 2.0 equiv) in *n*-hexane was prepared. The solution of **2** was pumped through the activated sodium packed-bed reactor (see **TP1**) by pump A (flow rate: 2.0 mL/min) into the precooling loop (V_{pre2} = 0.35 mL) at 25 °C. Subsequently upon reaching the steady state, it was injected for 2 min into a flask charged with a solution of TMEDA (0.80 mmol, 2.0 equiv) and toluene (**3b**, 37 mg, 0.40 mmol, 1.0 equiv) in hexane (2.0 mL) the mixture was stirred for 30 min at 25 °C. Before it was cooled to –20 °C and a solution of 1,6-dichlorohexane (**12h**, 124 mg, 0.80 mmol, 2.0 equiv) in THF (2.0 mL) was added. The reaction mixture was stirred at –20 °C for 10 min followed by another 30 min at 25 °C, before sat. *aq.* NH_4Cl solution was added for quenching the reaction mixture. The aqueous layer was extracted three times with EtOAc (3×30 mL) and the combined organic layers were dried over anhydrous $MgSO_4$ and filtrated. After removal of the solvent, flash column chromatographical purification (silica gel, pentane:EtOAc = 100:0 → 98:2) afforded the title compound **13h** as colorless oil (73 mg, 0.35 mmol, 87% yield).

^{1}H-NMR (400 MHz, CDCl$_3$): δ / ppm = 7.32 – 7.26 (m, 2H), 7.22 – 7.14 (m, 3H), 3.54 (t, *J* = 6.7 Hz, 2H), 2.69 – 2.53 (m, 2H), 1.85 – 1.72 (m, 2H), 1.65 (d, *J* = 14.9 Hz, 2H), 1.49 – 1.32 (m, 6H).
^{13}C-NMR (100 MHz, CDCl$_3$): δ / ppm = 142.9, 128.5 (2C), 128.4 (2C), 125.7, 45.3, 36.0, 32.7, 31.5, 29.2, 28.9, 26.9.
IR (Diamond-ATR, neat): $\tilde{\nu}$ / cm^{-1} = 3062, 3026, 2928, 2855, 1604, 1496, 1463, 1453, 1308, 1030, 746, 726, 698.
MS (EI, 70 eV): *m/z* (%) = 210 (20), 92 (68), 91 (100).
HRMS (EI-orbitrap): *m/z:* [M] calc. for [$C_{13}H_{19}Cl$]: 210.1175; found 210.1170.

3,5-dimethyl-4-(2-(naphthalen-1-yl)ethyl)isoxazole (13i)

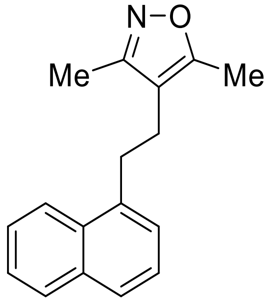

According to **TP4**, a solution of 3-(chloromethyl)heptane (**2**, 0.2 M, 2.0 equiv) in *n*-hexane was prepared. The solution of **2** was pumped through the activated sodium packed-bed reactor (see **TP1**) by pump A (flow rate: 2.0 mL/min) into the precooling loop (V_{pre2} = 0.35 mL) at 25 °C. Subsequently upon reaching the steady state, it was injected for 1 min into a flask charged with a solution of TMEDA (0.40 mmol, 2.0 equiv) and 1–methylnaphtalene (**3e**, 28 mg, 0.20 mmol, 1.0 equiv) in hexane (1.0 mL) the mixture was stirred for 30 min at 25 °C. Before it was cooled to –20 °C and a solution of 4-(chloromethyl)-3,5-dimethylisoxazole (**12f**, 58 mg, 0.40 mmol, 2.0 equiv) in THF (2.0 mL) was added. The reaction mixture was stirred at –20 °C for 10 min followed by another 30 min at 25 °C, before sat. *aq.* NH_4Cl solution was added for quenching the reaction mixture. The aqueous layer was extracted three times with EtOAc (3×30 mL) and the combined organic layers were dried over anhydrous $MgSO_4$ and filtrated. After removal of the solvent, flash column chromatographical purification (silica gel, pentane:EtOAc = 95:5 → 90:10) afforded the title compound **13i** as a colorless solid (35 mg, 0.14 mmol, 70% yield).

^{1}H-NMR (400 MHz, $CDCl_3$): δ / ppm = 8.01 (d, *J* = 8.3 Hz, 1H), 7.90 – 7.84 (m, 1H), 7.74 (d, *J* = 8.2 Hz, 1H), 7.58 – 7.47 (m, 2H), 7.35 (dd, *J* = 8.1, 7.1 Hz, 1H), 7.11 (d, *J* = 6.9 Hz, 1H), 3.21 (t, *J* = 7.3 Hz, 2H), 2.73 (t, *J* = 7.3 Hz, 2H), 2.08 (s, 3H), 1.90 (s, 3H).

^{13}C-NMR (100 MHz, $CDCl_3$): δ / ppm = 165.4, 159.7, 136.6, 134.0, 131.7, 129.1, 127.2, 126.8, 126.1, 125.7, 125.6, 123.3, 113.0, 33.3, 23.4, 10.6, 10.2.

IR (Diamond-ATR, neat): $\tilde{\nu}$ / cm^{-1} = 3064, 2942, 2927, 2871, 1638, 1452, 1437, 1423, 1393, 1262, 1193, 886, 807, 790, 775, 758, 749, 733, 682.

MS (EI, 70 eV): *m/z* (%) = 208 (16), 142 (12), 141 (100), 115 (26).

HRMS (EI-orbitrap): *m/z:* [M] calc. for [$C_{17}H_{17}NO$]: 251.1310; found 251.1304.

m.p. (°C): 92.6 – 94.8.

1-(5-chloropentyl)naphthalene (13j)

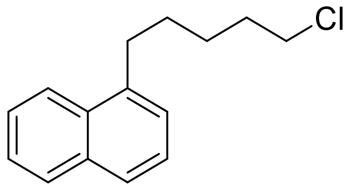

According to **TP4**, a solution of 3-(chloromethyl)heptane (**2**, 0.2 M, 2.0 equiv) in *n*-hexane was prepared. The solution of **2** was pumped through the activated sodium packed-bed reactor (see **TP1**) by pump A (flow rate: 2.0 mL/min) into the precooling loop (V_{pre2} = 0.35 mL) at 25 °C. Subsequently upon reaching the steady state, it was injected for 2 min into a flask charged with a solution of TMEDA (0.80 mmol, 2.0 equiv) and 1–methylnaphtalene (**3e**, 57 mg, 0.40 mmol, 1.0 equiv) in hexane (2.0 mL) the mixture was stirred for 30 min at 25 °C. Before it was cooled to –20 °C and a solution of 1-bromo-4-chlorobutane (**12i**, 137 mg, 0.80 mmol, 2.0 equiv) in THF (2.0 mL) was added. The reaction mixture was stirred at –20 °C for 10 min followed by another 30 min at 25 °C, before sat. *aq.* NH_4Cl solution was added for quenching the reaction mixture. The aqueous layer was extracted three times with EtOAc (3×30 mL) and the combined organic layers were dried over anhydrous $MgSO_4$ and filtrated. After removal of the solvent, flash column chromatographical purification (silica gel, pentane:EtOAc = 100:0 → 98:2) afforded the title compound **13j** as a colorless oil (68 mg, 0.29 mmol, 73% yield).

^{1}H-NMR (400 MHz, $CDCl_3$): δ / ppm = 8.04 (d, *J* = 8.5 Hz, 1H), 7.90 – 7.83 (m, 1H), 7.73 (d, *J* = 8.2 Hz, 1H), 7.56 – 7.46 (m, 2H), 7.44 – 7.38 (m, 1H), 7.33 (d, *J* = 5.8 Hz, 1H), 3.56 (t, *J* = 6.7 Hz, 2H), 3.10 (d, *J* = 15.5 Hz, 2H), 1.91 – 1.75 (m, 4H), 1.65 – 1.55 (m, 2H).

^{13}C-NMR (100 MHz, $CDCl_3$): δ / ppm = 138.5, 134.0, 132.0, 128.9, 126.7, 126.1, 125.9, 125.7, 125.6, 123.9, 45.2, 33.1, 32.7, 30.2, 27.2.

IR (Diamond-ATR, neat): $\tilde{\nu}$ / cm^{-1} = 3045, 2934, 2859, 1596, 1510, 1463, 1444, 1432, 1395, 1304, 1255, 1166, 797, 776, 730.

MS (EI, 70 eV): *m/z* (%) = 232 (20), 142 (11), 142 (12), 141 (100), 115 (18).

HRMS (EI-orbitrap): *m/z:* [M] calc. for [$C_{15}H_{17}Cl$]: 232.1019; found 232.1013.

(1-((1*R*,2*S*)-2-methoxycyclohexyl)ethyl)benzene (13k) mixture of diastereoisomeres

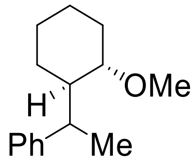

According to **TP3**, a solution of 3-(chloromethyl)heptane (**2**, 0.2 M, 2.0 equiv) in *n*-hexane was prepared. The solution of **2** was pumped through the activated sodium packed-bed reactor (see **TP1**) by pump A (flow rate: 2.0 mL/min) into the precooling loop (V_{pre2} = 0.35 mL) at 25 °C. Subsequently upon reaching the steady state, it was injected (2 min) into a flask charged with TMEDA (0.80 mmol, 2.0 equiv) in ethylbenzene (**3g**, 1.0 mL) and the resulting mixture was stirred at 25 °C for 30 min. The reaction flask was cooled to –20 °C and a solution of (1*R*,2*S*)-1-chloro-2-methoxycyclohexane (**12d**, 59 mg, 0.40 mmol, 1.0 equiv) in THF (1.0 mL) was added The reaction mixture was stirred at –20 °C for 10 min followed by another 30 min at 25 °C, before sat. *aq.* NH_4Cl solution was added for quenching the reaction mixture. The aqueous layer was extracted three times with EtOAc (3×30 mL) and the combined organic layers were dried over anhydrous $MgSO_4$ and filtrated. After removal of the solvent, flash column chromatographical purification (silica gel, pentane:EtOAc = 100:0 → 99:1) afforded the title compound **13k** as a slightly yellow oil (59 mg, 0.27 mmol, 68% yield, d.r. = 2.1:1.0).

Major:

^{1}H-NMR (400 MHz, $CDCl_3$): δ / ppm = 7.32 – 7.27 (m, 2H), 7.25 7.16 (m, 3H), 3.53 – 3.46 (m, 1H), 3.38 (s, 3H), 2.63 (td, *J* = 9.9, 4.1 Hz, 1H), 2.17 – 2.07 (m, 1H), 1.87 – 1.80 (m, 1H), 1.74 1.49 (m, 3H), 1.30 (d, *J* = 7.5 Hz, 3H), 1.21 – 0.96 (m, 3H), 0.79 – 0.65 (m, 1H).

^{13}C-NMR (100 MHz, $CDCl_3$): δ / ppm = 143.9, 129.0 (2C), 127.7 (2C), 125.9, 80.1, 55.2, 49.2, 37.5, 30.1, 25.7, 24.8, 24.3, 18.9.

Minor:

^{1}H-NMR (400 MHz, $CDCl_3$): δ / ppm = 7.32 – 7.27 (m, 2H), 7.25 7.16 (m, 3H), 3.44 – 3.39 (m, 1H), 3.39 (s, 3H), 3.02 – 2.95 (m, 1H), 2.17 – 2.07 (m, 1H), 1.74 1.49 (m, 4H), 1.22 (d, *J* = 7.2 Hz, 3H), 1.21 – 0.96 (m, 4H).

^{13}C-NMR (100 MHz, $CDCl_3$): δ / ppm = 146.6, 128.1 (2C), 128.0 (2C), 125.6, 80.6, 56.0, 49.2, 37.4, 30.3, 25.3, 24.6, 24.2, 13.6.

Mixture:

IR (Diamond-ATR, neat): $\tilde{\nu}$ / cm^{-1} = 2965, 2928, 2858, 2819, 1494, 1451, 1375, 1190, 1131, 1100, 958, 906, 769, 702.

MS (EI, 70 eV): *m/z* (%) =187 (15), 186 (100), 171 (49), 157 (25), 144 (42), 143 (49), 131 (11), 130 (15), 129 (57), 128 (16), 118 (48), 117 (27), 115 (22), 113 (26), 112 (15), 106 (30), 105 (67), 103 (14), 91 (45), 81 (40), 79 (27), 77 (13).

HRMS (EI-orbitrap): *m/z:* [M – CH_3OH] calc. for [$C_{14}H_{18}$]: 186.1409; found 186.1402.

1-(4-chlorobutyl)-1,2,3,4-tetrahydronaphthalene (13l)

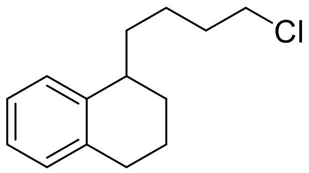

According to **TP4**, a solution of 3-(chloromethyl)heptane (**2**, 0.2 M, 2.0 equiv) in *n*-hexane was prepared. The solution of **2** was pumped through the activated sodium packed-bed reactor (see **TP1**) by pump A (flow rate: 2.0 mL/min) into the precooling loop (V_{pre2} = 0.35 mL) at 25 °C. Subsequently upon reaching the steady state, it was injected for 2 min into a flask charged with a solution of TMEDA (0.80 mmol, 2.0 equiv) and tetralin (**3h**, 53 mg, 0.40 mmol, 1.0 equiv) in hexane (2.0 mL) the mixture was stirred for 30 min at 25 °C. Before it was cooled to –20 °C and a solution of 1-bromo-4-chlorobutane (**12i**, 137 mg, 0.80 mmol, 2.0 equiv) in THF (2.0 mL) was added. The reaction mixture was stirred at –20 °C for 10 min followed by another 30 min at 25 °C, before sat. *aq.* NH_4Cl solution was added for quenching the reaction mixture. The aqueous layer was extracted three times with EtOAc (3×30 mL) and the combined organic layers were dried over anhydrous $MgSO_4$ and filtrated. After removal of the solvent, flash column chromatographical purification (silica gel, pentane:EtOAc = 100:0 → 98:2) afforded the title compound **13l** as a colorless oil (50 mg, 0.22 mmol, 56% yield).

^{1}H-NMR (400 MHz, $CDCl_3$): δ / ppm = 7.22 – 7.03 (m, 4H), 3.57 (td, *J* = 6.7, 1.2 Hz, 2H), 2.85 – 2.68 (m, 3H), 1.90 – 1.78 (m, 4H), 1.77 – 1.68 (m, 3H), 1.65 – 1.50 (m, 3H).

^{13}C-NMR (100 MHz, $CDCl_3$): δ / ppm = 141.3, 137.2, 129.2, 128.6, 125.6, 125.6, 45.2, 37.6, 36.3, 33.0, 29.8, 27.4, 24.8, 19.8.

IR (Diamond-ATR, neat): $\tilde{\nu}$ / cm^{-1} = 2933, 2860, 1490, 1450, 759, 742.

MS (EI, 70 eV): *m/z* (%) = 132 (10), 131 (100), 129 (41), 128 (33), 116 (12), 115 (37), 91 (27).

HRMS (EI-orbitrap): *m/z:* [M] calc. for [$C_{14}H_{19}Cl$]: 222.1175; found 222.1170.

(3-(4-methoxyphenyl)propane-1,1-diyl)dibenzene (13m)

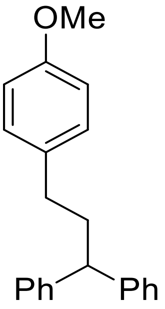

According to **TP3**, a solution of 3-(chloromethyl)heptane (**2**, 0.2 M, 2.0 equiv) in *n*-hexane was prepared. The solution of **2** was pumped through the activated sodium packed-bed reactor (see **TP1**) by pump A (flow rate: 2.0 mL/min) into the precooling loop (V_{pre2} = 0.35 mL) at 25 °C. Subsequently upon reaching the steady state, it was injected (2 min) into a flask charged with TMEDA (0.80 mmol, 2.0 equiv) in diphenylmethane (**3n**, 1.0 mL) and the resulting mixture was stirred at 25 °C for 30 min. The reaction flask was cooled to –20 °C and a solution of 1-(2-chloroethyl)-4-methoxybenzene (**12j**, 68 mg, 0.40 mmol, 1.0 equiv) in THF (1.0 mL) was added. The reaction mixture was stirred at –20 °C for 10 min followed by another 30 min at 25 °C, before sat. *aq.* NH_4Cl solution was added for quenching the reaction mixture. The aqueous layer was extracted three times with EtOAc (3×30 mL) and the combined organic layers were dried over anhydrous $MgSO_4$ and filtrated. After removal of the solvent, flash column chromatographical purification (silica gel, pentane:EtOAc = 100:0 → 98:2) afforded the title compound **13m** as a colorless oil (85 mg, 0.28 mmol, 70% yield).

^{1}H-NMR (400 MHz, $CDCl_3$): δ / ppm = 7.34 – 7.30 (m, 4H), 7.30 – 7.26 (m, 4H), 7.23 – 7.18 (m, 2H), 7.10 – 7.06 (m, 2H), 6.87 – 6.83 (m, 2H), 3.94 (t, *J* = 7.7 Hz, 1H), 3.81 (s, 3H), 2.55 (dd, *J* = 9.2, 6.4 Hz, 2H), 2.43 – 2.32 (m, 2H).

^{13}C-NMR (100 MHz, $CDCl_3$): δ / ppm = 157.8, 145.0 (2C), 134.2, 129.5 (2C), 128.6 (4C), 128.0 (4C), 126.3 (2C), 113.8 (2C), 55.4, 50.7, 37.6, 33.2.

IR (Diamond-ATR, neat): $\tilde{\nu}$ / cm^{-1} = 3060, 3026, 2932, 1612, 1600, 1512, 1494, 1464, 1449, 1300, 1245, 1178, 1033, 830, 747, 700.

MS (EI, 70 eV): *m/z* (%) = 302 (15), 168 (18), 168 (13), 167 (92), 166 (11), 165 (57), 152 (33), 135 (100), 122 (13), 121 (47), 105 (15), 91 (14), 77 (10).

HRMS (EI-orbitrap): *m/z:* [M] calc. for [$C_{22}H_{22}O$]: 302.1671; found 302.1665.

2-(2,2-diphenylethyl)tetrahydrofuran (13n)

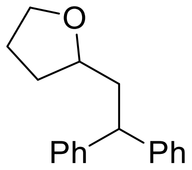

According to **TP3**, a solution of 3-(chloromethyl)heptane (**2**, 0.2 M, 2.0 equiv) in *n*-hexane was prepared. The solution of **2** was pumped through the activated sodium packed-bed reactor (see **TP1**) by pump A (flow rate: 2.0 mL/min) into the precooling loop (V_{pre2} = 0.35 mL) at 25 °C. Subsequently upon reaching the steady state, it was injected (2 min) into a flask charged with TMEDA (0.80 mmol, 2.0 equiv) in diphenylmethane (**3n**, 1.0 mL) and the resulting mixture was stirred at 25 °C for 30 min. The reaction flask was cooled to –20 °C and a solution of 2-(chloromethyl)tetrahydrofuran (**12k**, 48 mg, 0.40 mmol, 1.0 equiv) in THF (1.0 mL) was added. The reaction mixture was stirred at –20 °C for 10 min followed by another 30 min at 25 °C, before sat. *aq.* NH_4Cl solution was added for quenching the reaction mixture. The aqueous layer was extracted three times with EtOAc (3×30 mL) and the combined organic layers were dried over anhydrous $MgSO_4$ and filtrated. After removal of the solvent, flash column chromatographical purification (silica gel, pentane:EtOAc = 100:0 → 98:2) afforded the title compound **13n** as a colorless solid (48 mg, 0.19 mmol, 48% yield).

^{1}H-NMR (400 MHz, $CDCl_3$): δ / ppm = 7.29 – 7.25 (m, 8H), 7.20 – 7.15 (m, 2H), 4.15 (dd, *J* = 9.5, 6.3 Hz, 1H), 3.90 – 3.82 (m, 1H), 3.74 – 3.59 (m, 2H), 2.35 – 2.25 (m, 1H), 2.24 – 2.15 (m, 1H), 1.91 (dd, *J* = 37.0, 19.6 Hz, 2H), 1.85 – 1.74 (m, 1H), 1.54 – 1.41 (m, 1H).

^{13}C-NMR (100 MHz, $CDCl_3$): δ / ppm = 145.2, 144.6, 128.6 (2C), 128.6 (2C), 128.2 (2C), 127.9 (2C), 126.3, 126.2, 77.0, 67.6, 48.3, 41.8, 31.6, 25.8.

IR (Diamond-ATR, neat): $\tilde{\nu}$ / cm^{-1} = 3085, 3061, 3026, 3001, 2969, 2936, 2865, 2365, 2359, 2343, 2336, 1600, 1494, 1450, 1362, 1114, 1082, 1059, 1031, 1016, 786, 752, 739, 700.

MS (EI, 70 eV): *m/z* (%) = 205 (16), 178 (12), 168 (19), 168 (14), 167 (100), 165 (60), 152 (28), 115 (17).

HRMS (EI-orbitrap): *m/z:* [M] calc. for [$C_{18}H_{20}O$]: 252.1514; found 252.1507.

m.p. (°C): 57.0 – 59.6.

fenpiprane (7)

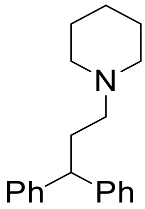

According to **TP4**, a solution of 3-(chloromethyl)heptane (**2**, 0.2 M, 2.0 equiv) in *n*-hexane was prepared. The solution of **2** was pumped through the activated sodium packed-bed reactor (see **TP1**) by pump A (flow rate: 2.0 mL/min) into the precooling loop (V_{pre2} = 0.35 mL) at 25 °C. Subsequently upon reaching the steady state, it was injected for 2 min into a flask charged with a solution of TMEDA (0.80 mmol, 2.0 equiv) and diphenylmethane (**3n**, 67 mg, 0.40 mmol, 1.0 equiv) in hexane (2.0 mL) the mixture was stirred for 30 min at 25 °C. The reaction mixture was cooled to –20 °C and a solution of 1-(2-chloroethyl)piperidine (**12l**, 118 mg, 0.80 mmol, 2.0 equiv) in THF (2.0 mL) was added. The reaction mixture was stirred at –20 °C for 10 min followed by another 30 min at 25 °C, before sat. *aq.* NH_4Cl solution was added for quenching the reaction mixture. The aqueous layer was extracted three times with EtOAc (3×30 mL) and the combined organic layers were dried over anhydrous $MgSO_4$ and filtrated. After removal of the solvent, flash column chromatographical purification (silica gel, pentane:EtOAc = 50:50 → 30:70) afforded the title compound **7** as a colorless oil (57 mg, 0.20 mmol, 51% yield).

^{1}H-NMR (400 MHz, $CDCl_3$): δ / ppm = 7.31 – 7.22 (m, 8H), 7.19 – 7.12 (m, 2H), 4.00 – 3.90 (m, 1H), 2.46 – 2.20 (m, 8H), 1.57 (q, *J* = 5.6 Hz, 4H), 1.42 (q, *J* = 5.9 Hz, 2H).

^{13}C-NMR (100 MHz, $CDCl_3$): δ / ppm = 145.1 (2C), 128.5 (4C), 128.0 (4C), 126.2 (2C), 57.9, 54.8 (2C), 49.5, 32.9, 26.1 (2C), 24.6.

IR (Diamond-ATR, neat): $\tilde{\nu}$ / cm^{-1} = 3026, 2933, 2852, 2801, 2767, 1494, 1450, 1118, 762, 748, 700.

MS (EI, 70 eV): *m/z* (%) = 180 (13), 179 (13), 178 (22), 165 (41), 152 (13), 115 (10), 98 (100), 96 (10).

HRMS (EI-orbitrap): *m/z:* [M] calc. for [$C_{20}H_{25}N$]: 279.1987; found 279.1983.

(((1*R*,2*S*)-2-methoxycyclohexyl)methylene)dibenzene (13o)

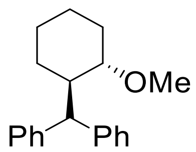

According to **TP4**, a solution of 3-(chloromethyl)heptane (**2**, 0.2 M, 2.0 equiv) in *n*-hexane was prepared. The solution of **2** was pumped through the activated sodium packed-bed reactor (see **TP1**) by pump A (flow rate: 2.0 mL/min) into the precooling loop (V_{pre2} = 0.35 mL) at 25 °C. Subsequently upon reaching the steady state, it was injected for 2 min into a flask charged with a solution of TMEDA (0.80 mmol, 2.0 equiv) and diphenylmethane (**3n**, 67 mg, 0.40 mmol, 1.0 equiv) in hexane (2.0 mL) the mixture was stirred for 30 min at 25 °C. Before it was cooled to –20 °C and a solution of (1*R*,2*S*)-1-chloro-2-methoxycyclohexane (**12d**, 118 mg, 0.80 mmol, 2.0 equiv) in THF (2.0 mL) was added. The reaction mixture was stirred at –20 °C for 10 min followed by another 30 min at 25 °C, before sat. *aq.* NH_4Cl solution was added for quenching the reaction mixture. The aqueous layer was extracted three times with EtOAc (3×30 mL) and the combined organic layers were dried over anhydrous $MgSO_4$ and filtrated. After removal of the solvent, flash column chromatographical purification (silica gel, pentane:EtOAc = 100:0 → 98:2) afforded the title compound **13o** as a slightly yellow solid (85 mg, 0.30 mmol, 76% yield).

^{1}H-NMR (400 MHz, $CDCl_3$): δ / ppm = 7.33 – 7.23 (m, 8H), 7.22 – 7.12 (m, 2H), 4.38 (d, *J* = 7.8 Hz, 1H), 3.24 (s, 3H), 2.87 (td, *J* = 7.1, 3.4 Hz, 1H), 2.55 – 2.42 (m, 1H), 1.97 – 1.85 (m, 1H), 1.85 – 1.74 (m, 1H), 1.71 – 1.50 (m, 2H), 1.46 – 1.35 (m, 1H), 1.33 – 1.19 (m, 2H), 1.13 – 1.01 (m, 1H).

^{13}C-NMR (100 MHz, $CDCl_3$): δ / ppm = 144.2, 143.2, 129.6 (2C), 128.4 (2C), 128.3 (2C), 128.2 (2C), 126.2, 125.9, 79.0, 55.7, 50.9, 44.0, 27.9, 25.3, 23.5, 22.8.

IR (Diamond-ATR, neat): $\tilde{\nu}$ / cm^{-1} = 3086, 3026, 2970, 2932, 2858, 2820, 2359, 2343, 2326, 1740, 1736, 1719, 1601, 1495, 1450, 1375, 1227, 1217, 1198, 1191, 1159, 1123, 1097, 1032, 754, 745, 730, 703.

MS (EI, 70 eV): *m/z* (%) = 179 (10), 178 (11), 167 (46), 166 (13), 166 (13), 165 (100), 164 (10), 152 (38), 115 (22), 91 (12), 81 (10), 79 (10).

HRMS (EI-orbitrap): *m/z:* [M – $HOCH_3$] calc. for [$C_{19}H_{20}$]: 248.1565; found 248.1559.

m.p. (°C): 95.4 – 98.9.

***cis*-4-(tert-butyl)cyclohexyl)methylene)dibenzene (13p)**

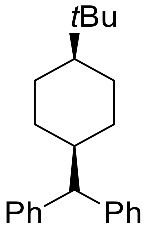

According to **TP4**, a solution of 3-(chloromethyl)heptane (**2**, 0.2 M, 2.0 equiv) in *n*-hexane was prepared. The solution of **2** was pumped through the activated sodium packed-bed reactor (see **TP1**) by pump A (flow rate: 2.0 mL/min) into the precooling loop (V_{pre2} = 0.35 mL) at 25 °C. Subsequently upon reaching the steady state, it was injected for 2 min into a flask charged with a solution of TMEDA (0.80 mmol, 2.0 equiv) and diphenylmethane (**3n**, 67 mg, 0.40 mmol, 1.0 equiv) in hexane (2.0 mL) the mixture was stirred for 30 min at 25 °C. The reaction mixture was cooled to –20 °C and a solution of *cis*-4-(tert-butyl)cyclohexyl 4-methylbenzenesulfonate (**12m**, 248 mg, 0.80 mmol, 2.0 equiv) in THF (2.0 mL) was added. The reaction mixture was stirred at –20 °C for 10 min followed by another 30 min at 25 °C, before sat. *aq.* NH_4Cl solution was added for quenching the reaction mixture. The aqueous layer was extracted three times with EtOAc (3×30 mL) and the combined organic layers were dried over anhydrous $MgSO_4$ and filtrated. After removal of the solvent, flash column chromatographical purification (silica gel, pentane:EtOAc = 100:0 → 98:2) afforded the title compound **13p** as a colorless solid (67 mg, 0.22 mmol, 55% yield).

^{1}H-NMR (400 MHz, $CDCl_3$): δ / ppm = 7.37 – 7.30 (m, 4H), 7.29 – 7.23 (m, 4H), 7.14 (t, *J* = 7.3 Hz, 2H), 4.05 (d, *J* = 12.3 Hz, 1H), 2.57 (d, *J* = 10.1 Hz, 1H), 1.63 – 1.51 (m, 2H), 1.48 – 1.33 (m, 4H), 1.31 – 1.20 (m, 2H), 1.09 – 0.97 (m, 1H), 0.88 (s, 9H).

^{13}C-NMR (100 MHz, $CDCl_3$): δ / ppm = 144.8 (2C), 128.6 (4C), 128.2 (4C), 126.1 (2C), 51.6, 48.3, 36.2, 32.8, 28.5 (2C), 27.7 (3C), 21.5 (2C).

IR (Diamond-ATR, neat): $\tilde{\nu}$ / cm^{-1} = 3026, 2962, 2954, 2928, 2858, 1597, 1584, 1491, 1474, 1466, 1449, 1390, 1373, 1360, 1177, 1074, 1031, 911, 854, 750, 741, 692.

MS (EI, 70 eV): *m/z* (%) = 168 (57), 168 (14), 167 (100), 165 (38), 152 (18), 83 (17).

HRMS (EI-orbitrap): *m/z:* [M] calc. for [$C_{23}H_{30}$]: 306.2348; found 306.2341.

m.p. (°C): 120.5 – 133.8.

***cis*-4-benzhydrylcyclohexyl)oxy)(tert-butyl)dimethylsilane (13q)**

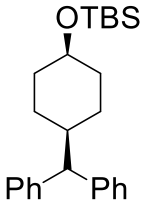

According to **TP3**, a solution of 3-(chloromethyl)heptane (**2**, 0.2 M, 2.0 equiv) in *n*-hexane was prepared. The solution of **2** was pumped through the activated sodium packed-bed reactor (see **TP1**) by pump A (flow rate: 2.0 mL/min) into the precooling loop (V_{pre2} = 0.35 mL) at 25 °C. Subsequently upon reaching the steady state, it was injected (2 min) into a flask charged with TMEDA (0.80 mmol, 2.0 equiv) in diphenylmethane (**3n**, 1.0 mL) and the resulting mixture was stirred at 25 °C for 30 min. The reaction flask was cooled to –20 °C and a solution of *trans*-4-((tert-butyldimethylsilyl)oxy)cyclohexyl 4-methylbenzenesulfonate (**12n**, 154 mg, 0.40 mmol, 1.0 equiv) in THF (1.0 mL) was added The reaction mixture was stirred at –20 °C for 10 min followed by another 30 min at 25 °C, before sat. *aq.* NH_4Cl solution was added for quenching the reaction mixture. The aqueous layer was extracted three times with EtOAc (3×30 mL) and the combined organic layers were dried over anhydrous $MgSO_4$ and filtrated. After removal of the solvent, flash column chromatographical purification (silica gel, pentane:EtOAc = 100:0 → 98:2) afforded the title compound **13q** as a colorless oil (83 mg, 0.22 mmol, 55% yield).

^{1}H-NMR (400 MHz, $CDCl_3$): δ / ppm = 7.37 – 7.32 (m, 4H), 7.31 – 7.26 (m, 4H), 7.19 – 7.13 (m, 2H), 3.95 (tt, *J* = 4.7, 2.1 Hz, 1H), 3.65 (d, *J* = 11.2 Hz, 1H), 2.27 – 2.10 (m, 1H), 1.71 – 1.59 (m, 2H), 1.51 – 1.31 (m, 6H), 0.93 (s, 9H), 0.05 (s, 6H).

^{13}C-NMR (100 MHz, $CDCl_3$): δ / ppm = 144.6 (2C), 128.5 (4C), 128.2 (4C), 126.1 (2C), 67.2, 58.4, 40.4, 33.3 (2C), 26.0 (3C), 25.9 (2C), 18.3, -4.7 (2C).

IR (Diamond-ATR, neat): $\tilde{\nu}$ / cm^{-1} = 3085, 3062, 3027, 2928, 2884, 2856, 1597, 1494, 1472, 1462, 1450, 1406, 1376, 1360, 1251, 1198, 1180, 1156, 1110, 1087, 1049, 1022, 1006, 939, 912, 886, 833, 807, 773, 752, 744, 702, 676.

MS (EI, 70 eV): *m/z* (%) = 323 (37), 247 (14), 169 (17), 168 (14), 167 (100), 165 (30), 152 (17), 143 (19), 75 (42).

HRMS (EI-orbitrap): *m/z:* [M – CH_3] calc. for [$C_{24}H_{33}OSi$] 365.2301; found 365.2290.

2-((5-methylthiazol-2-yl)methyl)adamantan-2-ol (15a)

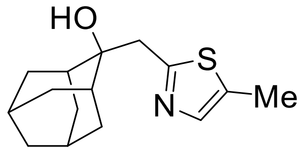

According to **TP4**, a solution of 3-(chloromethyl)heptane (**2**, 0.2 M, 2.0 equiv) in *n*-hexane was prepared. The solution of **2** was pumped through the activated sodium packed-bed reactor (see **TP1**) by pump A (flow rate: 2.0 mL/min) into the precooling loop (V_{pre2} = 0.35 mL) at 25 °C. Subsequently upon reaching the steady state, it was injected for 2 min into a flask charged with a solution of TMEDA (0.80 mmol, 2.0 equiv) and 2,4-dimethylthiazole (**14a**, 45 mg, 0.40 mmol, 1.0 equiv) in hexane (2.0 mL) the mixture was stirred for 1 h at –40 °C. Before a solution solution of adamantanone (**9k**, 120 mg, 0.80 mmol, 2.0 equiv) in THF (2.0 mL) was added. The reaction mixture was stirred at –40 °C for 10 min followed by another 30 min at 25 °C, before sat. *aq.* NH_4Cl solution was added for quenching the reaction mixture. The aqueous layer was extracted three times with EtOAc (3×30 mL) and the combined organic layers were dried over anhydrous $MgSO_4$ and filtrated. After removal of the solvent, flash column chromatographical purification (silica gel, pentane:EtOAc = 90:10 → 80:20) afforded the title compound **15a** as a colorless solid (75 mg, 0.28 mmol, 71% yield).

^{1}H-NMR (400 MHz, $CDCl_3$): δ / ppm = 6.70 (d, *J* = 1.0 Hz, 1H), 4.55 (s, 1H), 3.29 (s, 2H), 2.41 (d, *J* = 1.0 Hz, 3H), 2.28 (d, *J* = 9.9 Hz, 2H), 1.94 – 1.77 (m, 4H), 1.75 – 1.60 (m, 6H), 1.49 (d, *J* = 12.6 Hz, 2H).

^{13}C-NMR (100 MHz, $CDCl_3$): δ / ppm = 166.8, 152.7, 112.3, 75.3, 40.5, 38.4, 37.0 (2C), 34.7 (2C), 32.9 (2C), 27.4, 27.4, 17.2.

IR (Diamond-ATR, neat): $\tilde{\nu}$ / cm^{-1} = 3376, 3092, 2949, 2936, 2914, 2902, 2889, 2854, 1532, 1524, 1473, 1455, 1442, 1418, 1370, 1356, 1352, 1328, 1311, 1304, 1162, 1154, 1129, 1109, 1098, 1064, 1050, 1041, 1026, 1014, 1000, 994, 974, 929, 891, 864, 855, 773, 747, 668.

MS (EI, 70 eV): *m/z* (%) = 151 (18), 113 (100).

HRMS (EI-orbitrap): *m/z:* [M – H] calc. for [$C_{15}H_{20}NOS$]: 262.1266; found 262.1263.

m.p. (°C): 112.9 – 115.7.

***trans*-2-((4-methylthiazol-2-yl)methyl)cyclohexan-1-ol (15b)**

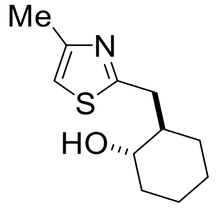

According to **TP4**, a solution of 3-(chloromethyl)heptane (**2**, 0.2 M, 2.0 equiv) in *n*-hexane was prepared. The solution of **2** was pumped through the activated sodium packed-bed reactor (see **TP1**) by pump A (flow rate: 2.0 mL/min) into the precooling loop (V_{pre2} = 0.35 mL) at 25 °C. Subsequently upon reaching the steady state, it was injected for 2 min into a flask charged with a solution of TMEDA (0.80 mmol, 2.0 equiv) and 2,4-dimethylthiazole (**14a**, 45 mg, 0.40 mmol, 1.0 equiv) in hexane (2.0 mL) the mixture was stirred for 1 h at 25 °C. Before the mixture was cooled to –40 °C and a solution of cyclohexene oxide (**9a**, 79 mg, 0.80 mmol, 2.0 equiv) in THF (2.0 mL) was added. The reaction mixture was stirred at –40 °C for 10 min followed by another 1 h at 25 °C, before sat. *aq.* NH_4Cl solution was added for quenching the reaction mixture. The aqueous layer was extracted three times with EtOAc (3×30 mL) and the combined organic layers were dried over anhydrous $MgSO_4$ and filtrated. After removal of the solvent, flash column chromatographical purification (silica gel, pentane:EtOAc = 30:70 → 10:90) afforded the title compound **15b** as a colorless oil (36 mg, 0.17 mmol, 43% yield).

^{1}H-NMR (400 MHz, $CDCl_3$): δ / ppm = 6.68 (s, 1H), 4.06 (s, 1H), 3.33 – 3.16 (m, 2H), 2.95 (dd, *J* = 14.9, 6.2 Hz, 1H), 2.38 (d, *J* = 0.9 Hz, 3H), 2.02 – 1.95 (m, 1H), 1.76 – 1.66 (m, 3H), 1.65 – 1.58 (m, 1H), 1.33 – 1.03 (m, 4H).

^{13}C-NMR (100 MHz, $CDCl_3$): δ / ppm = 169.5, 152.2, 112.7, 74.5, 45.8, 37.4, 35.5, 31.5, 25.7, 24.9, 17.1.

IR (Diamond-ATR, neat): $\tilde{\nu}$ / cm^{-1} = 3324, 2924, 2854, 1531, 1462, 1446, 1304, 1132, 1093, 1070, 1060, 1032, 730.

MS (EI, 70 eV): *m/z* (%) = 211 (25), 192 (13), 183 (27), 182 (20), 168 (20), 140 (64), 138 (10), 127 (19), 126 (100), 115 (20), 114 (31), 112 (25), 73 (19), 72 (50), 71 (45), 68 (11), 67 (13), 55 (12), 53 (13), 45 (32), 44 (24), 43 (65), 42 (11), 41 (32).

HRMS (EI-orbitrap): *m/z:* [M] calc. for [$C_{11}H_{17}NOS$]: 211.1031; found 211.1031.

(*S*)-1-(benzyloxy)-4-(4-methylthiazol-2-yl)butan-2-ol (15c)

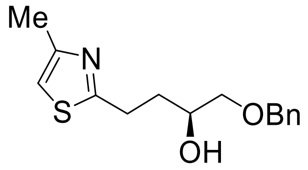

According to **TP4**, a solution of 3-(chloromethyl)heptane (**2**, 0.2 M, 2.0 equiv) in *n*-hexane was prepared. The solution of **2** was pumped through the activated sodium packed-bed reactor (see **TP1**) by pump A (flow rate: 2.0 mL/min) into the precooling loop (V_{pre2} = 0.35 mL) at 25 °C. Subsequently upon reaching the steady state, it was injected for 2 min into a flask charged with a solution of TMEDA (0.80 mmol, 2.0 equiv) and 2,4-dimethylthiazole (**14a**, 45 mg, 0.40 mmol, 1.0 equiv) in hexane (2.0 mL) the mixture was stirred for 1 h at 25 °C. Before the mixture was cooled to –40°C and a solution of (S)-2-((benzyloxy)methyl)oxirane (**9m**, 131 mg, 0.80 mmol, 2.0 equiv) in THF (2.0 mL) was added. The reaction mixture was stirred at –40 °C for 10 min followed by another 30 min at 25 °C, before sat. *aq.* NH_4Cl solution was added for quenching the reaction mixture. The aqueous layer was extracted three times with EtOAc (3×30 mL) and the combined organic layers were dried over anhydrous $MgSO_4$ and filtrated. After removal of the solvent, flash column chromatographical purification (silica gel, pentane:EtOAc = 30:70 → 10:90) afforded the title compound **15c** as a colorless oil (54 mg, 0.19 mmol, 49% yield).

^{1}H-NMR (400 MHz, $CDCl_3$): δ / ppm = 7.38 – 7.26 (m, 5H), 6.70 (d, *J* = 1.0 Hz, 1H), 4.54 (s, 2H), 3.94 – 3.84 (m, 1H), 3.61 (s, 1H), 3.51 – 3.47 (m, 1H), 3.46 – 3.38 (m, 1H), 3.16 – 3.09 (m, 2H), 2.39 (d, *J* = 0.9 Hz, 3H), 2.03 – 1.83 (m, 2H).

^{13}C-NMR (100 MHz, $CDCl_3$): δ / ppm = 170.2, 152.2, 138.0, 128.5 (2C), 127.8 (3C), 112.6, 74.4, 73.5, 69.6, 33.0, 29.7, 17.0.

IR (Diamond-ATR, neat): $\tilde{\nu}$ / cm^{-1} = 3339, 2921, 2857, 1531, 1452, 1445, 1364, 1303, 1203, 1088, 1075, 1028, 968, 734, 697.

MS (EI, 70 eV): *m/z* (%) = 171 (10), 168 (11), 156 (75), 127 (10), 126 (72), 111 (33), 91 (100), 72 (14), 71 (17), 65 (14), 45 (12), 43 (14).

HRMS (EI-orbitrap): *m/z:* [M] calc. for [$C_{15}H_{19}NO_2S$]: 277.1136; found 277.1129.

2-(2-isopropyl-4-methylthiazol-5-yl)adamantan-2-ol (17a)

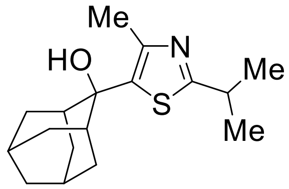

According to **TP4**, a solution of 3-(chloromethyl)heptane (**2**, 0.2 M, 2.0 equiv) in *n*-hexane was prepared. The solution of **2** was pumped through the activated sodium packed-bed reactor (see **TP1**) by pump A (flow rate: 2.0 mL/min) into the precooling loop (V_{pre2} = 0.35 mL) at 25 °C. Subsequently upon reaching the steady state, it was injected for 2 min into a flask charged with a solution of TMEDA (0.80 mmol, 2.0 equiv) and 2-isopropyl-4-methylthiazole (**14b**, 56 mg, 0.40 mmol, 1.0 equiv) in hexane (2.0 mL) the mixture was stirred for 1 h at –40 °C. Before a solution of adamantanone (**9k**, 120 mg, 0.80 mmol, 2.0 equiv) in THF (2.0 mL) was added. The reaction mixture was stirred at –40 °C for 10 min followed by another 1 h at 25 °C, before sat. *aq.* NH_4Cl solution was added for quenching the reaction mixture. The aqueous layer was extracted three times with EtOAc (3×30 mL) and the combined organic layers were dried over anhydrous $MgSO_4$ and filtrated. After removal of the solvent, flash column chromatographical purification (silica gel, pentane:EtOAc = 80:20 → 70:30) afforded the title compound **17a** as a colorless solid (85 mg, 0.29 mmol, 73% yield).

^{1}H-NMR (400 MHz, $CDCl_3$): δ / ppm = 3.17 (hept, *J* = 6.9 Hz, 1H), 2.47 (s, 3H), 2.42 (s, 2H), 2.36 (d, *J* = 13.0 Hz, 2H), 1.88 (d, *J* = 8.5 Hz, 3H), 1.85 – 1.74 (m, 4H), 1.71 (s, 2H), 1.64 (d, *J* = 12.2 Hz, 2H), 1.34 (s, 3H), 1.33 (s, 3H).

^{13}C-NMR (100 MHz, $CDCl_3$): δ / ppm = 173.9, 147.6, 136.9, 75.6, 38.6 (2C), 37.7, 35.3 (2C), 33.2, 33.0 (2C), 27.2, 26.7, 23.3 (2C), 18.1.

IR (Diamond-ATR, neat): $\tilde{\nu}$ / cm^{-1} = 3404, 2961, 2950, 2925, 2900, 2890, 2854, 1521, 1470, 1447, 1385, 1376, 1362, 1350, 1334, 1308, 1285, 1218, 1196, 1104, 1076, 1044, 1027, 1006, 962, 932, 898, 879, 841, 831, 672.

MS (EI, 70 eV): *m/z* (%) = 291 (42), 281 (12), 274 (27), 273 (22), 233 (15), 207 (29), 170 (39), 168 (100), 161 (86), 142 (15), 142 (35), 140 (14), 119 (14),117 (15), 93 (14), 91 (21), 79 (16), 77 (16), 72 (44), 69 (13), 57 (32), 55 (20), 52 (30), 48 (22), 48 21), 48 (72).

HRMS (EI-orbitrap): *m/z:* [M] calc. for [$C_{17}H_{25}NOS$]: 291.1657; found 291.1653.

m.p. (°C): 115.8 – 117.6.

***trans*-2-(2-(4-methylthiazol-2-yl)propan-2-yl)cyclohexan-1-ol (19a)**

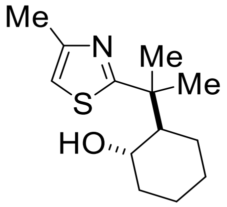

According to **TP4**, a solution of 3-(chloromethyl)heptane (**2**, 0.2 M, 2.0 equiv) in *n*-hexane was prepared. The solution of **2** was pumped through the activated sodium packed-bed reactor (see **TP1**) by pump A (flow rate: 2.0 mL/min) into the precooling loop (V_{pre2} = 0.35 mL) at 25 °C. Subsequently upon reaching the steady state, it was injected for 2 min into a flask charged with a solution of TMEDA (0.80 mmol, 2.0 equiv) and 2-isopropyl-4-methylthiazole (**14b**, 56 mg, 0.40 mmol, 1.0 equiv) in hexane (2.0 mL) the mixture was stirred for 1 h at –40 °C. Before a solution cyclohexene oxide (**9a**, 79 mg, 0.80 mmol, 2.0 equiv) in THF (2.0 mL) was added. The reaction mixture was stirred at –40 °C for 10 min followed by another 1 h at 25 °C, before sat. *aq.* NH_4Cl solution was added for quenching the reaction mixture. The aqueous layer was extracted three times with EtOAc (3×30 mL) and the combined organic layers were dried over anhydrous $MgSO_4$ and filtrated. After removal of the solvent, flash column chromatographical purification (silica gel, pentane:EtOAc = 80:20 → 70:30) afforded the title compound **19a** as a colorless solid (91 mg, 0.38 mmol, 95% yield).

^{1}H-NMR (400 MHz, $CDCl_3$): δ / ppm = 6.72 (s, 1H), 3.72 (s, 1H), 3.31 – 3.17 (m, 1H), 2.40 (s, 3H), 1.95 – 1.78 (m, 3H), 1.69 (d, *J* = 9.0 Hz, 2H), 1.48 (s, 3H), 1.45 (s, 3H), 1.33 – 1.14 (m, 3H), 1.03 – 0.91 (m, 1H).
^{13}C-NMR (100 MHz, $CDCl_3$): δ / ppm = 182.2, 151.7, 112.3, 73.7, 53.9, 42.9, 36.4, 30.6, 27.0, 26.4, 25.5, 25.2, 17.2.
IR (Diamond-ATR, neat): $\tilde{\nu}$ / cm^{-1} = 3276, 2940, 2919, 2851, 1530, 1458, 1447, 1385, 1366, 1351, 1342, 1326, 1300, 1289, 1193, 1121, 1097, 1067, 1034, 1008, 998, 863, 724, 687.
MS (EI, 70 eV): *m/z* (%) = 161 (28), 154 (32), 142 (12), 141 (83), 140 (100), 126 (17), 81 (11), 72 (25), 61 (11), 59 (12), 57 (15), 55 (14), 45 (10), 43 (12), 43 (66), 41 (20).
HRMS (EI-orbitrap): *m/z:* [M] calc. for [$C_{13}H_{21}NOS$]: 239.1344; found 239.1354.
m.p. (°C): 93.5 – 95.8.

4-methyl-2-(2-methyltetradecan-2-yl)thiazole (19b)

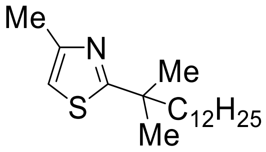

According to **TP4**, a solution of 3-(chloromethyl)heptane (**2**, 0.2 M, 2.0 equiv) in *n*-hexane was prepared. The solution of **2** was pumped through the activated sodium packed-bed reactor (see **TP1**) by pump A (flow rate: 2.0 mL/min) into the precooling loop (V_{pre2} = 0.35 mL) at 25 °C. Subsequently upon reaching the steady state, it was injected for 2 min into a flask charged with a solution of TMEDA (0.80 mmol, 2.0 equiv) and 2-isopropyl-4-methylthiazole (**14b**, 56 mg, 0.40 mmol, 1.0 equiv) in hexane (2.0 mL) the mixture was stirred for 1 h at –40 °C. Before a solution of 1-chlorododecane (**12a**, 164 mg, 0.80 mmol, 2.0 equiv) in THF (2.0 mL) was added. The reaction mixture was stirred at –40 °C for 10 min followed by another 16 h at 25 °C, before sat. *aq.* NH_4Cl solution was added for quenching the reaction mixture. The aqueous layer was extracted three times with EtOAc (3×30 mL) and the combined organic layers were dried over anhydrous $MgSO_4$ and filtrated. After removal of the solvent, flash column chromatographical purification (silica gel, pentane:EtOAc = 100:0 → 97:3) afforded the title compound **19b** as a colorless oil (109 mg, 0.35 mmol, 88% yield).

^{1}H-NMR (400 MHz, $CDCl_3$): δ / ppm = 6.69 (q, *J* = 1.0 Hz, 1H), 2.42 (d, *J* = 1.1 Hz, 3H), 1.73 – 1.64 (m, 2H), 1.39 (s, 6H), 1.29 – 1.15 (m, 20H), 0.90 – 0.84 (m, 3H).

^{13}C-NMR (100 MHz, $CDCl_3$): δ / ppm = 180.3, 152.1, 111.9, 44.4, 40.7, 32.1, 30.3, 29.8, 29.8 (2C), 29.8, 29.7, 29.5, 28.7 (2C), 24.7, 22.8, 17.5, 14.3.

IR (Diamond-ATR, neat): $\tilde{\nu}$ / cm^{-1} = 2957, 2922, 2853, 1530, 1478, 1458, 1384, 1375, 1363, 1304, 1259, 1210, 1140, 1121, 1050, 960, 862, 725, 702, 668.

MS (EI, 70 eV): *m/z* (%) = 281 (28), 225 (35), 209 (18), 207 (69), 191 (14), 141 (100), 140 (61), 126 (25).

HRMS (EI-orbitrap): *m/z:* [M] calc. for [$C_{19}H_{35}NS$]: 309.2490; found 309.2488.

2-(pyridin-2-ylmethyl)adamantan-2-ol (22a)

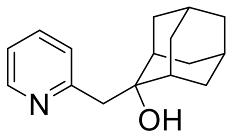

According to **TP4**, a solution of 3-(chloromethyl)heptane (**2**, 0.2 M, 2.0 equiv) in *n*-hexane was prepared. The solution of **2** was pumped through the activated sodium packed-bed reactor (see **TP1**) by pump A (flow rate: 2.0 mL/min) into the precooling loop (V_{pre2} = 0.35 mL) at 25 °C. Subsequently upon reaching the steady state, it was injected for 2 min into a flask charged with a solution of TMEDA (0.80 mmol, 2.0 equiv) and 2-picoline (**20a**, 37 mg, 0.40 mmol, 1.0 equiv) in hexane (2.0 mL) the mixture was stirred for 1 h at 25 °C. Before the mixture was cooled to –40°C and a solution of adamantanone (**9k**, 120 mg, 0.80 mmol, 2.0 equiv) in THF (2.0 mL) was added. The reaction mixture was stirred at –20 °C for 10 min followed by another 30 min at 25 °C, before sat. *aq.* NH_4Cl solution was added for quenching the reaction mixture. The aqueous layer was extracted three times with EtOAc (3×30 mL) and the combined organic layers were dried over anhydrous $MgSO_4$ and filtrated. After removal of the solvent, flash column chromatographical purification (silica gel, pentane:EtOAc = 85:15 → 75:25) afforded the title compound **22a** as a colorless crystals (83 mg, 0.34 mmol, 85% yield).

^{1}H-NMR (400 MHz, $CDCl_3$): δ / ppm = 8.50 – 8.45 (m, 1H), 7.60 (td, *J* = 7.7, 1.9 Hz, 1H), 7.17 – 7.10 (m, 2H), 6.06 (s, 1H), 3.13 (s, 2H), 2.31 (dd, *J* = 12.5, 3.1 Hz, 2H), 1.95 – 1.88 (m, 2H), 1.86 – 1.81 (m, 1H), 1.80 – 1.75 (m, 1H), 1.71 – 1.65 (m, 4H), 1.58 – 1.53 (m, 2H), 1.48 – 1.41 (m, 2H).

^{13}C-NMR (100 MHz, $CDCl_3$): δ / ppm = 159.8, 148.5, 136.9, 124.6, 121.5, 75.6, 43.7, 38.6, 37.4 (2C), 34.8 (2C), 32.9 (2C), 27.6, 27.5.

IR (Diamond-ATR, neat): $\tilde{\nu}$ / cm^{-1} = 3258, 2942, 2912, 2896, 2890, 2854, 1592, 1567, 1476, 1466, 1453, 1432, 1421, 1356, 1337, 1184, 1098, 1063, 1051, 1027, 1013, 1003, 992, 932, 886, 868, 761, 750, 704, 680, 664.

MS (EI, 70 eV): *m/z* (%) = 224 (22), 93 (100).

HRMS (EI-orbitrap): *m/z:* [M–H] calc. for [$C_{16}H_{20}NO$]: 242.1545; found 242.1538.

m.p. (°C): 84.3 87.1.

1,1-diphenyl-2-(pyridin-2-yl)ethan-1-ol (22b)

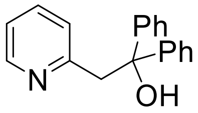

According to **TP4**, a solution of 3-(chloromethyl)heptane (**2**, 0.2 M, 2.0 equiv) in *n*-hexane was prepared. The solution of **2** was pumped through the activated sodium packed-bed reactor (see **TP1**) by pump A (flow rate: 2.0 mL/min) into the precooling loop (V_{pre2} = 0.35 mL) at 25 °C. Subsequently upon reaching the steady state, it was injected for 2 min into a flask charged with a solution of TMEDA (0.80 mmol, 2.0 equiv) and 2-picoline (**20a**, 37 mg, 0.40 mmol, 1.0 equiv) in hexane (2.0 mL) the mixture was stirred for 1 h at 25 °C. Before the mixture was cooled to –40°C and a solution of benzophenone (**9o**, 145 mg, 0.80 mmol, 2.0 equiv) in THF (2.0 mL) was added. The reaction mixture was stirred at –20 °C for 10 min followed by another 30 min at 25 °C, before sat. *aq.* NH_4Cl solution was added for quenching the reaction mixture. The aqueous layer was extracted three times with EtOAc (3×30 mL) and the combined organic layers were dried over anhydrous $MgSO_4$ and filtrated. After removal of the solvent, flash column chromatographical purification (silica gel, pentane:EtOAc = 80:20 → 60:40) afforded the title compound **22b** as a colorless crystals (110 mg, 0.39 mmol, 99% yield).

^{1}H-NMR (400 MHz, $CDCl_3$): δ / ppm = 8.41 – 8.29 (m, 1H), 7.74 (s, 1H), 7.54 – 7.44 (m, 5H), 7.29 – 7.19 (m, 4H), 7.18 – 7.11 (m, 2H), 7.08 – 6.99 (m, 2H), 3.71 (s, 2H).

^{13}C-NMR (100 MHz, $CDCl_3$): δ / ppm = 159.4, 148.0, 147.4 (2C), 137.0, 128.0 (4C), 126.6 (2C), 126.3 (4C), 124.7, 121.6, 78.5, 47.1.

IR (Diamond-ATR, neat): $\tilde{\nu}$ / cm^{-1} = 3212, 1594, 1568, 1491, 1479, 1445, 1437, 1416, 1248, 1221, 1196, 1104, 1054, 1032, 1010, 998, 908, 891, 791, 764, 749, 709, 697.

MS (EI, 70 eV): *m/z* (%) = 257 (20), 256 (100), 254 (14), 105 (10).

HRMS (EI-orbitrap): *m/z:* [M] calc. for [$C_{19}H_{17}NO$]: 275.1310; found 275.1306.

m.p. (°C): 149.4 – 152.0.

1,1-diphenyl-2-(pyridin-4-yl)ethan-1-ol (22c)

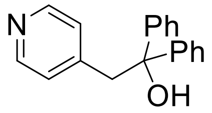

According to **TP4**, a solution of 3-(chloromethyl)heptane (**2**, 0.2 M, 2.0 equiv) in *n*-hexane was prepared. The solution of **2** was pumped through the activated sodium packed-bed reactor (see **TP1**) by pump A (flow rate: 2.0 mL/min) into the precooling loop (V_{pre2} = 0.35 mL) at 25 °C. Subsequently upon reaching the steady state, it was injected for 2 min into a flask charged with a solution of TMEDA (0.80 mmol, 2.0 equiv) and 4-picoline (**20b**, 37 mg, 0.40 mmol, 1.0 equiv) in hexane (2.0 mL) the mixture was stirred for 1 h at 25 °C. Before the mixture was cooled to –40°C and a solution of benzophenone (**9o**, 145 mg, 0.80 mmol, 2.0 equiv) in THF (2.0 mL) was added. The reaction mixture was stirred at –20 °C for 10 min followed by another 30 min at 25 °C, before sat. *aq.* NH_4Cl solution was added for quenching the reaction mixture. The aqueous layer was extracted three times with EtOAc (3×30 mL) and the combined organic layers were dried over anhydrous $MgSO_4$ and filtrated. After removal of the solvent, flash column chromatographical purification (silica gel, pentane:EtOAc = 80:20 → 40:60) afforded the title compound **22c** as a colorless crystals (95 mg, 0.35 mmol, 86% yield).

^{1}H-NMR (400 MHz, $CDCl_3$): δ / ppm = 8.24 – 8.14 (m, 2H), 7.42 – 7.34 (m, 4H), 7.33 – 7.26 (m, 4H), 7.26 – 7.21 (m, 2H), 6.85 – 6.77 (m, 2H), 3.58 (s, 2H), 3.23 (s, 1H).

^{13}C-NMR (100 MHz, $CDCl_3$): δ / ppm = 148.9 (2C), 146.3 (2C), 146.1, 128.3 (4C), 127.3 (2C), 126.3 (4C), 126.3 (2C), 78.1, 47.6.

IR (Diamond-ATR, neat): $\tilde{\nu}$ / cm^{-1} = 3144, 3059, 1605, 1493, 1446, 1418, 1212, 1051, 1003, 905, 814, 776, 753, 730, 710, 696.

MS (EI, 70 eV): *m/z* (%) = 257 (17), 256 (16), 207 (22), 183 (13), 182 (100), 180 (14), 170 (10), 169 (79), 168 (45), 167 (69), 166 (10), 105 (10), 91 (15).

HRMS (EI-orbitrap): *m/z:* [M–H_2O] calc. for [$C_{19}H_{15}N$]: 257.1204; found 257.1201.

m.p. (°C): 159.0 – 160.5.

4-(4-phenylbutyl)pyridine (22d)

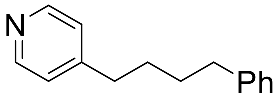

According to **TP4**, a solution of 3-(chloromethyl)heptane (**2**, 0.2 M, 2.0 equiv) in *n*-hexane was prepared. The solution of **2** was pumped through the activated sodium packed-bed reactor (see **TP1**) by pump A (flow rate: 2.0 mL/min) into the precooling loop (V_{pre2} = 0.35 mL) at 25 °C. Subsequently upon reaching the steady state, it was injected for 2 min into a flask charged with a solution of TMEDA (0.80 mmol, 2.0 equiv) and 4-picoline (**20b**, 37 mg, 0.40 mmol, 1.0 equiv) in hexane (2.0 mL) the mixture was stirred for 1 h at 25 °C. Before the mixture was cooled to –40°C and a solution of (3-chloropropyl)benzene (**12b**, 123 mg, 0.80 mmol, 2.0 equiv) in THF (2.0 mL) was added. The reaction mixture was stirred at –20 °C for 10 min followed by another 30 min at 25 °C, before sat. *aq.* NH_4Cl solution was added for quenching the reaction mixture. The aqueous layer was extracted three times with EtOAc (3×30 mL) and the combined organic layers were dried over anhydrous $MgSO_4$ and filtrated. After removal of the solvent, flash column chromatographical purification (silica gel, pentane:EtOAc = 90:10 → 60:40) afforded the title compound **22d** as a colorless oil (42 mg, 0.20 mmol, 50% yield).

^{1}H-NMR (400 MHz, $CDCl_3$): δ / ppm = 8.60 – 8.45 (m, 2H), 7.37 – 7.29 (m, 2H), 7.28 – 7.18 (m, 3H), 7.16 – 7.10 (m, 2H), 2.80 – 2.56 (m, 4H), 1.83 – 1.59 (m, 4H).

^{13}C-NMR (100 MHz, $CDCl_3$): δ / ppm = 151.5, 149.7 (2C), 142.3, 128.5 (4C), 125.9, 124.0 (2C), 35.8, 35.2, 31.0, 29.9.

IR (Diamond-ATR, neat): $\tilde{\nu}$ / cm^{-1} = 3064, 3025, 2934, 2858, 1601, 1558, 1496, 1453, 1414, 1219, 1030, 992, 839, 800, 774, 746, 698.

MS (EI, 70 eV): *m/z* (%) = 182 (12), 120 (13), 106 (100), 92 (10), 91 (100), 65 (12).

HRMS (EI-orbitrap): *m/z:* [M] calc. for [$C_{15}H_{17}N$]: 211.1361; found 211.1354.

(2*S*)-1-(benzyloxy)-4-phenyl-4-(pyridin-2-yl)butan-2-ol (22e) mixture of diastereoisomers

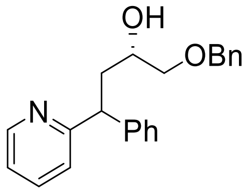

According to **TP4**, a solution of 3-(chloromethyl)heptane (**2**, 0.2 M, 2.0 equiv) in *n*-hexane was prepared. The solution of **2** was pumped through the activated sodium packed-bed reactor (see **TP1**) by pump A (flow rate: 2.0 mL/min) into the precooling loop (V_{pre2} = 0.35 mL) at 25 °C. Subsequently upon reaching the steady state, it was injected for 2 min into a flask charged with a solution of TMEDA (0.80 mmol, 2.0 equiv) and 2-benzylpyridine (**20c**, 68 mg, 0.40 mmol, 1.0 equiv) in hexane (2.0 mL) the mixture was stirred for 1 h at 25 °C. Before the mixture was cooled to –40°C and a solution of (S)-2-((benzyloxy)methyl)oxirane (**9m**, 131 mg, 0.80 mmol, 2.0 equiv) in THF (2.0 mL) was added. The reaction mixture was stirred at –20 °C for 10 min followed by another 30 min at 25 °C, before sat. *aq.* NH_4Cl solution was added for quenching the reaction mixture. The aqueous layer was extracted three times with EtOAc (3×30 mL) and the combined organic layers were dried over anhydrous $MgSO_4$ and filtrated. After removal of the solvent, flash column chromatographical purification (silica gel, pentane:EtOAc = 90:10 → 40:60) afforded the title compound (**22e**) as a slightly yellow oil (82 mg, 0.25 mmol, 62% yield; d.r. = 1.4:1.0).

Major:

^{1}H-NMR (400 MHz, $CDCl_3$): δ / ppm = 8.60 – 8.50 (m, 1H), 7.60 – 7.50 (m, 1H), 7.37 – 7.24 (m, 9H), 7.23 – 7.06 (m, 3H), 4.53 – 4.47 (m, 3H), 3.71 – 3.63 (m, 1H), 3.53 – 3.46 (m, 1H), 3.45 – 3.39 (m, 1H), 2.53 – 2.43 (m, 1H), 2.18 – 2.09 (m, 1H), 2.08 (s, 1H).

^{13}C-NMR (100 MHz, $CDCl_3$): δ / ppm = 163.0, 148.8, 143.6, 138.2, 136.9, 128.6 (2C), 128.5 (2C), 128.2 (2C), 127.9 (2C), 127.8, 126.6, 123.9, 121.6, 74.8, 73.4, 67.9, 49.4, 38.5.

Minor:

^{1}H-NMR (400 MHz, $CDCl_3$): δ / ppm = 8.60 – 8.50 (m, 1H), 7.60 – 7.50 (m, 1H), 7.37 – 7.24 (m, 9H), 7.23 – 7.06 (m, 3H), 4.52 (s, 2H), 4.40 (dd, *J* = 8.4, 6.4 Hz, 1H), 3.71 – 3.63 (m, 1H), 3.53 – 3.46 (m, 1H), 3.45 – 3.39 (m, 1H), 2.40 – 2.26 (m, 2H).

^{13}C-NMR (100 MHz, $CDCl_3$): δ / ppm = 163.8, 148.7, 143.7, 138.2, 136.7, 128.7 (2C), 128.5 (2C), 128.5 (2C), 127.9 (2C), 127.8, 126.7, 123.5, 121.5, 75.1, 73.5, 69.0, 50.1, 38.8.

Mixture:

IR (Diamond-ATR, neat): $\tilde{\nu}$ / cm^{-1} = 3396, 3062, 3028, 2915, 2858, 1589, 1569, 1495, 1472, 1452, 1433, 1363, 1308, 1251, 1205, 1149, 1121, 1088, 1074, 1052, 1028, 995, 909, 876, 802, 744, 733, 697.

MS (EI, 70 eV): *m/z* (%) = 213 (12), 212 (77), 194 (16), 183 (11), 182 (79), 180 (15), 169 (66), 169 (13), 168 (100), 168 (11), 167 (78), 166 (10), 91 (24).

HRMS (EI-orbitrap): *m/z:* [M] calc. for [$C_{22}H_{23}NO_2$]: 333.1729; found 333.1673.

2-(1,4-diphenylbutyl)pyridine (22f)

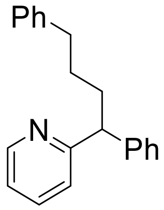

According to **TP4**, a solution of 3-(chloromethyl)heptane (**2**, 0.2 M, 2.0 equiv) in *n*-hexane was prepared. The solution of **2** was pumped through the activated sodium packed-bed reactor (see **TP1**) by pump A (flow rate: 2.0 mL/min) into the precooling loop (V_{pre2} = 0.35 mL) at 25 °C. Subsequently upon reaching the steady state, it was injected for 2 min into a flask charged with a solution of TMEDA (0.80 mmol, 2.0 equiv) and 2-benzylpyridine (**20c**, 68 mg, 0.40 mmol, 1.0 equiv) in hexane (2.0 mL) the mixture was stirred for 1 h at 25 °C. Before the mixture was cooled to –40°C and a solution of (3-chloropropyl)benzene (**12b**, 123 mg, 0.80 mmol, 2.0 equiv) in THF (2.0 mL) was added. The reaction mixture was stirred at –20 °C for 10 min followed by another 30 min at 25 °C, before sat. *aq.* NH_4Cl solution was added for quenching the reaction mixture. The aqueous layer was extracted three times with EtOAc (3×30 mL) and the combined organic layers were dried over anhydrous $MgSO_4$ and filtrated. After removal of the solvent, flash column chromatographical purification (silica gel, pentane:EtOAc = 95:5 → 85:15) afforded the title compound **22f** as a colorless oil (90 mg, 0.31 mmol, 78% yield).

^{1}H-NMR (400 MHz, $CDCl_3$): δ / ppm = 8.63 – 8.57 (m, 1H), 7.57 (td, *J* = 7.7, 1.9 Hz, 1H), 7.39 – 7.25 (m, 6H), 7.24 – 7.13 (m, 5H), 7.12 – 7.08 (m, 1H), 4.11 (t, *J* = 7.8 Hz, 1H), 2.76 – 2.62 (m, 2H), 2.41 – 2.27 (m, 1H), 2.23 – 2.10 (m, 1H), 1.68 – 1.59 (m, 2H).

^{13}C-NMR (100 MHz, $CDCl_3$): δ / ppm = 164.0, 149.4, 143.8, 142.5, 136.5, 128.6 (2C), 128.5 (2C), 128.4 (2C), 128.1 (2C), 126.5, 125.8, 122.8, 121.4, 53.8, 36.0, 34.8, 29.9.

IR (Diamond-ATR, neat): $\tilde{\nu}$ / cm^{-1} = 3061, 3025, 2934, 2858, 1601, 1588, 1568, 1494, 1471, 1452, 1432, 1148, 1073, 1050, 1030, 993, 910, 790, 745, 697.

MS (EI, 70 eV): *m/z* (%) = 183 (14), 182 (100), 170 (10), 169 (79), 168 (47), 167 (70), 166 (10), 91 (12).

HRMS (EI-orbitrap): *m/z:* [M] calc. for [$C_{21}H_{21}N$]: 287.1674; found 287.1677.

Synthesis of fingolimod (5)

***tert*-butyl (5-(hydroxymethyl)-2,2-dimethyl-1,3-dioxan-5-yl)carbamate**

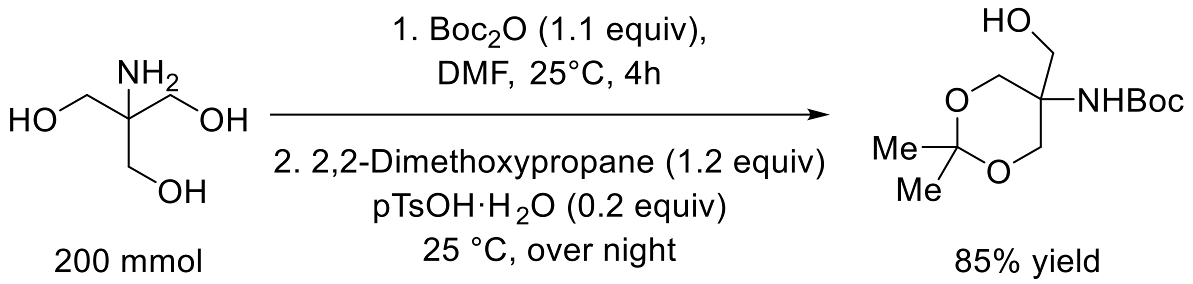

According to the literature,[6] Boc_2O (48.02 g, 220 mmol, 1.1 equiv) was added to a mixture of 2-amino-2-(hydroxymethyl)propane-1,3-diol (24.33 g, 200 mmol, 1.0 equiv) in DMF (180 mL) at 25 °C. The mixture was stirred at this temperature for 4 h. 2,2-Dimethoxypropane (25.00 g, 240 mmol, 1.2 equiv) and *p*-TsOH·H_2O (1.90 g, 10 mmol, 0.2 equiv) were added at 25 °C and the mixture was stirred over night at the same temperature. Et_2O (500 mL) was added, the mixture was washed with $NaHCO_3$ (3x200 mL) and brine (200 mL), the organic layer was dried over $MgSO_4$. Evaporation of the solvent led to the title compound as a colorless solid (44. 65 g, 171 mmol, 85% yield).

^{1}H-NMR (400 MHz, $CDCl_3$): δ / ppm = 5.34 (s, 1H), 4.29 (s, 1H), 3.90 – 3.76 (m, 4H), 3.72 (d, *J* = 6.6 Hz, 2H), 1.53 (s, 3H), 1.45 (s, 9H), 1.44 (s, 3H).

^{13}C-NMR (100 MHz, $CDCl_3$): δ / ppm = 156.6, 98.9, 80.6, 64.6 (2C), 53.5, 28.5 (3C), 27.6 (2C), 20.2.

[6] J. Doubský, S. Rádl, J. Cinibulk, Robert Klvaňa, *Org. Process. Res. Dev.* Doi: *10.1021/acs.oprd.1c00248*

(5-((*tert*-butoxycarbonyl)amino)-2,2-dimethyl-1,3-dioxan-5-yl)methyl methanesulfonate

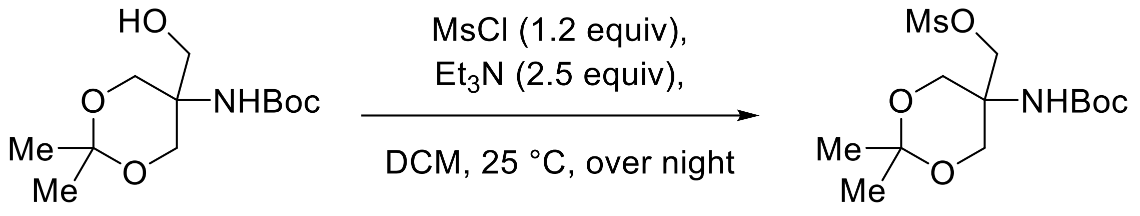

100 mmol 88% yield

According to the literature,[5] NEt_3 (25.3 g, 250 mmol, 2.5 equiv) was added at 0 °C to a solution of tert-butyl (5-(hydroxymethyl)-2,2-dimethyl-1,3-dioxan-5-yl)carbamate (26.1 g, 100 mmol, 1.0 equiv) in DCM (100 mL). MsCl was added dropwise at the same temperature, the reaction mixture was allowed to slowly warm up to 25 °C and stirred over night at this temperature. MTBE (200 mL) was added to the mixture the organic layer was washed with H_2O (2x300 mL), citric acid (10 wt%, 2x200 mL) and $NaHCO_3$ (200 mL) and dried over $MgSO_4$. Evaporation of solvents gave the title compound as a slightly yellow oil (29.9 g, 88.1 mmol, 88% yield).

^{1}H-NMR (400 MHz, $CDCl_3$): δ / ppm = 4.73 (s, 1H), 4.58 (s, 2H), 4.02 (d, *J* = 11.9 Hz, 2H), 3.88 (d, *J* = 12.0 Hz, 2H), 3.04 (s, 3H), 1.48 (s, 3H), 1.43 (s, 9H), 1.41 (s, 3H).

[5] J. Doubský, S. Rádl, J. Cinibulk, Robert Klvaňa, *Org. Process. Res. Dev.* Doi: *10.1021/acs.oprd.1c00248*

tert-butyl 6,6-dimethyl-5,7-dioxa-1-azaspiro[2.5]octane-1-carboxylate (25)

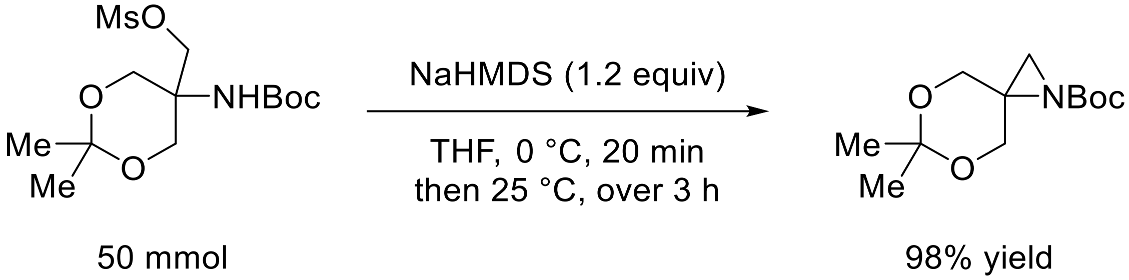

According to the literature,[5] NaHMDS (1.0 m, 60 mL, 60 mmol, 1.2 equiv) was added dropwise to a solution of (5-((tert-butoxycarbonyl)amino)-2,2-dimethyl-1,3-dioxan-5-yl)methyl methanesulfonate (16.97 g, 50 mmol, 1.0 equiv) in THF (250 mL) at –10 °C the mixture was stirred at the same temperature for 20 min. Afterwards the mixture was stirred for 3 h at 25 °C. The mixture was quenched with sat. *aq.* NH_4Cl (100 mL), H_2O (100 mL) was added, the mixture was diluted with MTBE (200 mL). The aqueous layer was extracted with MTBE (2x100 mL), the combined organic layers were washed with citric acid (10 wt%, 500 mL) and sat *aq.* $NaHCO_3$ (400 mL). The organic layers were dried over $MgSO_4$. Evaporation of solvents gave the title compound **25** as a beige solid (11.93 g, 49.05 mmol, 98% yield).

^{1}H-NMR (400 MHz, $CDCl_3$): δ / ppm = 3.96 (d, J = 12.5 Hz, 2H), 3.65 (d, J = 12.6 Hz, 2H), 2.21 (s, 2H), 1.52 (s, 3H), 1.47 (s, 12H).

^{13}C-NMR (100 MHz, $CDCl_3$): δ / ppm = 160.5, 98.7, 81.9, 64.0 (2C), 40.3, 35.5, 28.0 (3C), 24.7, 22.8.

[5] J. Doubský, S. Rádl, J. Cinibulk, Robert Klvaňa, *Org. Process. Res. Dev.* Doi: *10.1021/acs.oprd.1c00248*

1-methyl-4-octylbenzene (13s)

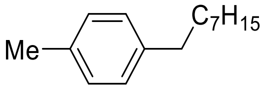

A solution of 3-(chloromethyl)heptane (**2**, 0.2 M, 2.0 equiv) in *n*-hexane was prepared. The solution of **2** was pumped through the activated sodium packed-bed reactor (see **TP1**) by pump A (flow rate: 2.0 mL/min) into the precooling loop (V_{pre2} = 0.35 mL) at 25 °C. Subsequently upon reaching the steady state, it was injected (40 min) into a flask charged with TMEDA (16.00 mmol, 2.0 equiv) in xylene (**3c**, 20.0 mL) and the resulting mixture was stirred at 25 °C for 1 h. The reaction flask was cooled to –20 °C and a solution of 1-chloroheptane (**12p**, 1.08 g, 8.00 mmol, 1.0 equiv) in THF (20.0 mL) was added The reaction mixture was stirred at –20 °C for 10 min before it was allowed to warm to 25 °C over night. Sat. *aq.* NH_4Cl solution was added for quenching the reaction mixture. The aqueous layer was extracted three times with EtOAc (3×30 mL) and the combined organic layers were dried over anhydrous $MgSO_4$ and filtrated. Removing the solvent afforded the title compound **13s** as a yellow oil (1.60 mg, 7.84 mmol, 98% yield) without further purification.

^{1}H-NMR (400 MHz, $CDCl_3$): δ / ppm = 7.11 – 7.02 (m, 4H), 2.60 – 2.51 (m, 2H), 2.32 (s, 3H), 1.67 – 1.50 (m, 2H), 1.37 – 1.21 (m, 10H), 0.92 – 0.83 (m, 3H).

^{13}C-NMR (100 MHz, $CDCl_3$): δ / ppm = 140.0, 135.1, 129.0 (2C), 128.4 (2C), 35.7, 32.0, 31.8, 29.6, 29.5, 29.4, 22.8, 21.1, 14.3.

IR (Diamond-ATR, neat): $\tilde{\nu}$ / cm^{-1} = 3358, 3020, 2956, 2923, 2854, 1516, 1464, 1458, 1378, 1118, 1022, 806, 723.

MS (EI, 70 eV): *m/z* (%) = 204 (27), 106 (21), 105 (100), 91 (16).

HRMS (EI-orbitrap): *m/z:* [M] calc. for [$C_{15}H_{24}$]: 204.1878; found 204.1872.

tert-butyl (2,2-dimethyl-5-(4-octylphenethyl)-1,3-dioxan-5-yl)carbamate (24)

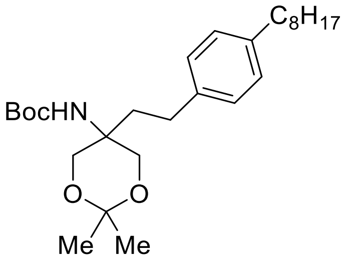

A solution of 3-(chloromethyl)heptane (**2**, 0.2 M, 0.8 equiv) in *n*-hexane was prepared. The solution of **2** was pumped through the activated sodium packed-bed reactor (see **TP1**) by pump A (flow rate: 2.0 mL/min) into the precooling loop (V_{pre2} = 0.35 mL) at 25 °C. Subsequently upon reaching the steady state, it was injected (4 min) into a flask charged with TMEDA (1.60 mmol, 0.8 equiv) and 1-methyl-4-octylbenzene (**13s**, 2.00 mmol, 1.0 equiv) in hexane (2.0 mL) and the resulting mixture was stirred at 25 °C for 30 min. The reaction mixture was cooled to –78 °C and MgCl·LiCl (0.5 M in THF, 1.2 mL, 0.60 mmol, 0.3 equiv) was added the mixture was stirred for 10 min at the same temperature.

In a second flask a mixture of tert-butyl 6,6-dimethyl-5,7-dioxa-1-azaspiro[2.5]octane-1-carboxylate (**25**, 97 mg, 0.40 mmol, 0.2 equiv) and CuBr·SMe_2 (16 mg, 0.08 mmol, 4 mol%) in THF (4.0 mL) was prepared and stirred for 10 min at 25 °C before it was cooled to –78 °C and the gas space was evacuated and refilled with argon for three times.

The mixture of electrophile **25** was transferred to the first flask filled with the organometallic reagent at –78 °C and the combined mixture was stirred at that temperature for 30 min before it was allowed to warm to 25 °c and stirred overnight.

Sat. *aq.* NH_4Cl solution was added for quenching the reaction mixture. The aqueous layer was extracted three times with EtOAc (3×30 mL) and the combined organic layers were dried over anhydrous $MgSO_4$ and filtrated. After removal of the solvent, flash column chromatographical purification (silica gel, pentane:EtOAc = 95:5 → 90:10) afforded the title compound **24** as a beige solid (107 mg, 0.24 mmol, 60% yield).

^{1}H-NMR (400 MHz, CDCl$_3$): δ / ppm = 7.08 (s, 4H), 4.98 (s, 1H), 3.90 (d, *J* = 11.6 Hz, 2H), 3.68 (d, *J* = 11.9 Hz, 2H), 2.60 – 2.46 (m, 4H), 2.02 – 1.91 (m, 2H), 1.62 – 1.53 (m, 2H), 1.48 (s, 9H), 1.44 (s, 3H), 1.42 (s, 3H), 1.28 (m, 10H), 0.90 – 0.85 (m, 3H).

^{13}C-NMR (100 MHz, CDCl$_3$): δ / ppm = 155.0, 140.7, 139.2, 128.6 (2C), 128.3 (2C), 98.5, 79.4, 66.5 (2C), 51.8, 35.7, 33.8, 32.0, 31.7, 29.6, 29.5, 29.4, 28.8, 28.6 (3C), 27.6, 22.8, 19.8, 14.3.

IR (Diamond-ATR, neat): $\tilde{\nu}$ / cm^{-1} = 3347, 2977, 2955, 2924, 2853, 1698, 1520, 1515, 1464, 1453, 1389, 1374, 1367, 1362, 1304, 1251, 1216, 1198, 1170, 1133, 1121, 1078, 1069, 1041, 1008, 1002, 940, 908, 827, 788, 731.

MS (EI, 70 eV): *m/z* (%) = 333 (12), 316 (14), 275 (23), 272 (11), 258 (25), 255 (14), 244 (10), 242 (11), 217 (21), 216 (100), 203 (19), 190 (17), 134 (15), 117 (27), 117 (11), 105 (23), 104 (13), 91 (11), 57 (75), 43 (12), 43 (15), 41 (17).

HRMS (EI-orbitrap): *m/z:* [M] calc. for [$C_{27}H_{45}NO_4$]: 447.3349; found 447.3347.

m.p. (°C): 68.0 – 70.2.

Fingolimod Hydrochloride (5)

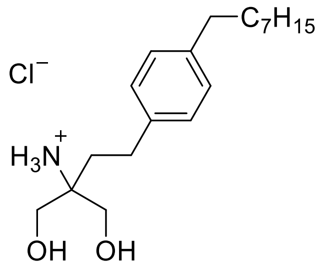

Aq. HCl (37%, 0.2 ml, ca. 2.4 mmol, 8.4 equiv) was added to a solution of *tert*-butyl (2,2-dimethyl-5-(4-octylphenethyl)-1,3-dioxan-5-yl)carbamate (**24**, 128 mg, 0.29 mmol, 1.0 equiv) in EtOH (5.5 mL). The mixture was heated to reflux for 2 d. Evaporation under reduced pressure led to the title compound **5** as slightly beige crystals (88 mg, 0.26 mmol, 89% yield).

^{1}H-NMR (400 MHz, DMSO-*d*$_6$): δ / ppm 7.82 (s, 3H), 7.10 (s, 4H), 5.38 (t, *J* = 5.0 Hz, 2H), 3.51 (d, *J* = 5.1 Hz, 4H), 2.60 – 2.51 (m, 4H), 1.80 – 1.71 (m, 2H), 1.58 – 1.46 (m, 2H), 1.31 – 1.18 (m, 10H), 0.89 – 0.81 (m, 3H).

^{13}C-NMR (100 MHz, DMSO-*d*$_6$): δ / ppm = 139.8, 138.8, 128.3 (2C), 128.0 (2C), 61.0, 60.2, 34.8, 33.3, 31.3, 31.1, 28.8, 28.7 (3C), 27.9, 22.1, 14.0.

IR (Diamond-ATR, neat): $\tilde{\nu}$ / cm^{-1} = 3354, 3261, 3034, 2952, 2920, 2872, 2851, 1600, 1515, 1469, 1456, 1439, 1417, 1242, 1117, 1067, 1045, 1030, 1020, 1002, 864, 824, 772, 764, 722.

HRMS (FTMS-ESI): *m/z:* [M–Cl$^-$] calc. for [$C_{19}H_{34}NO_2^+$]: 308.25841; found 308.25845.

[M–H$^+$] calc. for [$C_{19}H_{33}ClNO_2^-$]: 342.22053; found 342.22131.

m.p. (°C): 228.3 – 233.5.

Synthesis of salmeterol-*d_7* (6)

1-(3-(chloromethyl)-4-hydroxyphenyl)ethan-1-one

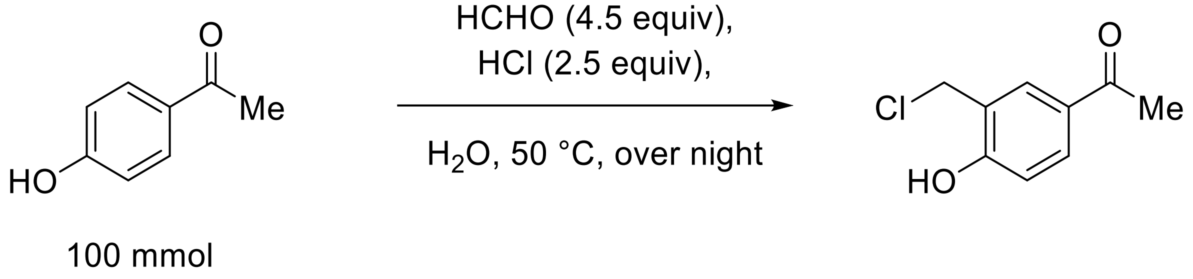

According to the literature[7], 4-Hydroxyacetophenone (13.62 g, 100 mmol, 1.0 equiv) was added *aq.* formaldehyde (37%, 33.5 mL, 450 mmol, 4.5 equiv) subsequently an aqueous solution of HCl (37%, 100 mL, 1223 mmol, 12.2 equiv) was added. The mixture was stirred over night at 50 °C. Filtration gave the crude title compound as a dark red solid which was used without further purification in the following step.

^{1}H-NMR (400 MHz, $CDCl_3$): δ / ppm = 7.95 (d, *J* = 2.2 Hz, 1H), 7.89 (dd, *J* = 8.4, 2.2 Hz, 1H), 6.91 (d, *J* = 8.4 Hz, 1H), 5.80 (s, 1H), 4.70 (s, 2H), 2.57 (s, 3H).

[7] N. Gisch, F. Pertenbreiter, J. Balzarini, C. Meier *J. Med. Chem.* **2008**, *51*, 8115 – 8123.

1-(4-hydroxy-3-(hydroxymethyl)phenyl)ethan-1-one

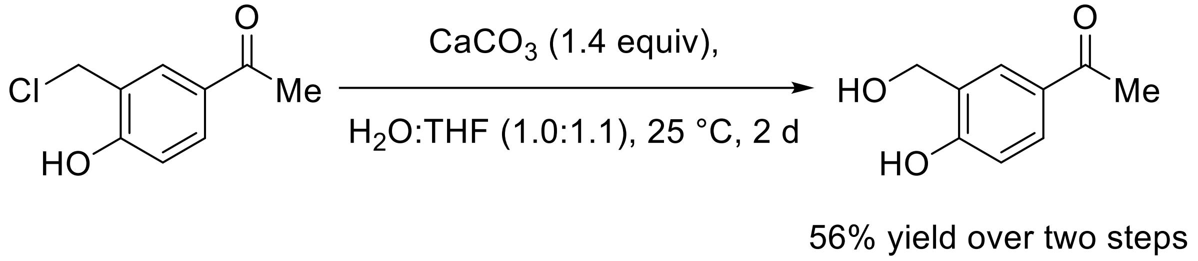

According to the literature[8], the crude 1-(3-(chloromethyl)-4-hydroxyphenyl)ethan-1-one was used without further purification and dissolved in THF (200 mL), and H_2O (175 mL). $CaCO_3$ (14.0 g, 140.0 mmol, 1.4 equiv) was added and the mixture was stirred for 2 d at 25 °C. The pH-value of the mixture was adjusted to pH = 6 by addition of aq. HCl (37%). The reaction mixture was extracted with EtOAc (3x200 mL). The combined organic layers were dried over $MgSO_4$. Flash column chromatographical purification (silica gel, DCM:MeOH = 19:1) afforded the title compound as a slightly pink solid (9.38 g, 56.4 mmol, 56% yield over two steps).

^{1}H-NMR (400 MHz, $CDCl_3$): δ / ppm = 8.06 (d, *J* = 2.0 Hz, 1H), 7.84 (dd, *J* = 8.5, 2.2 Hz, 1H), 7.70 (d, *J* = 2.2 Hz, 1H), 6.93 (d, *J* = 8.5 Hz, 1H), 4.96 (s, 2H), 2.54 (s, 3H) 2.32 (s, 1H).

^{13}C-NMR (100 MHz, $CDCl_3$): δ / ppm = 197.0, 161.0, 130.9, 129.8, 128.4, 124.2, 116.8, 65.0, 26.5.

[8] N. Gisch, F. Pertenbreiter, J. Balzarini, C. Meier *J. Med. Chem.* **2008**, *51*, 8115 – 8123.

1-(2,2-dimethyl-4*H*-benzo[*d*][1,3]dioxin-6-yl)ethan-1-one

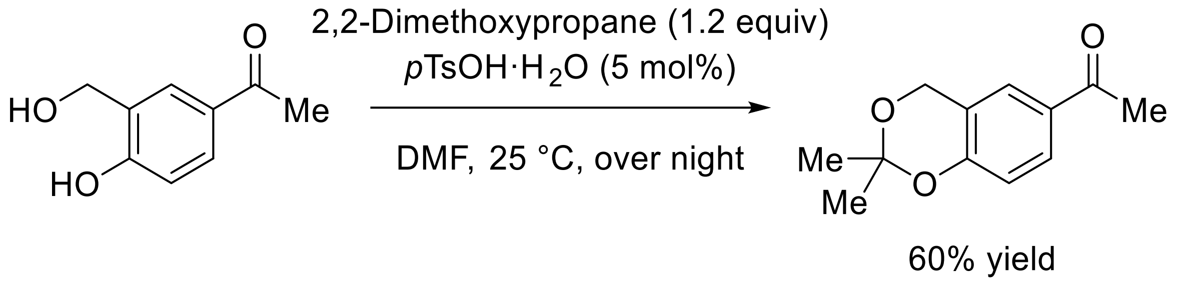

*p*TsOH·H_2O (0.52 g, 2.8 mmol, 5 mol%) was added to a mixture of 1-(4-hydroxy-3-(hydroxymethyl)phenyl)ethan-1-one (9.38 g, 56.4, 1.00 equiv) and 2,2-dimethoxypropane (7.00 g, 67.2 mmol, 1.2 equiv) in DMF (60 mL). The mixture was stirred over night at 25 °C. The solution was diluted with Et_2O (400 mL) and washed with $NaHCO_3$ (3x200 mL). The combined organic layers were dried over anhydrous $MgSO_4$. Solvents were removed *in vacuo* to give the title compound as a slightly orange solid (6.92 g, 33.6 mmol, 60% yield).

^{1}H-NMR (400 MHz, $CDCl_3$): δ / ppm = 7.79 (dd, *J* = 8.6, 2.2 Hz, 1H), 7.67 – 7.63 (m, 1H), 6.86 (d, *J* = 8.6 Hz, 1H), 4.89 (s, 2H), 2.55 (s, 3H), 1.56 (s, 6H).

^{13}C-NMR (100 MHz, $CDCl_3$): δ / ppm = 197.0, 155.8, 130.0, 129.2, 125.8, 119.3, 117.2, 100.7, 60.9, 26.5, 24.9 (2C).

2-bromo-1-(2,2-dimethyl-4*H*-benzo[*d*][1,3]dioxin-6-yl)ethan-1-one

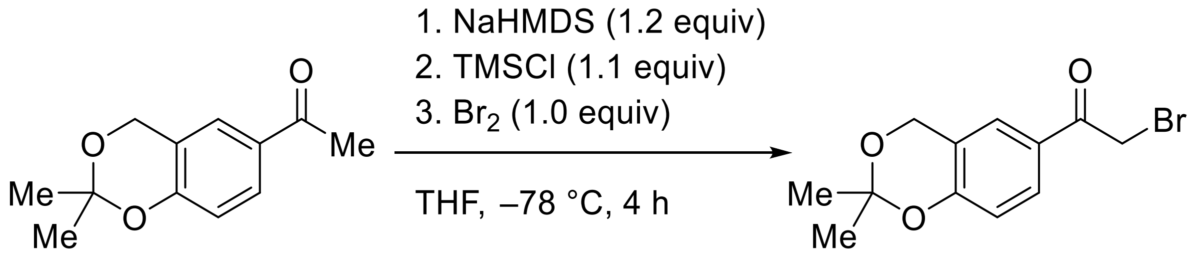

75% yield

NaHMDS (1.0 M in THF, 20 mmol, 20 mL, 1.2 equiv) was added dropwise to a solution of 1-(2,2-dimethyl-4H-benzo[d][1,3]dioxin-6-yl)ethan-1-one (3.40 g, 16.5 mmol, 1.0 equiv) in THF (120 mL) at –78 °C. The mixture was stirred for 40 min at the same temperature before TMSCl (1.98 g, 18.2 mmol, 1.1 equiv) was added and the mixture was stirred for another 2 h at –78 °C. Br_2 (2.64 g, 16.5 mmol, 1.0 equiv) was added dropwise at –78 C and stirring was continued for 1 h. The mixture was allowed to warm up to 25 °C and sat. aq. $Na_2S_2O_3$ (100 mL) was added. The aqueous layer was extracted with EtOAc (3x100 mL). The combined organic layers were dried over anhydrous $MgSO_4$. Flash column chromatographical purification (silica gel, pentane:EtOAc = 9:1) afforded the title compound as a colorless oil (3.52 g, 12.3 mmol, 75% yield).

^{1}H-NMR (400 MHz, $CDCl_3$): δ / ppm = 7.82 (dd, *J* = 8.7, 2.2 Hz, 1H), 7.72 – 7.61 (m, 1H), 6.88 (d, *J* = 8.6 Hz, 1H), 4.89 (s, 2H), 4.38 (s, 2H), 1.57 (s, 6H).

^{13}C-NMR (100 MHz, $CDCl_3$): δ / ppm = 190.1, 156.5, 129.8, 126.7, 126.6, 119.7, 117.5, 101.0, 60.8, 30.7, 25.0 (2C).

MS (EI, 70 eV): *m/z* (%) = 229 (15), 228 (15), 227 (14), 226 (15), 191 (16), 147 (24), 134 (10), 133 (100), 91 (14).

HRMS (EI-orbitrap): *m/z:* [M] calc. for [$C_{12}H_{13}$BrO3]: 284.0048; found 284.0044.

(S)-1-(2,2-dimethyl-4*H*-benzo[*d*][1,3]dioxin-6-yl)-2-((1-phenylethyl)amino)ethan-1-one

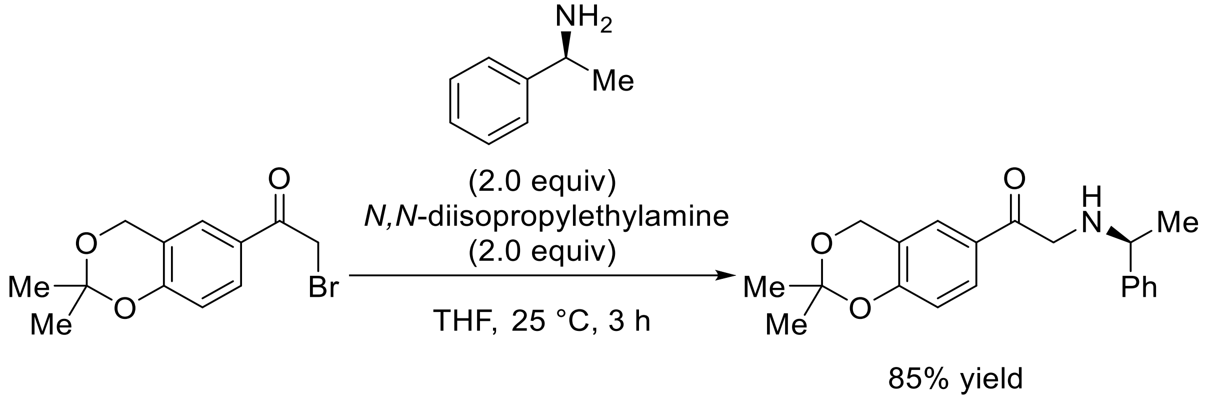

(S)-1-phenylethan-1-amine (2.9 g, 24.0 mmol, 2.0 equiv) was added at 0 °C to a solution of 2-bromo-1-(2,2-dimethyl-4H-benzo[d][1,3]dioxin-6-yl)ethan-1-one (3.4 g, 12.0 mmol, 1.0 equiv), *N,N*-diisopropyletylamine (3.1 g, 24.0 mmol, 2.0 equiv) and THF (26 mL). The mixture was stirred for 3 h at 25 °C. Solids were removed *via* filtration, solvents of the filtrate were removed *in vacuo* and the crude product was purified by flash column chromatograph (silica gel, pentane:EtOAc = 1:1) to give the title compound as a yellow oil (3.3 g, 10.1 mmol, 85%).

^{1}H-NMR (400 MHz, CDCl$_3$): δ / ppm = 7.68 (dd, *J* = 8.7, 2.2 Hz, 1H), 7.55 (d, *J* = 2.1 Hz, 1H), 7.38 – 7.29 (m, 4H), 7.29 – 7.21 (m, 1H), 6.80 (d, *J* = 8.6 Hz, 1H), 4.84 (s, 2H), 3.91 (d, *J* = 1.7 Hz, 2H), 3.82 (q, *J* = 6.6 Hz, 1H), 1.54 (s, 6H), 1.43 (d, *J* = 6.6 Hz, 3H).

^{13}C-NMR (100 MHz, CDCl$_3$): δ / ppm = 196.3, 156.0, 144.9, 128.8 (2C), 128.4, 128.1, 127.4, 126.9 (2C), 125.3, 119.4, 117.3, 100.7, 60.8, 58.4, 53.3, 24.9, 24.9, 24.7.

MS (EI, 70 eV): *m/z* (%) = 134 (30), 133 (21), 120 (22), 105 (100), 77 (15).

HRMS (EI-orbitrap): *m/z:* [M] calc. for [$C_{20}H_{23}NO_3$]: 325.1678; found 325.1662.

1-(2,2-dimethyl-4*H*-benzo[*d*][1,3]dioxin-6-yl)-2-(((S)-1-phenylethyl)amino)ethan-1-ol

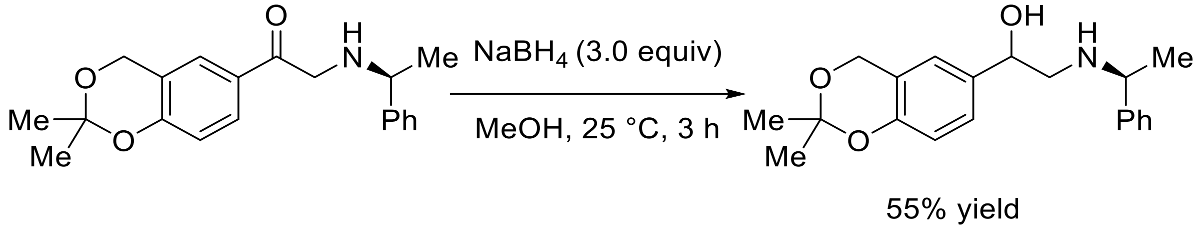

$NaBH_4$ (1.13 g, 30.0 mmol, 3.0 equiv) was added to a mixture of (S)-1-(2,2-dimethyl-4H-benzo[d][1,3]dioxin-6-yl)-2-((1-phenylethyl)amino)ethan-1-one (3.31 g, 10.0 mmol, 1.0 equiv) in MeOH (40 mL) at 0 °C.The mixture was stirred for 1 h at this temperature before it was allowed to warm up to 25 °C and stir for another 2 h at 25 °C. The mixture was filtered and the solid residue was washed with MeOH and H_2O to give the title compound as a colorless solid (1.81 g, 5.5 mmol, 55% yield.

^{1}H-NMR (400 MHz, $CDCl_3$): δ / ppm = 7.33 (m, 4H), 7.24 (m, 1H), 7.06 (dd, *J* = 8.5, 2.2 Hz, 1H), 6.94 (d, *J* = 2.1 Hz, 1H), 6.74 (dd, *J* = 8.5, 3.4 Hz, 1H), 4.80 (m, 2H), 4.56 (dd, *J* = 9.1, 3.4 Hz, 1H), 3.83 (q, *J* = 6.6 Hz, 1H), 2.74 (dd, *J* = 12.2, 3.6 Hz, 1H), 2.63 (dd, *J* = 12.2, 9.0 Hz, 1H), 1.53 (s, 6H), 1.45 (d, *J* = 6.6 Hz, 3H).

2-amino-1-(2,2-dimethyl-4*H*-benzo[*d*][1,3]dioxin-6-yl)ethan-1-ol (26)

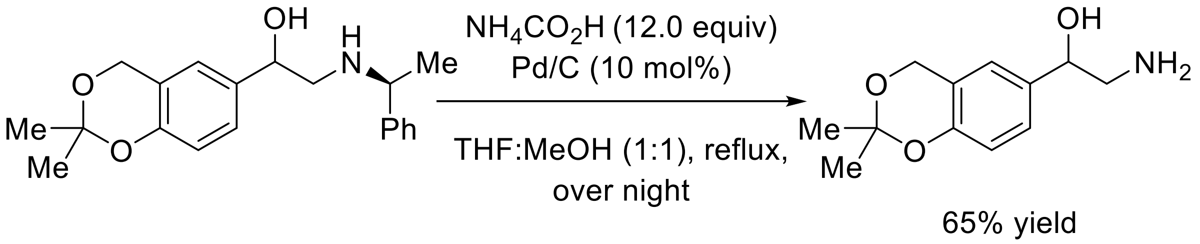

Pd/C (10 wt%, 0.53 g 0.5 mmol, 10 mol%) was added to a mixture of 1-(2,2-dimethyl-4H-benzo[d][1,3]dioxin-6-yl)-2-(((S)-1-phenylethyl)amino)ethan-1-ol (1.81 g, 5.5 mmol, 1.0 equiv), ammonium formate (4.16 g, 66 mmol, 12.0 equiv) in a solvent mixture of THF (10 mL) and MeOH (10 mL). The mixture was heated to reflux, after 4 h another 30 mL of THF were added and the mixture was stirred overnight under reflux. The mixture was filtrated and the solid residues were washed with THF and MeOH. Solvent of the filtrate was removed *in vacuo* and the crude product was purified *via* flash column chromatograph (silica gel, DCM:MeOH = 9:1) to give the title compound **26** as a slightly brown solid (0.80 g, 3.6 mmol, 65%).

^{1}H-NMR (400 MHz, $CDCl_3$): δ / ppm = 7.12 (dd, *J* = 8.4, 2.1 Hz, 1H), 7.02 – 6.95 (m, 1H), 6.79 (d, *J* = 8.3 Hz, 1H), 4.84 (s, 2H), 4.55 (dd, *J* = 8.0, 3.9 Hz, 1H), 2.98 (dd, *J* = 12.8, 3.8 Hz, 1H), 2.78 (dd, *J* = 12.7, 7.9 Hz, 1H), 1.72 (s, 2H), 1.53 (d, *J* = 1.9 Hz, 6H).

^{1}H NMR (400 MHz, DMSO): δ / ppm = 7.10 (dd, *J* = 8.4, 2.1 Hz, 1H), 7.02 (d, *J* = 2.1 Hz, 1H), 6.73 (d, *J* = 8.4 Hz, 1H), 4.80 (s, 2H), 4.43 (dd, *J* = 8.1, 4.1 Hz, 1H), 3.97 (s, 2H), 2.69 (dd, *J* = 12.8, 4.1 Hz, 1H), 2.60 (dd, *J* = 12.8, 8.1 Hz, 1H), 1.45 (s, 6H).

^{13}C-NMR (100 MHz, DMSO): δ / ppm = 149.7, 135.7, 125.7, 122.6, 119.0, 116.0, 99.1, 73.0, 60.2, 49.2, 24.6, 24.5.

4-(phenyl-d_5)butan-4,4-d_2-1-ol (10au)

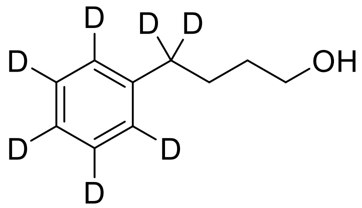

According to **TP3**, a solution of 3-(chloromethyl)heptane (**2**, 0.2 M, 2.0 equiv) in *n*-hexane was prepared. The solution of **2** was pumped through the activated sodium packed-bed reactor (see **TP1**) by pump A (flow rate: 2.0 mL/min) into the precooling loop (V_{pre2} = 0.35 mL) at 25 °C. Subsequently upon reaching the steady state, it was injected (2 min) into a flask charged with TMEDA (0.80 mmol, 2.0 equiv) in toluene-d_8 (**3p**, 1.0 mL) and the resulting mixture was stirred at 25 °C for 2 h. The reaction flask was cooled to –20 °C and a solution of oxetane (**9h**, 23 mg, 0.40 mmol, 1.0 equiv) in THF (1.0 mL) was added The reaction mixture was stirred at –20 °C for 10 min followed by another 30 min at 25 °C, before sat. *aq.* NH_4Cl solution was added for quenching the reaction mixture. The aqueous layer was extracted three times with EtOAc (3×30 mL) and the combined organic layers were dried over anhydrous $MgSO_4$ and filtrated. Removal of the solvent afforded the title compound **10au** as a colorless oil (47 mg, 0.30 mmol, 75% yield) without further purification.

^{1}H-NMR (400 MHz, $CDCl_3$): δ / ppm = 3.65 (t, J = 6.4 Hz, 2H), 1.75 – 1.66 (m, 2H), 1.66 – 1.51 (m, 3H).
^{13}C-NMR (100 MHz, $CDCl_3$): δ / ppm = 142.2, 128.1 (t, J = 23.7 Hz, 2C),127.9 (t, J = 24.2 Hz, 2C), 125.3 (t, J = 24.4 Hz), 62.8, 34.87 (p, J = 19.2 Hz), 32.3, 27.5.
IR (Diamond-ATR, neat): $\tilde{\nu}$ / cm^{-1} = 3327, 2932, 2862, 2273, 1455, 1433, 1384, 1328, 1057, 1034, 1010, 948, 821.
MS (EI, 70 eV): *m/z* (%) = 138 (13), 137 (12), 123 (25), 122 (16), 110 (100), 109 (63), 108 (16), 98 (94), 97 (33), 96 (13), 83 (11), 70 (13).
HRMS (EI-orbitrap): *m/z:* [M] calc. for [$C_{10}H_7D_7O$]: 157.1484; found 157.1478.

1-(4-((6-bromohexyl)oxy)butyl-1,1-d_2)benzene-2,3,4,5,6-d_5

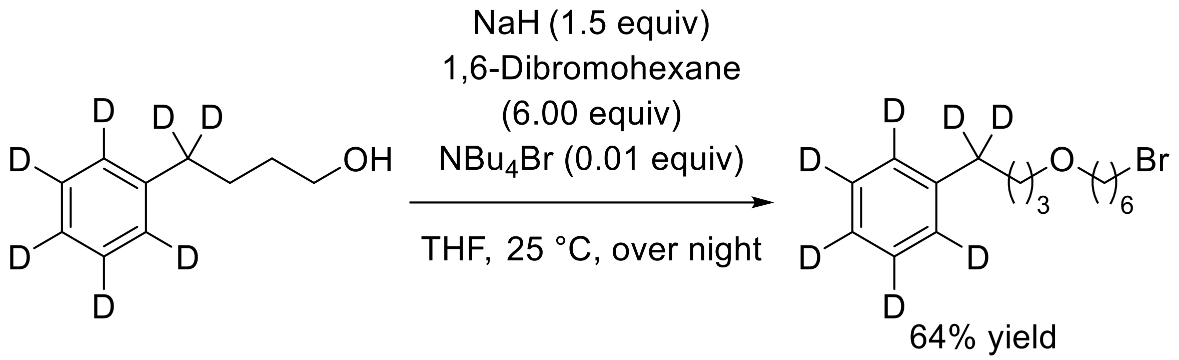

NaH (40 mg, 0.68 mmol, 1.50 equiv) was added to a solution of 4-(phenyl-d_5)butan-4,4-d_2-1-ol (**10au**, 70 mg, 0.45 mmol, 1.00 equiv) in THF (5.0 mL) at 25 °C, after stirring for 20 min NBu_4Br (2 mg, 0.005 mmol, 0.01 equiv) and 1,6-dibromohexane (659 mg, 2.7 mmol, 6.00 equiv) were added. The mixture was stirred over night at 25 °C. H_2O (20 mL) was added and the aqueous layer was extracted with EtOAc (3x50 mL). The combined organic layers were dried over anhydrous $MgSO_4$. Flash column chromatographical purification (silica gel, pentane → pentane:EtOAc = 9:1) afforded the title compound as a colorless oil (92 mg, 0.29 mmol, 64% yield).

^{1}H-NMR (400 MHz, $CDCl_3$): δ / ppm = 3.47 – 3.36 (m, 6H), 1.87 (p, *J* = 6.9 Hz, 2H), 1.71 – 1.54 (m, 6H), 1.49 – 1.35 (m, 4H).

^{13}C-NMR (100 MHz, $CDCl_3$): δ / ppm = 142.3, 128.0 (q, *J* = 23.6 Hz, 4C), 125.9 – 124.7 (m), 70.9, 70.8, 35.0 (p, *J* = 19.3 Hz), 34.0, 32.9, 29.7, 29.5, 28.1, 28.0, 25.5.

IR (Diamond-ATR, neat): $\tilde{\nu}$ / cm^{-1} = 2933, 2858, 1458, 1436, 1372, 1259, 1242, 1229, 1117.

MS (EI, 70 eV): *m/z* (%) = 138 (23), 137 (15), 111 (13), 110 (100), 109 (33), 98 (27), 97 (21), 96 (14), 83 (19), 55 (17), 43 (14), 41 (10).

HRMS (EI-orbitrap): *m/z:* [M] calc. for [$C_{16}H_{18}D_7BrO$]: 319.1528; found .319.1498.

1-(2,2-dimethyl-4*H*-benzo[*d*][1,3]dioxin-6-yl)-((6-(4-(phenyl-*d*$_5$)butoxy-4,4-*d*$_2$)hexyl)amino)ethan-1-ol

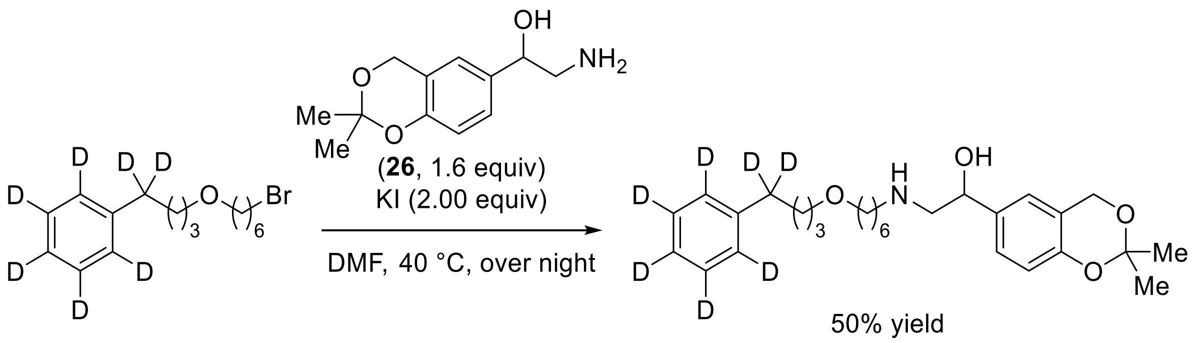

According to the literature,[9] KI (62 mg, 0.37 mmol, 1.0 equiv) and 2-amino-1-(2,2-dimethyl-4H-benzo[d][1,3]dioxin-6-yl)ethan-1-ol (**26**, 134 mg, 0.6 mmol, 1.6 equiv) was added to a mixture of 1-(4-((6-bromohexyl)oxy)butyl-1,1-*d*$_2$)benzene-2,3,4,5,6-*d*$_5$ (121 mg, 0.37 mmol, 1.0 equiv) in DMF (2 mL). The reaction mixture was heated to 40 °C and stirred over night. KI (62 mg, 0.37 mmol, 1.0 equiv) was added another time and again the mixture was stirred over night at 40 °C. The mixture was allowed to cool to 25 °C EtOAc was added (20 mL), the organic layer was washed with brine (3x30 mL). The combined organic layers were dried over anhydrous $MgSO_4$. Flash column chromatographical purification (silica gel, pentane:EtOAc:MeOH = 10:5:0.5 → EtOAc:MeOH = 19:1) afforded the title compound as a slightly brown resin (85 mg, 0.18 mmol, 50% yield).

^{1}H-NMR (400 MHz, CDCl$_3$): δ / ppm = 7.15 (dd, *J* = 8.4, 2.2 Hz, 1H), 7.01 (d, *J* = 2.1 Hz, 1H), 6.76 (d, *J* = 8.3 Hz, 1H), 6.62 (s, 2H), 5.13 (td, *J* = 10.1, 9.6, 2.9 Hz, 1H), 4.77 (s, 2H), 3.38 (t, *J* = 6.2 Hz, 2H), 3.34 (t, *J* = 6.5 Hz, 2H), 3.09 – 2.83 (m, 4H), 1.79 1.69 (m, 2H), 1.66 – 1.48 (m, 12H), 1.37 1.29 (m, 4H).

^{13}C-NMR (100 MHz, CDCl$_3$): δ / ppm = 151.1, 142.3, 132.6, 128.1 (t, *J* = 23.1 Hz, 2C), 127.8 (t, *J* = 23.7 Hz, 2C), 125.8, 125.2 (t, *J* = 24.0 Hz), 122.4, 119.6, 117.3, 99.7, 70.8, 70.7, 69.4, 60.9, 55.6, 48.7, 35.0 (quintet, *J* = 19.2 Hz), 29.6, 29.4, 28.0, 26.8, 26.8, 25.9, 24.9, 24.7.

IR (Diamond-ATR, neat): $\tilde{\nu}$ / cm^{-1} = 3332, 2994, 2937, 2859, 2798, 2248, 1620, 1594, 1500, 1454, 1384, 1374, 1356, 1263, 1202, 1144, 1117, 1063, 957, 908, 871, 823, 796, 727, 696.

MS (EI, 70 eV): *m/z* (%) = 270 (19), 269 (100), 135 (16), 112 (21), 98 (29), 97 (19), 96 (12), 55 (11), 44 (24).

HRMS (EI-orbitrap): *m/z:* [M + H$^+$] calc. for [$C_{28}H_{35}D_7NO_4^+$]: 463.3548; found: 463.3580.

[9] L. Jiang, C. Lin, Y. Qiu, X. Quan, J. Zhu, H. Shi, *J. Chem. Res.* **2016**, *40*, 564 – 566.

Salmeterol-d_7 (6)

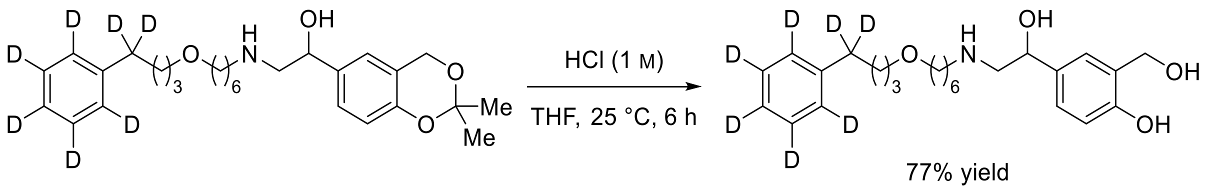

According to the literature,[10] HCl (1.0 M, 0.5 mL) was added to a mixture of 1-(2,2-dimethyl-4H-benzo[*d*][1,3]dioxin-6-yl)-((6-(4-(phenyl-d_5)butoxy-4,4-d_2)hexyl)amino)ethan-1-ol (80 mg, 0.17 mmol, 1.0 equiv) in THF (1 mL). The reaction mixture was stirred for 6 h at 25 °C. The mixture was extracted with EtOAc (3x30 mL). The combined organic layers were dried over anhydrous $MgSO_4$. The title compound **6** was obtained as a colorless solid (55 mg, 0.13 mmol, 77% yield).

^{1}H-NMR (400 MHz, $CDCl_3$): δ / ppm = 6.98 (dd, *J* = 8.2, 2.3 Hz, 1H), 6.87 (d, *J* = 2.2 Hz, 1H), 6.70 (d, *J* = 8.2 Hz, 1H), 5.13 (bs, 4H), 4.61 (s, 2H), 4.48 (t, *J* = 6.3 Hz, 1H), 3.40 (t, *J* = 6.2 Hz, 2H), 3.36 (t, *J* = 6.6 Hz, 2H), 2.70 – 2.59 (m, 2H), 2.59 – 2.46 (m, 2H), 1.68 – 1.57 (m, 4H), 1.56 – 1.48 (m, 2H), 1.47 – 1.38 (m, 2H), 1.33 – 1.22 (m, 4H).

^{13}C-NMR (100 MHz, $CDCl_3$): δ / ppm = 155.8, 142.3, 133.5, 128.1 (t, *J* = 22.6 Hz, 2C), 127.9 (t, *J* = 23.8 Hz, 2C), 126.5, 126.3, 125.9, 125.3 (t, *J* = 23.5 Hz), 116.4, 71.4, 71.0, 70.9, 63.0, 56.6, 49.4, 35.0 (quintet, *J* = 19.8 Hz), 29.7, 29.4, 29.4, 28.0, 27.2, 26.1.

IR (Diamond-ATR, neat): $\tilde{\nu}$ / cm^{-1} = 3292, 2927, 2895, 2860, 1612, 1508, 1456, 1447, 1375, 1263, 1148, 1113, 1038, 825.

HRMS (FTMS-ESI): *m/z:* [M+H$^+$] calc. for [$C_{25}H_{31}D_7NO_4^+$]: 423.32347; found 423.32346.
[M–H$^+$] calc. for [$C_{25}H_{29}D_7NO_4^-$]: 421.30892; found 421.30862.

[10] L. Jiang, C. Lin, Y. Qiu, X. Quan, J. Zhu, H. Shi, *J. Chem. Res.* **2016**, *40*, 564 – 566.

Synthesis of the SLAB 4-tridecylbenzenesulfonic acid (8)

tridecylbenzene (13t)

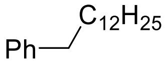

According to **TP3**, a solution of 3-(chloromethyl)heptane (**2**, 0.2 M, 2.0 equiv) in *n*-hexane was prepared. The solution of **2** was pumped through the activated sodium packed-bed reactor (see **TP1**) by pump A (flow rate: 2.0 mL/min) into the precooling loop (V_{pre2} = 0.35 mL) at 25 °C. Subsequently upon reaching the steady state, it was injected (12 min) into a flask charged with TMEDA (4.80 mmol, 1.7 equiv) in toluene (**3b**, 6.0 mL) and the resulting mixture was stirred at 25 °C for 2 h. The reaction flask was cooled to –20 °C and a solution of 1-chlorododecane (**12a**, 573 mg, 2.80 mmol, 1.0 equiv) in THF (7.0 mL) was added The reaction mixture was stirred at –20 °C for 10 min before it was allowed to warm to 25 °C and stirred overnight. Sat. *aq.* NH_4Cl solution was added for quenching the reaction mixture. The aqueous layer was extracted three times with EtOAc (3×30 mL) and the combined organic layers were dried over anhydrous $MgSO_4$ and filtrated. Removal of the solvent afforded the title compound **13t** as a colorless oil (721 mg, 2.77 mmol, 99% yield) without further purification.

^{1}H-NMR (400 MHz, $CDCl_3$): δ / ppm = 7.38 – 7.31 (m, 2H), 7.25 (d, *J* = 7.3 Hz, 3H), 2.72 – 2.63 (m, 2H), 1.74 – 1.63 (m, 2H), 1.42 – 1.31 (m, 20H), 0.96 (t, *J* = 6.9 Hz, 3H).

^{13}C-NMR (100 MHz, $CDCl_3$): δ / ppm = 143.1, 128.5 (2C), 128.4 (2C), 125.7, 36.2, 32.1, 31.7, 29.9, 29.9 (2C), 29.8, 29.8, 29.7, 29.5, 29.5, 22.9, 14.3.

IR (Diamond-ATR, neat): $\tilde{\nu}$ / cm^{-1} = 2955, 2922, 2853, 1496, 1466, 1454, 744, 722, 696.

MS (EI, 70 eV): *m/z* (%) = 133 (12), 105 (10), 92 (93), 91 (100).

HRMS (EI-orbitrap): *m/z:* [M] calc. for [$C_{19}H_{32}$]: 260.2504; found 260.2499.

4-tridecylbenzenesulfonic acid (8)

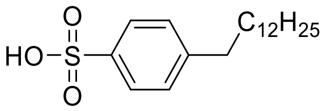

According to the literature[11], a solution of tridecylbenzene (**13t**, 500 mg, 1.92 mmol, 1.0 equiv) in $CHCl_3$ (5.0 mL) was prepared. Chlorosulfonic acid (**27**, 0.15 mL, 2.31 mmol, 1.2 equiv) was added at 0 °C and the mixture was stirred at the same temperatura for 3 h. Solvents were removed *in vacuo* and the obtained crude was dried over night under high vacuum. The residues were washed with small amounts of toluene and the solid residue was dried under vacuum to give the title compound **8** as grey solid (520 mg, 1.52 mmol, 80%).

^{1}H-NMR (400 MHz, MeOD): δ / ppm = 7.73 (d, *J* = 7.9 Hz, 2H), 7.26 (d, *J* = 7.9 Hz, 2H), 2.65 (t, *J* = 7.6 Hz, 2H), 1.62 (p, *J* = 6.4 Hz, 2H), 1.30 (m, 20H), 0.90 (t, *J* = 6.5 Hz, 3H).
^{13}C-NMR (100 MHz, MeOD): δ / ppm = 147.0, 143.1, 129.4 (2C), 127.0 (2C), 36.6, 33.1, 32.5, 30.8, 30.7 (3C), 30.7, 30.6, 30.5, 30.3, 23.7, 14.5.
IR (Diamond-ATR, neat): $\tilde{\nu}$ / cm^{-1} = 3389, 2956, 2922, 2872, 2852, 1716, 1126, 1035, 1008, 694.
MS (EI, 70 eV): *m/z* (%) = 340 (24), 292 (14), 172 (22), 139 (10), 123 (26), 92 (27), 91 (44), 71 (10), 61 (14), 57 (18), 55 (11), 45 (14), 43 (22), 43 (100), 41 (14).
HRMS (EI-orbitrap): *m/z:* [M] calc. for [$C_{19}H_{32}O_3S$]: 340.2072; found 340.2066.
m.p. (°C): 59.2 – 62.2.

[11] P. K. Bhowmik, A. Chang, J. Kim, E. J. Dizon, R. C. G. Principe, H. Han, *Crystals*, **2019**, *9*, 77.

Stereo- amd chemoselectivity studies on the Wurtz-type coupling of benzhydrylsodium (4n)

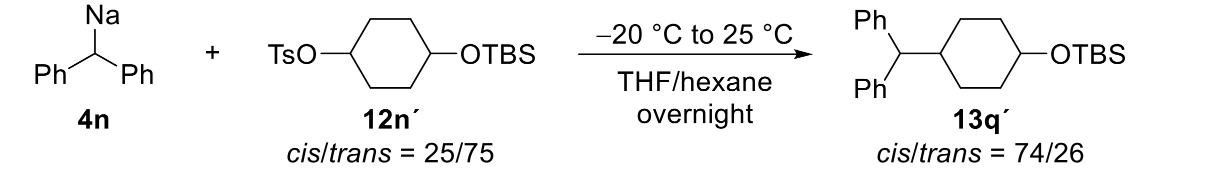

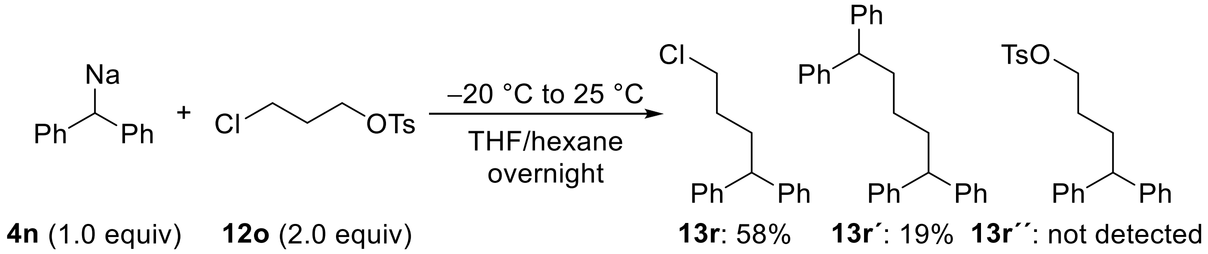

To exclude the possibility that the inversion of the stereochemistry during the reaction towards **13q** is due to an energetically favorable formation of the *cis*-product, the sequence was repeated using a mixture of *cis* and *trans* (25/75) electrophile **12n´**. As expected the stereochemistry was indeed inverted and the product **13q´** was obtained as a diastereomeric mixture (*cis/trans* = 74/26). Furthermore, we treated **4n** (1.0 equiv) with 3-chloropropyl tosylate (**12o**, 2.0 equiv). Substitution of the tosylate was the major pathway producing the alkylchloride **13r** in 58% yield. Whereas the twofold substitution took place to smaller degree (**13r´**, 19%). The chlorine-substituted product **13r´´** was not detected.

((4-benzhydrylcyclohexyl)oxy)(tert-butyl)dimethylsilane (13q´)

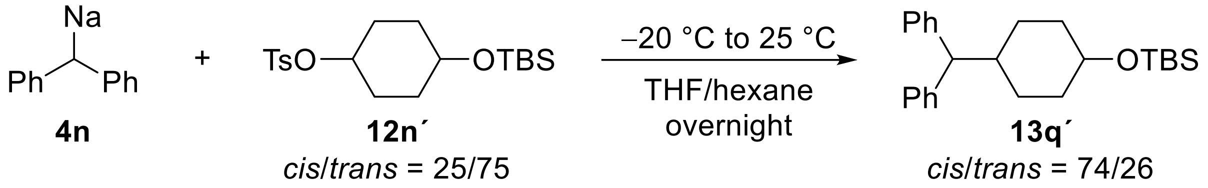

According to **TP3**, a solution of 3-(chloromethyl)heptane (**2**, 0.2 M, 2.0 equiv) in *n*-hexane was prepared. The solution of **2** was pumped through the activated sodium packed-bed reactor (see **TP1**) by pump A (flow rate: 2.0 mL/min) into the precooling loop (V_{pre2} = 0.35 mL) at 25 °C. Subsequently upon reaching the steady state, it was injected (2 min) into a flask charged with TMEDA (0.80 mmol, 2.0 equiv) in diphenylmethane (**3n**, 1.0 mL) and the resulting mixture was stirred at 25 °C for 30 min. The reaction flask was cooled to –20 °C and a solution of 4-((tert-butyldimethylsilyl)oxy)cyclohexyl 4-methylbenzenesulfonate (**12n**, 154 mg, 0.40 mmol, 1.0 equiv, *cis/trans* : 25/75) in THF (1.0 mL) was added. The reaction mixture was stirred at –20 °C for 10 min followed by another 30 min at 25 °C, before sat. *aq.* NH_4Cl solution was added for quenching the reaction mixture. The aqueous layer was extracted three times with EtOAc (3×30 mL) and the combined organic layers were dried over anhydrous $MgSO_4$ and filtrated. After removal of the solvent, flash column chromatographical

purification (silica gel, pentane:EtOAc = 100:0 → 98:2) afforded the title compound **13q´** as a colorless oil (*cis*/*trans* : 74/26)[12].

Major:

^{1}H-NMR (400 MHz, CDCl$_3$): δ / ppm = 7.37 – 7.32 (m, 4H), 7.31 – 7.26 (m, 4H), 7.19 – 7.13 (m, 2H), 3.95 (tt, *J* = 4.7, 2.1 Hz, 1H), 3.65 (d, *J* = 11.2 Hz, 1H), 2.27 – 2.10 (m, 1H), 1.71 – 1.59 (m, 2H), 1.51 – 1.31 (m, 6H), 0.93 (s, 9H), 0.05 (s, 6H).

^{13}C-NMR (100 MHz, CDCl$_3$): δ / ppm = 144.6 (2C), 128.5 (4C), 128.2 (4C), 126.1 (2C), 67.2, 58.4, 40.4, 33.3 (2C), 26.0 (3C), 25.9 (2C), 18.3, -4.7 (2C).

Minor:

^{1}H-NMR (400 MHz, CDCl$_3$): δ / ppm = 7.37 – 7.32 (m, 4H), 7.31 – 7.26 (m, 4H), 7.19 – 7.13 (m, 2H), 3.65 – 3.52 (m, 1H), 3.45 (d, *J* = 10.7 Hz, 1H), 2.15 – 2.06 (m, 1H), 1.86 – 1.82 (m, 2H), 1.51 – 1.31 (m, 6H), 0.91 (s, 9H), 0.07 (s, 6H).

^{13}C-NMR (100 MHz, CDCl$_3$): δ / ppm = 144.5 (2C), 128.6 (4C), 128.1 (4C), 126.2 (2C), 72.0, 59.3, 40.5, 35.9 (2C), 26.0 (3C), 26.1 (2C), 18.4, -4.43 (2C).

Mixture:

IR (Diamond-ATR, neat): $\tilde{\nu}$ / cm^{-1} = 3062, 3027, 2928, 2884, 2856, 1598, 1494, 1472, 1462, 1450, 1376, 1360, 1250, 1181, 1155, 1090, 1049, 1022, 1005, 964, 939, 912, 886, 863, 832, 772, 752, 743, 700, 675.

MS (EI, 70 eV): *m/z* (%) = 324 (10), 323 (41), 247, (17), 169 (22), 168 (13), 167 (100), 165 (34), 152 (20), 143 (25), 117 (12), 75 (39).

HRMS (EI-orbitrap): *m/z:* [M – CH$_3$] calc. for [$C_{24}H_{33}OSi$]: 365.2301; found 365.2295.

[12] Ratio was determined by ^{1}H-NMR.

((4-benzhydrylcyclohexyl)oxy)(tert-butyl)dimethylsilane (13q´)

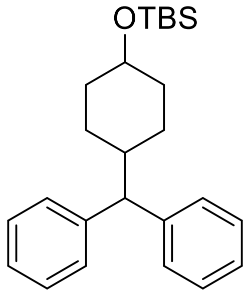

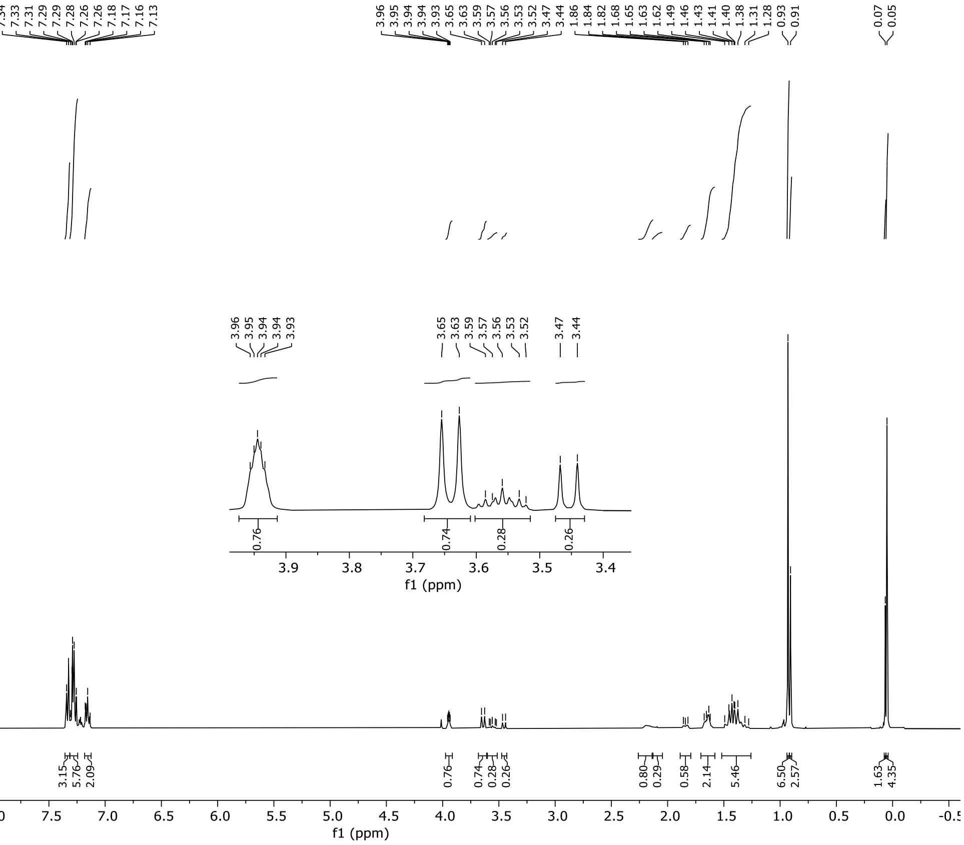

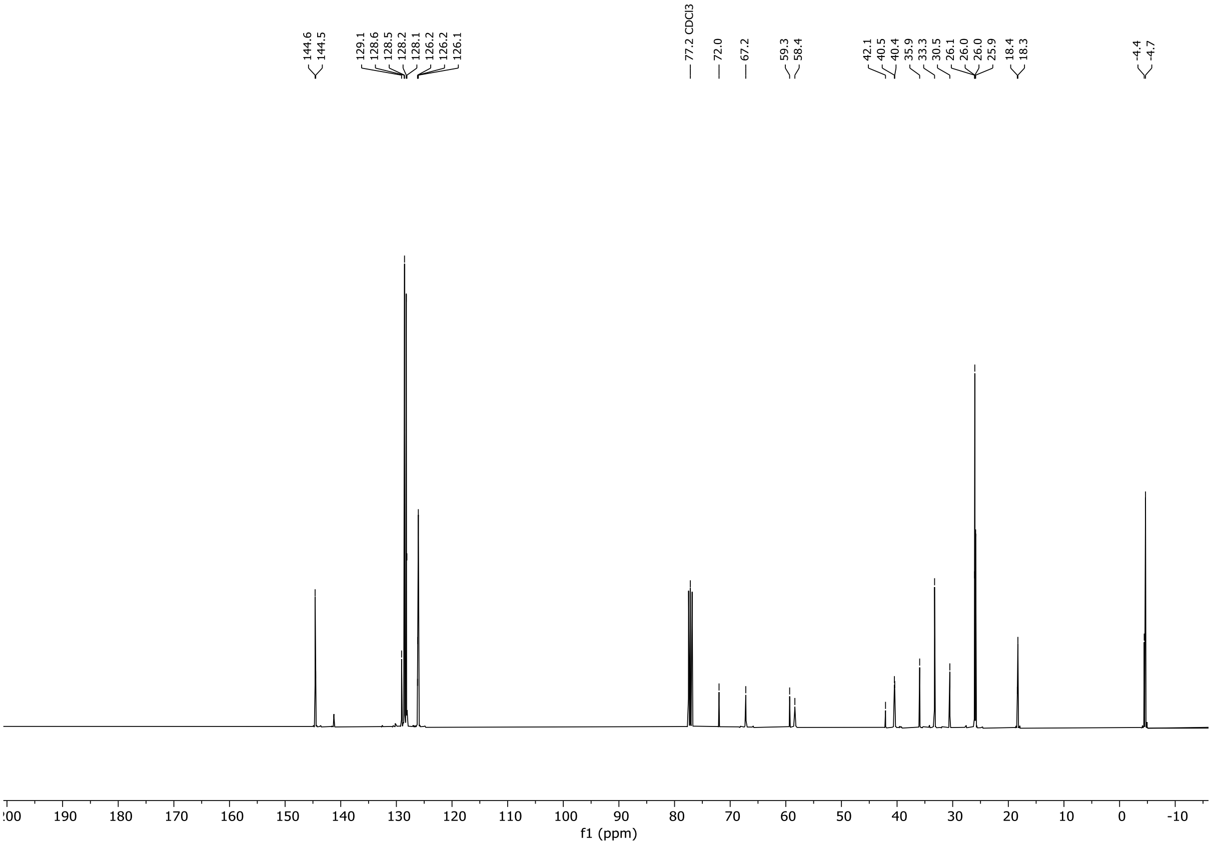

(4-chlorobutane-1,1-diyl)dibenzene (13r)

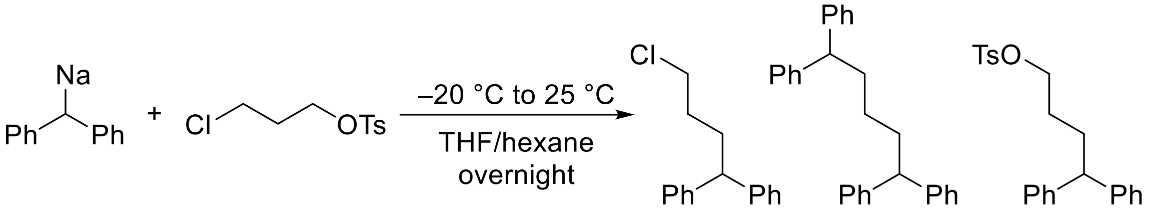

4n (1.0 equiv) **12o** (2.0 equiv) **13r**: 58% **13r´**: 19% **13r´´**: not detected

According to **TP4**, A solution of 3-(chloromethyl)heptane (**2**, 0.2 M, 2.0 equiv) in *n*-hexane was prepared. The solution of **2** was pumped through the activated sodium packed-bed reactor (see **TP1**) by pump A (flow rate: 2.0 mL/min) into the precooling loop (V_{pre2} = 0.35 mL) at 25 °C. Subsequently upon reaching the steady state, it was injected for 2 min into a flask charged with a solution of TMEDA (0.80 mmol, 2.0 equiv) and diphenylmethane (**3n**, 67 mg, 0.40 mmol, 1.0 equiv) in hexane (2.0 mL) the mixture was stirred for 30 min at 25 °C. Before it was cooled to –40 °C and a solution of 3-chloropropyl 4-methylbenzenesulfonate (**12o**, 199 mg, 0.80 mmol, 2.0 equiv) in THF (2.0 mL) was added. The reaction mixture was stirred at –40 °C for 1 h followed by another 1 h at 25 °C, before sat. *aq.* NH_4Cl solution was added for quenching the reaction mixture. The aqueous layer was extracted three times with EtOAc (3×30 mL) and the combined organic layers were dried over anhydrous $MgSO_4$ and filtrated. After removal of the solvent, flash column chromatographical purification (silica gel, pentane:EtOAc = 100:0 → 98:2) afforded the title compounds as a mixture of **13r**:**13r´** = 1.0:0.3[13] (total yield: 84 mg, **13r**: 0.23 mmol, 58% yield; **13r´:** 0.08 mmol, 19 % yield corresponding to a total conversion of **3n** of 96%. **13r´´** was not detected).

[13] Ratio was determined by ^{1}H-NMR.

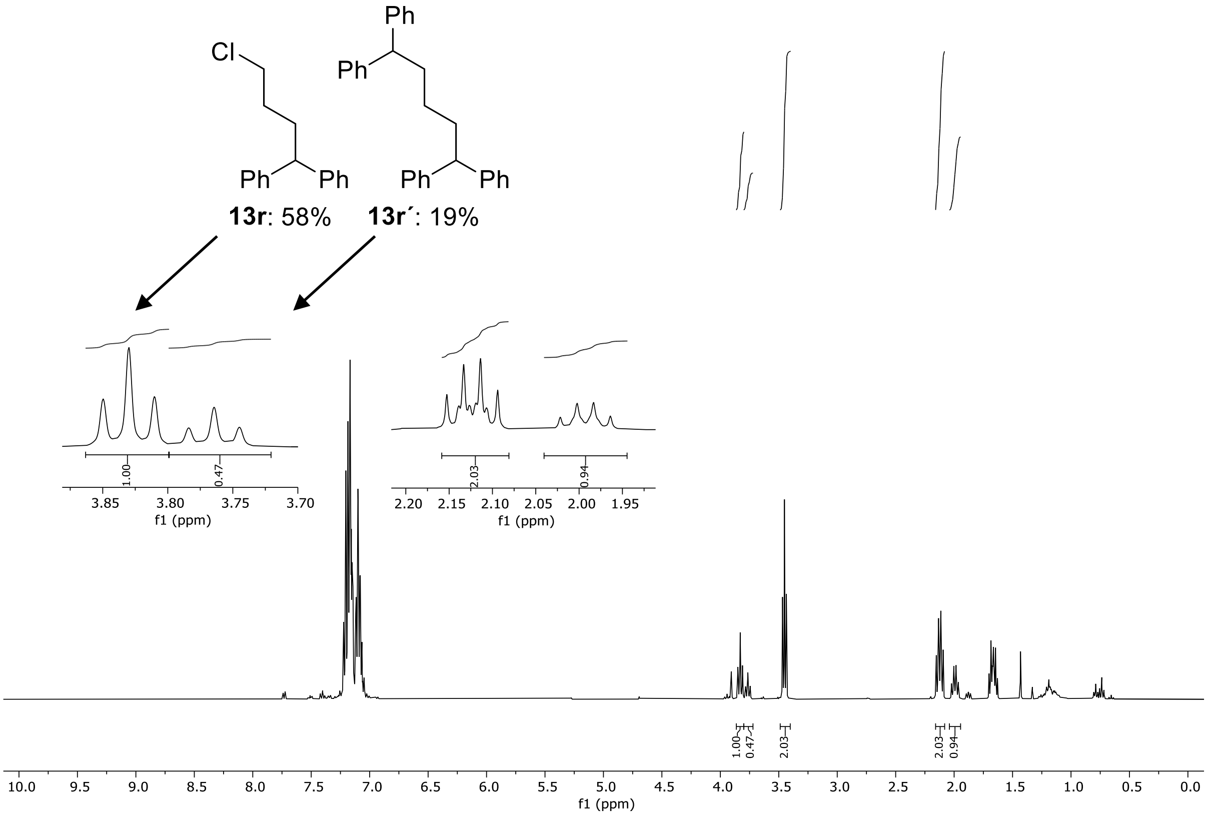

Cl
Ph
Ph
Ph
Ph
Ph
Ph
13r: 58%
13r´: 19%
1.00
0.47
3.85 3.80 3.75 3.70
f1 (ppm)
2.03
0.94
2.20 2.15 2.10 2.05 2.00 1.95
f1 (ppm)
1.00
0.47
2.03
2.03
0.94
10.0 9.5 9.0 8.5 8.0 7.5 7.0 6.5 6.0 5.5 5.0 4.5 4.0 3.5 3.0 2.5 2.0 1.5 1.0 0.5 0.0
f1 (ppm)

Temperature influence on the metalation site of 2-isopropyl-4-methylthiazole (14b)

A solution of 3-(chloromethyl)heptane (**2**, 0.2 M, 2.0 equiv) in *n*-hexane was prepared. The solution of **2** was pumped through the activated sodium packed-bed reactor (see **TP1**) by pump A (flow rate: 2.0 mL/min) into the precooling loop (V_{pre2} = 0.35 mL) at 25 °C. Subsequently upon reaching the steady state, it was injected for 2 min into a flask charged with a solution of TMEDA (0.80 mmol, 2.0 equiv) and 2-isopropyl-4-methylthiazole (**14b**, 56 mg, 0.40 mmol, 1.0 equiv) in hexane (2.0 mL) the mixture was stirred for 1 h at the corresponding temperature (T = –40 to 25 °C). Before a solution of adamantanone (**9k**, 120 mg, 0.80 mmol, 2.0 equiv) in THF (2.0 mL) was added. The reaction mixture was stirred at the corresponding temperature for 2 h before an aliquot was taken and analysed by GC.

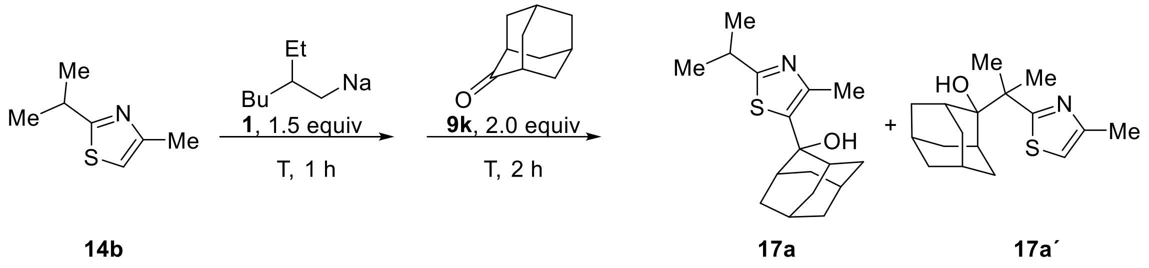

Table SI 6: Temperature screening for the sodiation of 2-isopropyl-4-methylthiazole (14b).

Entry	T [°C]	Conversion (**14b**) [%]	Ratio **17a/17a´**[a]
1	–40	93	99/1
2	–20	92	90/10
3	0	87	70/30
4	25	88	42/58

[a] Ratio was determined comparing the integrated area under the curve after GC-analysis.

A solution of 3-(chloromethyl)heptane (**2**, 0.2 M, 2.0 equiv) in *n*-hexane was prepared. The solution of **2** was pumped through the activated sodium packed-bed reactor (see **TP1**) by pump A (flow rate: 2.0 mL/min) into the precooling loop (V_{pre2} = 0.35 mL) at 25 °C. Subsequently upon reaching the steady state, it was injected for 2 min into a flask charged with a solution of TMEDA (0.80 mmol, 2.0 equiv) and 2-isopropyl-4-methylthiazole (**14b**, 56 mg, 0.40 mmol, 1.0 equiv) in hexane (2.0 mL) the mixture was stirred for 1 h at the corresponding temperature (T = –40 to 25 °C). Before a solution of cyclohexene oxide (**9a**, 79 mg, 0.80 mmol, 2.0 equiv) in THF (2.0 mL) was added. The reaction mixture was stirred at the corresponding temperature for 2 h before an aliquot was taken and analysed by GC. A second aliquot was taken and analysed after stirring for 20 h at the corresponding temperature.

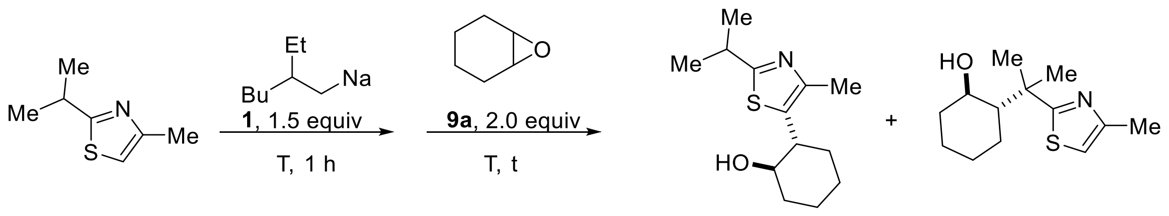

Table SI 7: Temperature screening for the sodiation of 2-isopropyl-4-methylthiazole (14b).

Entry	T [°C]	t [h]	Conversion (**14b**) [%]	Normalized GC-Yield **19a**[a]
1	–40	2	22	11
2	–20	2	38	41
3	0	2	78	87
4	25	2	93	47
5	–40	20	49	18
6	–20	20	98	71
7	0	20	90	100
8	25	20	94	46

[a]The largest integrated area under the curve corresponding to the product **19a** was normalized to 100% GC-yield the other integrals were adjusted accordingly.

Single crystal X-ray diffraction studies

***trans*-2-(naphthalen-1-ylmethyl)cyclohexan-1-ol (10v)**

Single crystals of compound **10v**, suitable for X-ray diffraction, were obtained by slow evaporation of DCM solution. The crystals were introduced into perfluorinated oil and a suitable single crystal was carefully mounted on the top of a thin glass wire. The X-ray intensity data were measured on a 'D8 Venture' system equipped with a 'Bruker D8 Venture TXS' 'rotating-anode X-ray tube' ('Mo Kα', λ = 0.71073 Å) and a 'multilayer mirror optics' monochromator.

Data collection[14], data reduction[15] and cell refinement[16] were performed with the Bruker specific software. Absorption correction using the multiscan method was applied. The structures were solved with SHELXS-97,[17] refined with SHELXL-97[18] and finally checked using PLATON.[19] Details for data collection and structure refinement are summarized in Table SI 8.

CCDC-2158223 contains supplementary crystallographic data for this compound. These data can be obtained free of charge from The Cambridge Crystallographic Data Centre *via* www.ccdc.cam.ac.uk/data_request/cif.

[14] Program package 'Bruker Instrument Service v3.0.21'.

[15] Program package 'SAINT V8.18C (Bruker AXS Inc., 2011)'.

[16] Program package 'APEX2 v2012.4-3 (Bruker AXS)'.

[17] Sheldrick, G. M. (1997) SHELXS-97: *Program for Crystal Structure Solution*, University of Göttingen, Germany

[18] Sheldrick, G. M. (1997) SHELXL-97: *Program for the Refinement of Crystal Structures*, University of Göttingen, Germany

[19] Spek, A. L. (1999) PLATON: *A Multipurpose Crystallographic Tool*, Utrecht University, Utrecht, The Netherlands

Table SI 8. Details for X-ray data collection and structure refinement for compound **10v**.

	**10v**
Empirical formula	$C_{17}H_{20}O$
Formula mass	240.33
T[K]	173(2)
Crystal size [mm]	0.16 × 0.04 × 0.02
Crystal description	colorless block
Crystal system	monoclinic
Space group	*P*21/*c*
a [Å]	11.7544(8)
b [Å]	5.1712(4)
c [Å]	22.3353(15)
α [°]	90.0
β [°]	99.832(2)
γ [°]	90.0
V [$Å^3$]	1337.70(16)
Z	4
$\rho_{calcd.}$ [g cm^{-3}]	1.193
μ [mm^{-1}]	0.072
F(000)	520
Θ range [°]	3.52 – 25.24
Index ranges	$-15 \leq h \leq 15$
	$-6 \leq k \leq 6$
	$-28 \leq l \leq 28$
Reflns. collected	37157
Reflns. obsd.	2523
Reflns. unique	3062 (R_{int} = 0.0598)
R_1, wR_2 (2σ data)	0.0509, 0.1078
R_1, wR_2 (all data)	0.0651, 0.1142
GOOF on F^2	1.093
Peak/hole [e $Å^{-3}$]	0.216 / -0.228

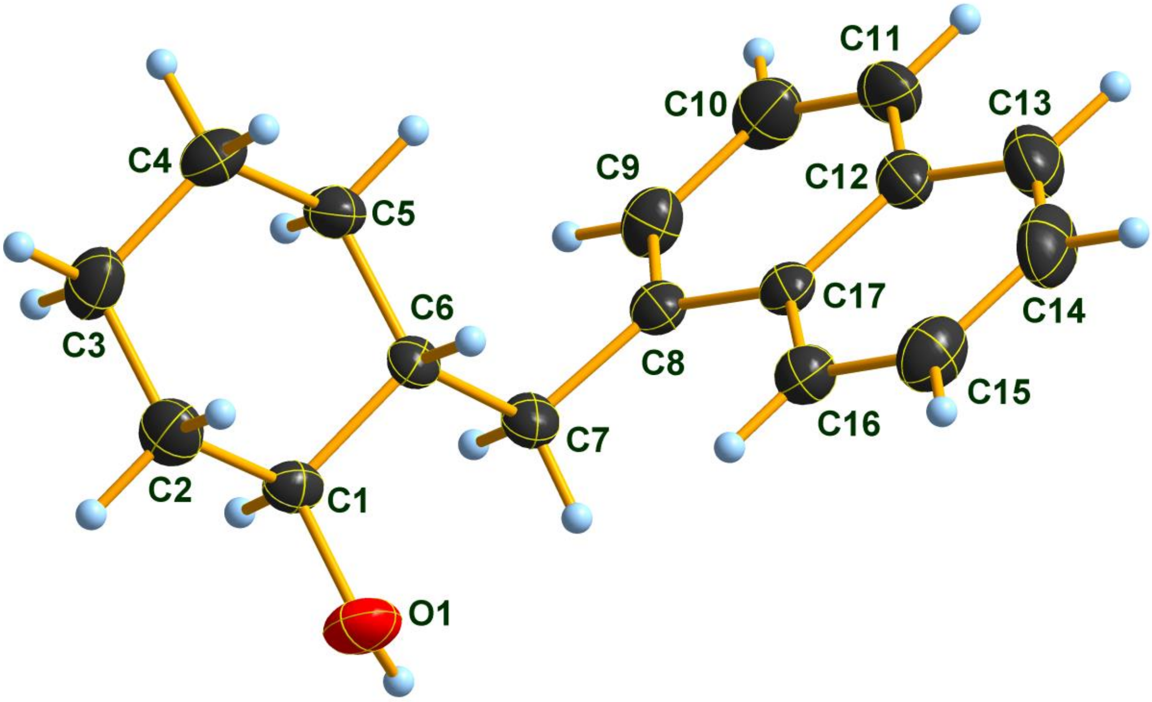

Figure 5. Molecular structure of compound **10v** in the crystal. DIAMOND[20] representation; thermal ellipsoids are drawn at 50 % probability level. The hydrogen atom of the OH group is disordered over two positions; only the more strongly occupied position is shown.

Table SI 9. Selected bond lengths (Å) of compound **10v**.

O1 – C1	1.431(2)	C16 – C15	1.367(2)
C1 – C2	1.519(2)	C8 – C9	1.370(2)
C1 – C6	1.529(2)	C2 – C3	1.522(2)
C6 – C5	1.530(2)	C11 – C10	1.351(2)
C6 – C7	1.540(2)	C11 – C12	1.418(2)
C5 – C4	1.521(2)	C13 – C14	1.354(3)
C4 – C3	1.520(2)	C13 – C12	1.414(2)
C17 – C16	1.422(2)	C9 – C10	1.407(2)
C17 – C12	1.427(2)	C15 – C14	1.409(2)
C17 – C8	1.429(2)	C7 – C8	1.509(2)

Table SI 10. Selected bond angles (°) of compound **10v**.

O1 – C1 – C2	109.2(1)	C9 – C8 – C17	119.0(1)
O1 – C1 – C6	111.7(1)	C9 – C8 – C7	119.8(1)

[20] DIAMOND, Crystal Impact GbR., Version 3.2i.

C2 – C1 – C6	112.5(1)	C17 – C8 – C7	121.2(1)
C1 – C6 – C5	110.3(1)	C1 – C2 – C3	111.3(1)
C1 – C6 – C7	110.6(1)	C10 – C11 – C12	120.8(1)
C5 – C6 – C7	111.9(1)	C14 – C13 – C12	121.2(2)
C4 – C5 – C6	112.6(1)	C13 – C12 – C11	121.7(1)
C3 – C4 – C5	110.8(1)	C13 – C12 – C17	119.1(1)
C16 – C17 – C12	118.0(1)	C11 – C12 – C17	119.1(1)
C16 – C17 – C8	123.0(1)	C8 – C9 – C10	121.8(2)
C12 – C17 – C8	119.0(1)	C11 – C10 – C9	120.3(2)
C8 – C7 – C6	114.6(1)	C4 – C3 – C2	111.1(1)
C15 – C16 – C17	121.1(1)	C16 – C15 – C14	120.3(2)
C13 – C14 – C15	120.3(2)		

Table SI 11. Selected torsion angles (°) of compound **10v**.

O1 – C1 – C6 – C5	-176.3(1)	C6 – C1 – C2 – C3	55.0(2)
C2 – C1 – C6 – C5	-53.1(2)	C14 – C13 – C12 – C11	-179.7(2)
O1 – C1 – C6 – C7	59.4(2)	C14 – C13 – C12 – C17	-0.3(2)
C2 – C1 – C6 – C7	-177.5(1)	C10 – C11 – C12 – C13	178.3(2)
C1 – C6 – C5 – C4	53.6(2)	C10 – C11 – C12 – C17	-1.1(2)
C7 – C6 – C5 – C4	177.1(1)	C16 – C17 – C12 – C13	0.3(2)
C6 – C5 – C4 – C3	-55.6(2)	C8 – C17 – C12 – C13	-179.0(1)
C1 – C6 – C7 – C8	-177.7(1)	C16 – C17 – C12 – C11	179.8(1)
C5 – C6 – C7 – C8	59.0(2)	C8 – C17 – C12 – C11	0.5(2)
C12 – C17 – C16 – C15	-0.1(2)	C17 – C8 – C9 – C10	-1.5(2)
C8 – C17 – C16 – C15	179.2(1)	C7 – C8 – C9 – C10	178.8(1)
C16 – C17 – C8 – C9	-178.5(1)	C12 – C11 – C10 – C9	0.5(2)
C12 – C17 – C8 – C9	0.8(2)	C8 – C9 – C10 – C11	0.8(2)
C16 – C17 – C8 – C7	1.2(2)	C5 – C4 – C3 – C2	56.1(2)
C12 – C17 – C8 – C7	-179.5(1)	C1 – C2 – C3 – C4	-56.0(2)
C6 – C7 – C8 – C9	-102.2(2)	C17 – C16 – C15 – C14	-0.2(2)
C6 – C7 – C8 – C17	78.1(2)	C12 – C13 – C14 – C15	0.0(3)
O1 – C1 – C2 – C3	179.6(1)	C16 – C15 – C14 – C13	0.2(2)

(((1*R*,2*S*)-2-methoxycyclohexyl)methylene)dibenzene (13o)

Single crystals of compound **13o**, suitable for X-ray diffraction, were obtained by slow evaporation of DCM solution. The crystals were introduced into perfluorinated oil and a suitable single crystal was carefully mounted on the top of a thin glass wire. Data collection was performed with an Oxford Xcalibur 3 diffractometer equipped with a Spellman generator (50 kV, 40 mA) and a Kappa CCD detector, operating with Mo-K_{α} radiation (λ = 0.71071 Å).

Data collection and data reduction were performed with the CrysAlisPro software.[21] Absorption correction using the multiscan method[22] was applied. The structures were solved with SHELXS-97,[23] refined with SHELXL-97[24] and finally checked using PLATON.[25] Details for data collection and structure refinement are summarized in Table SI 12.

CCDC-2158222 contains supplementary crystallographic data for this compound. These data can be obtained free of charge from The Cambridge Crystallographic Data Centre *via* www.ccdc.cam.ac.uk/data_request/cif.

[21] Program package 'CrysAlisPro 1.171.40.84a (Rigaku OD, 2020)'.
[22] Program package 'CrysAlisPro 1.171.40.84a (Rigaku OD, 2020)'.
[23] Sheldrick, G. M. (1997) SHELXS-97: *Program for Crystal Structure Solution*, University of Göttingen, Germany.
[24] Sheldrick, G. M. (1997) SHELXL-97: *Program for the Refinement of Crystal Structures*, University of Göttingen, Germany.
[25] Spek, A. L. (1999) PLATON: *A Multipurpose Crystallographic Tool*, Utrecht University, Utrecht, The Netherlands.

Table SI 12. Details for X-ray data collection and structure refinement for compound **13o**.

	**13o**
Empirical formula	$C_{20}H_{24}O$
Formula mass	280.39
T[K]	123(2)
Crystal size [mm]	0.40 × 0.30 × 0.25
Crystal description	colorless block
Crystal system	Orthorhombic
Space group	*P*212121
a [Å]	5.9937(2)
b [Å]	14.6073(6)
c [Å]	18.3043(7)
α [°]	90.0
β [°]	90.0
γ [°]	90.0
V [Å^3]	1602.57(10)
Z	4
$\rho_{calcd.}$ [g cm^{-3}]	1.162
μ [mm^{-1}]	0.069
F(000)	608
Θ range [°]	2.23 – 25.24
Index ranges	$-7 \leq h \leq 7$
	$-19 \leq k \leq 19$
	$-24 \leq l \leq 24$
Reflns. collected	27266
Reflns. obsd.	3446
Reflns. unique	3947 (R_{int} = 0.0451)
R_1, wR_2 (2σ data)	0.0443, 0.0979
R_1, wR_2 (all data)	0.0537, 0.1029
GOOF on F^2	1.040
Peak/hole [e Å^{-3}]	0.307 / -0.152

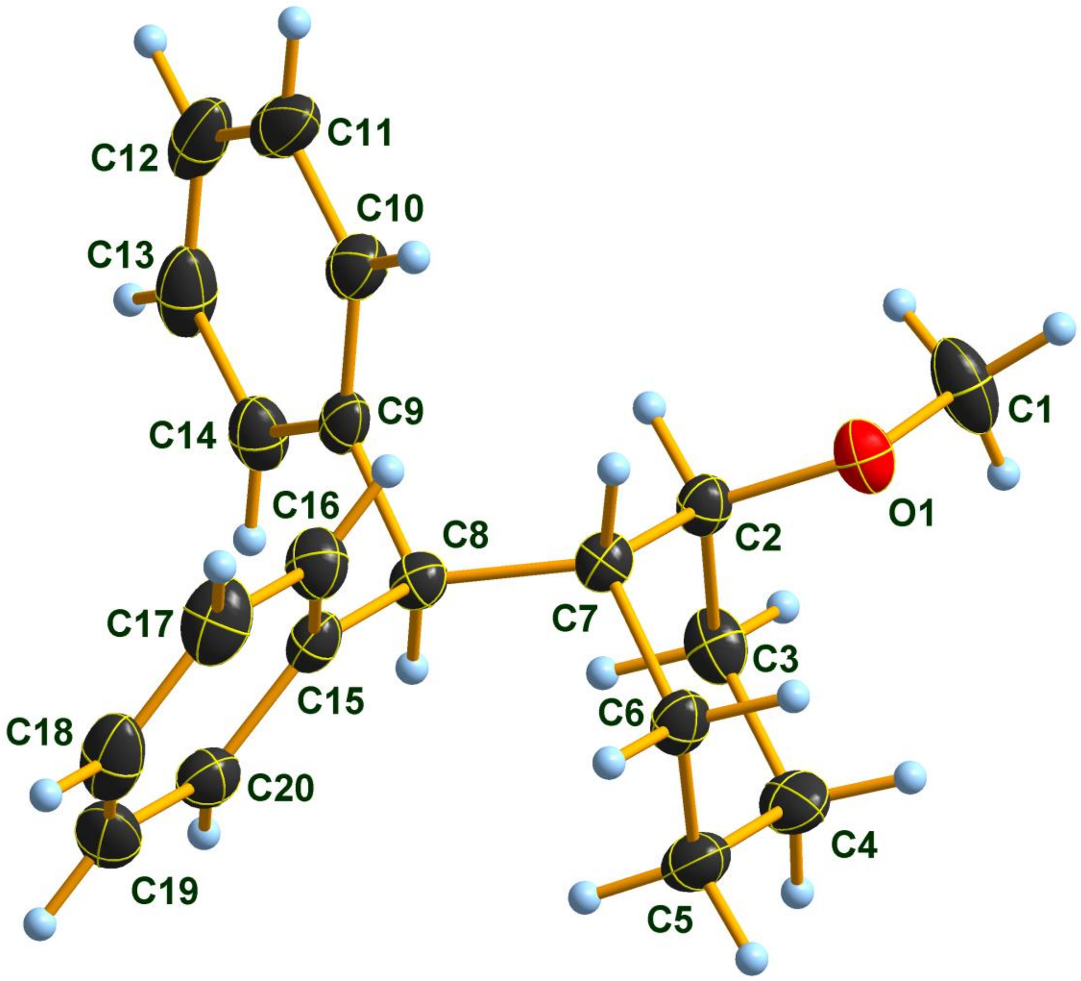

Figure 6. Molecular structure of compound **13o** in the crystal. DIAMOND[26] representation; thermal ellipsoids are drawn at 50 % probability level.

Table SI 13. Selected bond lengths (Å) of compound **13o**.

O1 – C1	1.427(3)	C16 – C17	1.394(3)
O1 – C2	1.435(2)	C10 – C11	1.391(3)
C6 – C5	1.526(3)	C20 – C19	1.391(3)
C6 – C7	1.541(3)	C19 – C18	1.373(4)
C2 – C3	1.530(3)	C18 – C17	1.389(4)
C2 – C7	1.538(3)	C11 – C12	1.382(4)
C8 – C15	1.528(3)	C13 – C12	1.378(4)
C8 – C9	1.530(3)	C13 – C14	1.397(3)
C8 – C7	1.549(3)	C15 – C16	1.394(3)
C9 – C10	1.392(3)	C15 – C20	1.394(3)

[26] DIAMOND, Crystal Impact GbR., Version 3.2i.

C9 – C14	1.394(3)	C3 – C4	1.527(3)
C5 – C4	1.532(3)		

Table SI 14. Selected bond angles (°) of compound **13o**.

C1 – O1 – C2	112.3(2)	C17 – C16 – C15	120.8(2)
C5 – C6 – C7	112.7(2)	C11 – C10 – C9	120.6(2)
O1 – C2 – C3	111.8(2)	C19 – C20 – C15	121.0(2)
O1 – C2 – C7	106.7(2)	C3 – C4 – C5	110.4(2)
C3 – C2 – C7	111.0(2)	C18 – C19 – C20	120.4(2)
C15 – C8 – C9	110.8(2)	C19 – C18 – C17	119.7(2)
C15 – C8 – C7	112.9(2)	C12 – C11 – C10	120.3(2)
C9 – C8 – C7	113.0(2)	C12 – C13 – C14	120.2(2)
C10 – C9 – C14	118.6(2)	C18 – C17 – C16	120.1(2)
C10 – C9 – C8	122.2(2)	C9 – C14 – C13	120.4(2)
C14 – C9 – C8	119.2(2)	C13 – C12 – C11	119.8(2)
C6 – C5 – C4	110.8(2)	C2 – C7 – C6	109.8(2)
C16 – C15 – C20	118.1(2)	C2 – C7 – C8	110.6(2)
C16 – C15 – C8	122.4(2)	C6 – C7 – C8	113.0(2)
C20 – C15 – C8	119.5(2)	C4 – C3 – C2	111.3(2)

Table SI 15. Selected torsion angles (°) of compound **13o**.

C1 – O1 – C2 – C3	64.2(2)	O1 – C2 – C3 – C4	61.2(2)
C1 – O1 – C2 – C7	-174.2(2)	C7 – C2 – C3 – C4	-57.8(2)
C15 – C8 – C9 – C10	74.8(2)	C20 – C15 – C16 – C17	0.1(3)
C7 – C8 – C9 – C10	-53.0(2)	C8 – C15 – C16 – C17	178.5(2)
C15 – C8 – C9 – C14	-104.2(2)	C14 – C9 – C10 – C11	0.2(3)
C7 – C8 – C9 – C14	128.0(2)	C8 – C9 – C10 – C11	-178.8(2)
C7 – C6 – C5 – C4	55.3(2)	C16 – C15 – C20 – C19	-0.1(3)
C9 – C8 – C15 – C16	-75.5(2)	C8 – C15 – C20 – C19	-178.7(2)
C7 – C8 – C15 – C16	52.3(2)	C2 – C3 – C4 – C5	57.5(2)
C9 – C8 – C15 – C20	102.9(2)	C6 – C5 – C4 – C3	-55.7(2)
C7 – C8 – C15 – C20	-129.3(2)	C15 – C20 – C19 – C18	0.1(3)
O1 – C2 – C7 – C6	-66.8(2)	C20 – C19 – C18 – C17	0.1(3)
C3 – C2 – C7 – C6	55.2(2)	C9 – C10 – C11 – C12	0.1(3)
O1 – C2 – C7 – C8	167.8(2)	C19 – C18 – C17 – C16	-0.1(4)
C3 – C2 – C7 – C8	-70.2(2)	C15 – C16 – C17 – C18	0.1(3)
C5 – C6 – C7 – C2	-54.7(2)	C10 – C9 – C14 – C13	-0.5(3)

C5 – C6 – C7 – C8	69.3(2)	C8 – C9 – C14 – C13	178.6(2)
C15 – C8 – C7 – C2	178.3(2)	C12 – C13 – C14 – C9	0.4(3)
C9 – C8 – C7 – C2	-55.1(2)	C14 – C13 – C12 – C11	-0.1(3)
C15 – C8 – C7 – C6	54.7(2)	C10 – C11 – C12 – C13	-0.2(3)
C9 – C8 – C7 – C6	-178.6(2)		

***cis*-4-(tert-butyl)cyclohexyl)methylene)dibenzene (13p)**

Single crystals of compound **13p**, suitable for X-ray diffraction, were obtained by slow evaporation of DCM solution. The crystals were introduced into perfluorinated oil and a suitable single crystal was carefully mounted on the top of a thin glass wire. Data collection was performed with an Oxford Xcalibur 3 diffractometer equipped with a Spellman generator (50 kV, 40 mA) and a Kappa CCD detector, operating with Mo-K_{α} radiation (λ = 0.71071 Å).

Data collection and data reduction were performed with the CrysAlisPro software.[27] Absorption correction using the multiscan method[28] was applied. The structures were solved with SHELXS-97,[29] refined with SHELXL-97[30] and finally checked using PLATON.[31] Details for data collection and structure refinement are summarized in Table SI 16.

CCDC-2158220 contains supplementary crystallographic data for this compound. These data can be obtained free of charge from The Cambridge Crystallographic Data Centre *via* www.ccdc.cam.ac.uk/data_request/cif.

[27] Program package 'CrysAlisPro 1.171.40.84a (Rigaku OD, 2020'.
[28] Program package 'CrysAlisPro 1.171.40.84a (Rigaku OD, 2020'.
[29] Sheldrick, G. M. (1997) SHELXS-97: *Program for Crystal Structure Solution*, University of Göttingen, Germany.
[30] Sheldrick, G. M. (1997) SHELXL-97: *Program for the Refinement of Crystal Structures*, University of Göttingen, Germany.
[31] Spek, A. L. (1999) PLATON: *A Multipurpose Crystallographic Tool*, Utrecht University, Utrecht, The Netherlands.

Table SI 16. Details for X-ray data collection and structure refinement for compound **13p**.

	**13p**
Empirical formula	$C_{23}H_{30}$
Formula mass	306.47
T[K]	123(2)
Crystal size [mm]	0.40 × 0.25 × 0.10
Crystal description	colorless block
Crystal system	monoclinic
Space group	*P*21/*m*
a [Å]	5.9437(3)
b [Å]	14.9446(7)
c [Å]	10.7329(6)
α [°]	90.0
β [°]	104.224(5)
γ [°]	90.0
V [$Å^3$]	924.13(8)
Z	2
$\rho_{calcd.}$ [g cm^{-3}]	1.101
μ [mm^{-1}]	0.061
F(000)	336
Θ range [°]	2.39 – 25.24
Index ranges	$-8 \le h \le 8$
	$-20 \le k \le 20$
	$-14 \le l \le 14$
Reflns. collected	16402
Reflns. obsd.	1926
Reflns. unique	2564 (R_{int} = 0.0460)
R_1, wR_2 (2σ data)	0.0493, 0.1194
R_1, wR_2 (all data)	0.0686, 0.1326
GOOF on F^2	1.030
Peak/hole [e $Å^{-3}$]	0.359 / -0.197

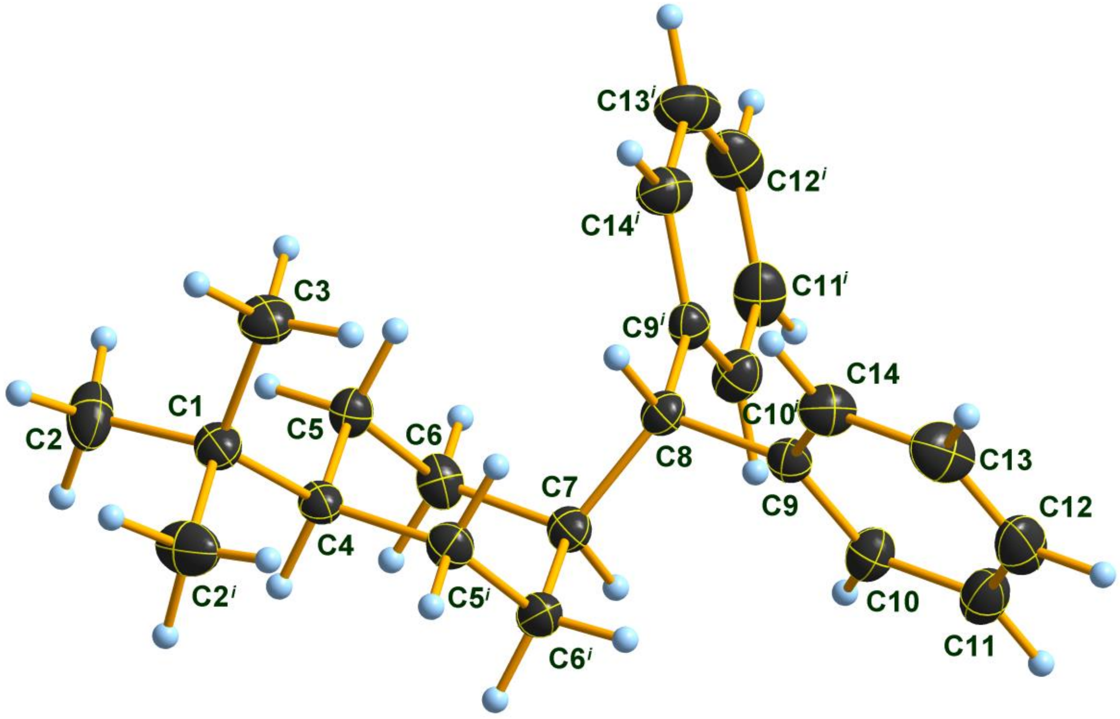

Figure 7. Molecular structure of compound **13p** in the crystal. DIAMOND[32] representation; thermal ellipsoids are drawn at 50 % probability level. Symmetry code: *i*: x, 0.5-y, z.

Table SI 17. Selected bond lengths (Å) of compound **13p**. Symmetry code: *i*: x, 0.5-y, z.

C1 – C2	1.531(2)	C12 – C13	1.377(2)
C1 – C2^{i}	1.531(2)	C12 – C11	1.382(2)
C1 – C3	1.533(2)	C8 – C9	1.523(1)
C1 – C4	1.551(2)	C8 – C9^{i}	1.523(1)
C5 – C6	1.532(2)	C8 – C7	1.556(2)
C5 – C4	1.533(1)	C14 – C9	1.392(2)
C6 – C7	1.533(1)	C14 – C13	1.392(2)
C10 – C11	1.388(2)	C9 – C10	1.395(2)

Table SI 18. Selected bond angles (°) of compound **13p**. Symmetry code: *i*: x, 0.5-y, z.

C2 – C1 – C2^{i}	108.0(1)	C13 – C12 – C11	119.6(1)
C2 – C1 – C3	108.7(1)	C9 – C8 – C9^{i}	112.0(1)
C2^{i} – C1 – C3	108.7(1)	C9 – C8 – C7	112.1(1)
C2 – C1 – C4	109.7(1)	C9^{i} – C8 – C7	112.1(1)
C2^{i} – C1 – C4	109.7(1)	C6^{i} – C7 – C6	109.3(1)

[32] DIAMOND, Crystal Impact GbR., Version 3.2i.

C3 – C1 – C4	112.0(1)	C6^i – C7 – C8	112.1(1)
C6 – C5 – C4	111.4(1)	C6 – C7 – C8	112.1(1)
C5^i – C4 – C5	108.8(1)	C9 – C14 – C13	120.9(1)
C5^i – C4 – C1	114.6(1)	C14 – C9 – C10	117.9(1)
C5 – C4 – C1	114.6(1)	C14 – C9 – C8	120.0(1)
C5 – C6 – C7	112.7(1)	C10 – C9 – C8	122.1(1)
C12 – C11 – C10	120.2(1)	C11 – C10 – C9	121.0(1)
C12 – C13 – C14	120.3(1)		

Table SI 19. Selected torsion angles (°) of compound **13p**. Symmetry code: *i*: x, 0.5-y, z.

C6 – C5 – C4 – C5^i	57.2(1)	C9 – C8 – C7 – C6	-54.9(2)
C6 – C5 – C4 – C1	-173.2(1)	C13 – C14 – C9 – C10	0.4(2)
C2 – C1 – C4 – C5^i	-175.8(1)	C13 – C14 – C9 – C8	179.7(1)
C2 – C1 – C4 – C5^i	-57.4(2)	C9 – C8 – C9 – C14	108.4(1)
C3 – C1 – C4 – C5^i	63.4(1)	C7 – C8 – C9 – C14	-124.6(1)
C2 – C1 – C4 – C5	57.4(2)	C9 – C8 – C9 – C10	-72.3(2)
C2 – C1 – C4 – C5	175.8(1)	C7 – C8 – C9 – C10	54.7(1)
C3 – C1 – C4 – C5	-63.4(1)	C14 – C9 – C10 – C11	-0.4(2)
C4 – C5 – C6 – C7	-57.1(1)	C8 – C9 – C10 – C11	-179.7(1)
C5 – C6 – C7 – C6^i	53.6(2)	C13 – C12 – C11 – C10	0.1(2)
C5 – C6 – C7 – C8	-71.2(1)	C9 – C10 – C11 – C12	0.2(2)
C9 – C8 – C7 – C6^i	54.9(2)	C11 – C12 – C13 – C14	-0.1(2)
C9 – C8 – C7 – C6^i	-178.2(1)	C9 – C14 – C13 – C12	-0.1(2)
C9 – C8 – C7 – C6	178.2(1)		

2-((5-methylthiazol-2-yl)methyl)adamantan-2-ol (15a)

Single crystals of compound **15a**, suitable for X-ray diffraction, were obtained by slow evaporation of DCM solution. The crystals were introduced into perfluorinated oil and a suitable single crystal was carefully mounted on the top of a thin glass wire. Data collection was performed with an Oxford Xcalibur 3 diffractometer equipped with a Spellman generator (50 kV, 40 mA) and a Kappa CCD detector, operating with Mo-K_α radiation (λ = 0.71071 Å).

Data collection and data reduction were performed with the CrysAlisPro software.[33] Absorption correction using the multiscan method[34] was applied. The structures were solved with SHELXS-97,[35] refined with SHELXL-97[36] and finally checked using PLATON.[37] Details for data collection and structure refinement are summarized in Table SI 20.

CCDC-2158221 contains supplementary crystallographic data for this compound. These data can be obtained free of charge from The Cambridge Crystallographic Data Centre *via* www.ccdc.cam.ac.uk/data_request/cif.

[33] Program package 'CrysAlisPro 1.171.40.84a (Rigaku OD, 2020'.
[34] Program package 'CrysAlisPro 1.171.40.84a (Rigaku OD, 2020'.
[35] Sheldrick, G. M. (1997) SHELXS-97: *Program for Crystal Structure Solution*, University of Göttingen, Germany.
[36] Sheldrick, G. M. (1997) SHELXL-97: *Program for the Refinement of Crystal Structures*, University of Göttingen, Germany.
[37] Spek, A. L. (1999) PLATON: *A Multipurpose Crystallographic Tool*, Utrecht University, Utrecht, The Netherlands.

Table SI 20. Details for X-ray data collection and structure refinement for compound **15a**.

	**15a**
Empirical formula	$C_{15}H_{21}NOS$
Formula mass	263.39
T[K]	123(2)
Crystal size [mm]	0.40 × 0.30 × 0.15
Crystal description	colorless block
Crystal system	monoclinic
Space group	*P*21/*n*
a [Å]	6.4494(2)
b [Å]	15.6555(7)
c [Å]	13.3328(5)
α [°]	90.0
β [°]	94.321(3)
γ [°]	90.0
V [$Å^3$]	1342.37(9)
Z	4
$\rho_{calcd.}$ [g cm^{-3}]	1.303
μ [mm^{-1}]	0.229
F(000)	568
Θ range [°]	2.01 – 25.24
Index ranges	$-9 \leq h \leq 9$
	$-22 \leq k \leq 22$
	$-19 \leq l \leq 19$
Reflns. collected	27047
Reflns. obsd.	3282
Reflns. unique	4089 (R_{int} = 0.0435)
R_1, wR_2 (2σ data)	0.0418, 0.1000
R_1, wR_2 (all data)	0.0560, 0.1091
GOOF on F^2	1.055
Peak/hole [e $Å^{-3}$]	0.360 / -0.173

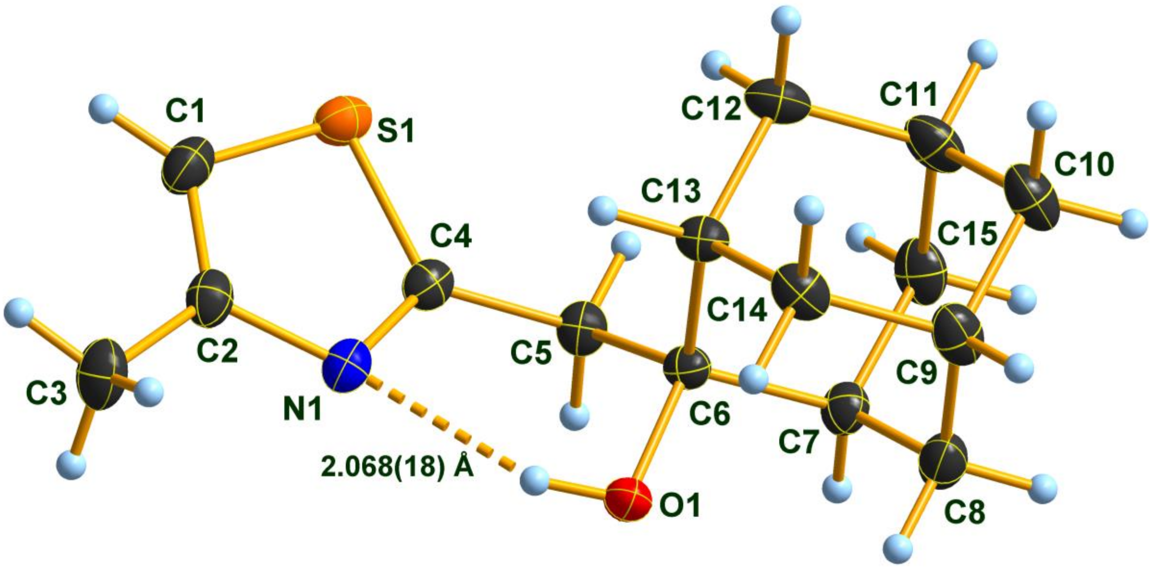

Figure 8. Molecular structure of compound **15a** in the crystal. DIAMOND[38] representation; thermal ellipsoids are drawn at 50 % probability level. Symmetry code: *i*: x, 0.5-y, z.

Table SI 21. Selected bond lengths (Å) of compound **15a**.

S1 – C1	1.715(2)	C11 – C15	1.533(2)
S1 – C4	1.725(1)	C11 – C12	1.537(2)
C6 – O1	1.444(1)	C11 – C10	1.537(2)
C6 – C7	1.541(2)	C2 – C1	1.363(2)
C6 – C13	1.543(2)	C2 – C3	1.493(2)
C6 – C5	1.545(2)	C7 – C8	1.535(2)
C4 – N1	1.310(2)	C7 – C15	1.541(2)
C4 – C5	1.497(2)	C9 – C8	1.534(2)
C13 – C14	1.537(2)	C9 – C14	1.534(2)
C13 – C12	1.540(2)	C9 – C10	1.536(2)
N1 – C2	1.384(2)		

Table SI 22. Selected bond angles (°) of compound **15a**.

C1 – S1 – C4	89.7(1)	C2 – C1 – S1	110.4(1)
O1 – C6 – C7	106.7(1)	C9 – C14 – C13	109.9(1)
O1 – C6 – C13	110.1(1)	C15 – C11 – C12	109.7(1)
C7 – C6 – C13	108.4(1)	C15 – C11 – C10	109.2(1)
O1 – C6 – C5	107.5(1)	C12 – C11 – C10	109.3(1)
C7 – C6 – C5	110.7(1)	C11 – C12 – C13	109.8(1)

[38] DIAMOND, Crystal Impact GbR., Version 3.2i.

C13 – C6 – C5	113.3(1)	C11 – C15 – C7	109.9(1)
N1 – C4 – C5	123.4(1)	C9 – C10 – C11	109.2(1)
N1 – C4 – S1	114.1(1)	C8 – C7 – C15	108.4(1)
C5 – C4 – S1	122.5(1)	C8 – C7 – C6	109.7(1)
C14 – C13 – C12	108.6(1)	C15 – C7 – C6	110.6(1)
C14 – C13 – C6	109.4(1)	C8 – C9 – C14	109.2(1)
C12 – C13 – C6	110.5(1)	C8 – C9 – C10	109.6(1)
C4 – N1 – C2	111.4(1)	C14 – C9 – C10	109.6(1)
C1 – C2 – N1	114.5(1)	C9 – C8 – C7	109.8(1)
C1 – C2 – C3	126.5(1)	C4 – C5 – C6	112.6(1)
N1 – C2 – C3	119.1(1)		

Table SI 23. Selected torsion angles (°) of compound **15a**.

C1 – S1 – C4 – N1	-0.3(1)	S1 – C4 – C5 – C6	127.8(1)
C1 – S1 – C4 – C5	-179.9(1)	O1 – C6 – C5 – C4	65.0(1)
O1 – C6 – C13 – C14	56.0(1)	C7 – C6 – C5 – C4	-178.8(1)
C7 – C6 – C13 – C14	-60.4(1)	C13 – C6 – C5 – C4	-56.9(1)
C5 – C6 – C13 – C14	176.4(1)	N1 – C2 – C1 – S1	-0.7(2)
O1 – C6 – C13 – C12	175.5(1)	C3 – C2 – C1 – S1	179.3(1)
C7 – C6 – C13 – C12	59.1(1)	C4 – S1 – C1 – C2	0.6(1)
C5 – C6 – C13 – C12	-64.1(1)	C8 – C9 – C14 – C13	-59.7(1)
C5 – C4 – N1 – C2	179.6(1)	C10 – C9 – C14 – C13	60.4(1)
S1 – C4 – N1 – C2	0.0(1)	C12 – C13 – C14 – C9	-60.0(1)
C4 – N1 – C2 – C1	0.5(2)	C6 – C13 – C14 – C9	60.6(1)
C4 – N1 – C2 – C3	-179.6(1)	C15 – C11 – C12 – C13	59.0(1)
O1 – C6 – C7 – C8	-58.1(1)	C10 – C11 – C12 – C13	-60.7(1)
C13 – C6 – C7 – C8	60.5(1)	C14 – C13 – C12 – C11	60.2(1)
C5 – C6 – C7 – C8	-174.7(1)	C6 – C13 – C12 – C11	-59.7(1)
O1 – C6 – C7 – C15	-177.5(1)	C12 – C11 – C15 – C7	-58.9(1)
C13 – C6 – C7 – C15	-59.0(1)	C10 – C11 – C15 – C7	60.8(1)
C5 – C6 – C7 – C15	65.8(1)	C8 – C7 – C15 – C11	-60.7(1)
C14 – C9 – C8 – C7	59.5(1)	C6 – C7 – C15 – C11	59.6(1)
C10 – C9 – C8 – C7	-60.5(1)	C8 – C9 – C10 – C11	59.8(2)
C15 – C7 – C8 – C9	60.3(1)	C14 – C9 – C10 – C11	-59.9(2)
C6 – C7 – C8 – C9	-60.5(1)	C15 – C11 – C10 – C9	-59.9(1)
N1 – C4 – C5 – C6	-51.7(2)	C12 – C11 – C10 – C9	60.1(2)

Chiral HPLC Analysis

(*R*/*S*)-4-(3,5-dimethylphenyl)butan-2-ol (10h)

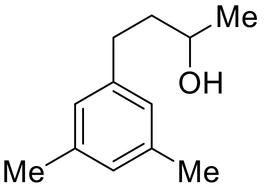

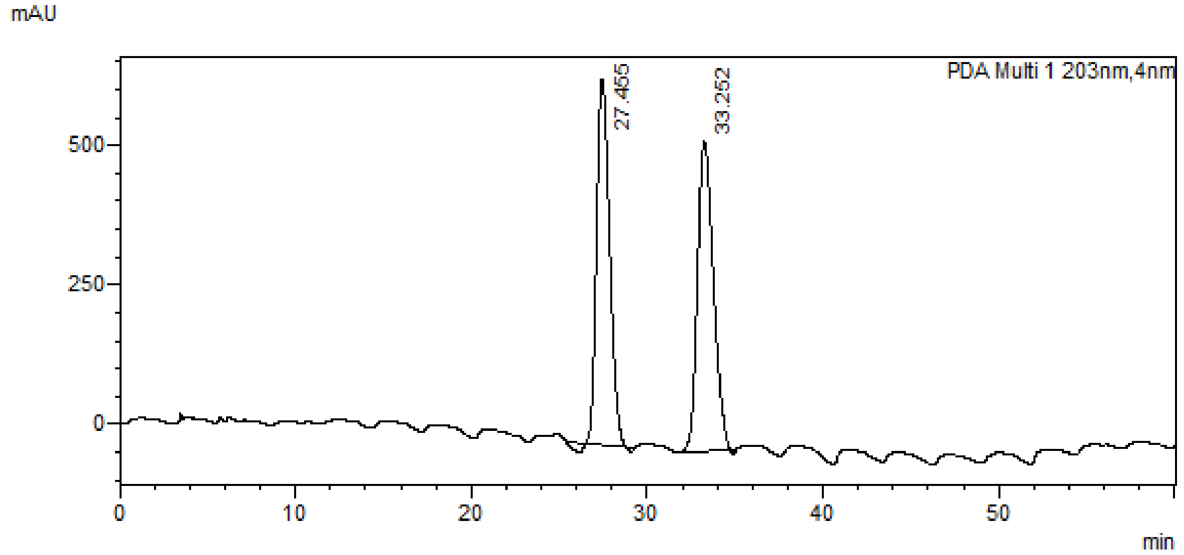

<Peak Table>

PDA Ch1 203nm

Peak#	Ret. Time	Area	Height	Area%
1	27.455	32783298	655404	48.321
2	33.252	35060950	554727	51.679
Total		67844248	1210131	100.000

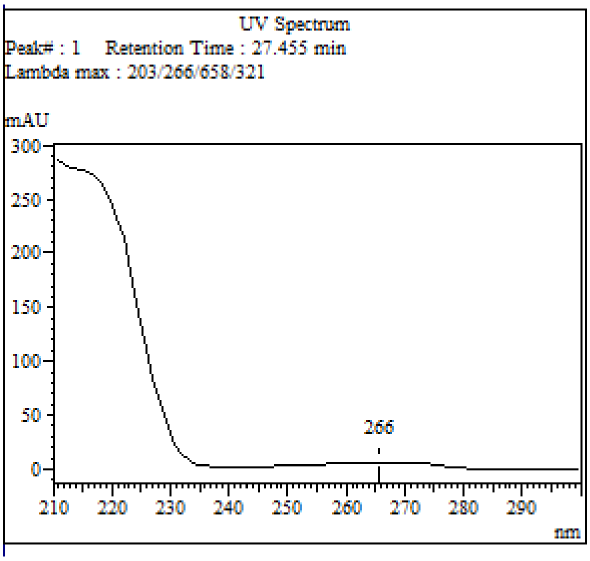

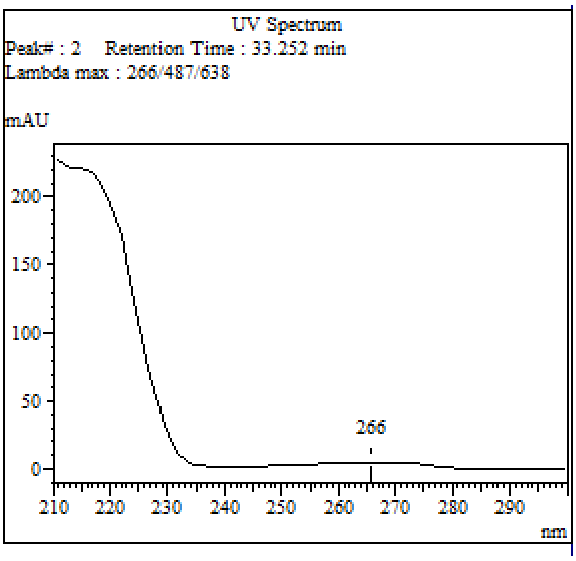

(*S*)-4-(3,5-dimethylphenyl)butan-2-ol (10h)

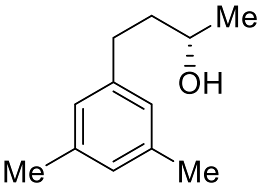

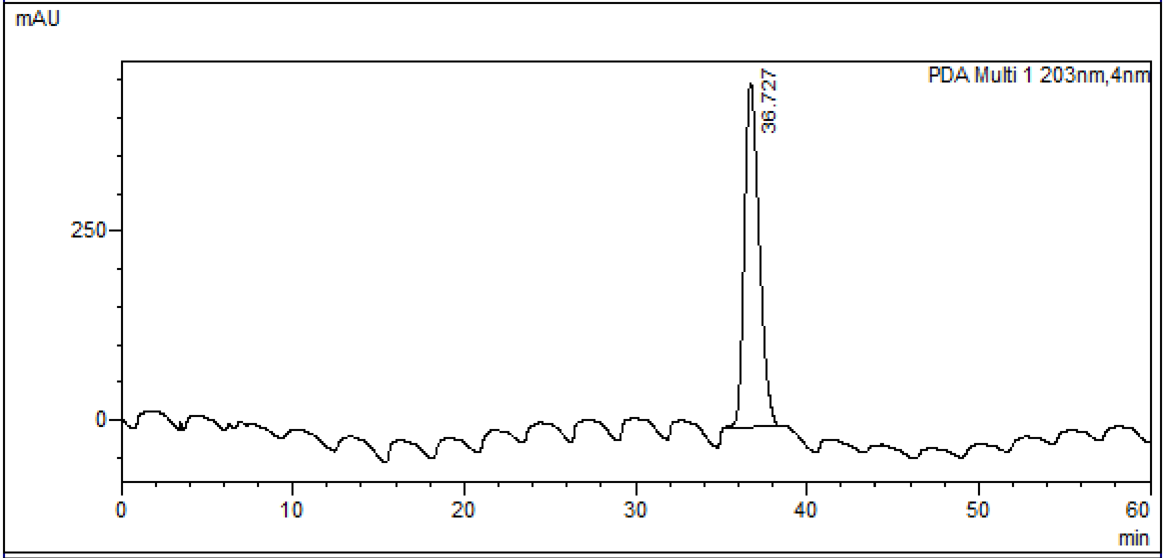

<Peak Table>

PDA Ch1 203nm

Peak#	Ret. Time	Area	Height	Area%
1	36.727	27397926	454345	100.000
Total		27397926	454345	100.000

UV Spectrum

Peak# : 1 Retention Time : 36.727 min

Lambda max : 266/658/322/645

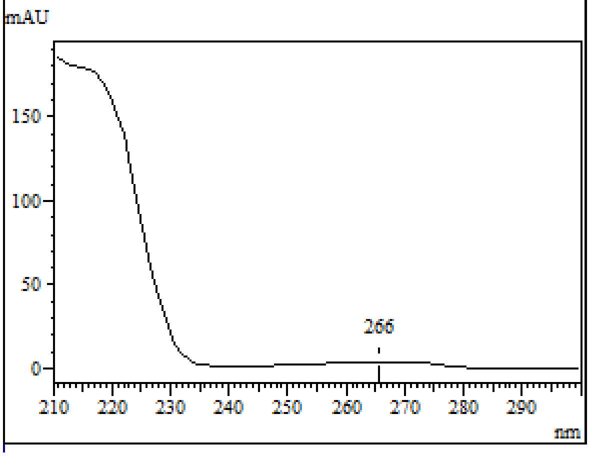

UV Spectrum

NMR-Spectra

***trans*-2-(3,5-dimethylbenzyl)cyclohexan-1-ol (10a)**

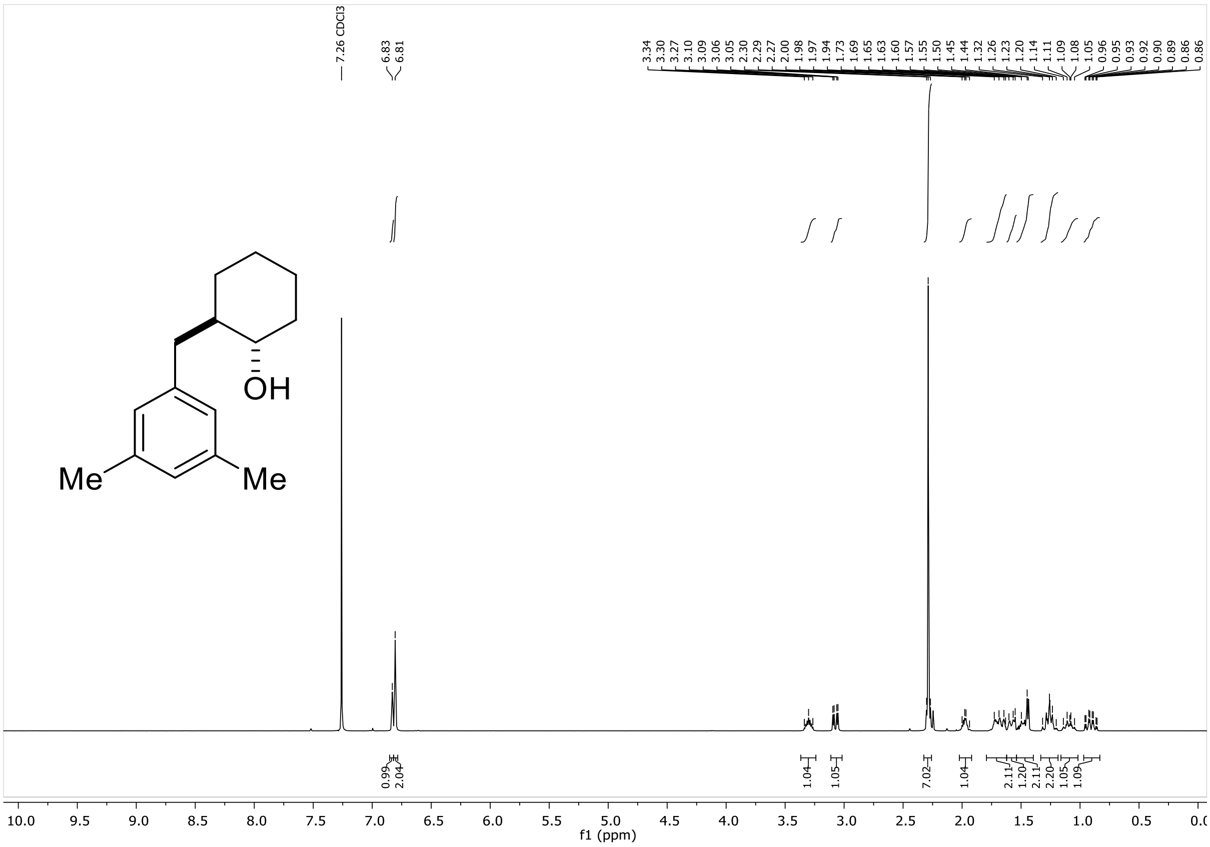

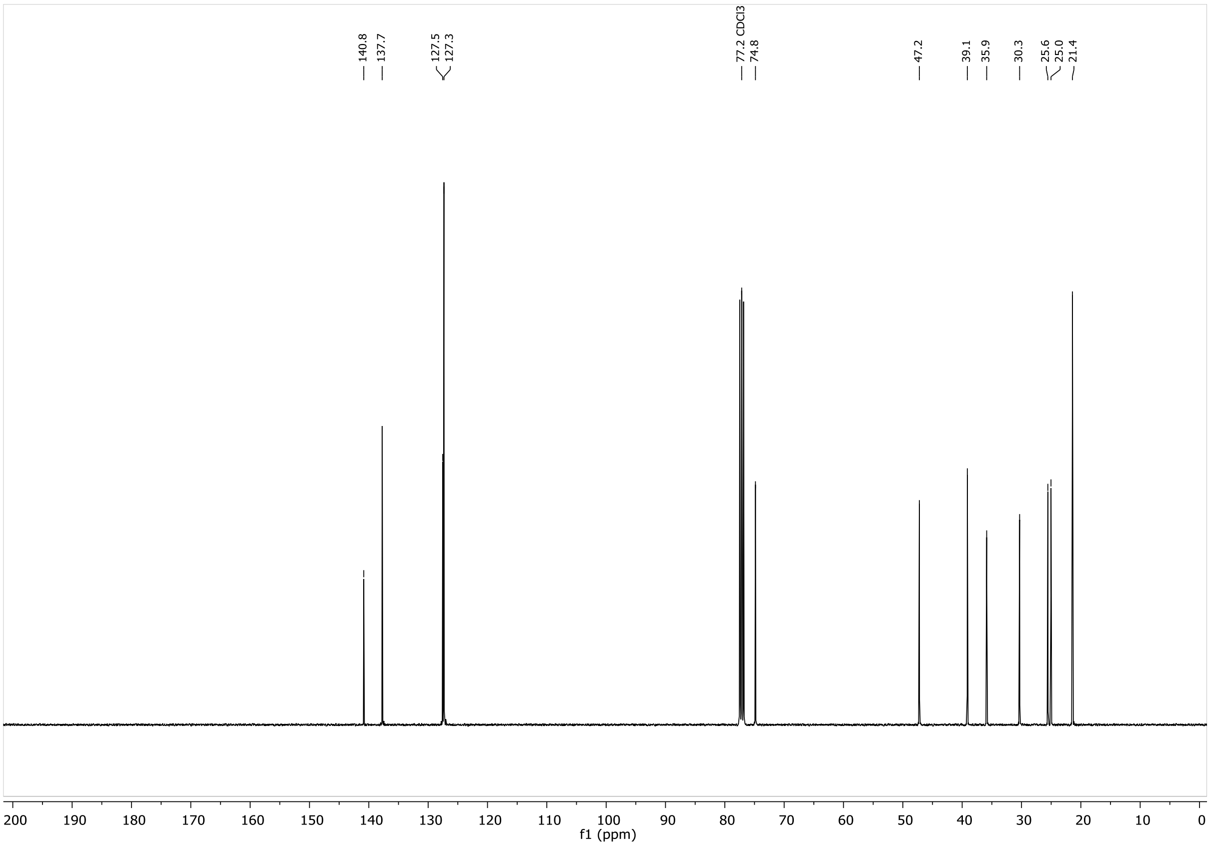

trans-2-benzylcyclohexan-1-ol (10b)

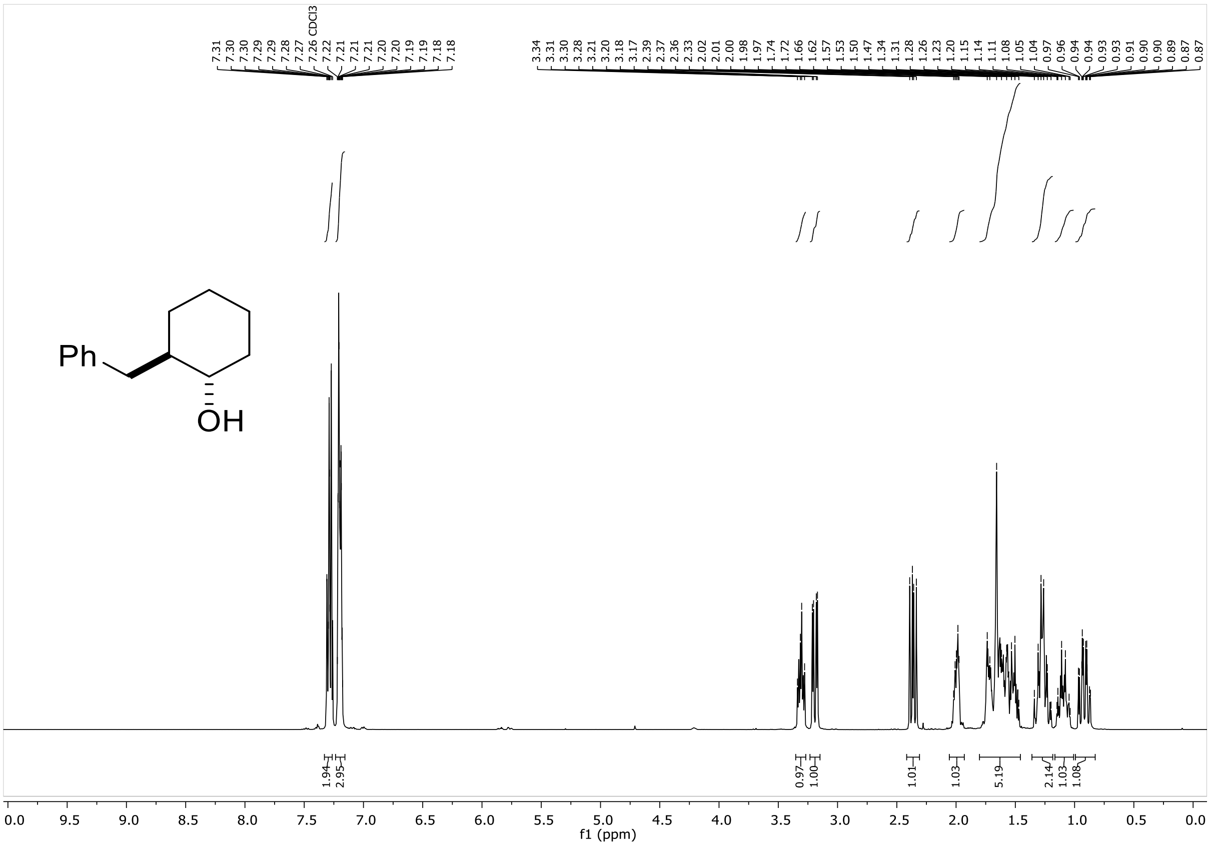

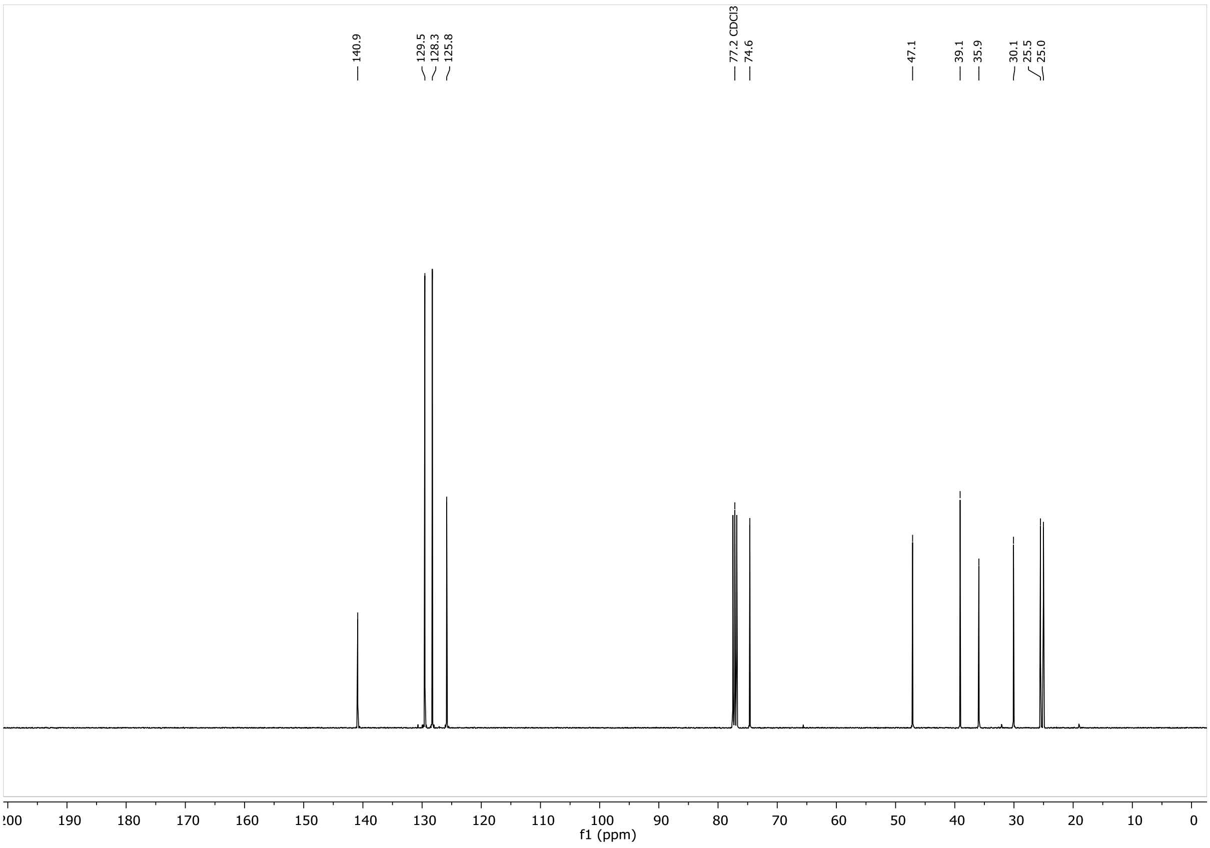

trans-2-(4-methylbenzyl)cyclohexan-1-ol (10c)

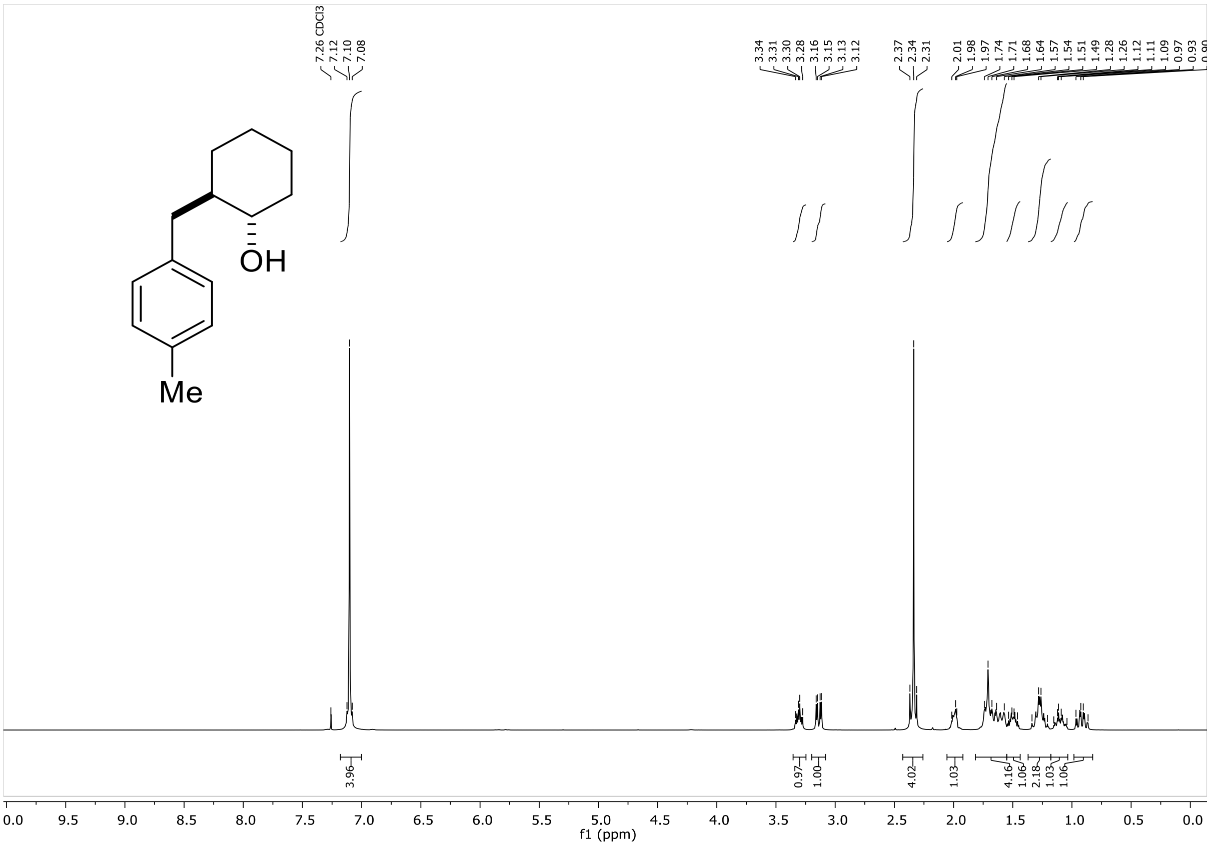

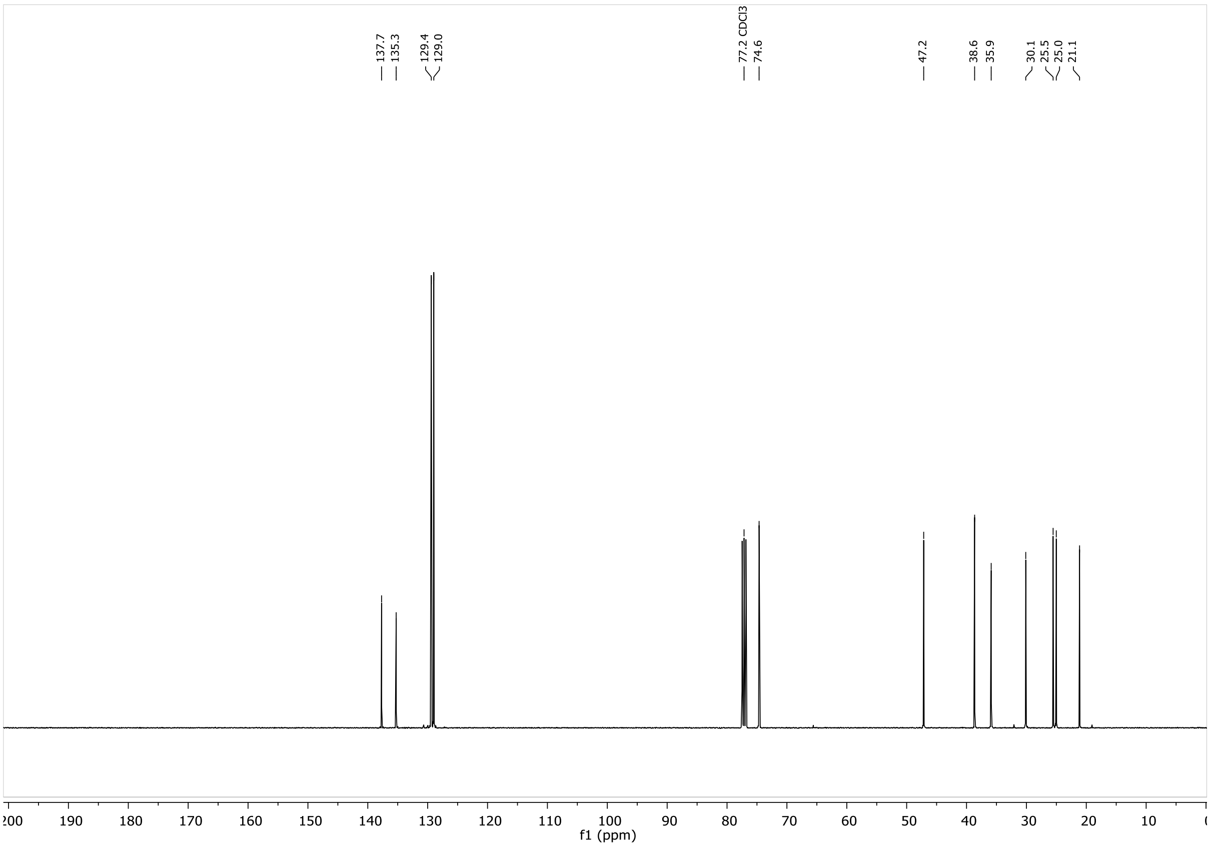

2-methyl-4-phenylbutan-2-ol (10d)

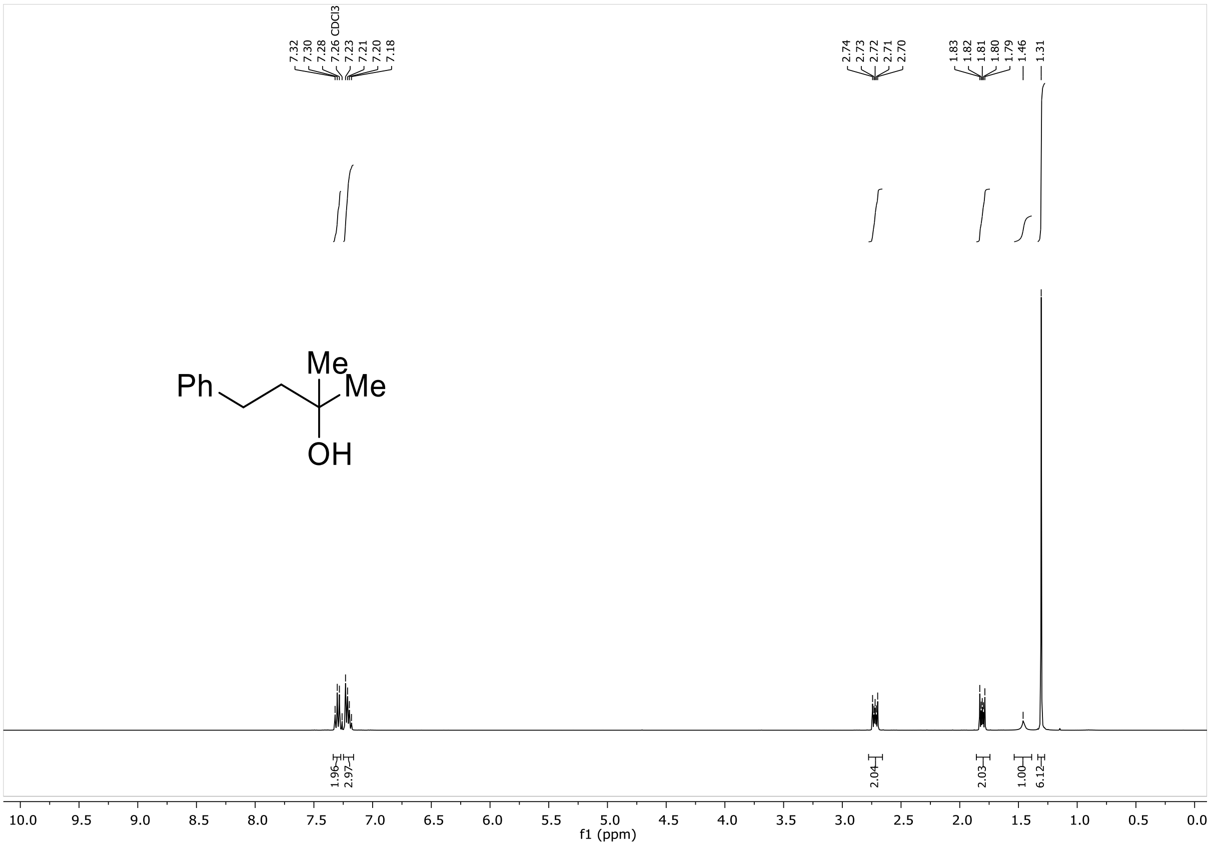

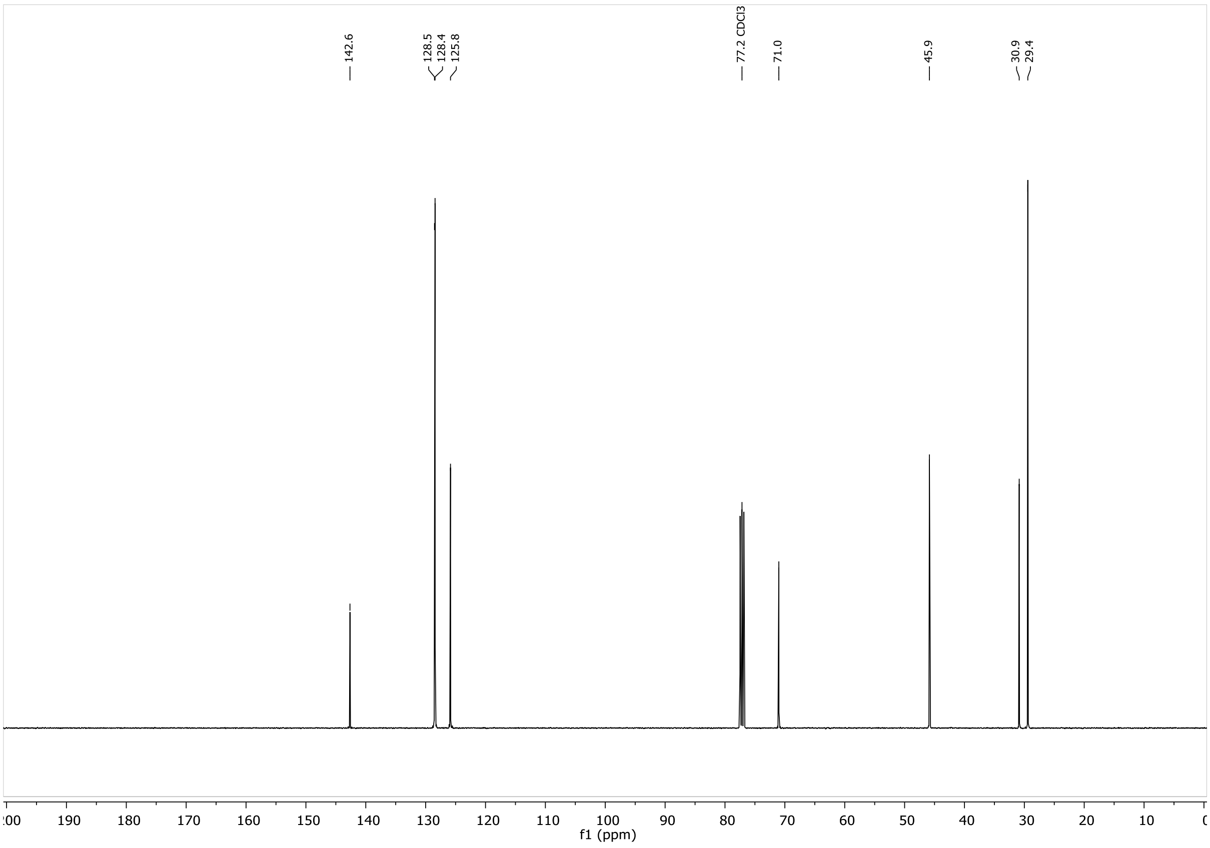

2-methyl-4-(p-tolyl)butan-2-ol (10e)

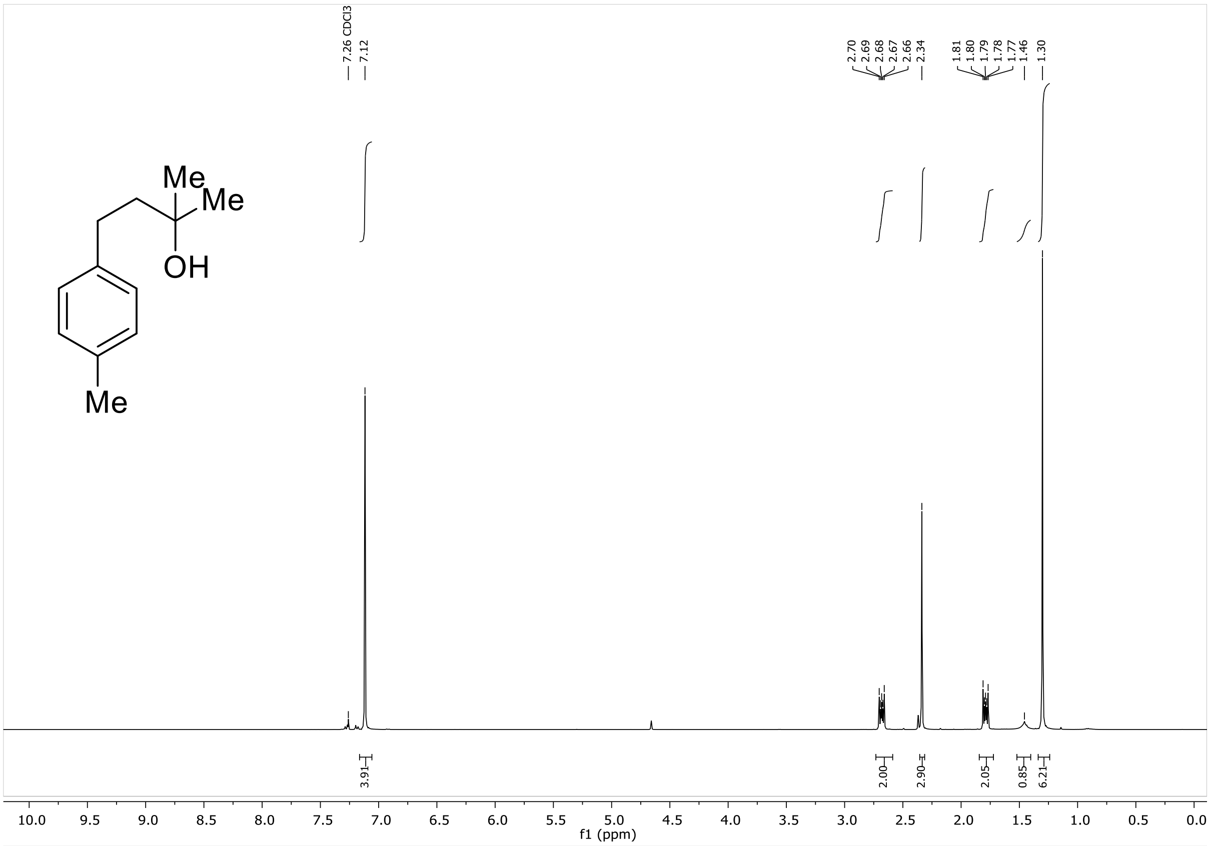

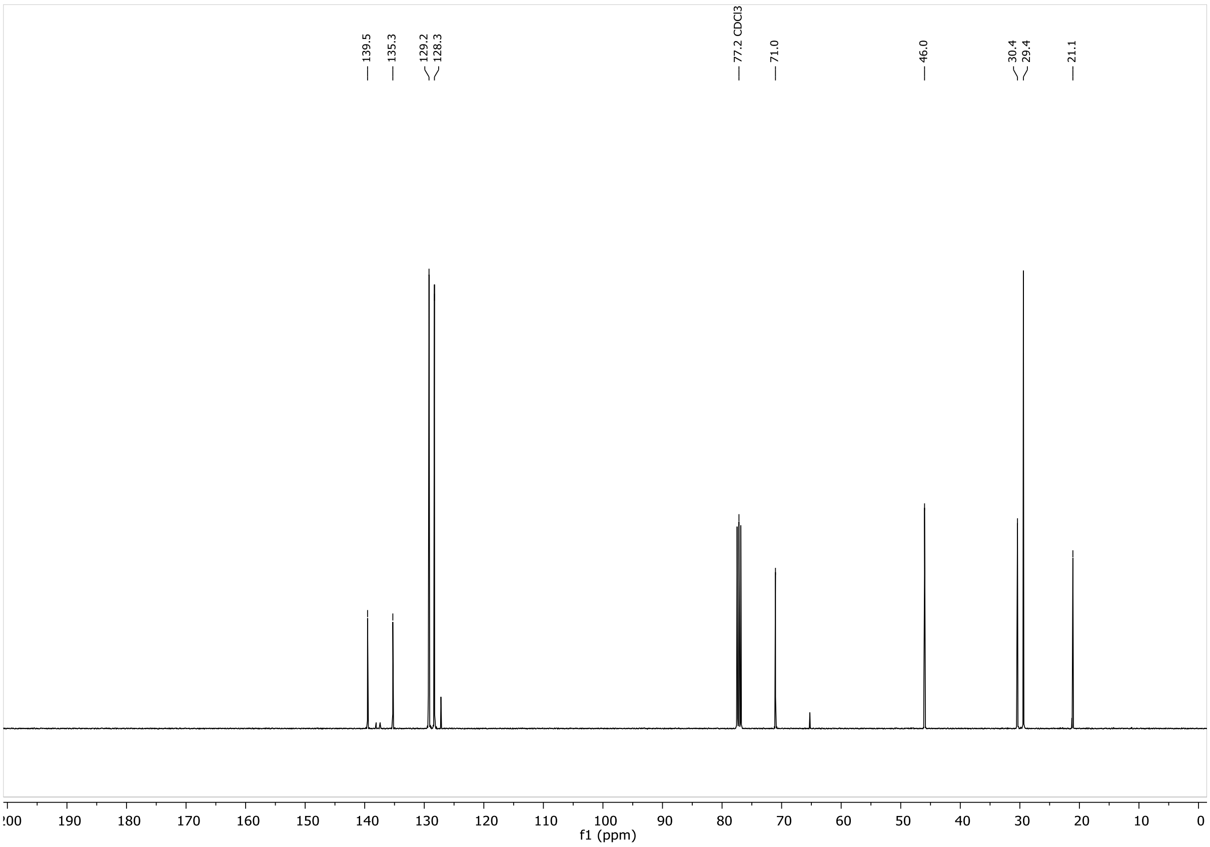

4-(3,5-dimethylphenyl)-2-methylbutan-2-ol (10f)

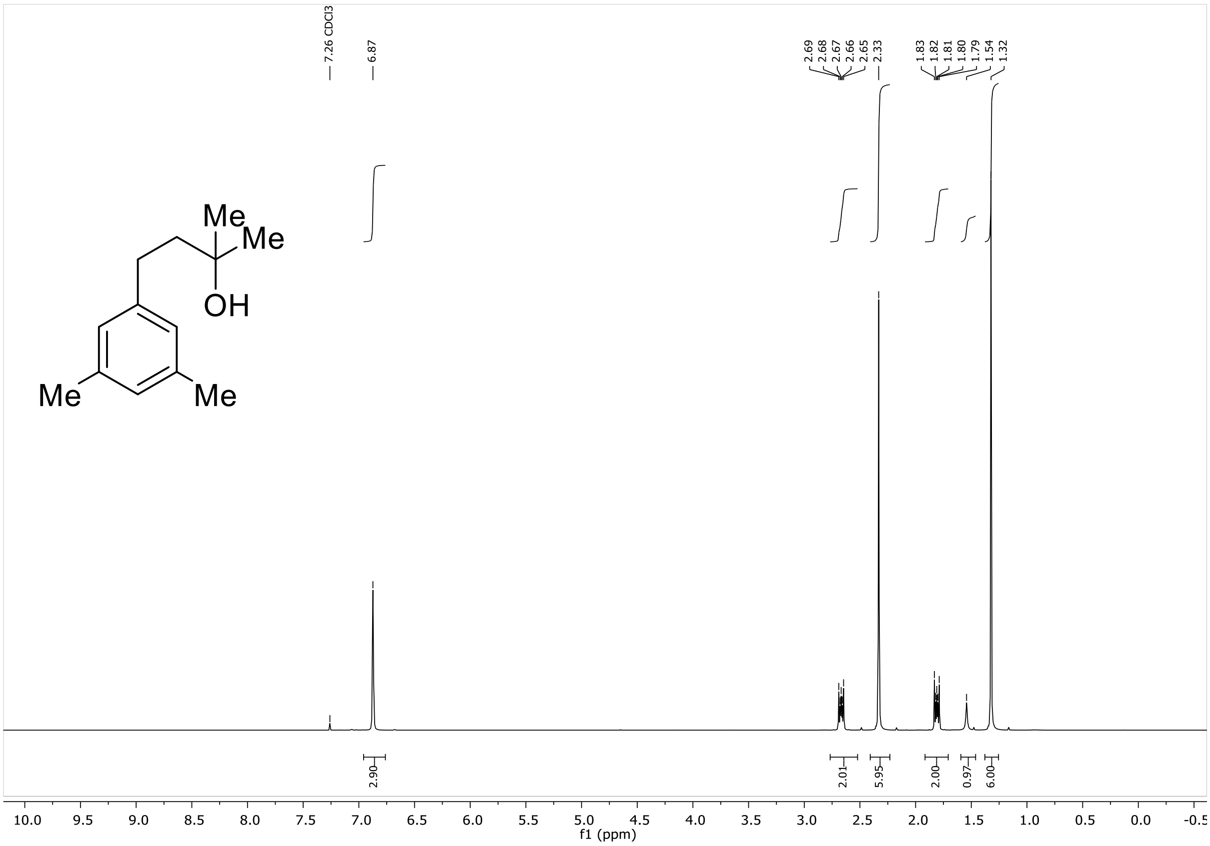

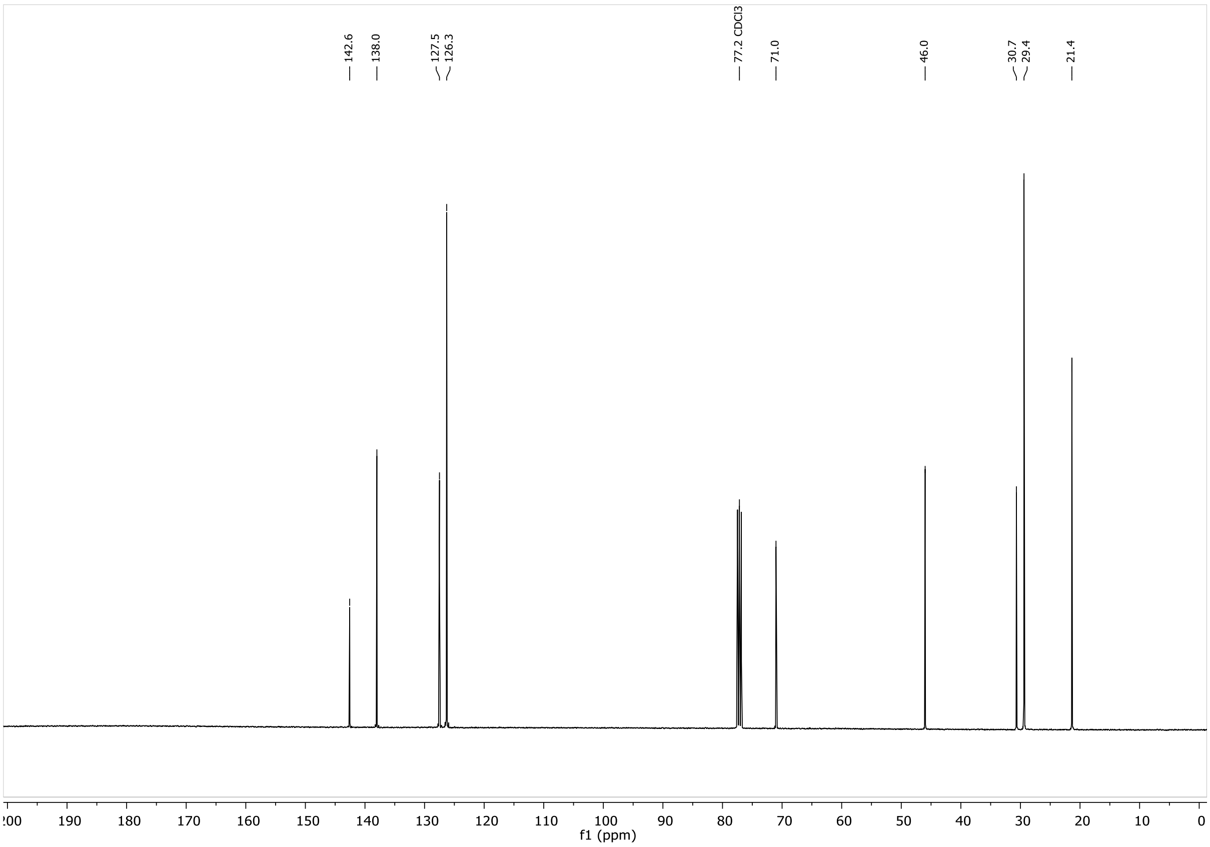

1-(3,5-dimethylphenyl)pentan-3-ol (10g)

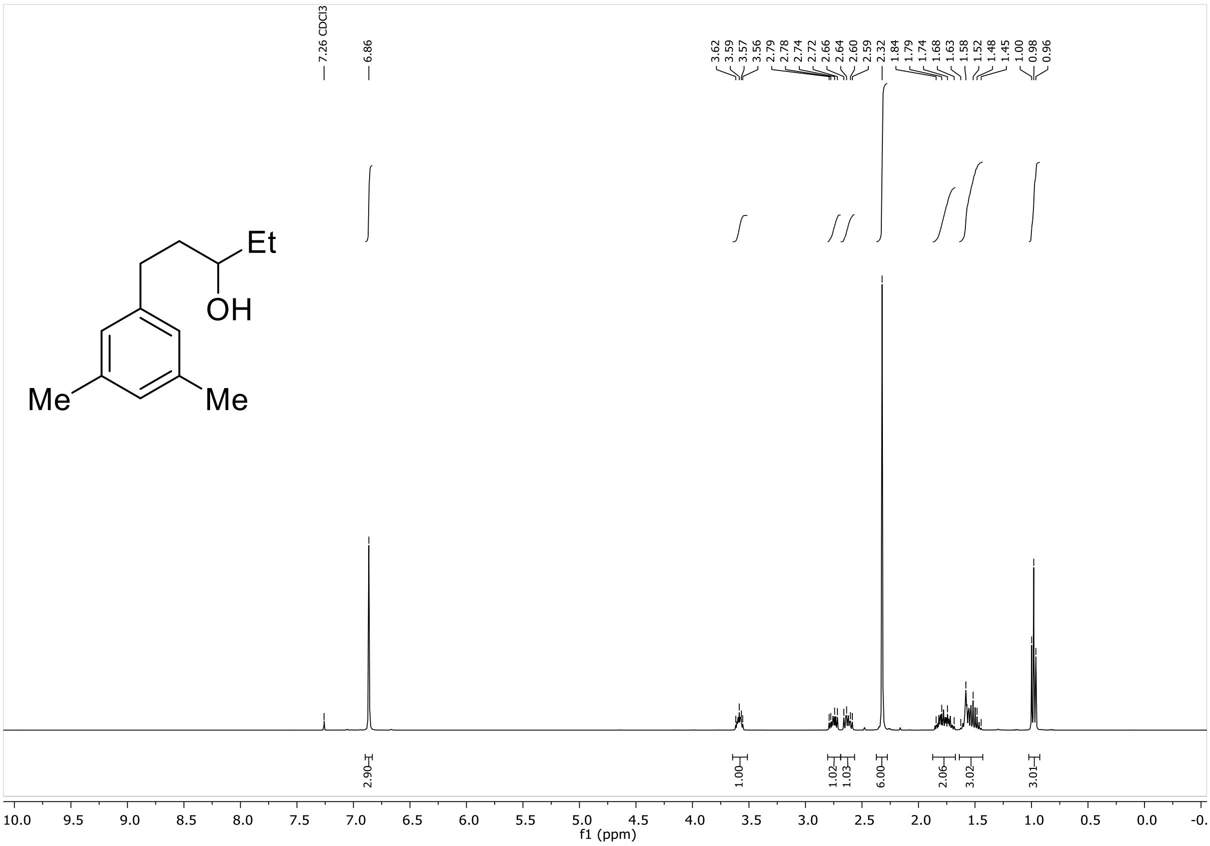

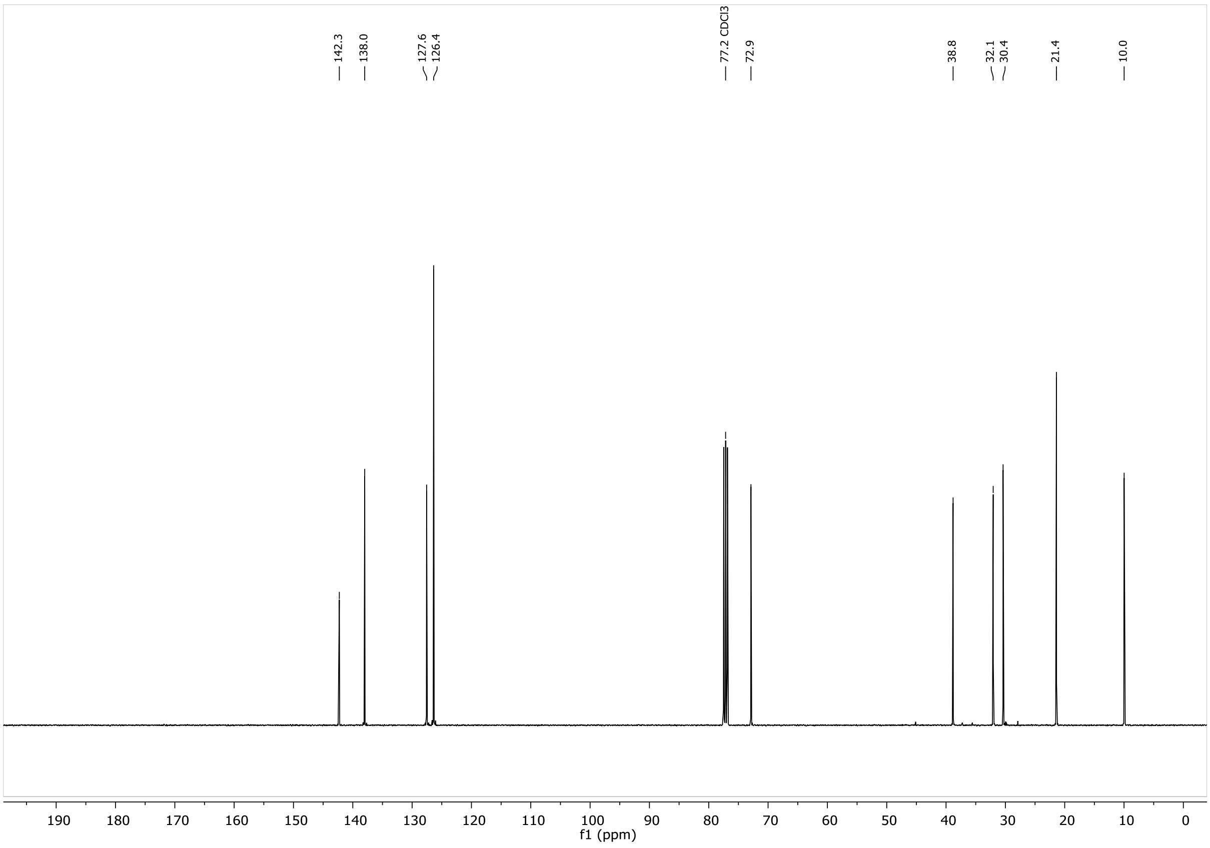

(*S*)-4-(3,5-dimethylphenyl)butan-2-ol (10h)

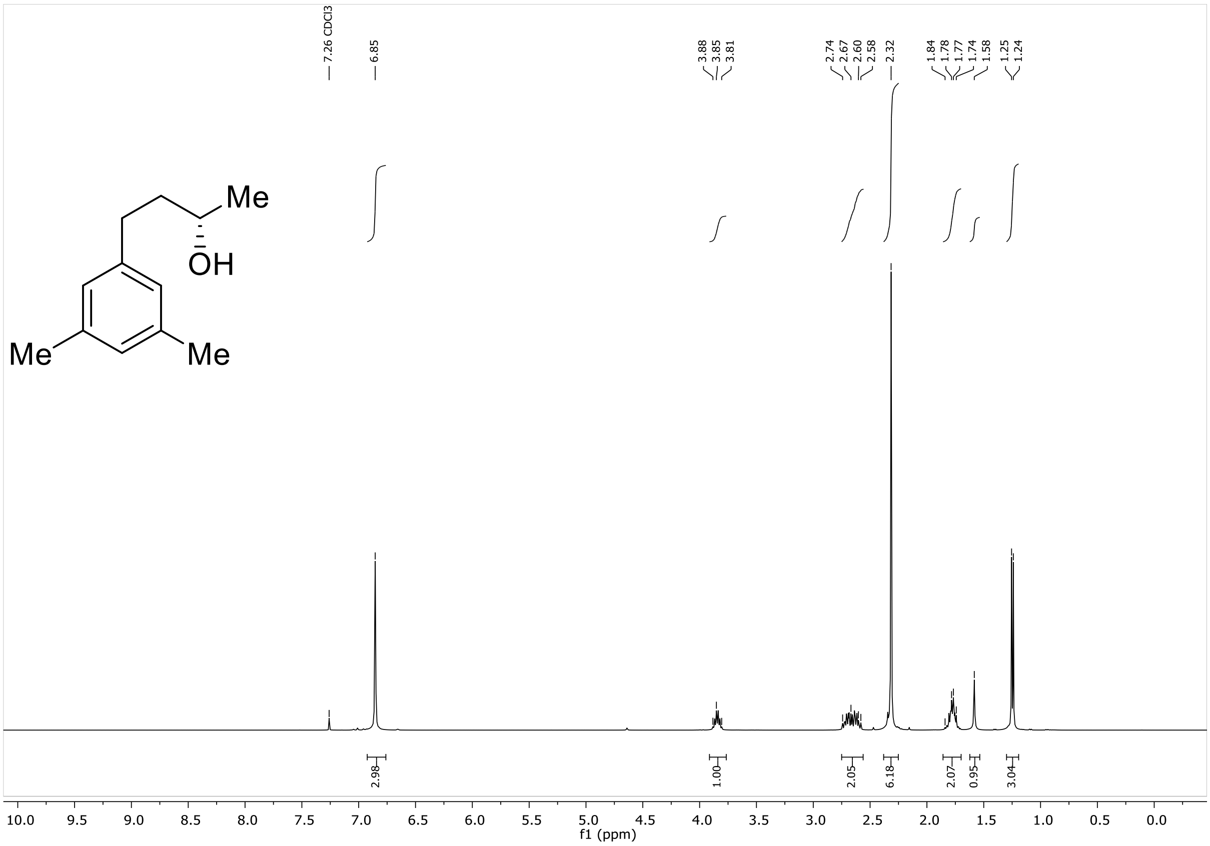

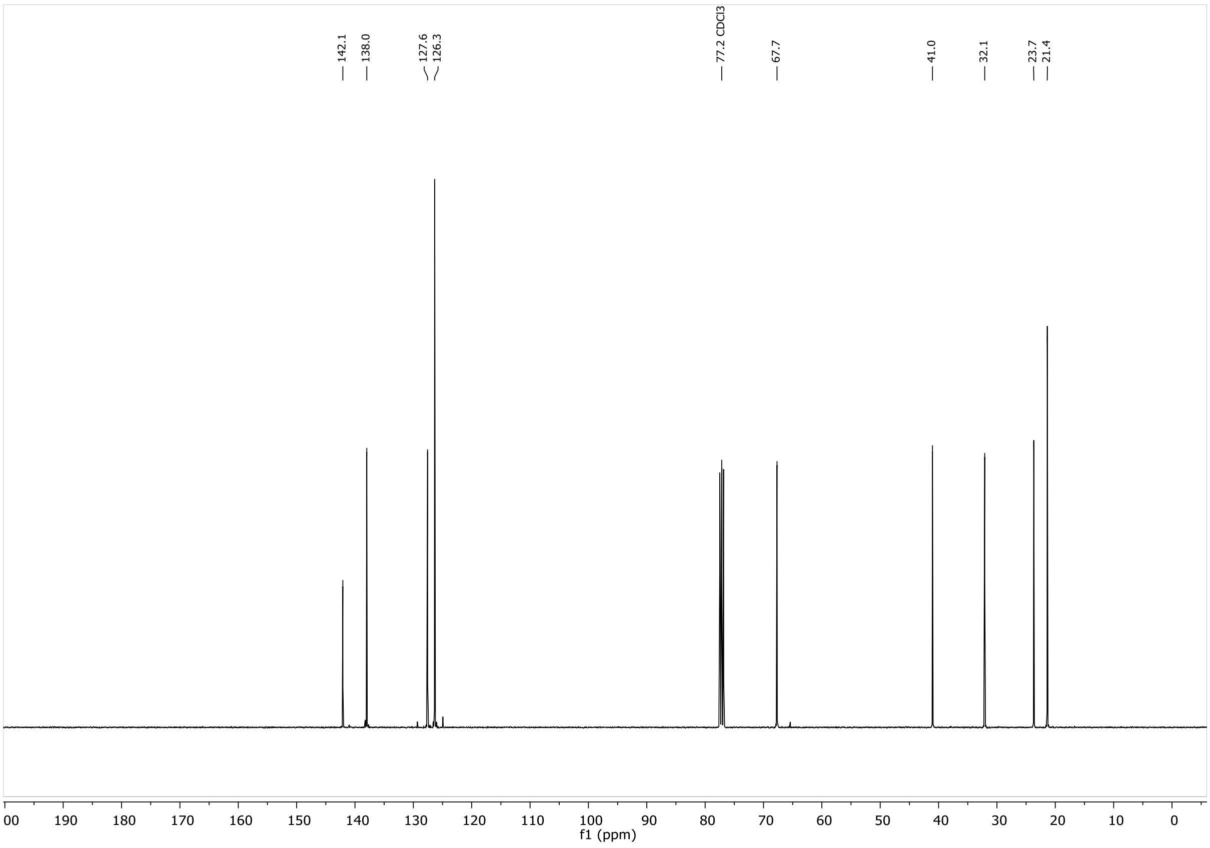

1-phenylbutan-1-ol (10i)

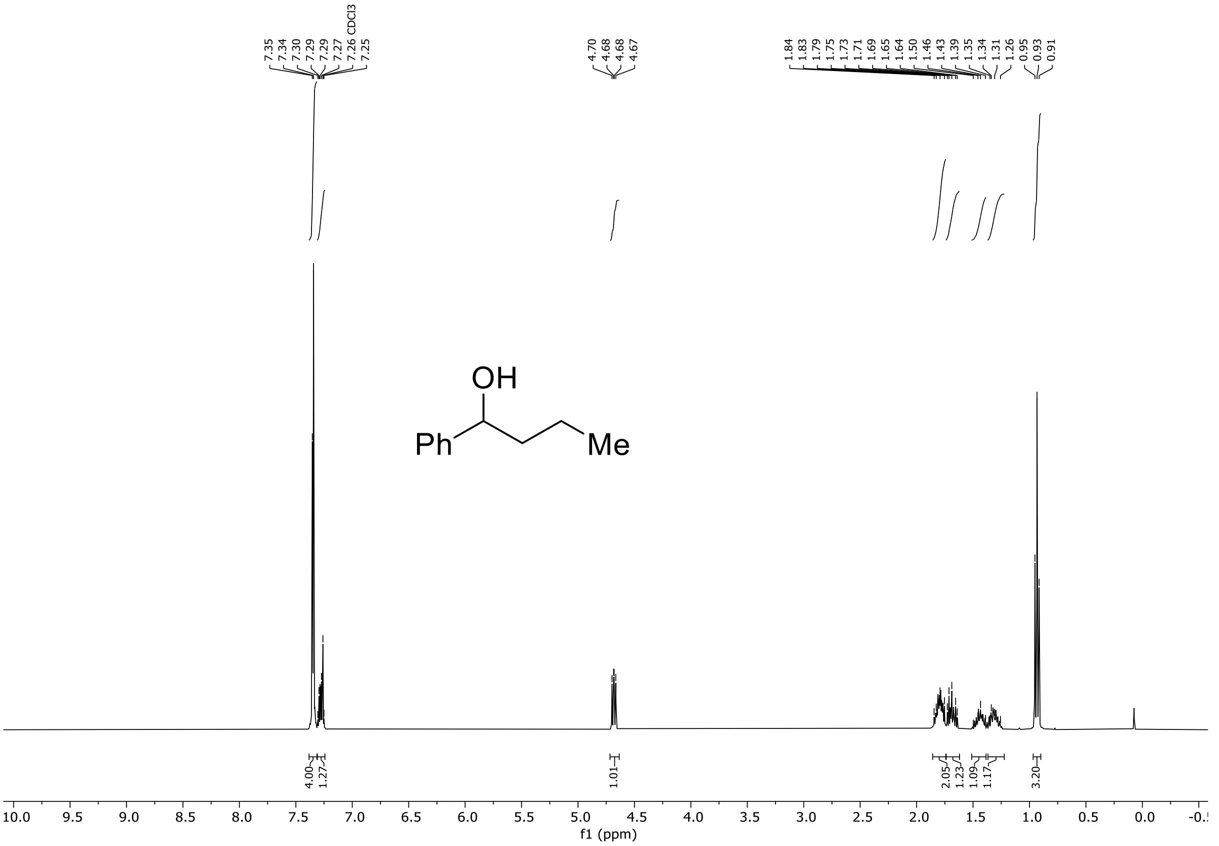

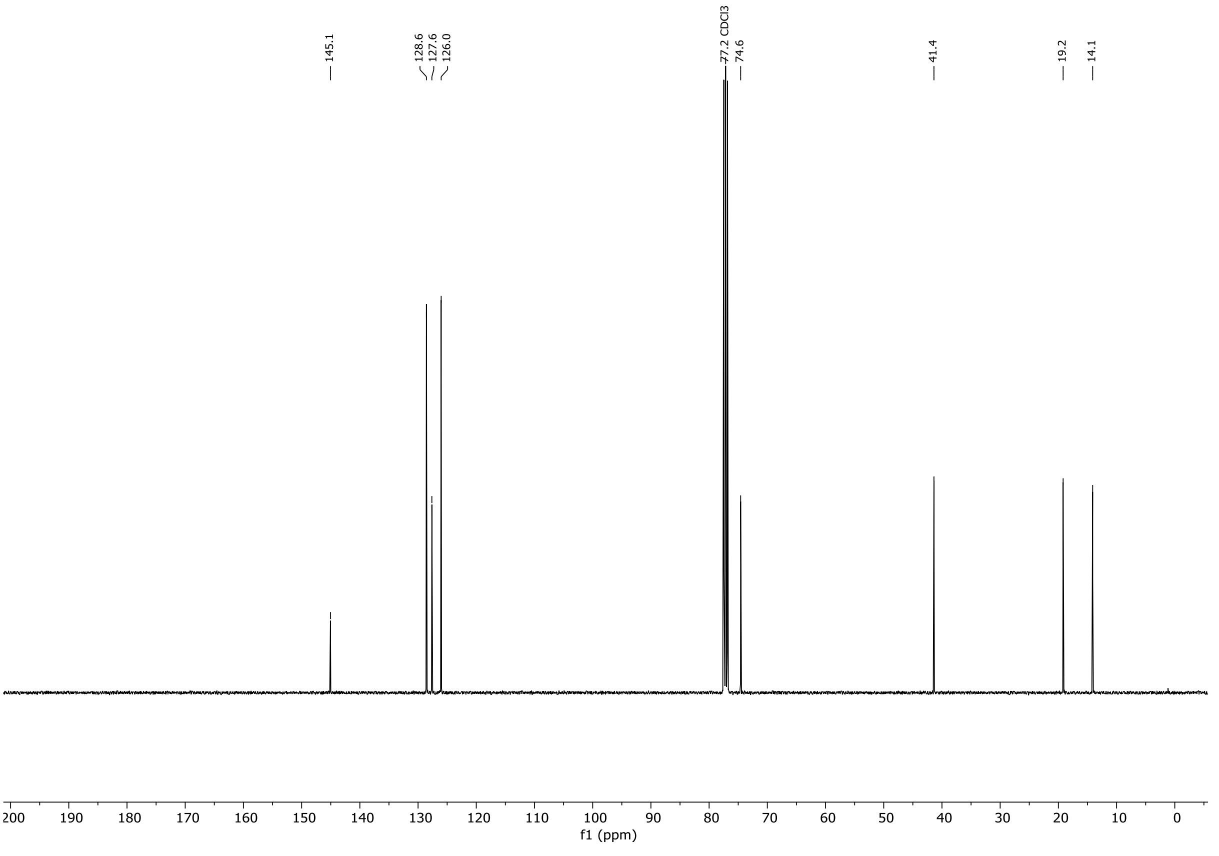

1-phenylbutan-2-ol (10j)

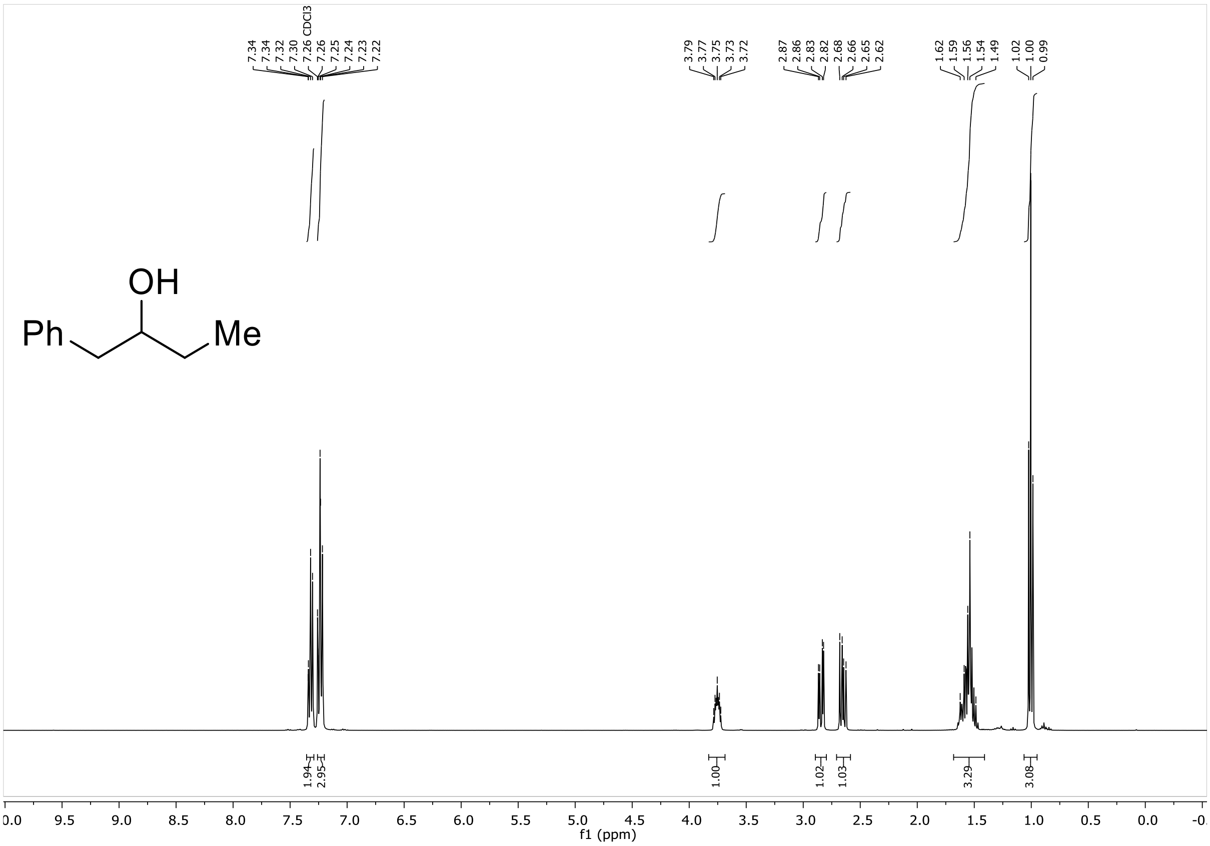

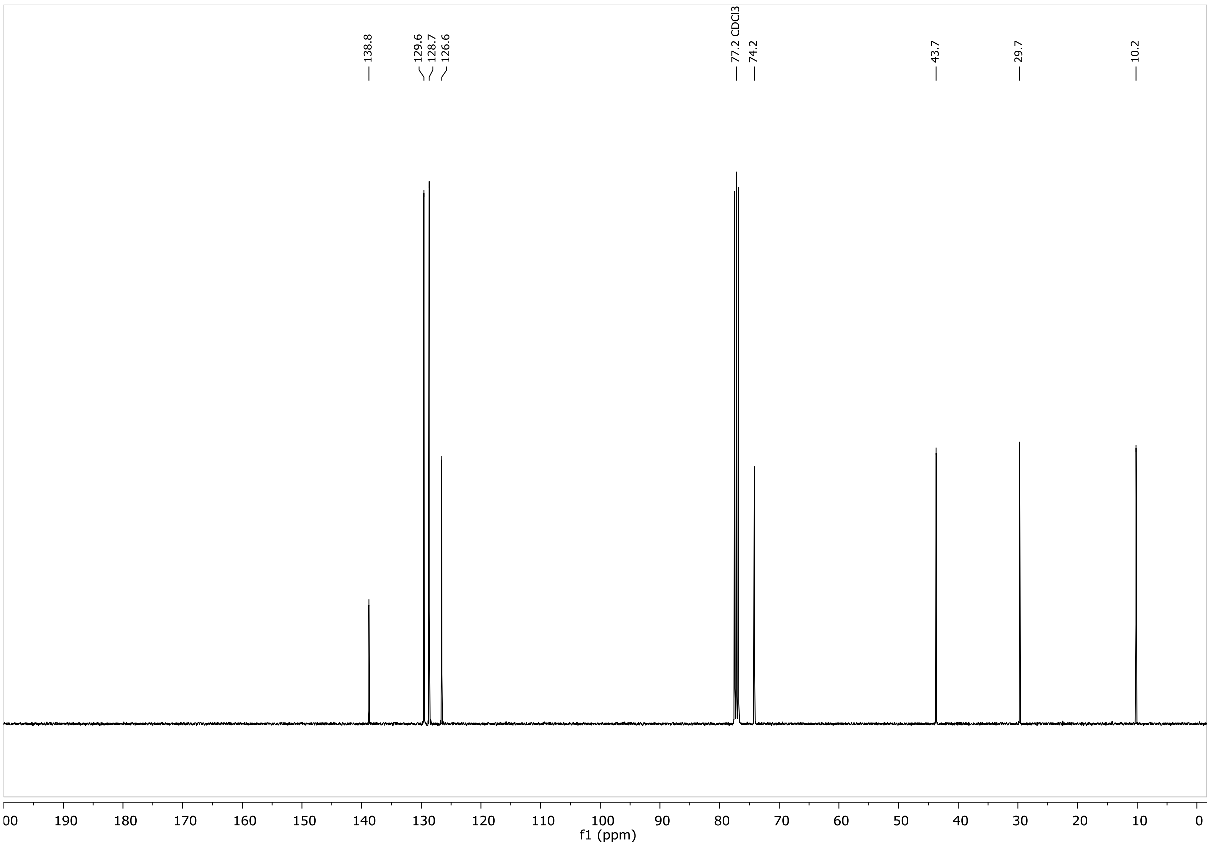

4-phenylbutan-2-ol (10k)

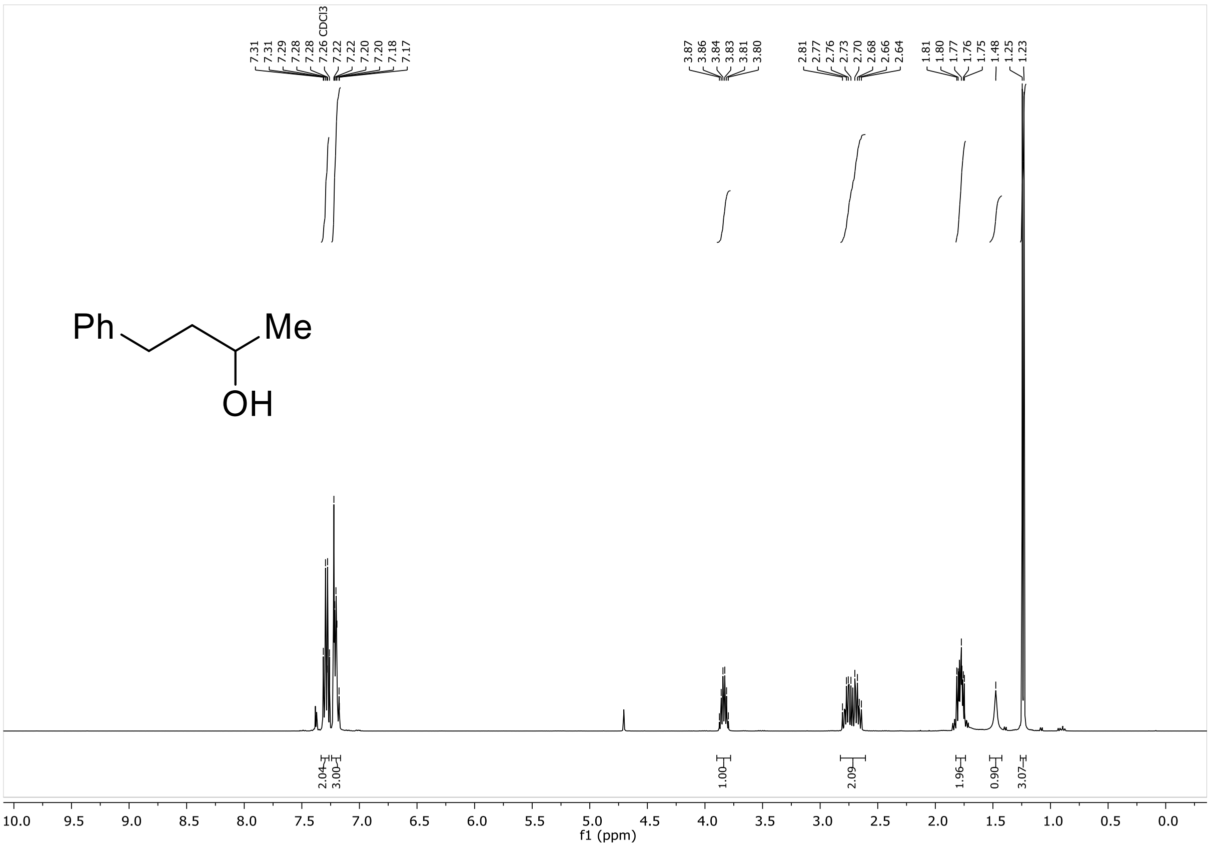

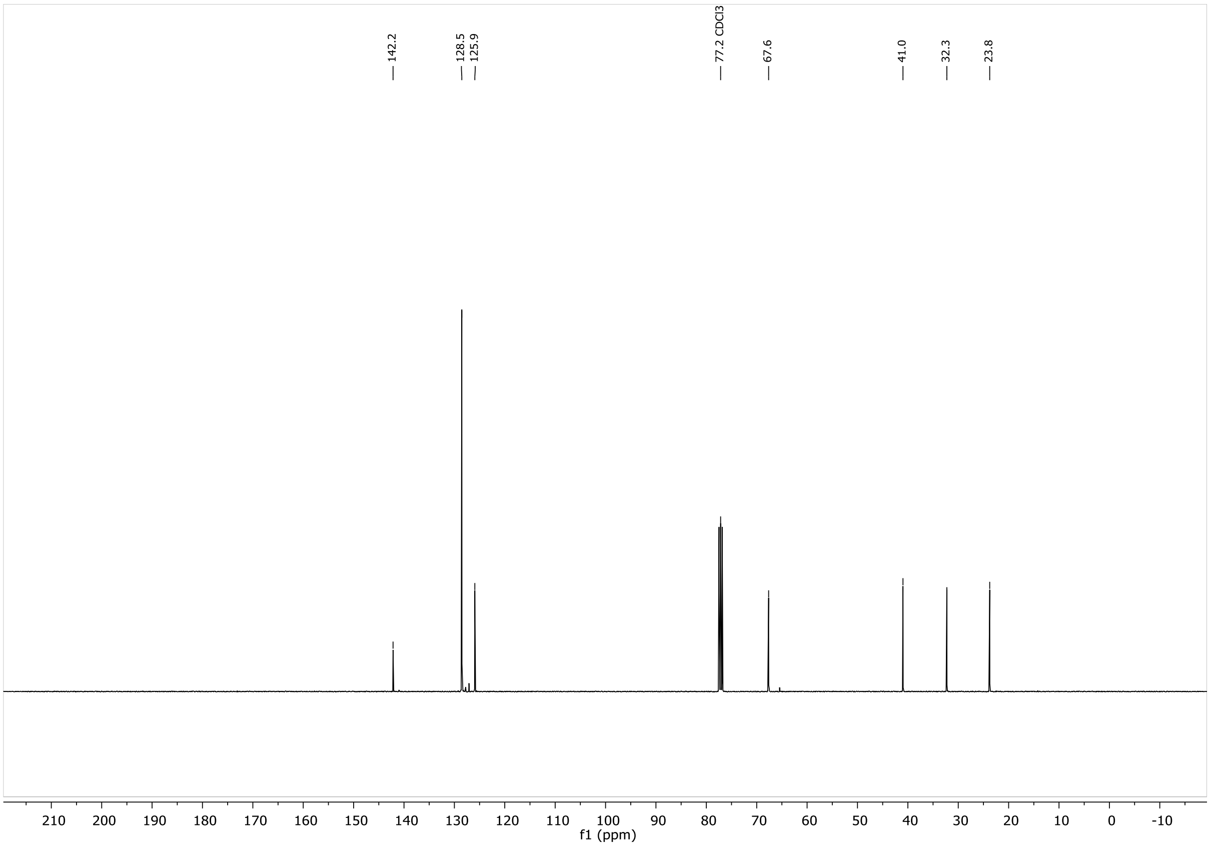

4-phenylbutan-1-ol (10l)

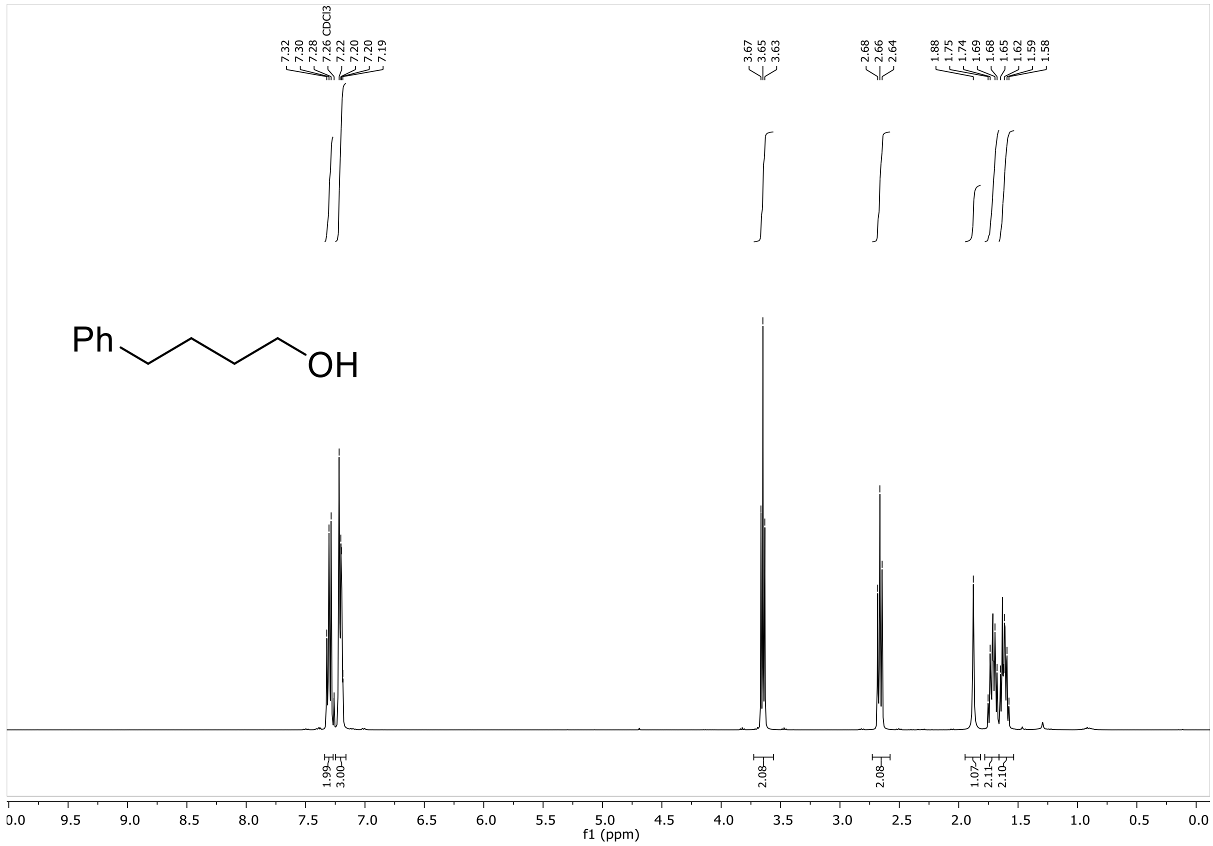

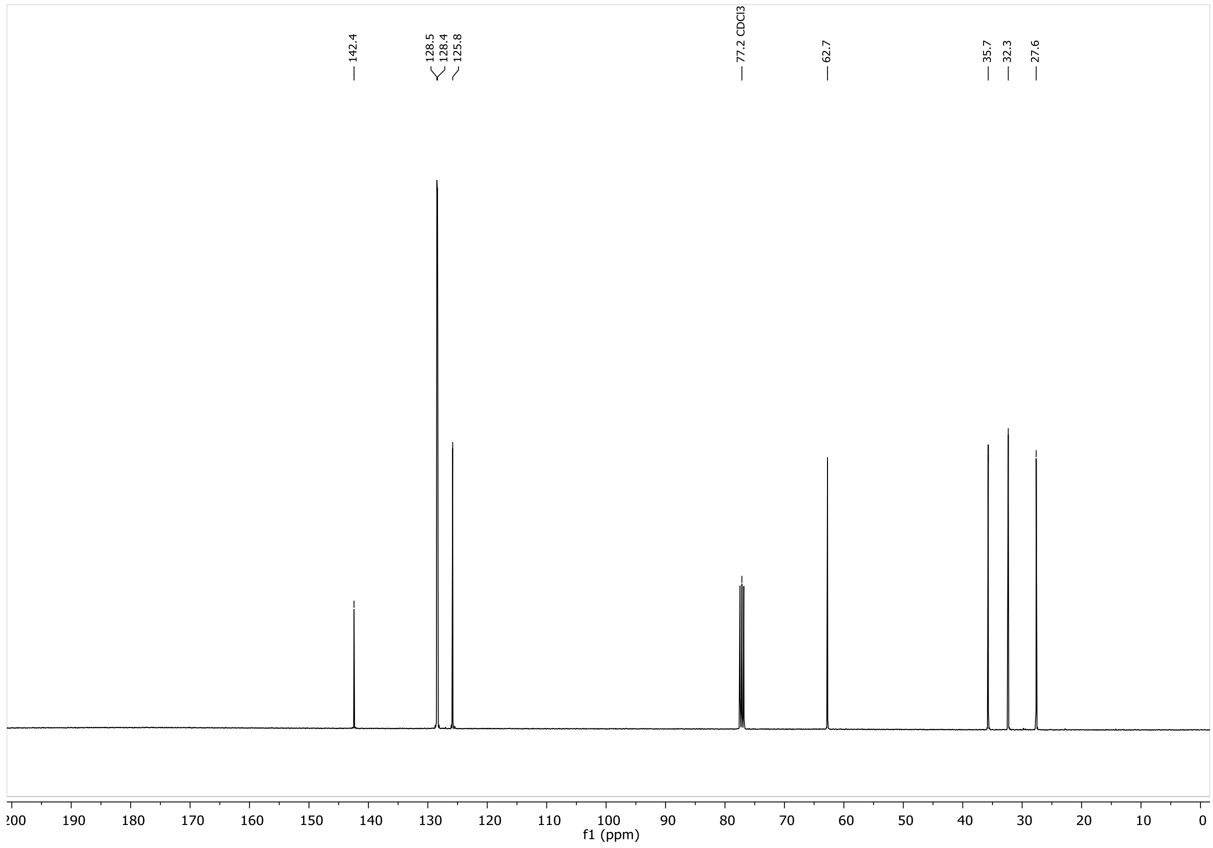

4-(p-tolyl)butan-1-ol (10m)

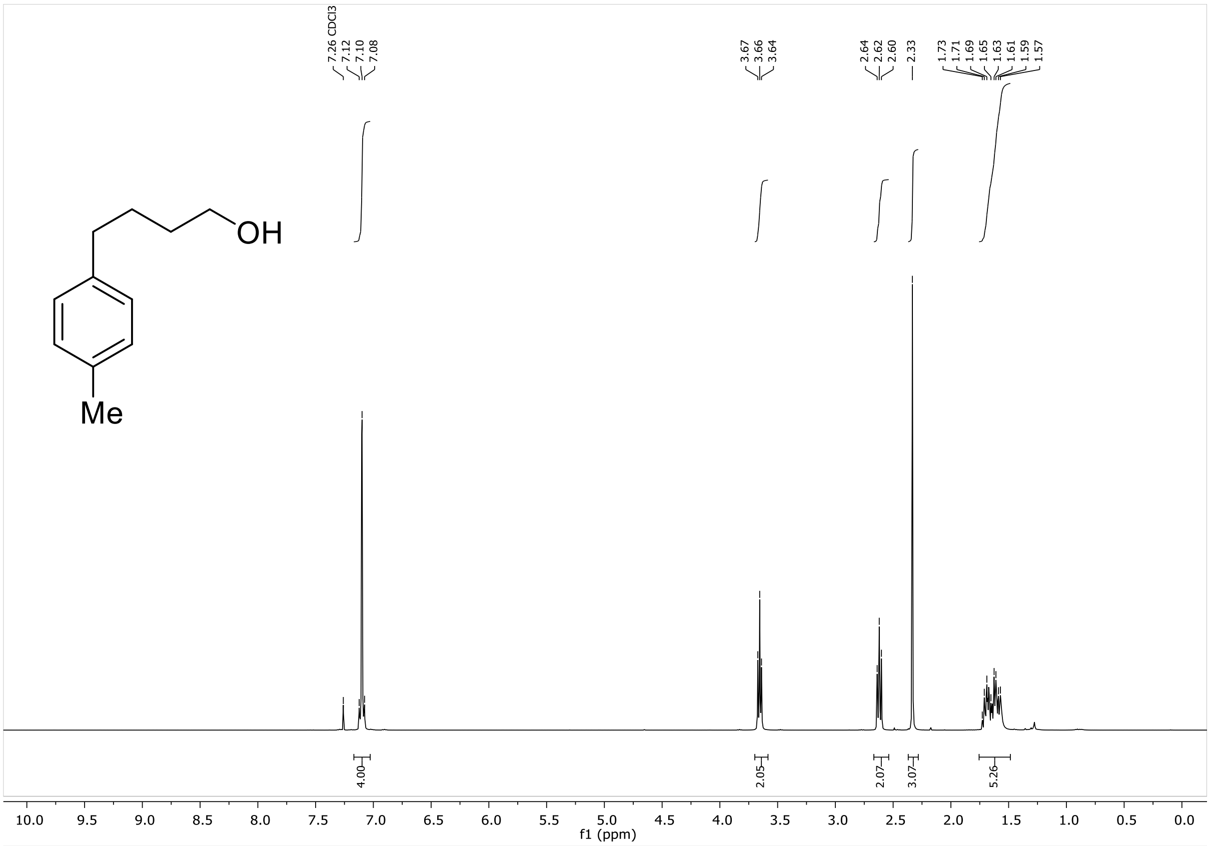

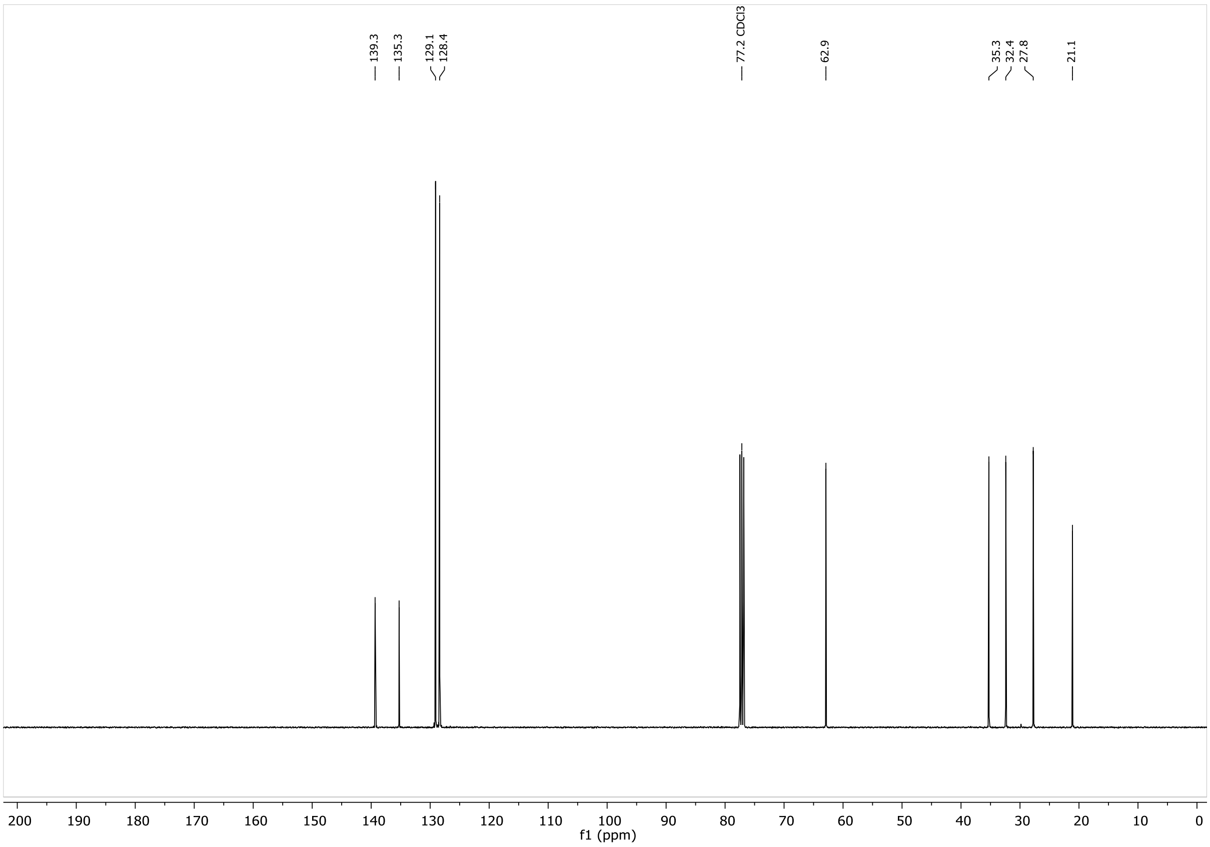

4-(3,5-dimethylphenyl)butan-1-ol (10n)

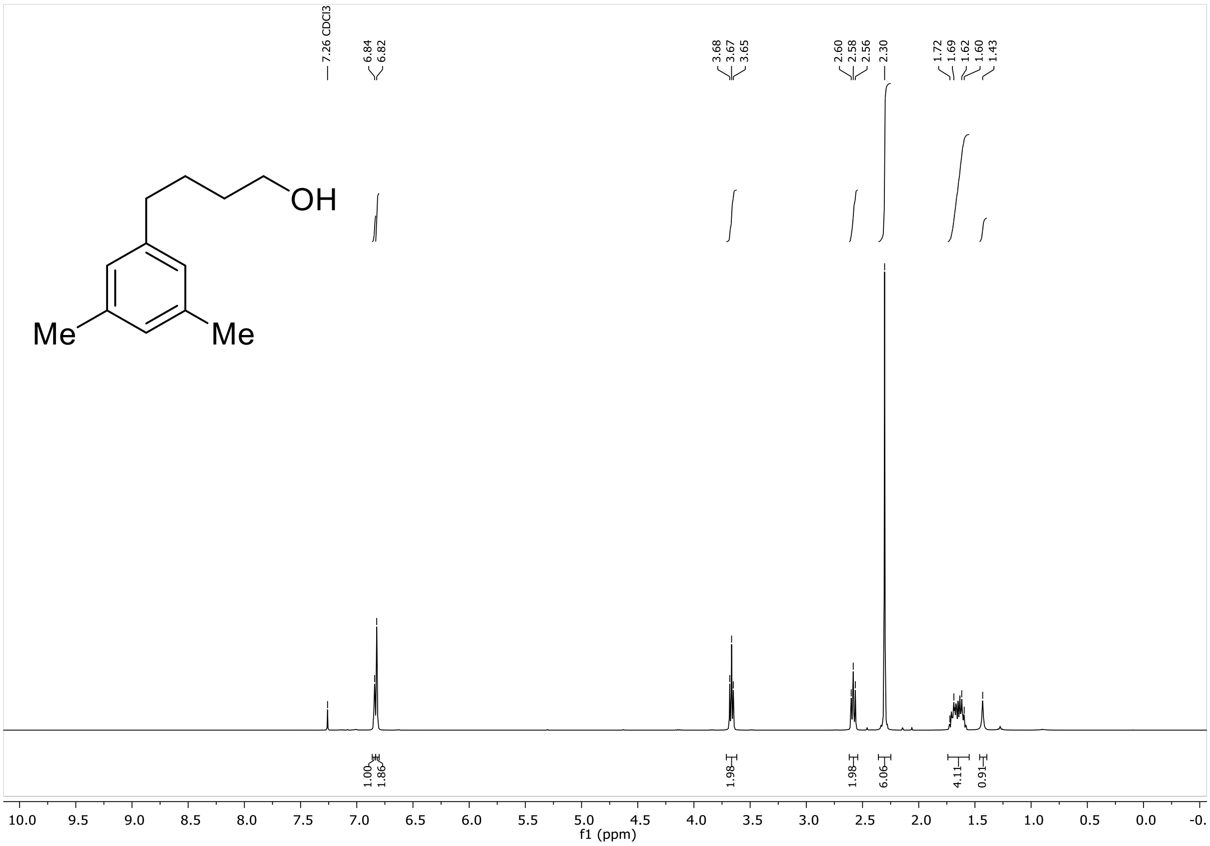

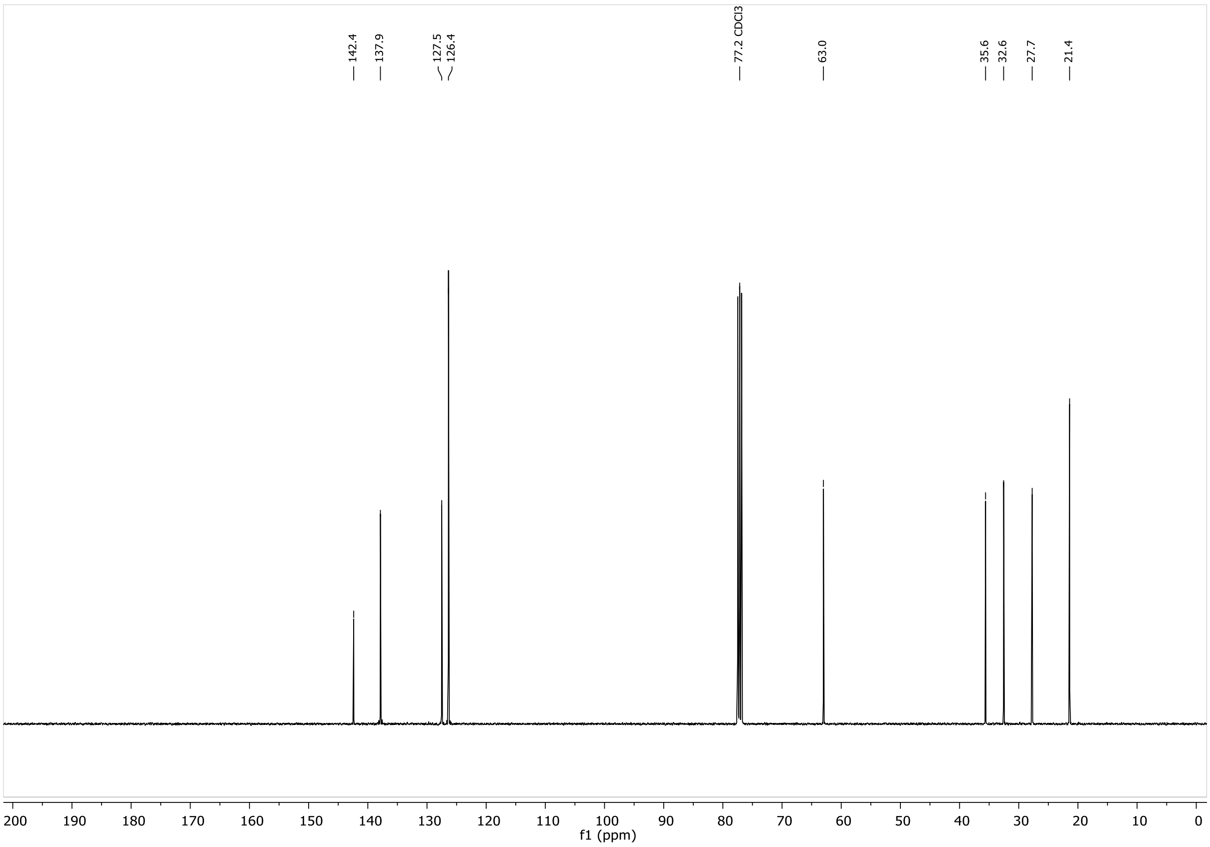

(2,6-dichlorophenyl)(phenyl)methanol (10o)

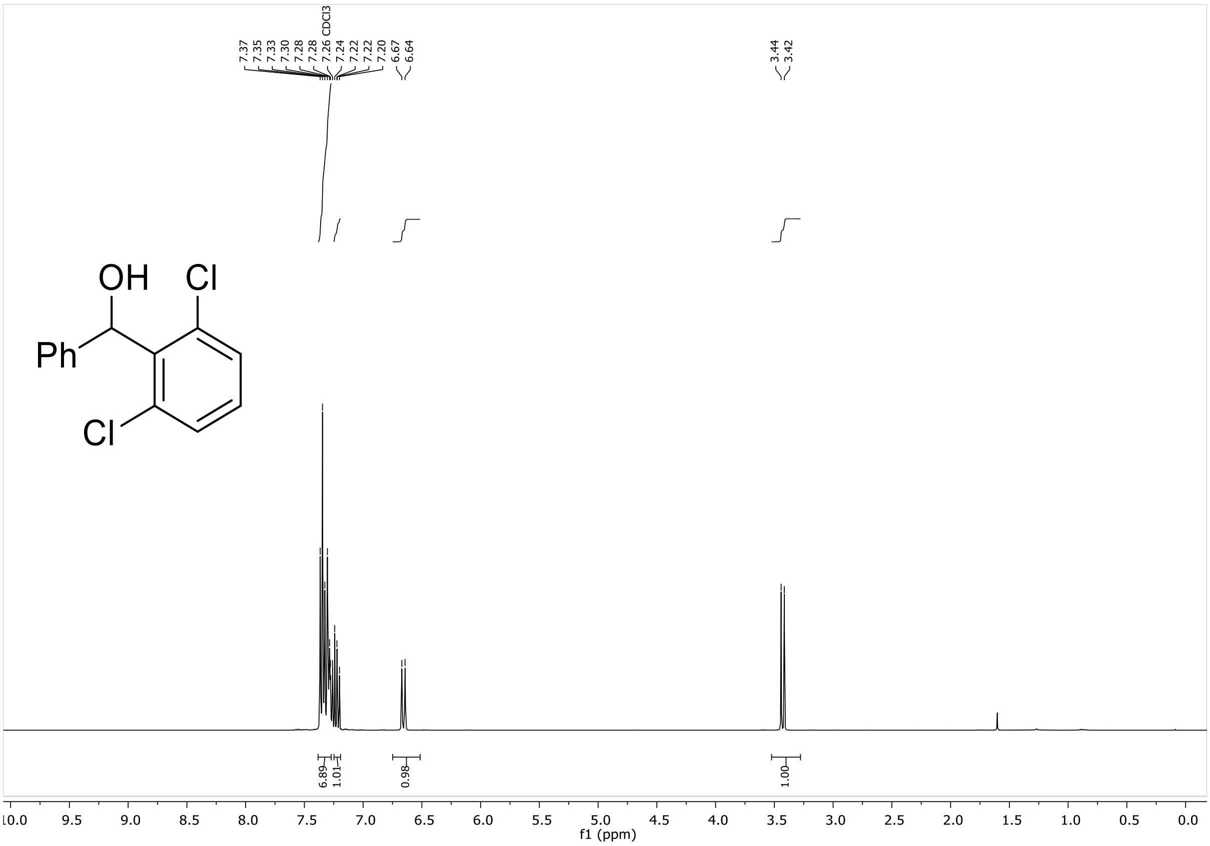

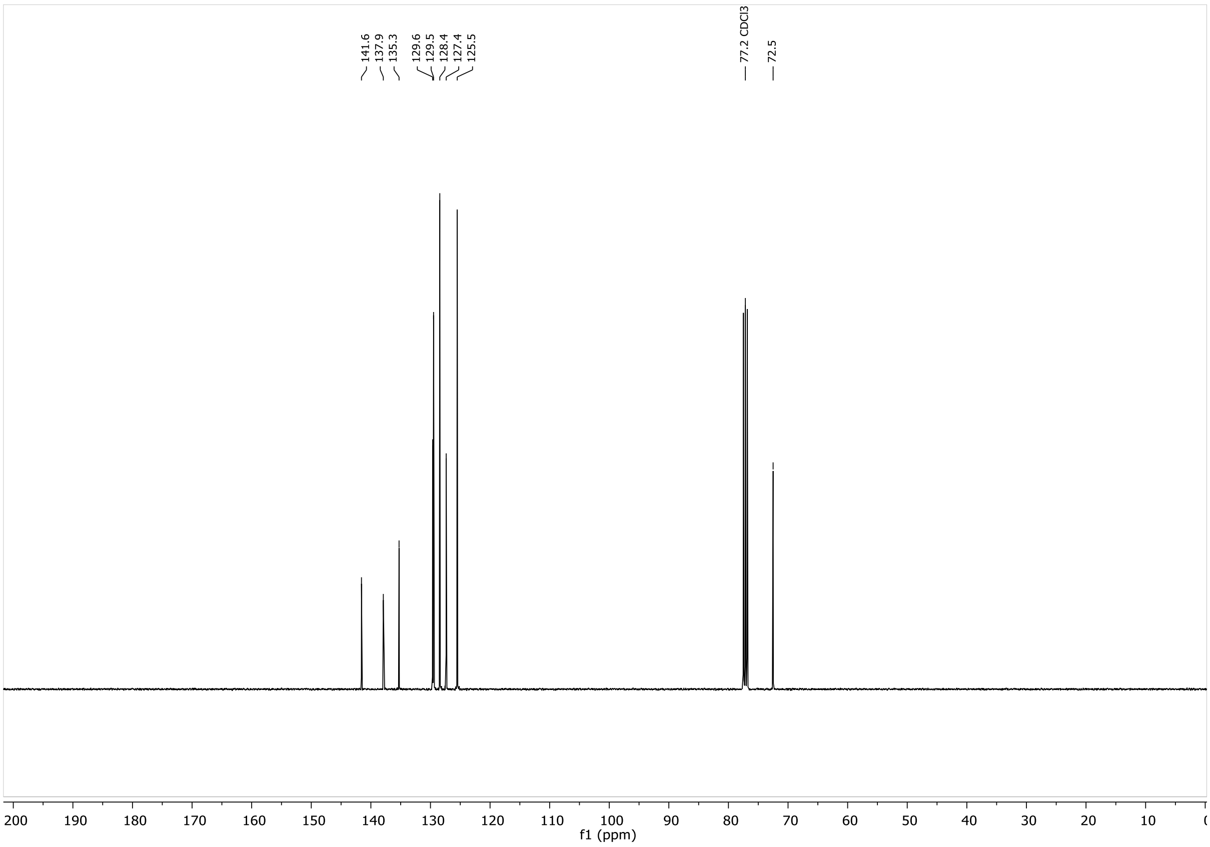

dicyclopropyl(phenyl)methanol (10p)

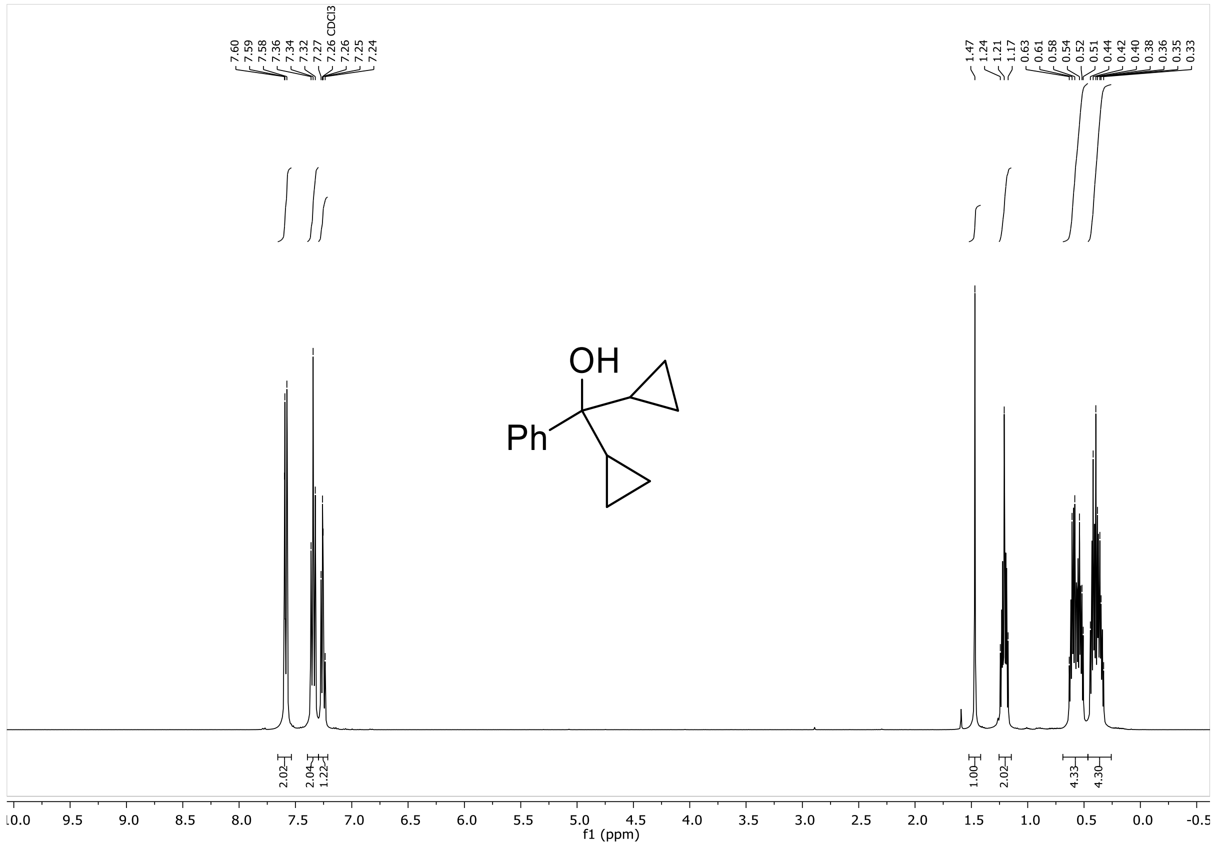

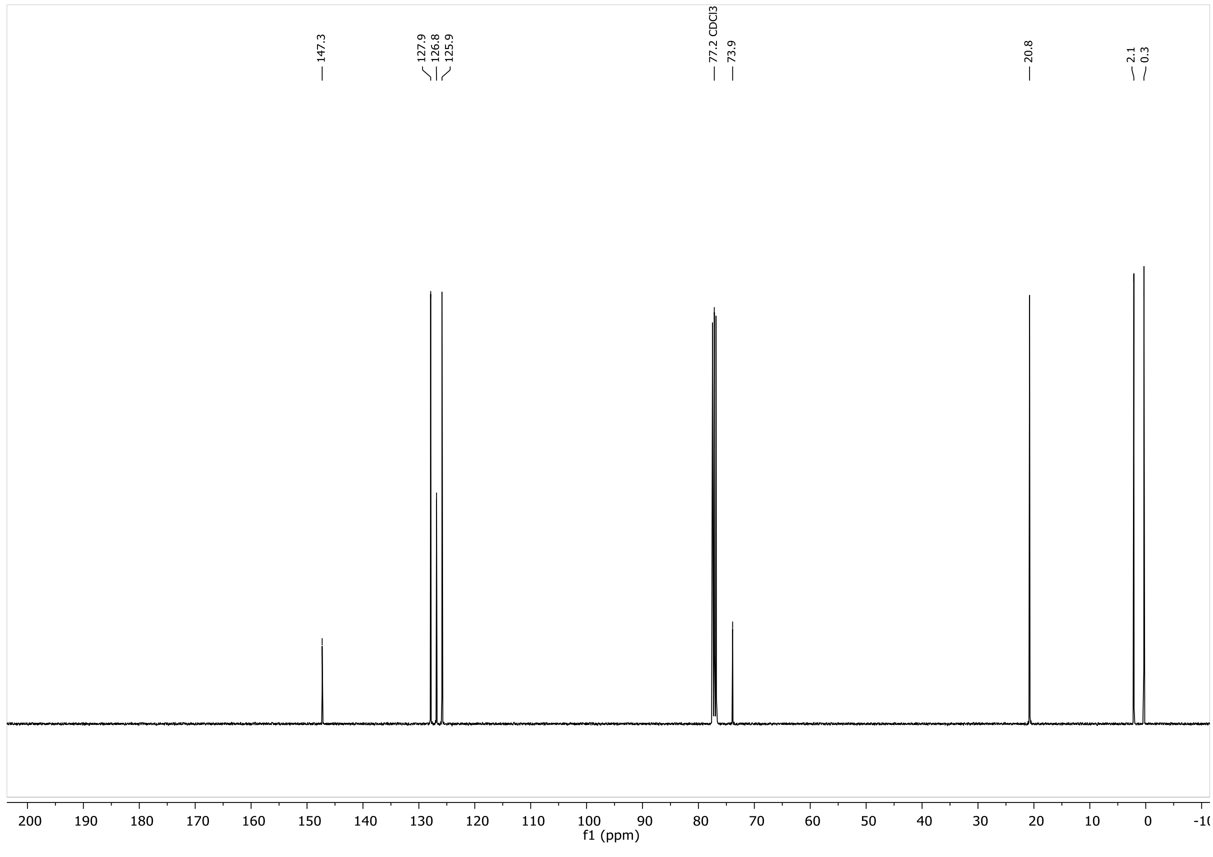

2-phenyladamantan-2-ol (10q)

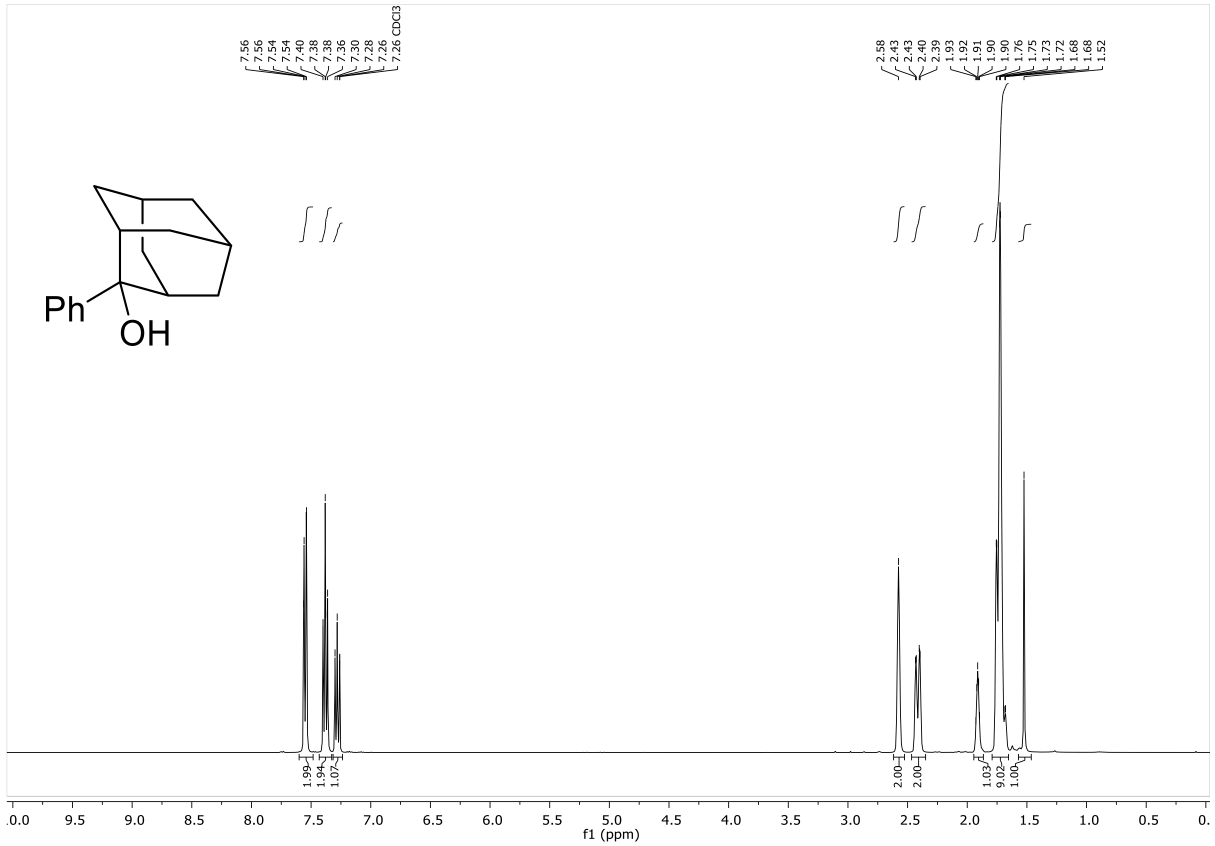

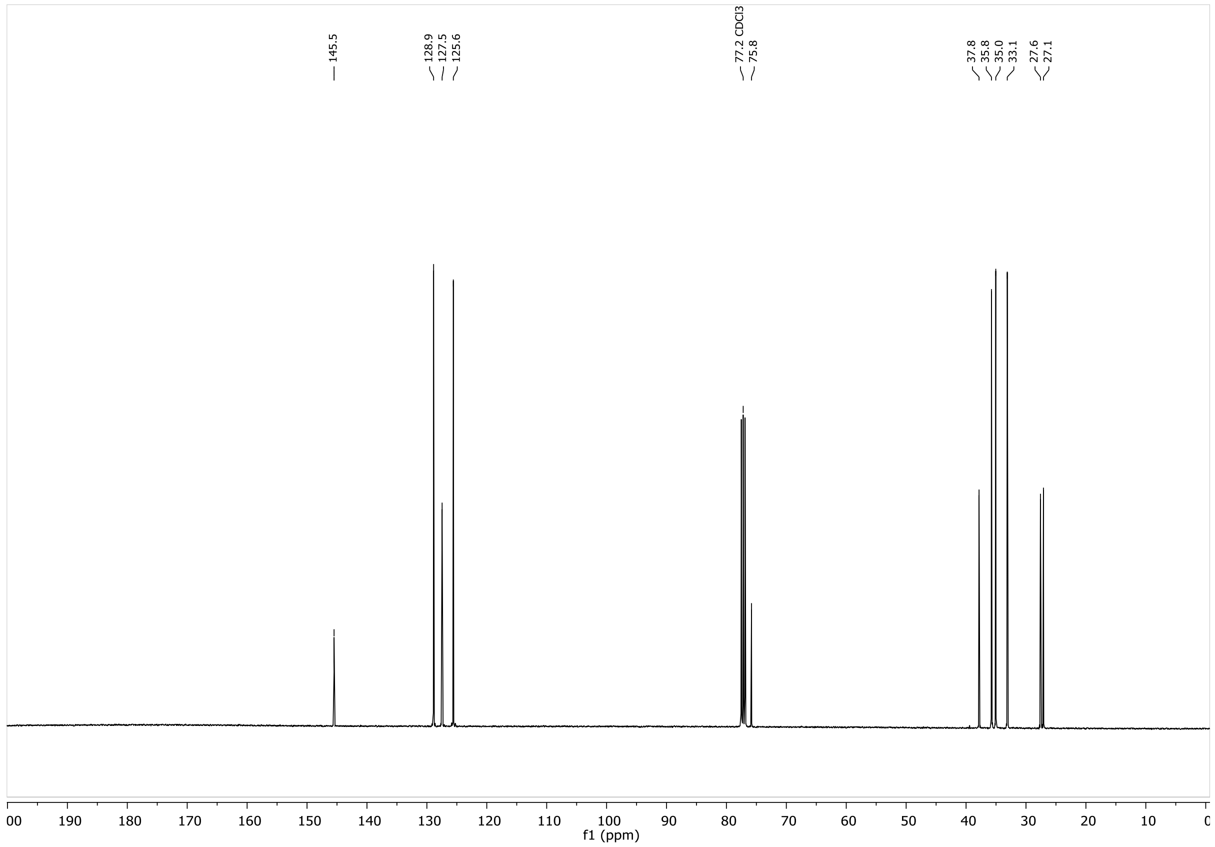

N-benzhydrylaniline (10r)

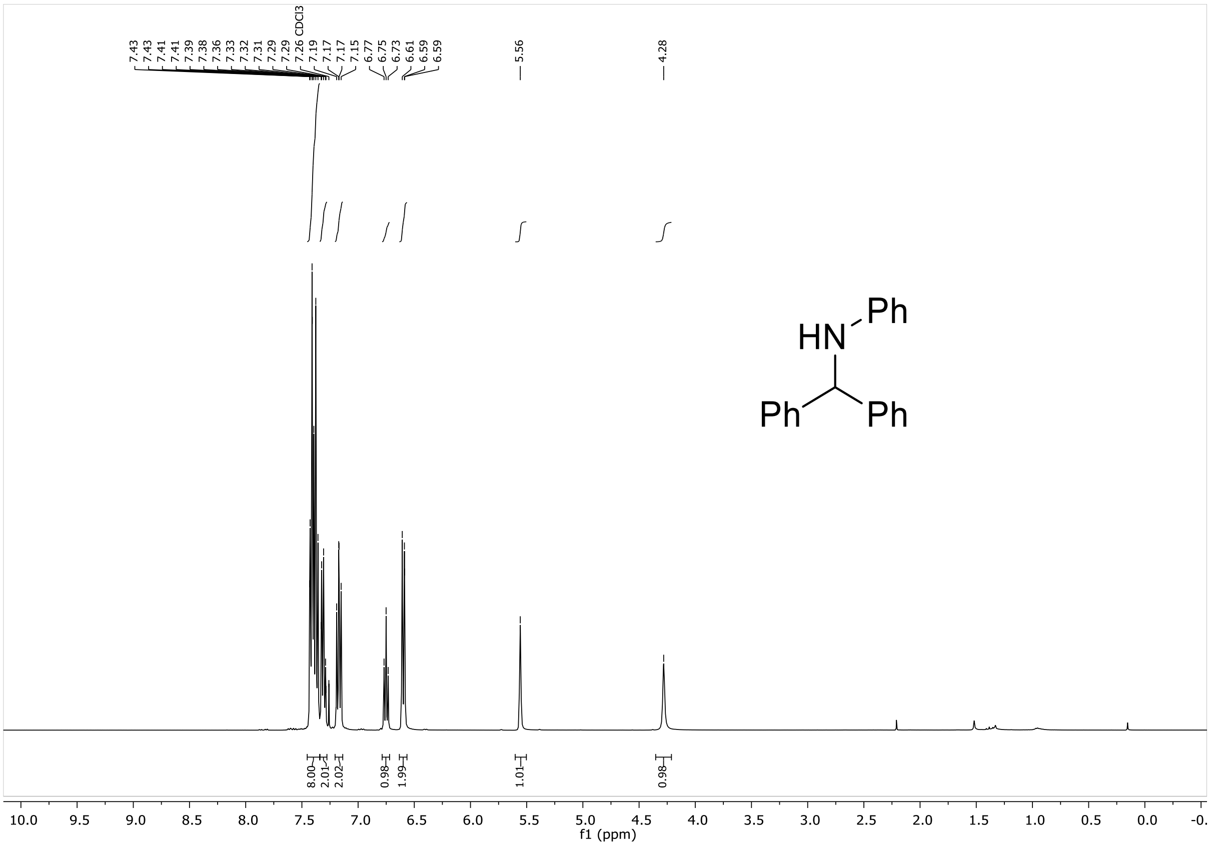

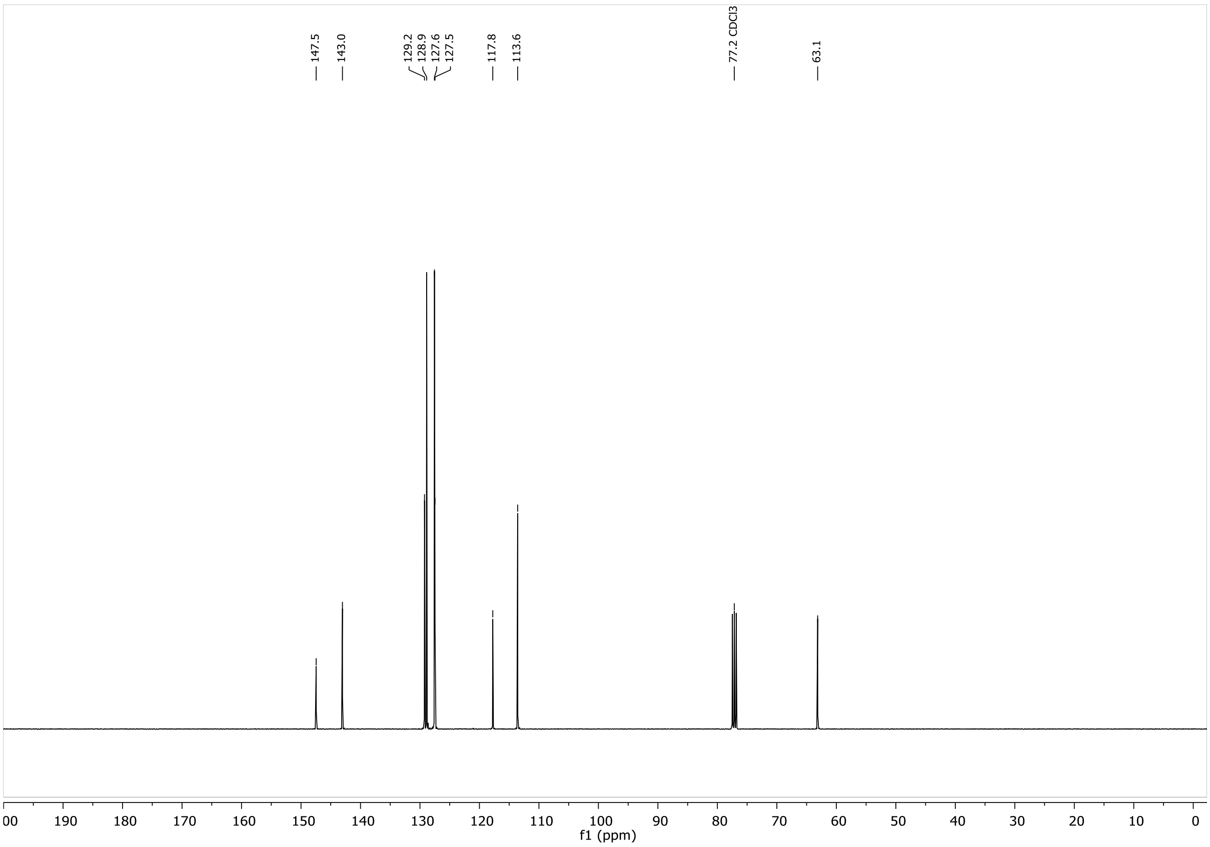

2-benzyladamantan-2-ol (10s)

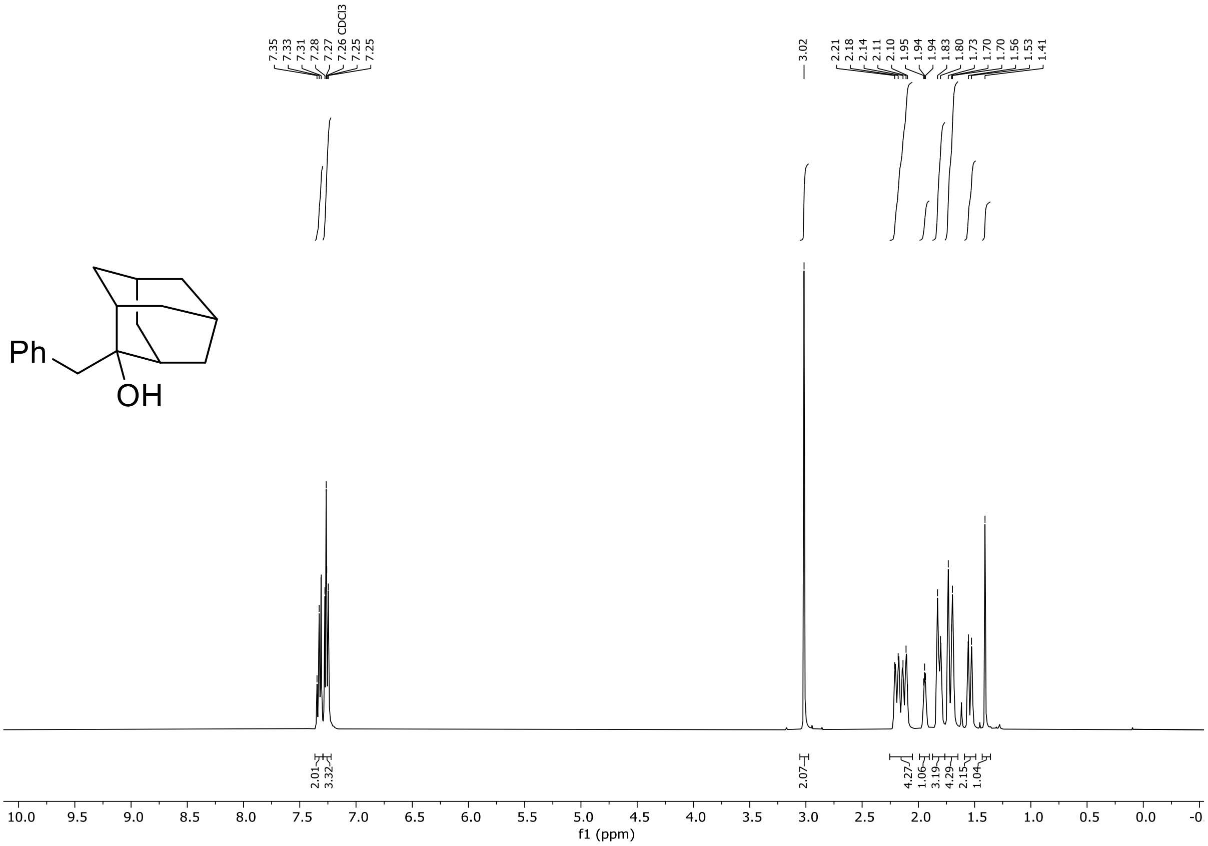

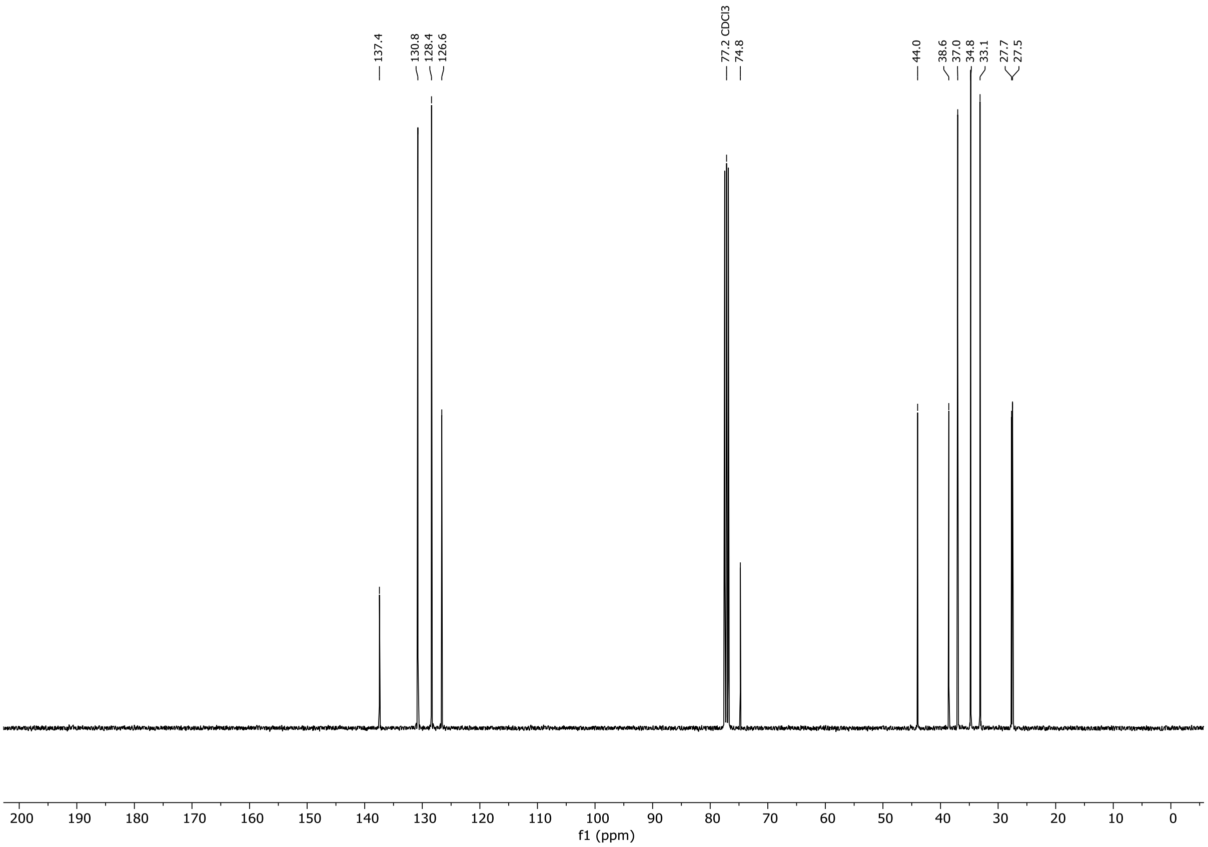

(*S*)-1-(benzyloxy)-4-phenylbutan-2-ol (10t)

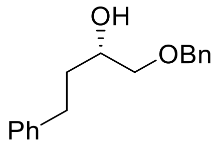

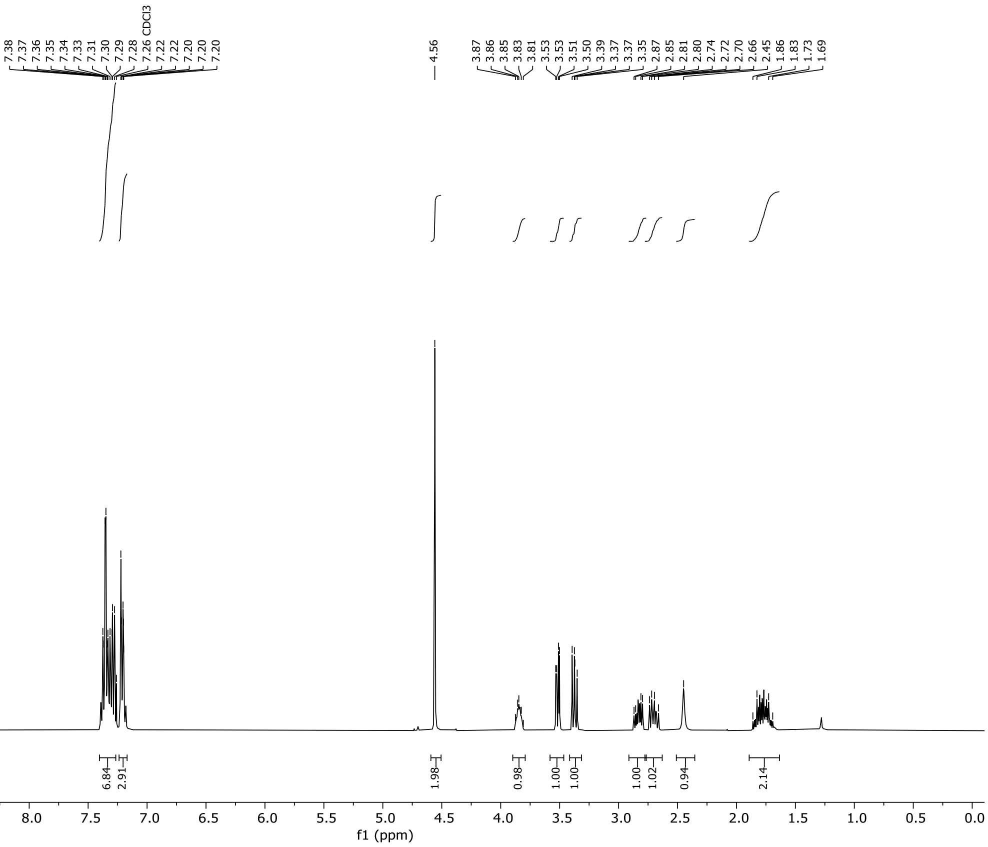

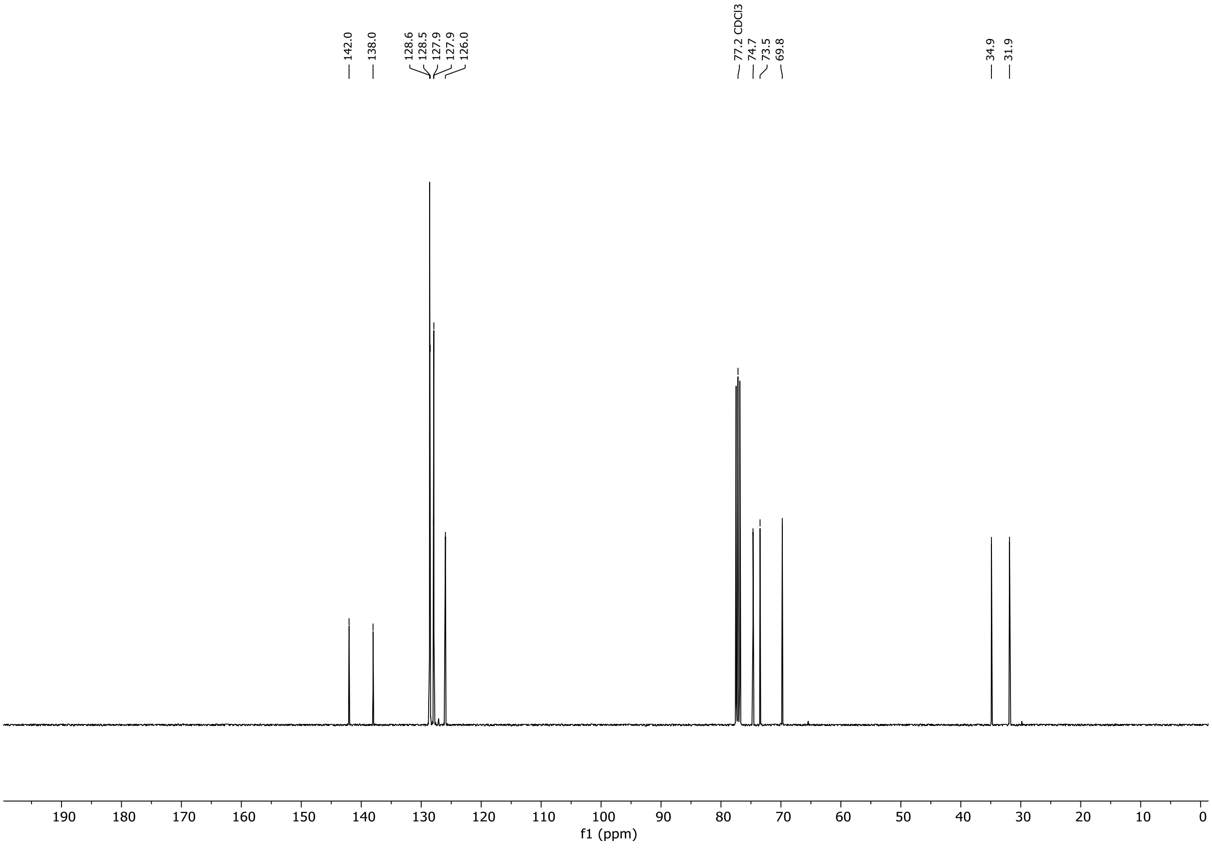

(*S*)-1-((tert-butyldimethylsilyl)oxy)-4-phenylbutan-2-ol (10u)

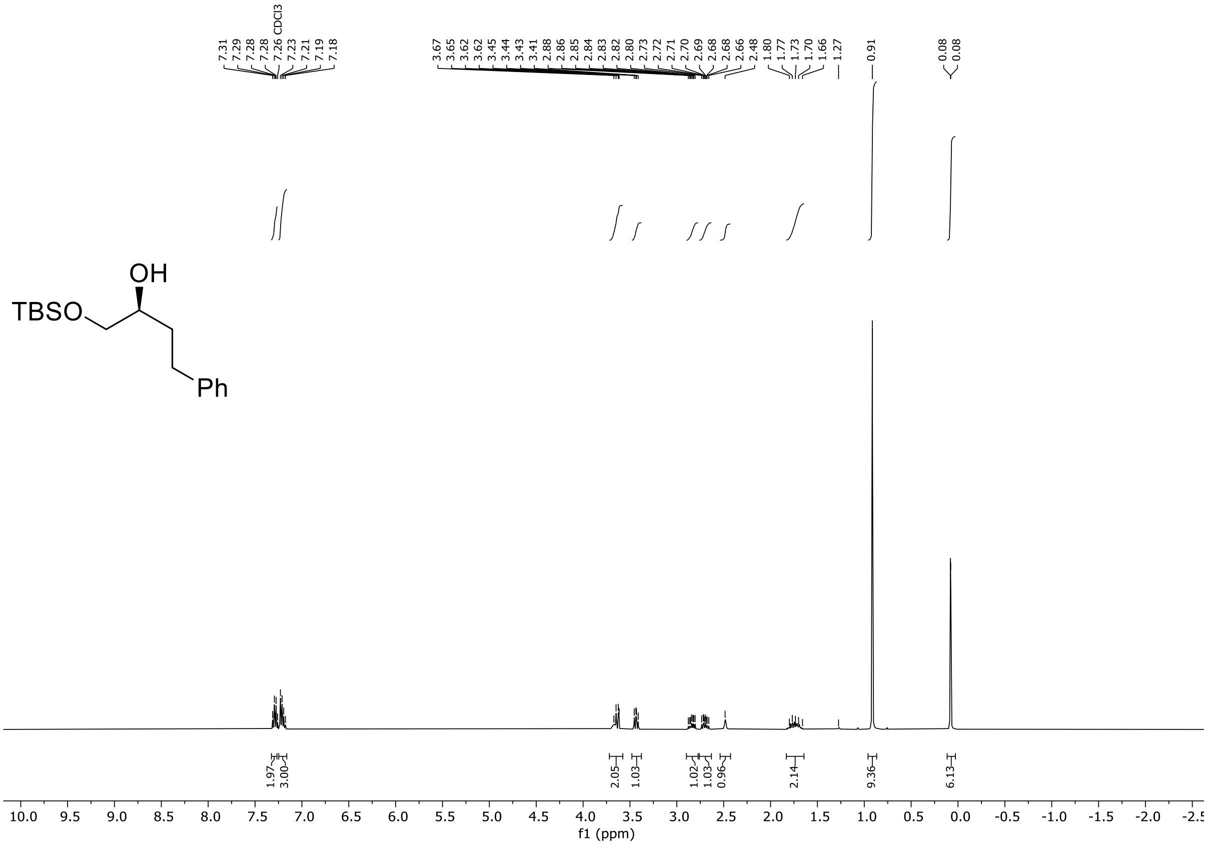

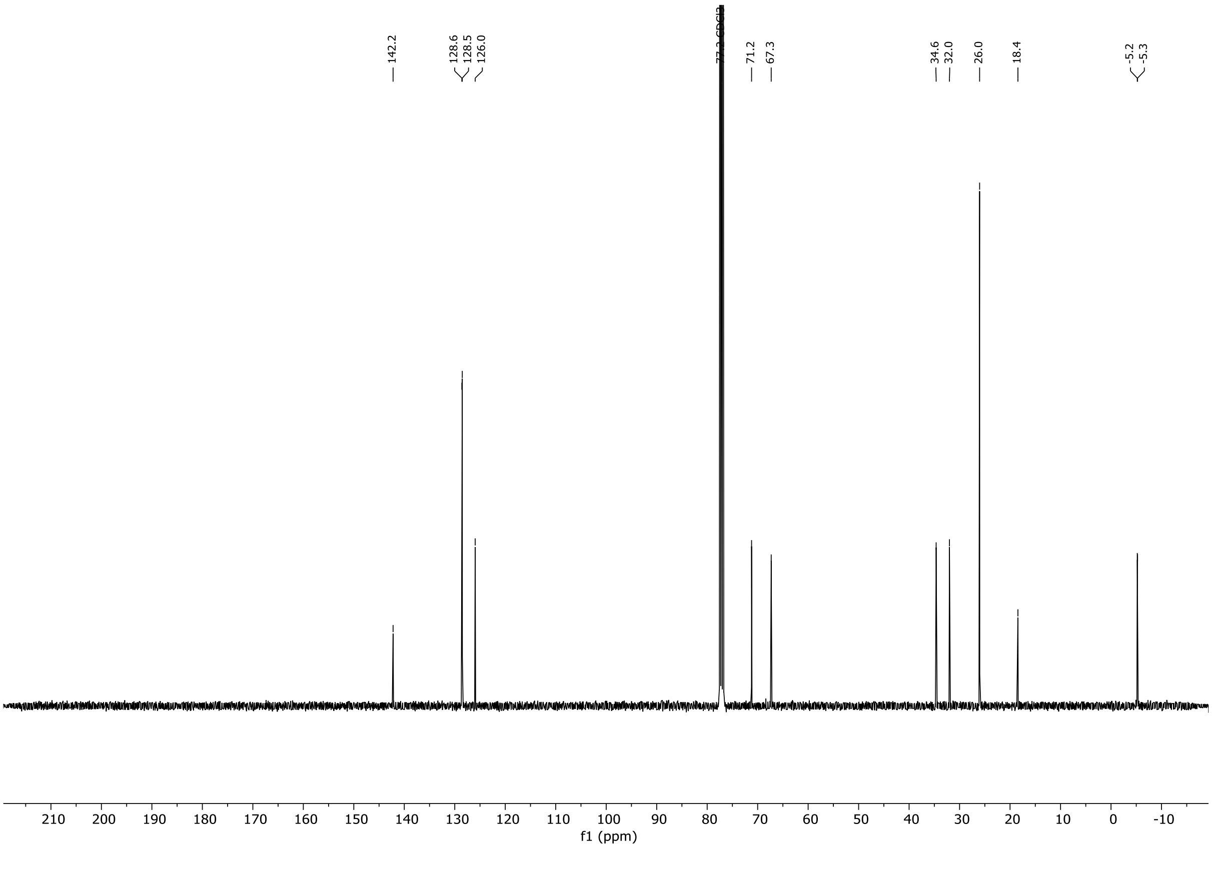

(*S*)-2-((tert-butyldimethylsilyl)oxy)-4-phenylbutan-1-ol (10u)

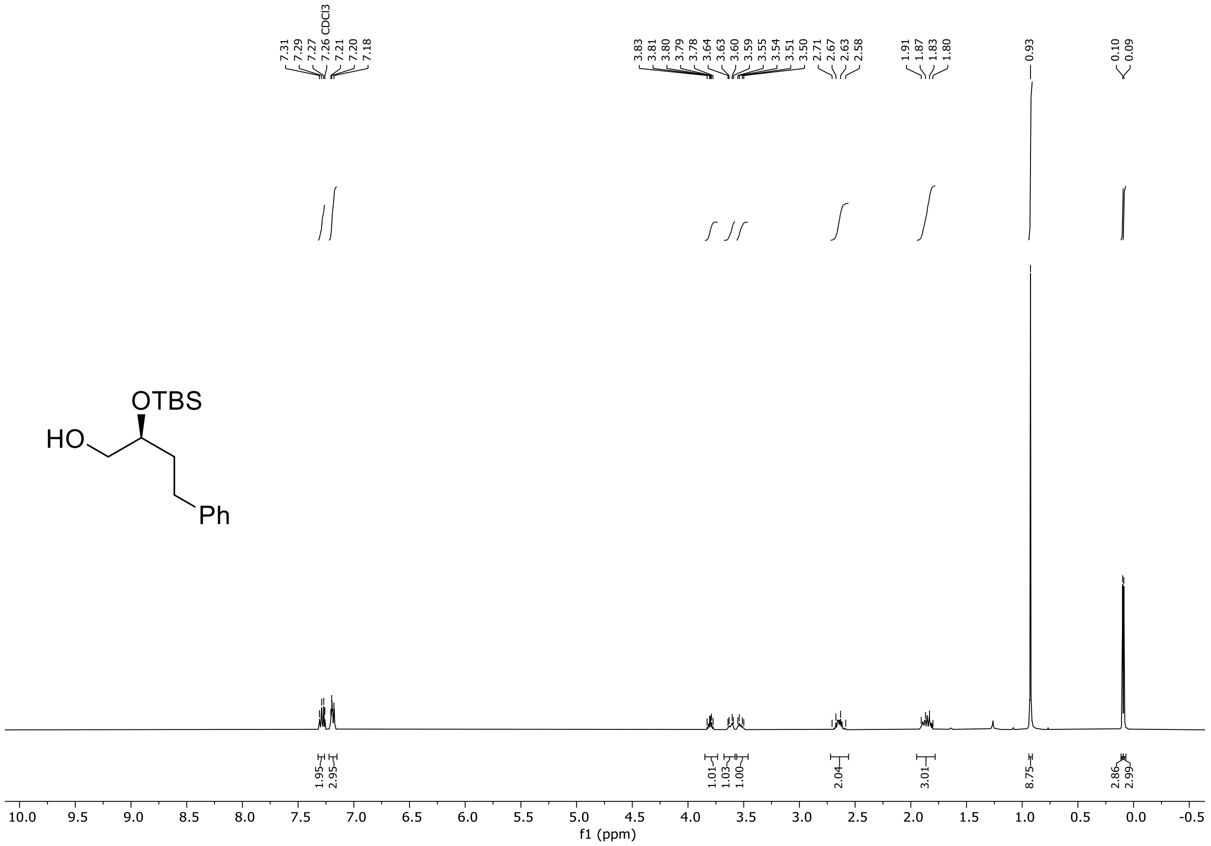

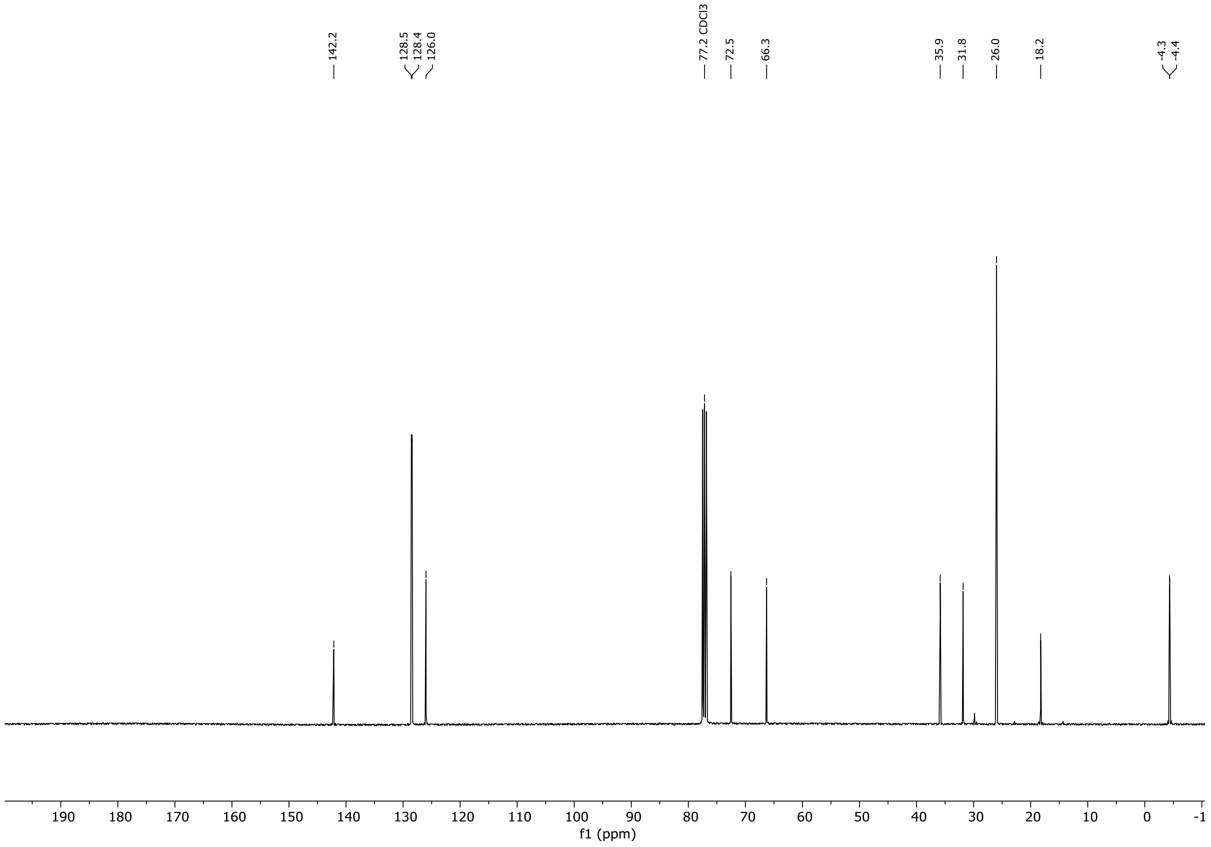

4-phenylbutane-1,2-diol (11u)

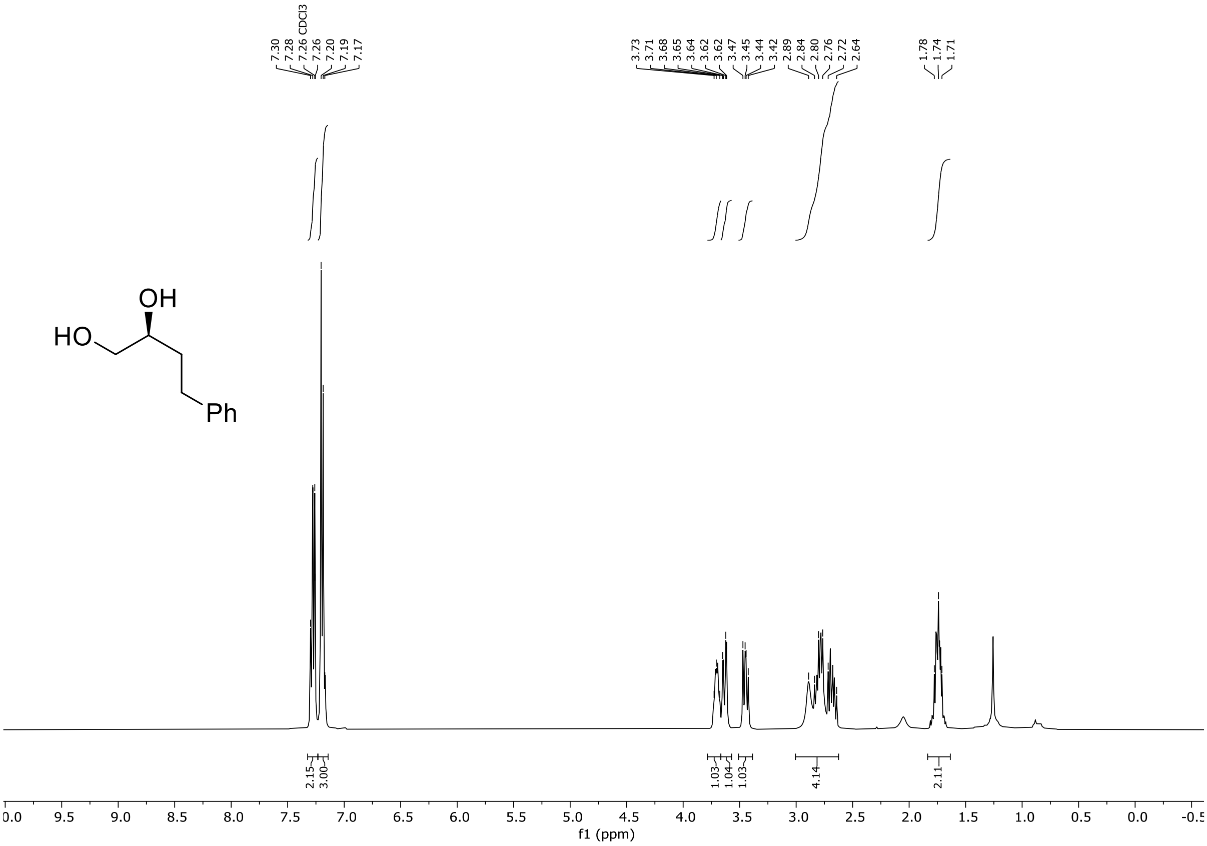

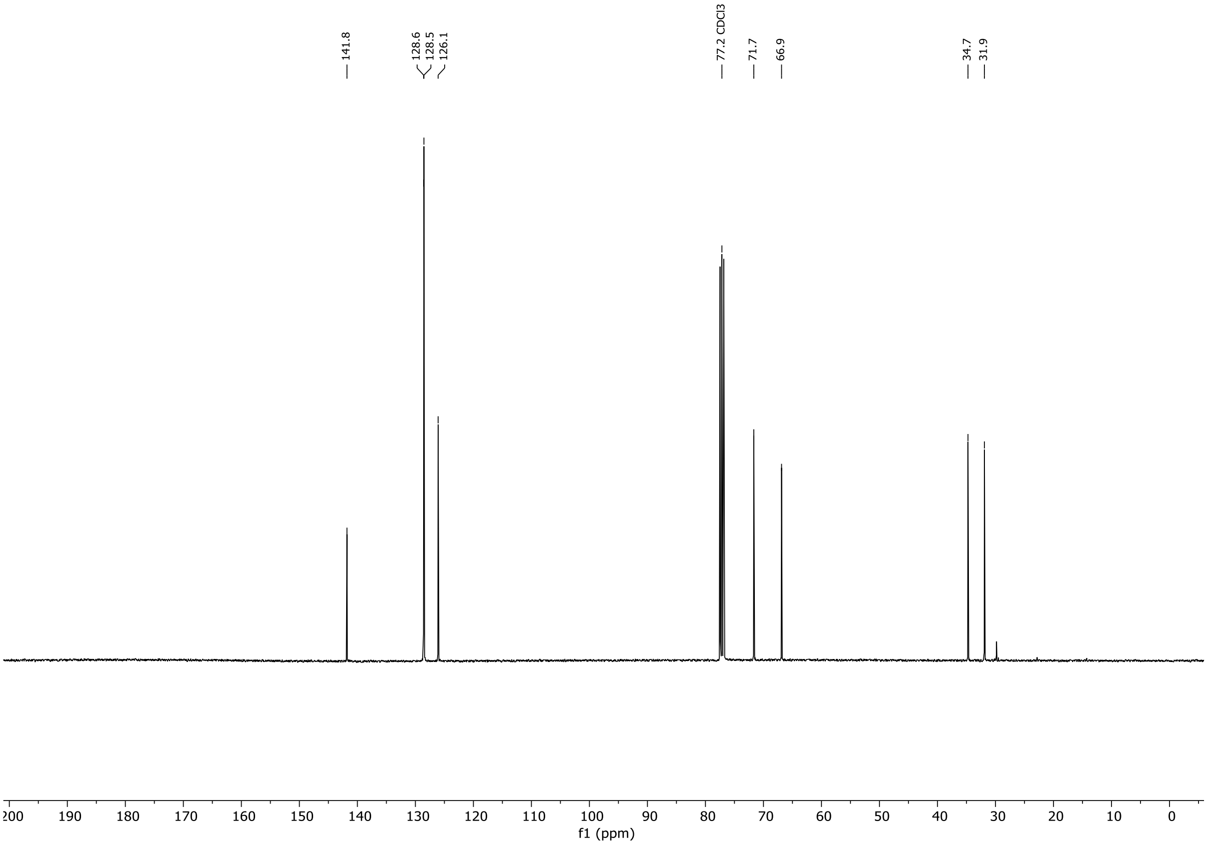

trans-2-(naphthalen-1-ylmethyl)cyclohexan-1-ol (10v)

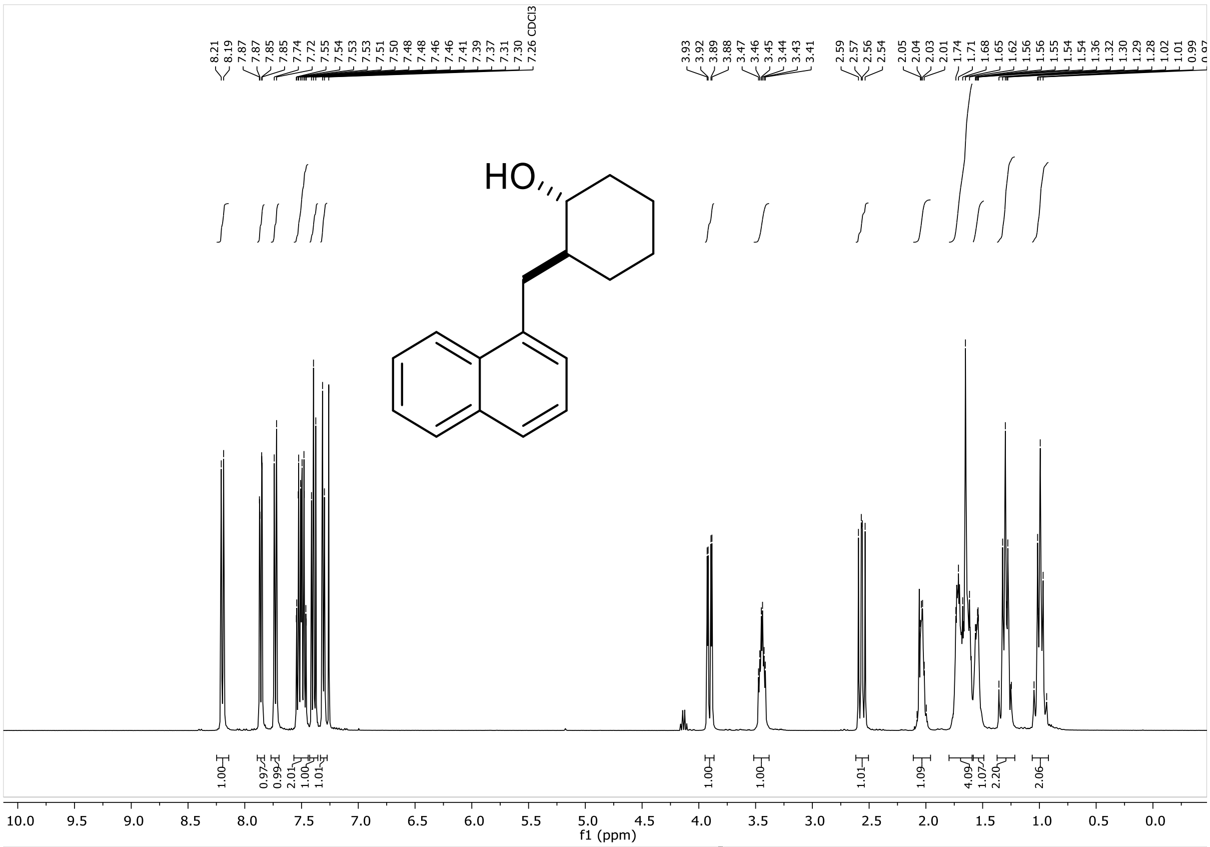

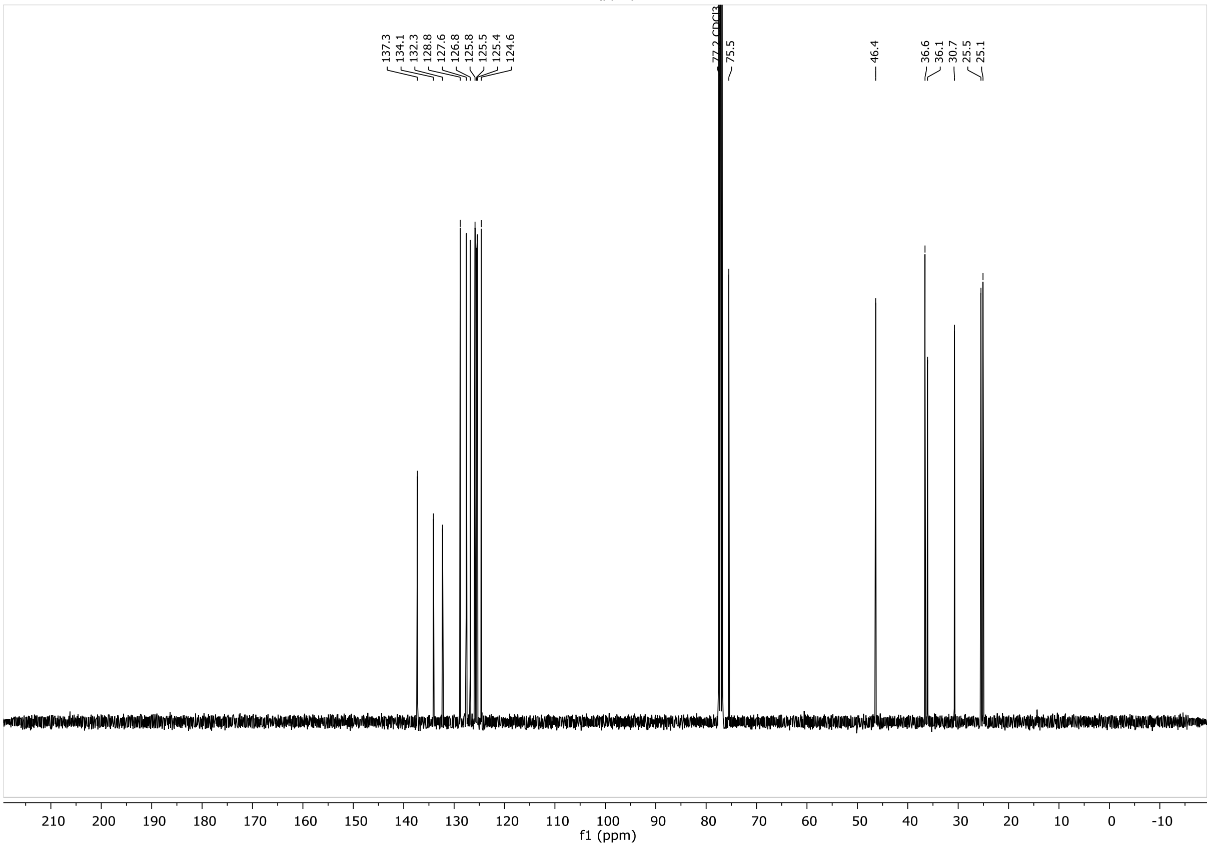

2-methyl-4-(naphthalen-1-yl)butan-2-ol (10w)

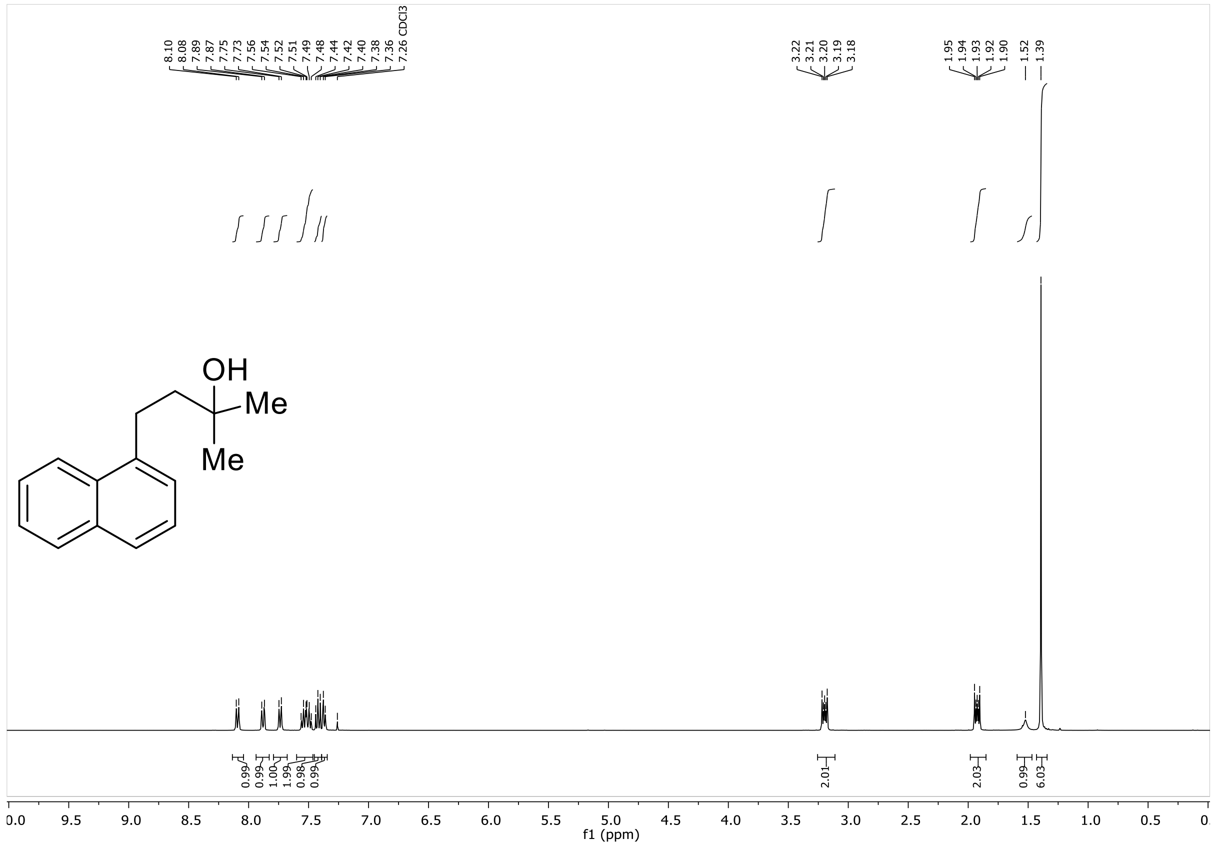

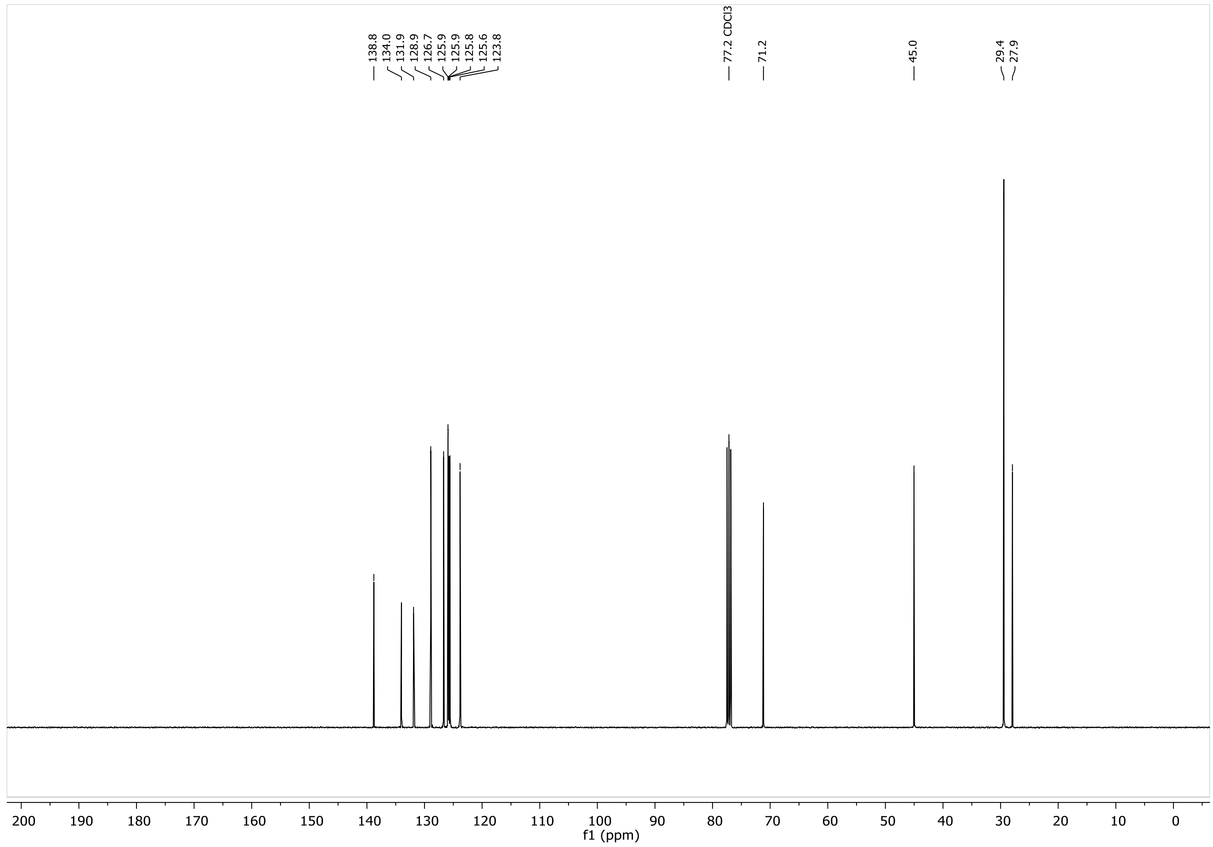

4-(naphthalen-1-yl)butan-1-ol (10x)

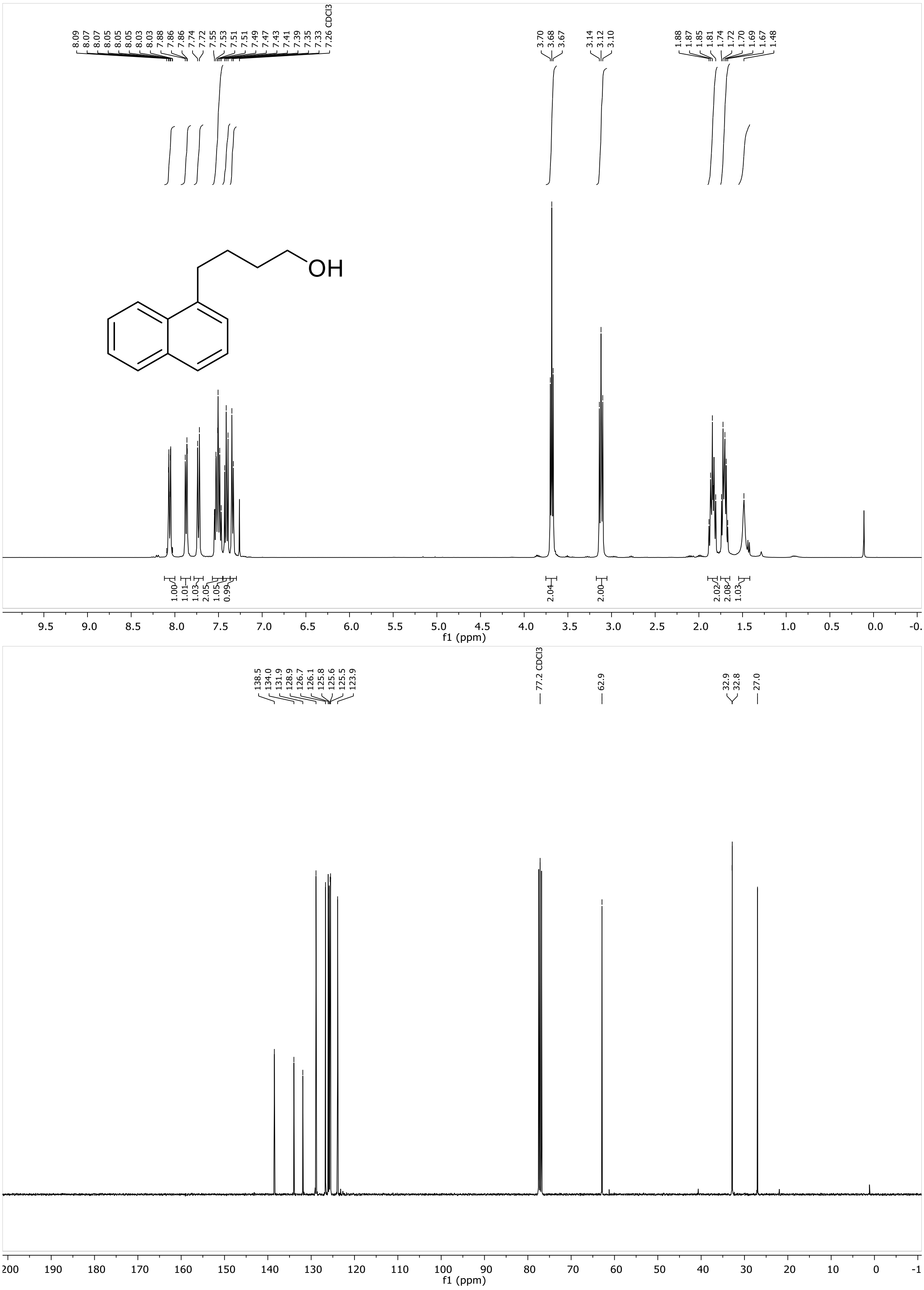

2-(naphthalen-1-yl)-1,1-diphenylethan-1-ol (10y)

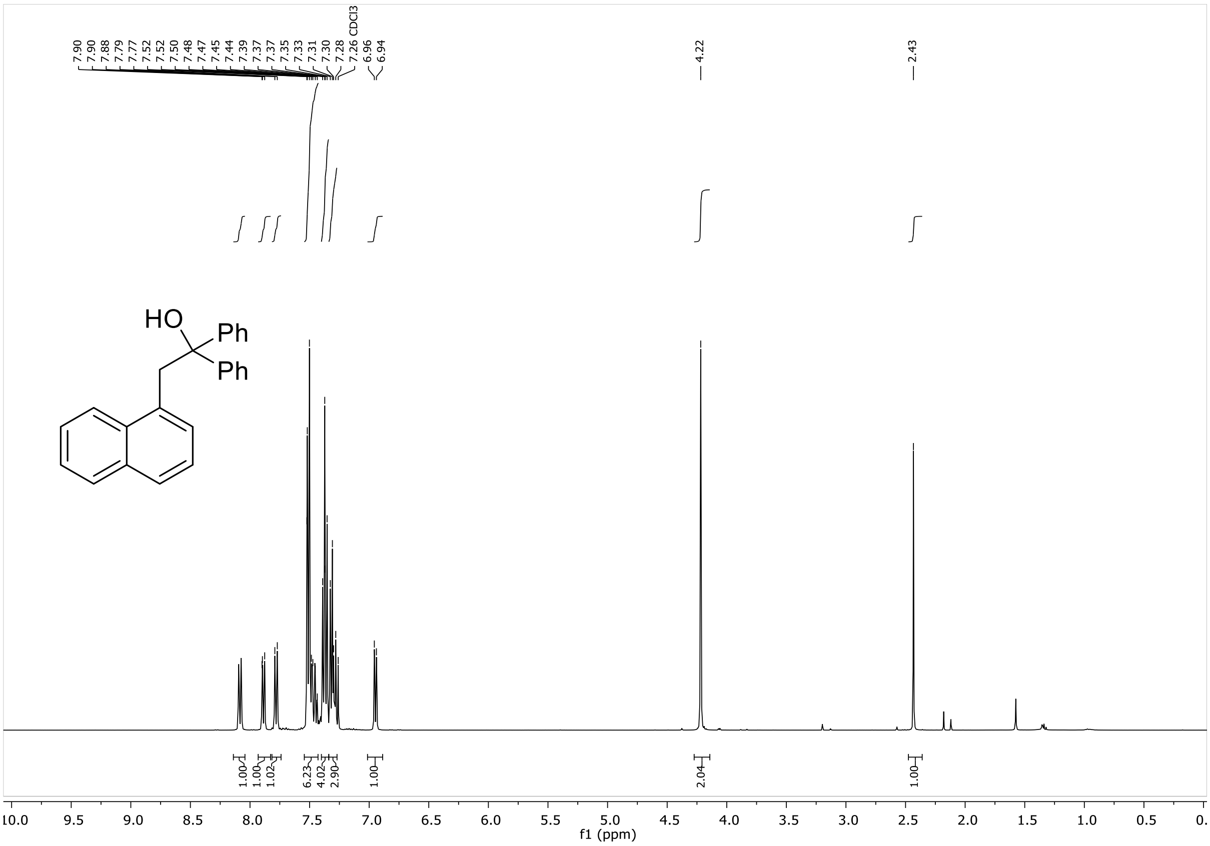

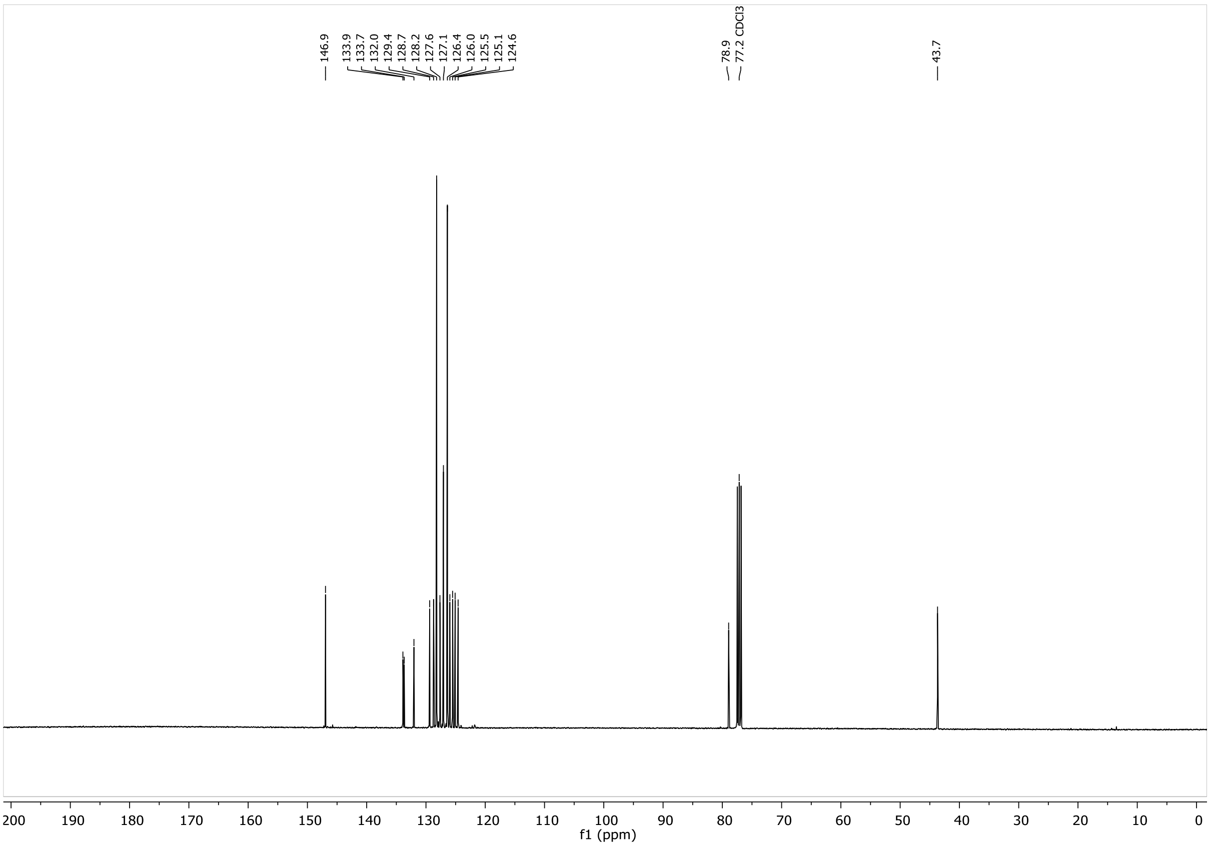

1,1-dicyclopropyl-2-(4-ethylphenyl)ethan-1-ol (10z)

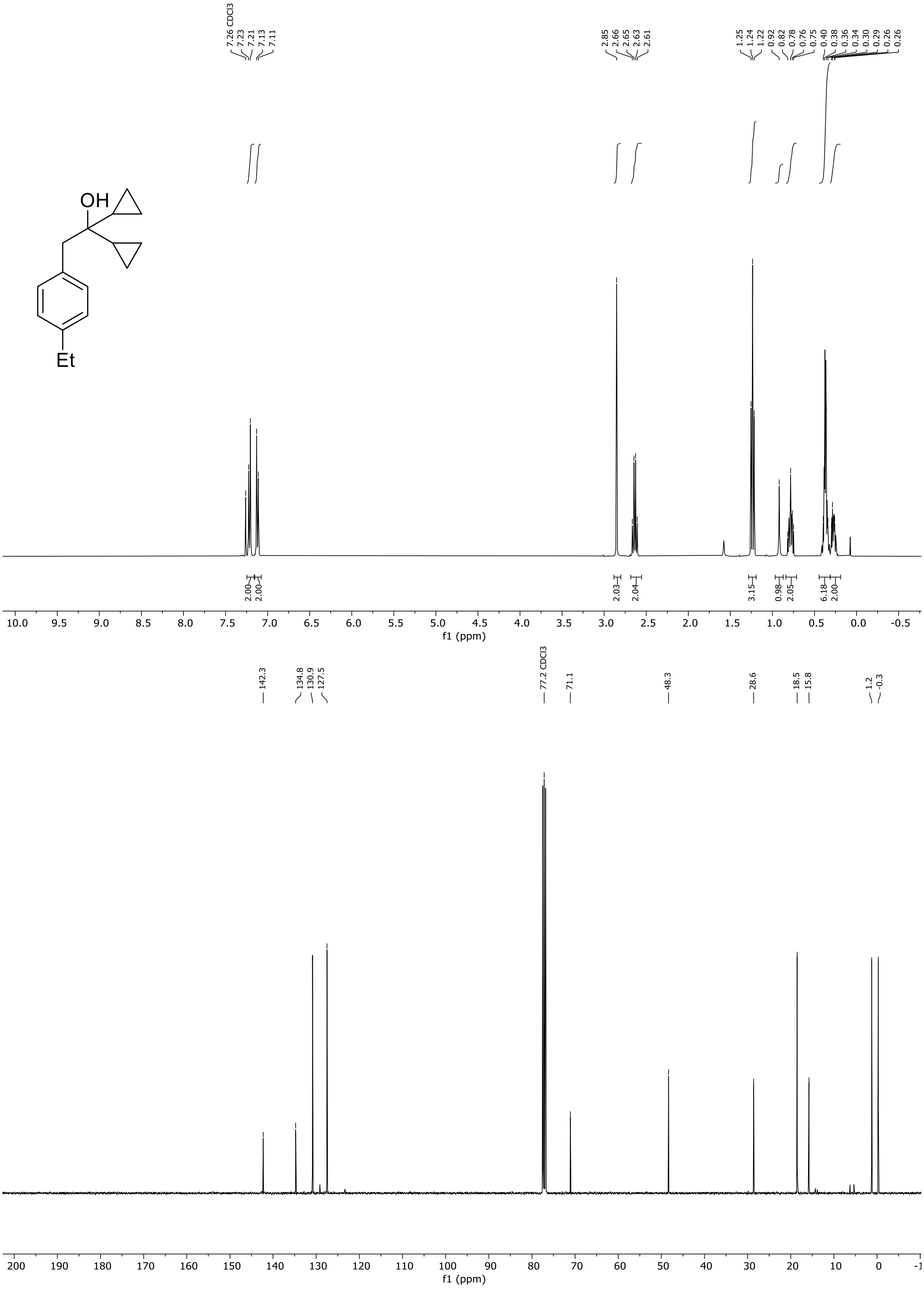

***trans*-2-(4-ethylbenzyl)cyclohexan-1-ol (10aa)**

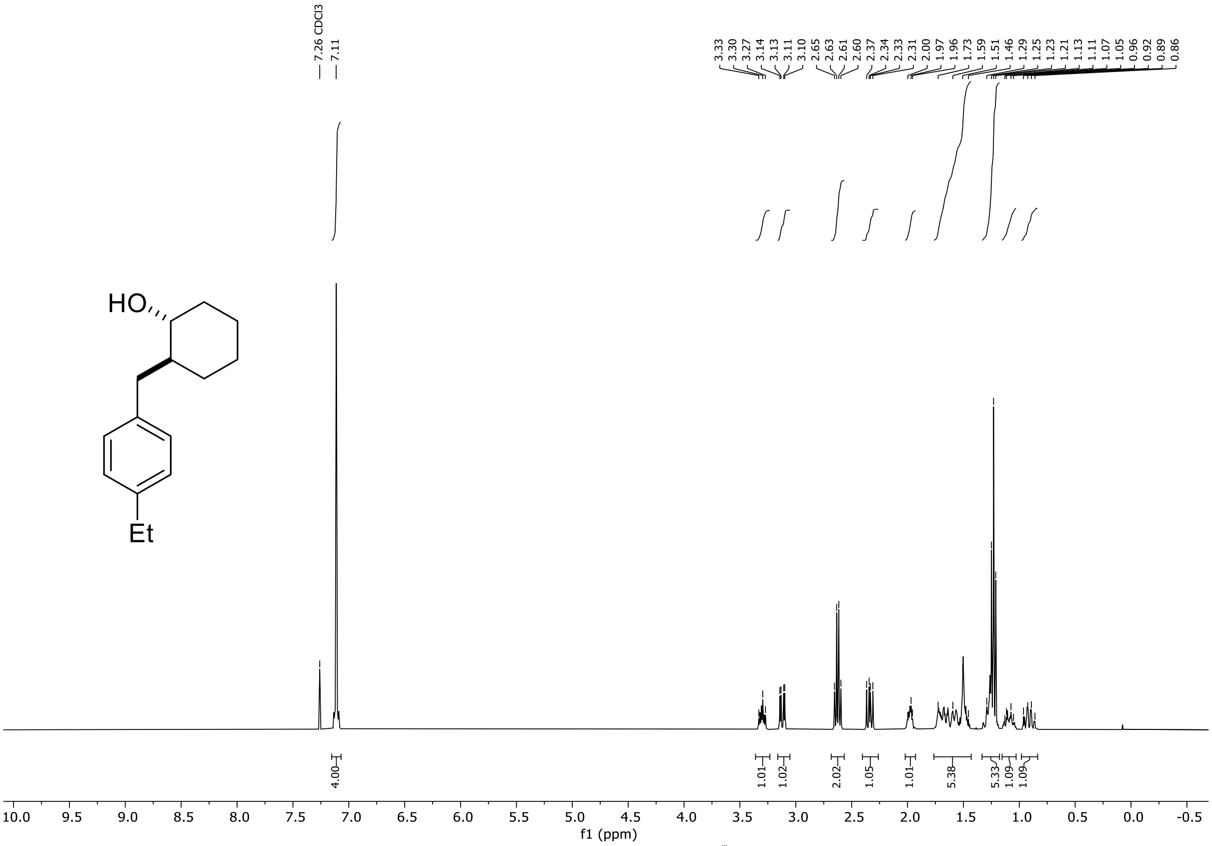

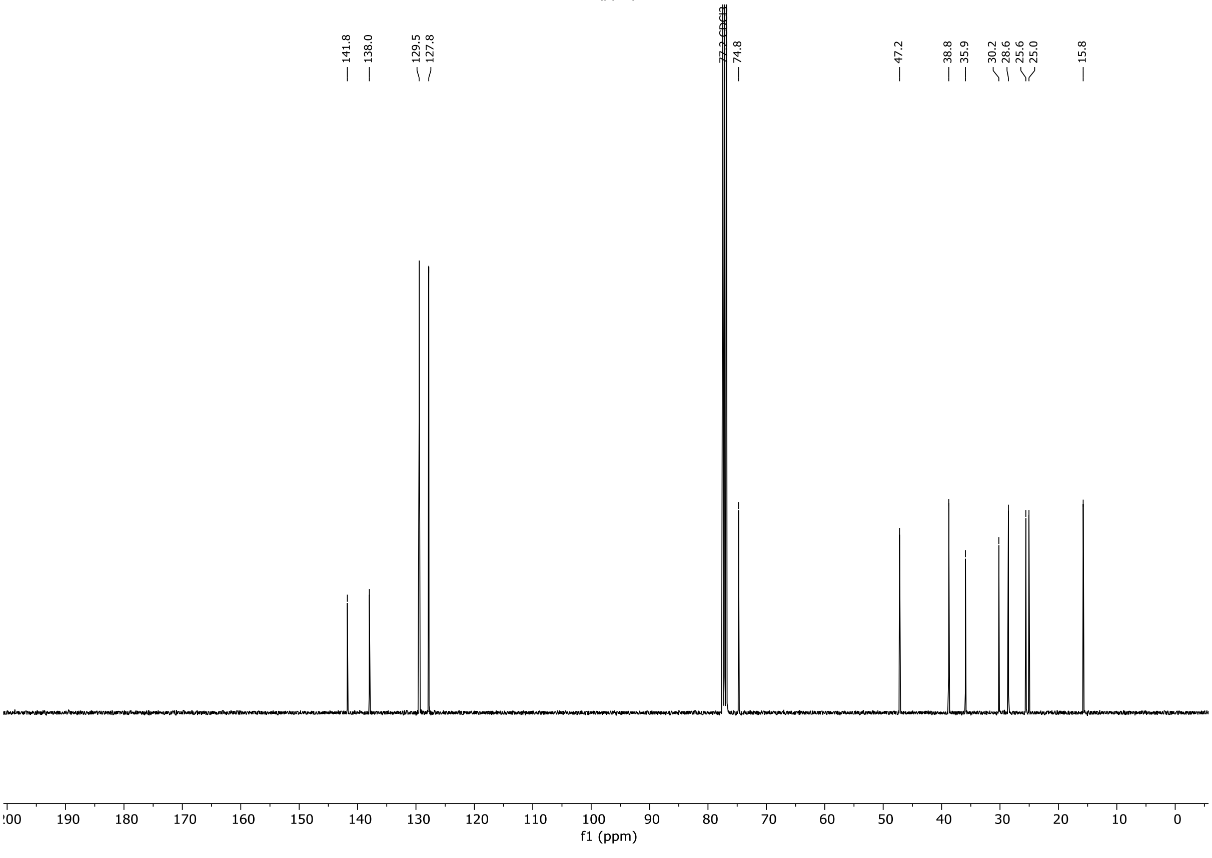

4-(1-phenylethyl)heptan-4-ol (10ab)

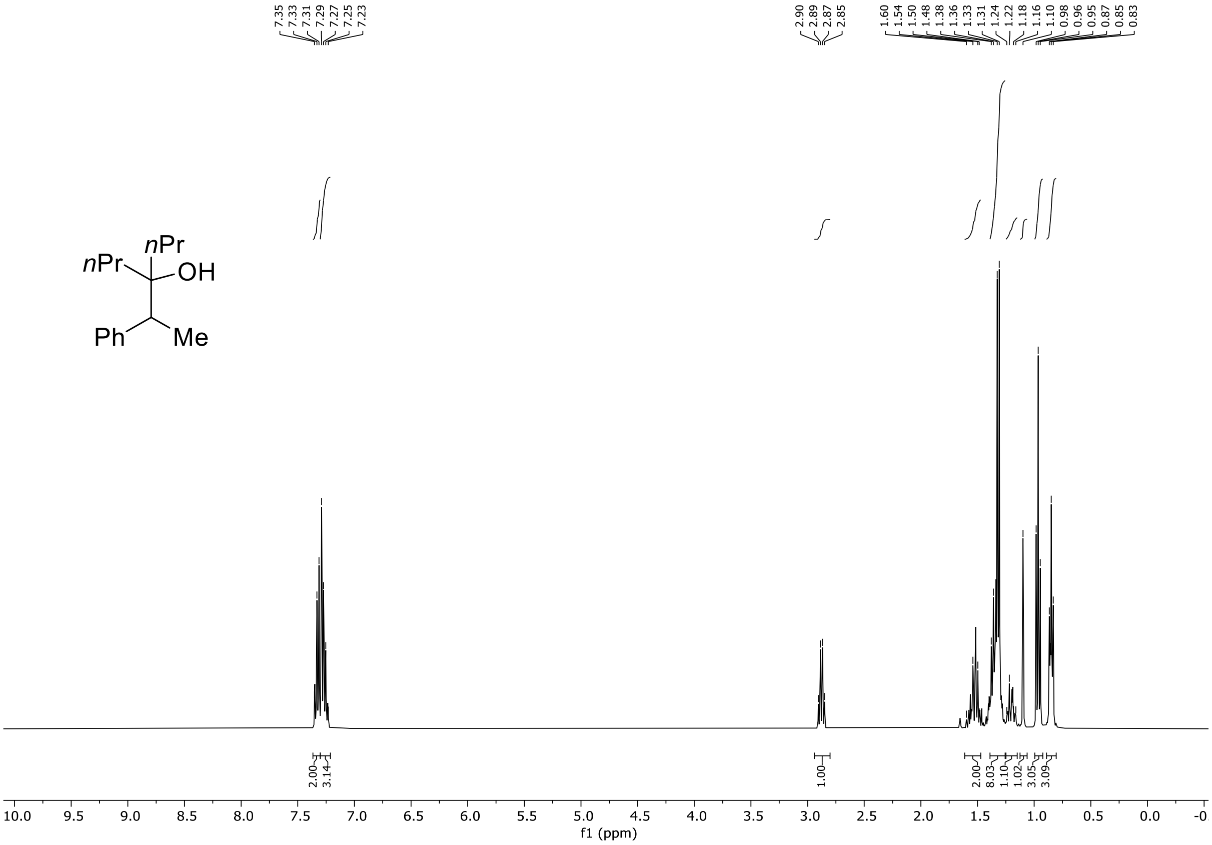

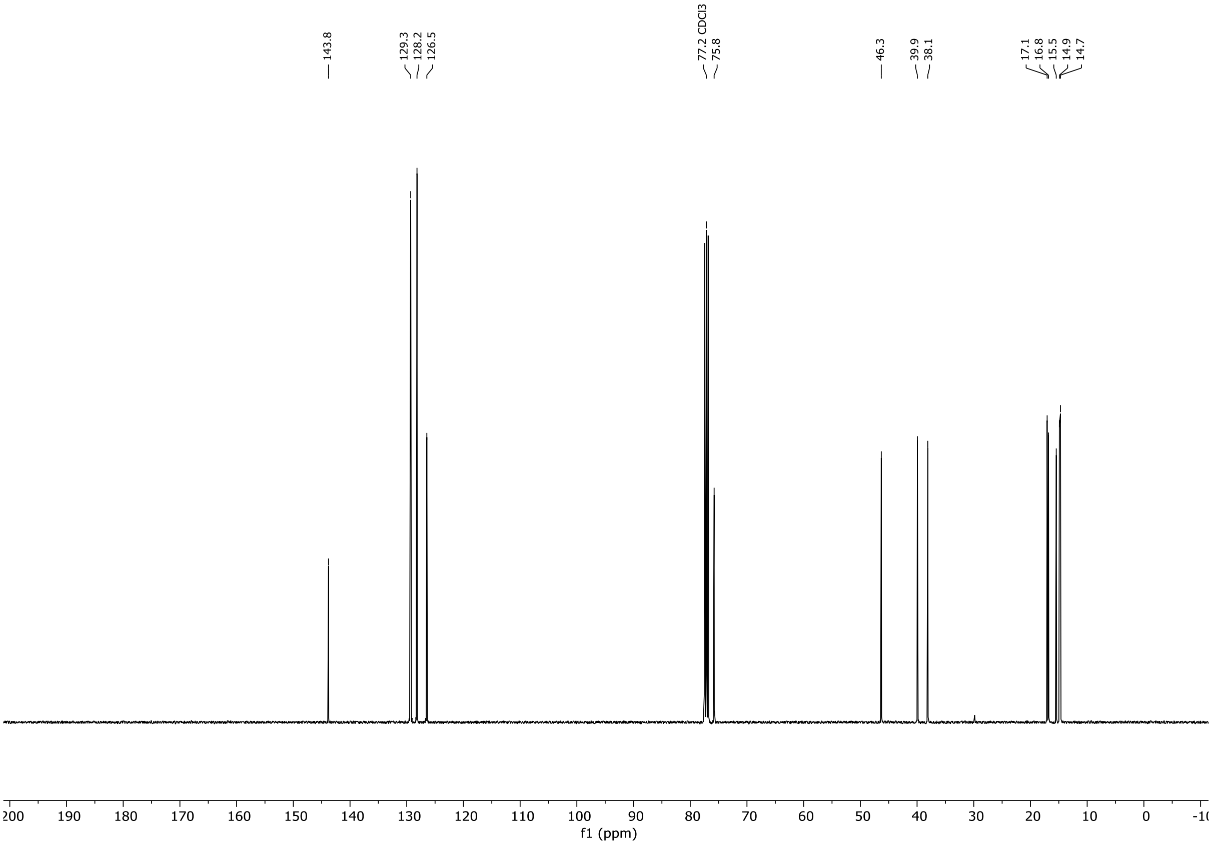

1,1-dicyclopropyl-2-phenylpropan-1-ol (10ac)

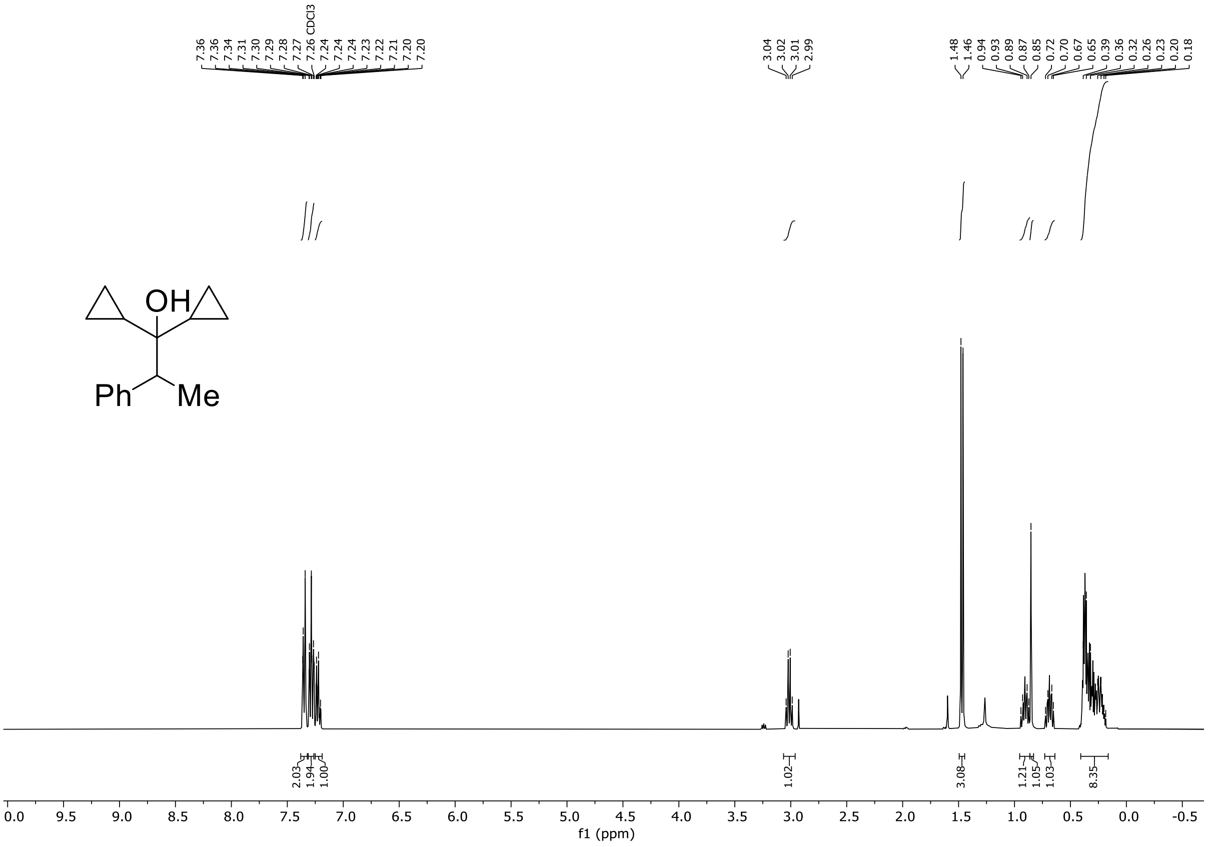

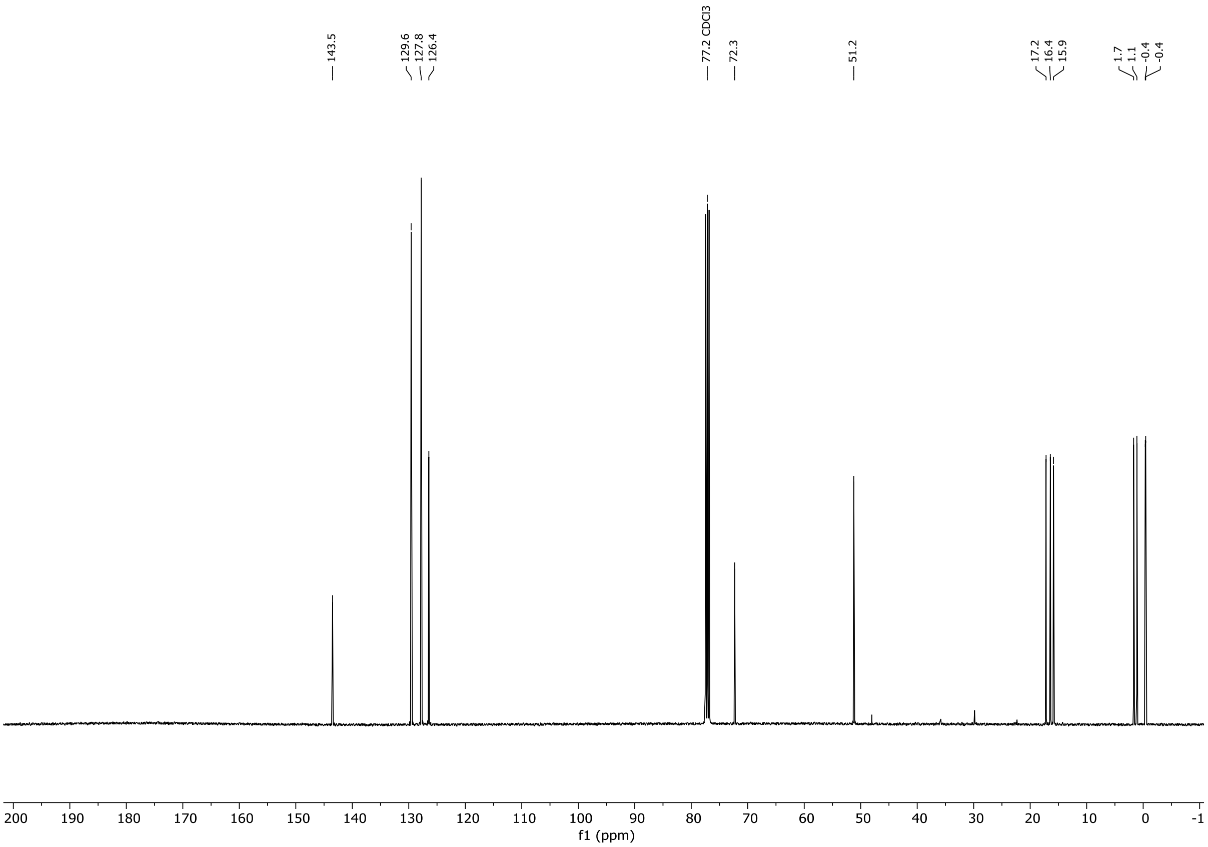

trans-(1-phenylethyl)cyclohexan-1-ol (10ad) mixture of diastereoisomeres

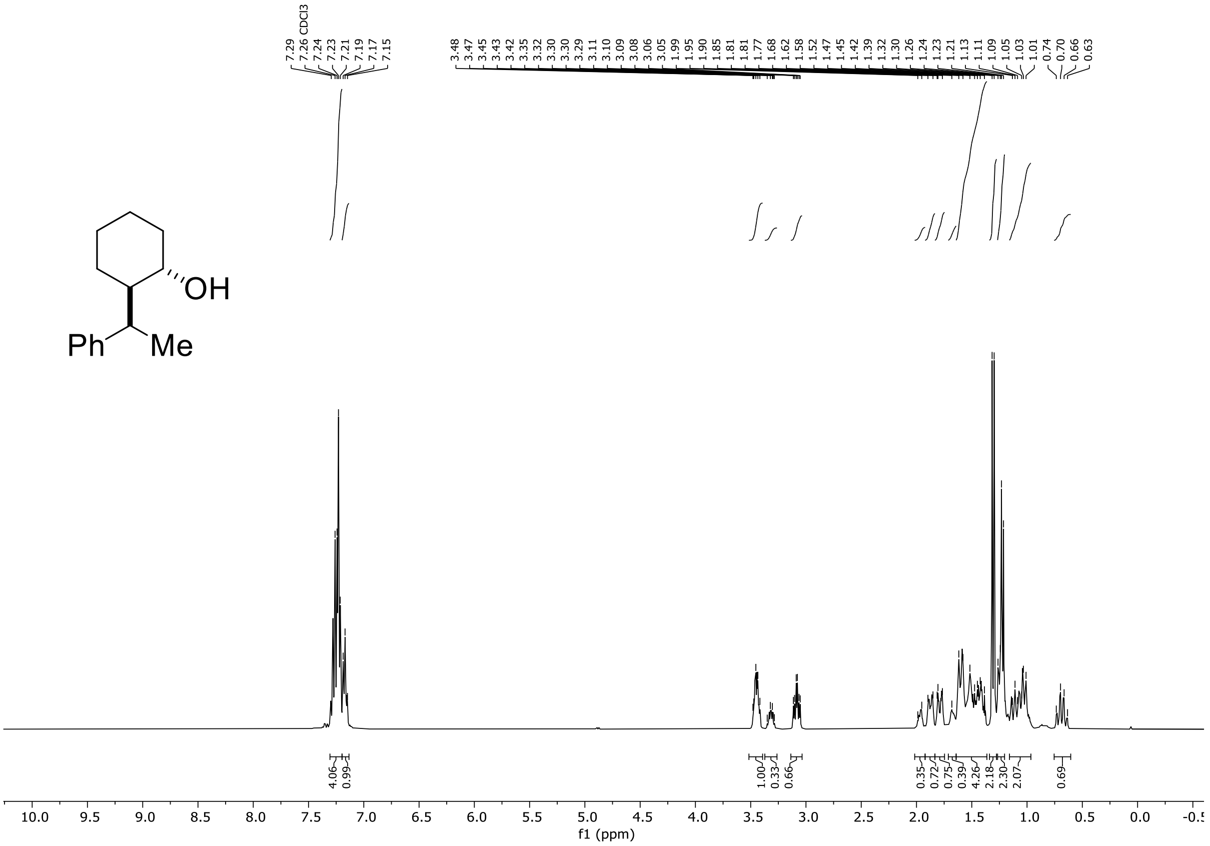

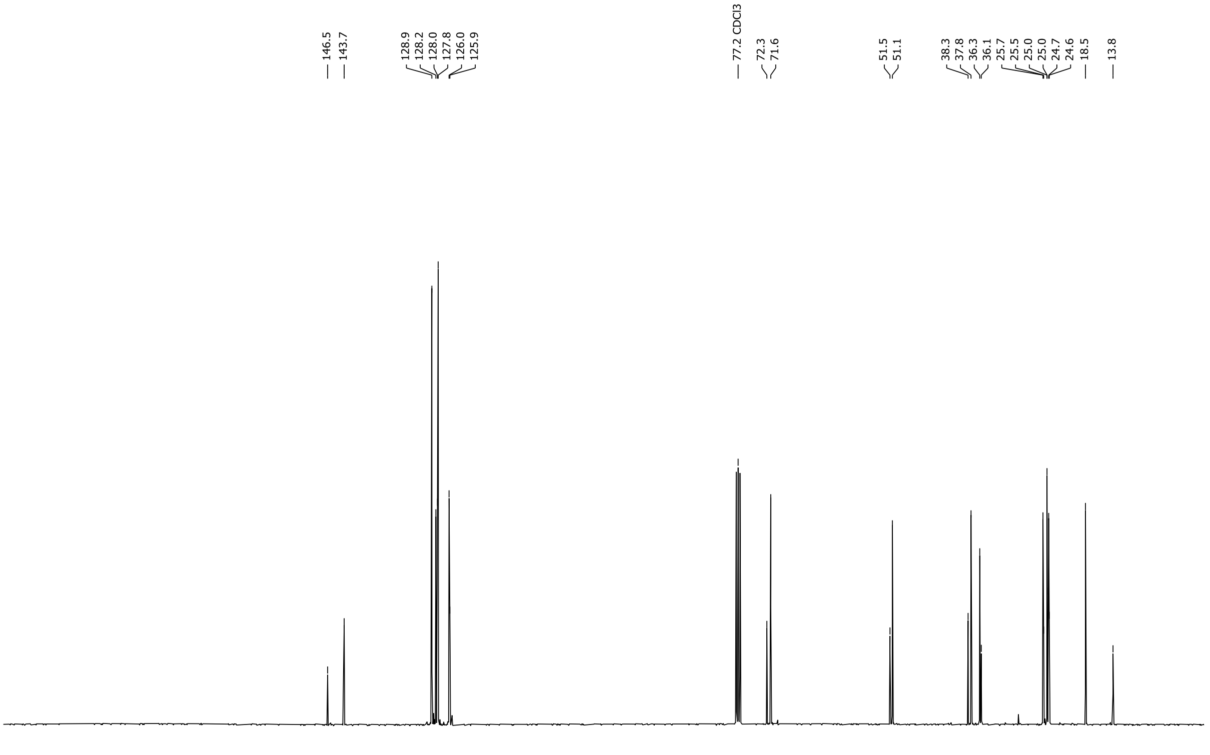

(2*S*)-1-(benzyloxy)-4-phenylpentan-2-ol (10ae) mixture of diastereoisomers

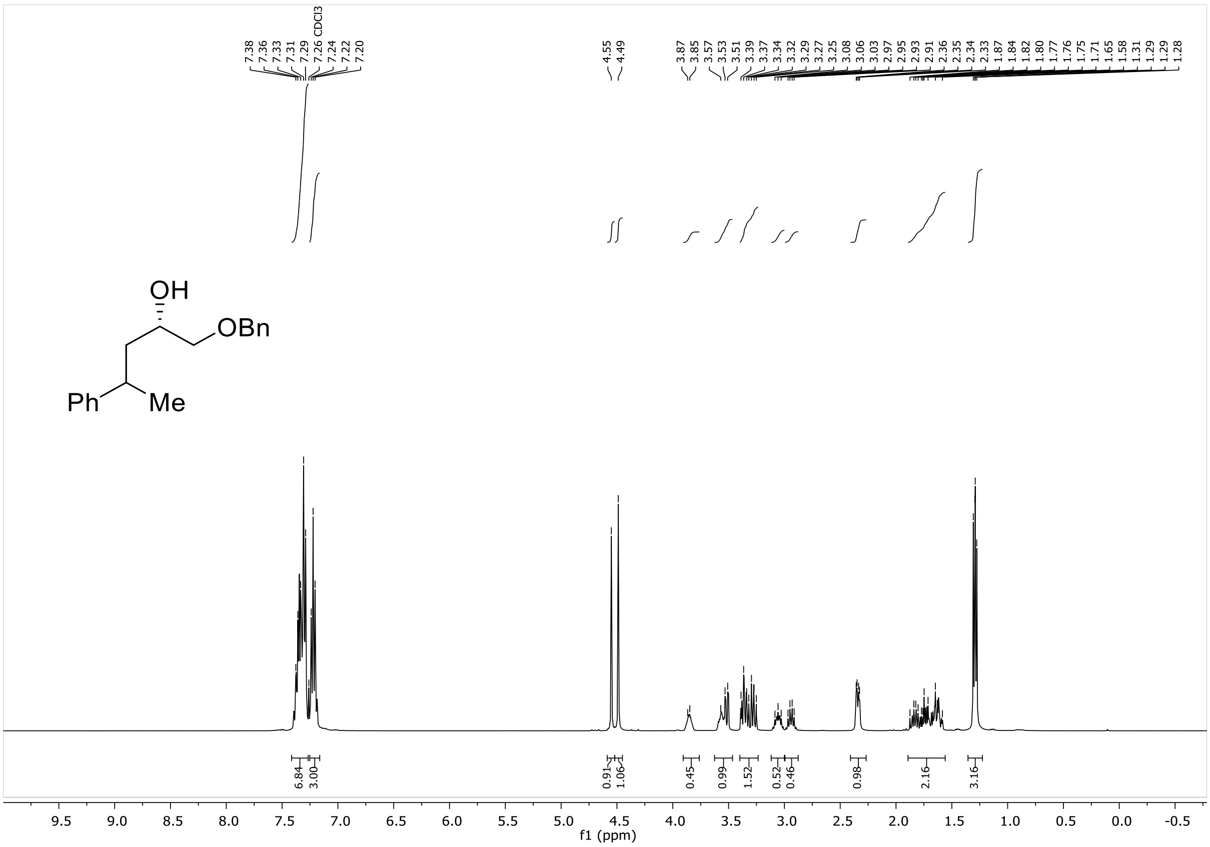

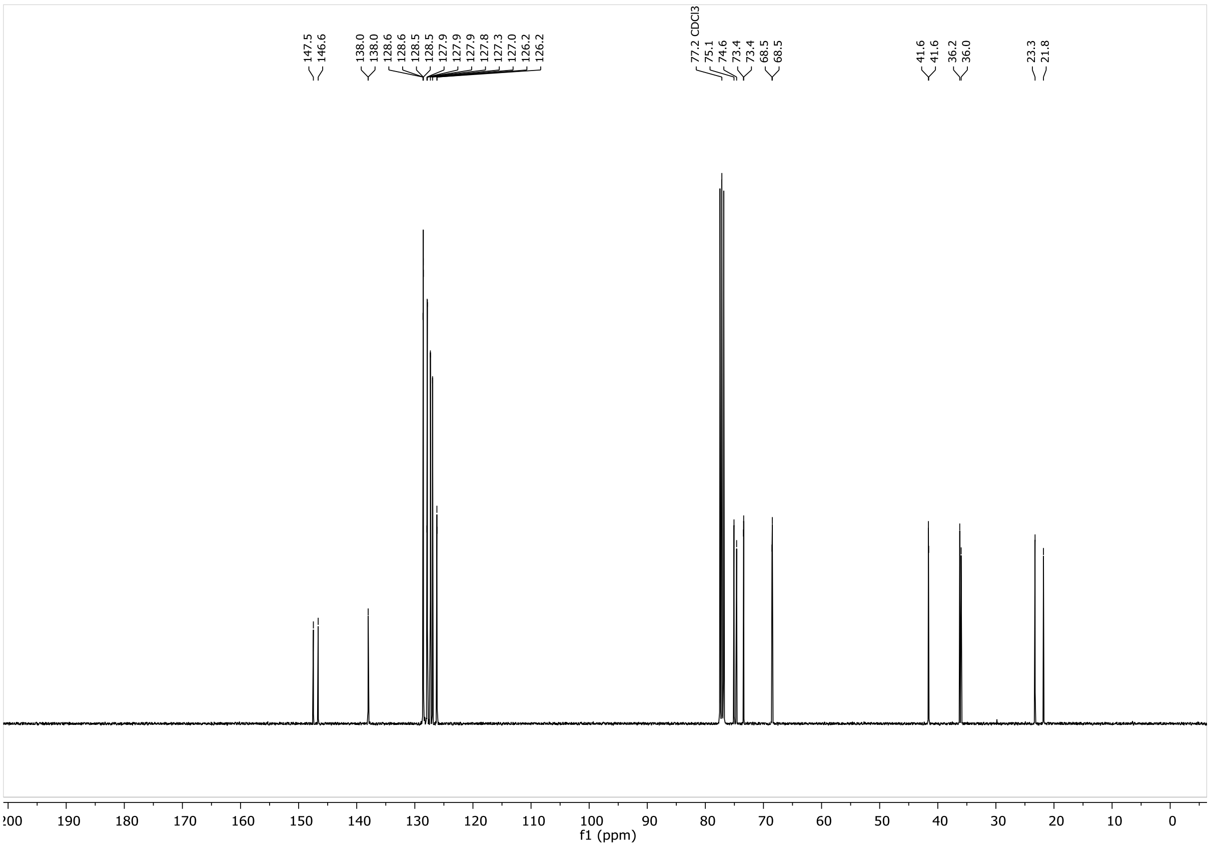

dicyclopropyl(1,2,3,4-tetrahydronaphthalen-1-yl)methanol (10af)

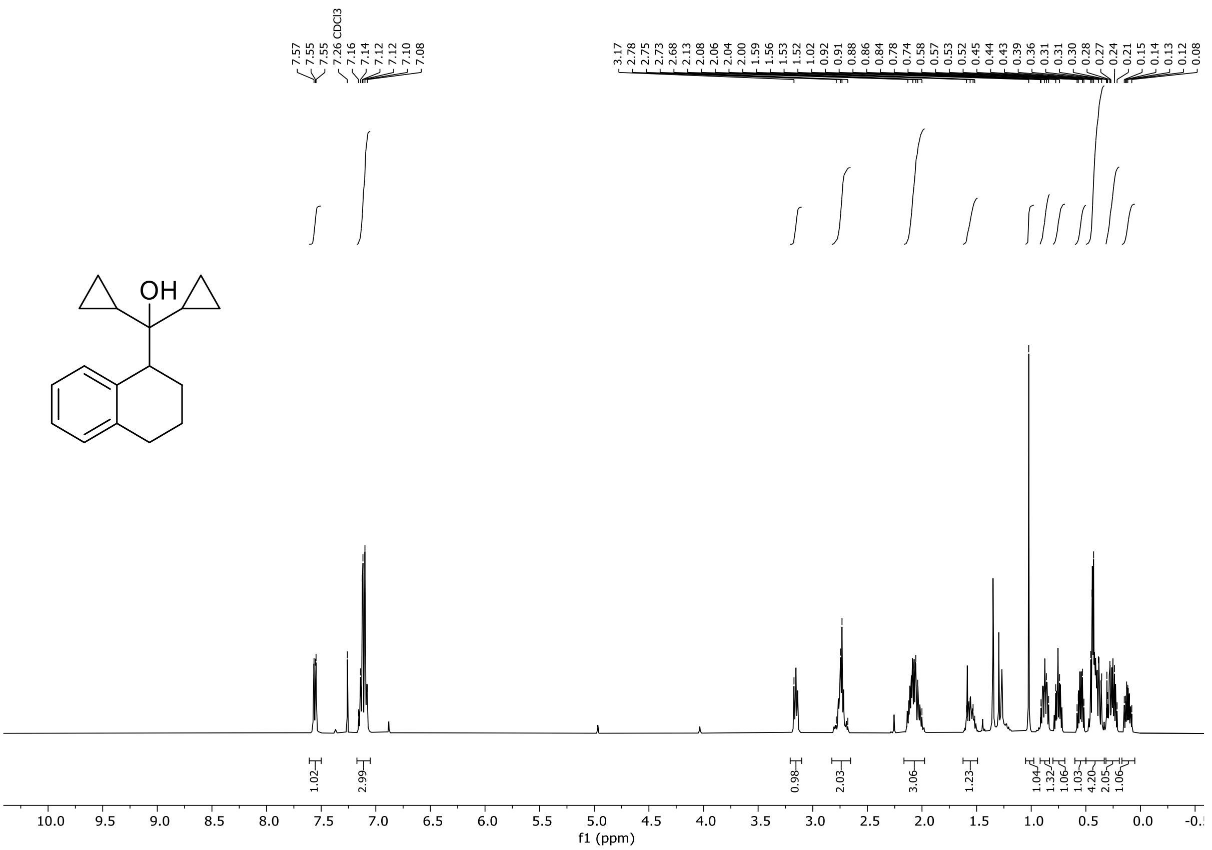

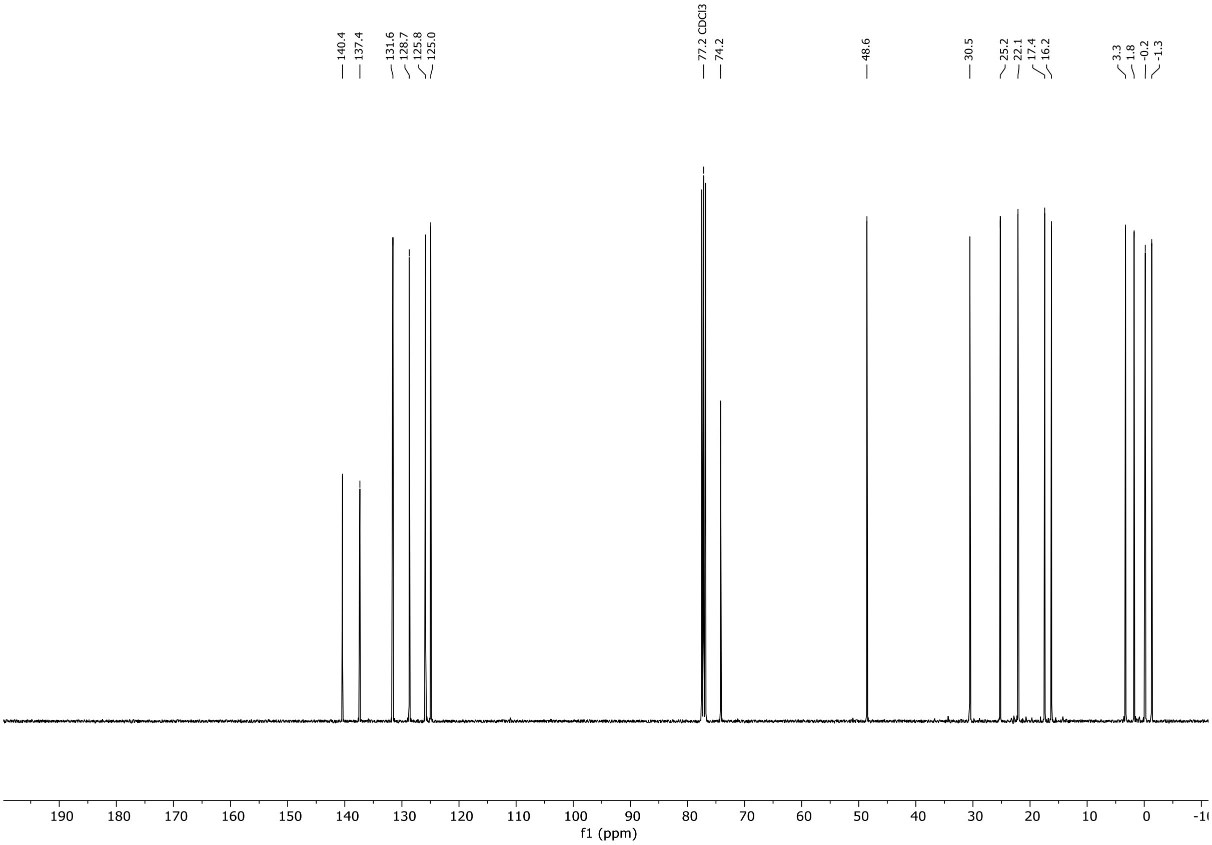

4-(1,2,3,4-tetrahydronaphthalen-1-yl)heptan-4-ol (10ag)

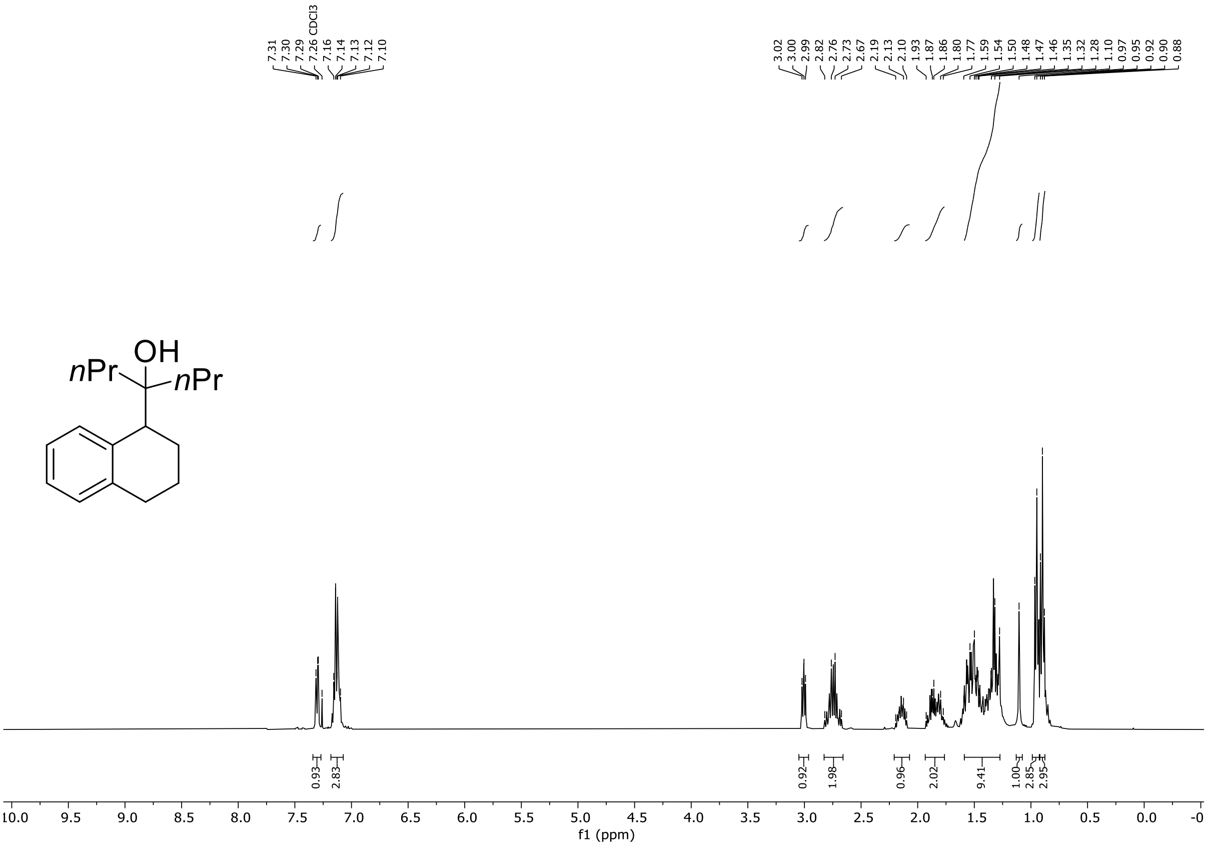

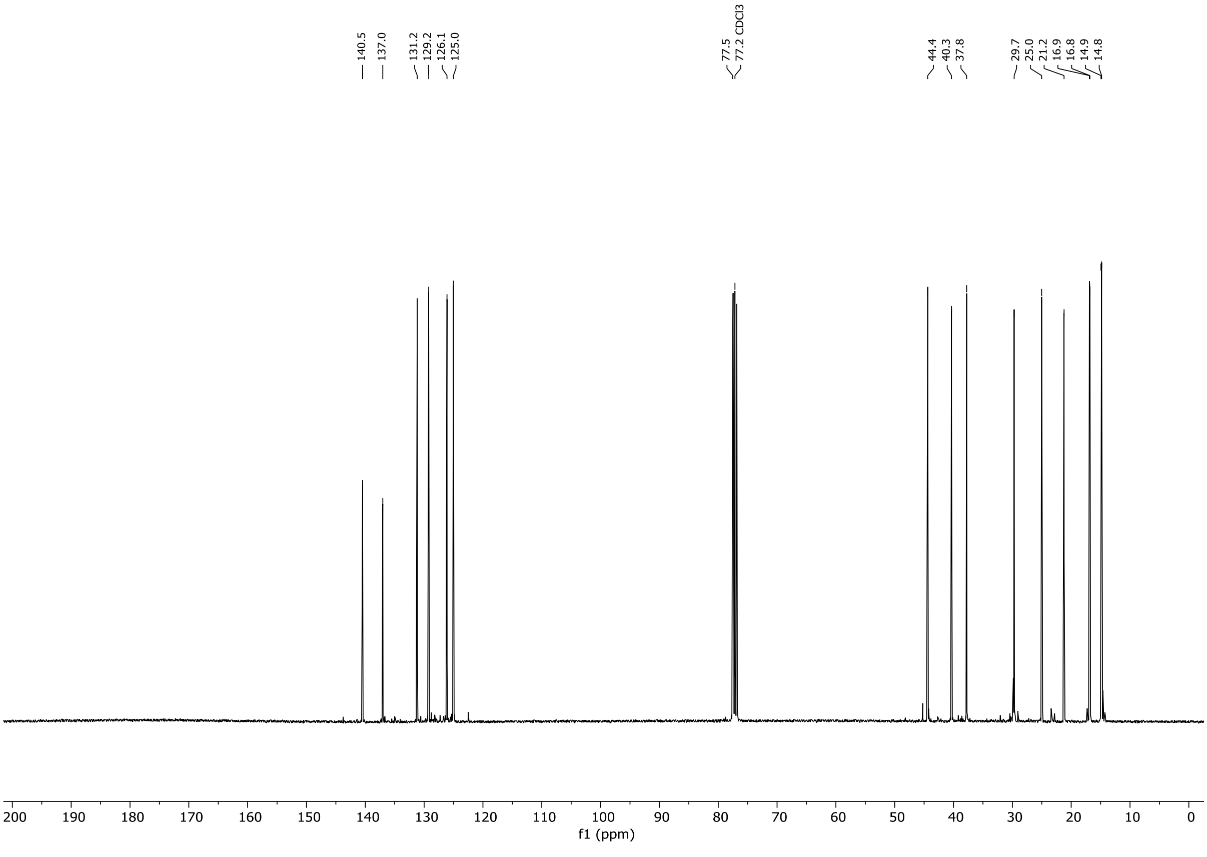

trans-2-(1,2,3,4-tetrahydronaphthalen-1-yl)cyclohexan-1-ol (10ah)

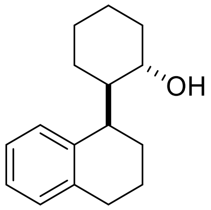

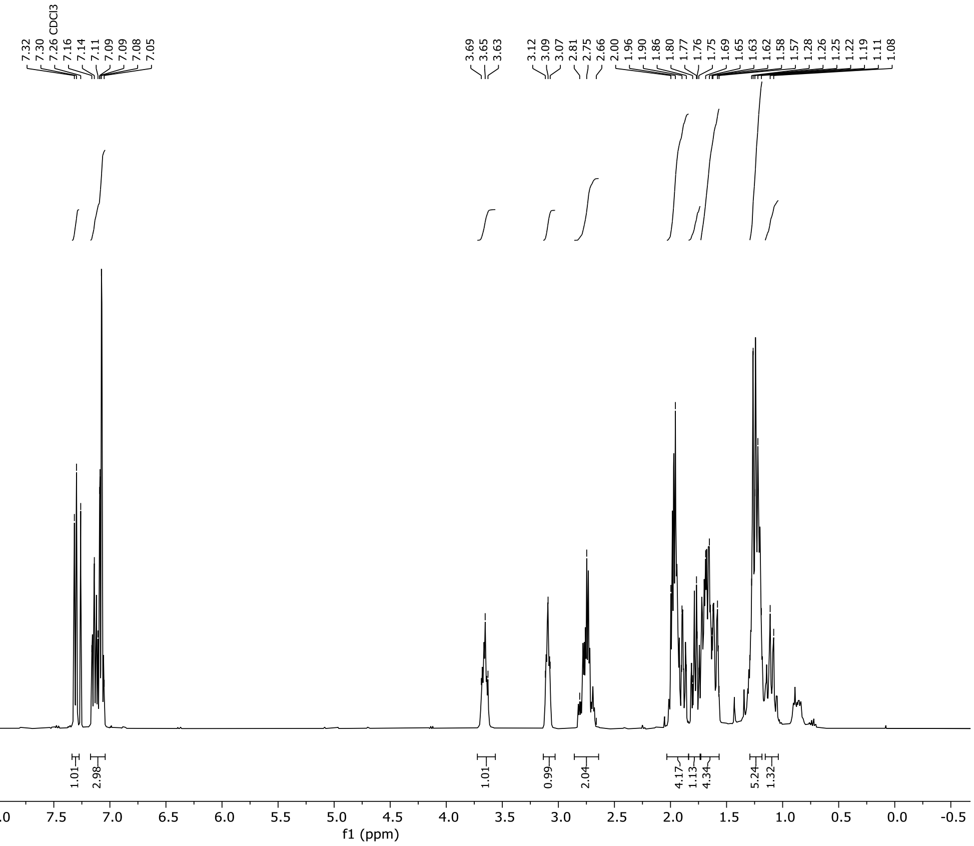

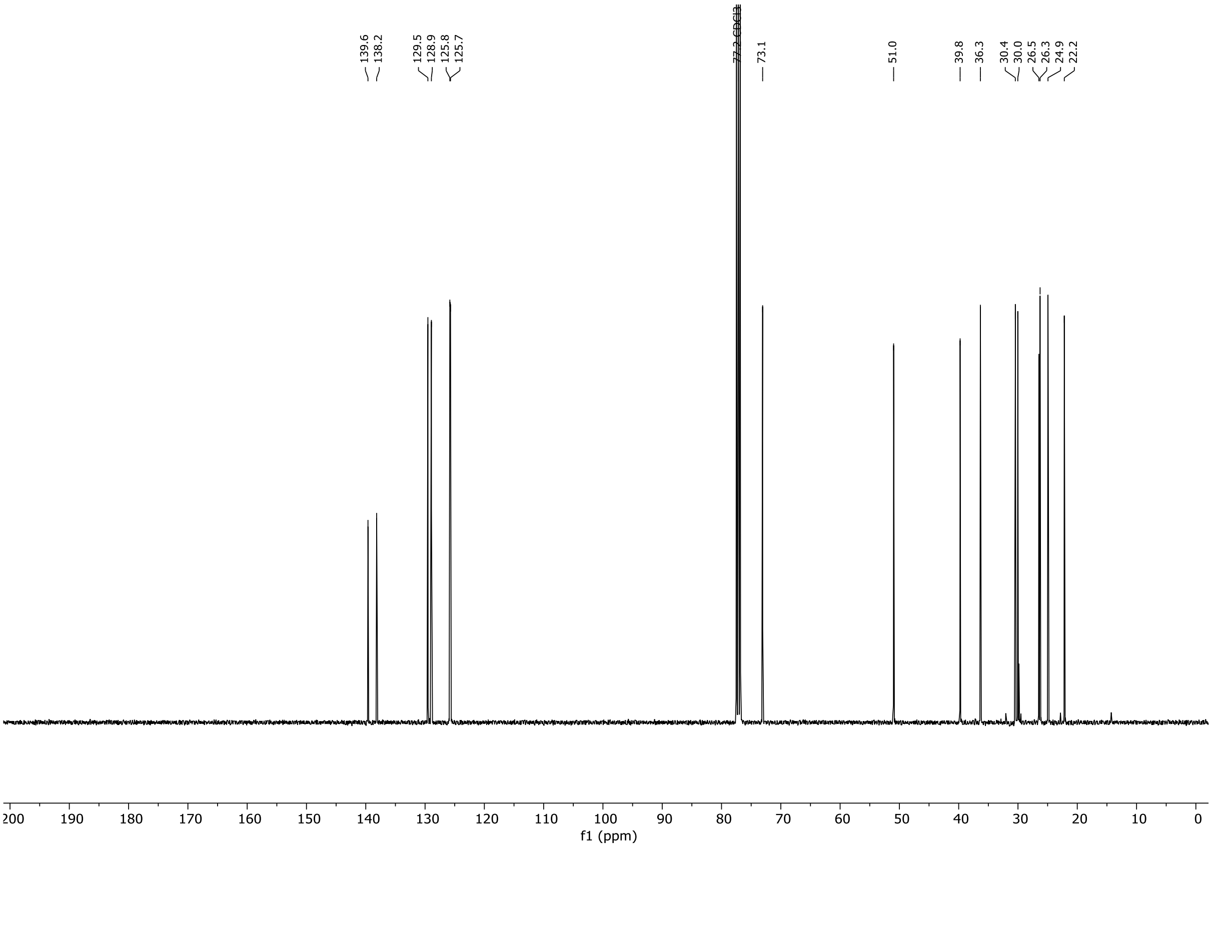

trans-2-(2-methoxy-3-methylbenzyl)cyclohexan-1-ol (10ai)

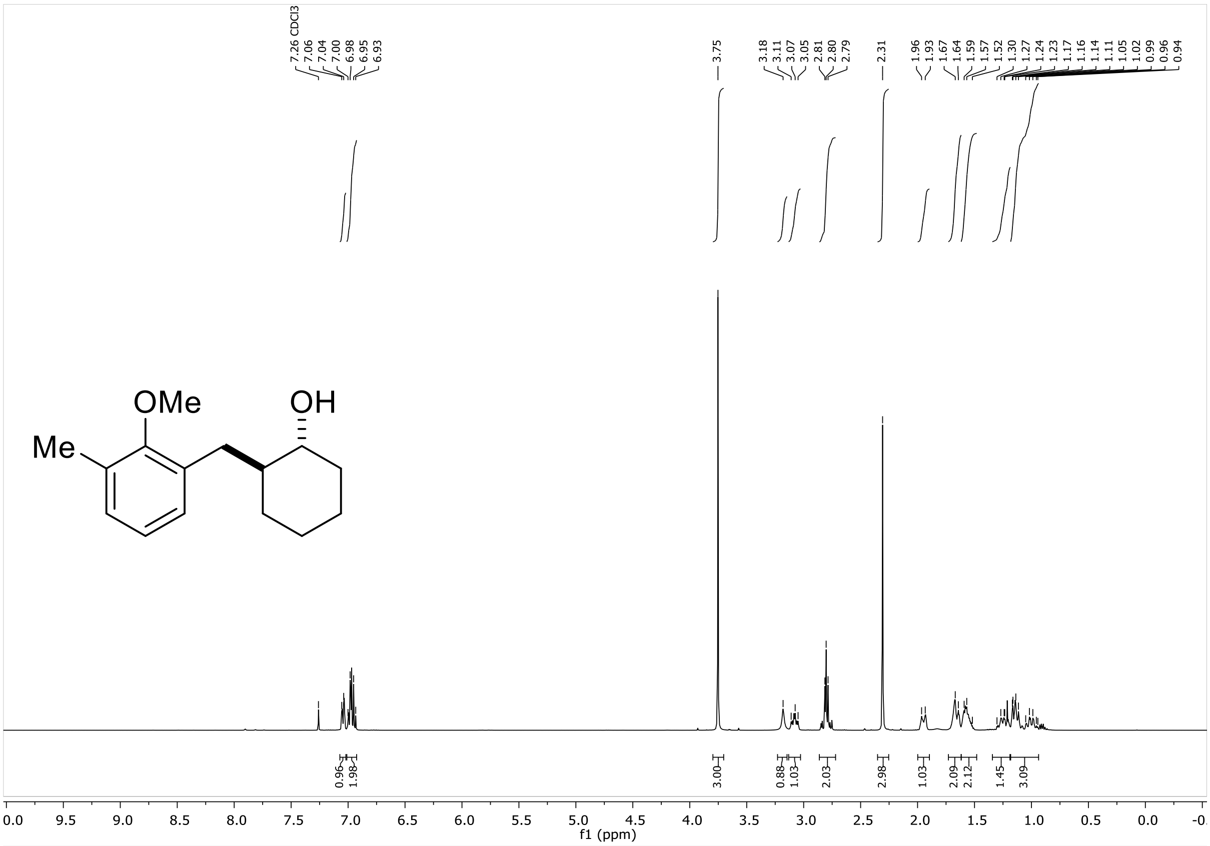

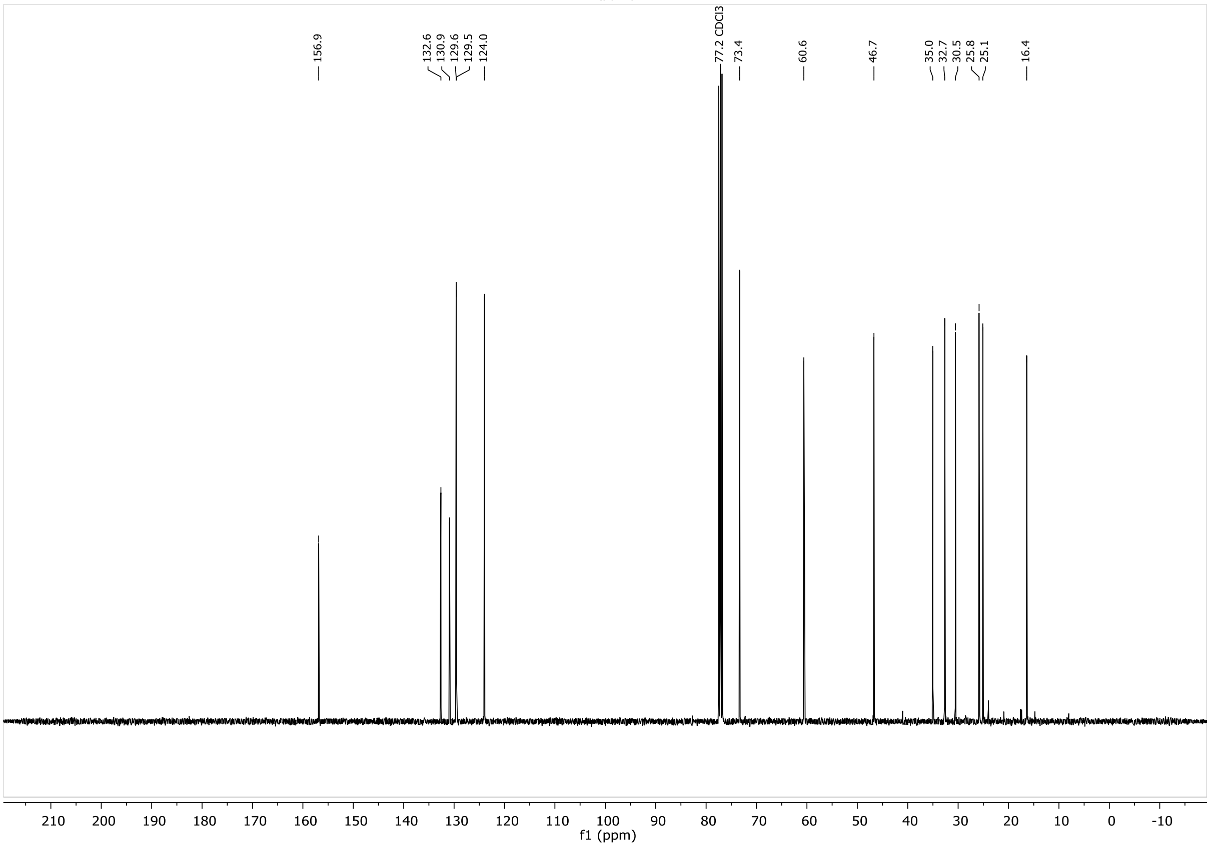

2-(2-fluoro-3,5-dimethylbenzyl)adamantan-2-o (10aj)

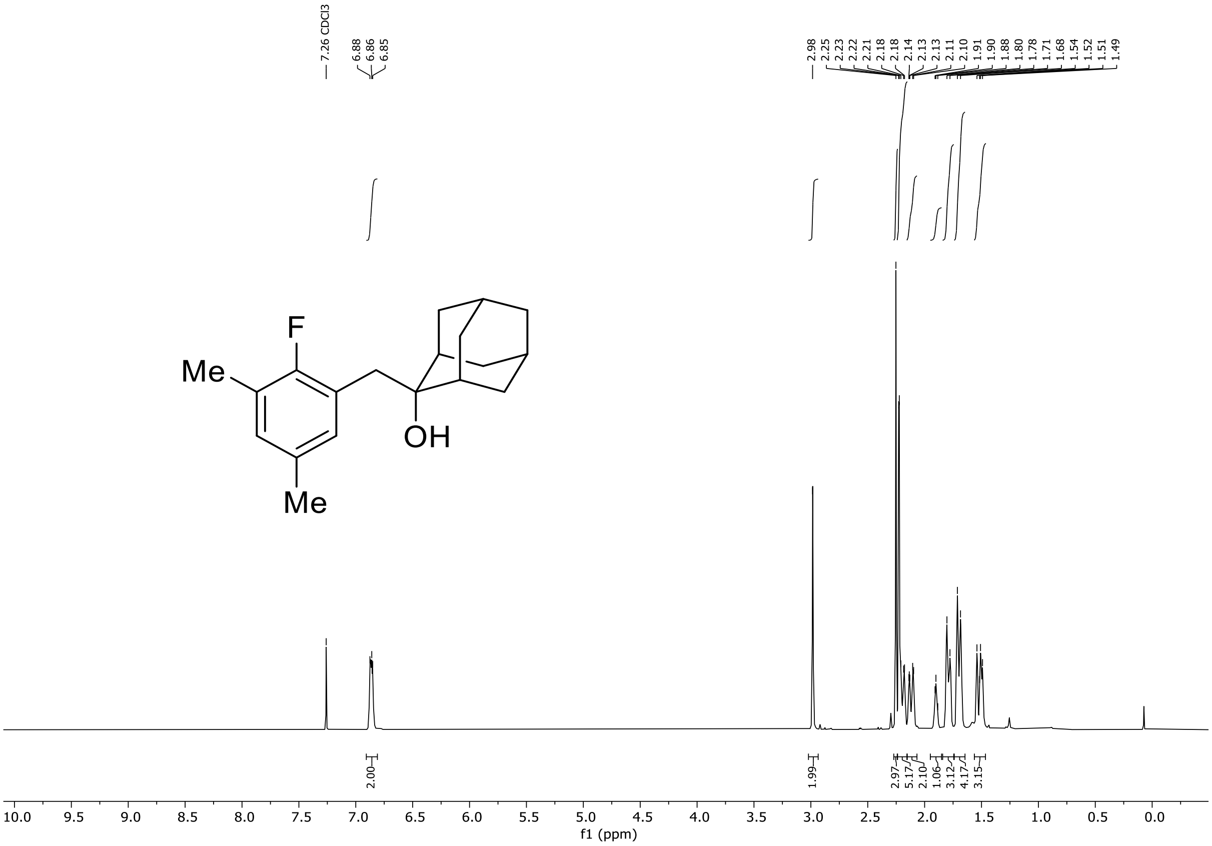

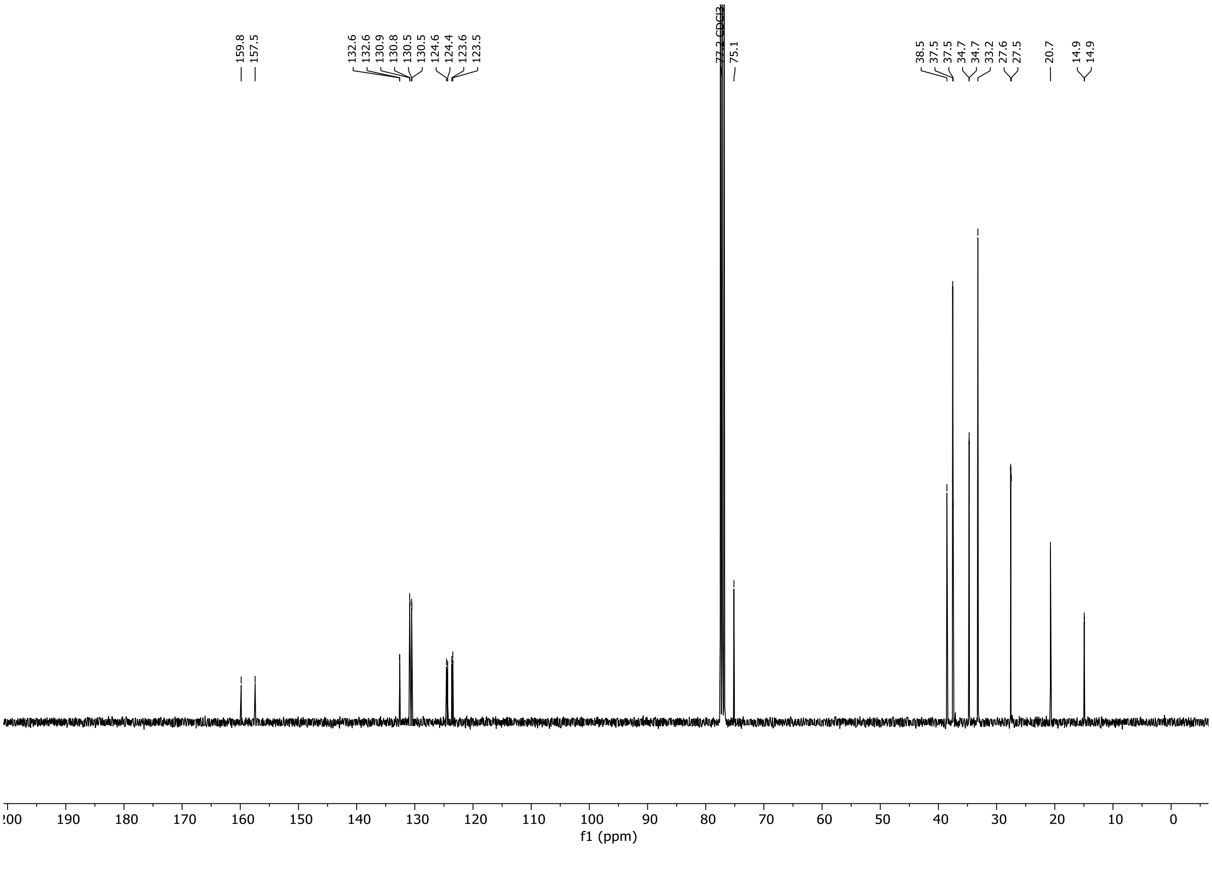

***trans*-2-((2-hydroxycyclohexyl)methyl)-N,N-diisopropylbenzamide (10ak) mixture of diastereoisomers**

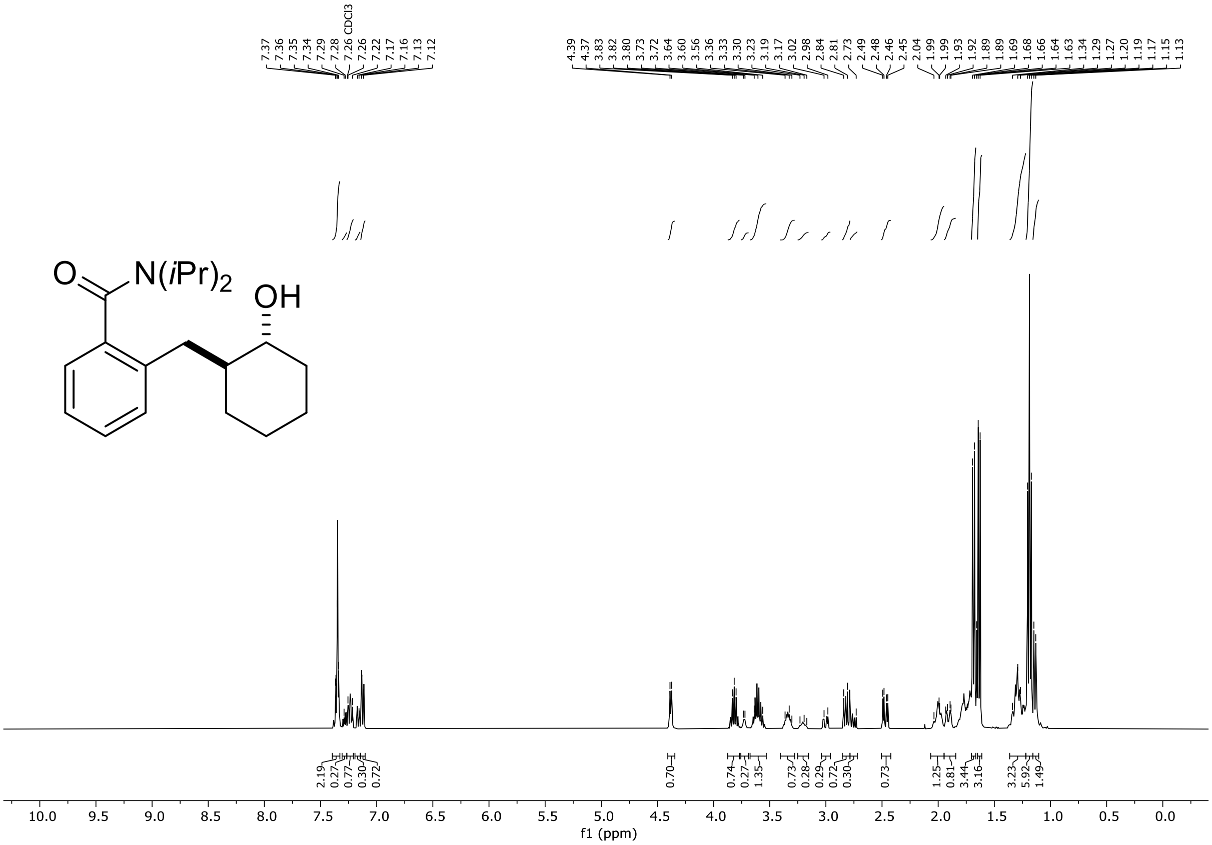

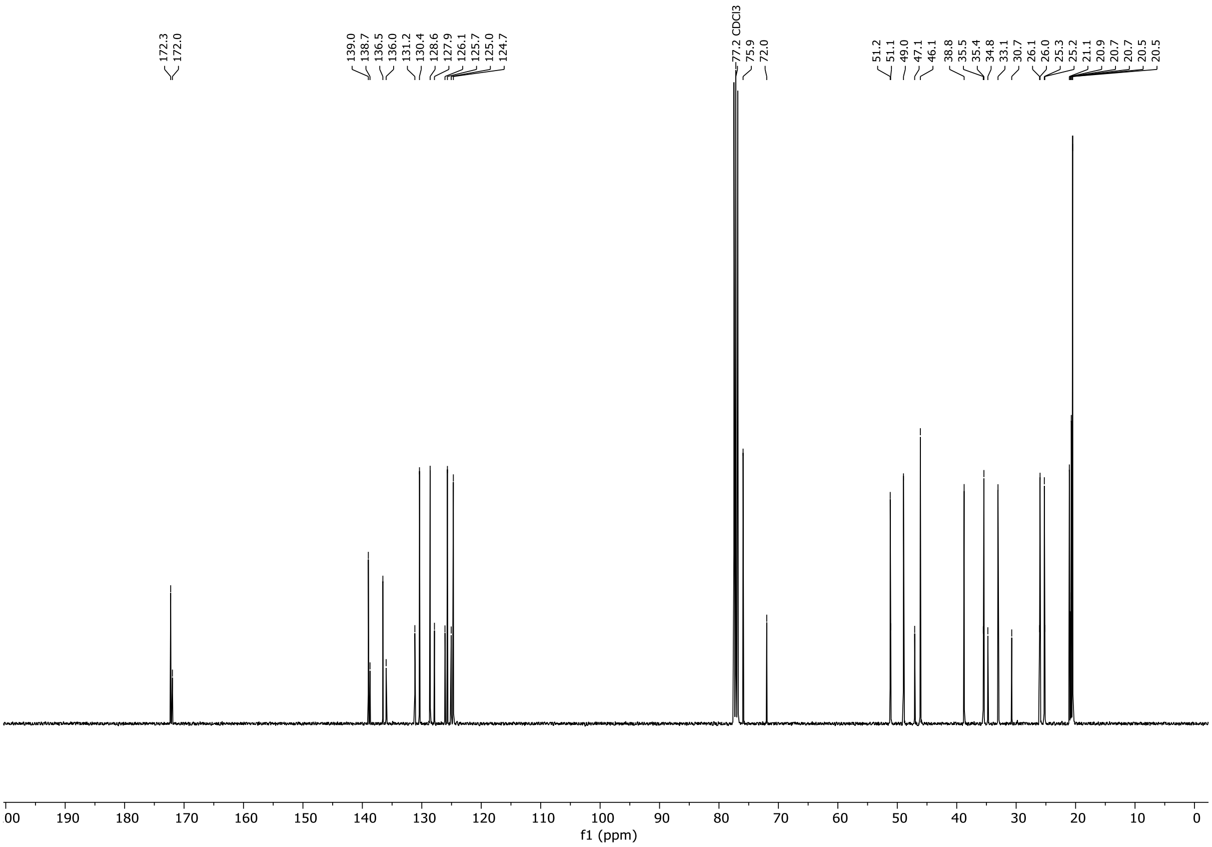

2-((2-hydroxyadamantan-2-yl)methyl)-N,N-diisopropylbenzamide (10al)

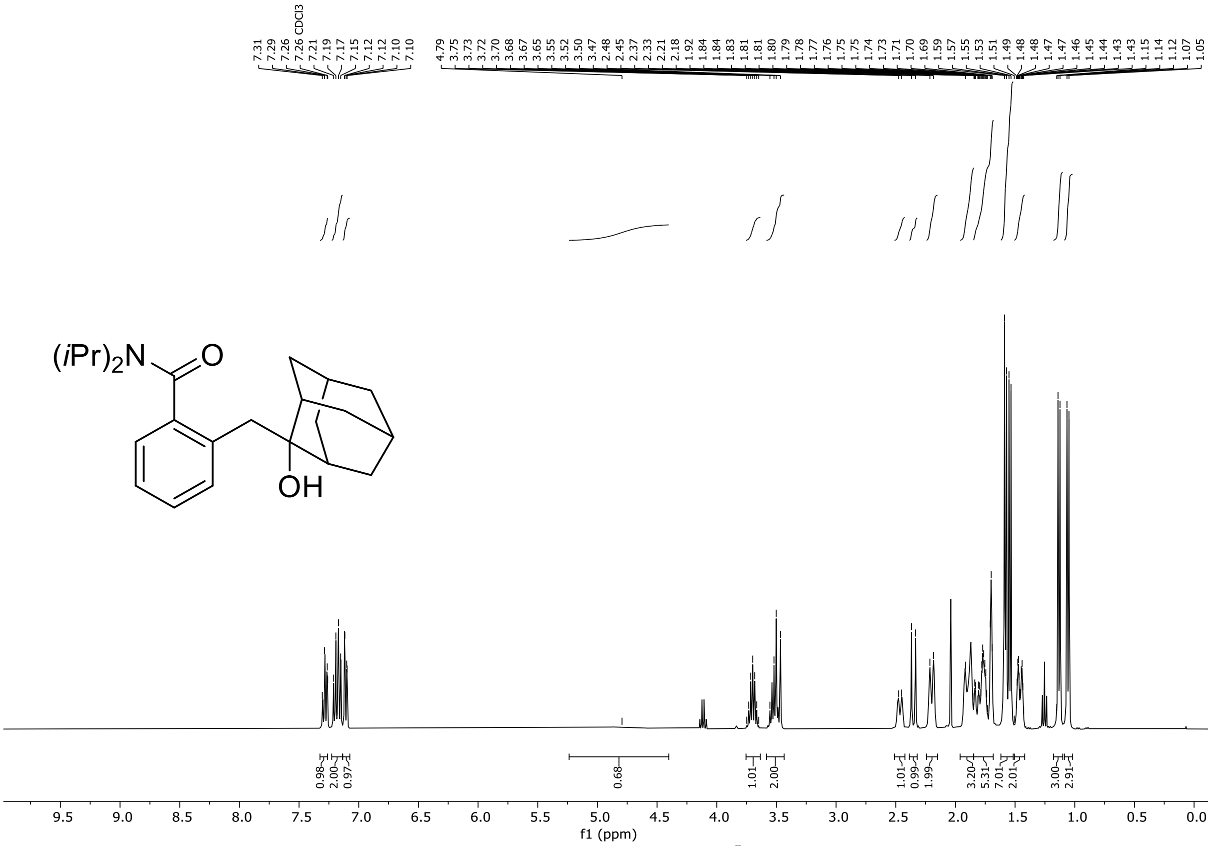

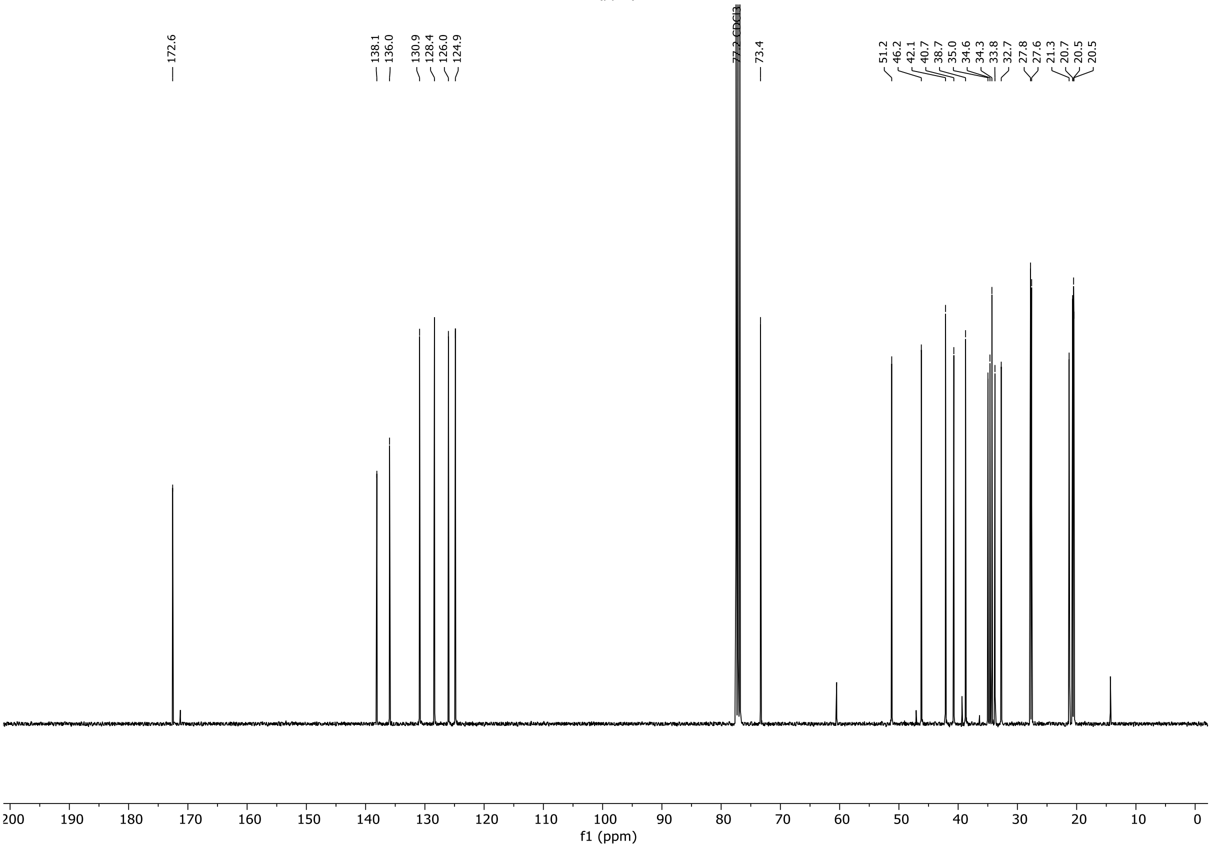

***trans*-2-(phenyl(phenylthio)methyl)cyclohexan-1-ol (10am) mixture of diastereoisomeres**

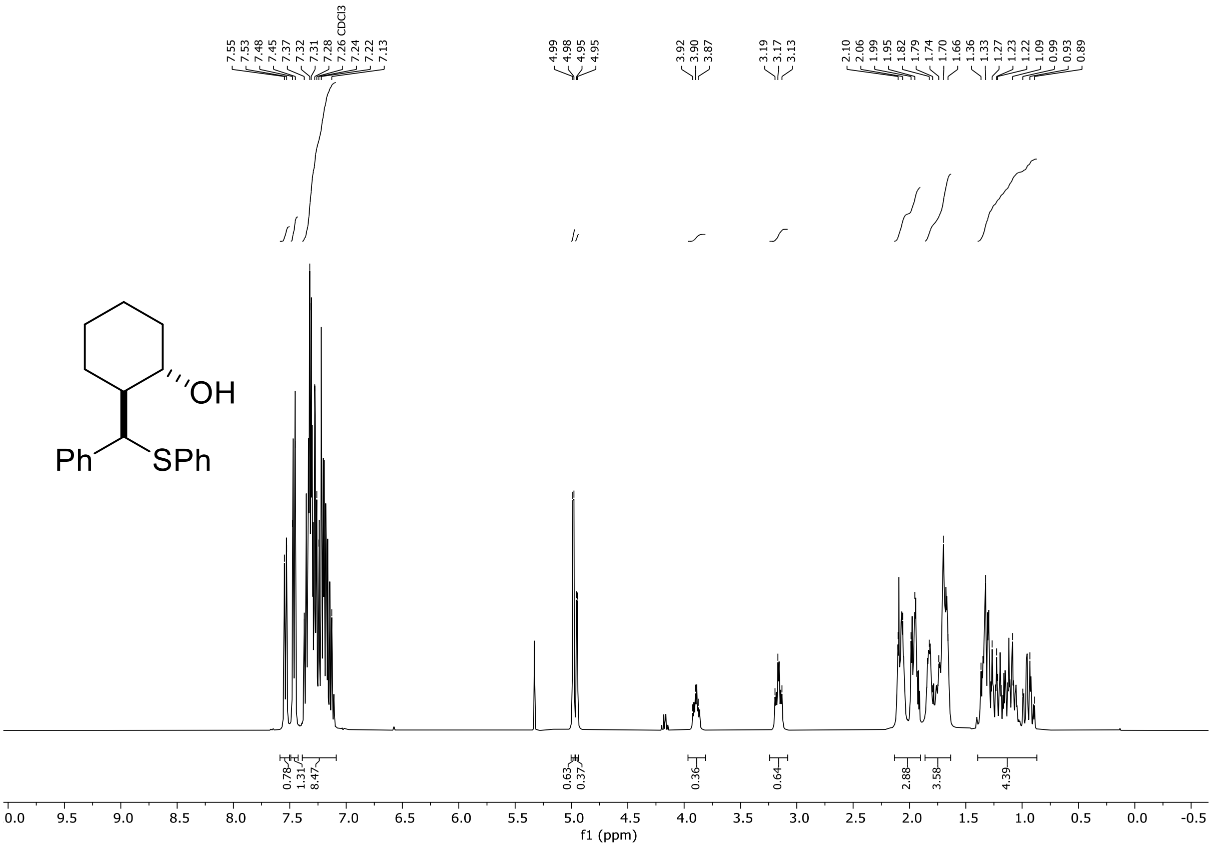

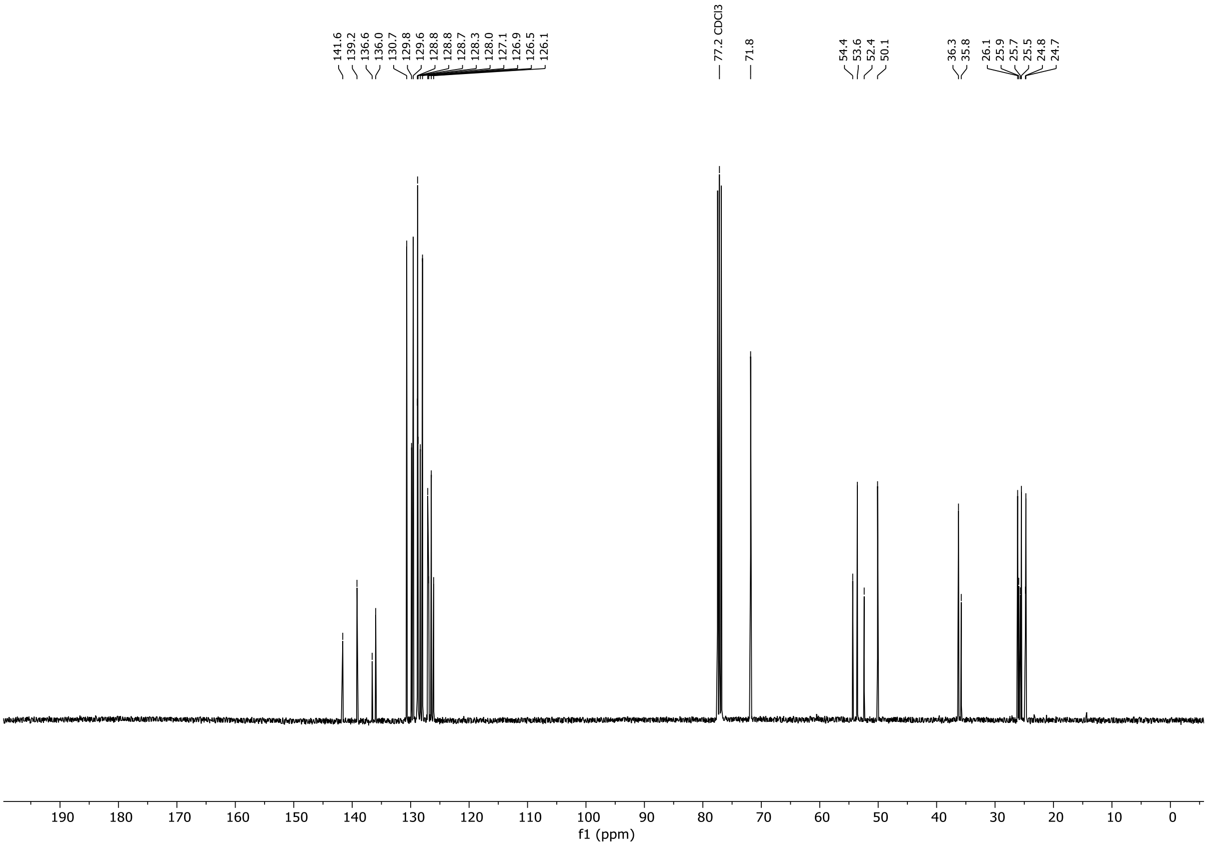

4-phenyl-4-(phenylthio)butan-1-ol (10an)

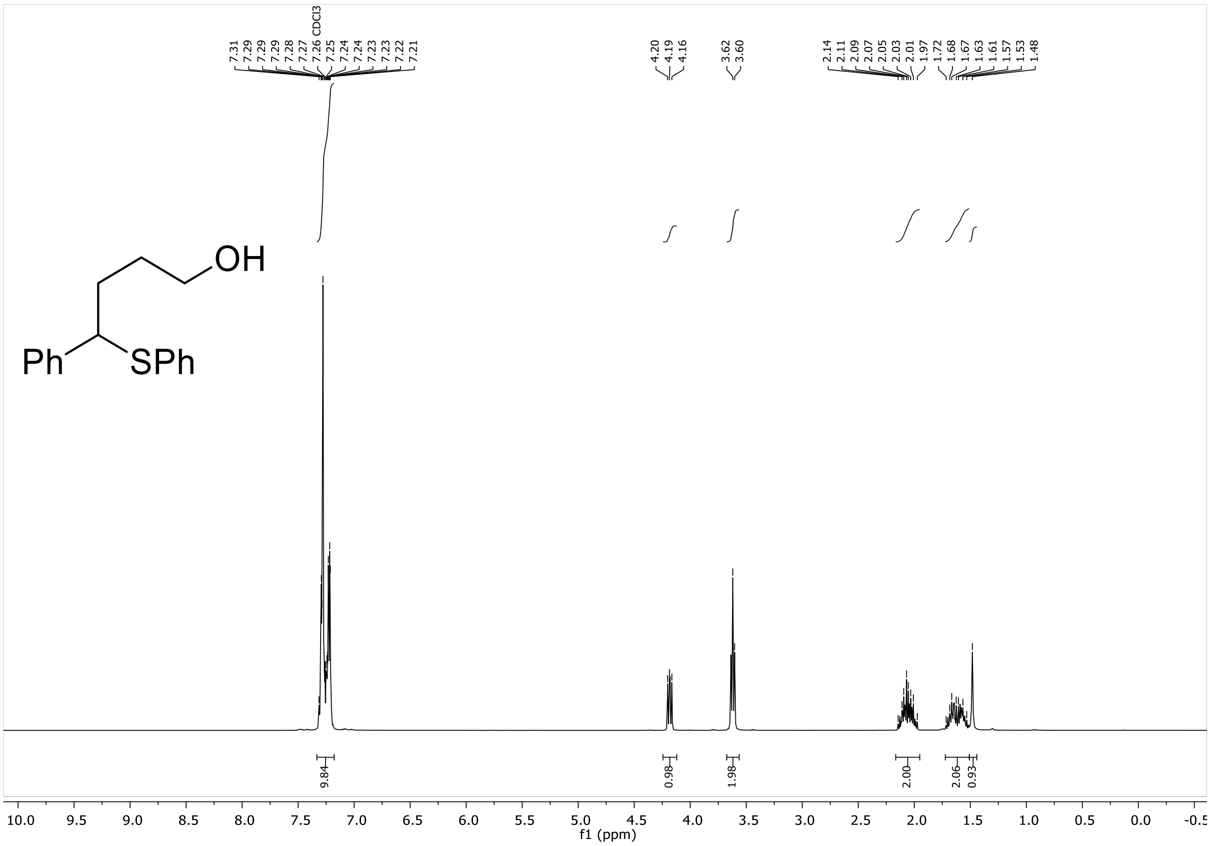

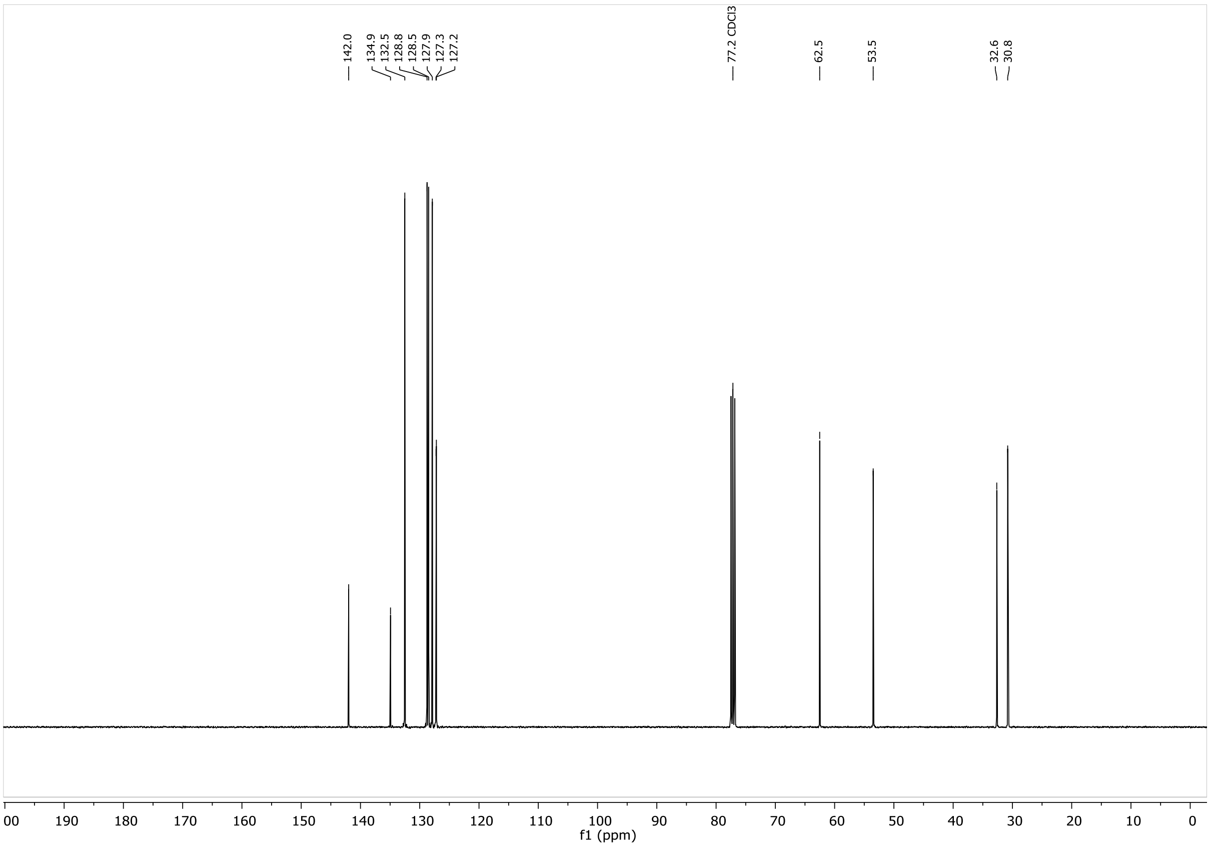

***trans*-2-((2-hydroxycyclohexyl)-2-phenylacetonitrile (10ao) mixture of diastereoisomeres**

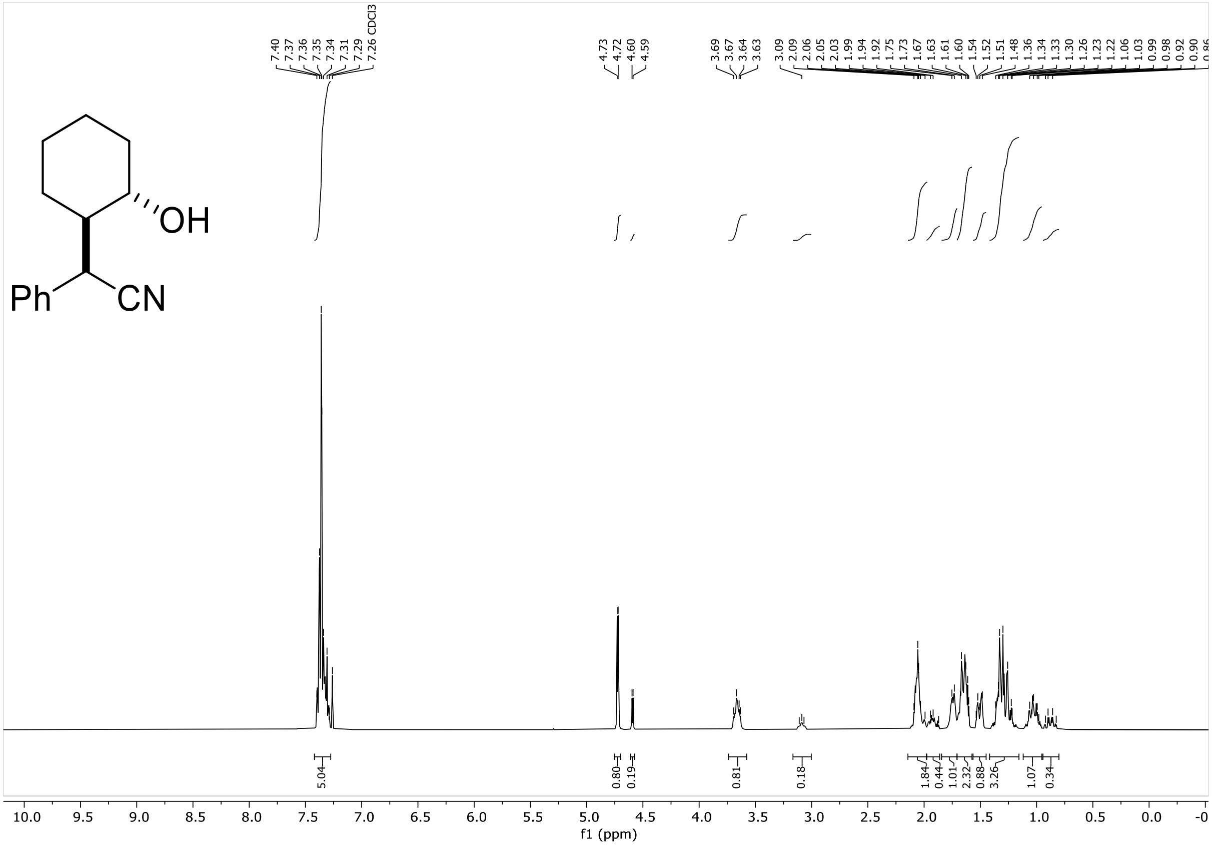

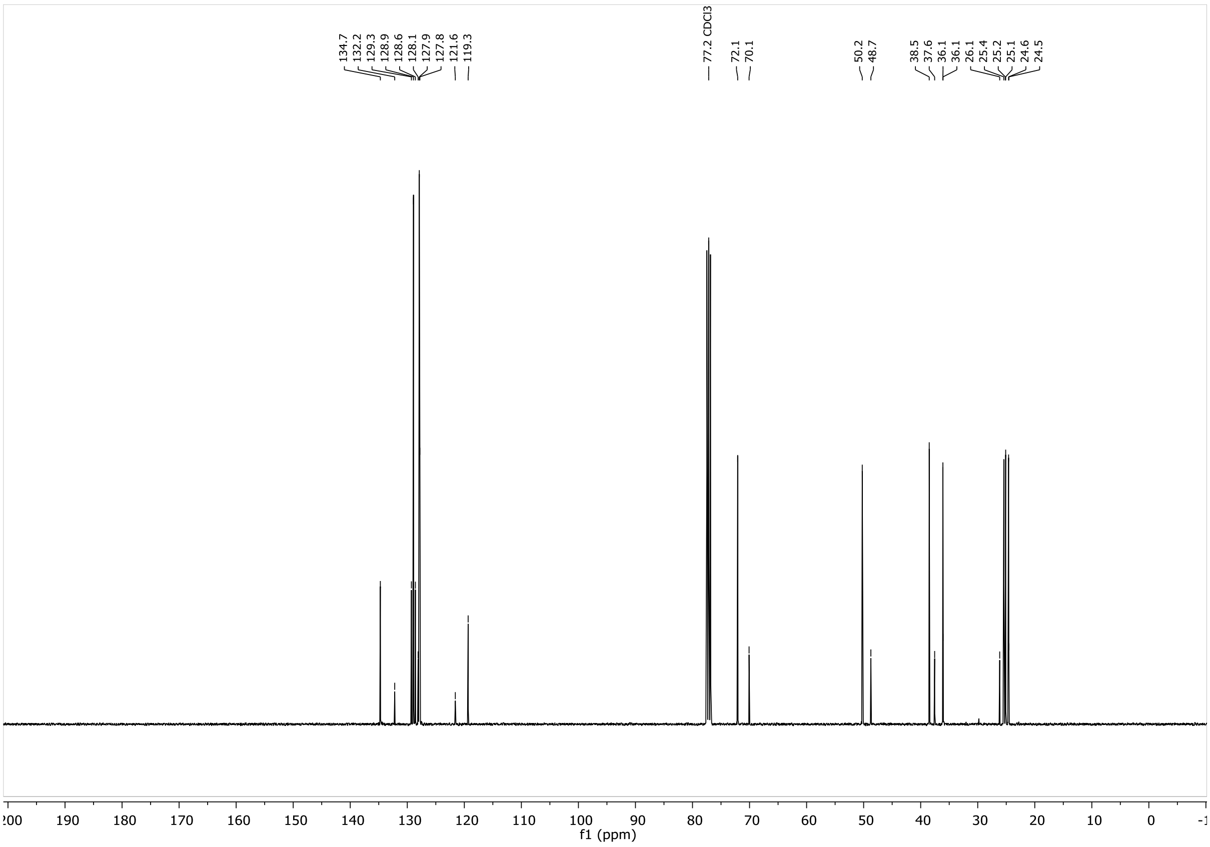

***trans*-2-benzhydrylcyclohexan-1-ol (10ap)**

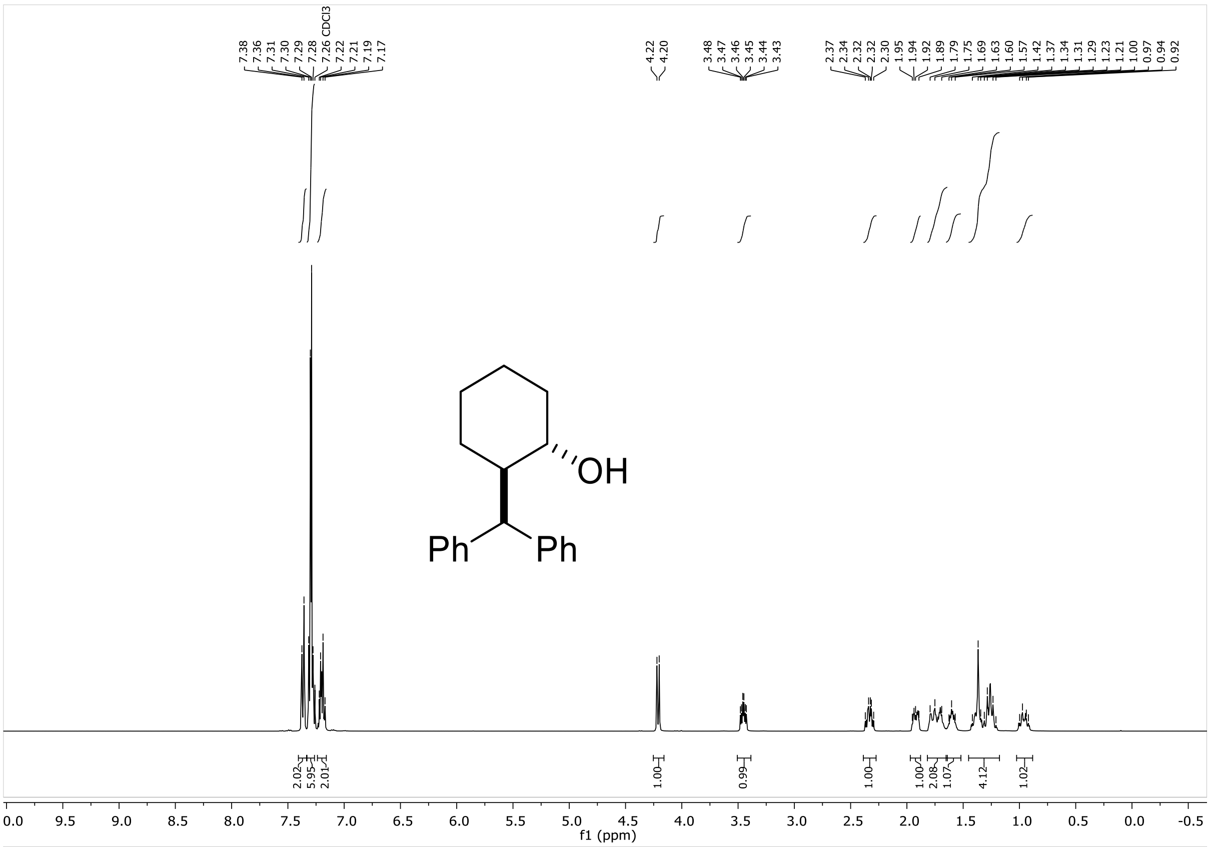

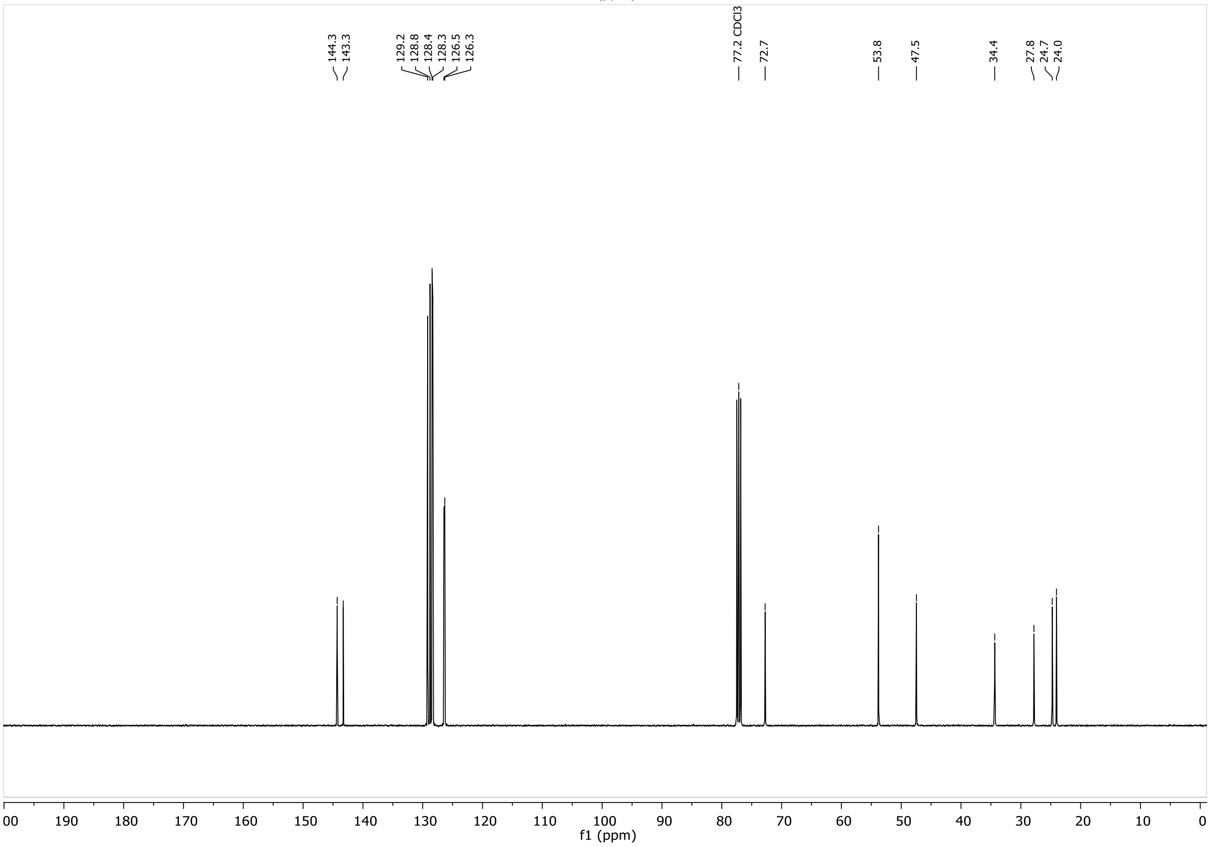

(*S*)-1-(benzyloxy)-4,4-diphenylbutan-2-ol (10aq)

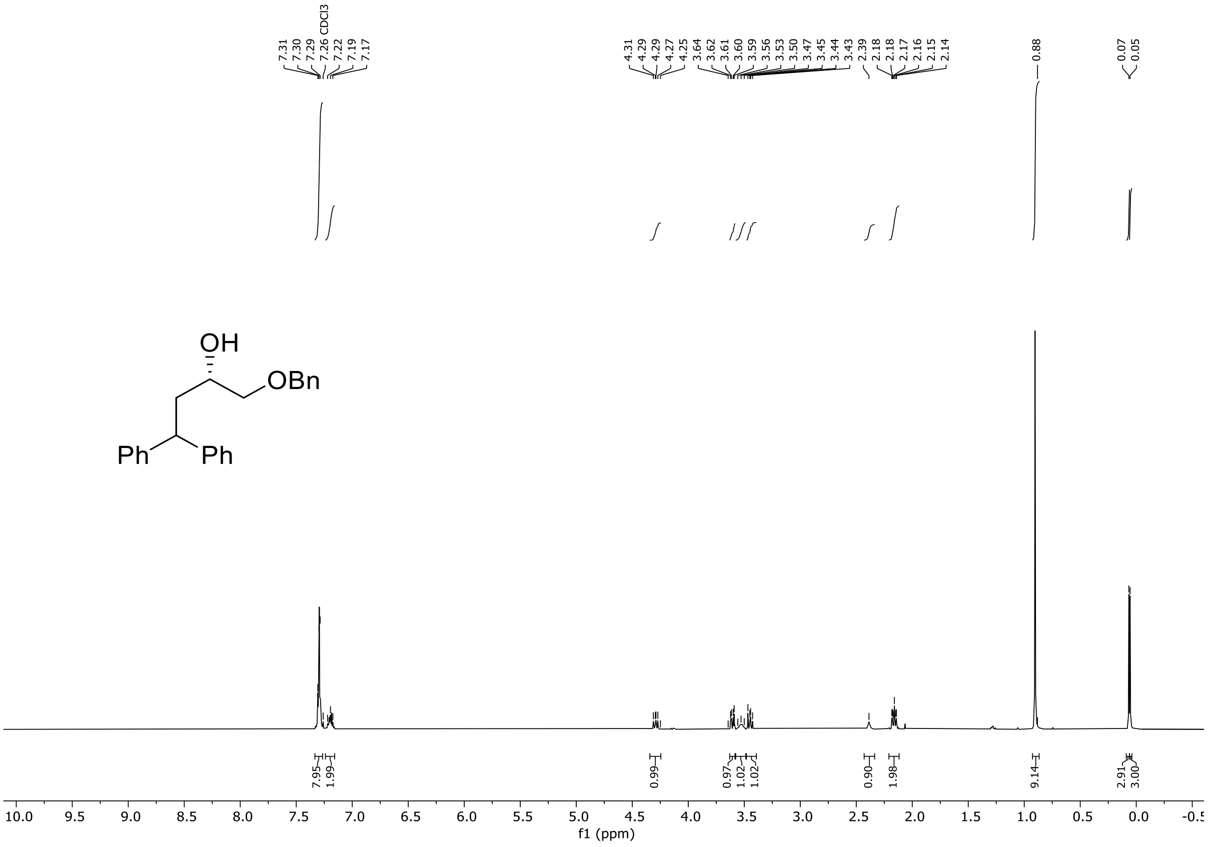

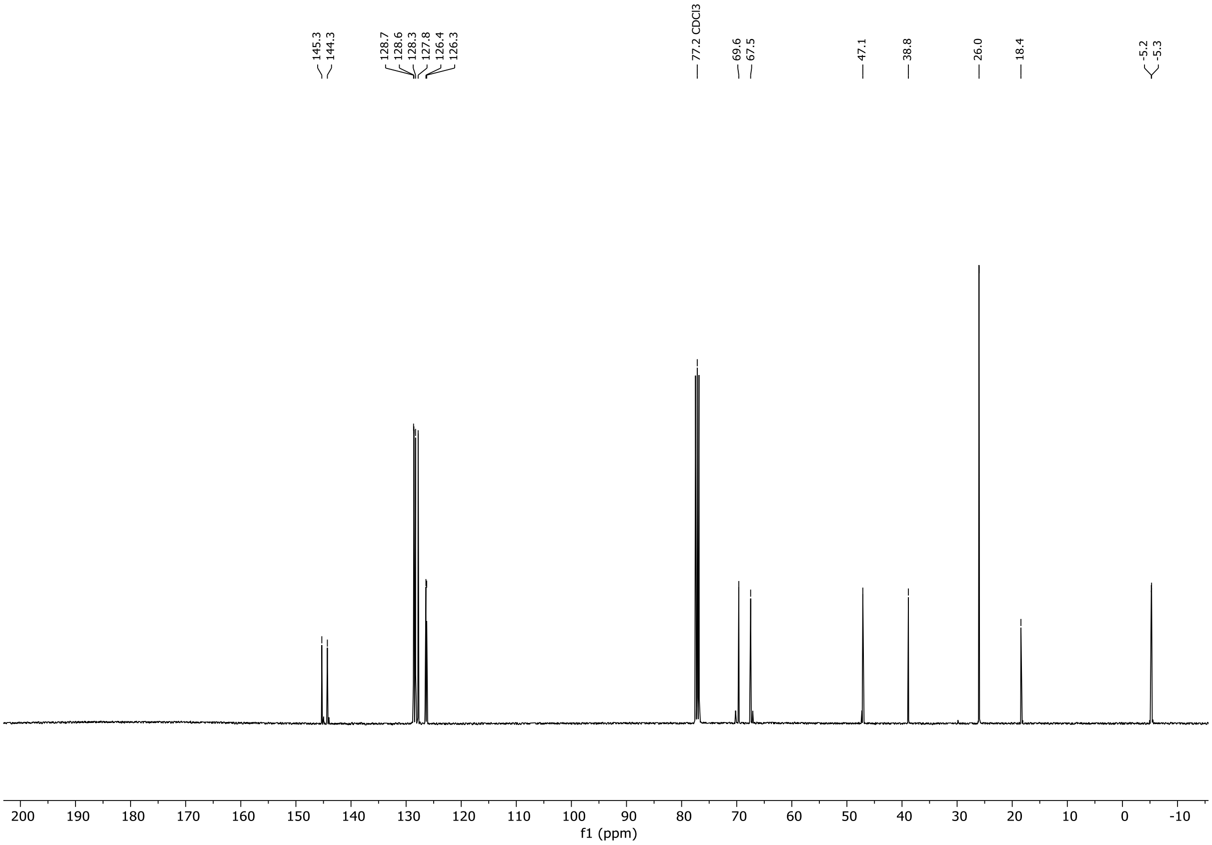

(*S*)-1-((tert-butyldimethylsilyl)oxy)-4,4-diphenylbutan-2-ol (10ar)

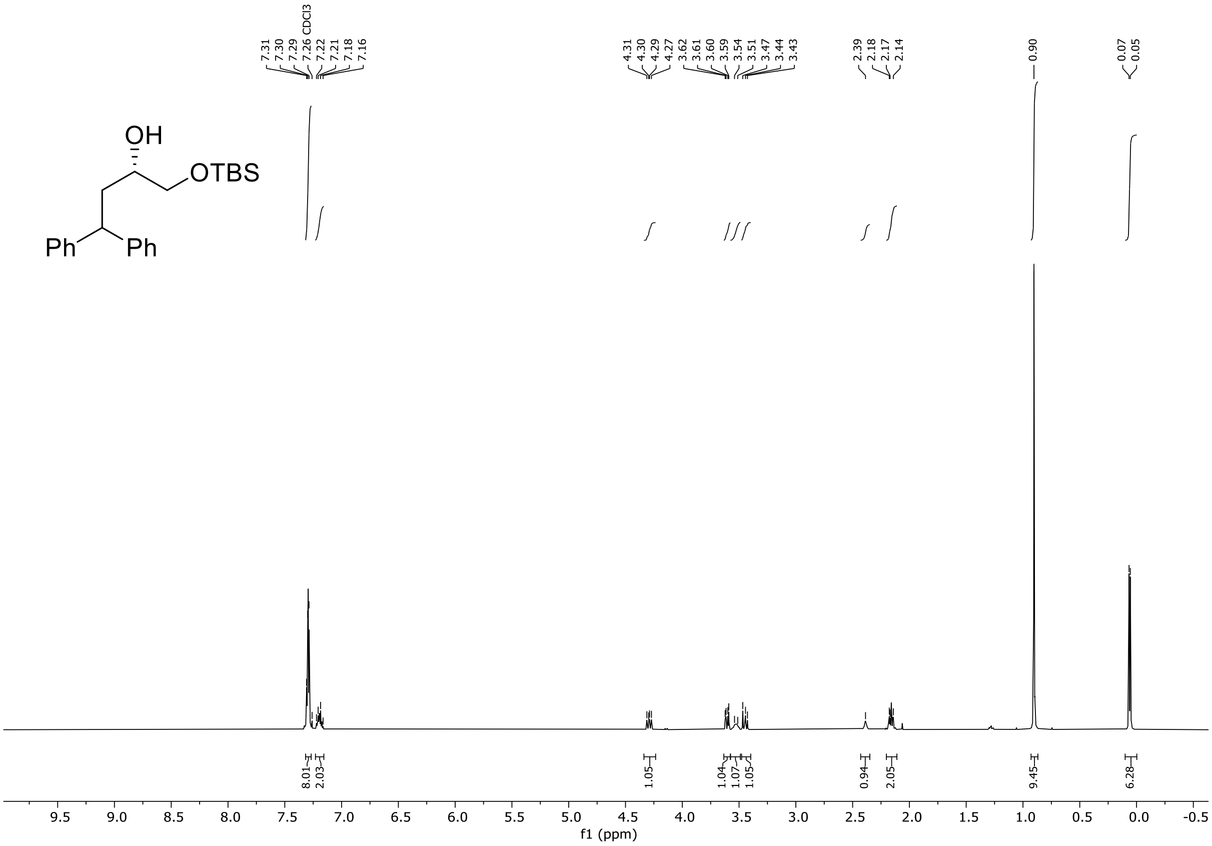

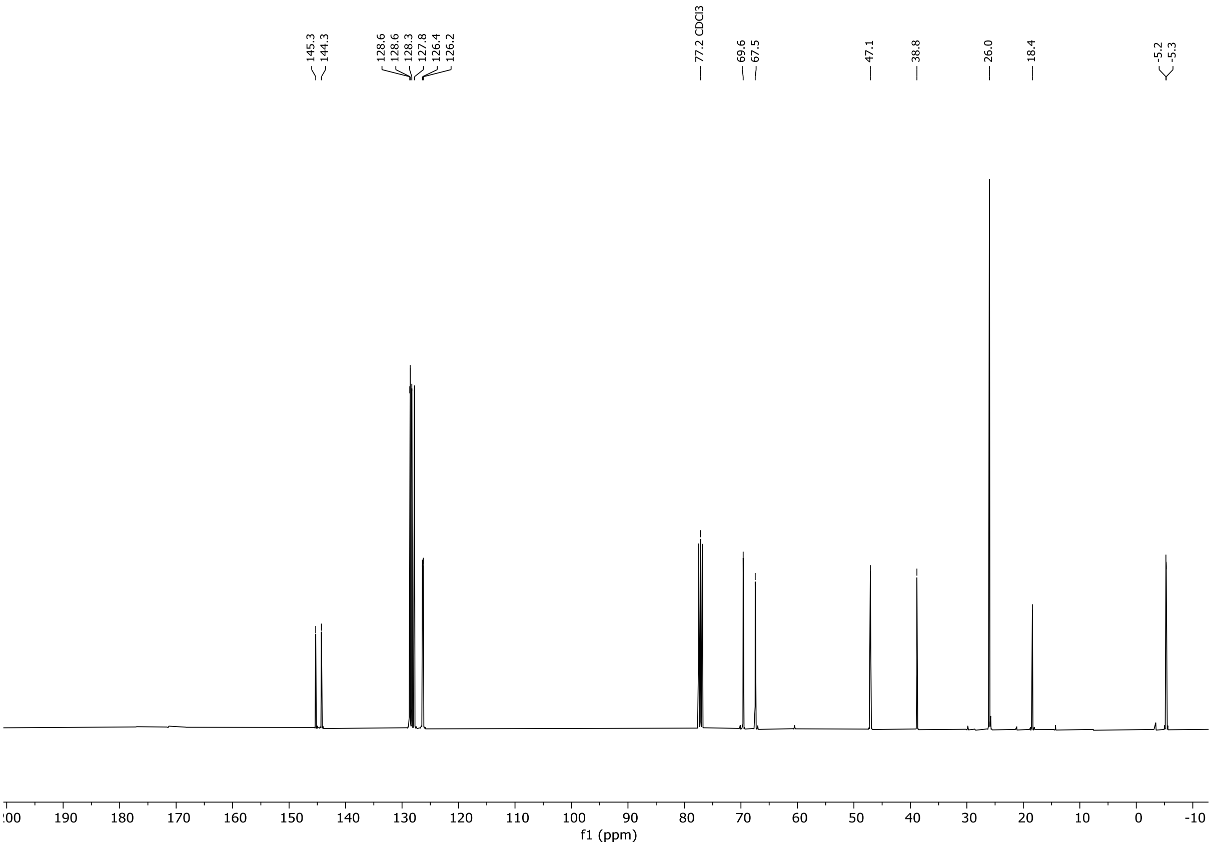

(*S*)-2-((tert-butyldimethylsilyl)oxy)-4,4-diphenylbutan-1-ol (10ar´)

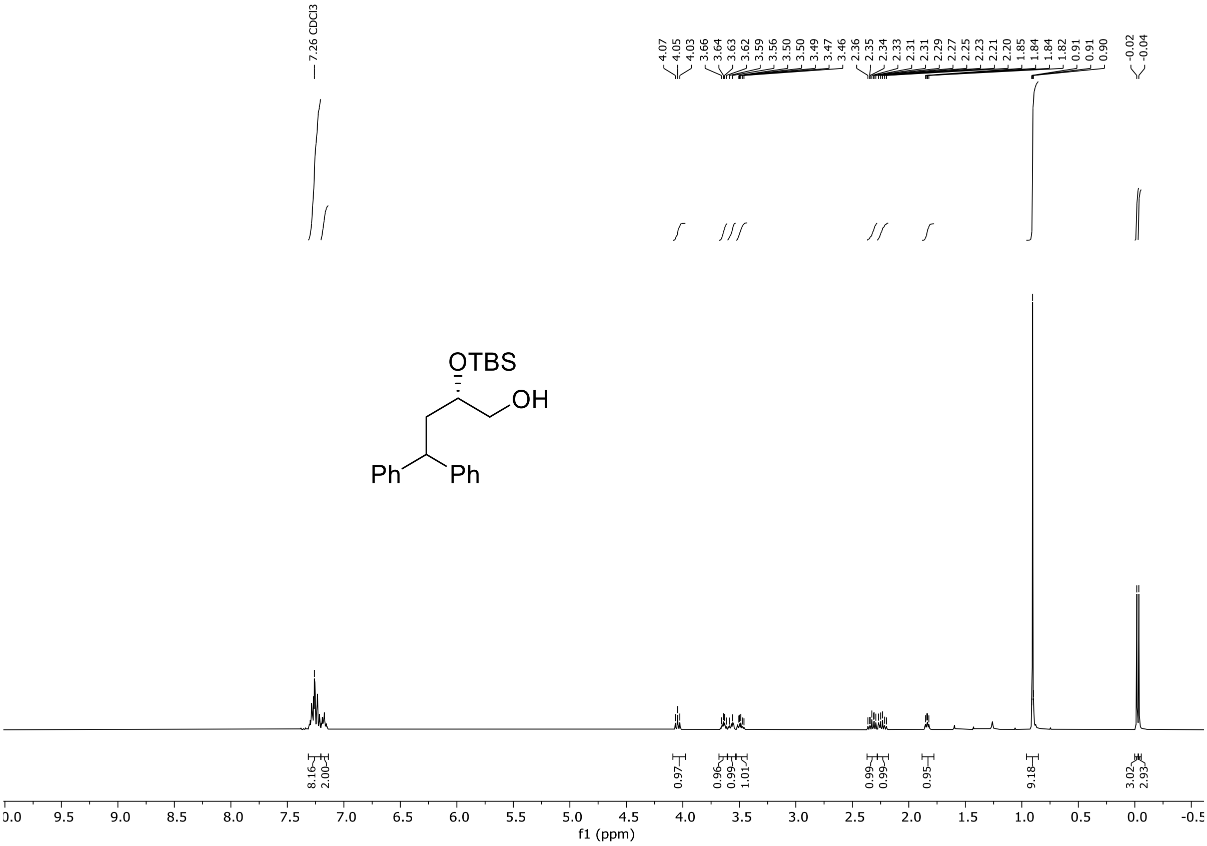

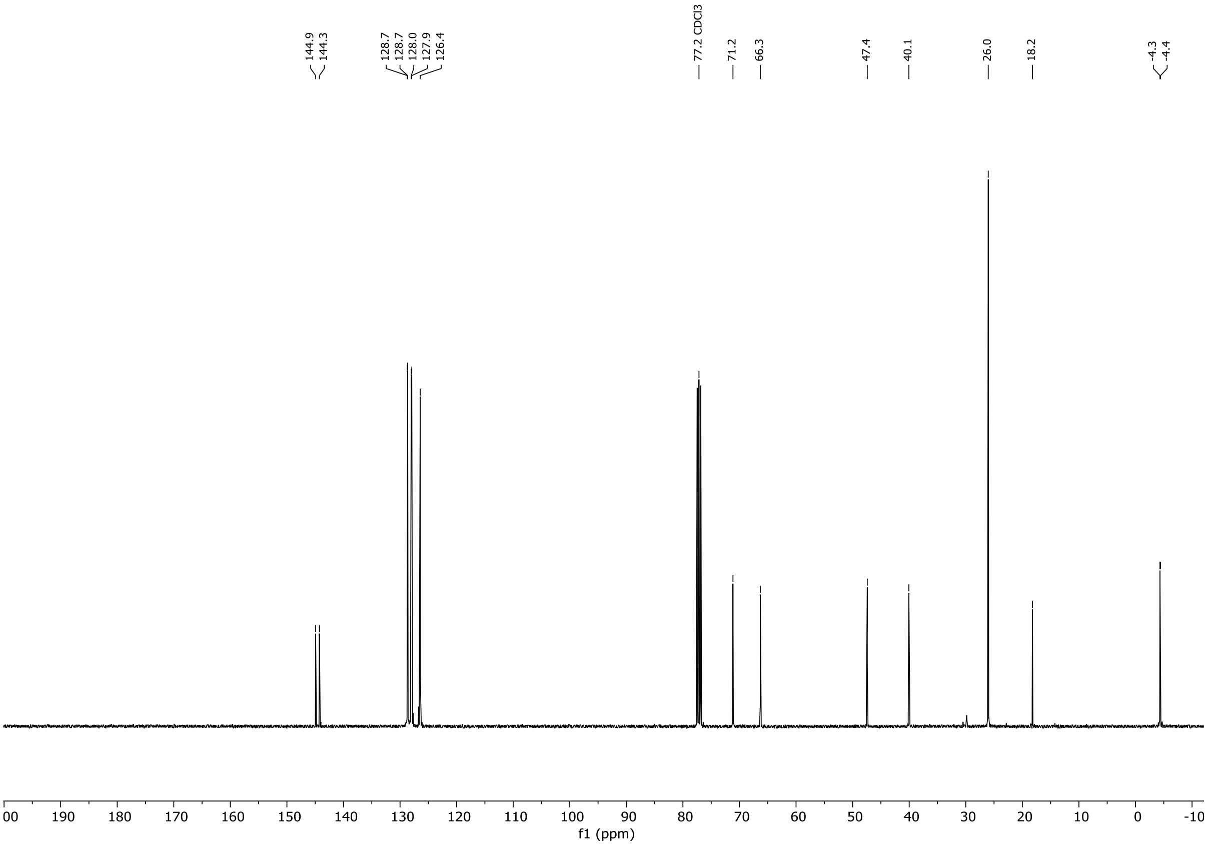

4,4-diphenylbutan-1-ol (10as)

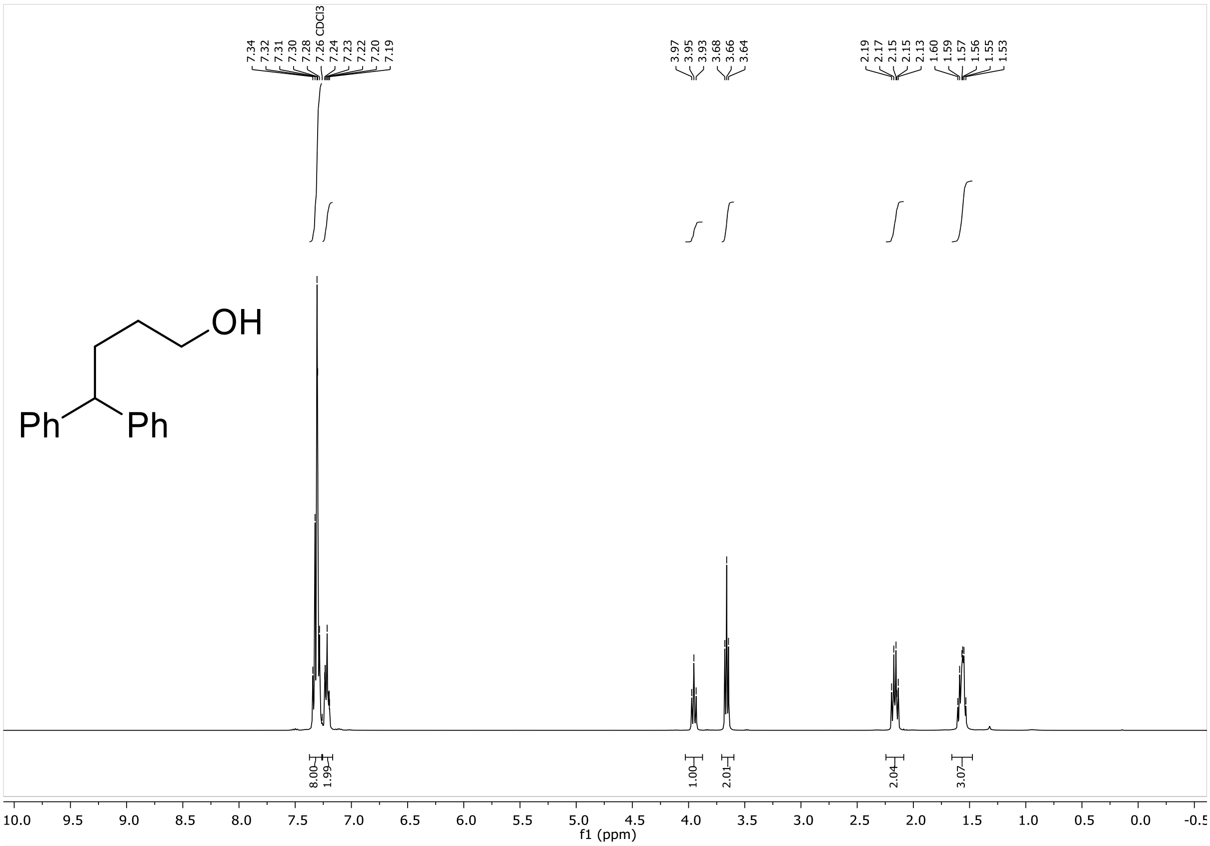

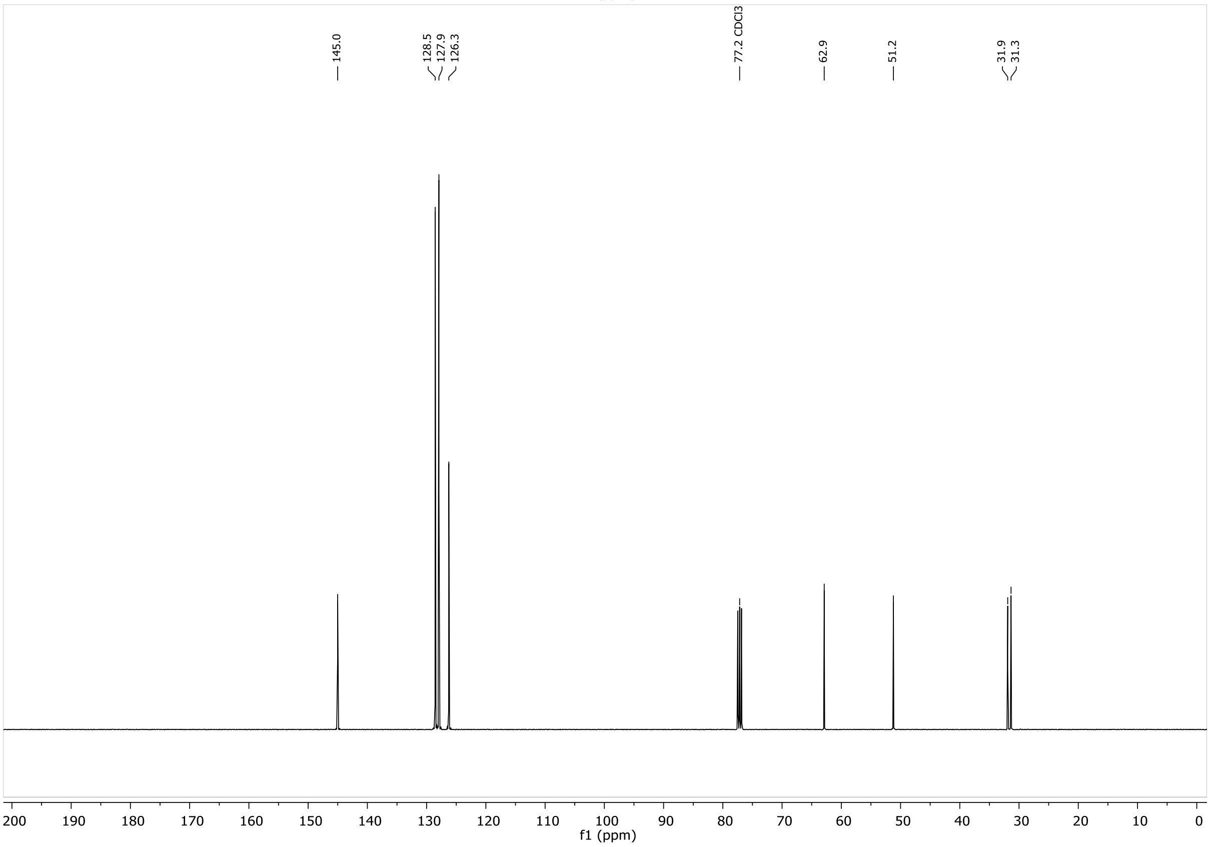

2-(phenyl-*d*$_5$)adamantan-2-ol (10at)

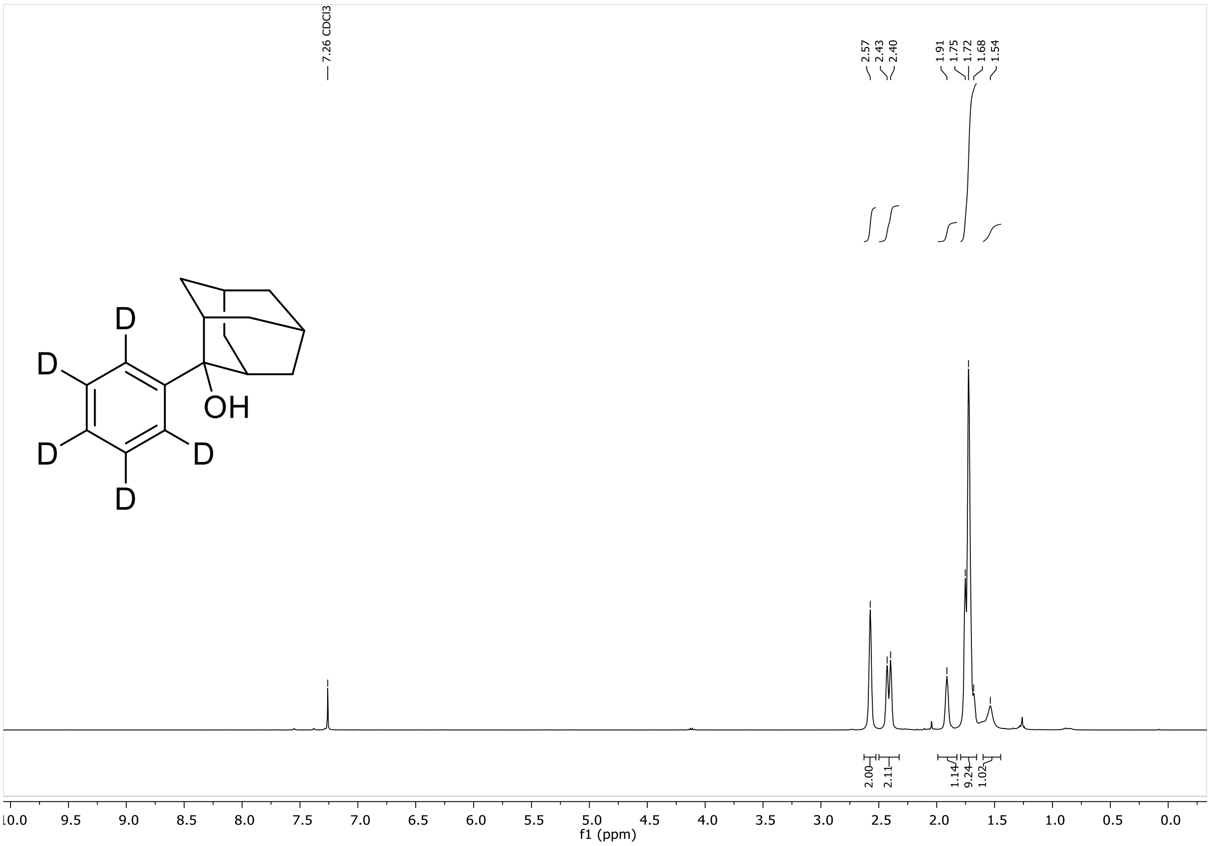

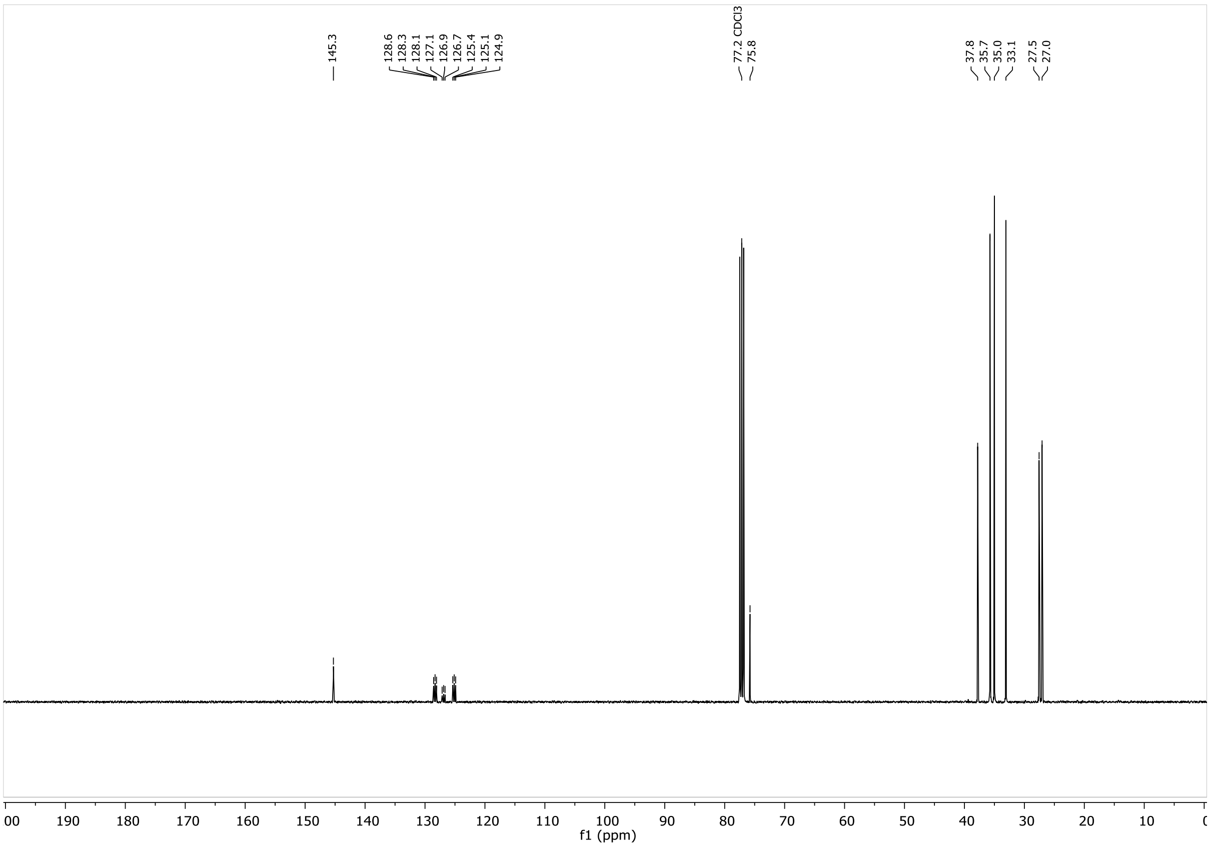

1,3-dimethyl-5-tridecylbenzene (13a)

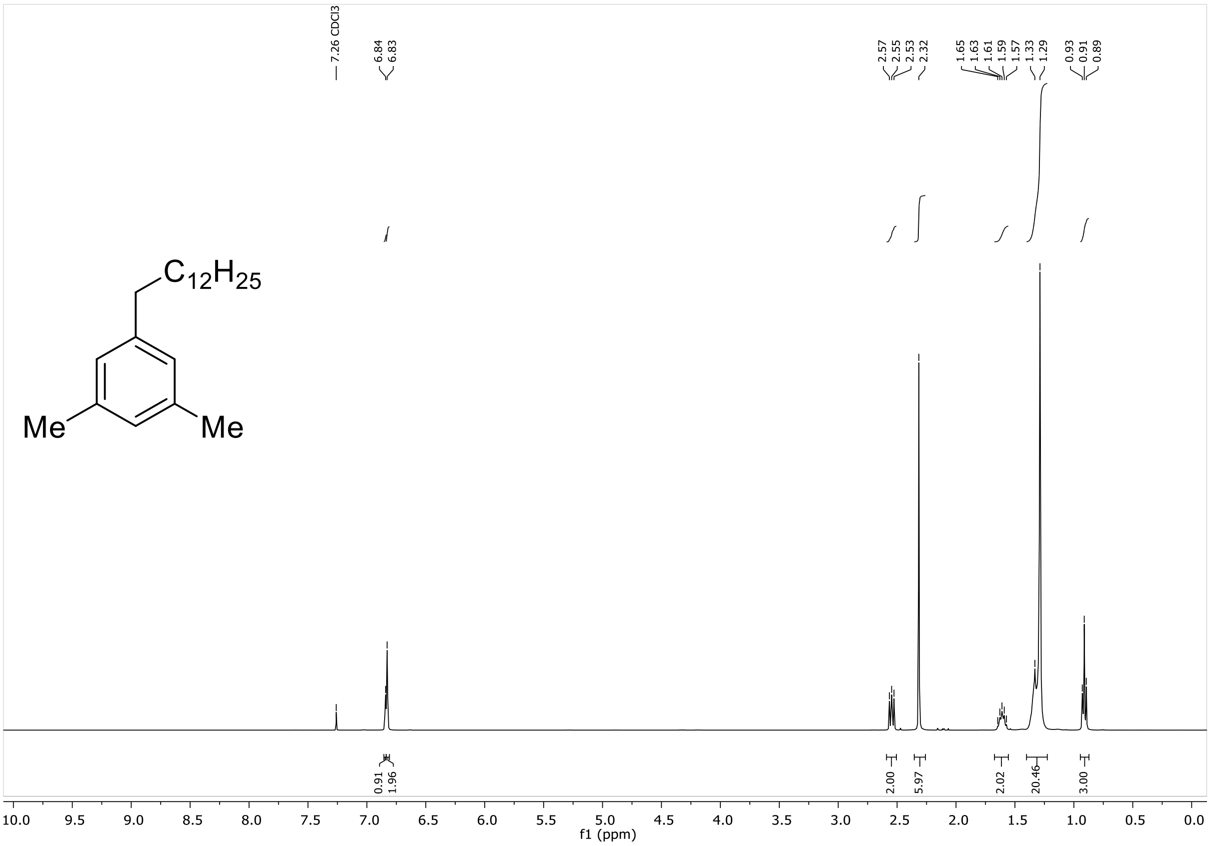

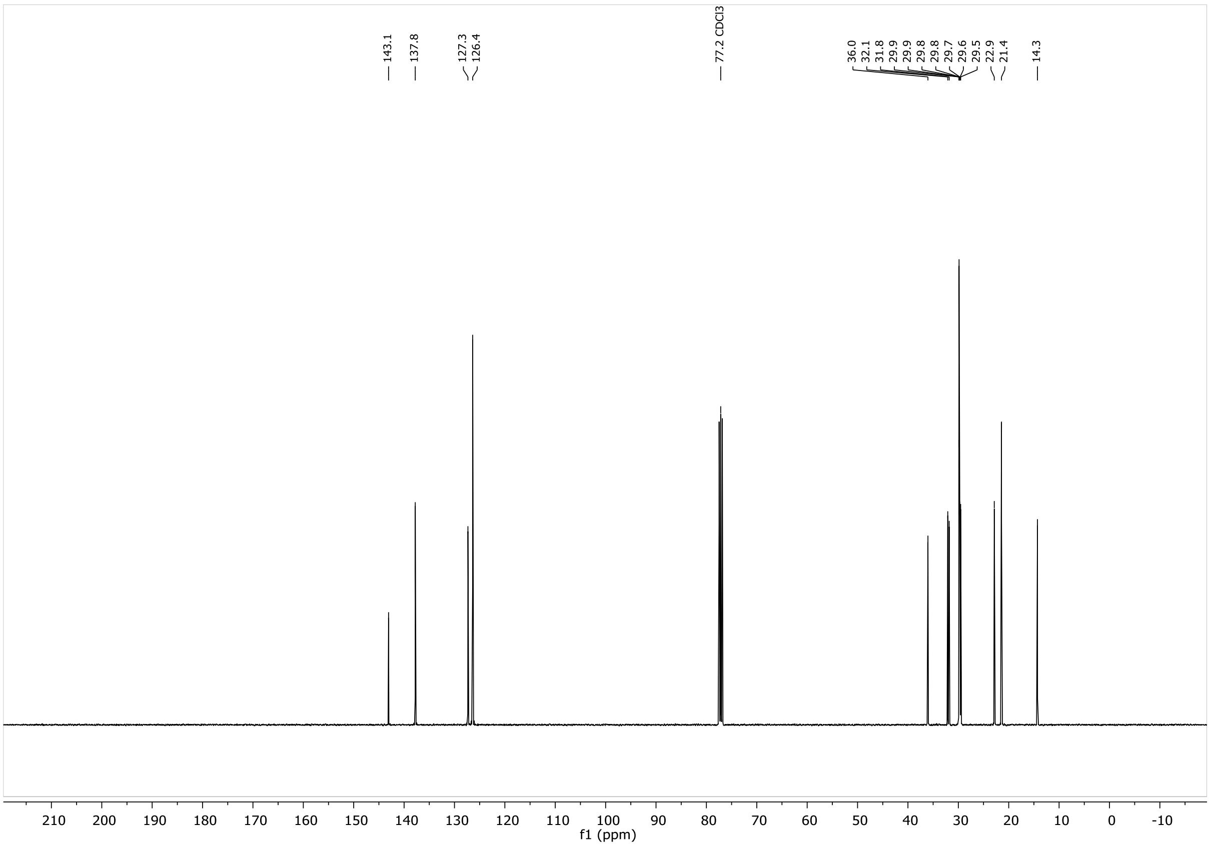

1,3-dimethyl-5-(4-phenylbutyl)benzene (13b)

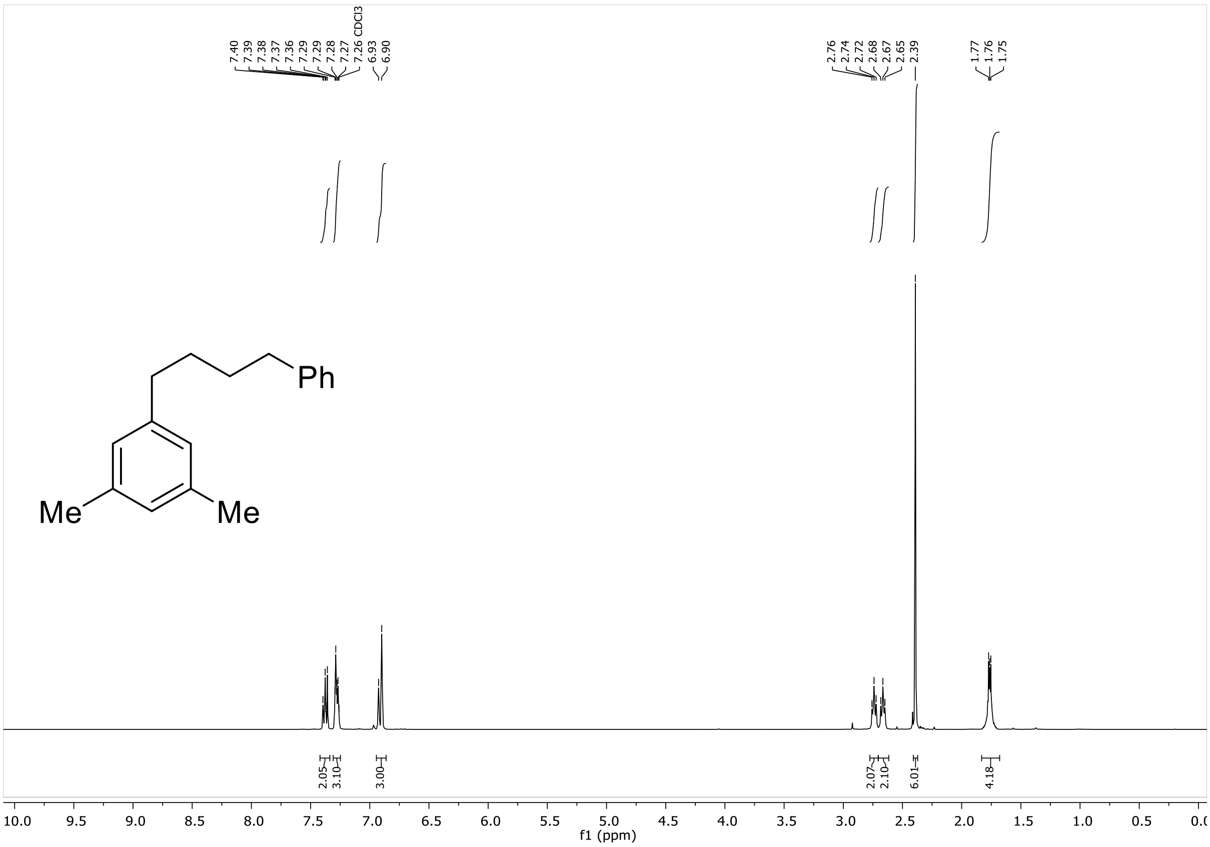

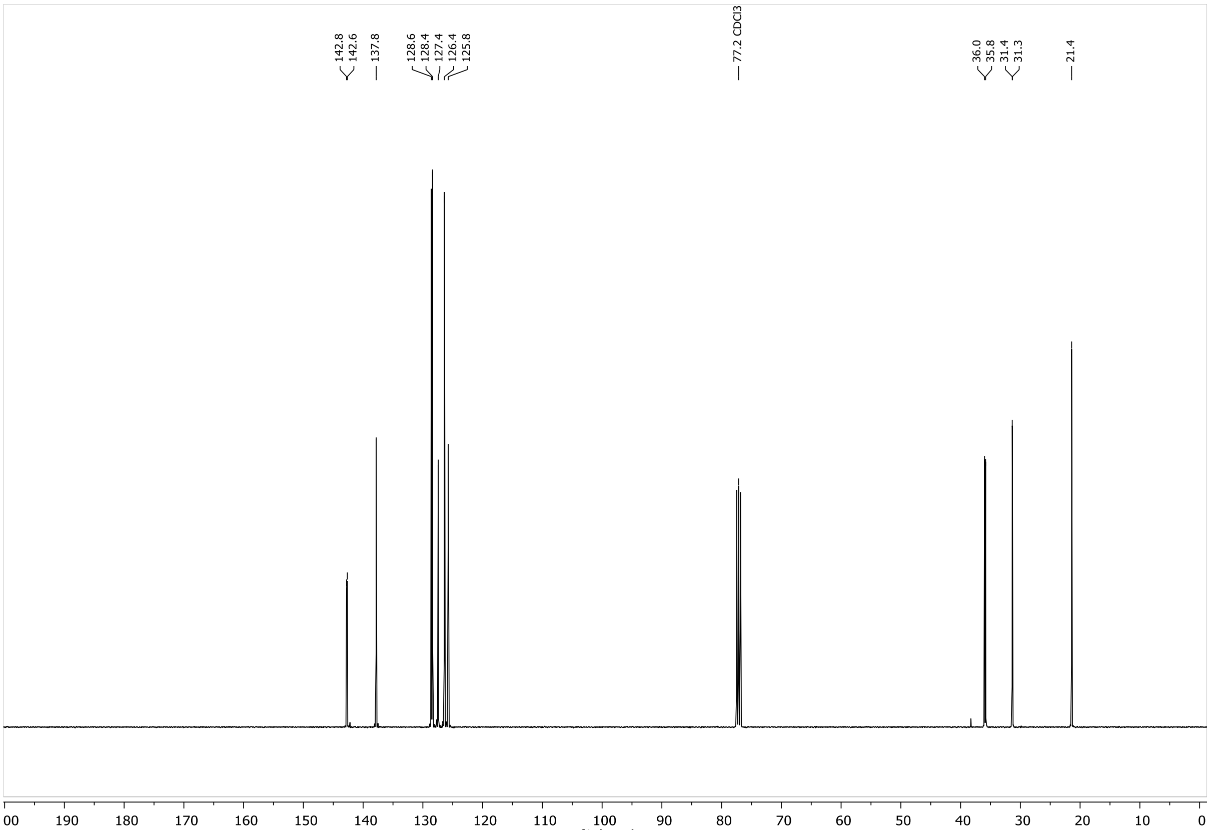

(4-(3,5-dimethylphenyl)butyl)trimethylsilane (13c)

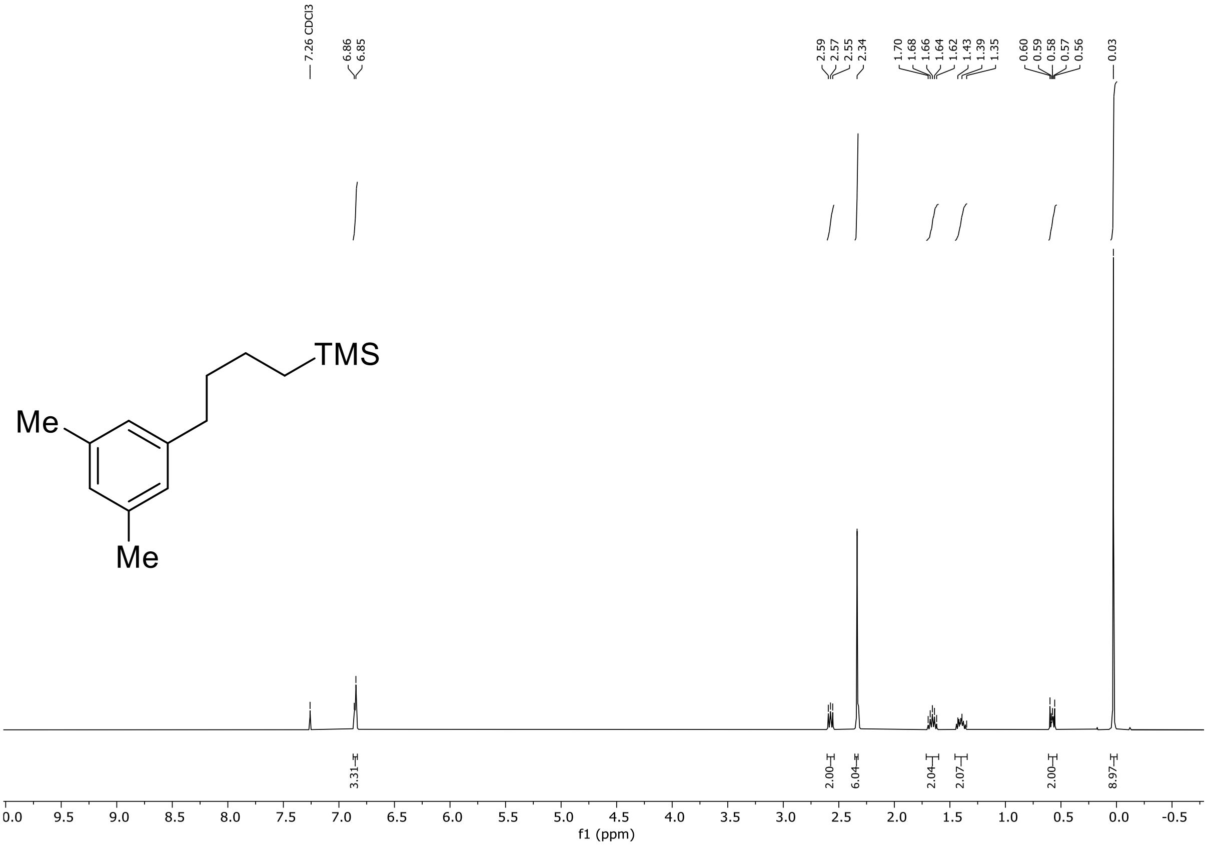

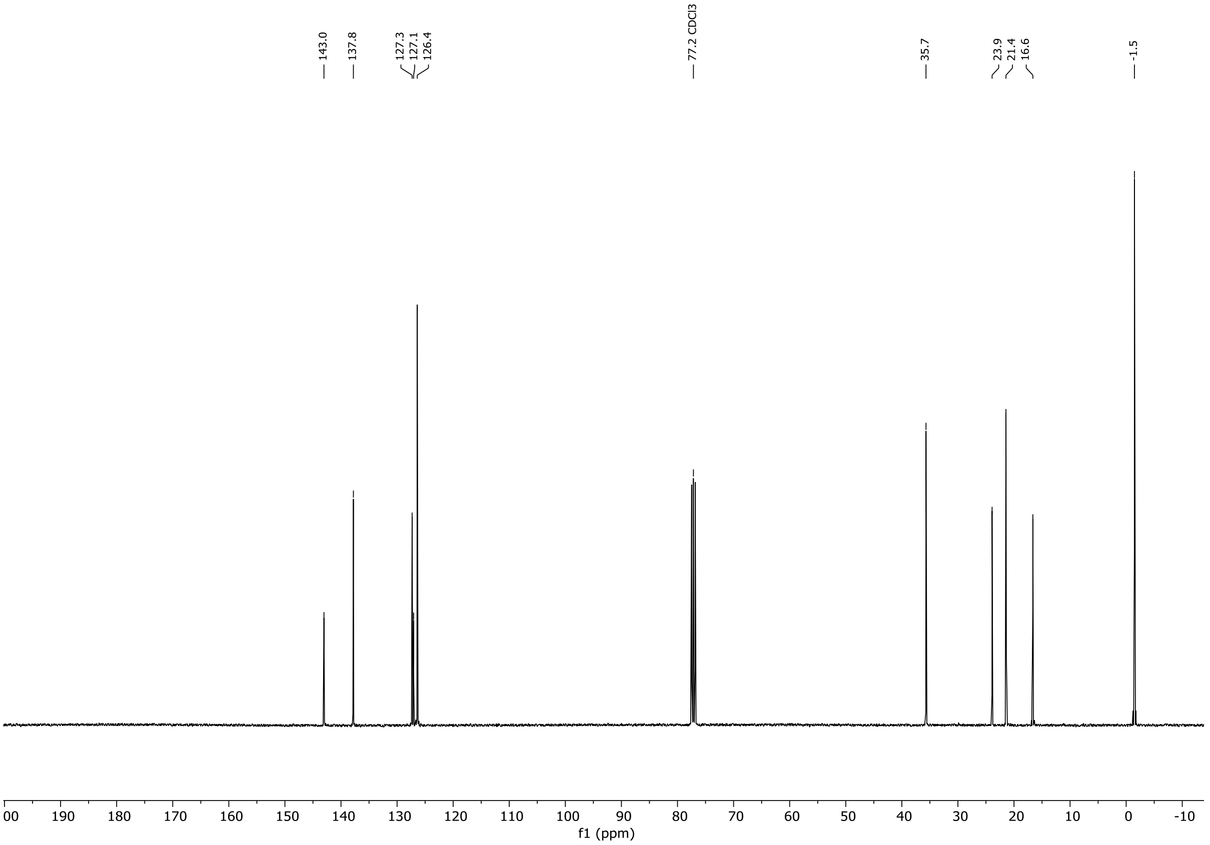

1-(((1*R*,2*S*)-2-methoxycyclohexyl)methyl)-3,5-dimethylbenzene (13d)

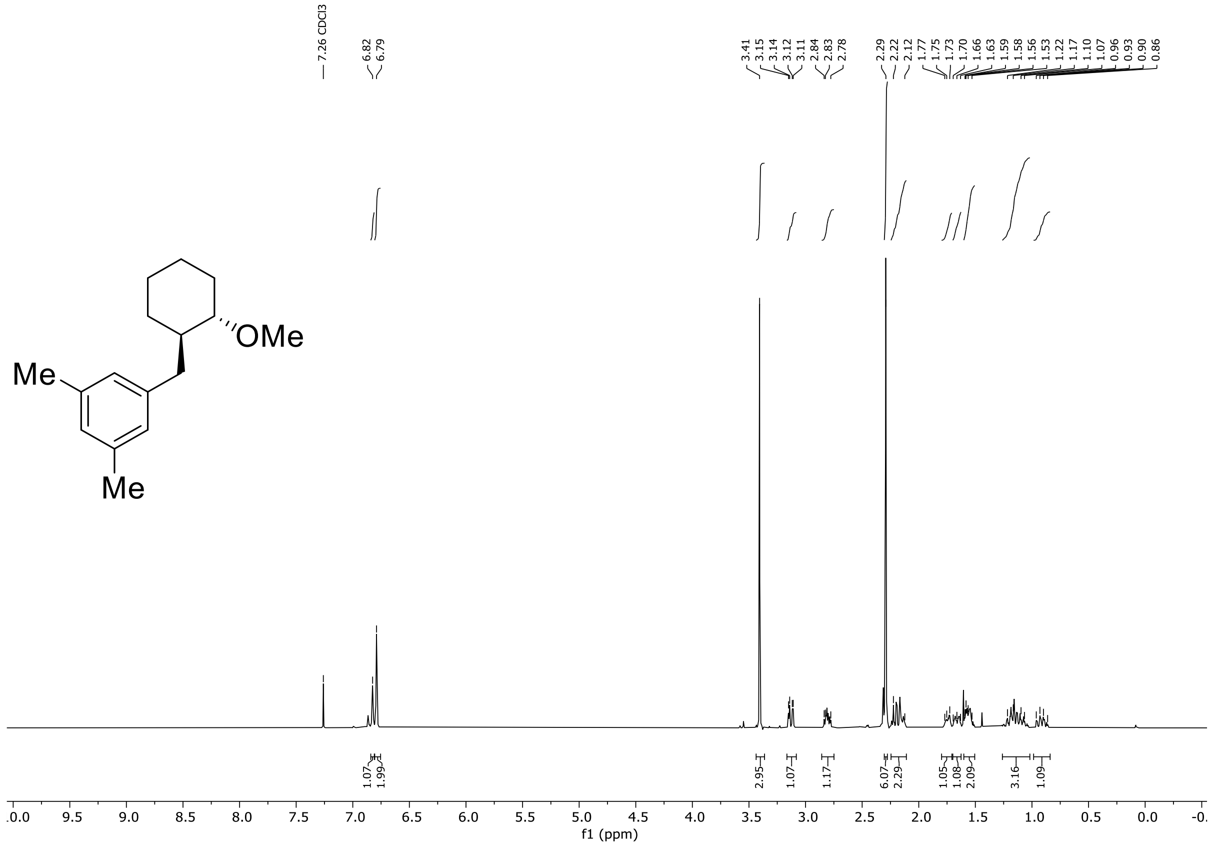

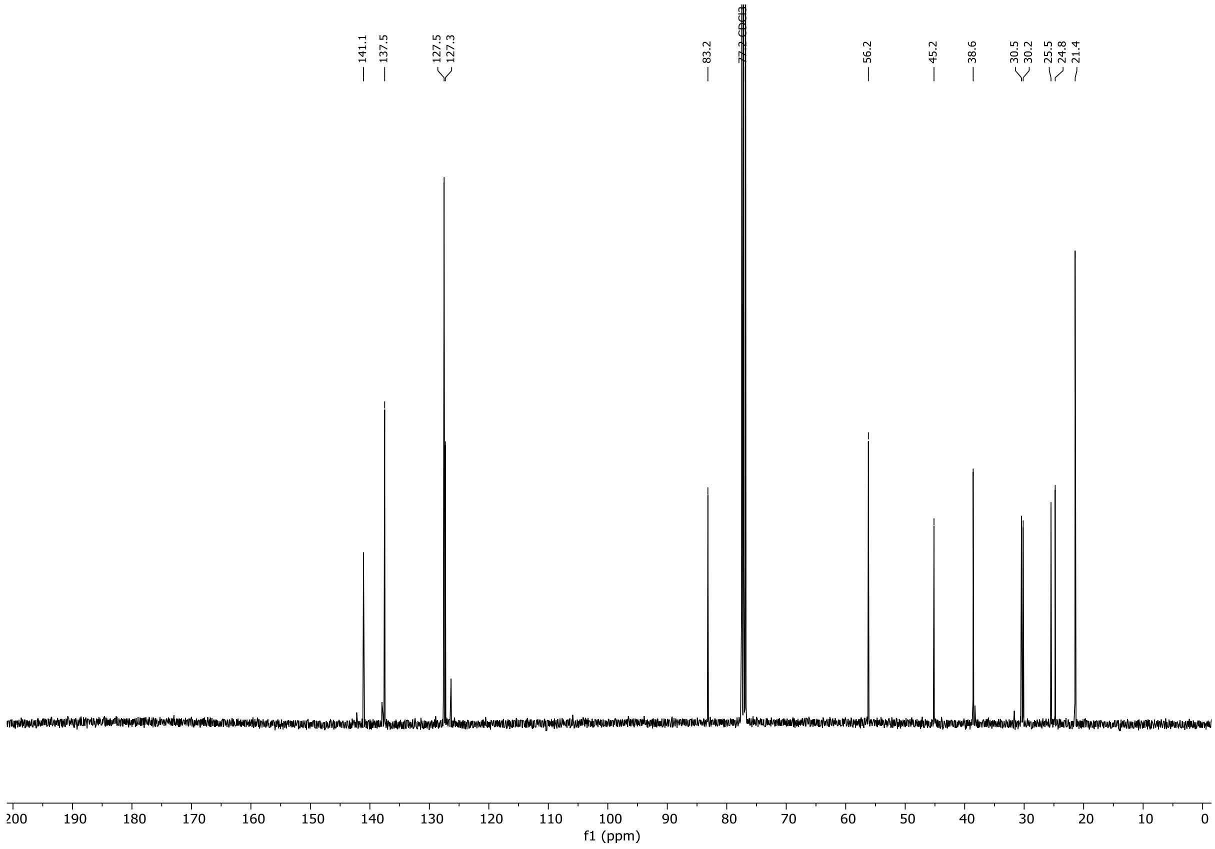

1,3-dimethyl-5-(3-(trifluoromethyl)phenethyl)benzene (13e)

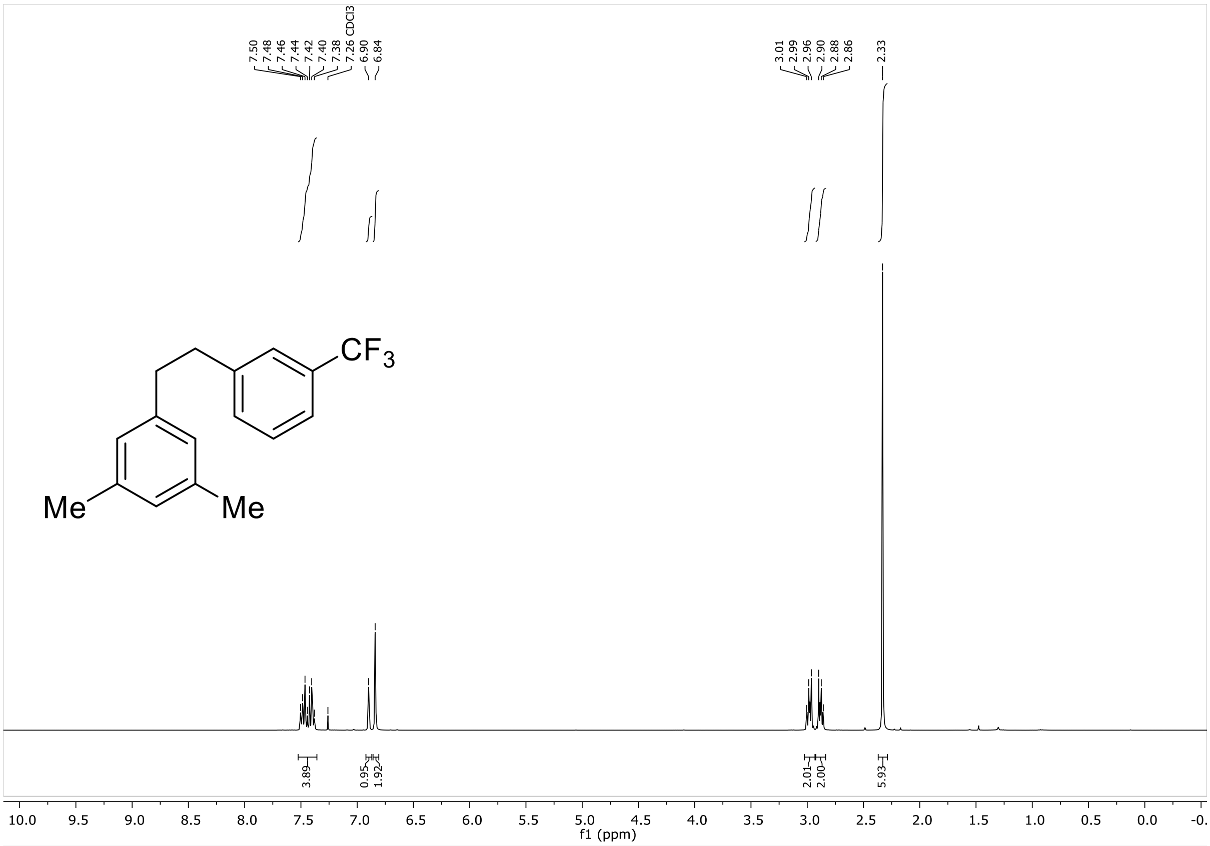

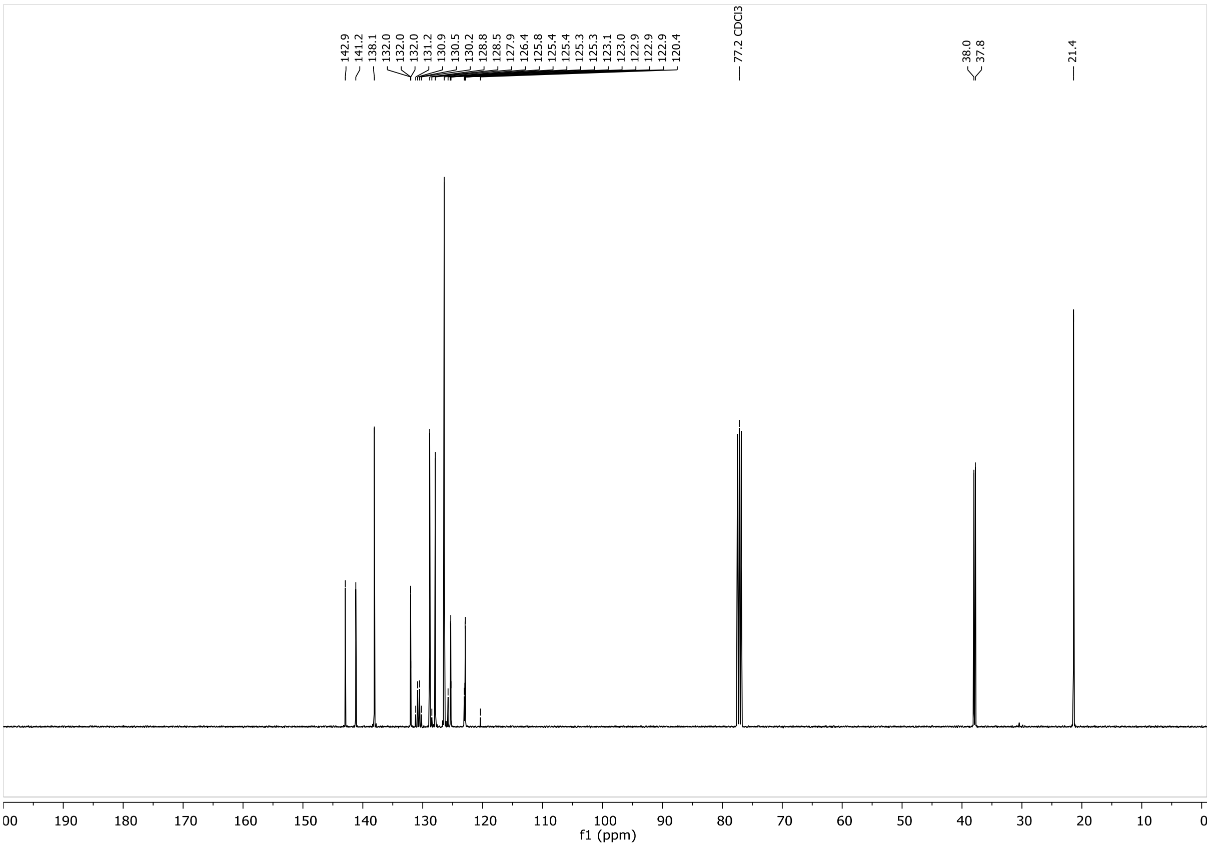

4-(3,5-dimethylphenethyl)-3,5-dimethylisoxazole (13f)

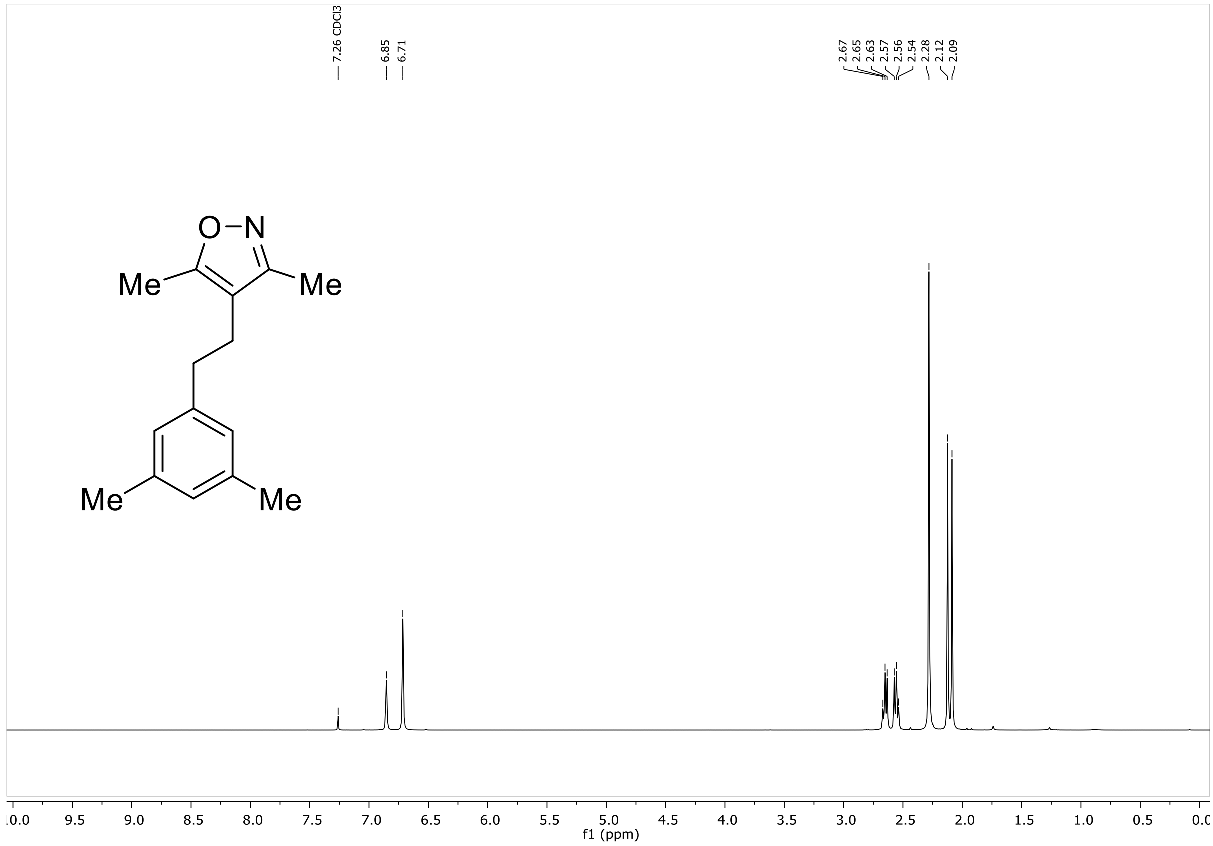

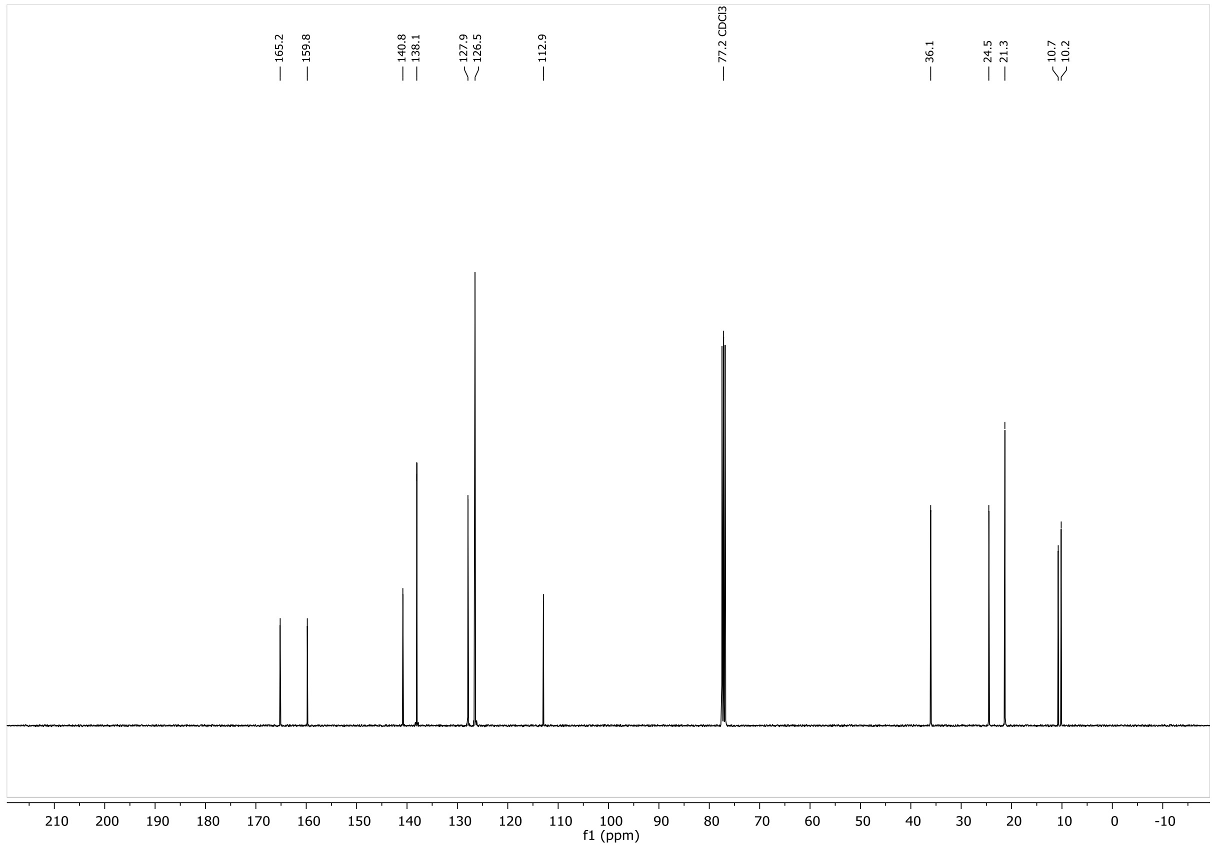

(*E*)-1,3-dimethyl-5-(4-phenylbut-3-en-1-yl)benzene (13g)

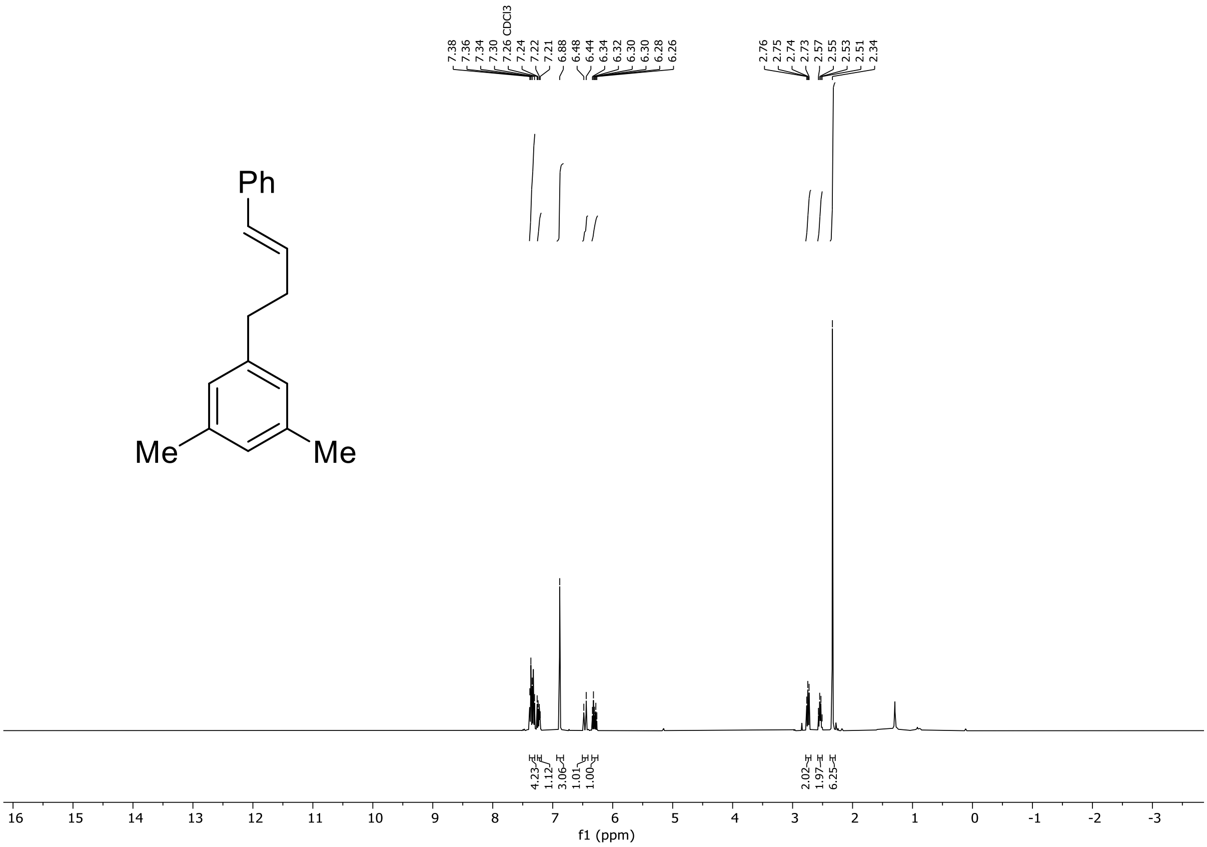

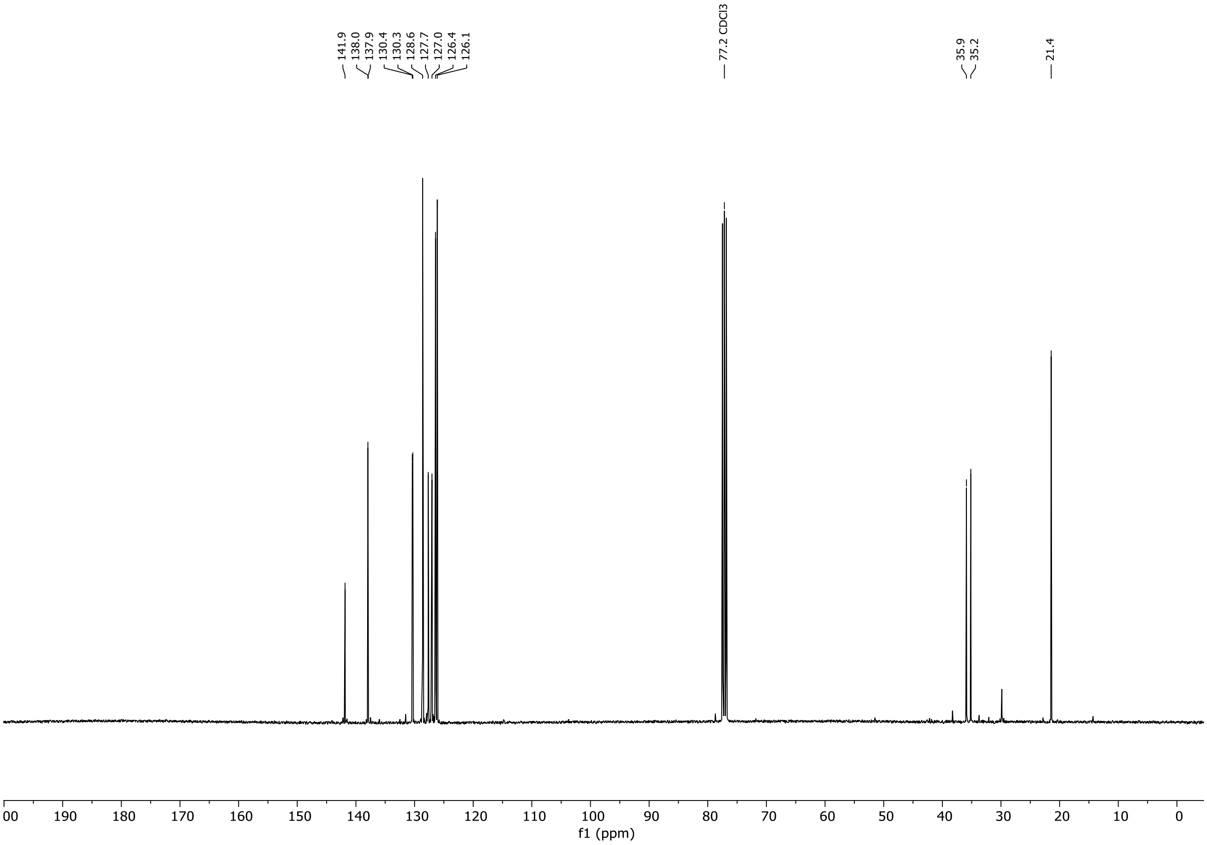

(7-chloroheptyl)benzene (13h)

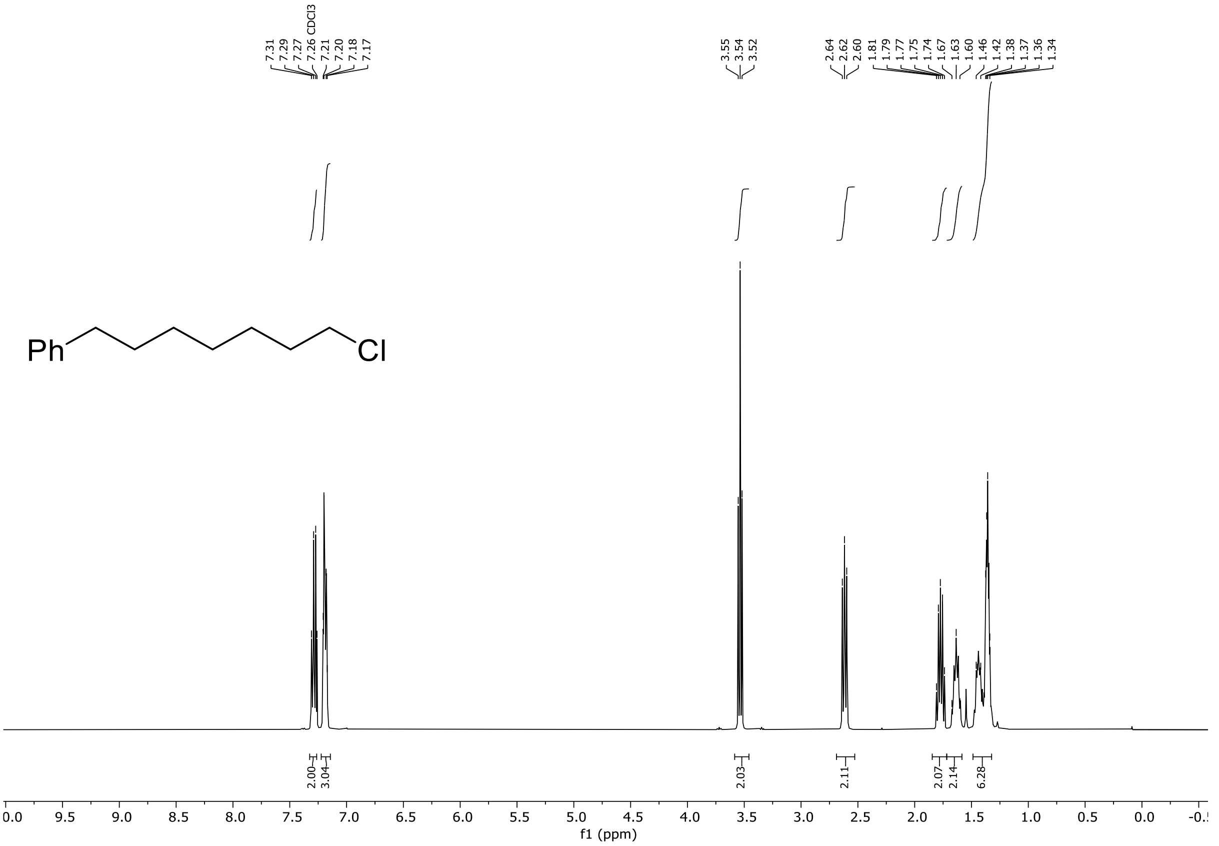

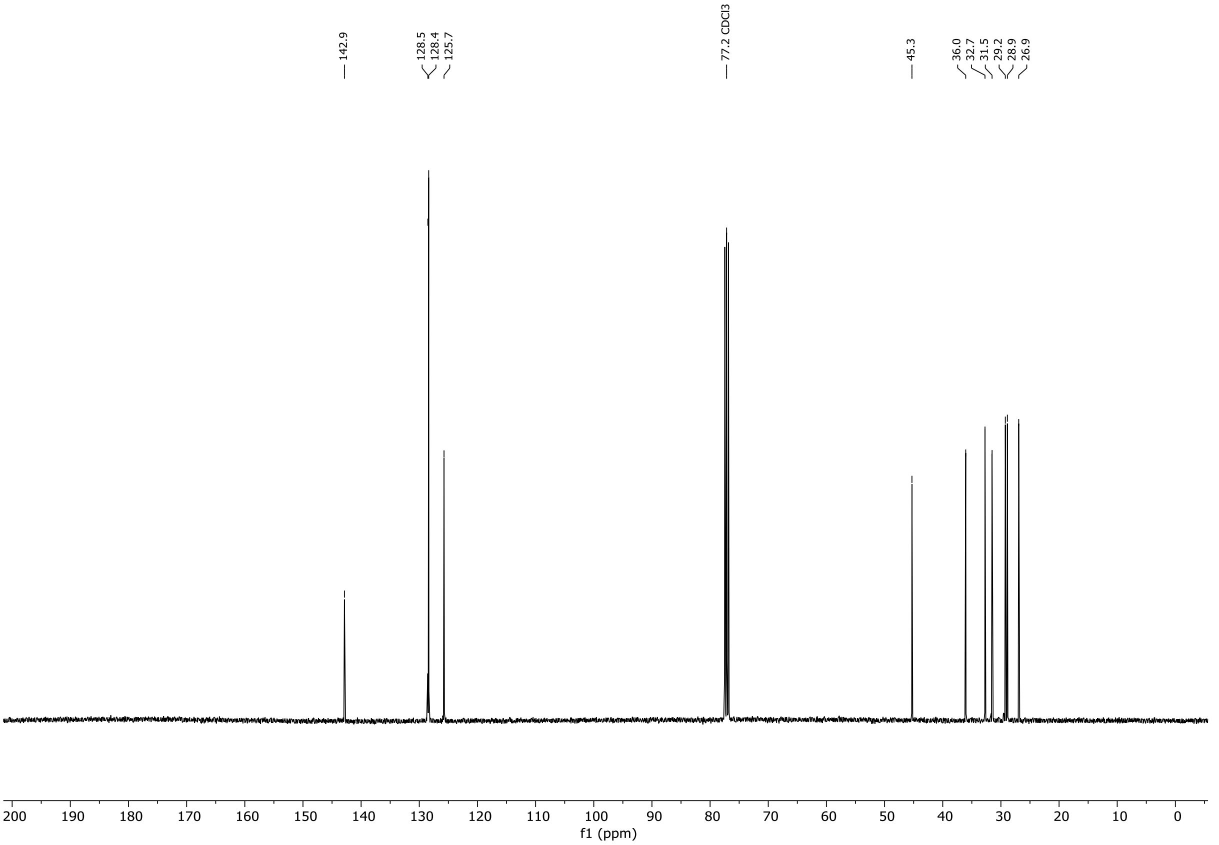

3,5-dimethyl-4-(2-(naphthalen-1-yl)ethyl)isoxazole (13i)

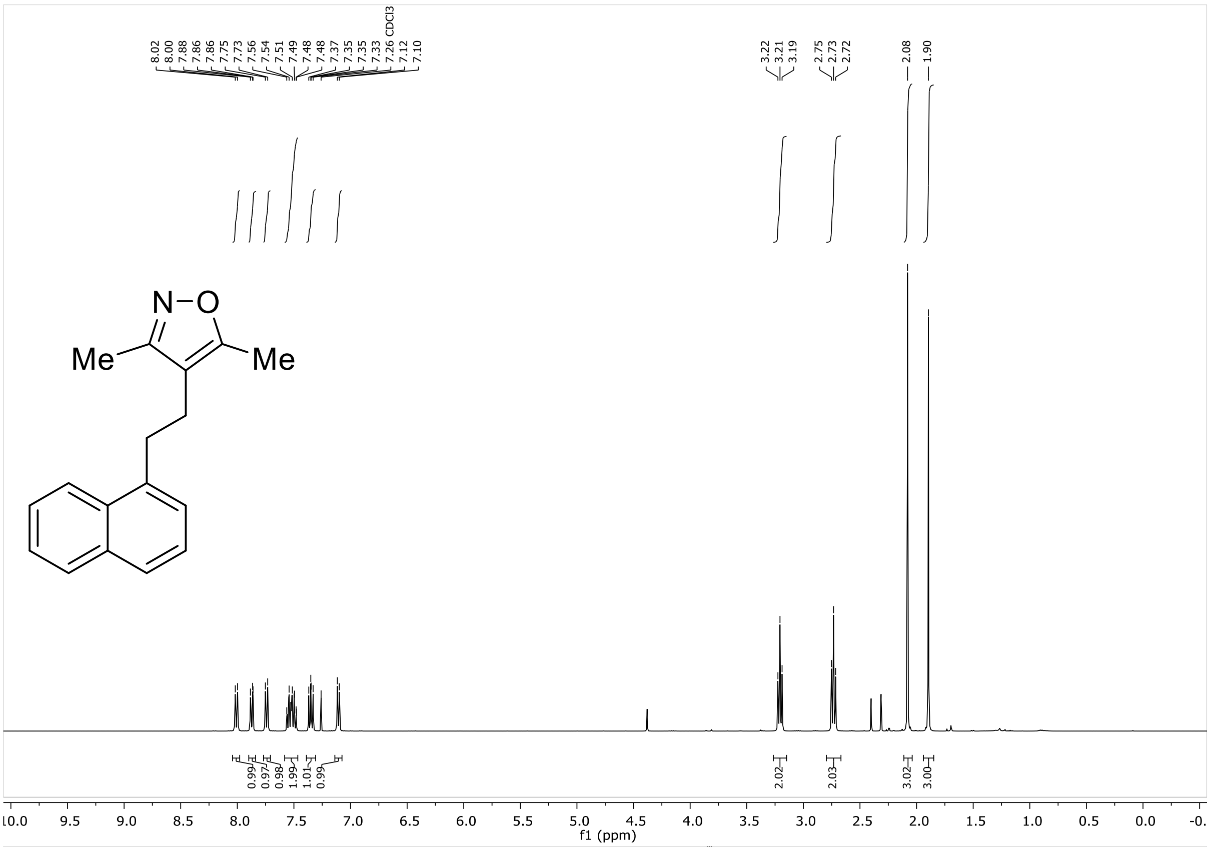

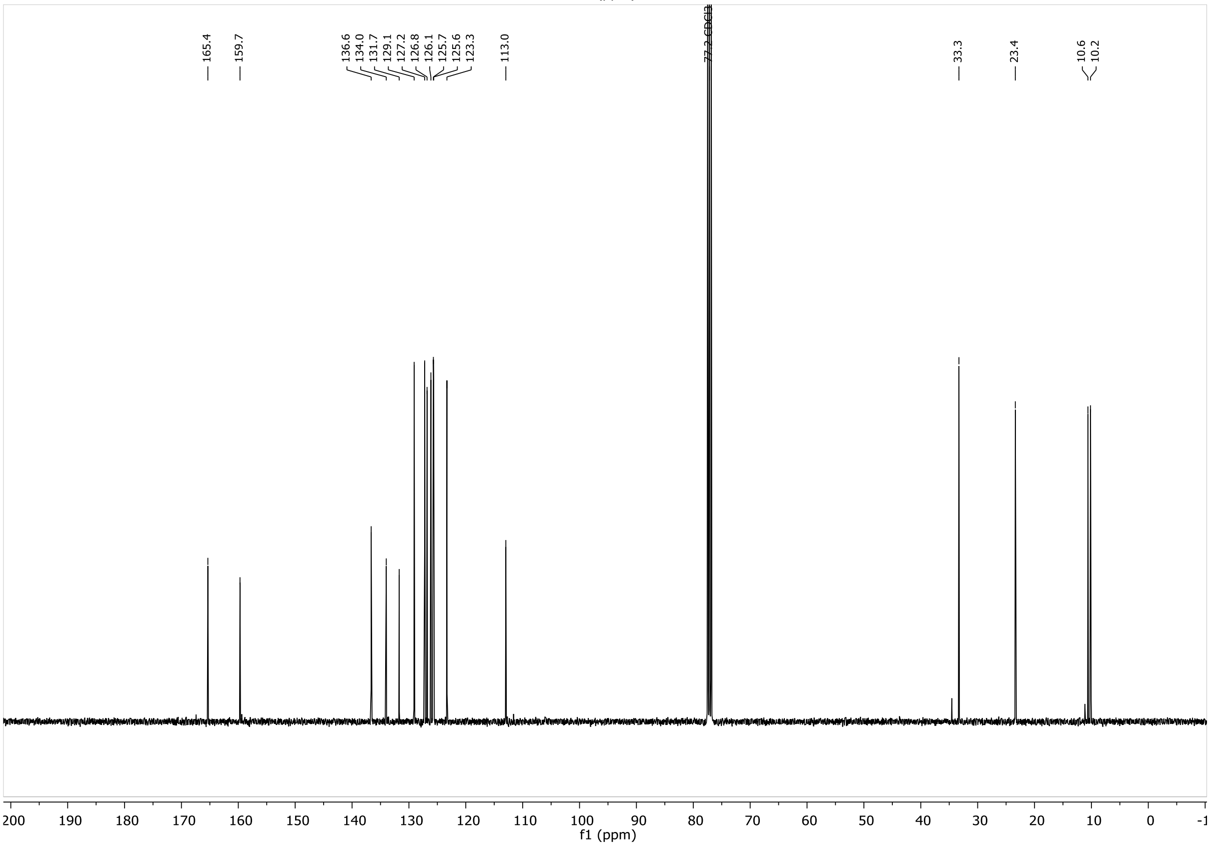

1-(5-chloropentyl)naphthalene (13j)

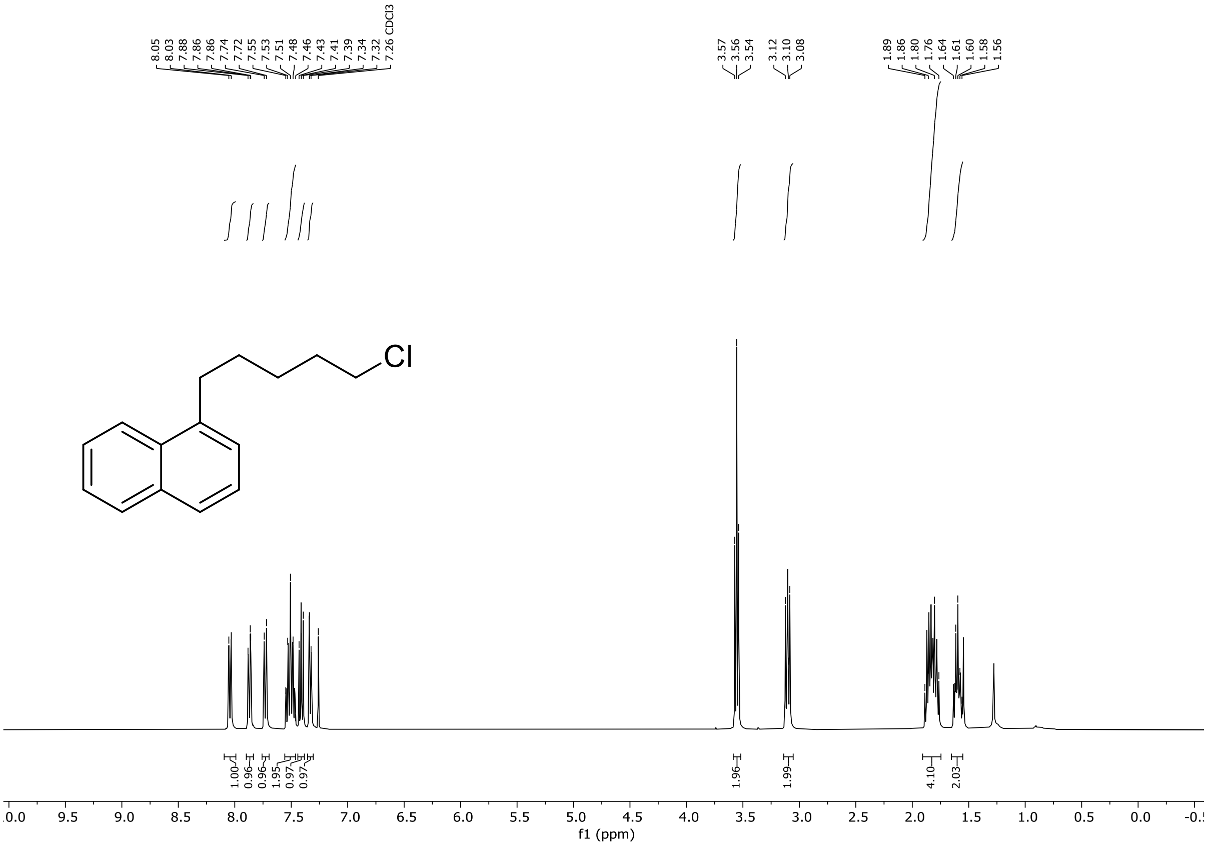

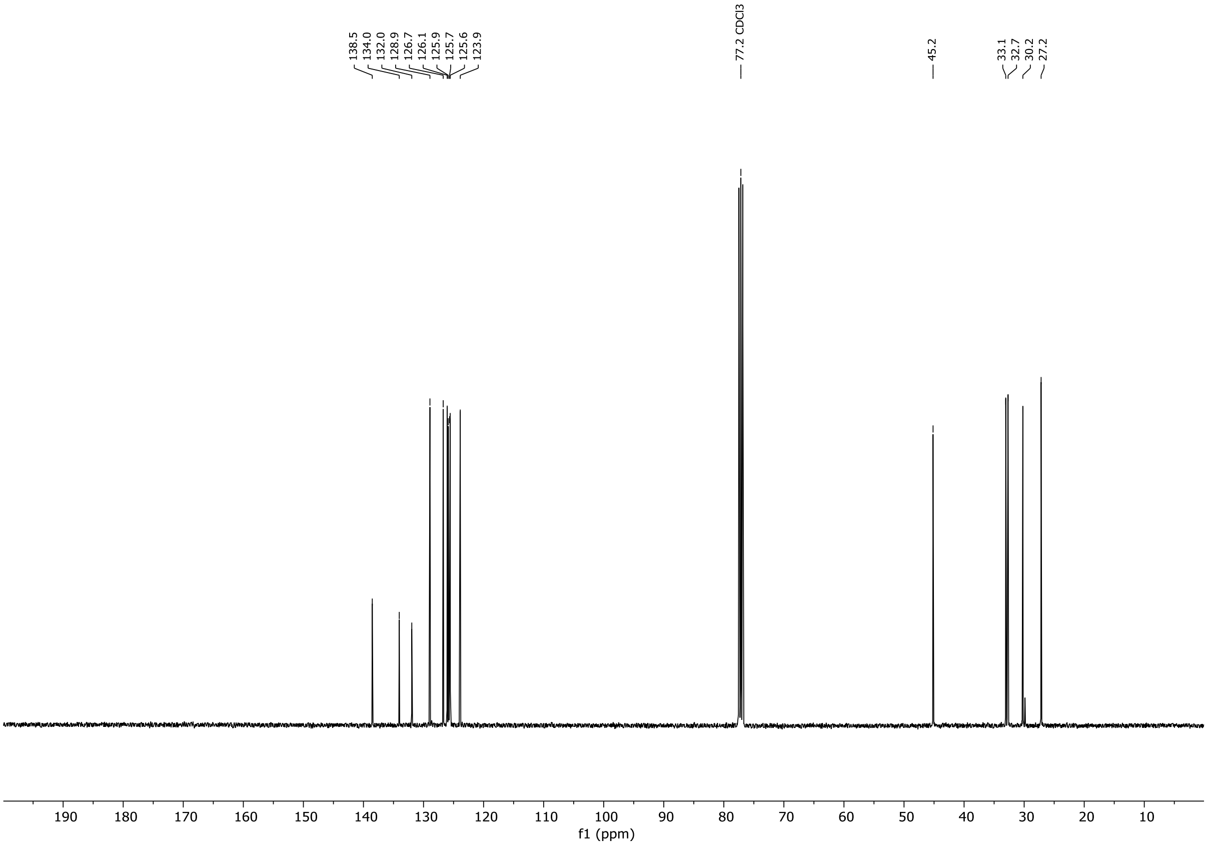

(1-((1*R*,2*S*)-2-methoxycyclohexyl)ethyl)benzene (13k) mixture of diastereoisomeres

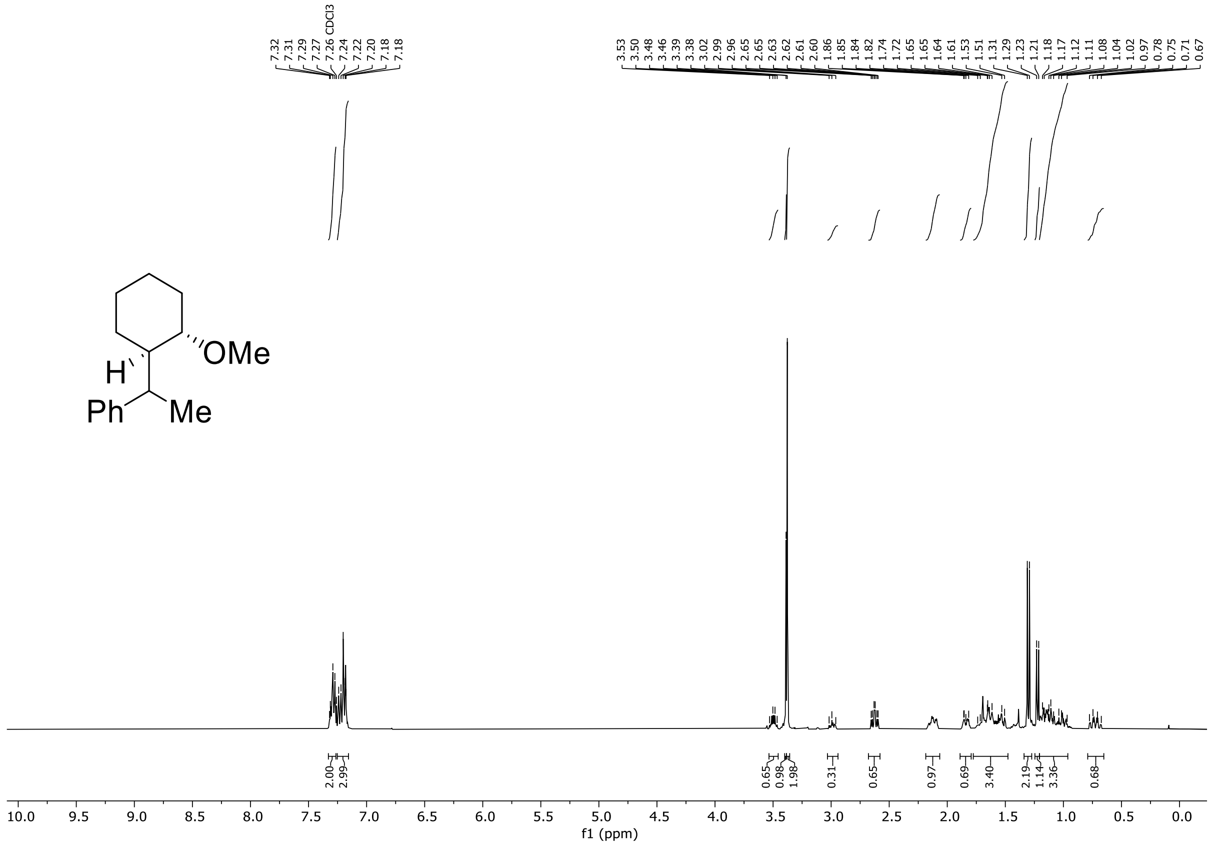

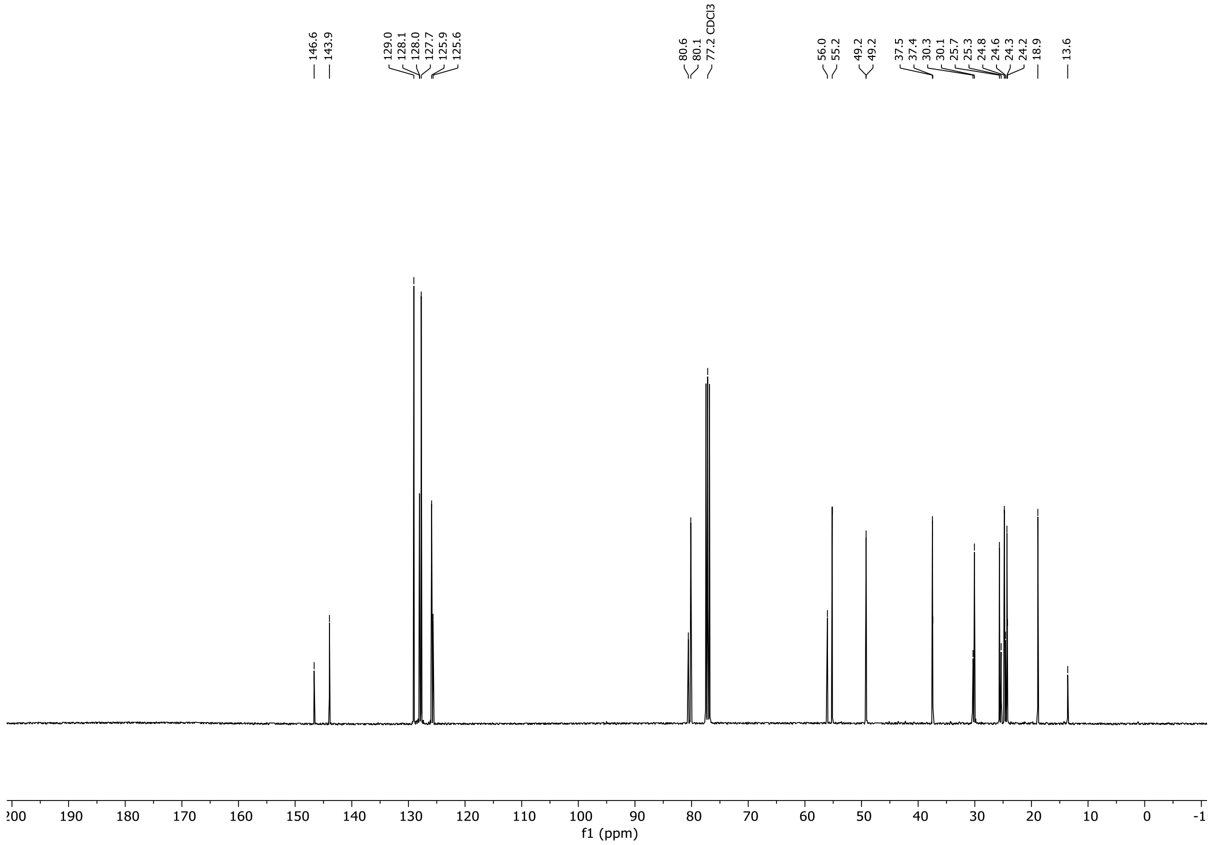

1-(4-chlorobutyl)-1,2,3,4-tetrahydronaphthalene (13l)

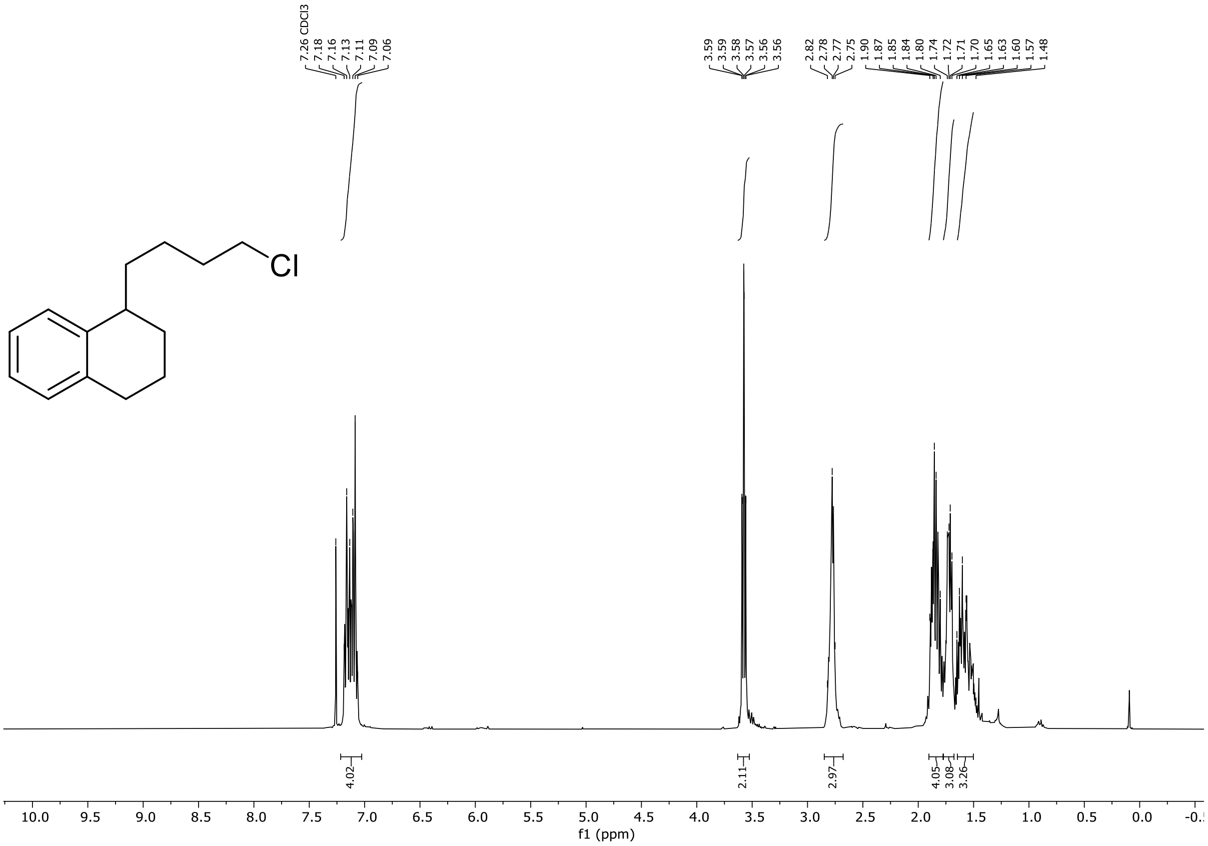

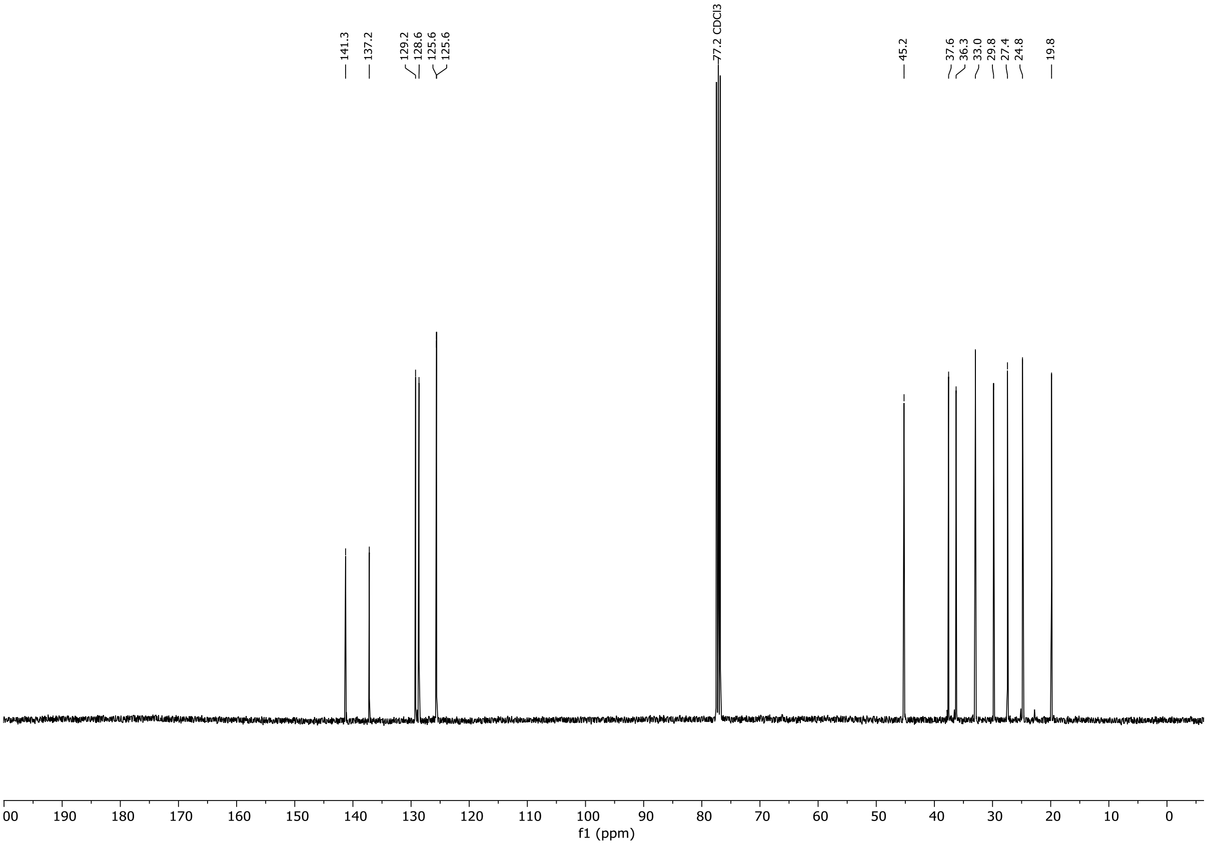

(3-(4-methoxyphenyl)propane-1,1-diyl)dibenzene (13m)

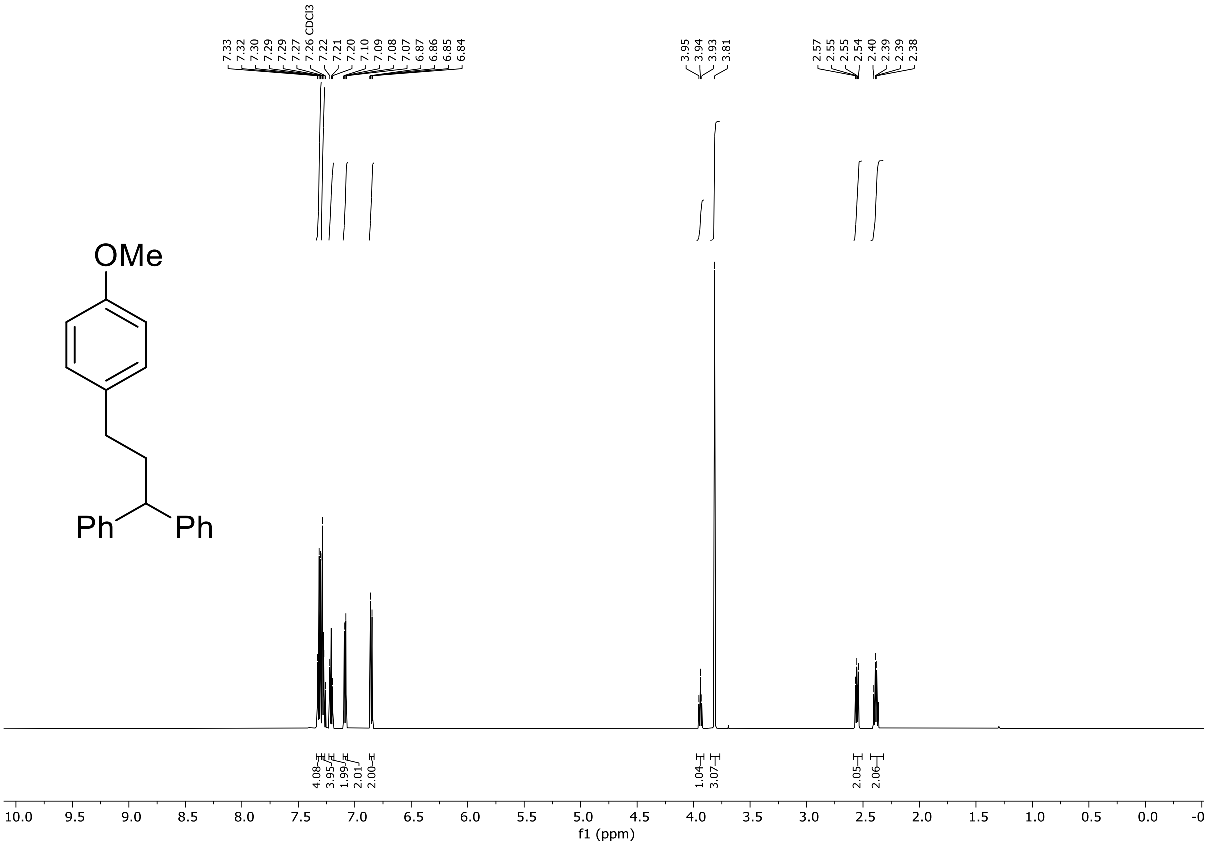

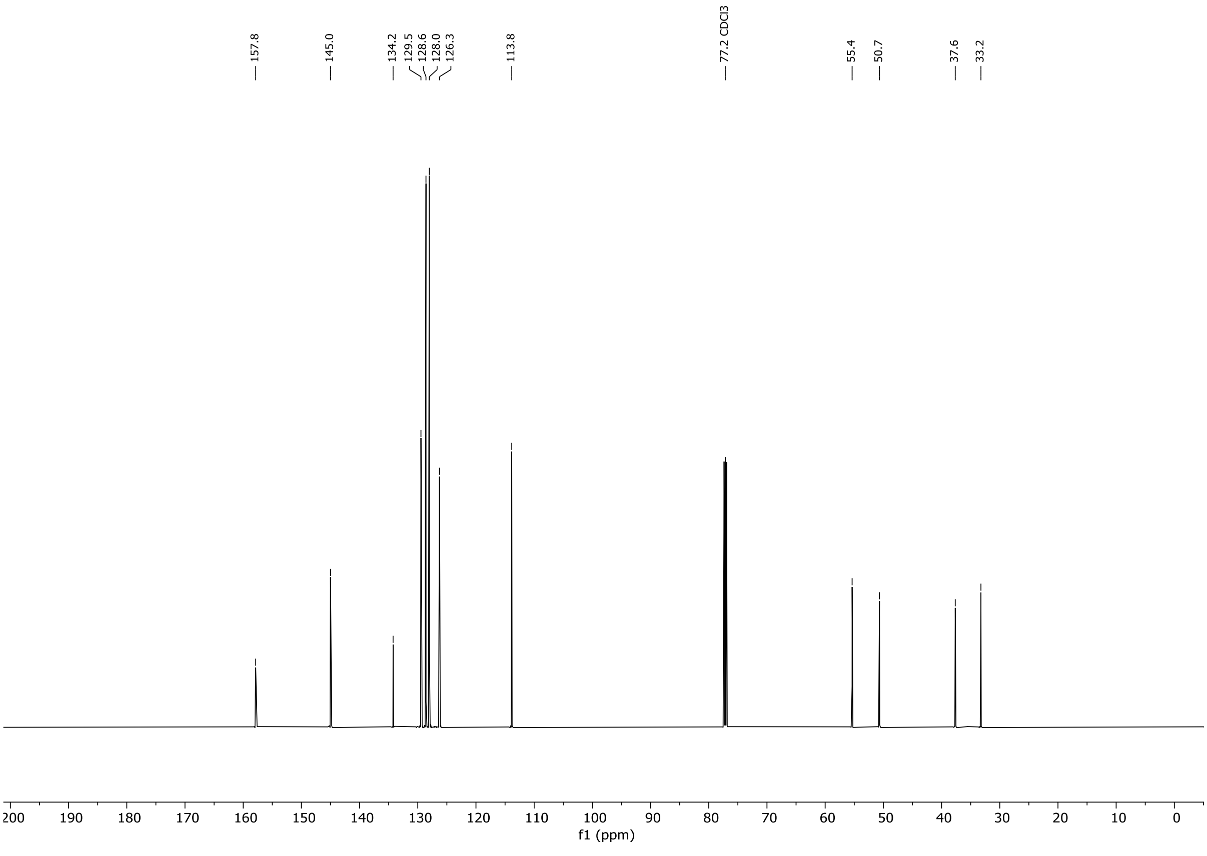

2-(2,2-diphenylethyl)tetrahydrofuran (13n)

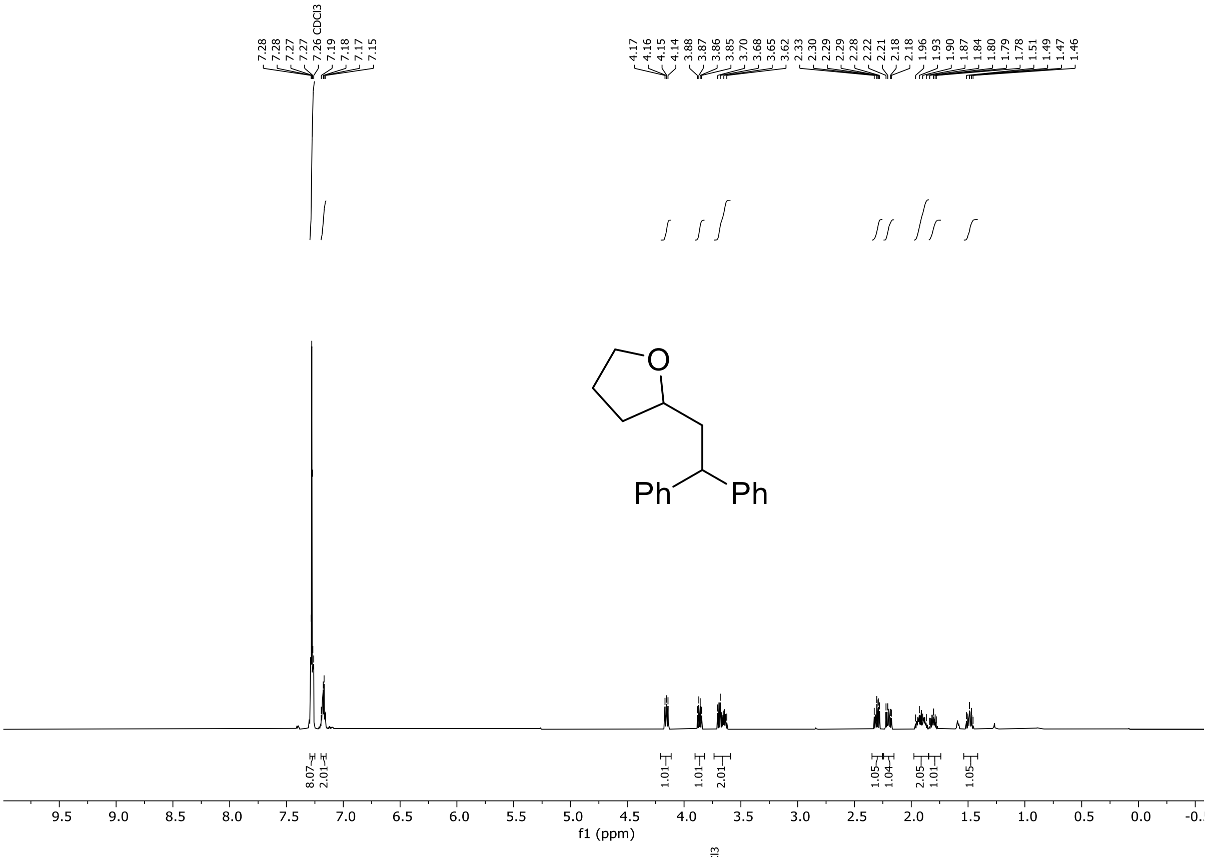

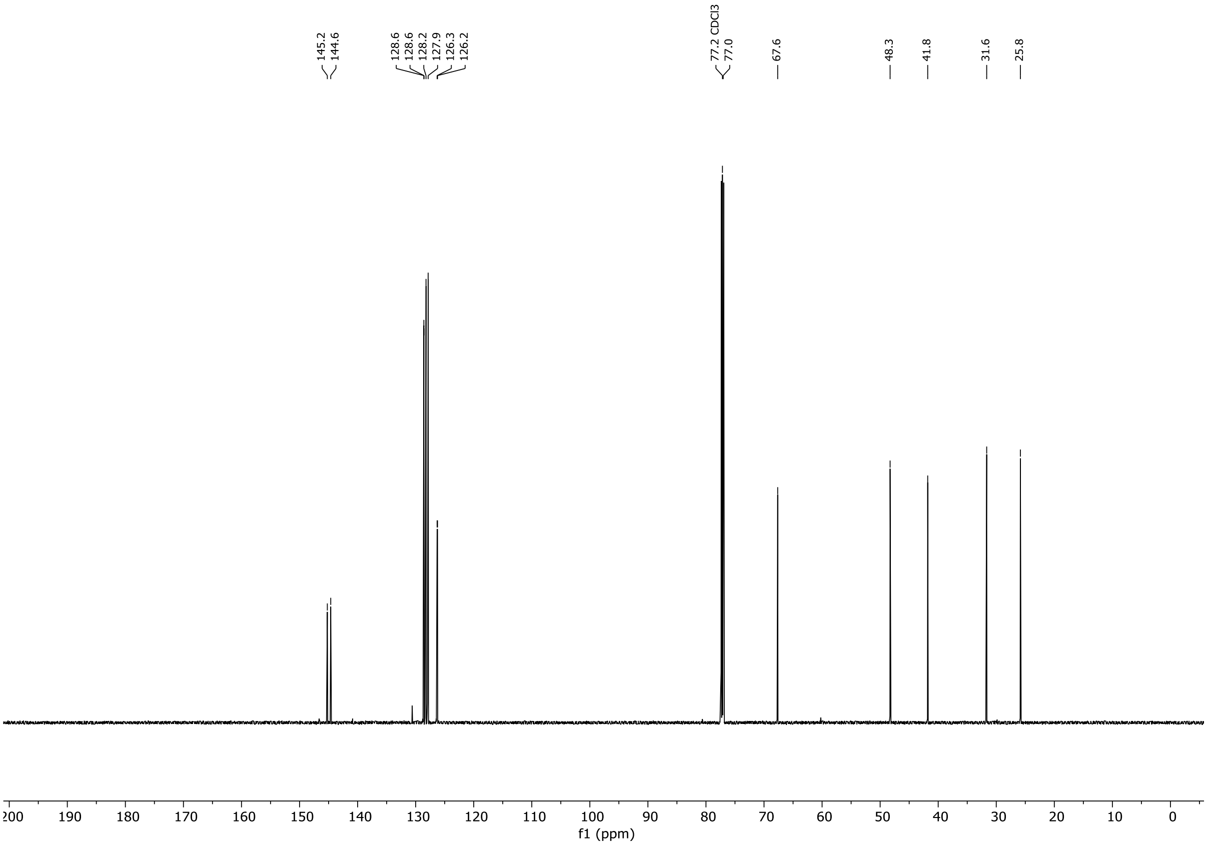

fenpiprane (7)

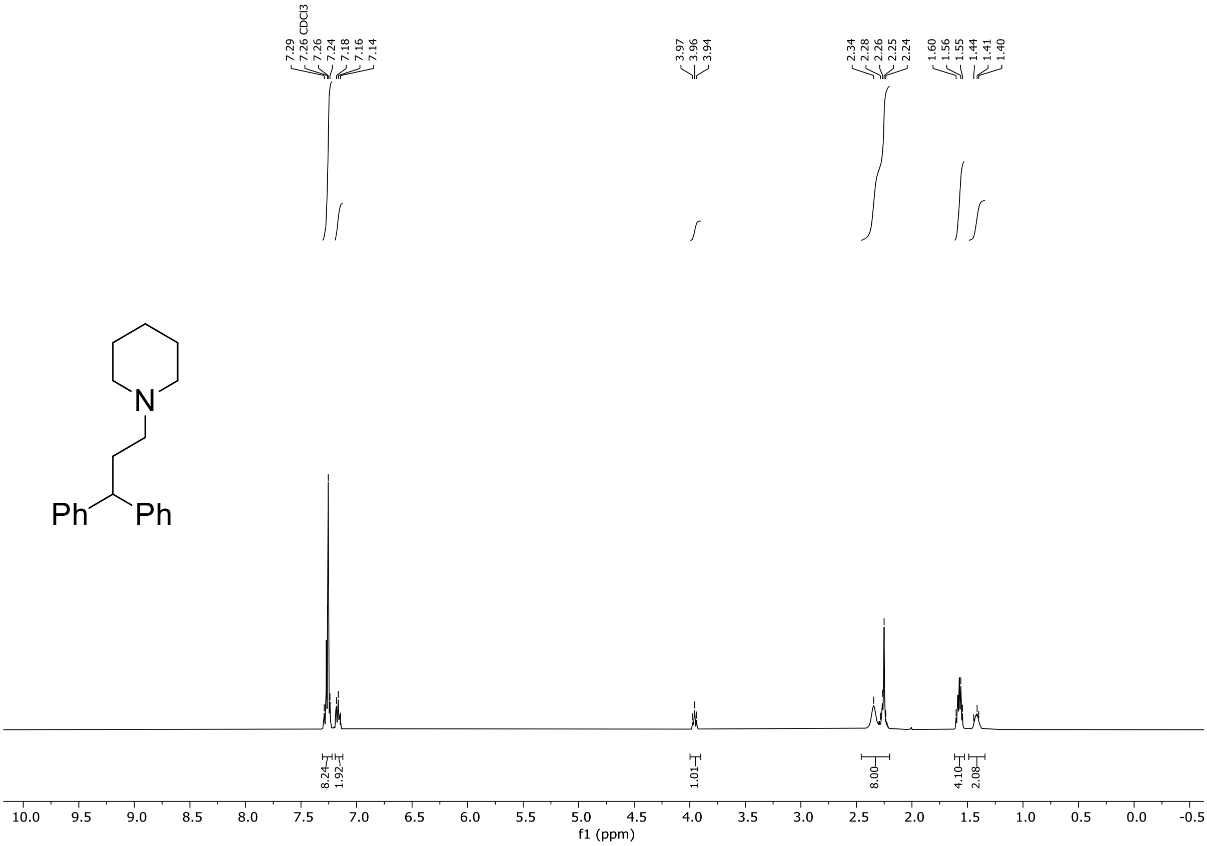

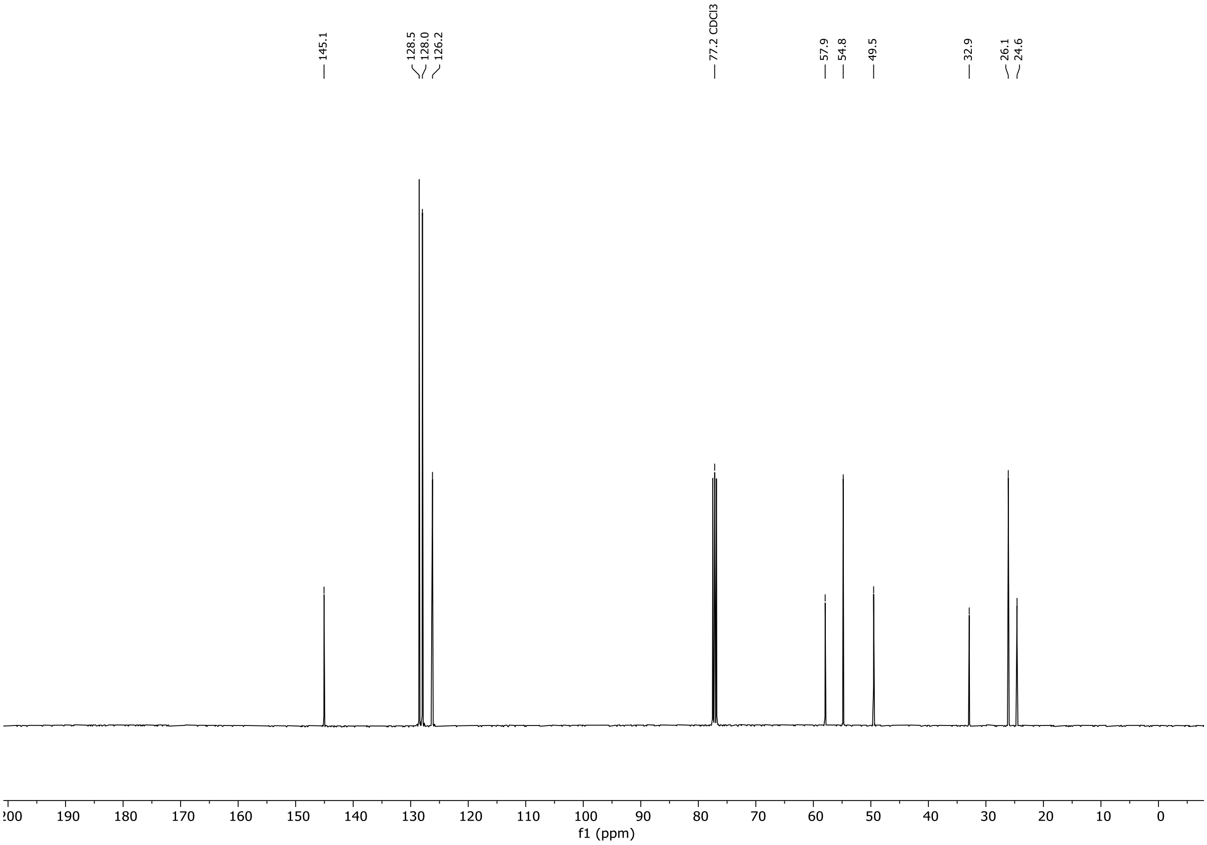

(((1*R*,2*S*)-2-methoxycyclohexyl)methylene)dibenzene (13o)

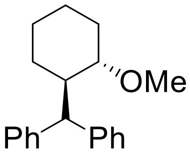

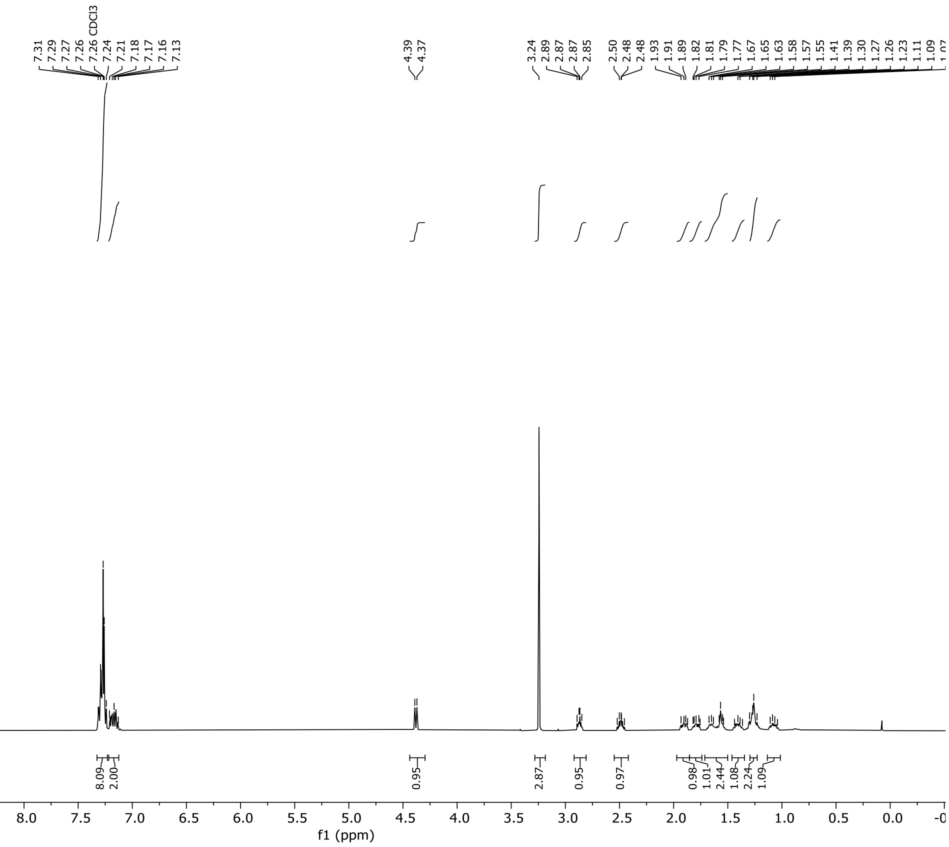

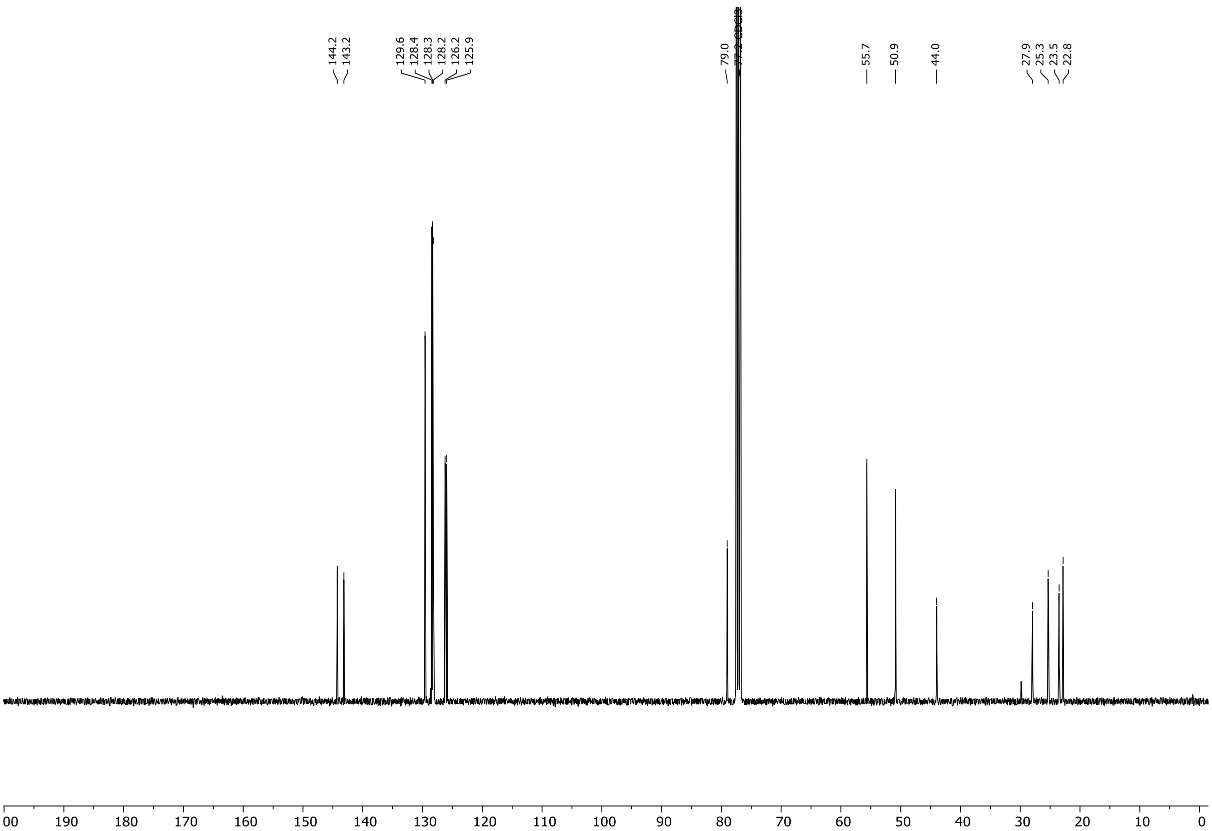

***cis*-4-(tert-butyl)cyclohexyl)methylene)dibenzene (13p)**

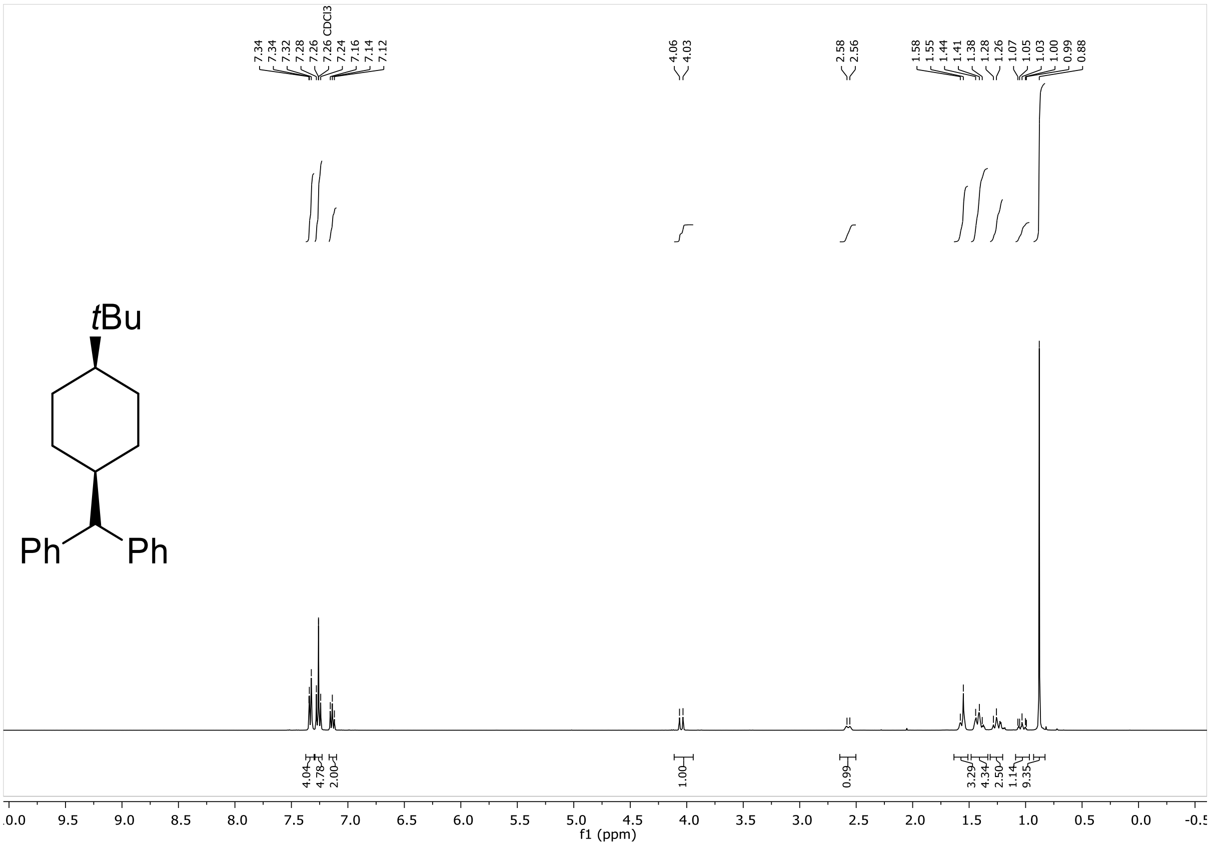

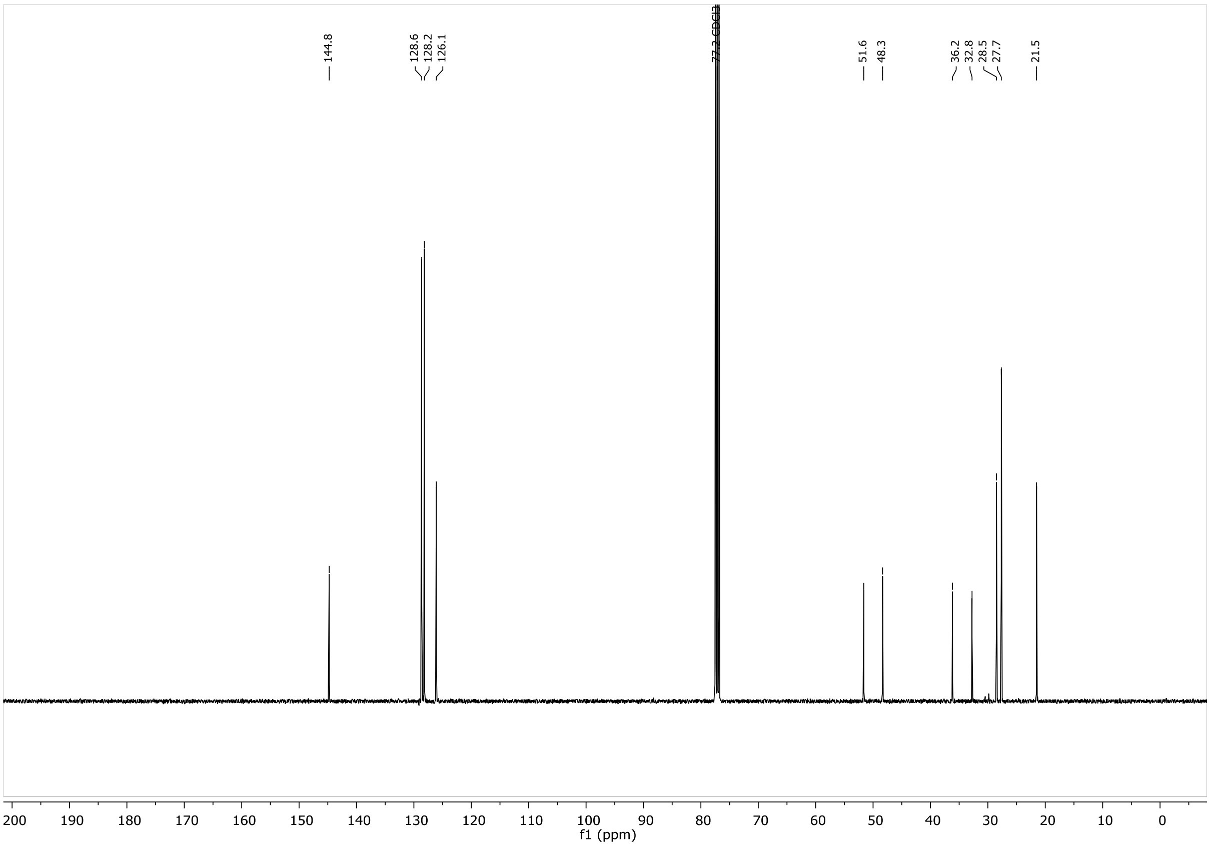

cis-4-benzhydrylcyclohexyl)oxy)(tert-butyl)dimethylsilane (13q)

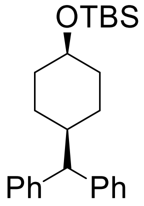

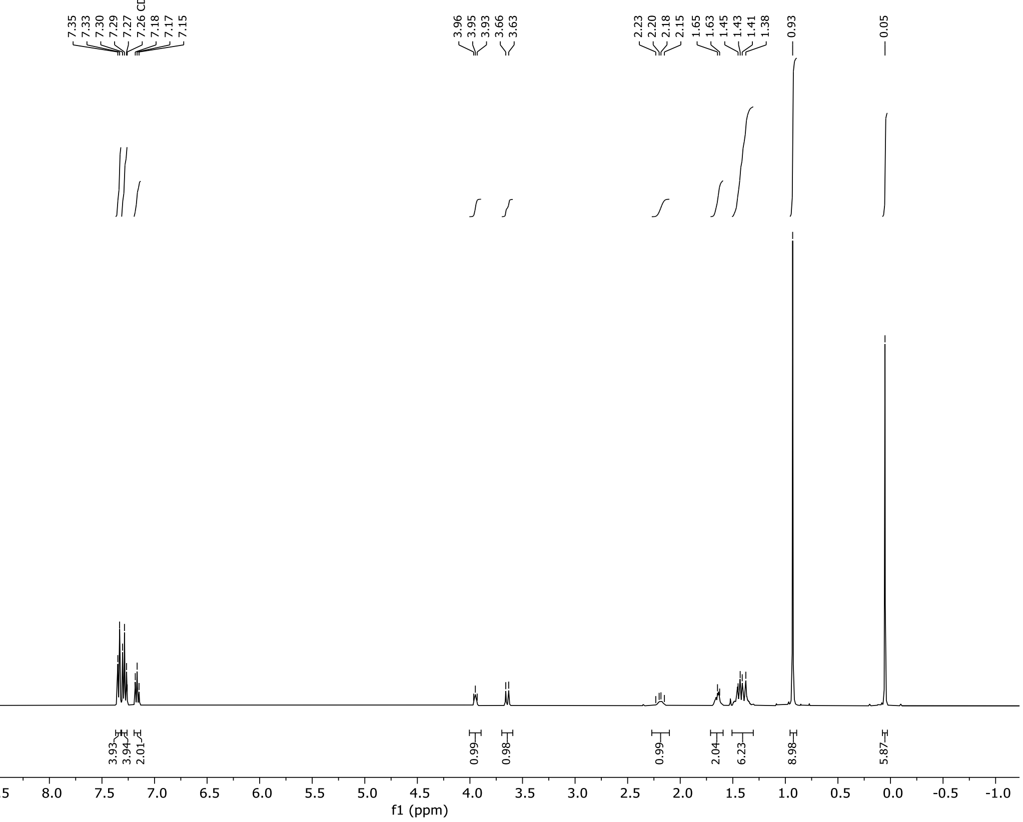

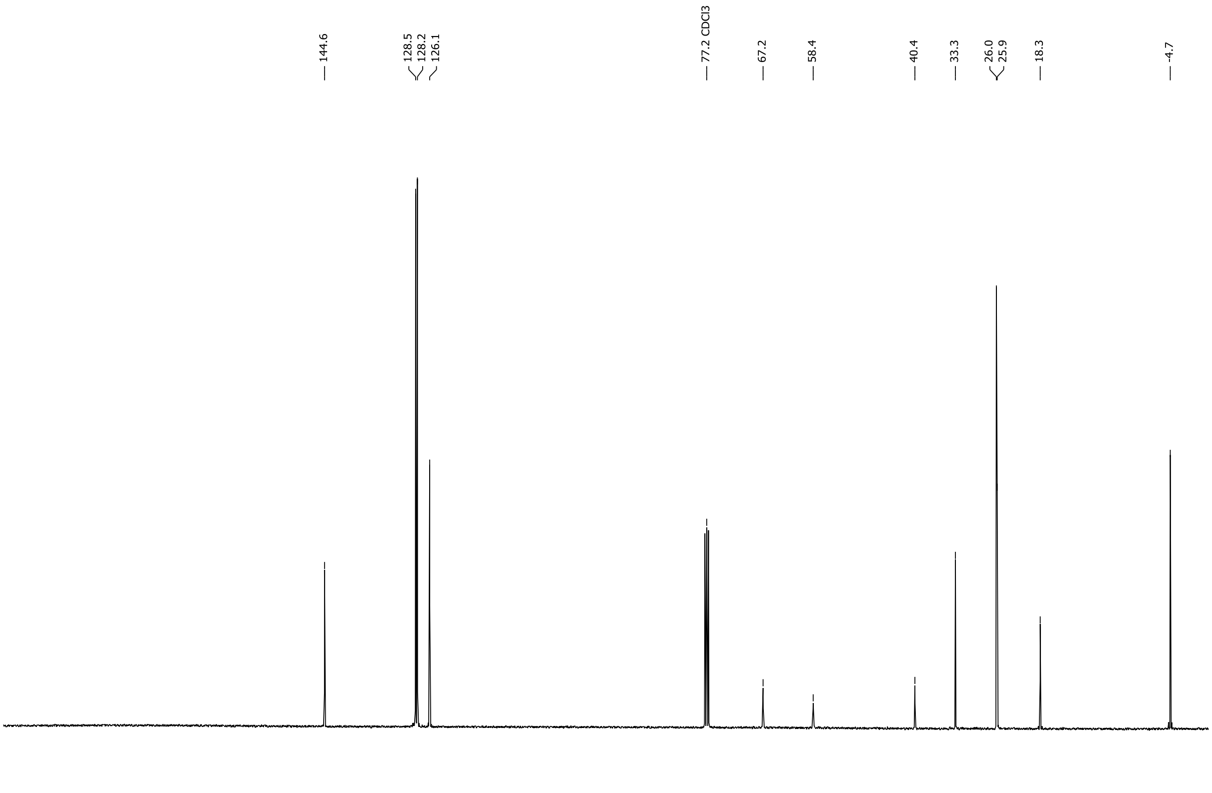

2-((5-methylthiazol-2-yl)methyl)adamantan-2-ol (15a)

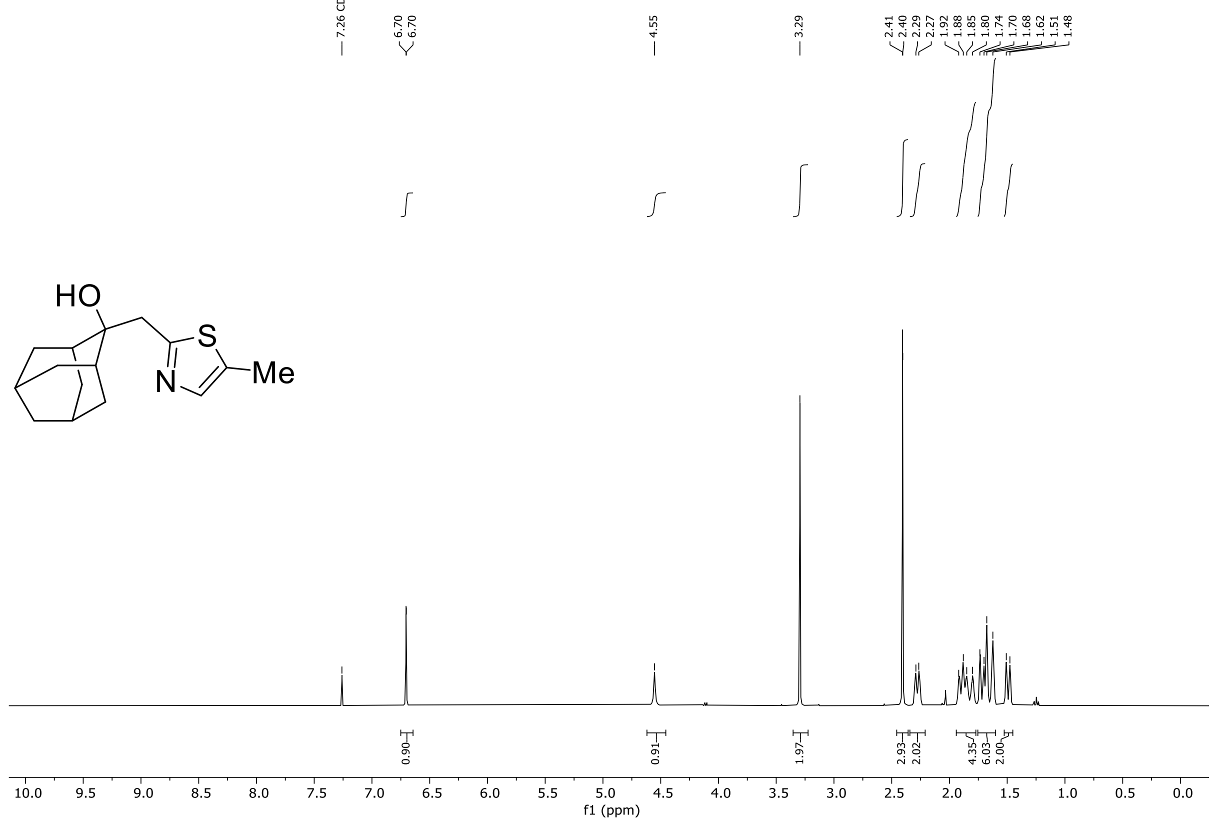

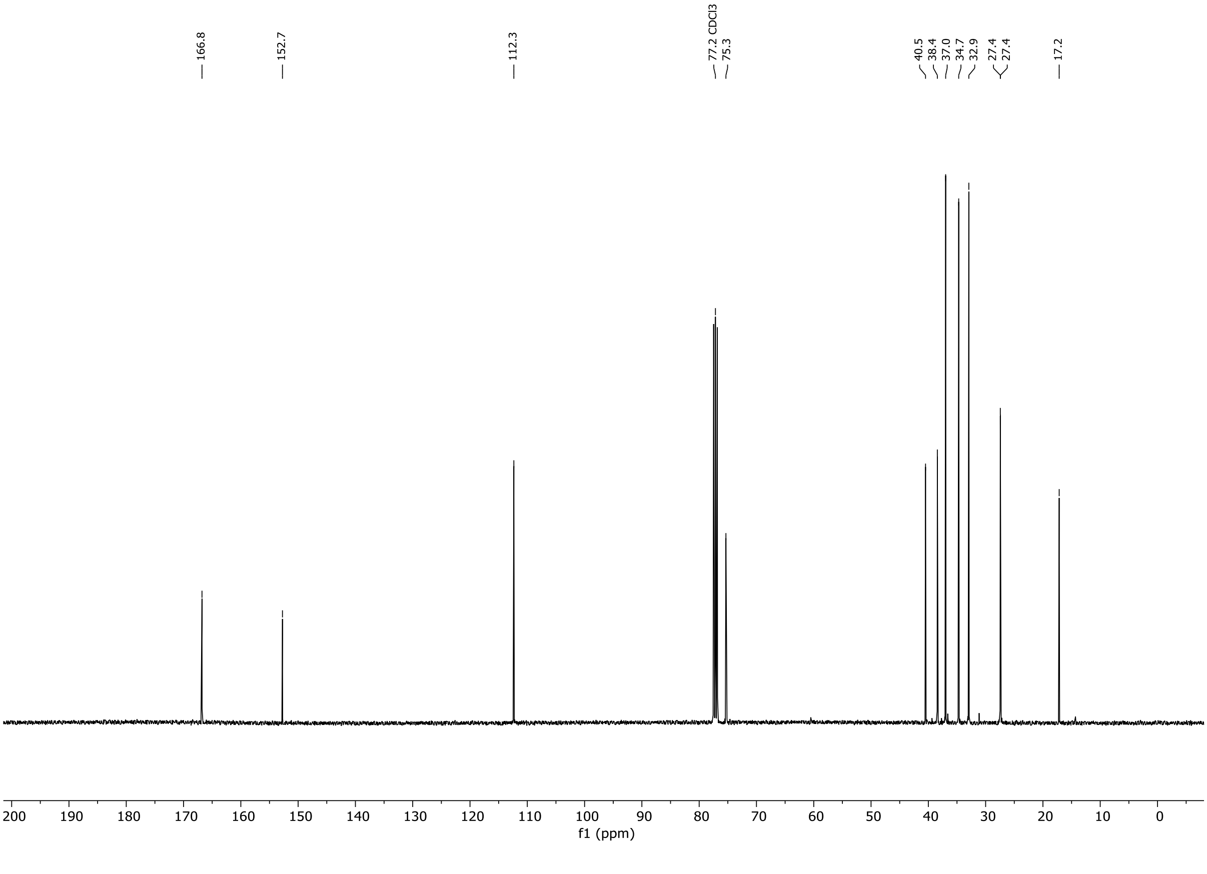

trans-2-((4-methylthiazol-2-yl)methyl)cyclohexan-1-ol (15b)

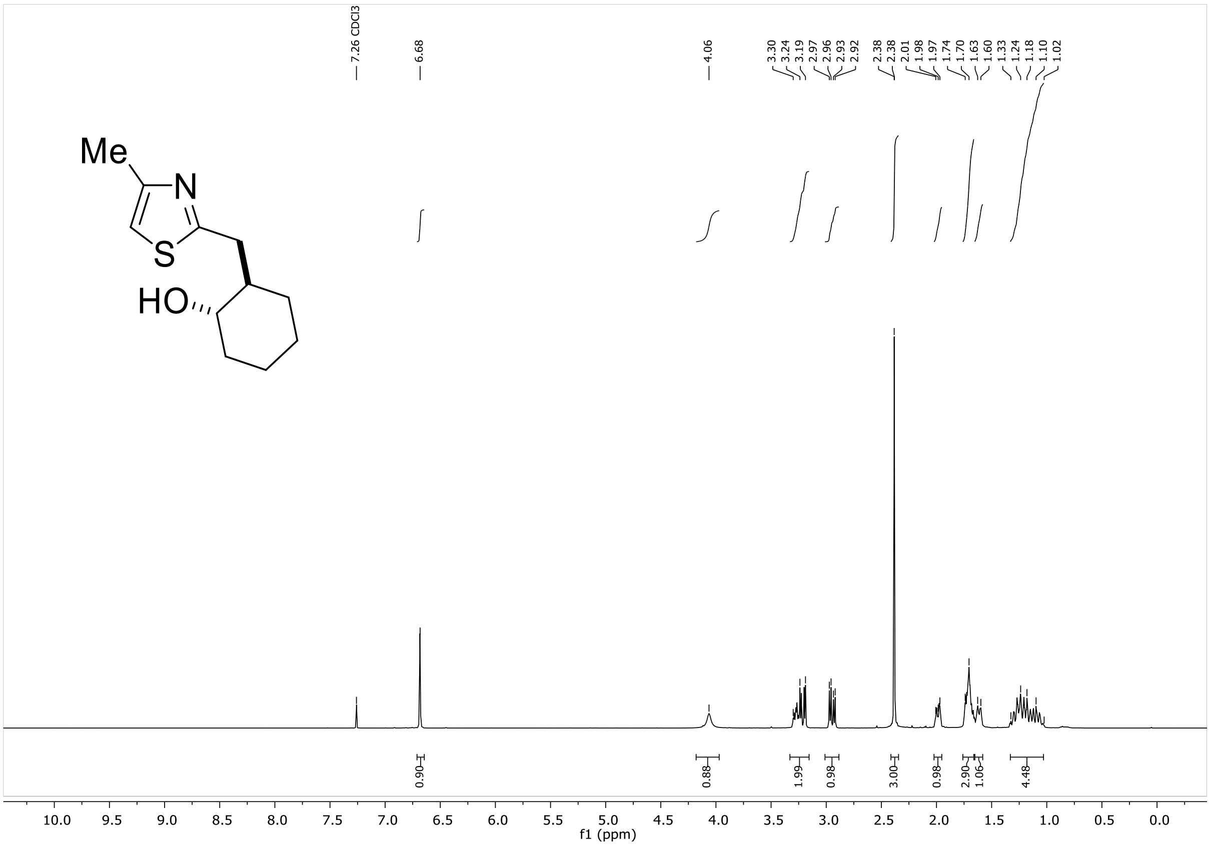

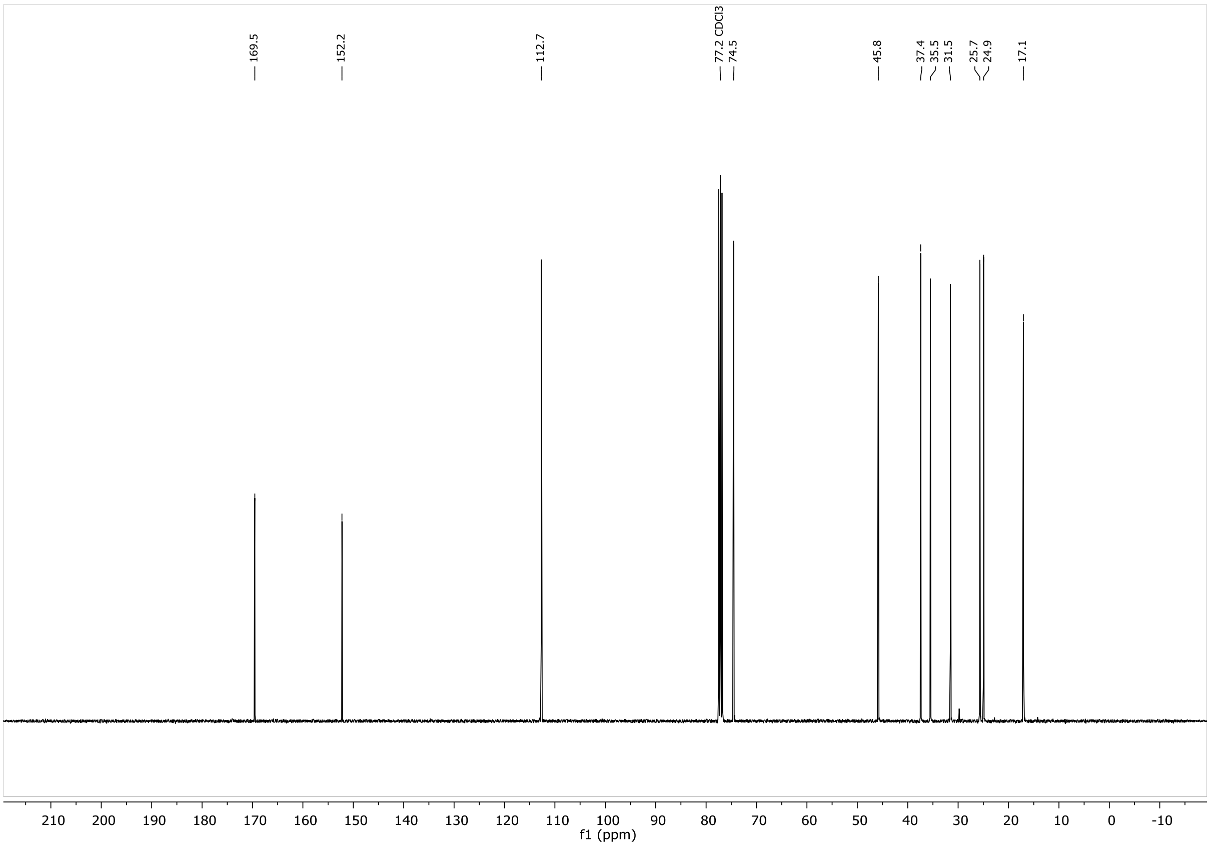

(*S*)-1-(benzyloxy)-4-(4-methylthiazol-2-yl)butan-2-ol (15c)

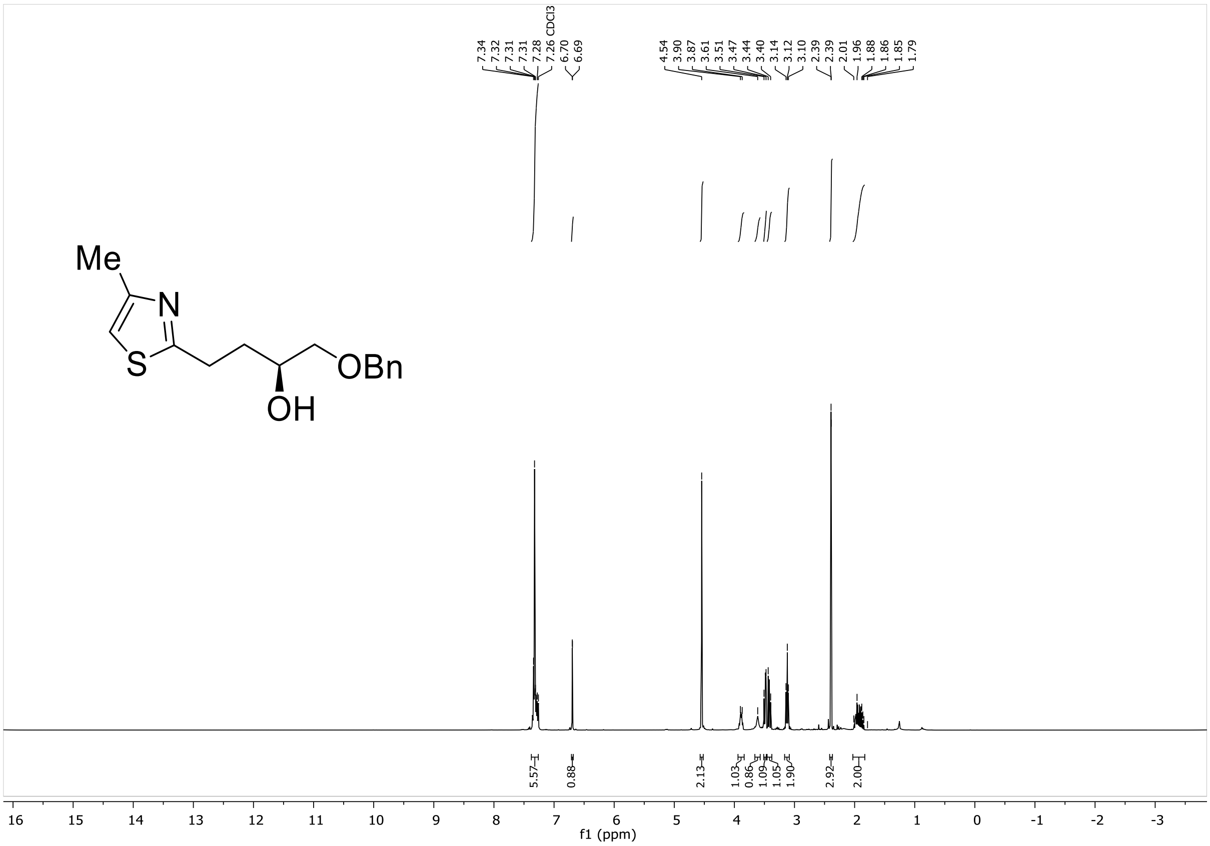

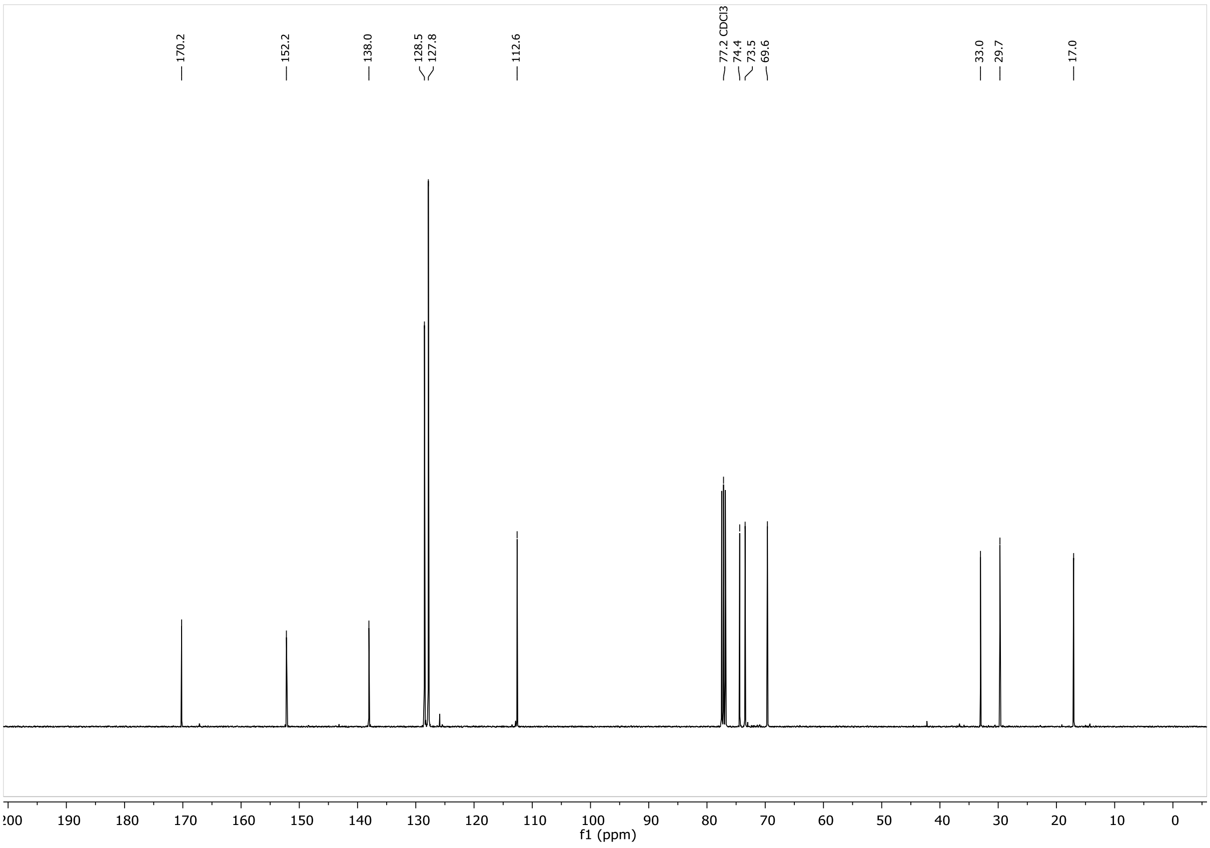

2-(2-isopropyl-4-methylthiazol-5-yl)adamantan-2-ol (17a)

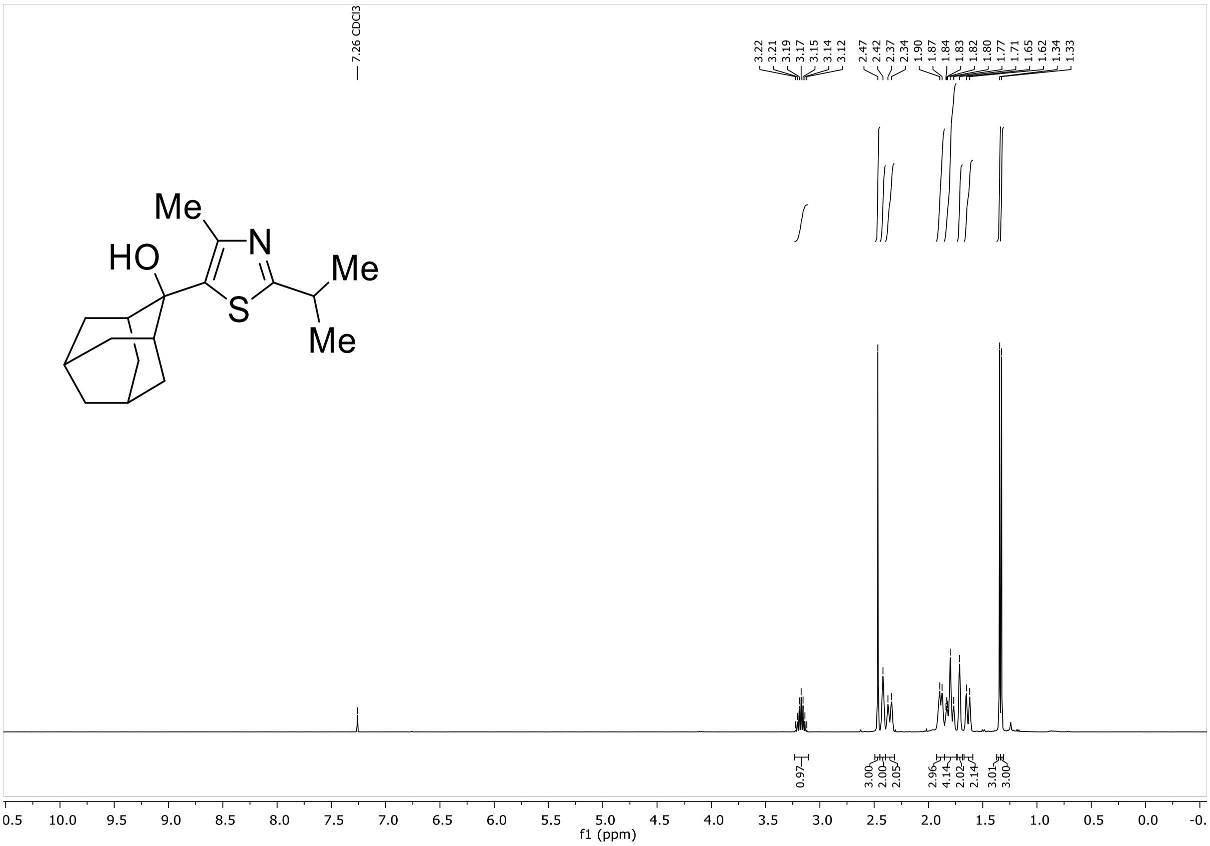

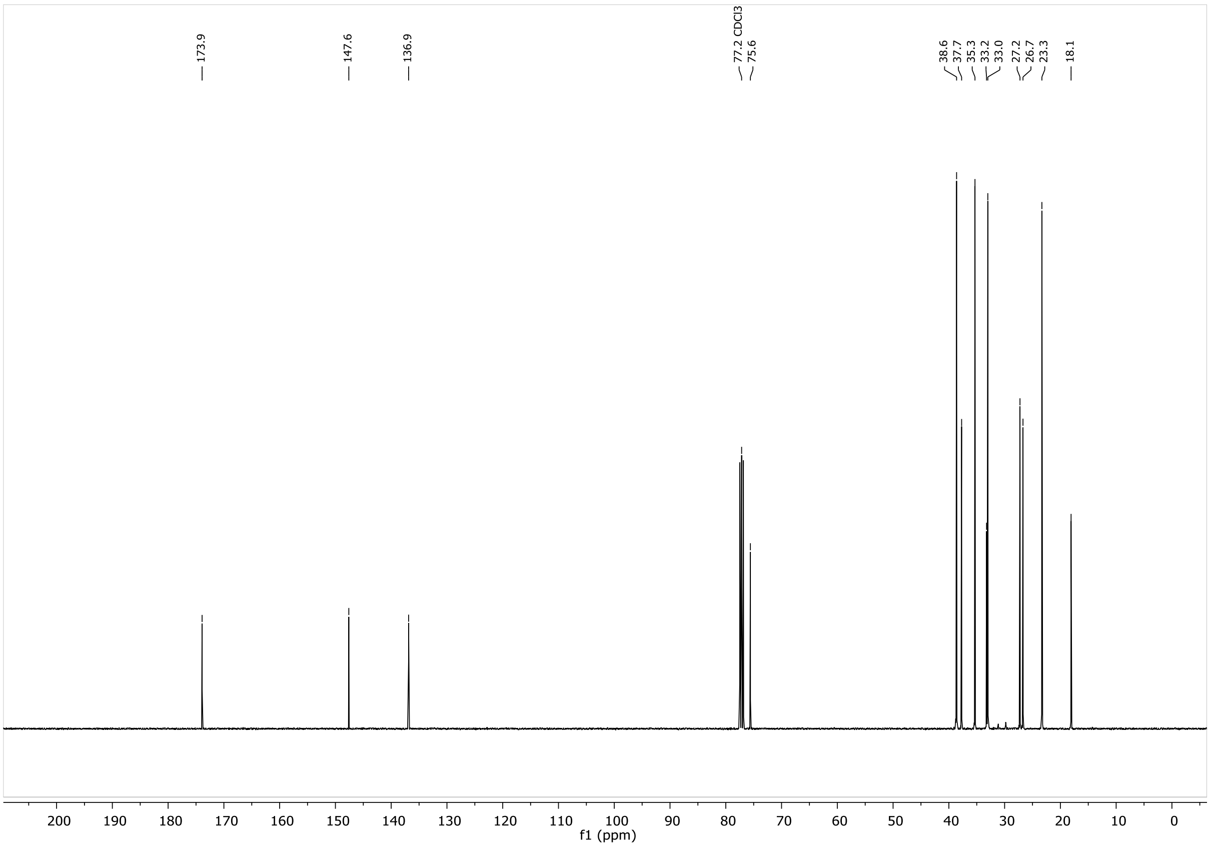

***trans*-2-(2-(4-methylthiazol-2-yl)propan-2-yl)cyclohexan-1-ol (19a)**

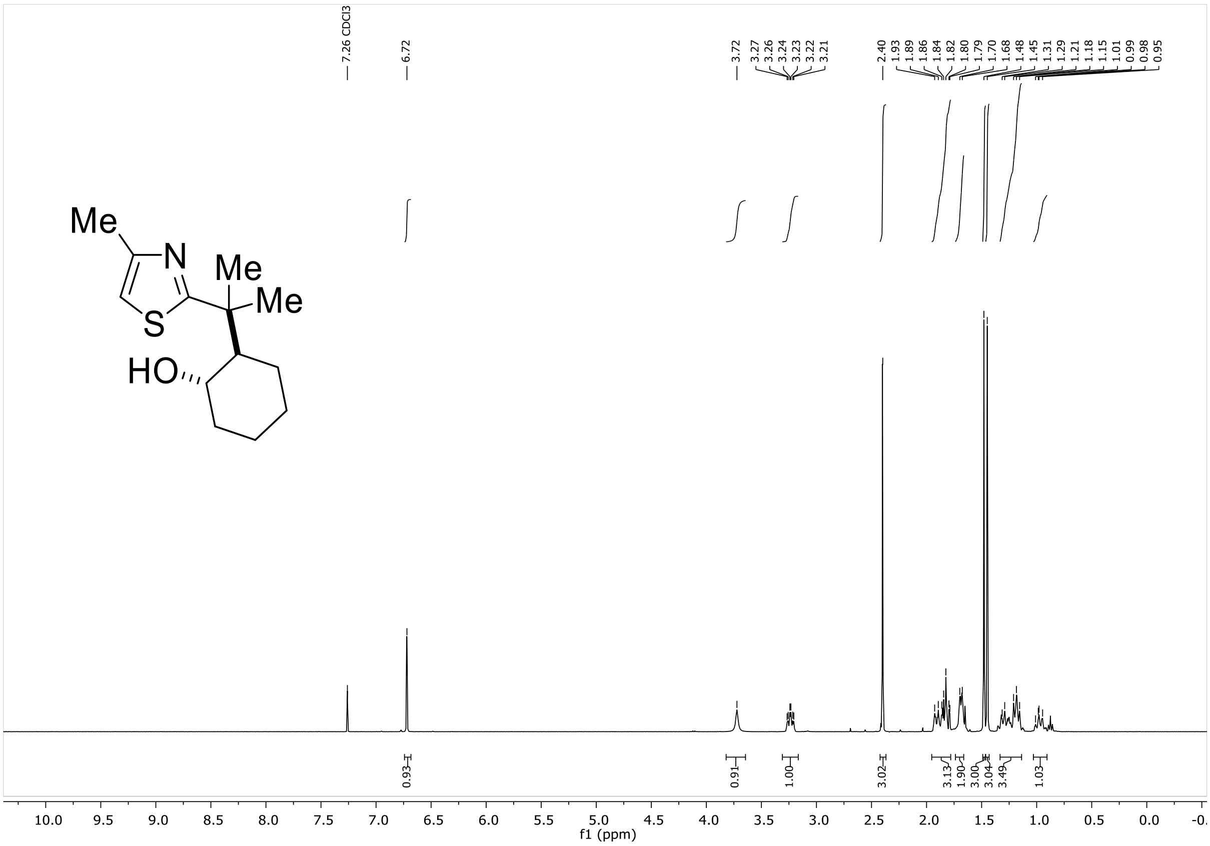

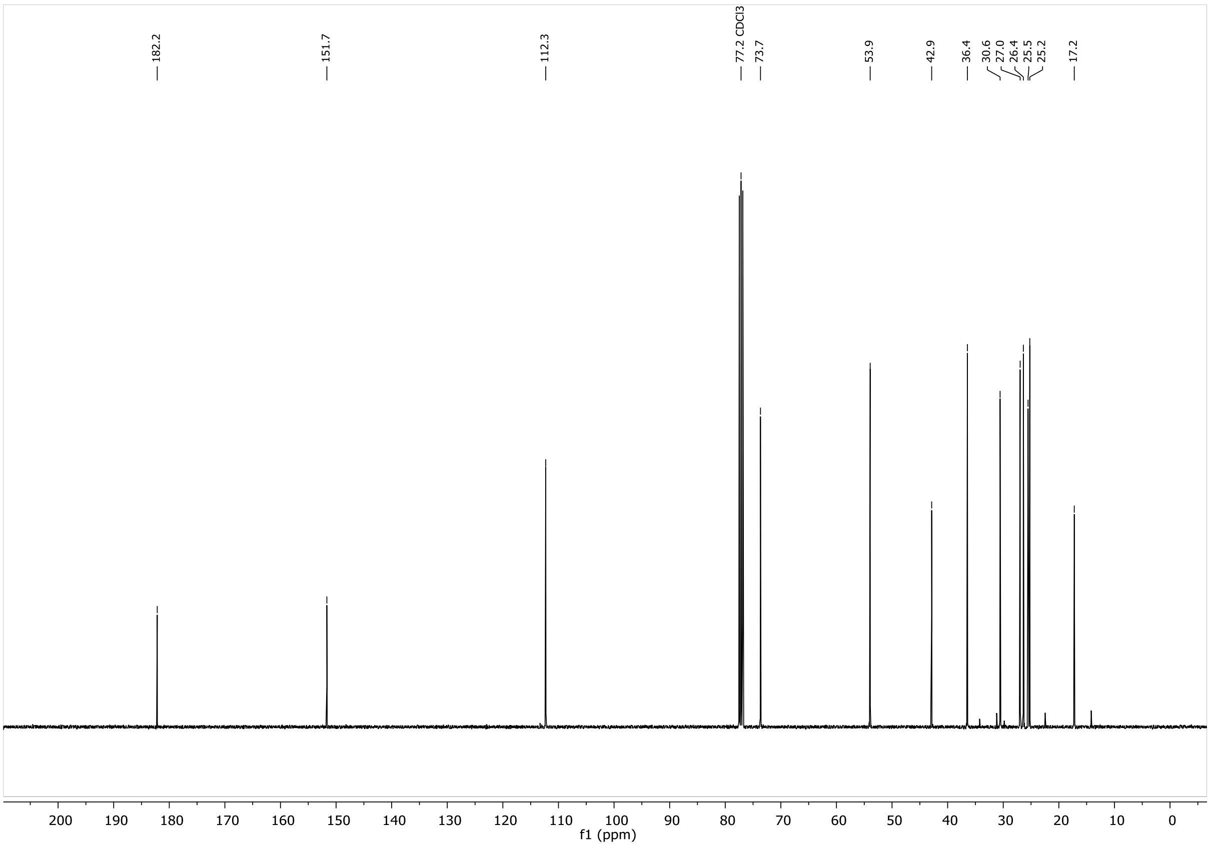

4-methyl-2-(2-methyltetradecan-2-yl)thiazole (19b)

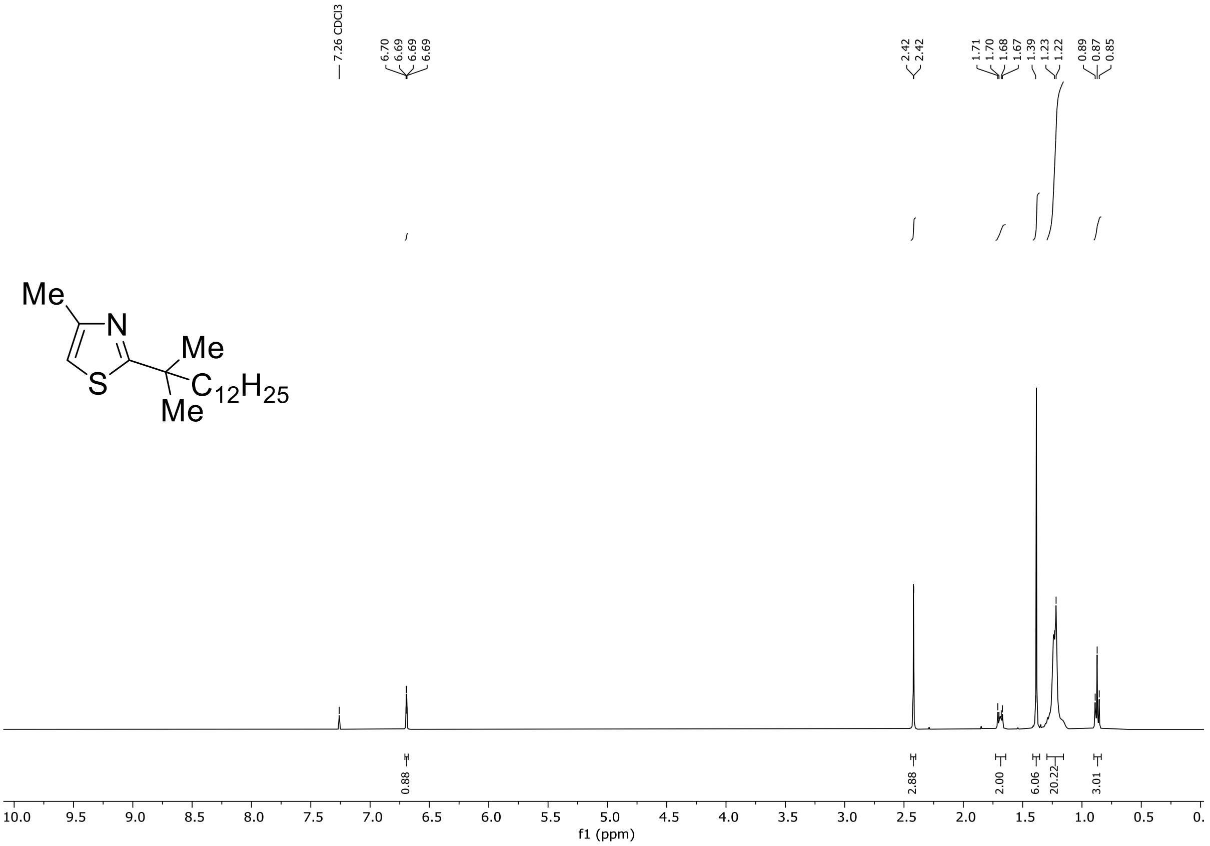

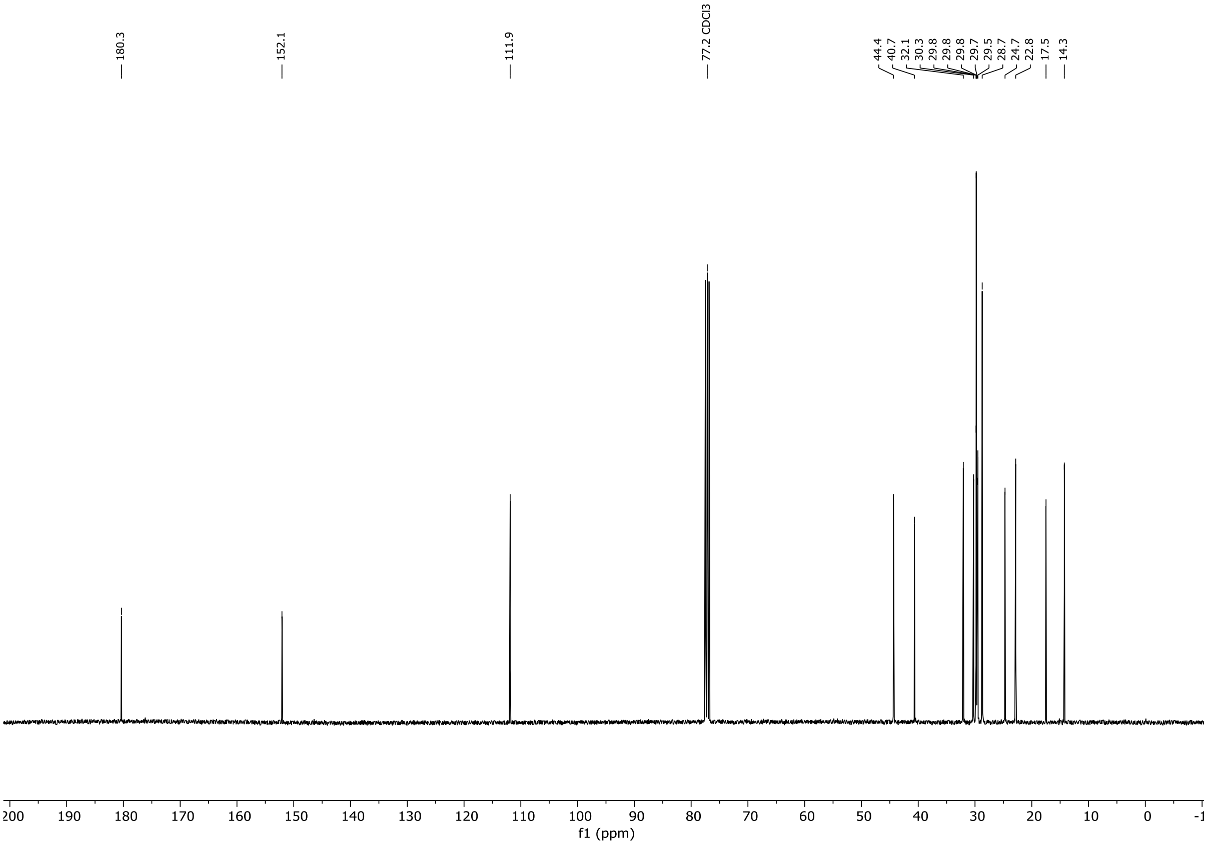

2-(pyridin-3-ylmethyl)adamantan-2-ol (22a)

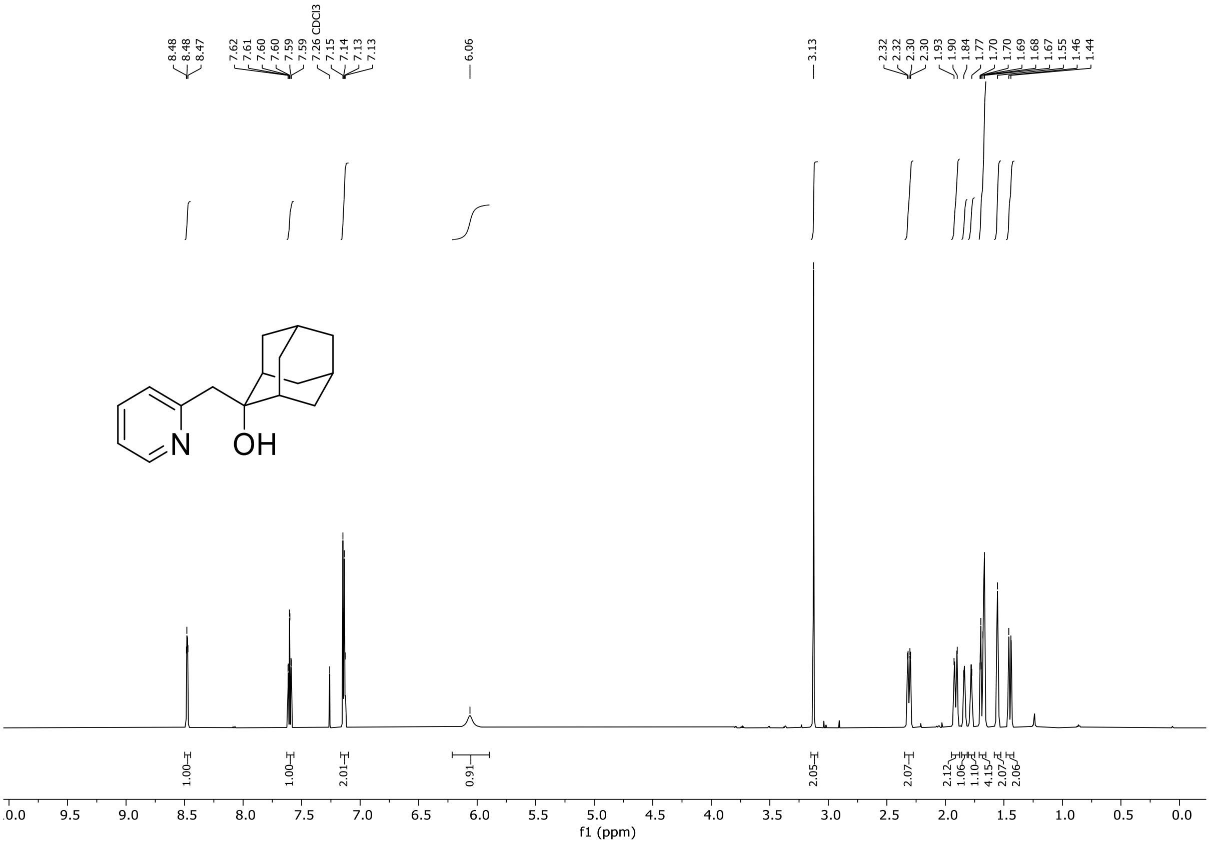

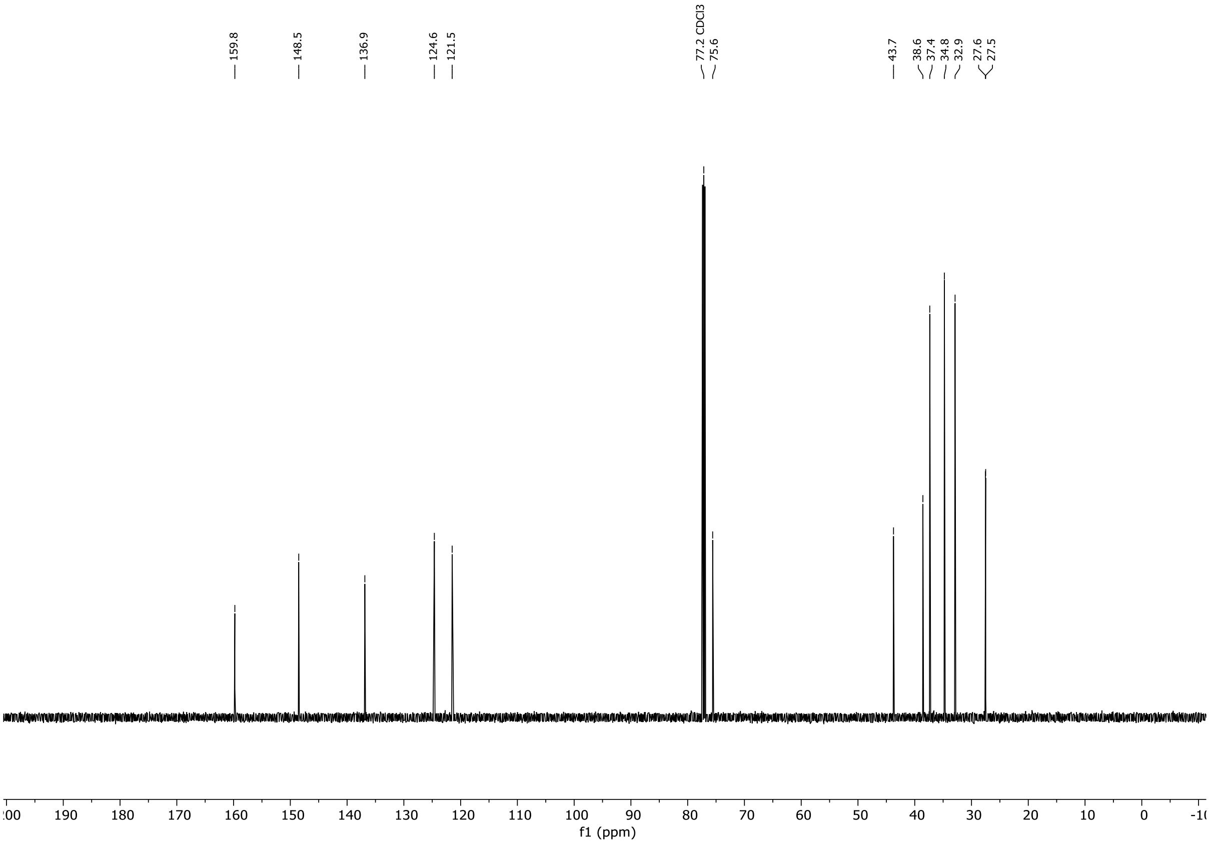

1,1-diphenyl-2-(pyridin-2-yl)ethan-1-ol (22b)

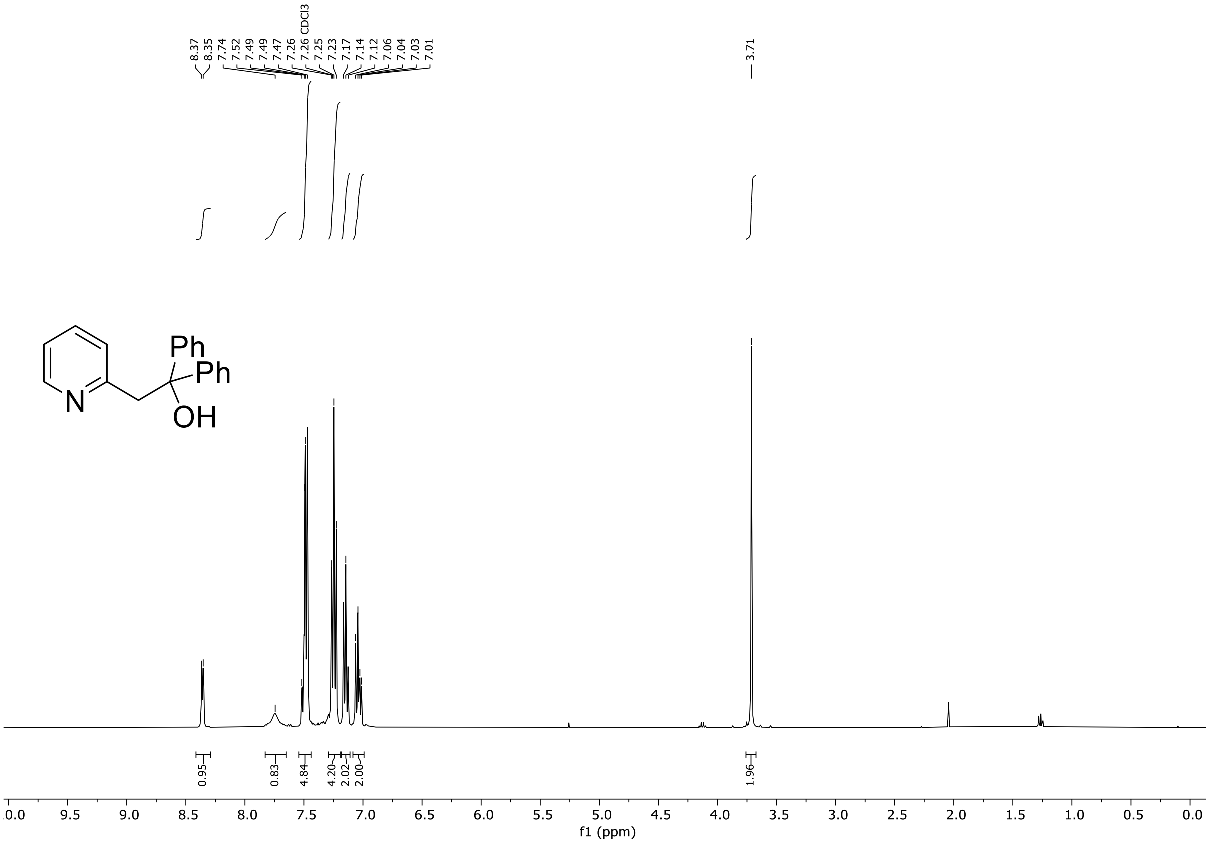

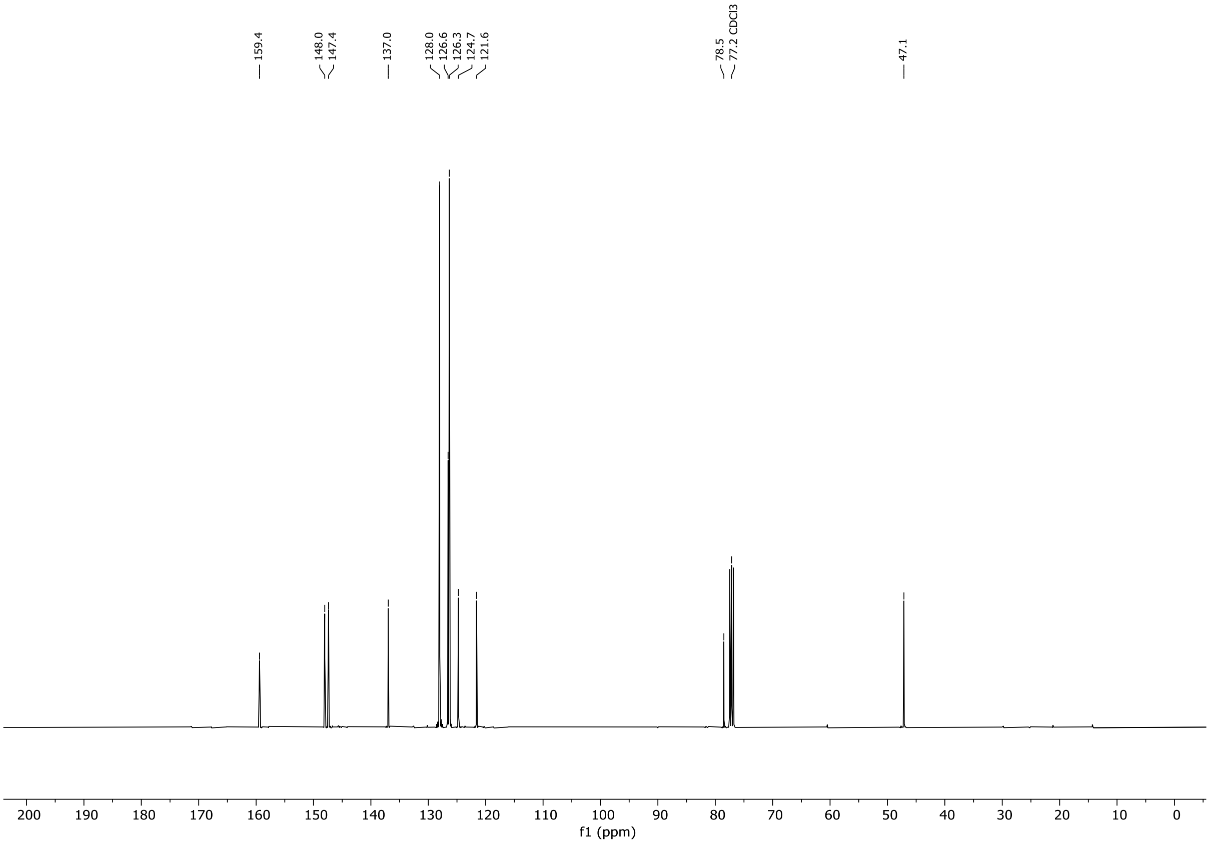

1,1-diphenyl-2-(pyridin-4-yl)ethan-1-ol (22c)

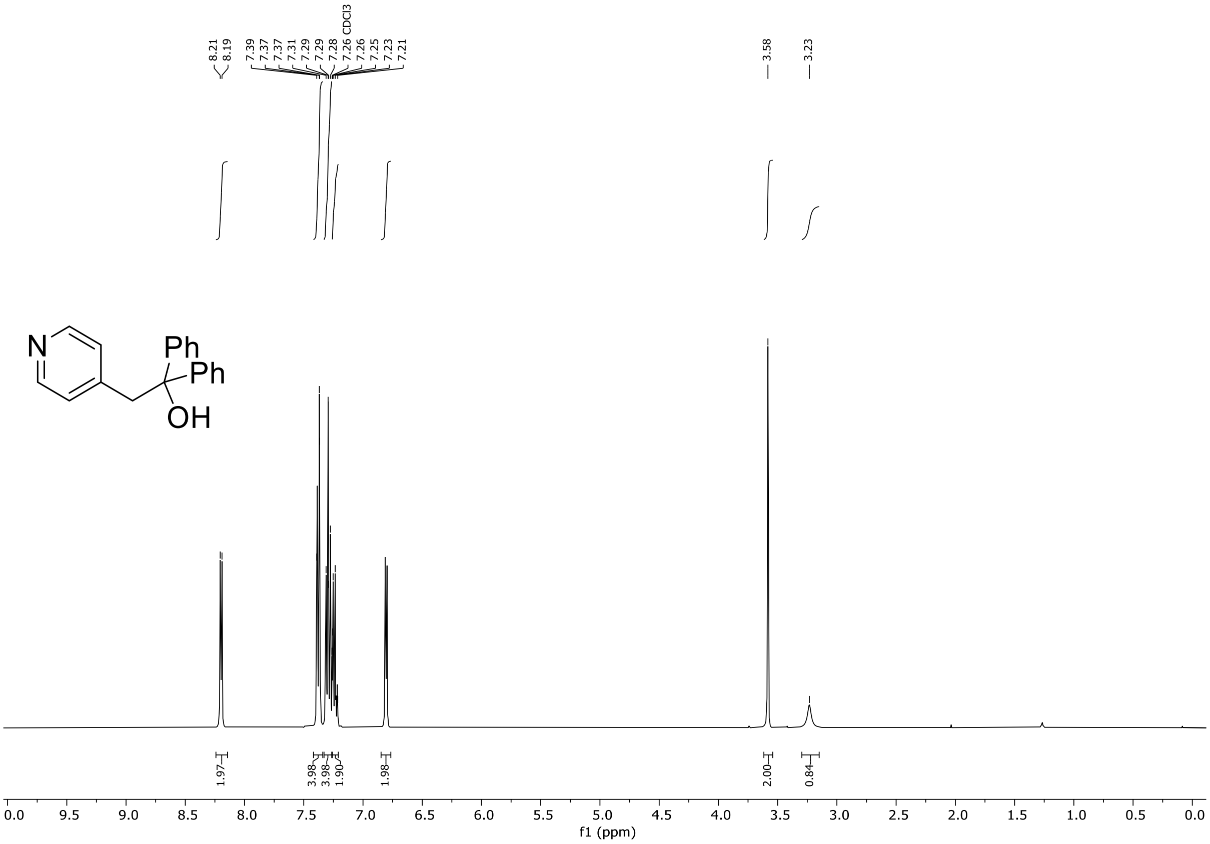

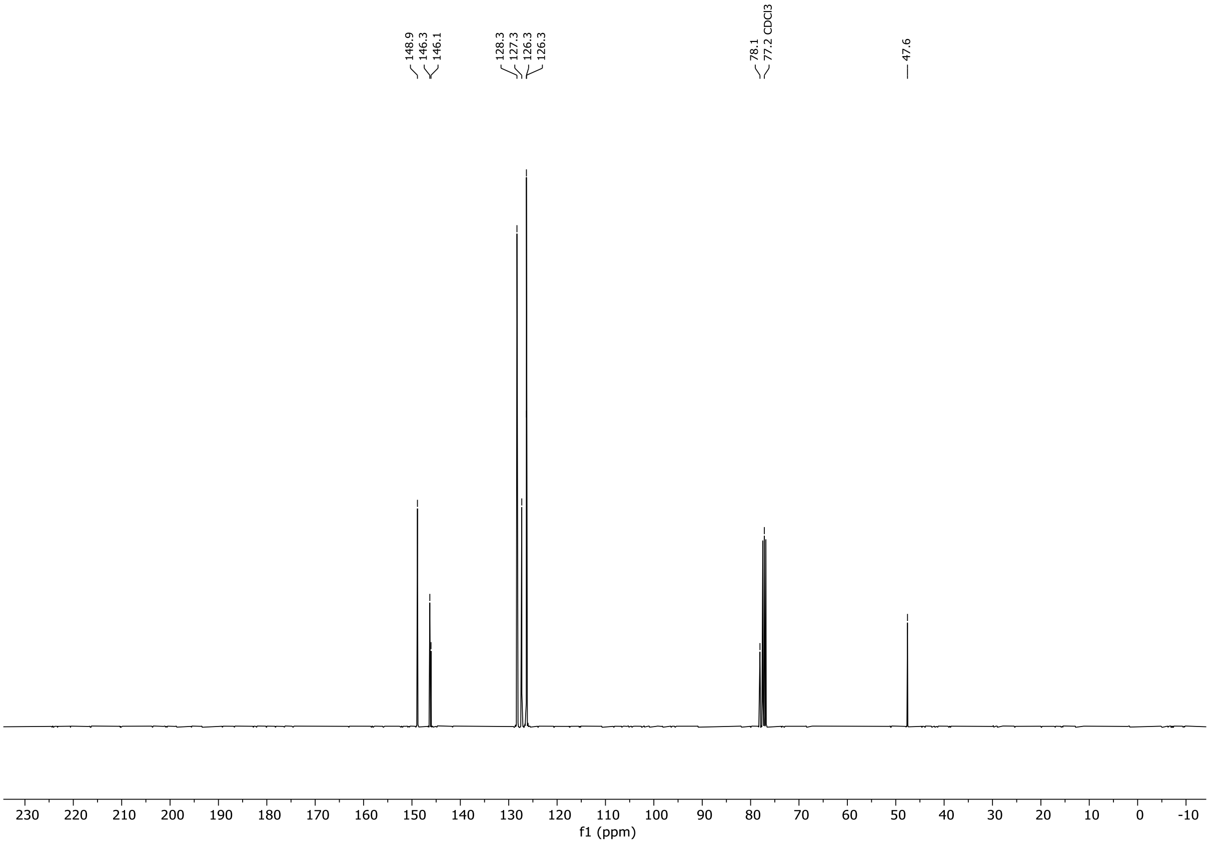

4-(4-phenylbutyl)pyridine (22d)

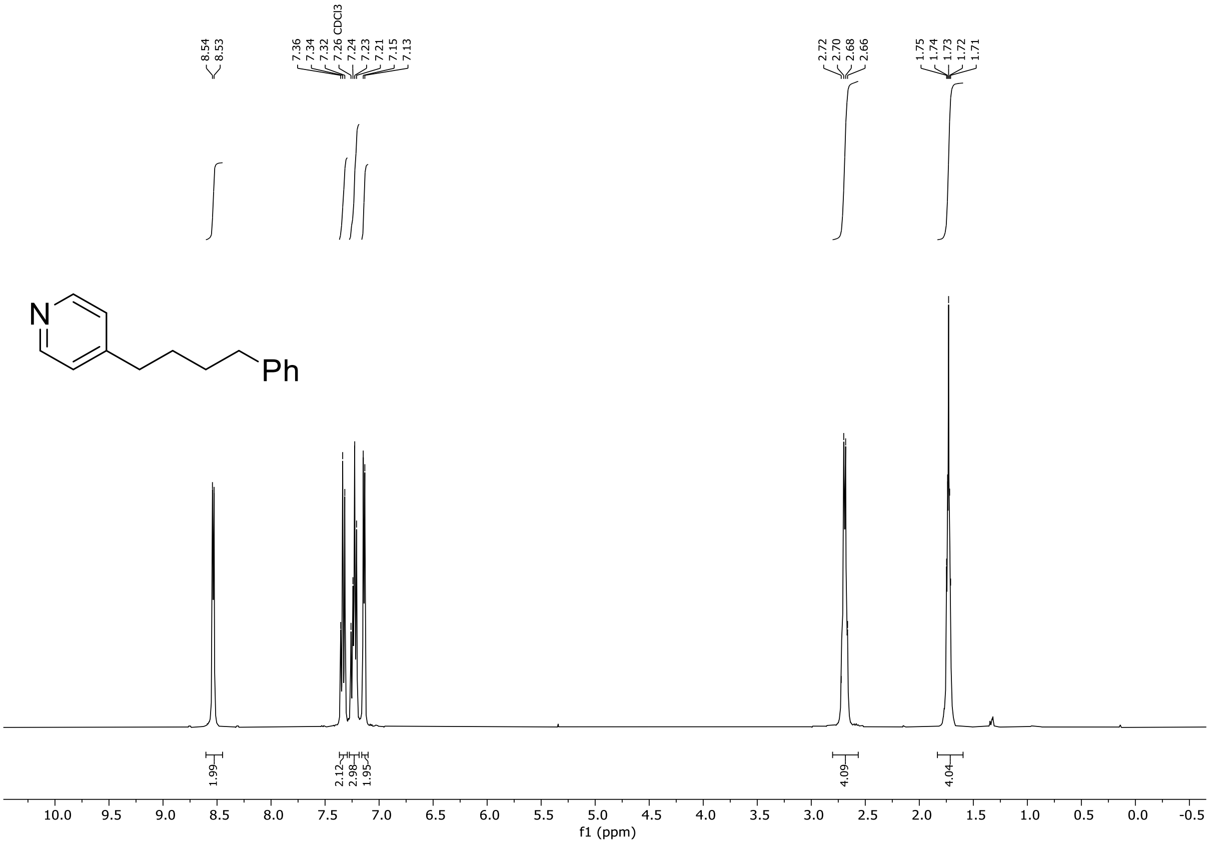

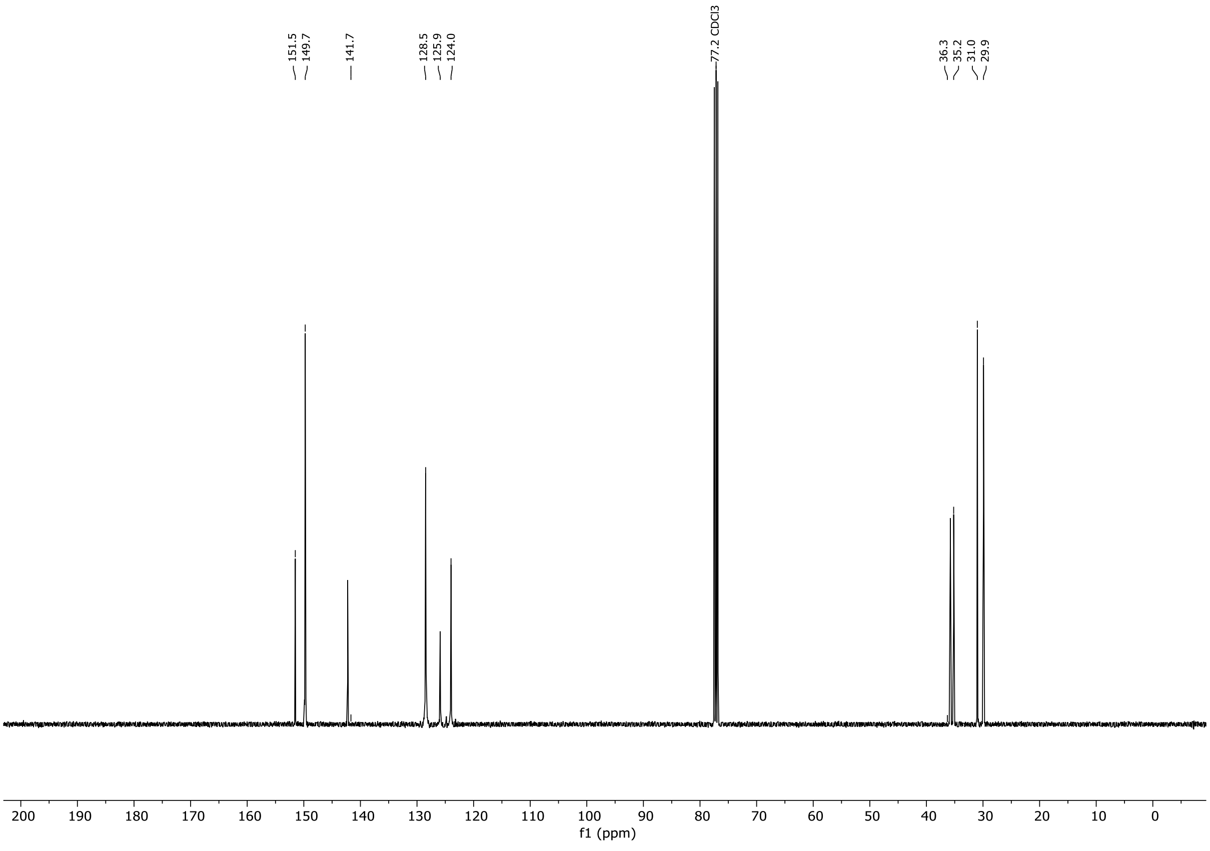

(2*S*)-1-(benzyloxy)-4-phenyl-4-(pyridin-2-yl)butan-2-ol (22e) mixture of diastereoisomers

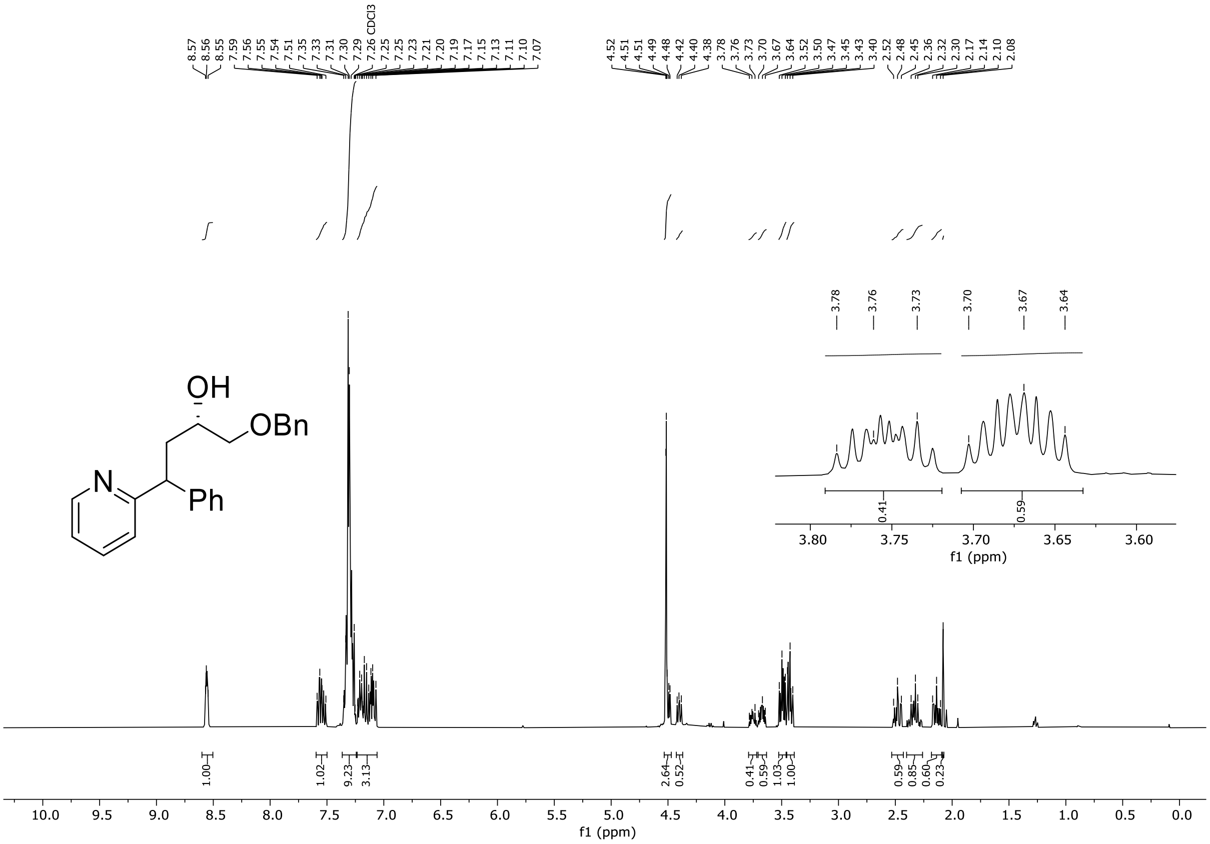

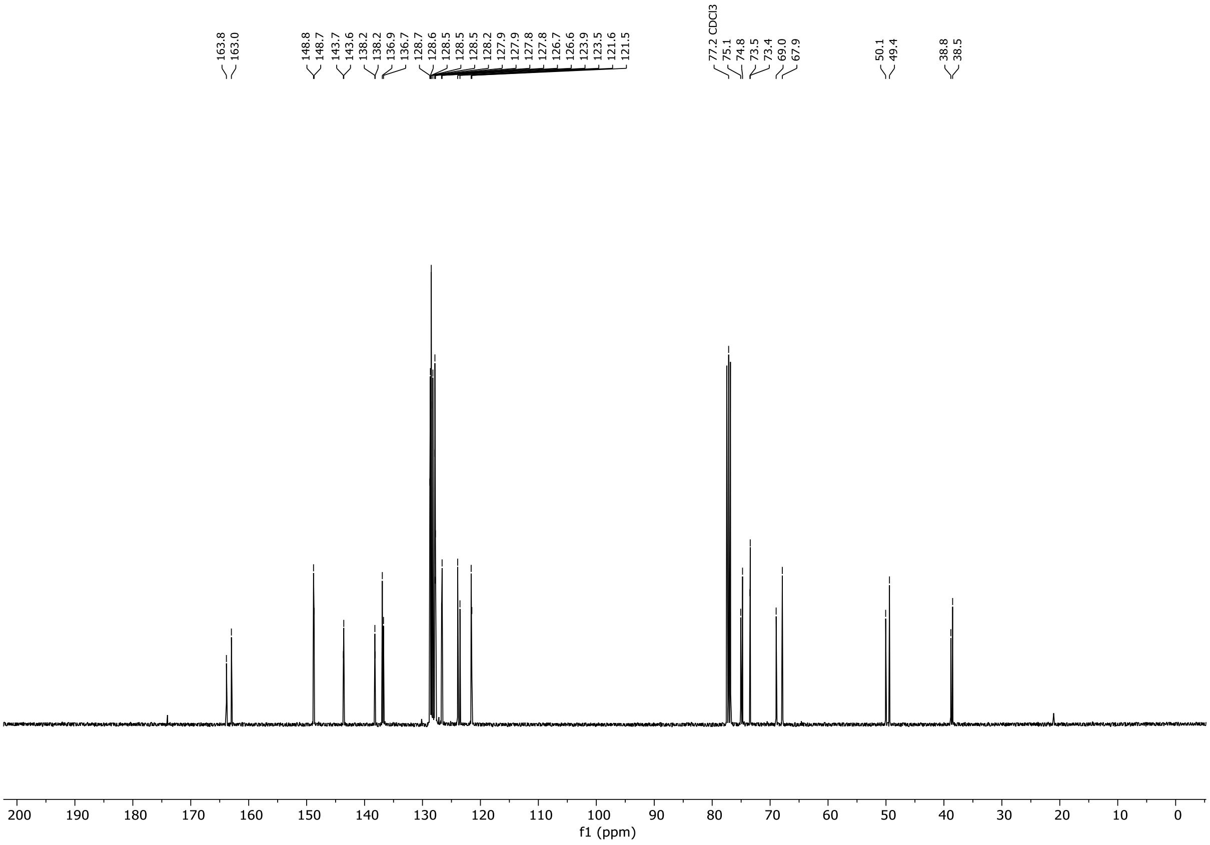

2-(1,4-diphenylbutyl)pyridine (22f)

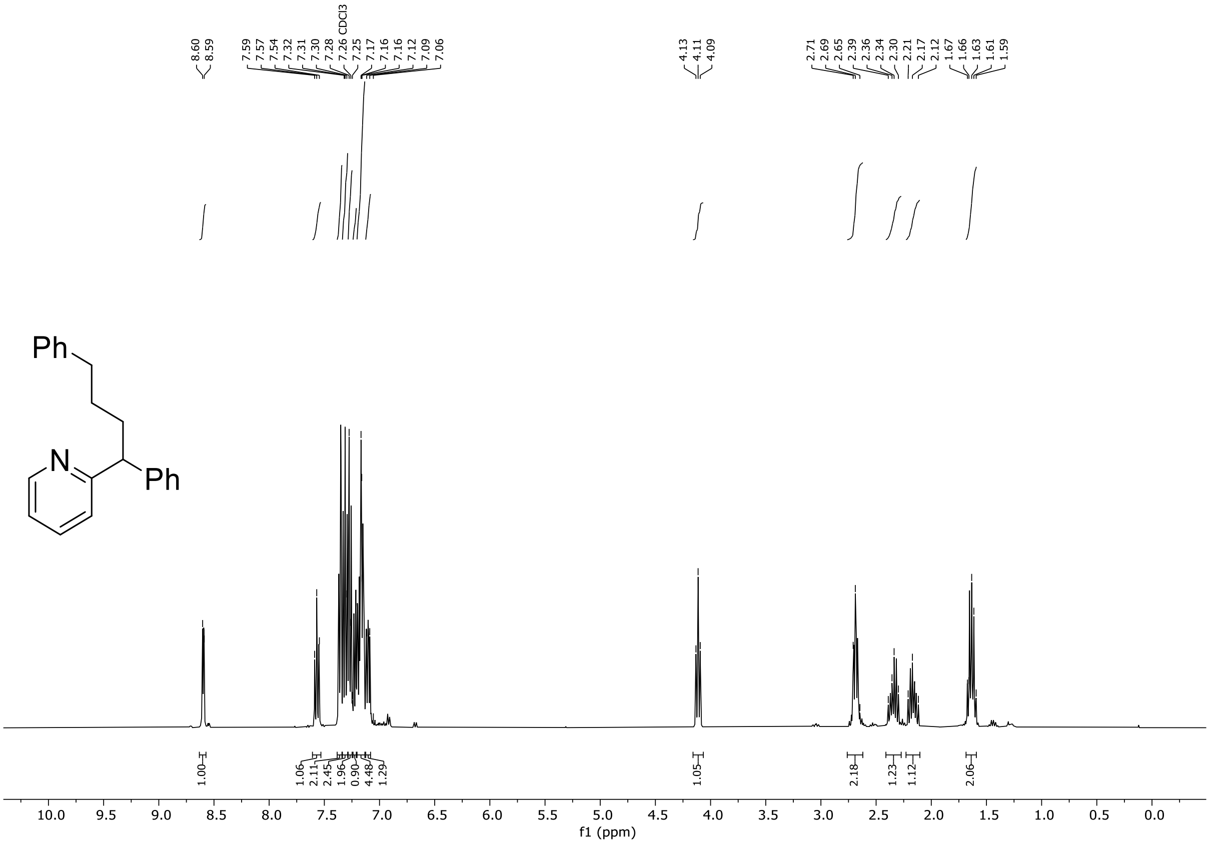

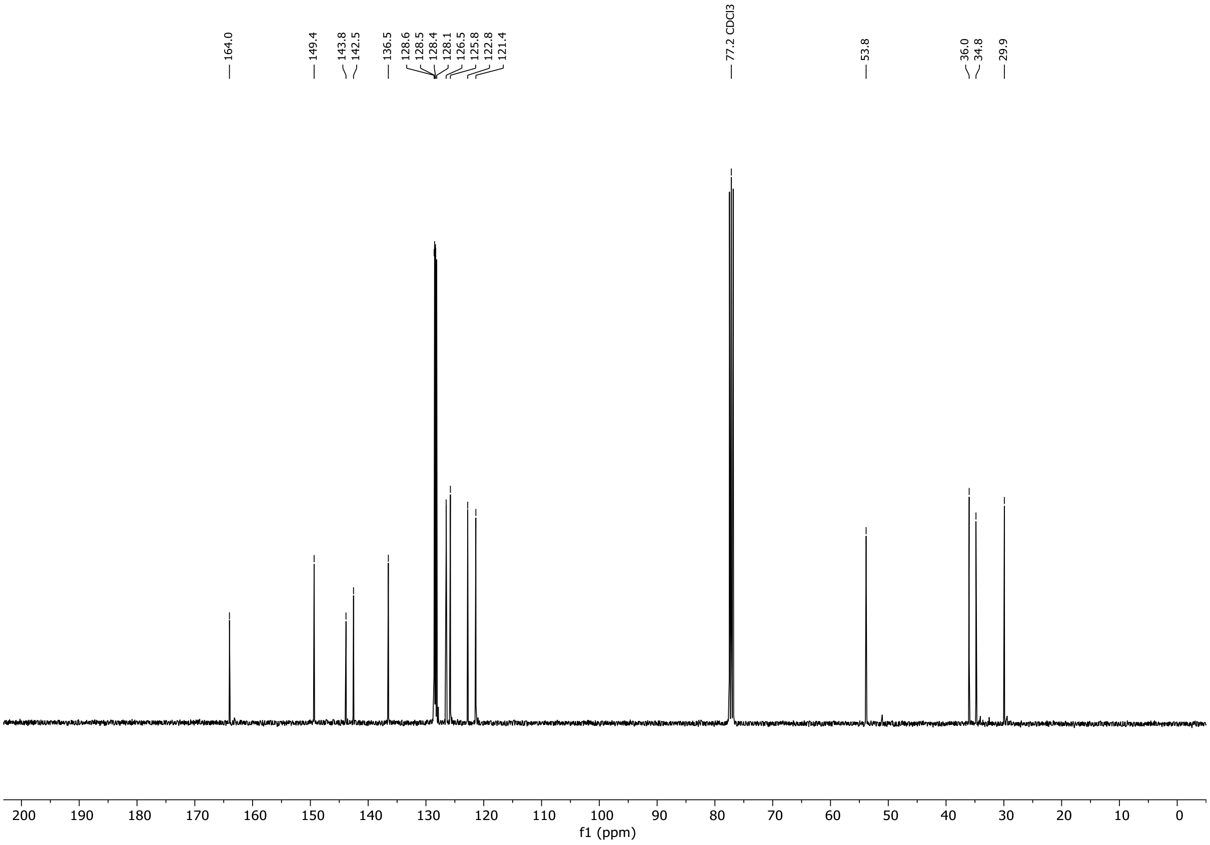

tert-butyl 6,6-dimethyl-5,7-dioxa-1-azaspiro[2.5]octane-1-carboxylate (25)

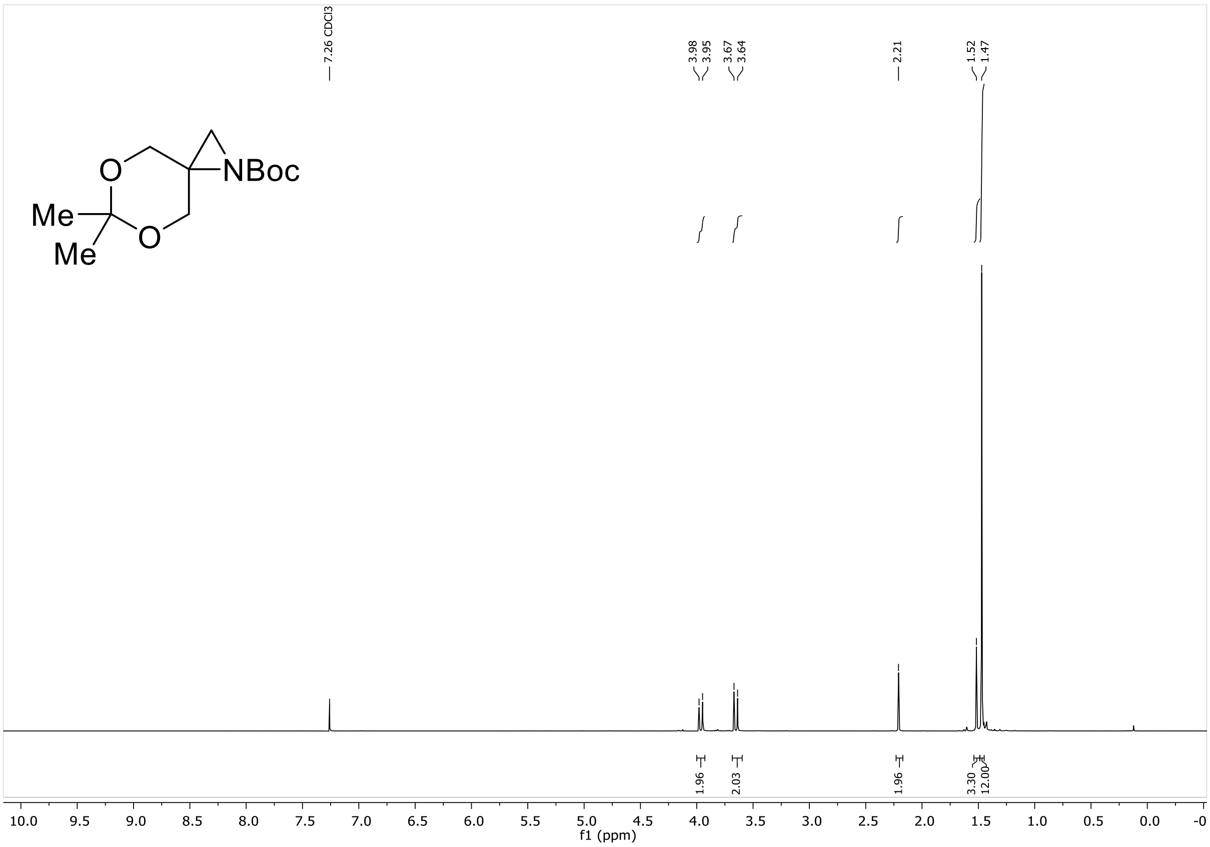

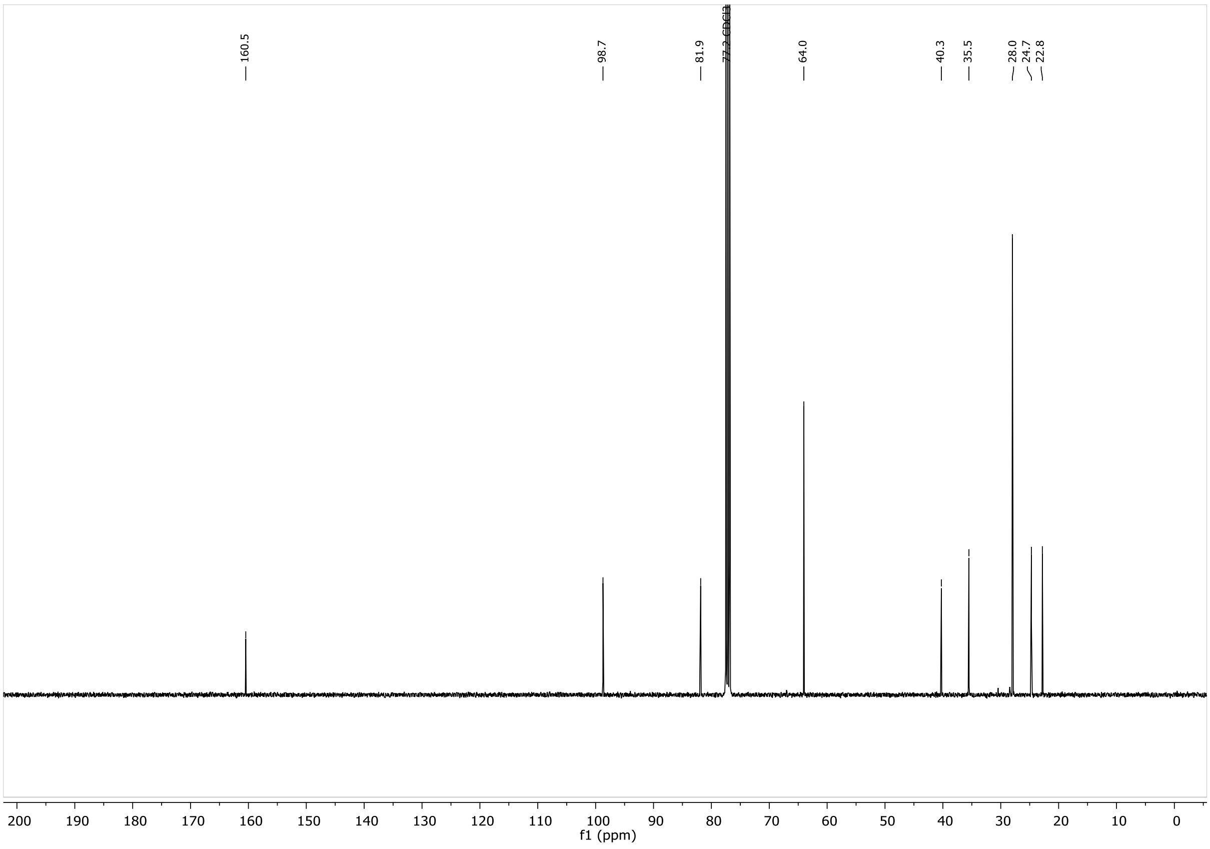

1-methyl-4-octylbenzene (13s)

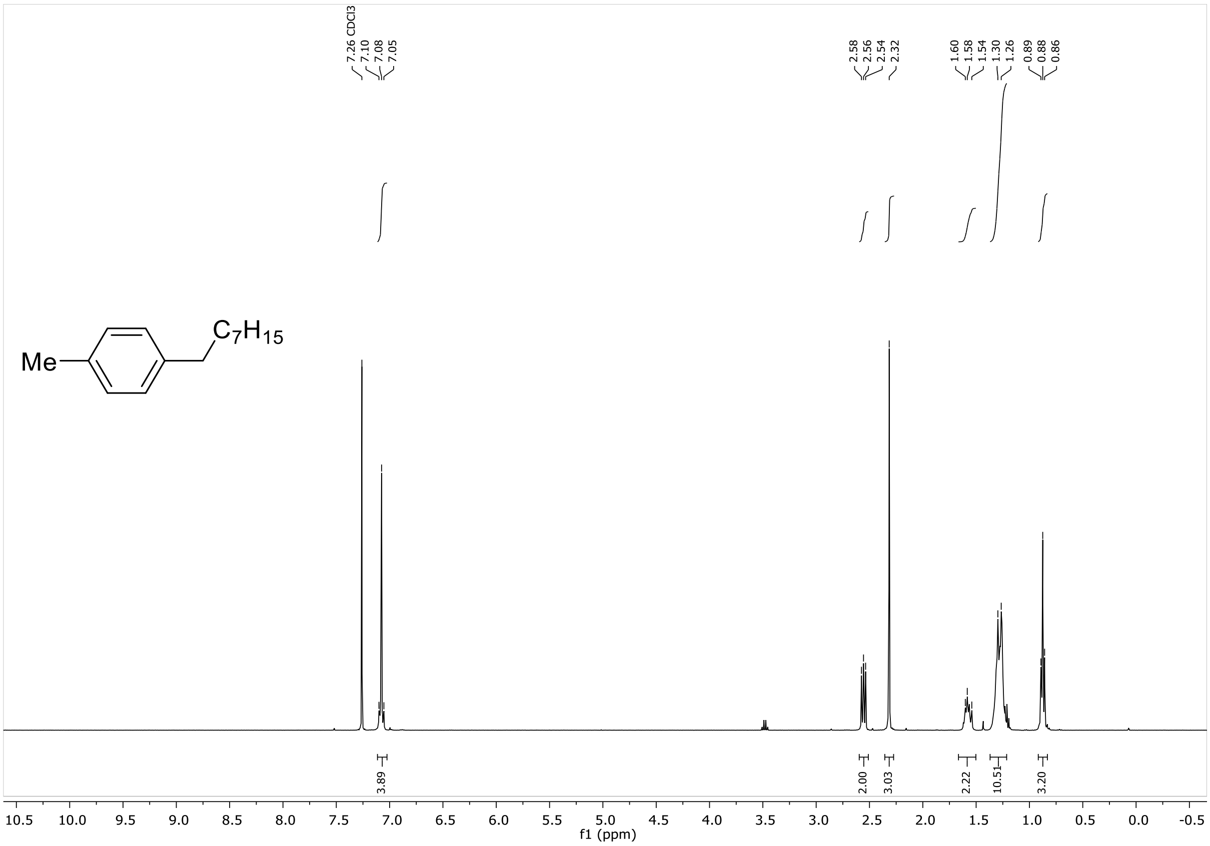

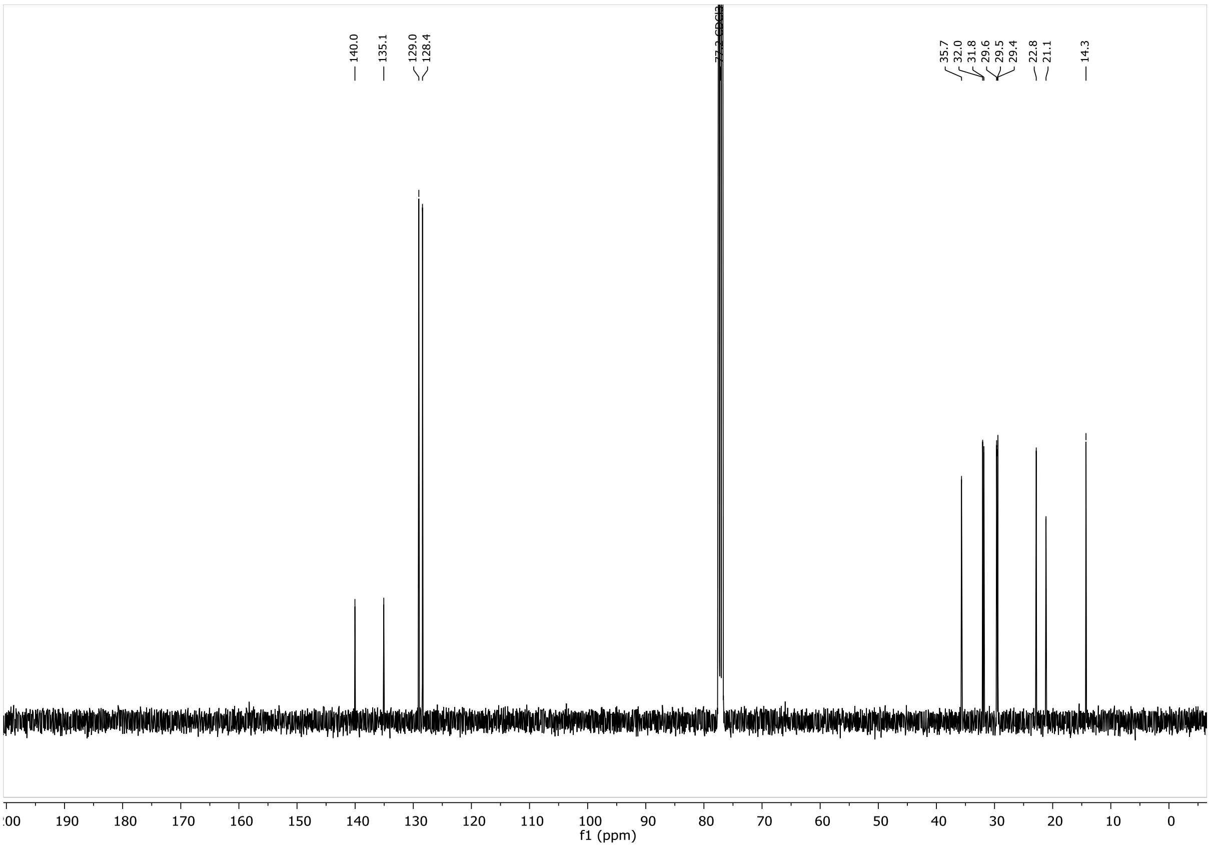

***tert*-butyl (2,2-dimethyl-5-(4-octylphenethyl)-1,3-dioxan-5-yl)carbamate (24)**

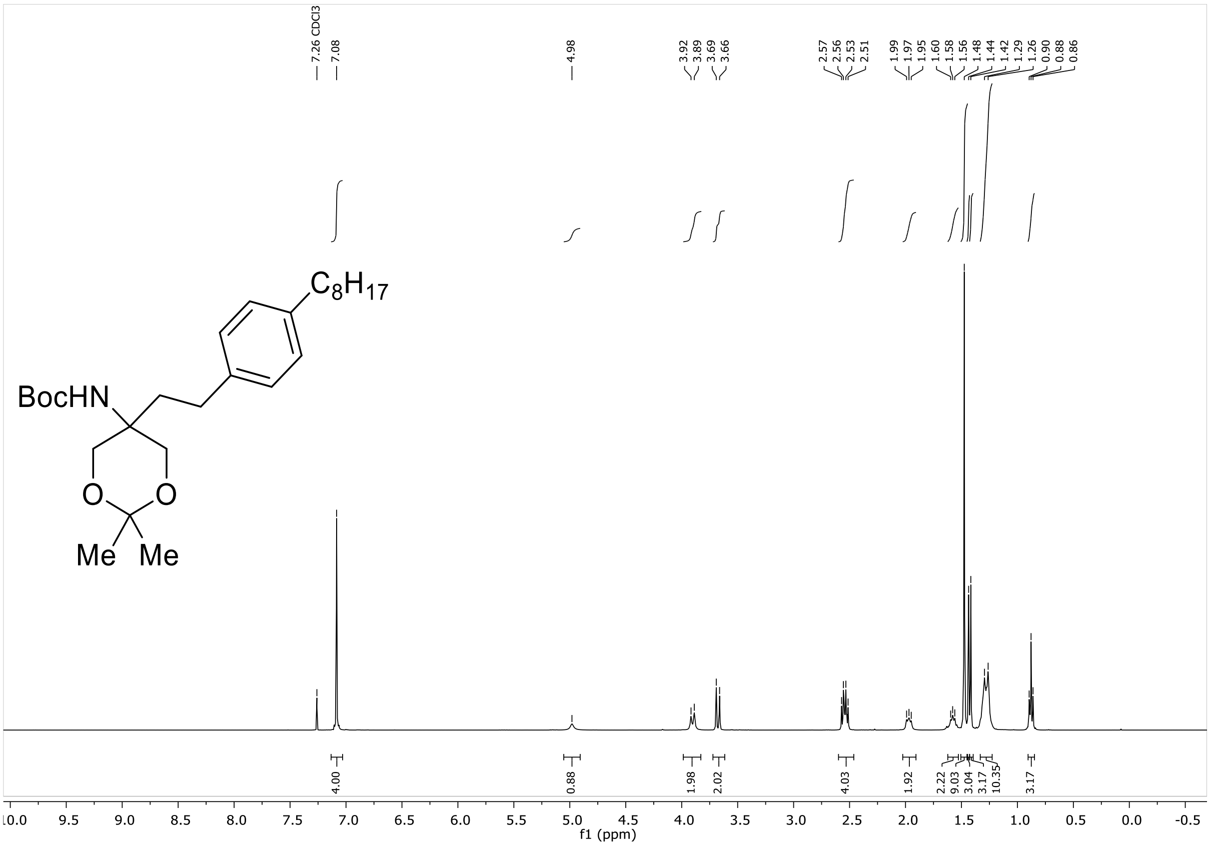

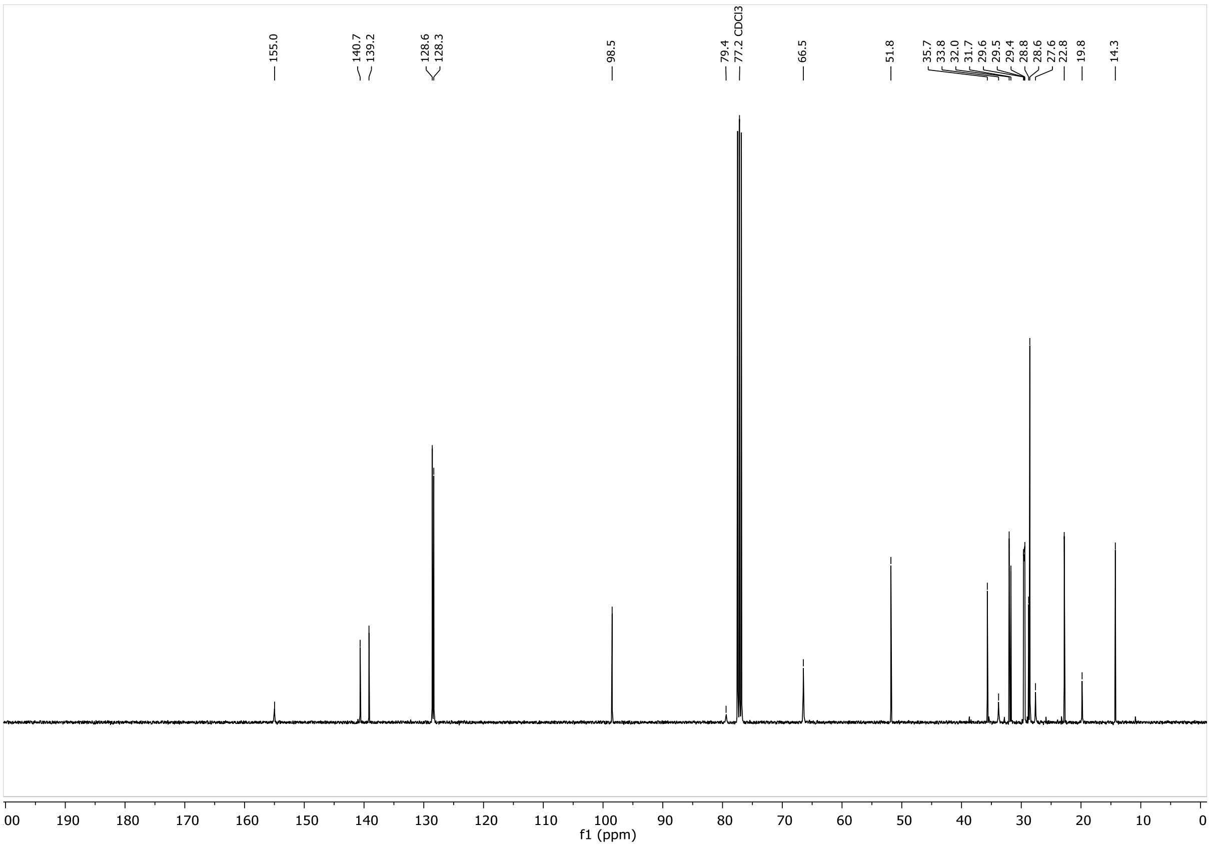

Fingolimod Hydrochloride (5)

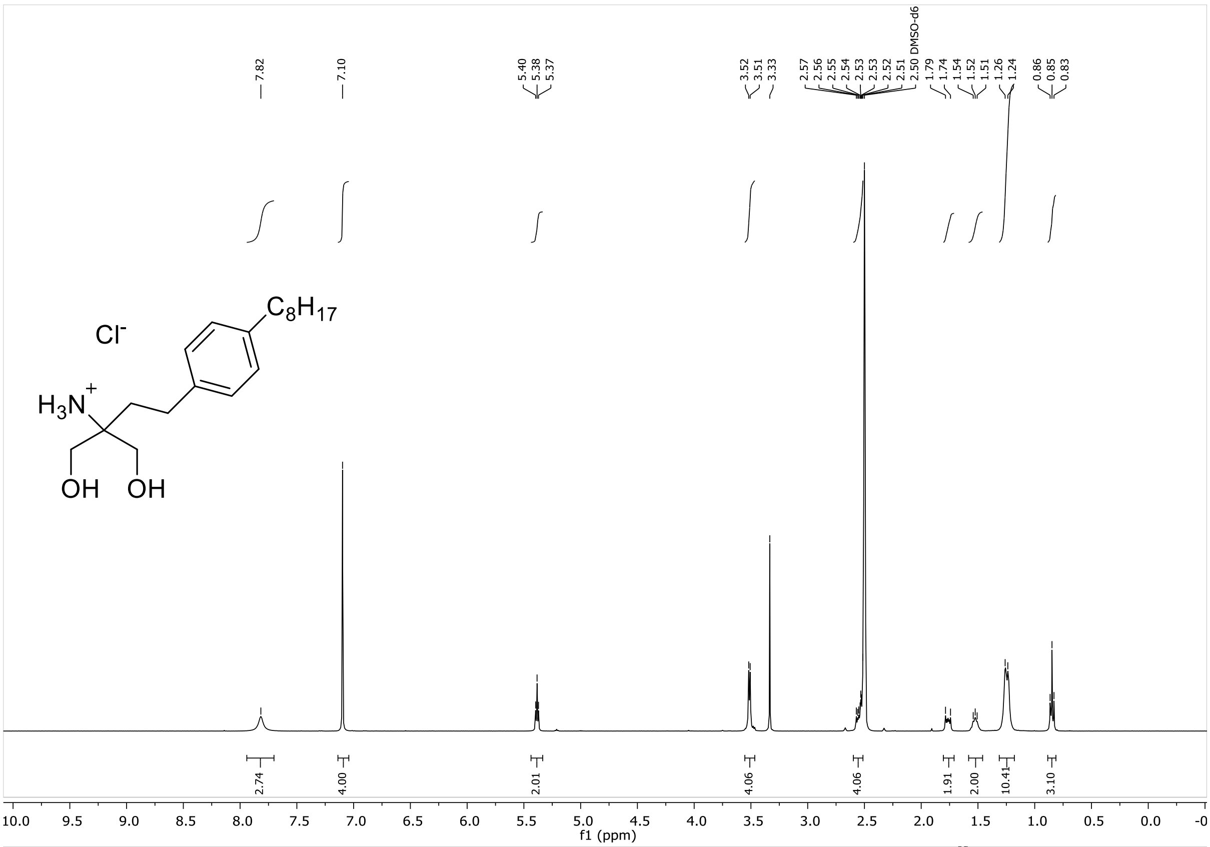

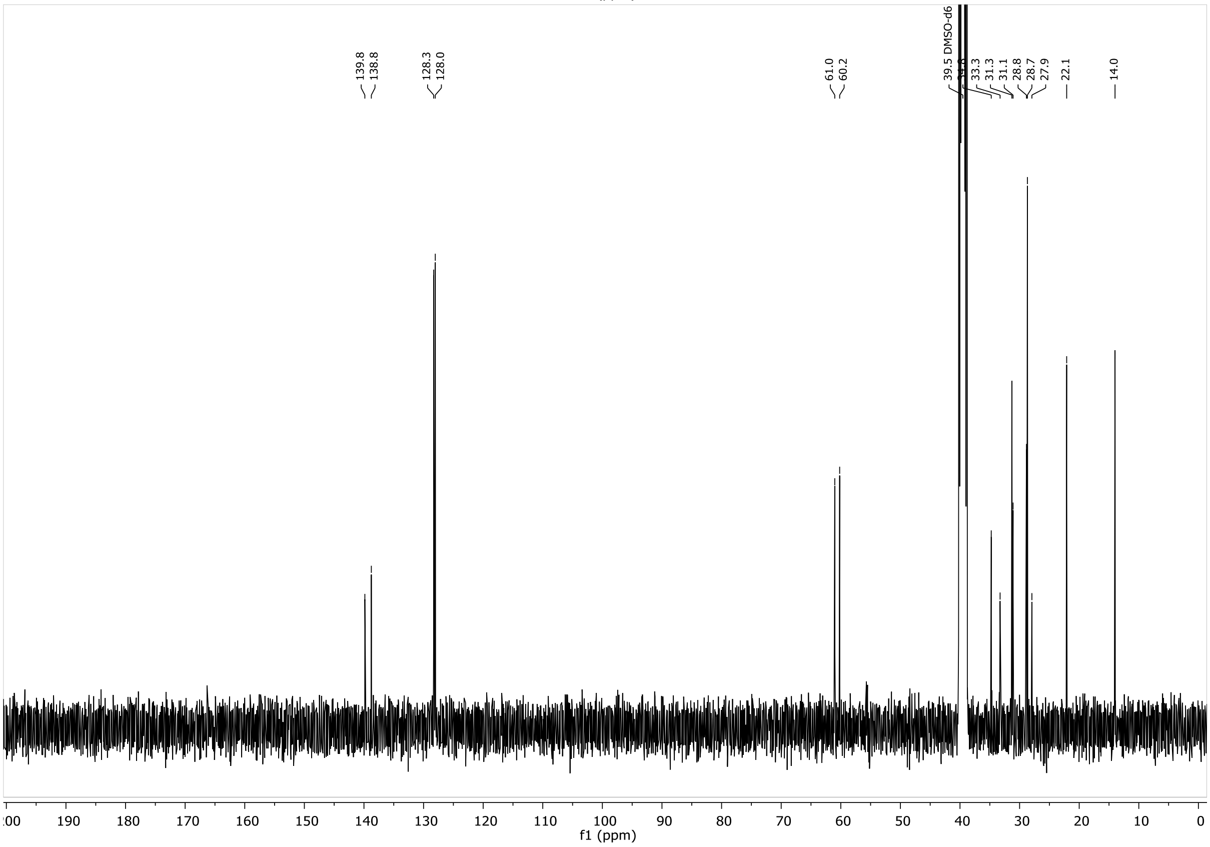

2-amino-1-(2,2-dimethyl-4*H*-benzo[*d*][1,3]dioxin-6-yl)ethan-1-ol (26)

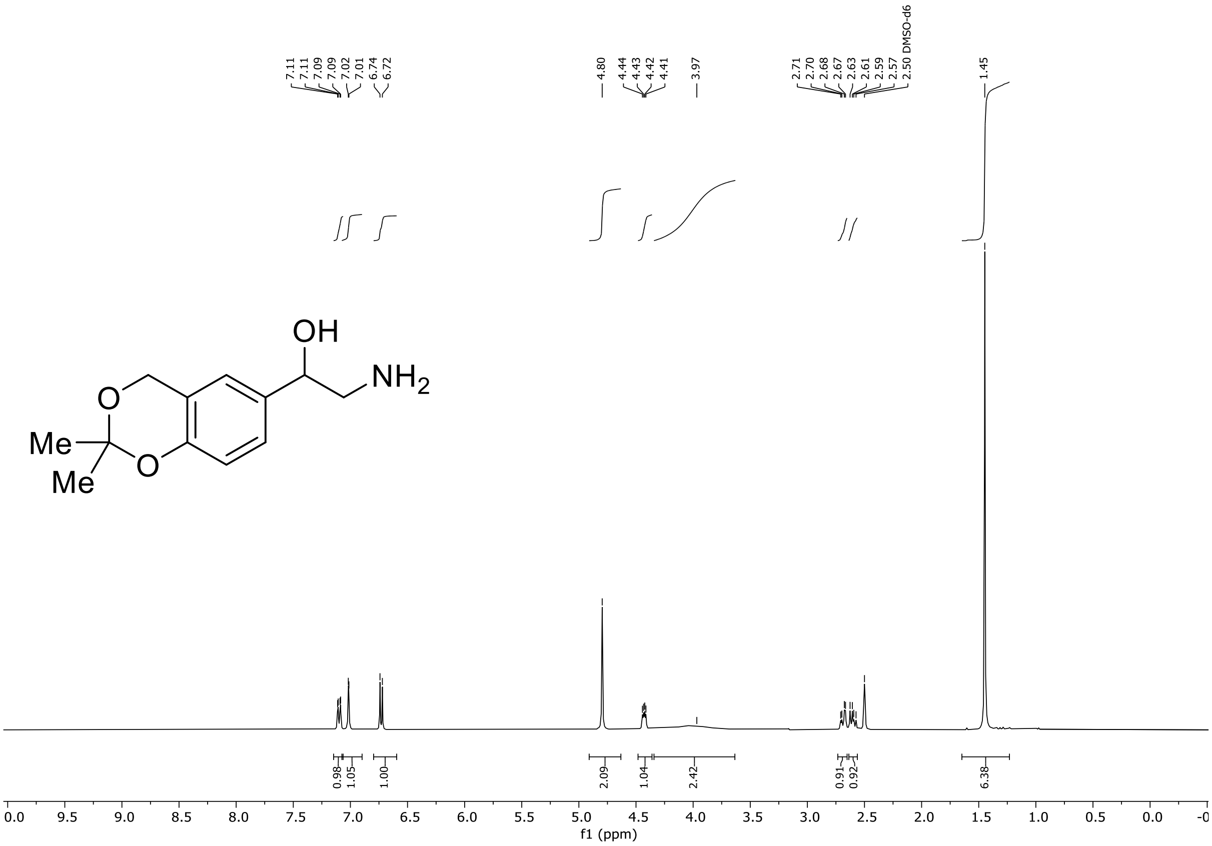

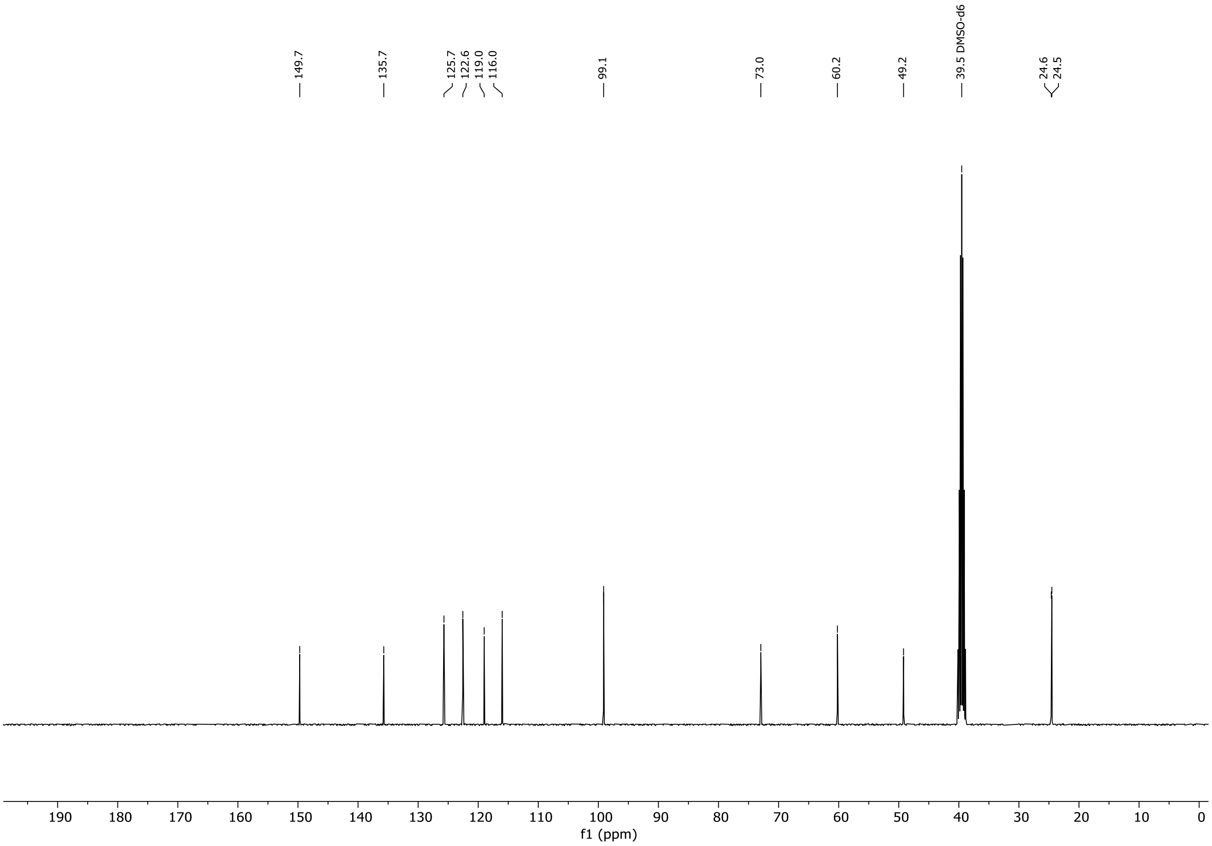

4-(phenyl-d_5)butan-4,4-d_2-1-ol (10au)

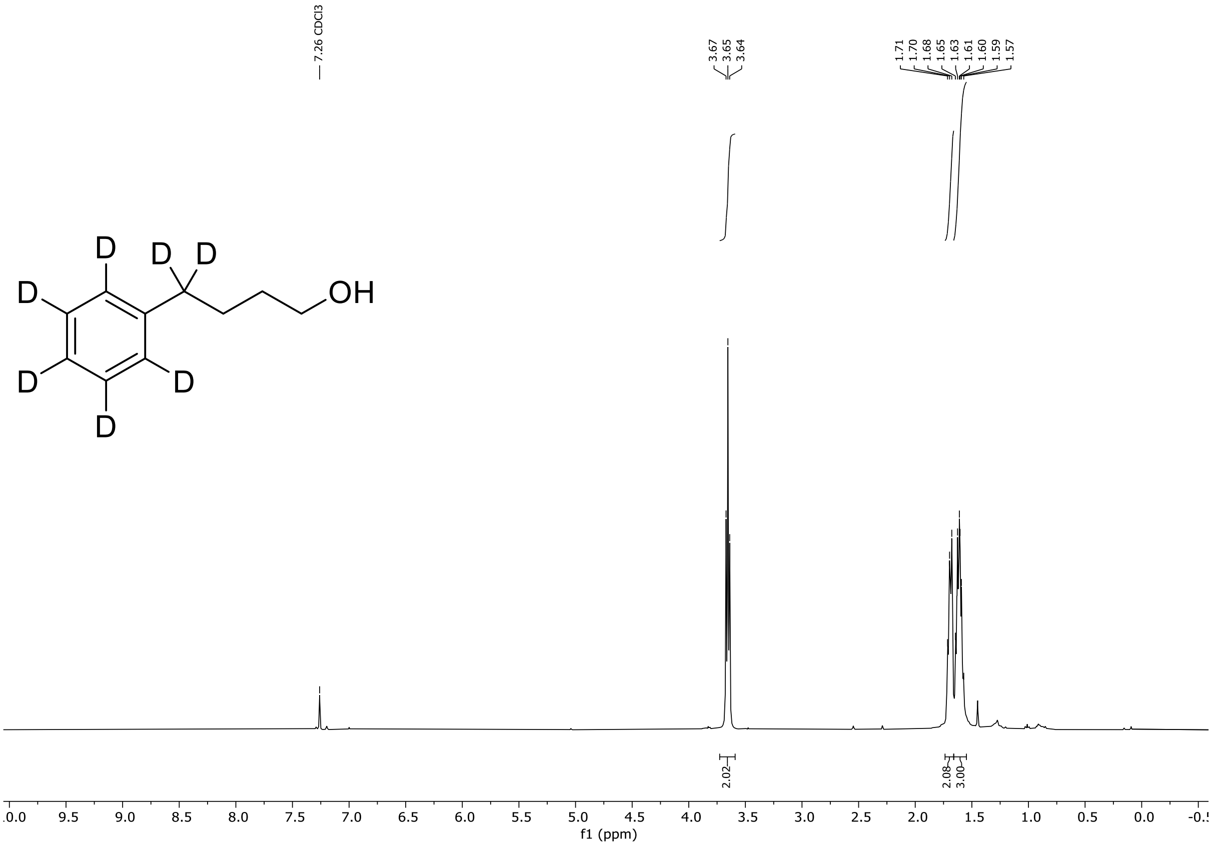

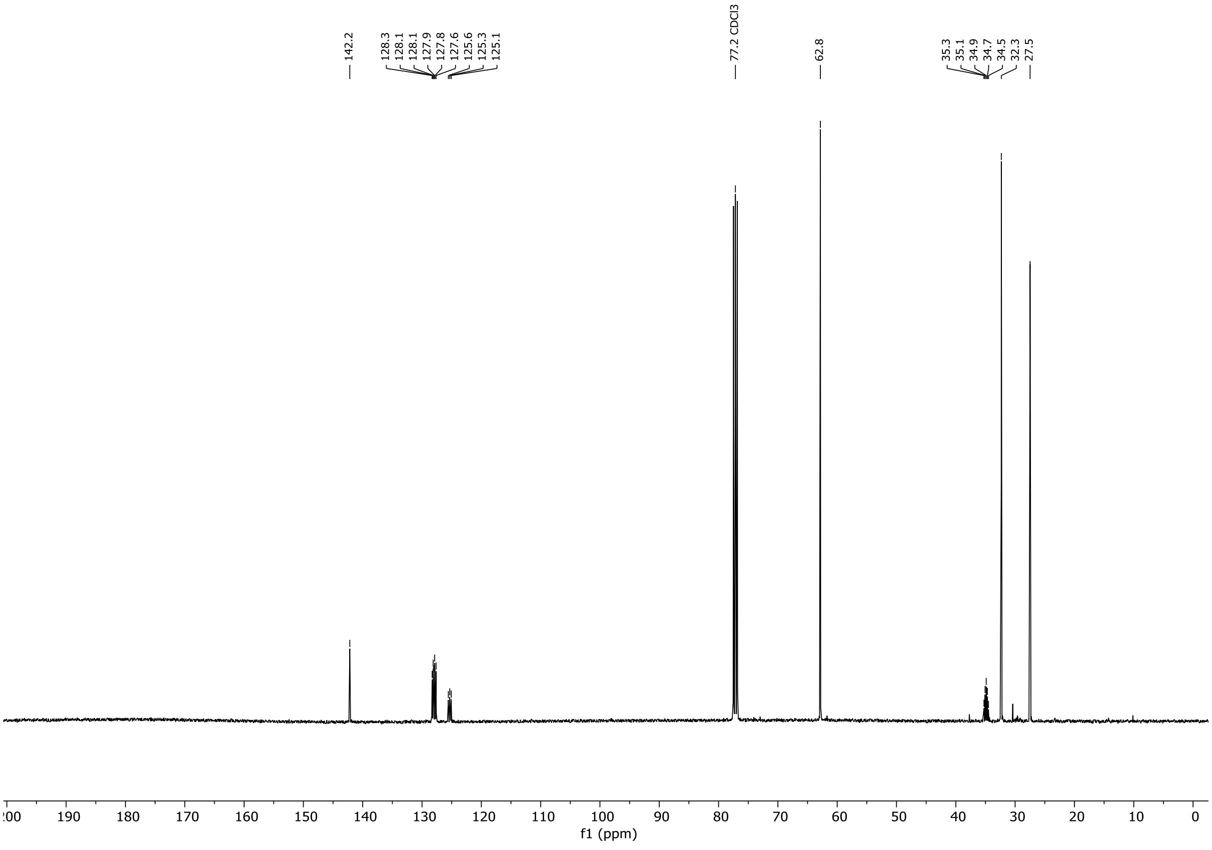

1-(4-((6-bromohexyl)oxy)butyl-1,1-d_2)benzene-2,3,4,5,6-d_5

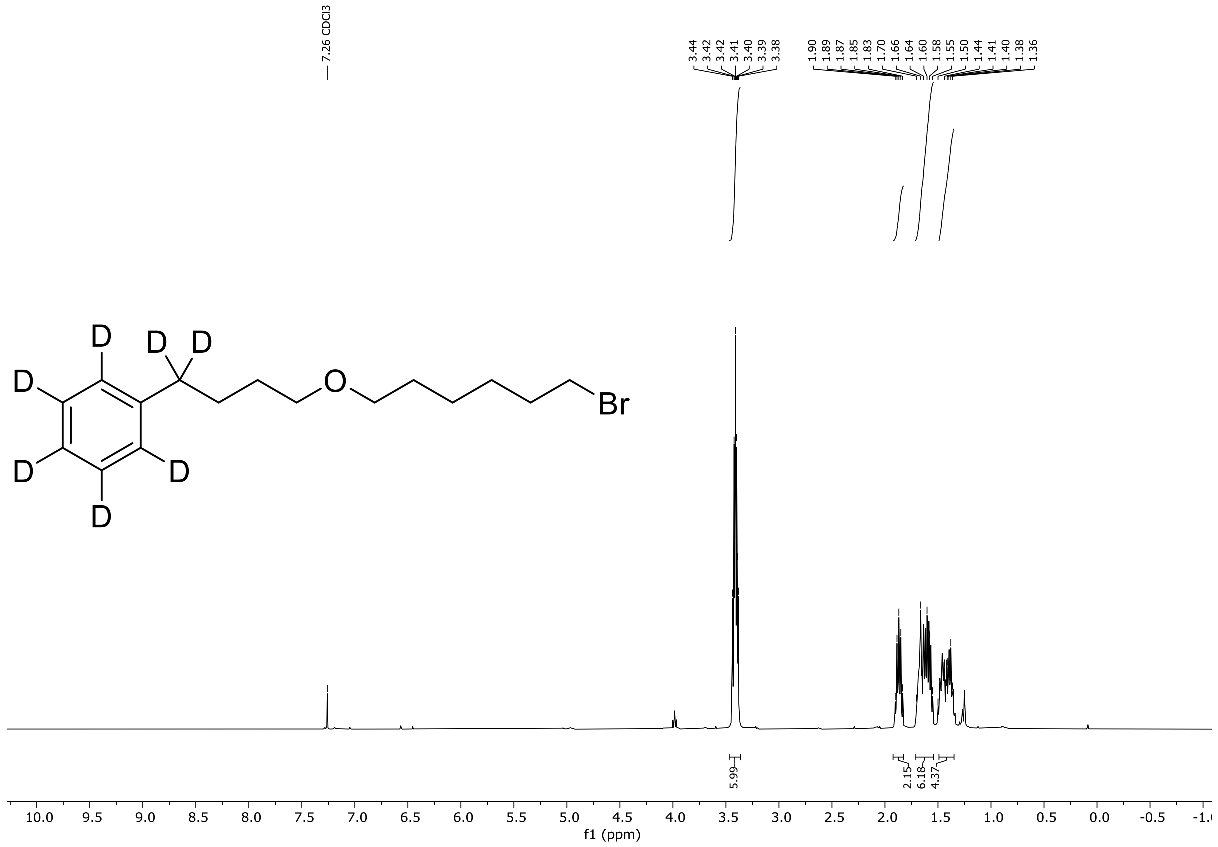

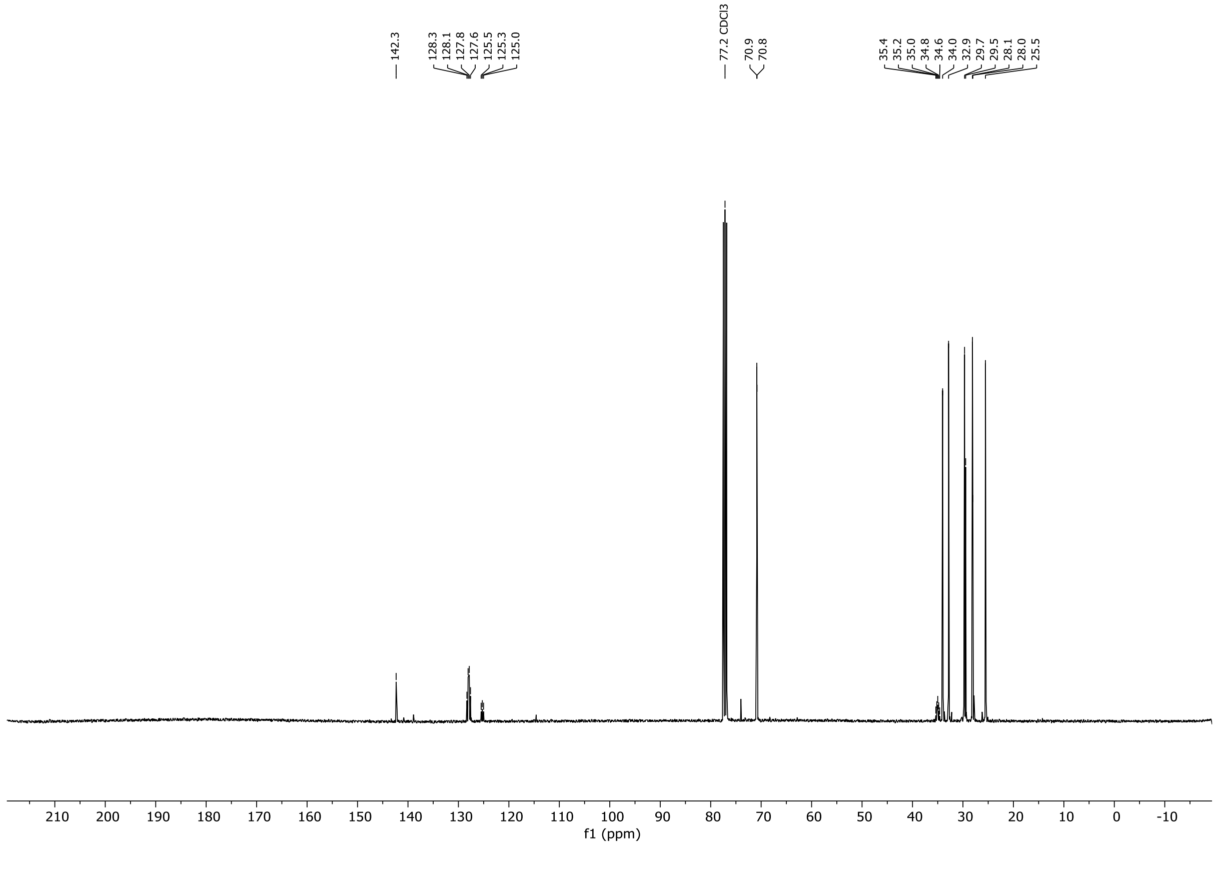

1-(2,2-dimethyl-4*H*-benzo[*d*][1,3]dioxin-6-yl)-((6-(4-(phenyl-d_5)butoxy-4,4-d_2)hexyl)amino)ethan-1-ol

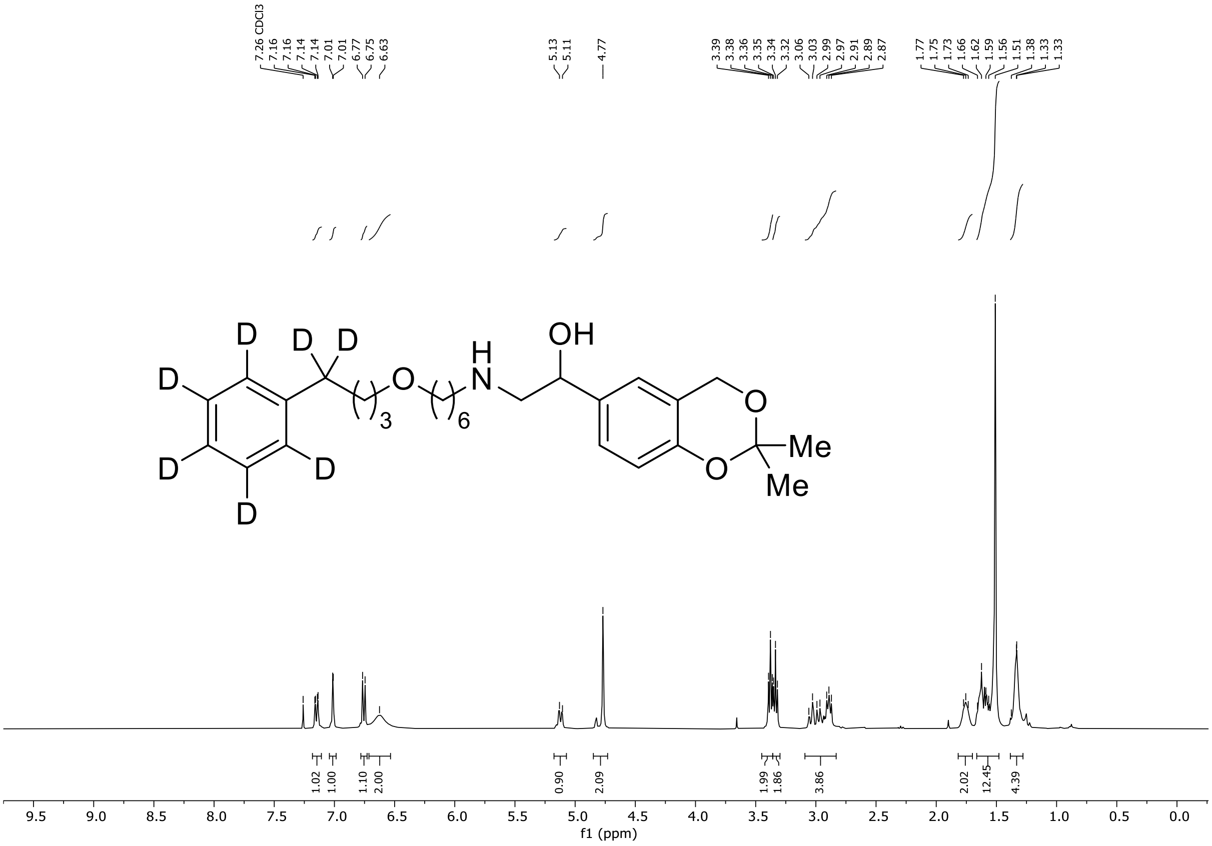

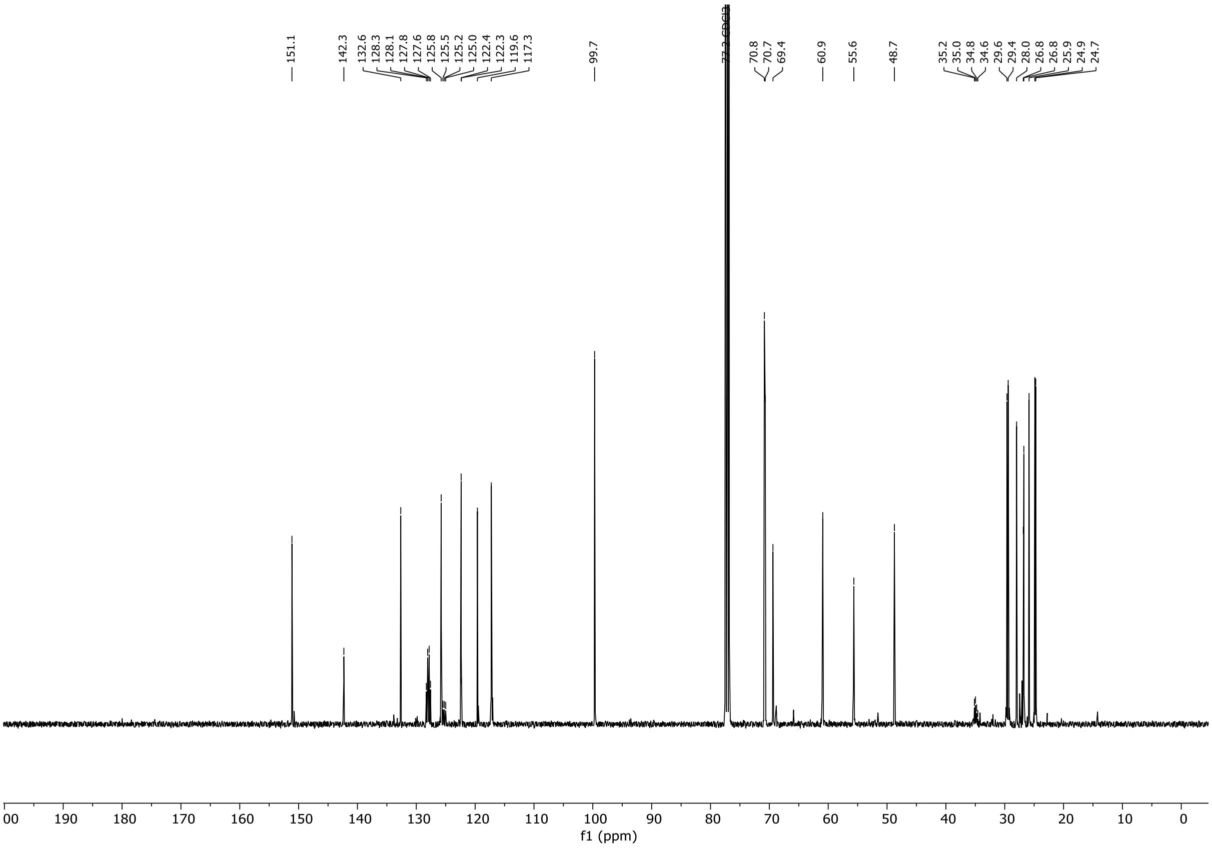

Salmeterol-d_7 (6)

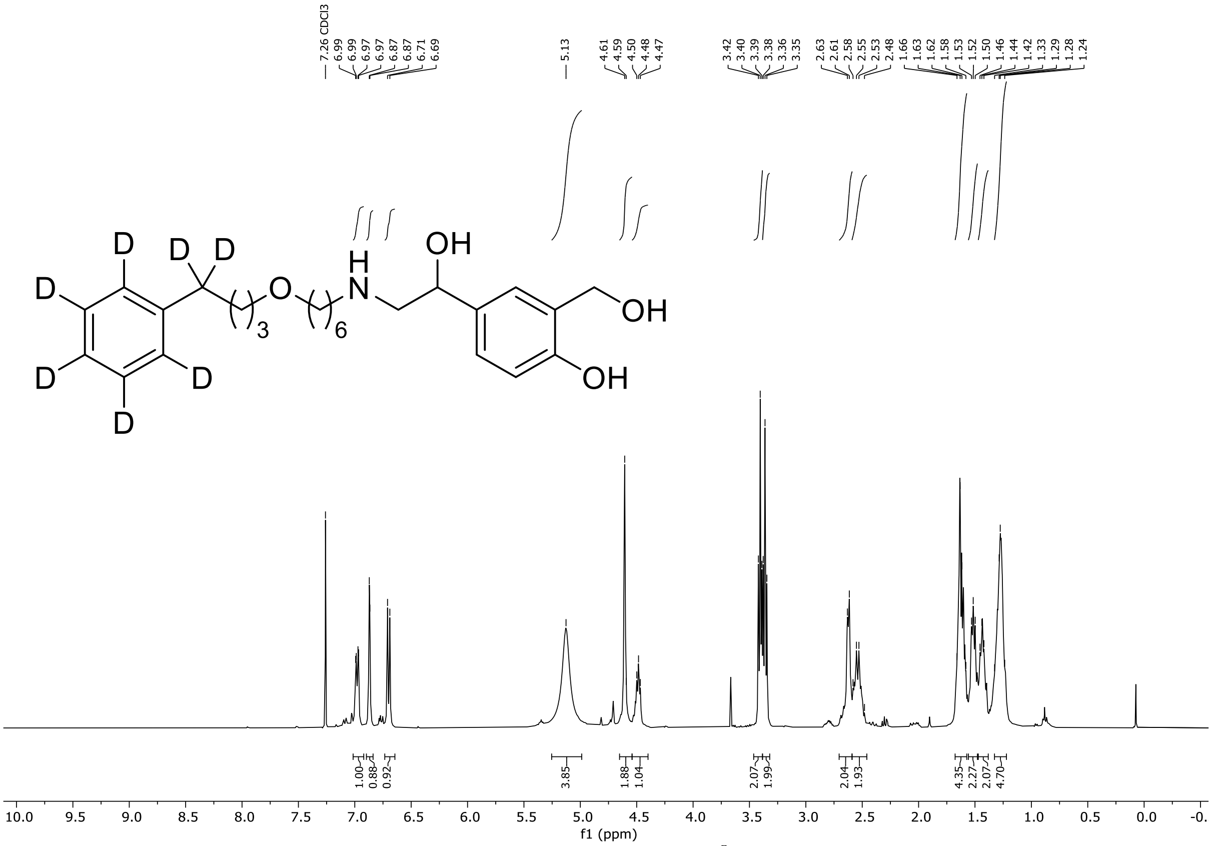

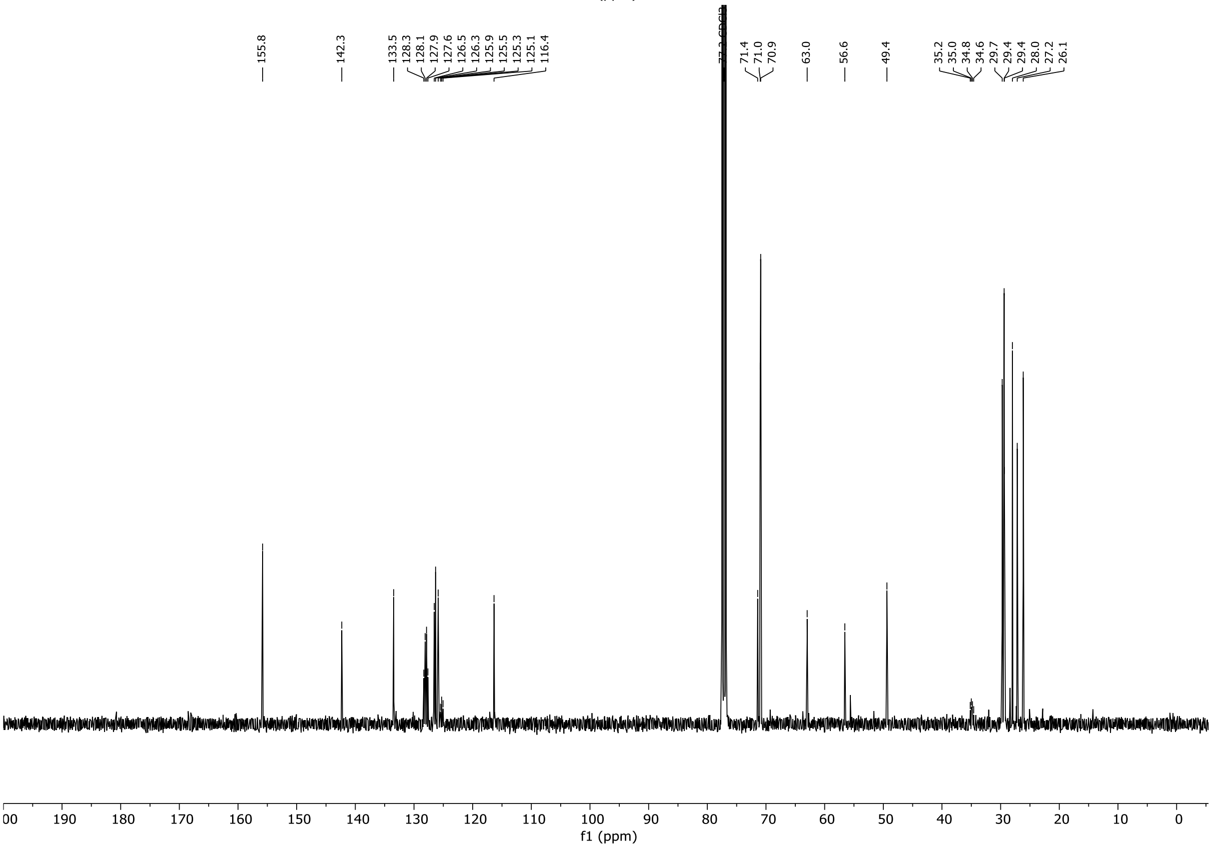

tridecylbenzene (13t)

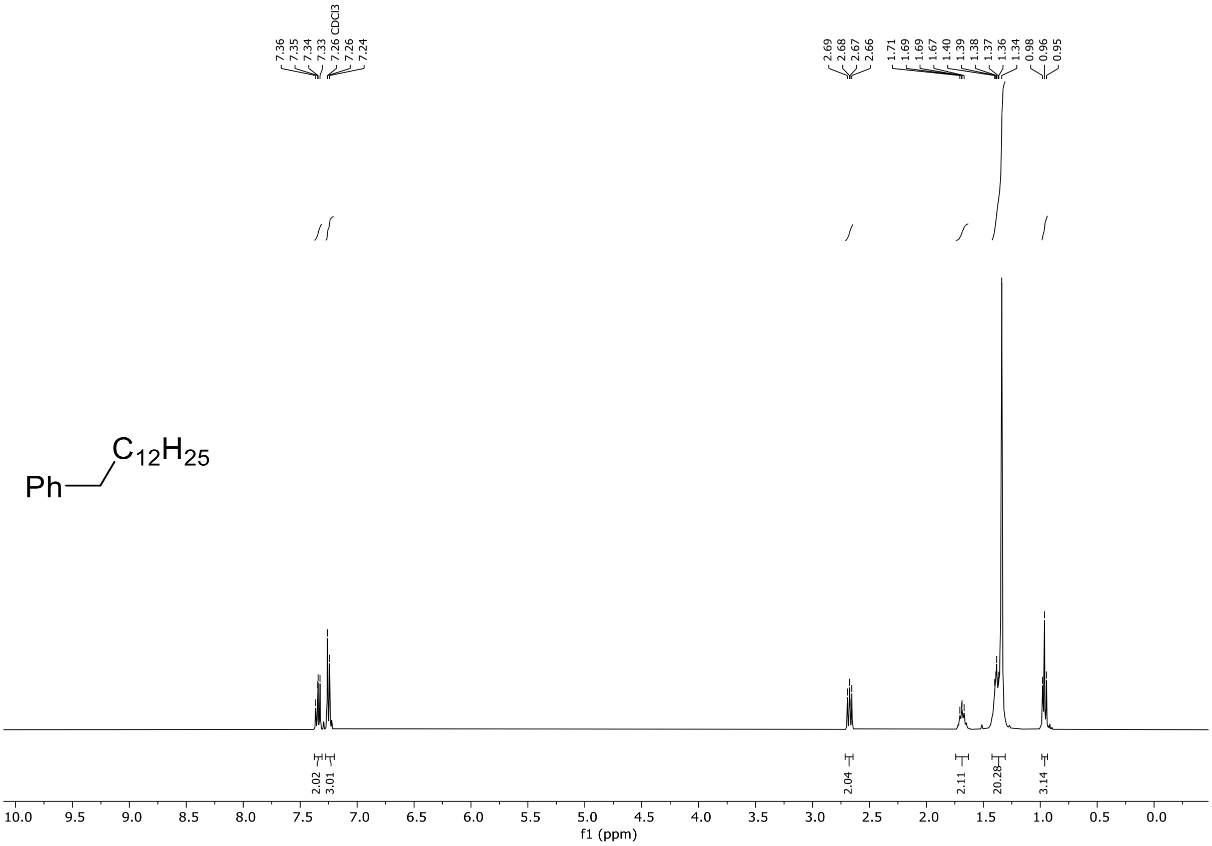

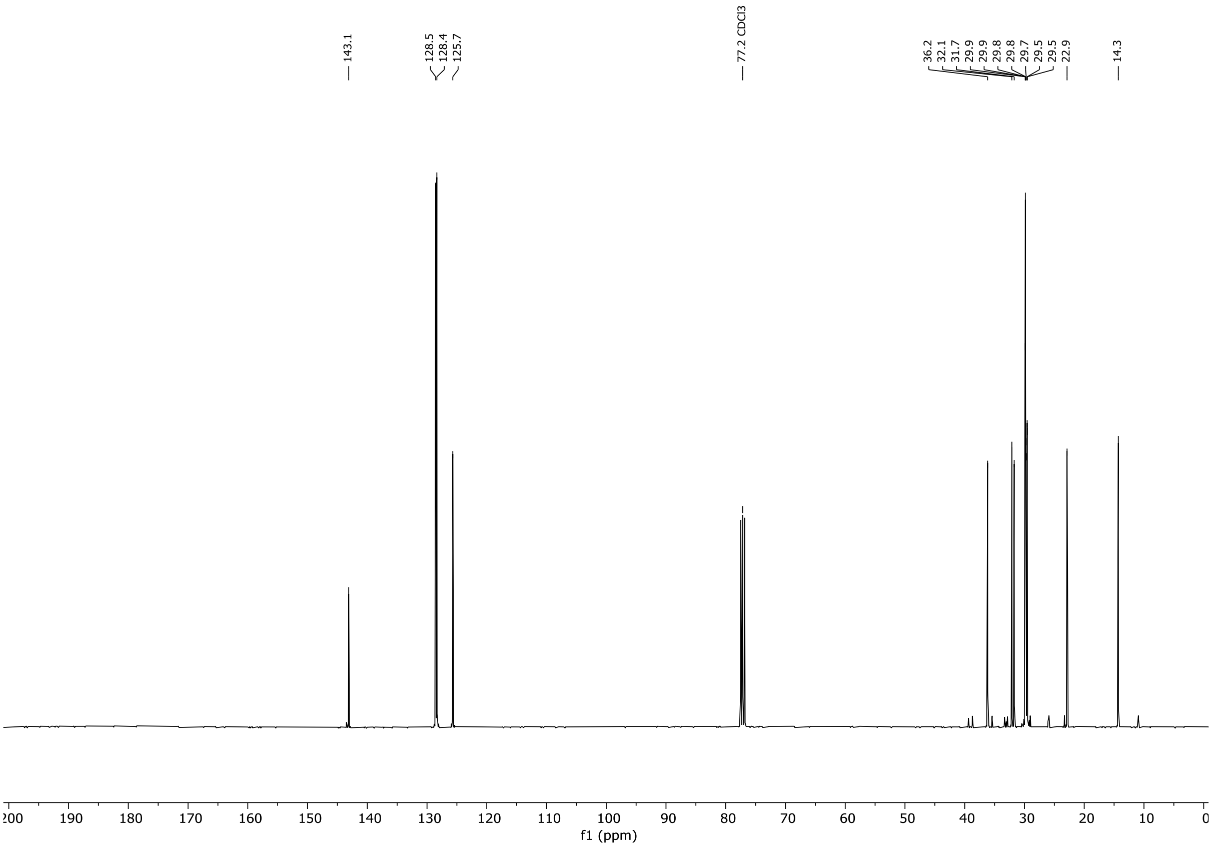

4-tridecylbenzenesulfonic acid (8)

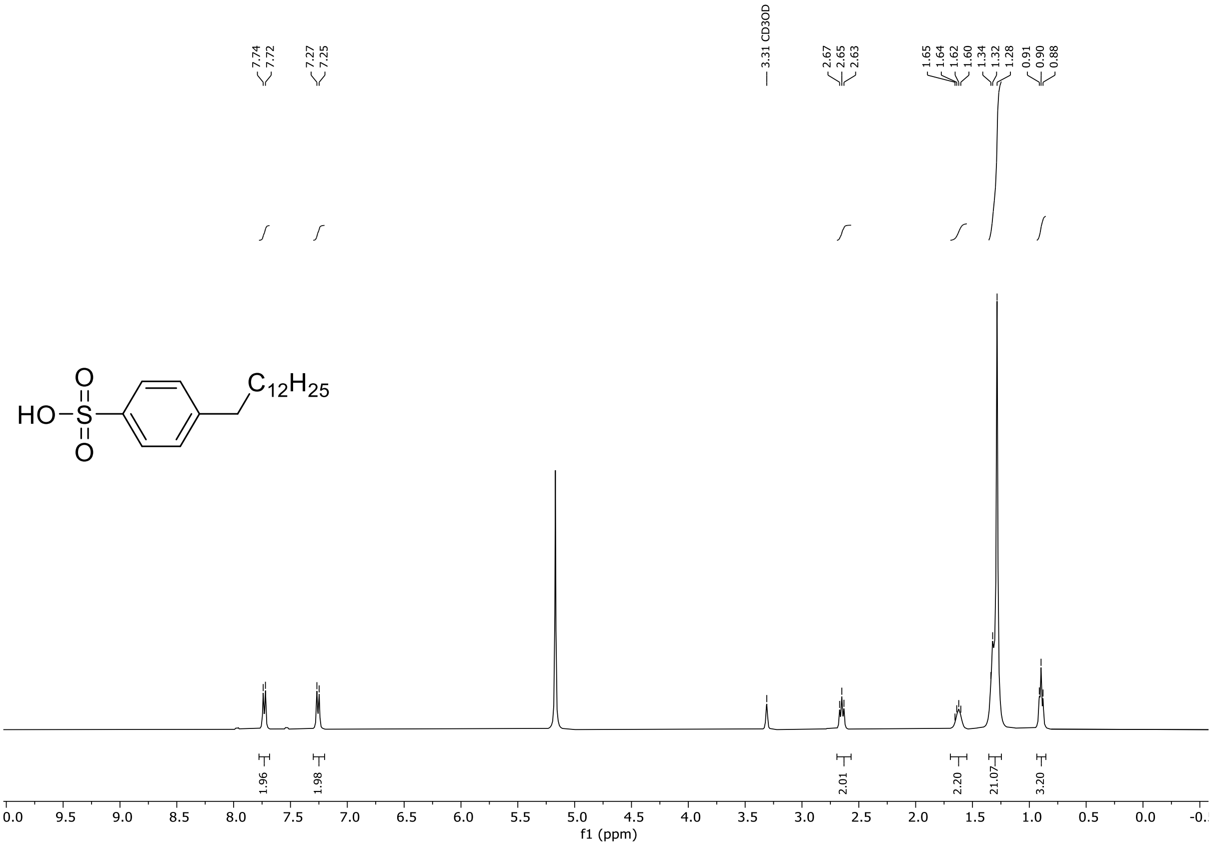

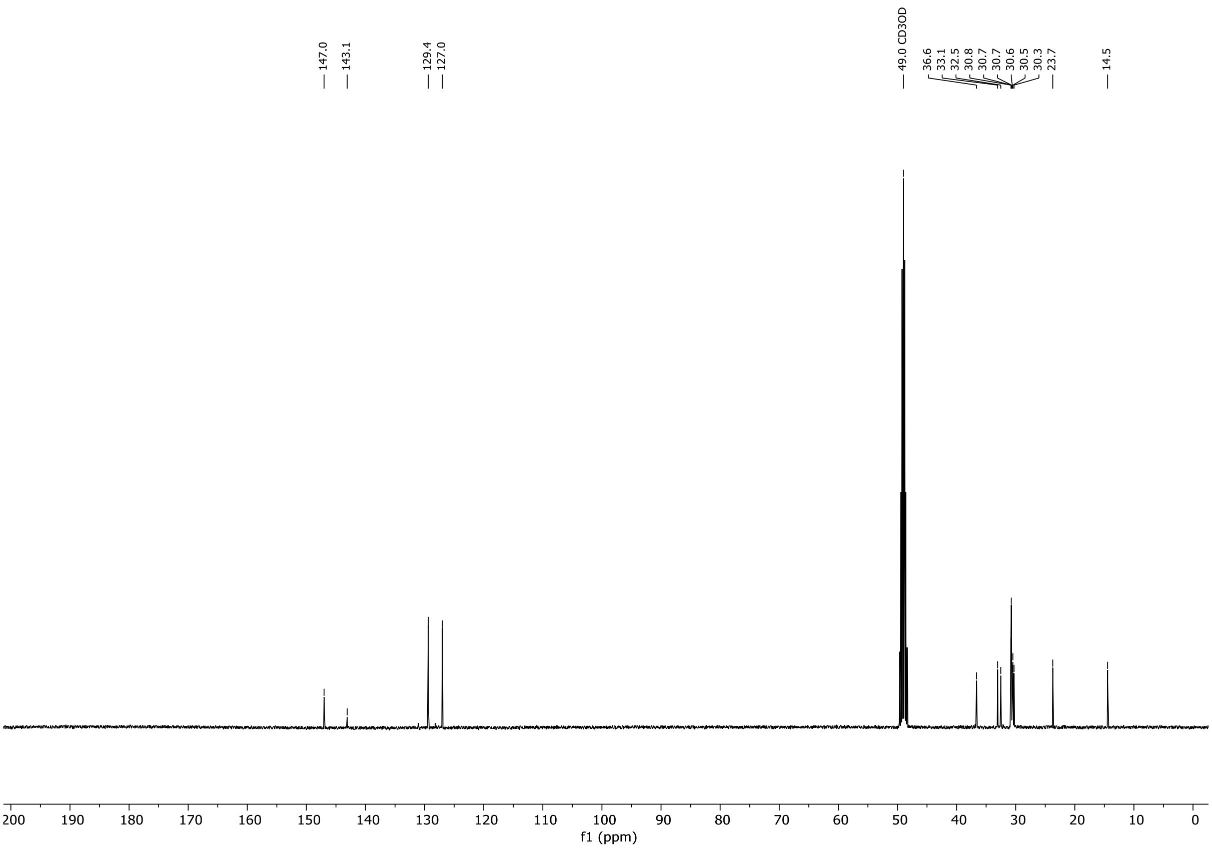

***trans*-4-((tert-butyldimethylsilyl)oxy)cyclohexyl 4-methylbenzenesulfonate (12n)**

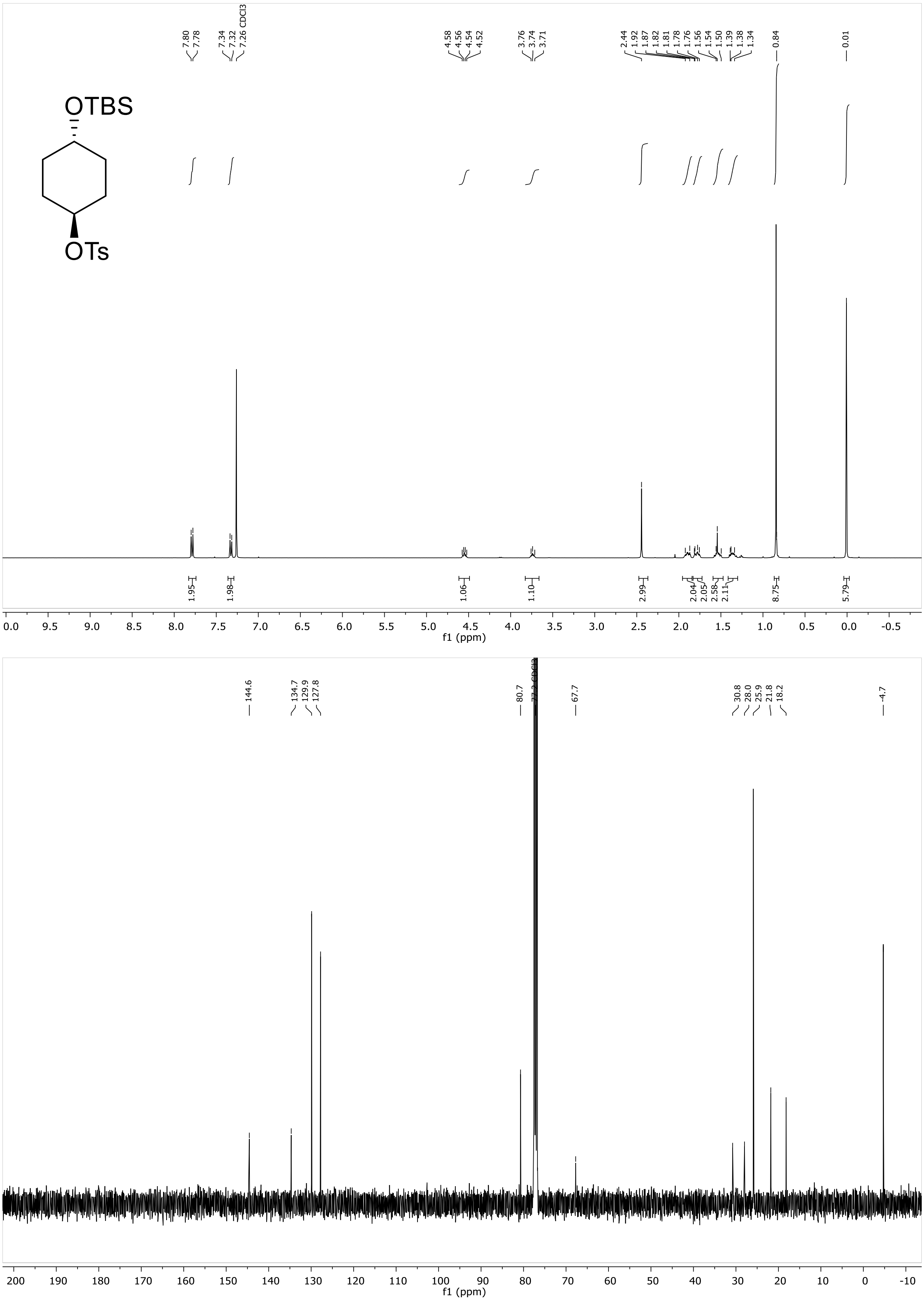

trans-4-(tert-butyl)cyclohexyl 4-methylbenzenesulfonate (12m)

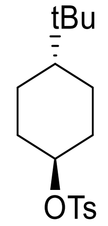

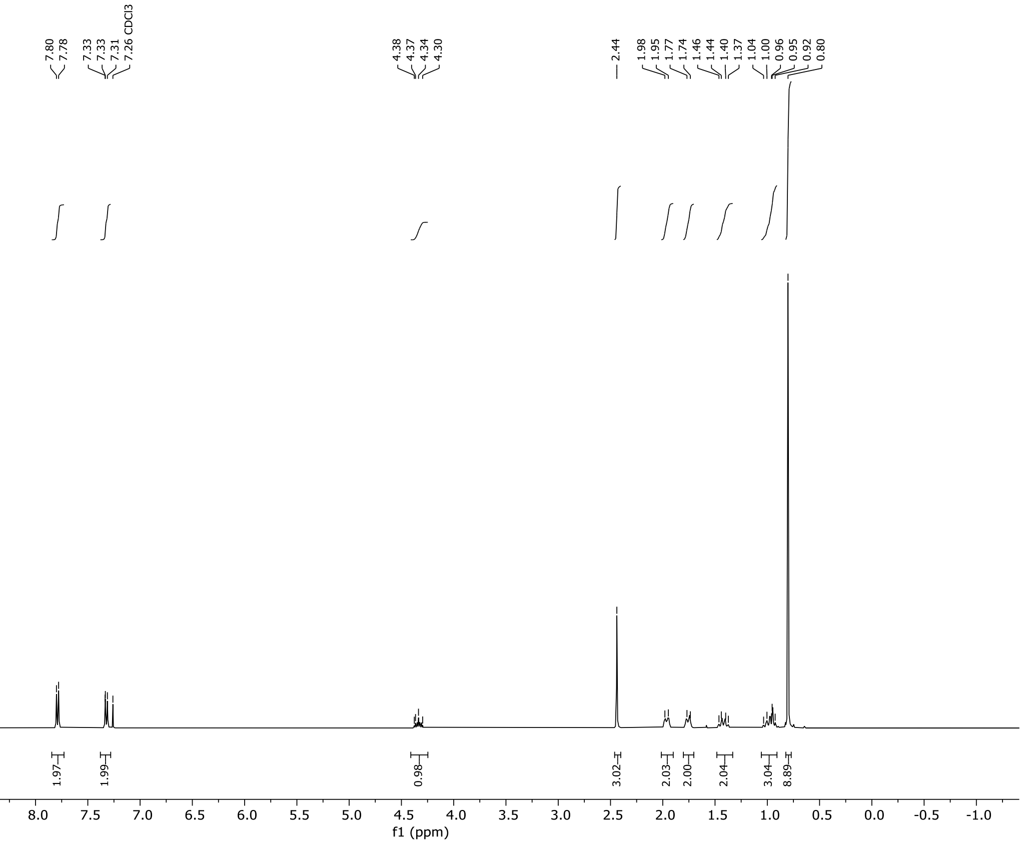

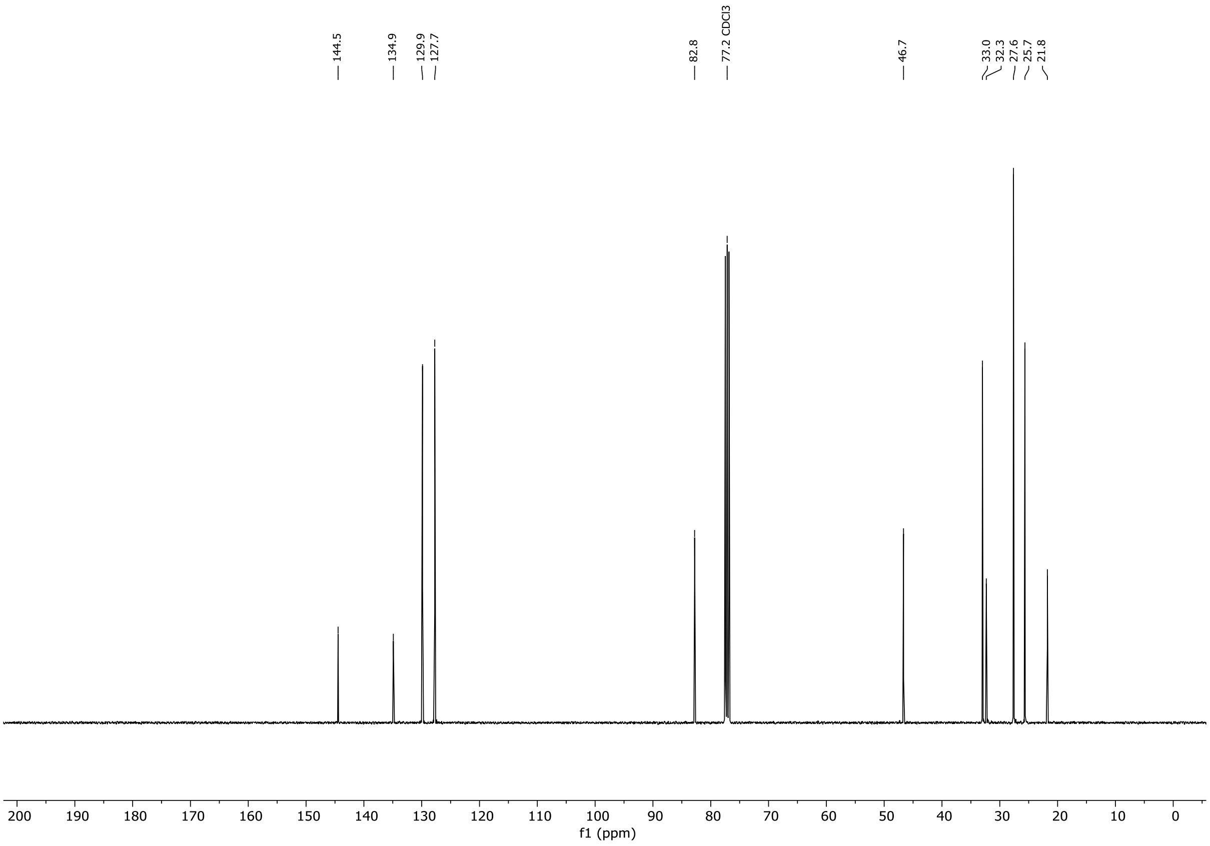

(1*R*,2*S*)-1-chloro-2-methoxycyclohexane (12d)

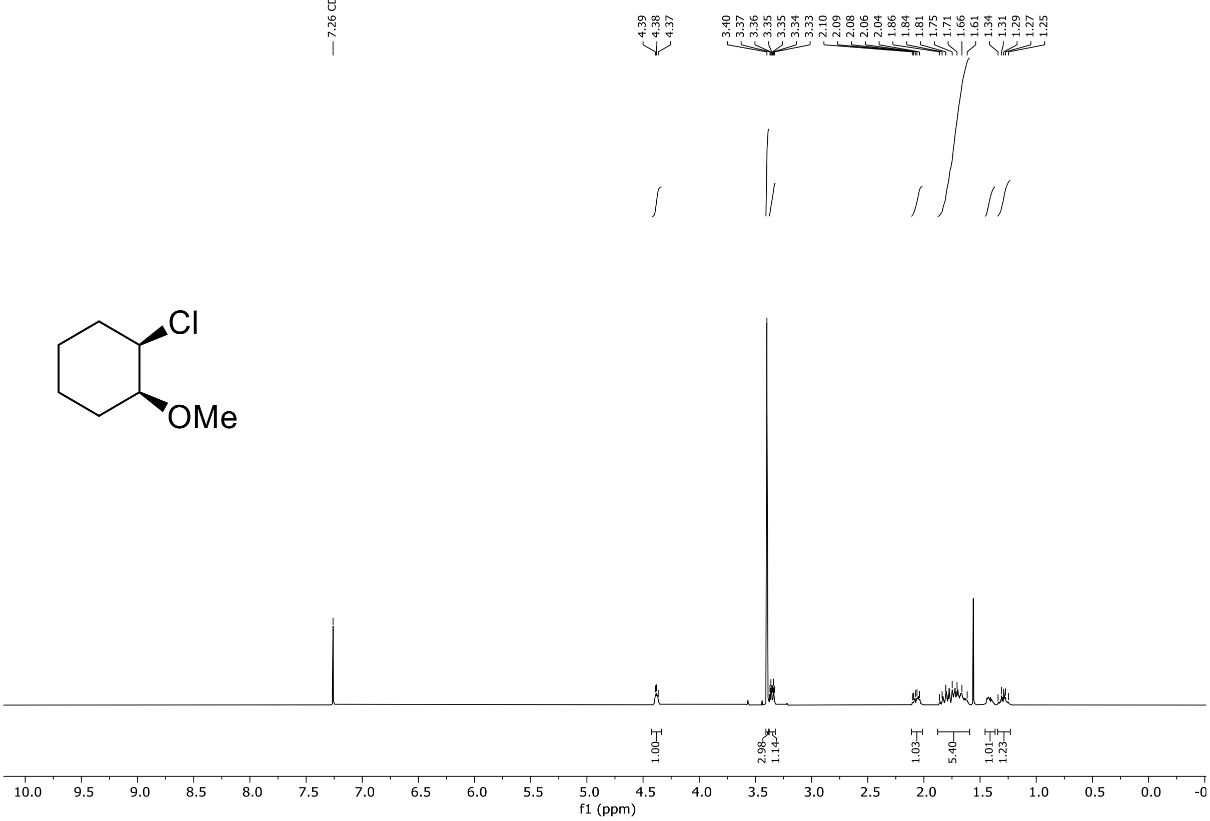

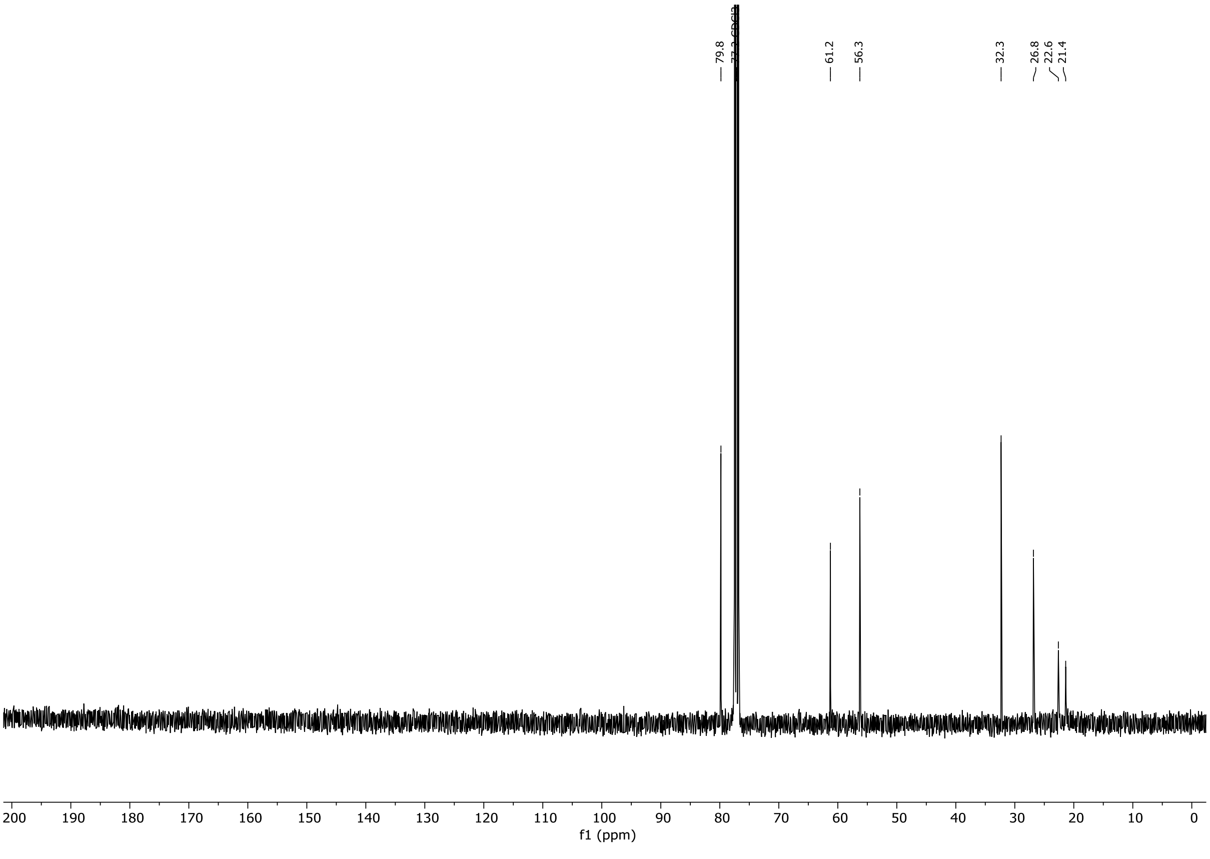

Supplement: Supplementary file 1 — Supporting Information [file ANIE-61-0-s001.pdf]
